# Cobalt-Catalyzed (3 + 2) Cycloaddition of Cyclopropene-Tethered Alkynes: Versatile Access to Bicyclic Cyclopentadienyl Systems and Their CpM Complexes

**DOI:** 10.1021/acscatal.4c03080

**Published:** 2024-07-20

**Authors:** Carlos Lázaro-Milla, Eduardo da Concepción, Israel Fernández, José L. Mascareñas, Fernando López

**Affiliations:** † Centro Singular de Investigación en Química Biolóxica e Materiais Moleculares (CiQUS) and Departamento de Química Orgánica, Universidade de Santiago de Compostela, 15782 Santiago de Compostela, Spain; ‡ Departamento de Química Orgánica I, Facultad de Ciencias Químicas, Universidad Complutense de Madrid, 28040 Madrid, Spain; § Misión Biológica de Galicia, Consejo Superior de Investigaciones Científicas (CSIC), 36080 Pontevedra, Spain

**Keywords:** cyclopropene, cycloaddition, cobalt, catalysis, cyclopentadienes, CpM catalysts

## Abstract

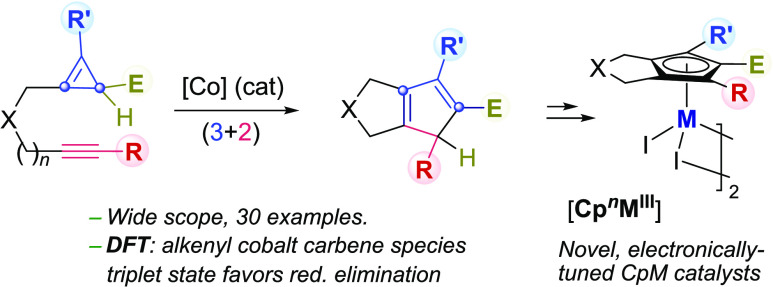

Low-valent cobalt complexes can promote intramolecular (3 + 2) cycloadditions of alkyne-tethered cyclopropenes to provide bicyclic systems containing highly substituted cyclopentadienyl moieties with electronically diverse functional groups. The adducts can be easily transformed into new types of CpRh(III) and CpIr(III) complexes, which show catalytic activity in several relevant transformations. Preliminary computational (DFT) and experimental studies provide relevant information on the mechanistic peculiarities of the cobalt-catalyzed process and allow us to rationalize its advantages over the homologous rhodium-promoted reaction.

## Introduction

Metal-catalyzed cycloadditions based on the activation of C–C bonds in strained systems are among the most practical and efficient methods to construct polycyclic products from simple materials.^1^ Most examples so far reported rely on precursors that combine a cyclopropyl unit with an external unsaturated handle for metal coordination, such as vinylcyclopropanes (VCPs) or alkylidenecyclopropanes (ACPs).^2^ In the case of ACPs, it is well established that the annulation is initiated by oxidative insertion of the metal complex into their proximal or distal C–C bond to give metallacyclobutanes that insert into unsaturated partners and eventually give formal (3 + *n*) cycloadducts.^2^

Curiously, while this type of metal-catalyzed cycloadditions of ACPs have been extensively developed, related processes with cyclopropenes (CPEs), which are more strained, and present an *endo*- instead of an *exo*-cyclic double bond, are much more scarce.^3^ This is likely due to their high propensity to generate carbene intermediates, which tend to evolve through cyclopropanations and C–H insertions, among other pathways.^3a^ Indeed, metal-catalyzed (3 + *n*) annulations of cyclopropenes are limited to a handful of processes, most of them promoted by precious metals like Rh, Pd, or Ru.^4,5^

In light of our recent findings that cobalt catalysts can promote cycloadditions with ACPs,^6,7^ through different mechanisms than those promoted by their palladium and rhodium counterparts, we wondered about the potential of this metal in the catalytic cycloaddition chemistry of CPEs.

**Scheme 1:**
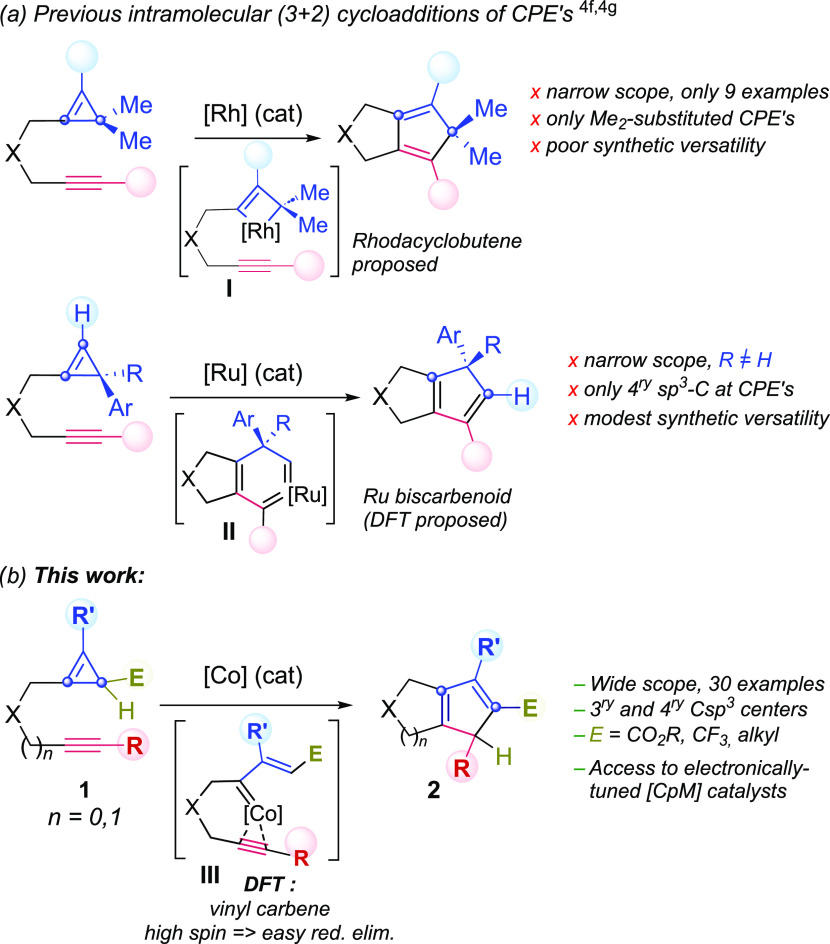
Previous Metal-Catalyzed (3 + 2) Cycloadditions of CPEs and This Work

Herein, we report the discovery that alkynyl-tethered cyclopropenes (**1**) undergo a smooth formal (3 + 2) cycloaddition to yield a wide variety of appealing cyclopentadienyl bicyclic products (**2**, Scheme 1b). Compared to all previously reported related annulations of CPEs (Scheme 1a),^4f,4g^ the current method presents a distinctive and much broader scope, as it is not restricted to the use of CPEs bearing fully substituted C(sp^3^) centers (Scheme 1c). From a mechanistic perspective, density functional theory (DFT) studies confirm that our cobalt catalyst follows a path involving alkenyl cobalt carbene species (**III**), rather than metalacyclobutene (**I**) or metaladicarbenoids (**II**), as proposed for Rh and Ru-catalyzed related processes^4f,4g^ (Scheme 1a). More importantly, the superiority of cobalt with respect to similar rhodium catalysts can be explained in terms of the propensity of this metal to transition between singlet and triplet states, which eventually enables lower energy barriers, especially for the reductive elimination.

Finally, we also demonstrate that the generated cyclopentadienyl adducts can be easily transformed into bicyclic η^5^-cycylopentadienyl metal(III) complexes that bear a wide variety of electronically withdrawing substituents at the Cp ring, a type of complexes that cannot be prepared by alternative methods.^8^ Notably, these “CpRh” and “CpIr” complexes are active in several relevant catalytic transformations.

## Results and Discussion

We began our studies with the alkynyl-tethered cyclopropane **1a**, a substrate that can be efficiently obtained through a single cyclopropenation reaction from abis-propargyl tosyl amide precursor and ethyl diazoacetate.^9^ Gratifyingly, treatment of a solution of this precursor in 1,2-DCE at 80 °C with the low-valent cobalt complex generated in situ from CoBr_2_ (10 mol %), dppp (12 mol %), indium (50 mol %) and NaBAr^F^_4_ (10 mol %), led to the bicyclic cycloadduct **2a**, in an excellent 90% yield (Table 1, entry 1). Mechanistically interesting, the sp^3^-carbon of the cyclopropene moiety of **1a** ends up as a sp^2^-center in **2a**, suggesting that a hydrogen shift had occurred throughout or after the cycloaddition process.

**Table 1:**
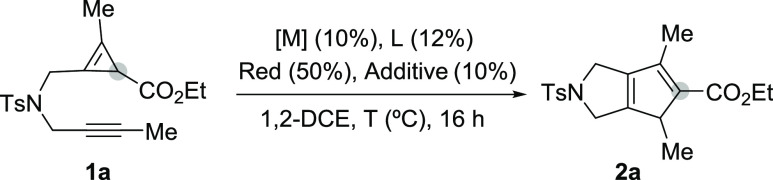
Initial Evaluation of the (3 + 2) Intramolecular Cycloaddition of **1a**^a^

entry	[M]	red	L	additive	*T* (°C)	yield (%)
1	CoBr_2_	In	dppp	NaBAr^F^_4_	80	90
2	CoBr_2_	In	dppp	NaBAr^F^_4_	70	66^b^
3	CoBr_2_	In	dppp	NaBAr^F^_4_	110	80
4	CoBr_2_	Zn	dppp	NaBAr^F^_4_	80	72
5	CoBr_2_	Zn	dppp	ZnBr_2_	80	57
6	CoBr_2_	Zn	dppp	ZnI_2_	80	31
7	CoBr_2_	In	dppe	NaBAr^F^_4_	80	30
8	CoBr_2_	In	dppf	NaBAr^F^_4_	80	61
9	CoBr_2_	In	*rac*-Binap	NaBAr^F^_4_	80	43
10^c^	CoBr_2_	In	dppp	NaBAr^F^_4_	80	91
11^d^	CoBr_2_	In	dppp	NaBAr^F^_4_	80	80
12^e^	RhCl(PPh_3_)_3_	-		-	100	-f
13^g^	[Rh(cod)_2_]BF_4_	-	*rac*-Binap	-	100	-f
14^g^	Cp*RuCl(cod)	-		-		-f

a *Conditions*: A solution of **1a**, [M] (10 mol %), **L** (12 mol %), reductant (Red, 50 mol %) and additive (10 mol %), in 1,2-DCE, was heated for 16 h at the indicated temperature. Full conversion of **1a** unless otherwise noted.

b 83% conversion.

c Carried out at 1 mmol scale.

d Carried out with CoBr_2_ (5%), dppp (6%), In (25%), and NaBAr^F^_4_ (5%).

e Carried out in toluene.

f A complex mixture of products was observed.

g Carried out in PhCF_3_.

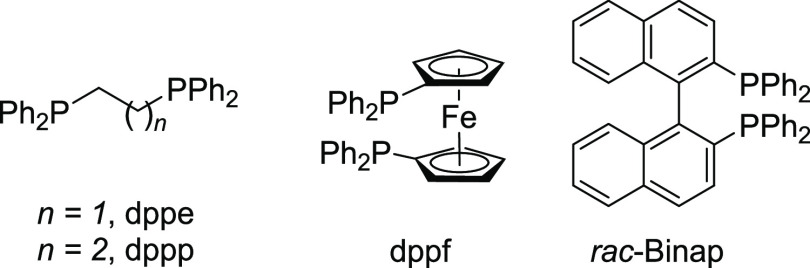

Lowering the reaction temperature led to partial conversions of **1a** toward the same product (entry 2), whereas increasing the temperature to 110 °C produced a slightly lower yield of **2a** (entry 3). The reaction could also be carried out using alternative reductants to In, like Zn (entry 4), and also using more simple halogen abstractors such as ZnBr_2_ (entry 5) or ZnI_2_, albeit in this latter case, with a significantly lower yield (entry 6). Likewise, bisphosphine ligands other than dppp, like dppe, Binap, or dppf could also be used but have a variable impact on the yield of **2a** (30–61% yield, entries 7–9). On the other hand, when the reaction was carried out without one of the four components that generate the active catalyst (either CoBr_2_, ligand, reductant, or additive), or under mere thermal conditions, the cycloaddition product was not detected.^9^ On the contrary, under optimal conditions, the reaction proved to be very robust, as it could scaled up to 1 mmol without significantly affecting the yield or selectivity (entry 10). Likewise, the loading of catalyst and additives could also be reduced by half without a major impact (entry 11). Remarkably, rhodium(I) catalysts that had previously been shown capable of promoting the cycloaddition of dimethyl-substituted cyclopropenes (Scheme 1a, top),^4f^ proved to be completely unsuccessful, leading to complex mixtures of products at 100 °C (entries 12 and 13), and to the recovery of the cycloaddition precursor at lower temperatures.^9^ Likewise, the ruthenium complex Cp*RuCl(cod) (Scheme 1a, bottom),^4g^ or alternative metal complexes previously used in reactions of cyclopropenes,^3a^ were neither successful (entry 14 and Table S1).^9^ Therefore, these control experiments confirm the unique capabilities of the cobalt catalyst for the designed reaction, even when compared with the rhodium counterpart.

**Scheme 2:**
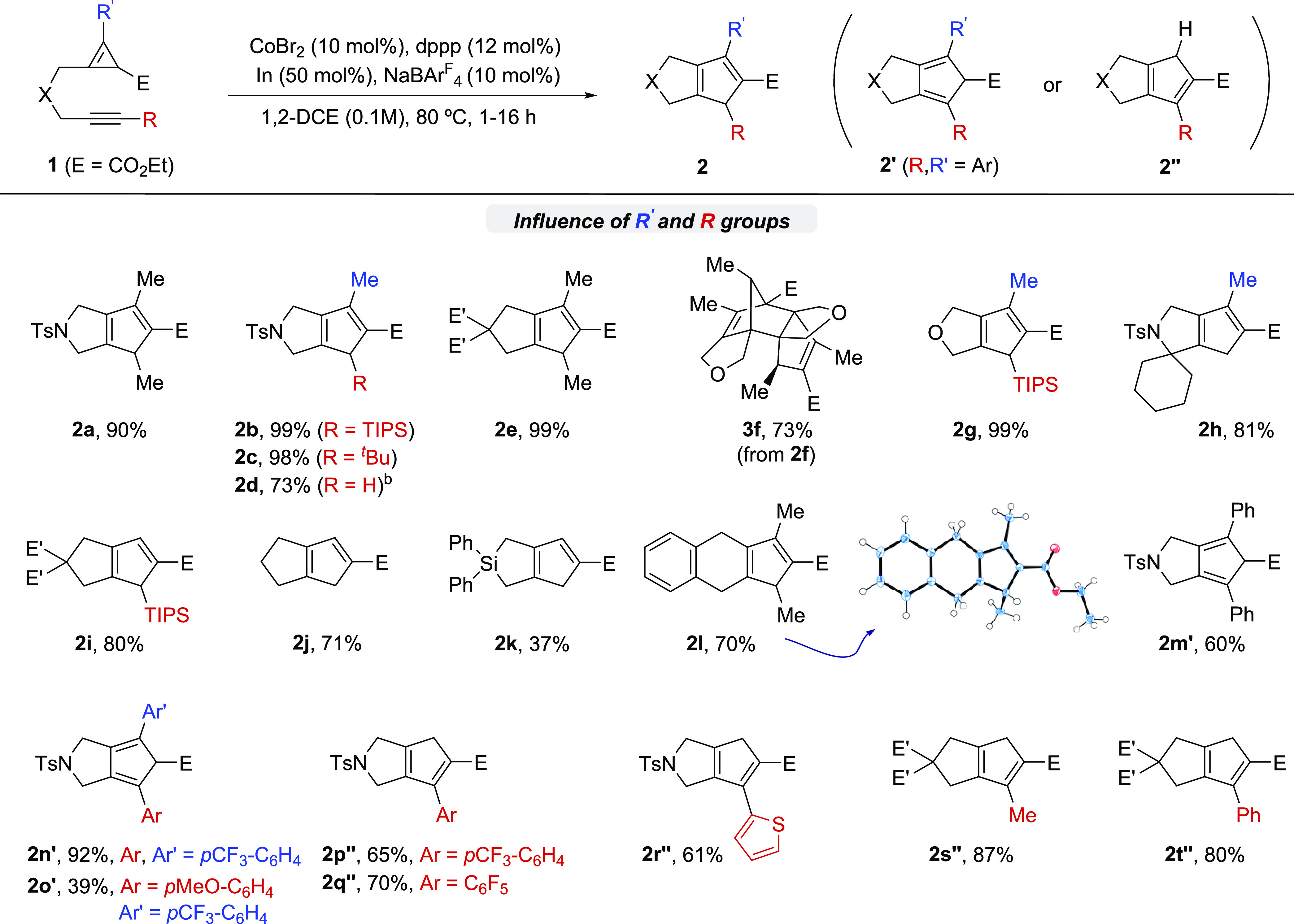
Scope of the Cobalt-Catalyzed (3 + 2) Cycloaddition Conditions: A solution of **1** in 1,2-DCE (0.1 M), CoBr_2_ (10 mol %), dppp (12 mol %), In (50 mol %) and NaBAr^F^_4_ (10 mol %) in 1,2-DCE was heated at 80 °C, unless otherwise noted. Yields of isolated products after chromatography. E′ = CO_2_Me. Product **2d** was obtained from a precursor bearing a TMS group at the alkyne (R = TMS).

With the optimized conditions in hand, we evaluated the scope of the method. *N*-tosyl derivatives related to **1a**, but bearing bulkier groups at the terminal position of the alkyne, such as a TIPS, a *tert*-butyl, or a TMS moiety, were also suitable substrates for the reaction (Scheme 2, products **2b**-**2d**). Curiously, the TMS derivative undergoes an in situ desilylation process to provide product **2d** in a good 73% yield. In all of these cases, the hydrogen atom initially located at the cyclopropene ring was formally transferred to the terminal carbon of the former alkyne.

Substrates bearing a malonate or an ether connecting tether (**1e**, **1f**) were also efficiently transformed into the expected products. Curiously, the product **2f** undergoes an in situ Diels–Alder reaction to provide the bis-adduct **3f** with complete stereoselectivity. This thermal process, which was also observed in the cycloaddition of **1a** when the heating was extended for several days,^9^ can be avoided just by using precursors with bulkier substituents. Indeed, a substrate bearing a bulky TIPS group at the alkyne delivers the desired product, **2g**, in almost quantitative yield, without traces of the self-Diels–Alder product of type **3**.

The cycloaddition method tolerates the presence of substituents at the connecting tether, as can be deduced from the reaction delivering **2h** (81% yield). The presence of a substituent at the alkene terminus of the CPE (i.e., R′) is not mandatory. Thus, bicyclic pentadienyl products of type **2i**–**2k**, bearing a hydrogen atom at that former position, were obtained in good or excellent yields. Moreover, the good yield of **2j** demonstrates that the assistance of the Thorpe-Ingold effect is not required to achieve an effective annulation. Accordingly, the connecting tether could also be elongated in one carbon atom so that the tricyclic system **2l** was obtained in a good 70% yield. Its structure was confirmed by X-ray analysis.^9^

Mechanistically relevant, when both the alkene of the CPE and the alkyne moiety bear a phenyl substituent (R, R′ = Ph), the resulting product (i.e., **2m**′) presents the diene with a different configuration to that of the above cycloadducts. In this case, a hydrogen shift did not take place. The same type of adducts are obtained from related substrates in which the electronic nature of the aromatic substituents has been modified, as demonstrated in the cycloadditions leading to **2n**′ and **2o**′. In both cases, the diene unit is in conjugation with the aromatic substituents. Remarkably, similar substrates in which the CPE is not substituted (R′ = H), the reaction yields bicyclic products of type **2″** (**2p″**–**2t″**, 61–87% yield), which are constitutional isomers of the previous ones. Notably, the reaction of the *p*CF_3_–C_6_H_4_ precursor **1p**, to afford **2p″**, could be scaled up to 2.5 mmol without any detrimental effect on the yield or rate.

All of these results confirm a good scope and that the topological configuration of the conjugated diene in the product depends on the type of cycloaddition precursor. Nonetheless, regardless of their nature, the resulting cyclopentadiene product is always obtained with complete regioselectivity with regard to C–C bond ring cleavage.

**Scheme 3:**
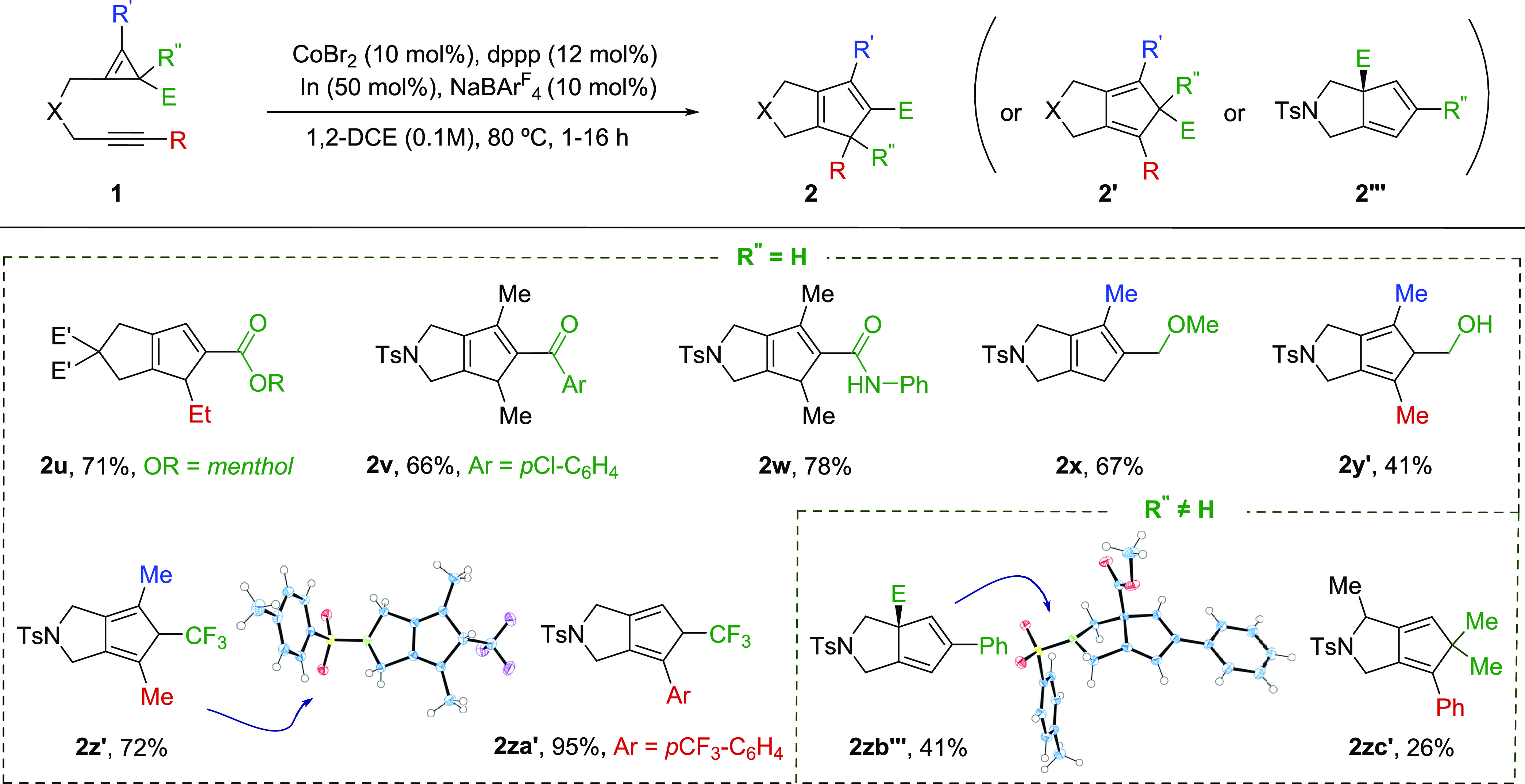
Scope of the Cobalt-Catalyzed (3 + 2) Cycloaddition Conditions: A solution of **1**, CoBr_2_ (10%), dppp (12%), In (50%) and NaBAr^F^_4_ (20%) in 1,2-DCE were heated at 80 °C unless otherwise noted. Yields of isolated products after chromatography.

We next analyzed precursors in which the CPE bears substituents other than the ethyl carboxylate at their C(sp^3^) center (Scheme 3). The presence of other esters (**2u**), a ketone (**2v**), or a carboxamide (**2w**) at this position is well tolerated. More importantly, precursors with alkyl substituents such as a methyl ether (e.g., CH_2_OMe) or a hydroxylmethyl group are also suitable for the reaction, providing the products **2x** and **2y**′, respectively. Curiously, in this latter case (**2y**′), as well as in the cycloaddition of CPEs bearing a trifluoromethyl group at the sp^3^-carbon (E = CF_3_), isomeric products of type **2**′, were obtained (e.g., **2y**′, **2z**′ and **2za**′). Finally, we also tested the cycloaddition of precursors equipped with two substituents at the cyclopropene sp^3^ carbon (R^″^ ≠ H, Scheme 3). A substrate bearing a phenyl and a carboxylic ester at this center led to a cyclopentadienyl adduct, **2zb‴**, in which the carboxy ester experienced a formal 1,3-shift.^10,11^ On the other hand, a precursor bearing two methyl groups at the CPE (R″ and E = Me) led to the expected bicyclic adduct **2zc″** (26% yield), together with a formal dehydro Diels–Alder product (**4zc**, 52% yield).^12^

Overall, all the above results confirm the unique capacity of the cobalt catalytic system to perform highly efficient (3 + 2) cycloadditions with CPEs bearing a broad variety of substituents.

**Figure 1:**
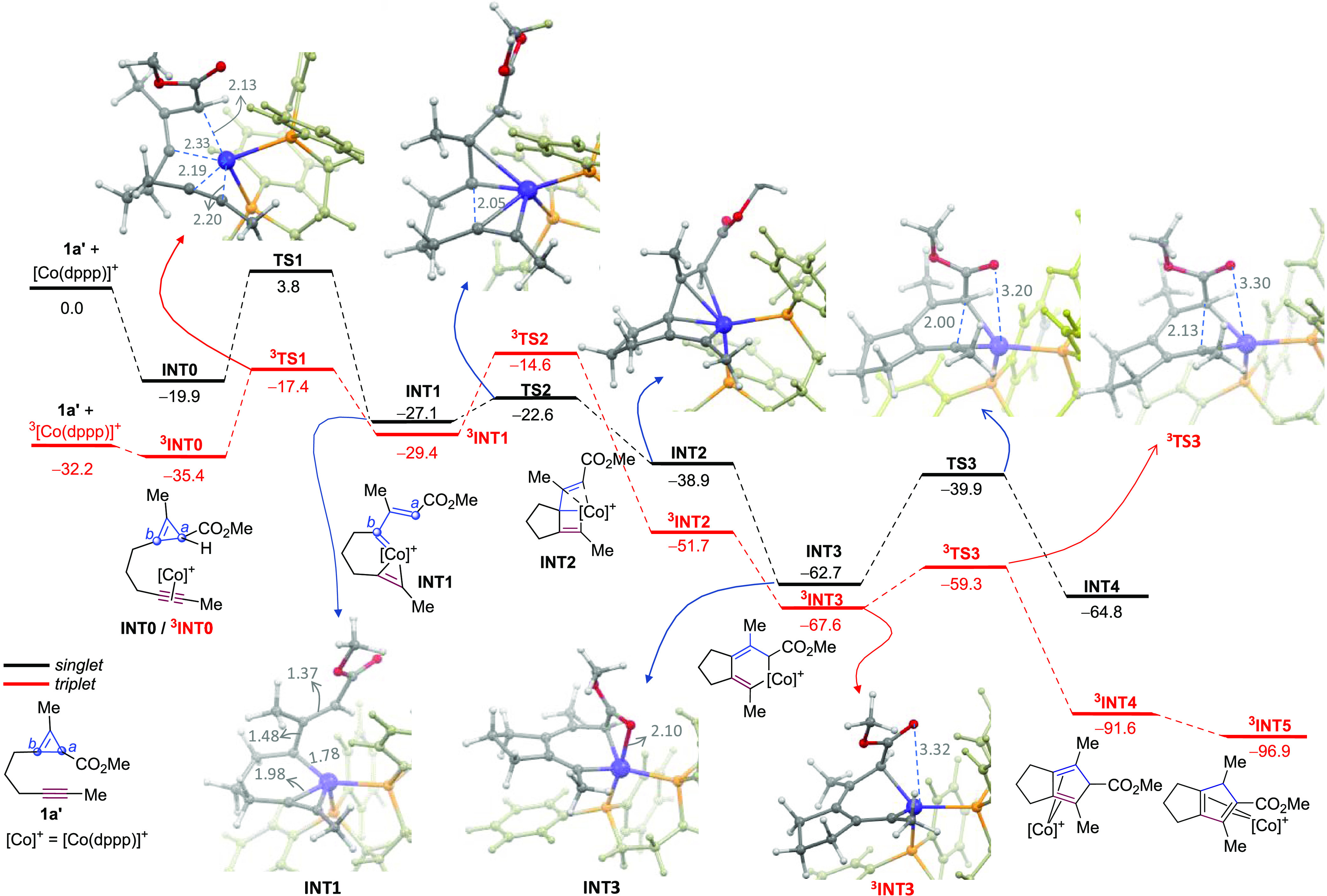
Computed reaction profile for the reaction model of **1a**′ and [Co(dppp)]^+^. Relative free energies (Δ*G*, at 353 K) are given in kcal/mol. All data were computed at the PCM(DCE)-B3LYP-D3/def2-TZVPP//PCM(DCE)-B3LYP-D3/def2-SVP level. 3D representations of the stationary points are truncated for clarity. Note that the distance C = O–Co in **INT3** (2.10 Å) is much shorter than in ^**3**^**INT3** (3.32 Å), **TS3** (3.20 Å) and ^**3**^**TS3** (3.30 Å).

To gain mechanistic insights, we performed Density Functional Theory (DFT) calculations at the dispersion-corrected PCM(DCE)-B3LYP-D3/def2-TZVPP//PCM(DCE)-B3LYP-D3/def2-SVP level.^9^ We explored the transformation of model CPE-alkyne **1a**′ mediated by the cationic Co(I) complex [(dppp)Co]^+^ (Figure 1). Given that cobalt complexes are well known to easily generate open-shell triplet species,^13^ we also analyzed the triplet energy hypersurface (red pathway). The calculations suggest that the process begins with the exergonic coordination of the alkyne to the Co(I) complex, leading to the initial intermediates **INT0** and ^**3**^**INT0**, the latter being 15.5 kcal/mol more stable than the singlet counterpart. The alkyne coordination facilitates the cobalt-promoted C–C bond cleavage of the CPE unit to deliver cobaltacyclobutene species ^**3**^**INT1** via ^**3**^**TS1**. The next step turned out to be significantly more facile via the singlet state (ΔΔ*G*^‡^ = 10.3 kcal/mol). Thus, we propose a spin crossover to the vinyl Co(I) carbene intermediate **INT1**,^14^ that undergoes an intramolecular (2 + 2) cycloaddition with the alkyne through a very accessible energy barrier of only 4.5 kcal/mol (**TS2**). The resulting cobaltacyclobutene species **INT2** would easily evolve into the much more stable cobaltacyclohexadiene intermediate **INT3**. Worth to note, although the analogous reaction step involving ^**3**^**INT1** also entails a feasible energy barrier (Δ*G*^‡^ = 14.8 kcal/mol), its transition state, ^3^**TS2**, lies significantly above **TS2** (ΔΔ*G* = 10.3 kcal/mol) and even above the initial ^**3**^**TS1** (ΔΔ*G* = 2.8 kcal/mol).

Remarkably, cobaltacyclohexadiene (**INT3**) can be converted into the cobalt-bound cyclopentadiene adduct **INT4**, via **TS3**, with an energy barrier of 22.8 kcal/mol in a process that is almost energetically thermoneutral (Δ*G* = −2.1 kcal/mol). However, we found that the triplet state intermediate ^**3**^**INT3** is 5.1 kcal/mol more stable than its singlet counterpart (**INT3**), and can readily evolve to ^**3**^**INT4**, via ^**3**^**TS3**, with an energy barrier of only 8.3 kcal/mol, almost 15 kcal/mol lower than that required for the singlet species (via **TS3**). Analysis of the relevant structures reveals that going from **INT3** to **TS3** in the singlet pathway requires decoordination of the ester carbonyl from cobalt (see **TS3**, Figure 1); however, in the triplet pathway,^**3**^**TS3** is an early transition state very similar to ^**3**^**INT3**, both of them with the carbonyl oxygen not bound to the cobalt center. This lack of ester coordination in the triplet path seems to be also consonant with the success of substrates that lack such functional groups (e.g., CF_3_, CH_2_OMe).

Finally, after the reductive elimination, the release of the cobalt complex would yield the product of type **2**′, which is experimentally observed when R and R′ are aromatic groups or when the methyl ester is replaced by a “CH_2_OH” or “CF_3_” groups (Scheme 2, **2m**–**2o′** and Scheme 3, **2z′**, **2za′**). For the ester-equipped substrate **1a′**, which bears two methyl groups, a 1,5-hydrogen shift is favored to deliver the more stable cyclopentadienyl bicyclic system of type **2**.^15^

Therefore, although the singlet and triplet profiles can both be considered viable, the lower energetic barrier in the triplet state of cyclopropene insertion and, particularly, of the reductive elimination might be behind the high efficiency of the cobalt-promoted cycloaddition. This becomes even more evident when comparing the reactivity of our cobalt catalyst with that of related Rh(I) counterparts,^4f^ for which the reductive elimination necessarily occurs via a singlet state. Indeed, our calculations for this step involving the analogous complex [Rh(dppp)]^+^ indicate that it requires a substantial energy barrier of 23.0 kcal/mol.^9^ While this is a theoretically accessible value, this catalyst failed to promote conversions at low temperatures and led to a complex mixture of products at 100 °C (Table 1, entries 8 and 9 and Table S1).^9^

Overall, these data suggest that the superior performance of the cobalt catalyst over its rhodium counterpart in our annulations stems from the activation of triplet energy surfaces, which tunnel the reaction to the desired cycloadduct over alternative side pathways.

**Scheme 4:**
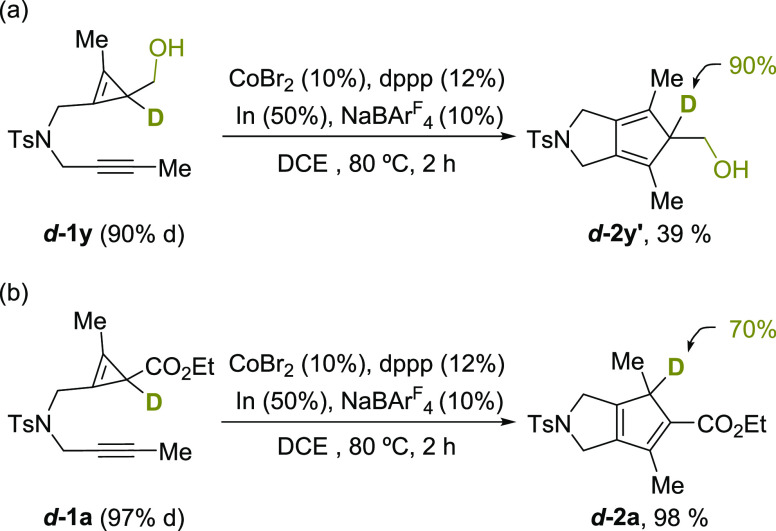
Isotope Labeling Experiments

To further shed light on the nature of the final diene isomerization, we analyzed the cycloadditions of the deuterium-labeled precursors ***d*****-1y** and ***d*****-2a** (Scheme 4). When ***d*****-1y** (with 90% deuterium content, Scheme 4) was submitted to standard reaction conditions, the expected nonisomerized cyclopentadienyl product ***d-*****2y**′, which retained a 90% of the deuterium content at its Csp^3^-center, was exclusively obtained (Scheme 4a).^9^ On the other hand, the cycloaddition of ***d*****-1a** afforded the isomerized product ***d-*****2a**, incorporating a 70% of deuterium at the new C(sp^3^) center. Despite the slight loss of deuterium content, the result is consistent with a [1,5] migration of the deuterium/hydrogen atom (Scheme 4b), although an acid–base prototropy cannot be fully discarded.^16^

Overall, our methodology provides a direct, robust, and versatile entry into a broad variety of highly substituted bicyclic cyclopentadienes, which are difficult to obtain using alternative procedures.^17^ Among different applications, cyclopentadienes can be used for the synthesis of cyclopentadienyl metal complexes (“CpML_*n*_”), a type of organometallic species that has found countless applications in catalysis.^8,18^ It is well known that the electronic characteristics of these Cp ligands can have a significant influence on the reactivity of the derived Cp-metal catalyst,^19^ especially in the case of electron-deficient Cps.^20^ However, routes to prepare highly electronically deficient Cps are scarce and have low versatility. Given that our methodology delivers a wide range of bicyclic cyclopentadienes equipped with electron-withdrawing groups, we reasoned that it could offer a direct entry to metal complexes exhibiting ring-fused, highly electronically deficient Cps.^21^

**Scheme 5:**
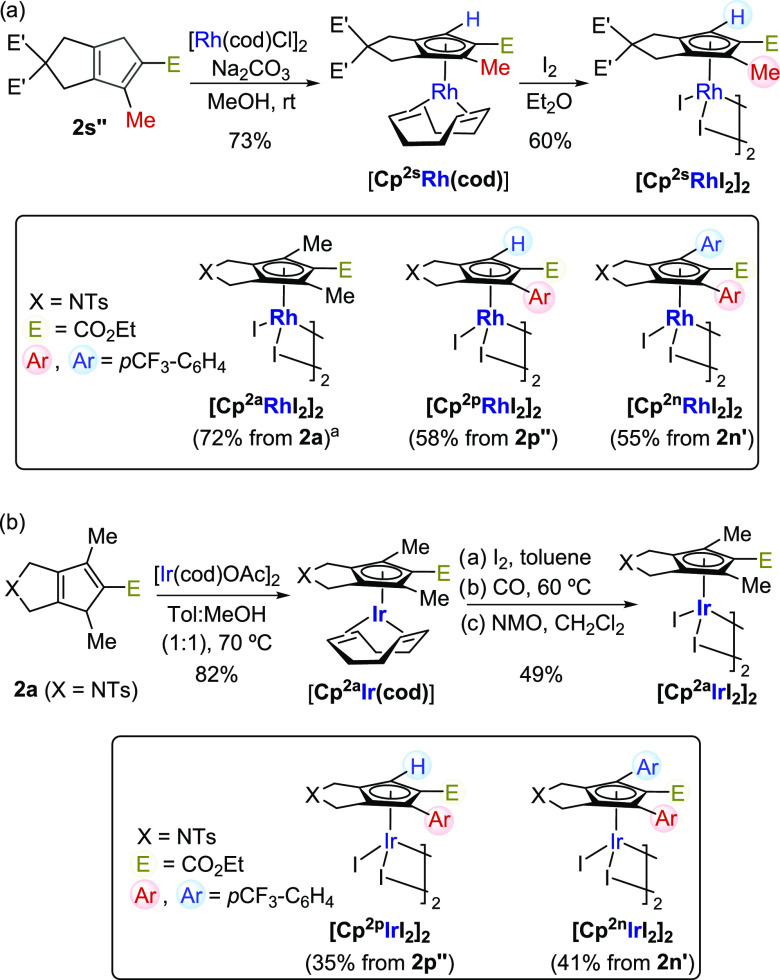
Transformation of Cyclopentadienyl Systems 2 into CpM^III^ Complexes (M = Rh or Ir) Prepared using [Rh(cod)OAc]_2_ instead of [Rh(cod)Cl]_2_/Na_2_CO_3_; See the Supporting Information for further details.

Gratifyingly, treatment of adduct **2s** with stoichiometric amounts of [Rh(cod)Cl]_2_ and a base led to the CpRh(I) derivative **[Cp**^**2s**^**Rh(cod)]** in 73% yield (Scheme 5a).^22^ Its structure was fully confirmed by X-ray crystallographic analysis.^9^ Oxidation of this Rh(I) complex with I_2_ led to Rh(III) dimer **[Cp**^**2s**^**RhI**_**2**_**]**_**2**_ in good yield. Importantly, the route allows the easy formation of other complexes, such as **[Cp**^**2p**^**RhI**_**2**_**]**_**2**_ and **[Cp**^**2n**^**RhI**_**2**_**]**_**2**_, which respectively bear, besides the CO_2_Et moiety, one or two *p*CF_3_-phenyl groups at the Cp ring.

Moreover, the bicyclic cyclopentadienyl products of type **2** can also be used to prepare related Cp-iridium counterparts. In this case, the Ir(I) complex was prepared using [Ir(cod)OAc]_2_ as the iridium(I) source, and the resulting Ir(III) complex was obtained following a two-step protocol through the carbon monoxide derivatives of type [CpIrI_2_(CO)] (Scheme 5b).^19,22^ Remarkably, a comparison of the CO stretching frequencies of the carbonyl iridium precursors [**Cp**^**2p**^**Ir(CO)I**_**2**_] (2064.9 cm^–1^) and [**Cp**^**2n**^**Ir(CO)I**_**2**_] (2065.4 cm^–1^) with those of previously reported counterparts,^19j^ confirmed that these are probably the ones with the highest electron-deficient character.^9^

**Scheme 6:**
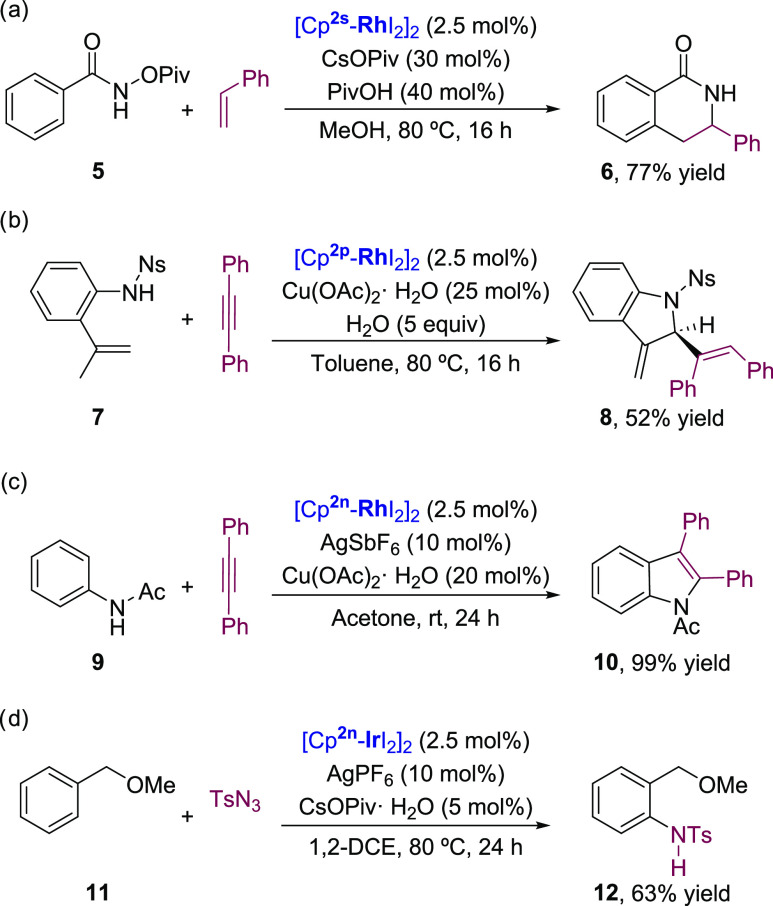
Catalytic Transformations Promoted by CpM^III^ Complexes (M = Rh or Ir)

With these new “CpM(III)” complexes in hand, we ran preliminary assays to confirm that they can promote catalytic transformations. As can be seen in Scheme 6, the catalytic system generated from **[Cp**^**2s**^**RhI**_**2**_**]**_**2**_, CsOPiv and PivOH promoted the formal [4 + 2] annulation between *O*-pivaloyl benzamides like **5** and styrene,^23^ to deliver the corresponding isoquinolone **6** in a very good yield (Scheme 6a), comparable to that obtained with the canonical Cp*Rh(III) catalysts. Moreover, the more electron-deficient complexes **[Cp**^**2p**^**RhI**_**2**_**]**_**2**_ and **[Cp**^**2n**^**RhI**_**2**_**]**_**2**_, bearing a CO_2_Et and one or two *p*CF_3_Ph groups at the cyclopentadienyl ring, were able to promote reactions that had been so far exclusive of the highly electron-deficient derivative [Cp^E^RhCl_2_]_2_ [Cp^E^ = 1,3,4-(Me)_3_,2,5-(CO_2_Et)_2_Cp]. In particular, the annulation of 2-alkenyl anilide **7** and diphenylacetylene, in the presence of the catalyst generated from **[Cp**^**2p**^**RhI**_**2**_**]**_**2**_, afforded the 2-alkenyl indoline **8** in 52% yield (Scheme 6b).^24^ Likewise, the reaction promoted by **[Cp**^**2n**^**RhI**_**2**_**]**_**2**_ between acetanilide **9** and diphenylacetylene provided the *N*-acetyl indole **10** in almost quantitative yield (Scheme 6c).^25^

Finally, the catalytic potential of the respective iridium complexes was also tested. Thus, the *ortho*-amination reaction methyl benzyl ethers with tosyl azides, a reaction which so far had been proven exclusive of the highly electron-deficient catalyst [Cp^E^IrI_2_]_2_,^19j^ could also be promoted by **[Cp**^**2n**^**IrI**_**2**_**]**_**2**_ (Scheme 6d). Thus, under nonoptimized reaction conditions, the *ortho*-amination product **12** was obtained in 63% yield.

Overall, these preliminary results confirm that the above-prepared Rh(III) and Ir(III) metal complexes, featuring ring-fused, highly substituted Cp ligands, are efficient catalysts and very promiscuous in terms of reactivity. Moreover, due to their selective performance, we anticipate that complexes of type **[Cp**^**2p**^**MI**_**2**_**]**_**2**_ and **[Cp**^**2n**^**MI**_**2**_**]**_**2**_, bearing up to three electron-withdrawing substituents at the Cp ring, hold great promise for the discovery of novel reactivities.

## Conclusions

In summary, we have unveiled a cobalt-catalyzed intramolecular cycloaddition between CPEs and alkynes that provides a straightforward and versatile entry to bicyclic, highly substituted cyclopentadienyl systems. The use of cobalt-based catalysts instead of the rhodium counterparts is mandatory for the success of the reaction, which is particularly efficient when electron-withdrawing substituents are present in the CPE and/or alkyne. Our current data firmly suggest that the success of the cobalt catalytic systems stems from their ability to open triplet potential energy surfaces, which in our cycloaddition is especially relevant for the reductive elimination step. Different from previous methods, the current system is particularly efficient with CPEs bearing a tertiary C(sp^3^) center so that the cyclic products could be easily converted into a variety of previously unknown cyclopentadienyl metal (CpM) catalysts with tunable electronic or steric properties. Those with several electron-withdrawing substituents at the Cp ligand have proven to be particularly promising. In this regard, the accessory cycle of the Cp ligand might represent an interesting handle for the introduction of designed modifications and/or chirality. Work in this direction is actively in progress.

## Data Availability

The data underlying this study are available in the published article and its Supporting Information.

## Supporting Information Available

Full experimental procedures, optimization of the catalyst, and characterization of all new compounds, including ^1^H-, ^13^C NMR spectra (PDF)
