# Supplementary material for: Cobalt-Catalyzed (3 + 2) Cycloaddition of Cyclopropene-Tethered Alkynes: Versatile Access to Bicyclic Cyclopentadienyl Systems and Their CpM Complexes
Source: ACS Catal. 2024 Jul 20;14(15):11574–83. doi: 10.1021/acscatal.4c03080 (PMC11307490; doi:10.1021/acscatal.4c03080)
Supplement: Supplementary file 1 — cs4c03080_si_001.pdf [file cs4c03080_si_001.pdf]

Supporting Information

Cobalt-Catalyzed (3+2) Cycloaddition of Cyclopropene-tethered Alkynes: Versatile Access to Bicyclic Cyclopentadienyl Systems and their CpM Complexes

Carlos Lázaro-Milla,[a] Eduardo da Concepción,[a] Israel Fernández,*[b] José L. Mascareñas,*[a] Fernando López*[a,c]

[a] Centro Singular de Investigación en Química Biológica y Materiales Moleculares (CiQUS) and Departamento de Química Orgánica. Universidad de Santiago de Compostela. C/ Jenaro de la Fuente, s/n, 15782, Santiago de Compostela, Spain

[b] Departamento de Química Orgánica I and Centro de Innovación en Química Avanzada (ORFEO-CINQA), Facultad de Ciencias Químicas, Universidad Complutense de Madrid, 28040 Madrid, Spain

[c] Misión Biológica de Galicia, Consejo Superior de Investigaciones Científicas (CSIC), 36080, Pontevedra, Spain

Corresponding Authors:

Dr. Fernando Lopéz: fernando.lopez@csic.es

Prof. José L. Mascareñas: joseluis.mascarenas@usc.es

Prof. Dr. Israel Fernández: israel@quim.ucm.es

Table of contents

**1. General Procedures**	**S3**
**2. Optimization and additional experiments**	**S4**
**3. Procedures for the synthesis of reaction precursors 1**	**S6**
- General procedure A: Cyclopropenation of diynes (illustrated for **1a**)	S6
- General procedure B: Cyclopropenation of propargyl malonates followed by propargylation (illustrated for **1s**)	S13
- General procedure C: Cyclopropenation of diynes with trifluoromethyl diazomethane (illustrated for **1z**)	S15
- Procedure for the preparation of **1h**	S16
- Procedure for the preparation of **1w**	S17
- Procedure for the preparation of **1y**	S18
- Procedure for the preparation of **1x**	S18
- Procedure for the preparation of **1zc**	S19
- Preparation of deuterated precursors (***d*-1a** and ***d*-1y**)	S20
**4. Co-catalyzed (3+2) cycloaddition of cyclopropene-tethered alkynes 1** (exemplified for **1a**)	**S21**
**5. Synthesis of cyclopentadienyl metal complexes**	**S33**
**6. Catalytic transformations promoted by CpM(III) complexes**	**S41**
**7. Computational details**	**S43**
**8. NMR spectra**	**S75**
**9. IR study**	**S182**
**10. References**	**S183**

1. General procedures

All reactions were conducted in dry solvents under Ar atmosphere unless otherwise stated. The abbreviation "rt" refers to reactions carried out at 20-25 °C. Reaction mixtures were stirred using Teflon-coated magnetic stir bars. Reaction temperatures were maintained using Thermowatch-controlled silicone oil baths. Dry solvents were obtained from a solvent purification system (Mbraun, SPS-5) or freshly distilled under Ar from an appropriate drying agent before use (toluene from Na / benzophenone, CH_2Cl_2 distilled form CaH_2). The water utilized in the reactions is sourced from a Milli-Q® water purification system. All other reagents for the synthesis of precursors were purchased from Aldrich. Thin-layer chromatography (TLC) was performed on pre-coated silica gel F_{254} plates and were visualized by observation under UV light, or by treating the plates with either vanillin, *p*-anisaldehyde, ninhydrin, potassium permanganate or cerium nitrate solutions, followed by heating. Flash chromatography was carried out on silica gel (40-60 µm) unless otherwise stated. Dryings were performed with anhydrous Na_2SO_4. Concentration refers to the removal of volatile solvents via distillation using a Buchi rotary evaporator, followed by residual solvent removal under high vacuum. In reactions carried out in sealed tubes, the mixture must be at rt before sealing, to avoid overpressure at high temperatures.

^1H, ^{13}C and ^{19}F spectra were recorded in $CDCl_3$, CD_2Cl_2, DMSO-d_6 or C_6D_6 at Varian Mercury 300 MHz or Bruker AVIII 500 MHz spectrometers. Carbon types were determined from DEPT-NMR experiments. NMR spectra were analyzed using MestreNova© processing software (www.mestrelab.com). 1,3,5-Trimethoxybenzene was used as internal standard for analyzing the reaction crudes by NMR.

^1H-NMR spectral data are reported as follows: chemical shift (δ ppm), integration, multiplicity (s = singlet, d = doublet, t = triplet, q = quartet, dd = double doublet, td = triple doublet, m = multiplet, br = broad). Data for ^{13}C are reported in terms of chemical shift relative to the residual solvent peak.

Mass spectra were acquired using chemical ionization (CI) or electron impact techniques (EI) and were recorded at the CACTUS facility of the USC. IR spectral data were recorded at Jasco FT/IR-6X1 ATR spectrometer.

X-ray crystallographic analysis[1] was performed at the CACTUS facility of the University of Santiago de Compostela.

2. Optimization and additional experiments

Optimization of the Co-catalyzed (3+2) intramolecular cycloaddition of cyclopropene-tethered alkynes 1

Table S1. Evaluation of different catalysts, reductants, additives and temperatures, **1a** as model substrate.[a]

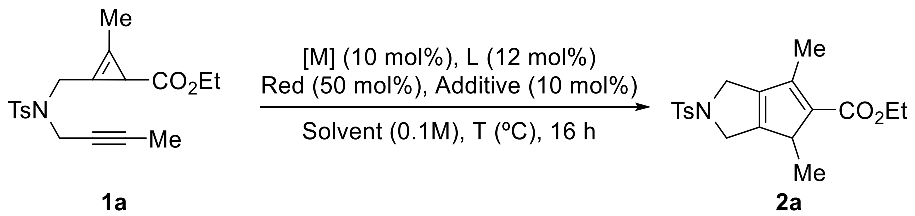

Entry	[M]	Red	Ligand	Additive	Solvent	T (°C)	Yield (%)
1	$CoBr_2$	In	dppp	$NaBAr^F_4$	DCE	80	90
2	$CoBr_2$	In	dppp	$NaBAr^F_4$	DCE	70	66[b]
3	$CoBr_2$	In	dppp	$NaBAr^F_4$	DCE	60	0[d]
4	$CoBr_2$	In	dppp	$NaBAr^F_4$	DCE	40	0[d]
5	$CoBr_2$	In	dppp	$NaBAr^F_4$	DCE	110	80
6	$CoBr_2$	Zn	dppp	$NaBAr^F_4$	DCE	80	72
7	$CoBr_2$	Zn	dppp	$ZnBr_2$	DCE	80	57
8	$CoBr_2$	Zn	dppp	ZnI_2	DCE	80	31
9	CoI_2	Zn	dppp	ZnI_2	DCE	80	23
10	$CoBr_2$	In	dppp	$InBr_3$	DCE	80	15
11	$CoBr_2$	In	dppe	$NaBAr^F_4$	DCE	80	30
12	$CoBr_2$	In	dppf	$NaBAr^F_4$	DCE	80	61
13	$CoBr_2$	In	*rac*-Binap	$NaBAr^F_4$	DCE	80	43
14[e]	$CoBr_2$	In	dppp	$NaBAr^F_4$	DCE	80	80[f]
15[h]	$CoBr_2$	In	dppp	$NaBAr^F_4$	DCE	80	66[i]
16	$RhCl(PPh_3)_3$	-	-	-	toluene	100	-[c]
17	$[Rh(cod)_2]BF_4$	-	*rac*-Binap	-	$PhCF_3$	100	-[c]
18	$[Rh(cod)_2]BF_4$	-	dppp	-	DCE	80	-[g]
19	$[Rh(cod)_2]BF_4$	-	dppp	-	DCE	110	-[g]
20	$[Rh(cod)_2]BF_4$	-	dppp	-	toluene	80	-[g]
21	$Cp^*RuCl(cod)$	-	-	-	$PhCF_3$	-	-[c]
22	Au-Dalphos	-	-	$AgSbF_6$	CH_2Cl_2	rt	0[d]
23	$[Ir(cod)Cl]_2$	-	*rac*-Binap	-	toluene	100	-[c]
24	$[Cp^*IrCl_2]_2$	-	-	-	toluene	100	-[c]

[a] Conditions: A solution of **1a**, [M] (10 mol%), L (12 mol%), reductant (Red, 50 mol%) and additive (10 mol%), in 1,2-DCE, was heated for 16 h at the indicated temperature. [b] 83% conversion. [c] A complex mixture of products was observed. [d] The starting material **1a** was fully recovered. [e] Reaction carried out with half amount of catalyst and additives: $CoBr_2$ (5 mol%), dppp (6 mol%), In (25 mol%) and $NaBAr^F_4$ (5 mol%), in 1,2-DCE, was heated for 16 h at 80 °C. [f] 89% conversion. [g] Partial decomposition of the starting material. The formation of significative amounts of

products was not observed. [h] Reaction carried out under standard conditions but with reduced amount of In (30 mol%). [i] Full conversion of **1a** was observed together with the formation of significative amounts of byproducts.

NOTE: When the reaction under conditions of entry 1 was carried out without one of the four components that generate the active catalyst (either $CoBr_2$, dppp, In or $NaBAR^F{}_4$), the cycloaddition product was not detected. Likewise, treatment of **1a** under thermal conditions (heating up to 130 ºC) did not afford the product.

Additional Rh-catalyzed experiments

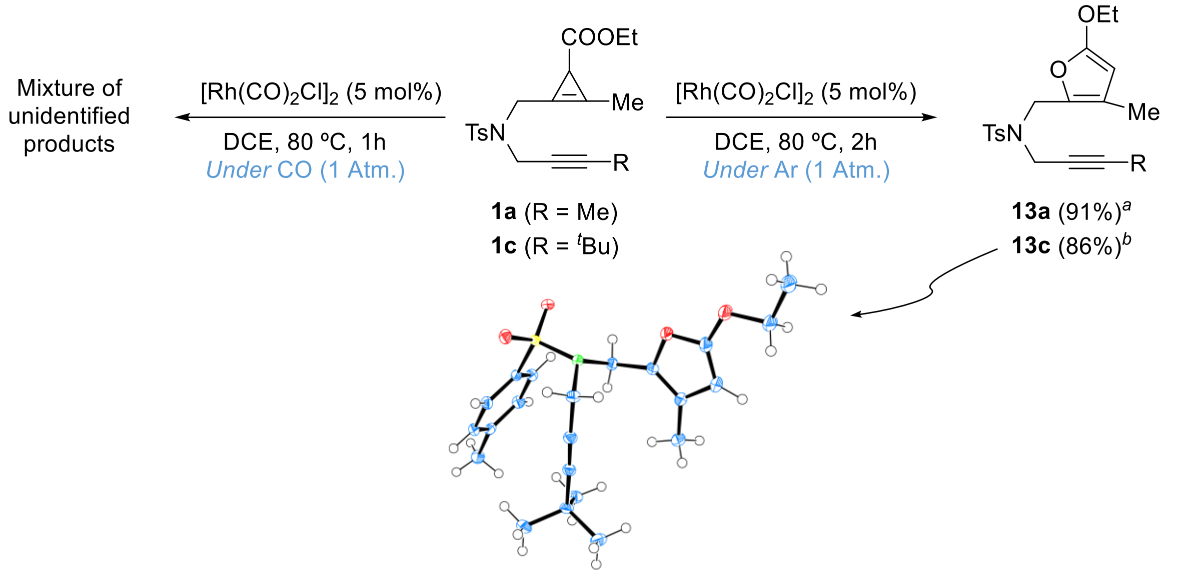

X-ray crystallographic analysis of **13c** (CCDC 2364701)

[a] Unstable product, fast decomposition, not isolable. Yield determined by NMR of the crude. [b] Yield of isolated product.

This type of Rh-catalyzed cyclopropene ring expansion was previously described by Padwa and coworkers.[2]

_N_-(4,4-dimethylpent-2-yn-1-yl)-_N_-((5-ethoxy-3-methylfuran-2-yl)methyl)-4-methylbenzenesulfonamide (13c)

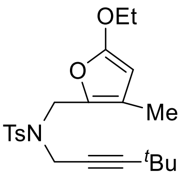

$[Rh(CO)_2Cl]_2$ (1.4 mg, 0.05 mmol), was added to a dried Schlenk tube under Argon. Then, **1c** (30.0 mg, 0.074 mmol) in 1,2-DCE (0.75 mL) was added under Ar atmosphere and the resulting mixture was stirred at 80 °C until complete consumption of starting material was indicated by TLC (2h). The mixture was filtered through a short pad of Florisil® and concentrated under vacuum. The resulting crude was purified by flash column chromatography using silica gel (95:5 → 90:10 hexane/EtOAc), to afford 26 mg of *N*-(4,4-dimethylpent-2-yn-1-yl)-*N*-((5-ethoxy-3-methylfuran-2-yl)methyl)-4-methylbenzenesulfonamide **13c**, as a white solid (86% yield). R_f = 0.66 (7:3 hexane/ EtOAc). **^{1}H NMR (300 MHz, $CDCl_3$):** δ 7.76 (m, 2H), 7.29 (m, 2H), 4.96 (s, 1H), 4.29 (s, 1H), 4.02 (m, 4H), 2.43 (s, 3H), 2.00 (s, 3H), 1.38 (t, *J* = 7.1 Hz, 3H), 1.01 (s, 9H). **^{13}C NMR (75 MHz, $CDCl_3$):** δ 159.9 (C), 143.0 (C), 136.7 (C), 133.6 (C), 129.4 (2CH), 127.7 (2CH), 121.4 (C), 94.3 (C), 83.8 (CH), 71.4 (C), 66.5 (CH_2), 40.5 (CH_2), 36.2 (CH_2), 30.6

(3CH$_3$), 27.1 (C), 21.4 (CH$_3$), 14.6 (CH$_3$), 10.3 (CH$_3$). **HRMS** (APCI-FIA-TOF): *m/z* calculated for $C_{22}H_{30}NO_4S$ [M + H]$^+$: 404.1890, found 404.1894.

Intermolecular experiments[a]

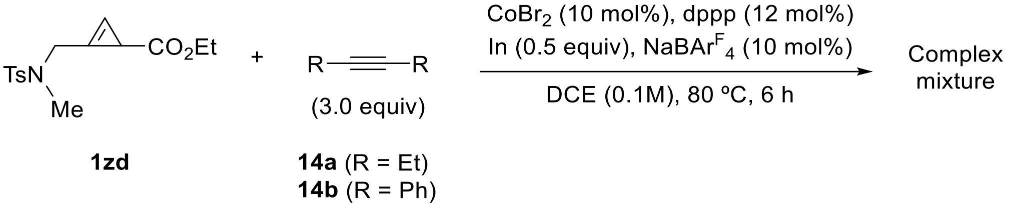

[a] Intermolecular experiments were performed following the general procedure for the Co-catalyzed (3+2) cycloaddition of cyclopropene-tethered alkynes **1**, using **1zd** as cyclopropene partner (1.0 equiv) and symmetrical alkynes **14** (3.0 equiv).

3. Procedures for the synthesis of reaction precursors 1

General procedure A: Cyclopropenation of diynes (illustrated for 1a)

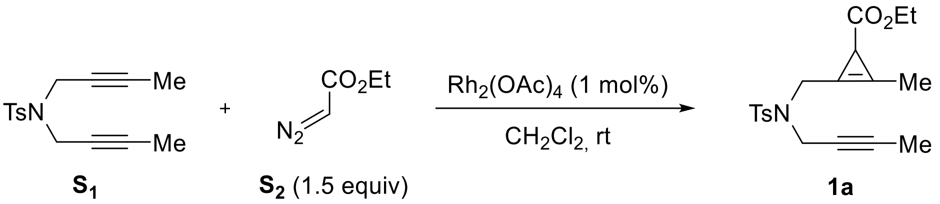

[$Rh_2(OAc)_4$] (16.0 mg, 0.036 mmol, 1.0 mol %) and freshly distilled CH_2Cl_2 (3.6 mL) were added to a dried Schlenk tube. The homogeneous green solution was stirred for 5 min before a *N,N*-di(but-2-yn-1-yl)-4-methylbenzenesulfonamide **S$_1$** (1.00 g, 3.63 mmol) was added. Ethyl 2-diazoacetate **S$_2$** (714 mg, 5.44 mmol, 87% purity) in anhydrous CH_2Cl_2 (7.3 mL) was added to this solution through a syringe pump at a rate of 0.5 mL/h, at rt. Upon completion of addition, the solvent was removed under reduced pressure. The crude was purified by flash chromatography on silica gel (9:1 $\rightarrow$ 8:2 hexane:EtOAc) to afford 683 mg of ethyl 2-(((*N*-(but-2-yn-1-yl)-4-methylphenyl)sulfonamido)methyl)-3-methylcycloprop-2-ene-1-carboxylate, **1a**, as a colorless oil (52% yield). R_f = 0.53 (7:3 hexane/EtOAc). **^{1}H NMR (300 MHz, CDCl$_3$):** δ 7.73 (m, 2H), 7.29 (m, 2H), 4.33 (s, 2H), 4.17 – 4.02 (m, 4H), 2.42 (s, 3H), 2.03 (s, 1H), 1.93 (s, 3H), 1.60 (t, *J* = 2.4 Hz, 3H), 1.24 (t, *J* = 7.1 Hz, 3H). **^{13}C NMR (75 MHz, CDCl$_3$):** δ 175.3 (CO), 143.4 (C), 136.0 (C), 129.3 (2CH), 127.8 (2CH), 107.5 (C), 101.3 (C), 81.8 (C), 71.5 (C), 60.1 (CH$_2$), 41.6 (CH$_2$), 37.3 (CH$_2$), 23.1 (CH), 21.4 (CH$_3$), 14.3 (CH$_3$), 9.6 (CH$_3$), 3.3 (CH$_3$). **HRMS** (APCI-FIA-TOF): *m/z* calculated for $C_{19}H_{25}NO_4S$ [M + H]$^+$: 362.1648, found 362.1450.

Ethyl 2-methyl-3-(((4-methyl-*N*-(3-(triisopropylsilyl)prop-2-yn-1-yl)phenyl)sulfonamido)methyl)cycloprop-2-ene-1-carboxylate (1b)

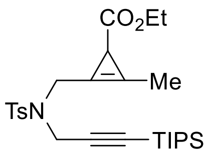

Prepared following the general procedure A, from *N*-(but-2-yn-1-yl)-4-methyl-*N*-(3-(triisopropylsilyl)prop-2-yn-1-yl)benzenesulfonamide (1.70 g, 4.07 mmol), using ethyl diazoacetate (800 mg, 6.10 mmol, 87% purity) to afford 960 mg of **1b** (52% yield, pale yellow oil) after purification by flash chromatography on silica gel (100:4 → 90:10 hexane/EtOAc). R_f = 0.53 (8:2 hexane/EtOAc). *Rotamers are detected by NMR, only the main signals are described*. **^{1}H NMR (300 MHz, $CDCl_3$):** *δ* 7.74 (m, 2H), 7.29 (m, 2H), 4.41 (m, 2H), 4.25 (s, 2H), 4.13 (m, 2H), 2.42 (s, 3H), 2.10 (s, 1H), 2.00 (s, 3H), 1.27 (m, 3H), 1.08 (m, 3H), 1.02 – 0.92 (m, 18H). **^{13}C NMR (75 MHz, $CDCl_3$):** *δ* 175.3 (CO), 143.5 (C), 136.1 (C), 129.7 (2CH), 127.6 (2CH), 108.4 (C), 101.3 (C), 99.4 (C), 87.5 (C), 60.1 (CH_2), 41.1 (CH_2), 37.5 (CH_2), 23.1 (CH_3), 21.4 (CH), 18.4 (6CH_3), 14.3 (CH_3), 11.0 (3CH), 9.6 (CH_3). **HRMS** (APCI-FIA-TOF): *m/z* calculated for $C_{27}H_{42}NO_4SSi$ $[M + H]^+$: 504.2598, found 504.2615.

Ethyl 2-(((*N*-(4,4-dimethylpent-2-yn-1-yl)-4-methylphenyl)sulfonamido)methyl)-3-methylcycloprop-2-ene-1-carboxylate (1c)

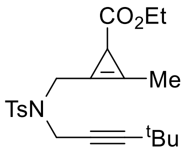

Prepared following the general procedure A, from *N*-(but-2-yn-1-yl)-*N*-(4,4-dimethylpent-2-yn-1-yl)-4-methylbenzenesulfonamide (890 mg, 2.80 mmol), using ethyl diazoacetate (551 mg, 4.20 mmol, 87% purity) to afford 612 mg of **1c** (73% yield, colorless oil) after purification by flash chromatography on silica gel (90:10 → 85:15 hexane/EtOAc). R_f = 0.37 (8:2 hexane/EtOAc). *Rotamers are detected by NMR, only the main signals are described*. **^{1}H NMR (300 MHz, $CDCl_3$):** *δ* 7.52 (m, 2H), 7.11 (m, 2H), 4.12 (m, 2H), 3.89 (m, 4H), 2.21 (s, 3H), 1.86 (s, 1H), 1.76 (s, 3H), 1.03 (t, *J* = 7.0 Hz, 3H), 0.96 (m, 3H), 0.81 (s, 6H). **^{13}C NMR (75 MHz, $CDCl_3$):** *δ* 174.4 (CO), 142.9 (C), 135.5 (C), 129.0 (2CH), 127.1 (2CH), 107.4 (C), 100.8 (C), 94.2 (C), 70.1 (C), 59.3 (CH_2), 40.5 (CH_2), 36.5 (CH_2), 30.0 (2CH_3), 27.2 (C), 26.5 (CH_3), 22.5 (CH), 20.8 (CH_3), 13.8 (CH_3), 8.9 (CH_3). **HRMS** (APCI-FIA-TOF): *m/z* calculated for $C_{22}H_{30}NO_4S$ $[M + H]^+$: 404.1890, found 404.1889.

Ethyl 2-methyl-3-(((4-methyl-*N*-(3-(trimethylsilyl)prop-2-yn-1-yl)phenyl)sulfonamido)methyl)cycloprop-2-ene-1-carboxylate (1d)

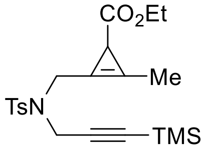

Prepared following the general procedure A, from *N*-(but-2-yn-1-yl)-4-methyl-*N*-(3-(trimethylsilyl)prop-2-yn-1-yl)benzenesulfonamide (1.36 g, 4.09 mmol), using ethyl diazoacetate (805 mg, 6.13 mmol, 87% purity) to afford 900 mg of **1d** (52% yield, colorless oil) after purification by flash chromatography on silica gel (90:10 → 85:15 hexane/EtOAc). R_f = 0.33 (8:2 hexane/EtOAc). *Rotamers are detected by NMR, only the main signals are described*. **^{1}H NMR (300 MHz, $CDCl_3$):** *δ* 7.74 (m, 2H), 7.31 (m, 2H), 4.37 (m, 2H), 4.11 (m, 4H), 2.43 (s, 3H), 2.08 (s, 1H), 1.99 (s, 3H), 1.26 (t, *J* = 7.1 Hz, 3H), 0.20 (s, 3H), 0.04 (s, 6H). **^{13}C NMR (75 MHz, $CDCl_3$):** *δ* 175.2 (CO), 143.5 (C), 135.8 (C), 129.5 (2CH), 127.7 (2CH), 108.2 (C), 101.3 (C), 97.6 (C), 91.1 (C), 60.1 (CH_2), 41.4 (CH_2), 37.7 (CH_2), 23.2

(CH), 21.5 (CH_3), 14.3 (CH_3), 9.6 (CH_3), -0.4 ($2CH_3$), -1.8 (CH_3). **HRMS** (APCI-FIA-TOF): *m/z* calculated for $C_{22}H_{30}NO_4S$ $[M + H]^+$: 420.1659, found 420.1668.

Dimethyl 2-(but-2-yn-1-yl)-2-((3-(ethoxycarbonyl)-2-methylcycloprop-1-en-1-yl)methyl)malonate (1e)

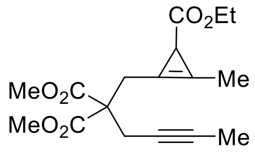

Prepared following the general procedure A, from dimethyl 2,2-di(but-2-yn-1-yl)malonate (473 mg, 2.0 mmol), using ethyl diazoacetate (393 mg, 3.0 mmol, 87% purity) to afford 341 mg of **1e** (53% yield, colourless oil) after purification by flash chromatography on silica gel (9:1 hexane/Et_2O). R_f = 0.64 (8:2 hexane/ Et_2O). **^{1}H NMR (300 MHz, $CDCl_3$):** δ 4.06 (m, 2H), 3.71 (s, 6H), 3.19 (s, 2H), 2.80 (s, 2H), 2.01 (s, 3H), 1.98 (s, 1H), 1.71 (s, 3H), 1.22 (t, *J* = 7.1 Hz, 3H). **^{13}C NMR (75 MHz, $CDCl_3$):** δ 175.8 (CO), 169.9 (CO), 169.8 (CO), 105.6 (C), 101.6 (C), 79.0 (C), 72.9 (C), 59.8 (CH_2), 56.1 (C), 52.8 ($2CH_3$), 28.3 (CH_2), 23.5 (CH_2), 22.5 (CH), 14.3 (CH_3), 9.9 (CH_3), 3.3 (CH_3). **HRMS** (APCI-FIA-TOF): *m/z* calculated for $C_{17}H_{21}O_6$ $[M - H]^+$: 321.1333, found 321.1337.

Ethyl 2-((but-2-yn-1-yloxy)methyl)-3-methylcycloprop-2-ene-1-carboxylate (1f)

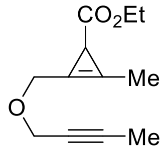

Prepared following the general procedure A, from 1-(but-2-yn-1-yloxy)but-2-yne (300 mg, 2.45 mmol), using ethyl diazoacetate (483 mg, 3.68 mmol, 87% purity) to afford 188 mg of **1f** (36% yield, colourless oil) after purification by flash chromatography on silica gel (100:10 hexane/EtOAc). R_f = 0.34 (8:2 hexane/EtOAc). **^{1}H NMR (300 MHz, $CDCl_3$):** δ 4.40 (s, 2H), 4.11 – 3.98 (m, 4H), 2.10 (s, 1H), 2.04 (s, 3H), 1.76 (s, 3H), 1.16 (t, *J* = 7.1 Hz, 3H). **^{13}C NMR (75 MHz, $CDCl_3$):** δ 175.4 (CO), 106.4 (C), 102.7 (C), 82.7 (C), 74.4 (C), 62.1 (CH_2), 59.8 (CH_2), 57.8 (CH_2), 22.8 (CH), 14.1 (CH_3), 9.6 (CH_3), 3.3 (CH_3). **HRMS** (APCI-FIA-TOF): *m/z* calculated for $C_{12}H_{17}O_3$ $[M + H]^+$: 209.1172, found 209.1171.

Ethyl 2-methyl-3-(((3-(triisopropylsilyl)prop-2-yn-1-yl)oxy)methyl)cycloprop-2-ene-1-carboxylate (1g)

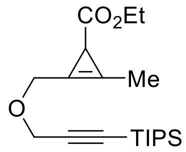

Prepared following the general procedure A, from (3-(but-2-yn-1-yloxy)prop-1-yn-1-yl)triisopropylsilane (306 mg, 1.15 mmol),using ethyl diazoacetate (228 mg, 1.73 mmol, 87% purity) to afford 114 mg of **1g** (28% yield, colorless oil) after purification by flash chromatography on silica gel (95:5 hexane/EtOAc). R_f = 0.6 (8:2 hexane/EtOAc). **^{1}H NMR (300 MHz, $CDCl_3$):** δ 4.53 (s, 2H), 4.24 (d, *J* = 4.5 Hz, 2H), 4.11 (q, *J* = 7.1 Hz, 2H), 2.18 (s, 1H), 2.11 (s, 3H), 1.23 (t, *J* = 7.3 Hz, 3H), 1.08 – 1.03 (m, 21H). **^{13}C NMR (75 MHz, $CDCl_3$):** δ 175.6 (CO), 107.1 (C), 102.8 (C), 102.5 (C), 88.2 (C), 61.8 (CH_2), 60.0 (CH_2), 58.0 (CH_2), 22.9 (CH), 18.5 ($6CH_3$), 14.3 (CH_3), 11.1 (3CH), 9.8 (CH_3). **HRMS** (APCI-FIA-TOF): *m/z* calculated for $C_{20}H_{35}O_3Si$ $[M + H]^+$: 351.2350, found 351.2348.

Dimethyl 2-((3-(ethoxycarbonyl)cycloprop-1-en-1-yl)methyl)-2-(3-(triisopropylsilyl)prop-2-yn-1-yl)malonate (1i)

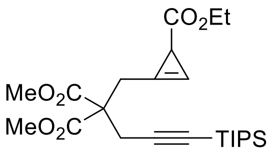

Prepared following the general procedure A, from dimethyl 2-(prop-2-yn-1-yl)-2-(3-(triisopropylsilyl)prop-2-yn-1-yl)malonate (738 mg, 2.02 mmol), using ethyl diazoacetate (398 mg, 3.04 mmol, 87% purity) to afford 355 mg of **1i** (39% yield, colorless oil) after purification by flash chromatography on silica gel (9:1 hexane/Et_2O). R_f = 0.12 (8:2 hexane/Et_2O). **^{1}H NMR (300 MHz, $CDCl_3$):** δ 6.51 (s, 1H), 4.11 (q, *J* = 7.1 Hz, 2H), 3.74 (s, 6H), 3.35 (s, 2H), 3.03 (d, *J* = 17.2 Hz, 1H), 2.93 (d, *J* = 17.3 Hz, 1H), 2.11 (d, *J* = 1.5 Hz, 1H), 1.25 (t, *J* = 7.2 Hz, 3H), 1.04 (m, 21H). **^{13}C NMR (75 MHz, $CDCl_3$):** δ 175.4 (CO), 169.4 (CO), 169.3 (CO), 111.1 (C), 102.1 (C), 98.1 (CH), 84.5 (C), 60.2 (CH_2), 56.0 (C), 52.9 ($2CH_3$), 28.2 (CH_2), 24.5 (CH_2), 19.6 (CH), 18.5 ($6CH_3$), 14.3 (CH_3), 11.1 (3CH). **HRMS** (APCI-FIA-TOF): *m/z* calculated for $C_{24}H_{39}O_6Si$ $[M + H]^+$: 451.2510, found 451.2519.

Ethyl 2-(pent-4-yn-1-yl)cycloprop-2-ene-1-carboxylate (1j)

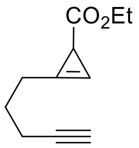

Prepared following the general procedure A, from hepta-1,6-diyne (184 mg, 2.0 mmol) using ethyl diazoacetate (393 mg, 3.0 mmol, 87% purity) to afford 174 mg of **1j** (49% yield, pale yellow oil) after purification by flash chromatography on silica gel (100:2 $\rightarrow$ 100:4 hexane/EtOAc). R_f = 0.5 (8:2 hexane/EtOAc). **^{1}H NMR (300 MHz, $CDCl_3$):** δ 6.37 (s, 1H), 4.10 (m, 2H), 2.61 (t, *J* = 7.3 Hz, 2H), 2.25 (m, 2H), 2.11 (s, 1H), 1.94 (t, *J* = 2.7 Hz, 1H), 1.79 (m, 2H), 1.22 (t, *J* = 7.1 Hz, 3H). **^{13}C NMR (75 MHz, $CDCl_3$):** δ 176.2 (CO), 114.8 (C), 94.9 (CH), 83.3 (C), 68.9 (CH), 60.1 (CH_2), 25.5 (CH_2), 23.9 (CH_2), 19.6 (CH), 17.7 (CH_2), 14.2 (CH_3). **HRMS** (APCI-FIA-TOF): *m/z* calculated for $C_{11}H_{15}O_2$ $[M + H]^+$: 179.1067, found 179.1059.

Ethyl 2-((diphenyl(prop-2-yn-1-yl)silyl)methyl)cycloprop-2-ene-1-carboxylate (1k)

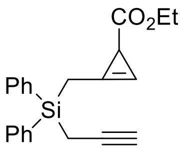

Prepared following the general procedure A, from diphenyldi(prop-2-yn-1-yl)silane (300 mg, 1.15 mmol), using ethyl diazoacetate (227 mg, 1.72 mmol, 87% purity) to afford 123 mg of **1k** (30% yield, pale yellow oil) after purification by flash chromatography on silica gel (100:4 $\rightarrow$ 100:6 hexane/EtOAc). R_f = 0.45 (8:2 hexane/EtOAc). **^{1}H NMR (300 MHz, $CDCl_3$):** δ 7.65 (m, 4H), 7.43 (m, 6H), 6.25 (s, 1H), 4.01 (q, *J* = 7.1 Hz, 2H), 2.72 (m, 2H), 2.19 (d, *J* = 3.0 Hz, 2H), 2.06 (d, *J* = 1.5 Hz, 1H), 1.94 (t, *J* = 2.9 Hz, 1H), 1.21 (t, *J* = 7.1 Hz, 3H). **^{13}C-NMR (75 MHz, $CDCl_3$):** δ 176.2 (CO), 134.7 (4CH), 130.1 (4CH), 127.9 (2CH), 111.9 (C), 94.2 (CH), 80.6 (C), 68.85 (CH), 60.0 (CH_2), 20.9 (CH_3), 14.2 (CH_3), 10.9 (CH_2), 3.6 (CH_2). **HRMS** (APCI-FIA-TOF): *m/z* calculated for $C_{22}H_{23}O_2Si$ $[M + H]^+$: 347.1462, found 347.1469.

Ethyl 2-(2-(but-2-yn-1-yl)benzyl)-3-methylcycloprop-2-ene-1-carboxylate (1l)

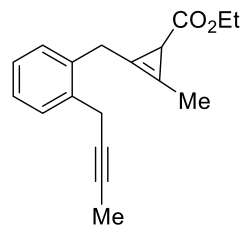

Prepared following the general procedure A, from 1,2-di(but-2-yn-1-yl)benzene (300 mg, 1.65 mmol), using ethyl diazoacetate (324 mg, 2.47 mmol, 87% purity) to afford 115 mg of **1l** (26% yield, pale yellow oil) after purification by flash chromatography on silica gel (100:5 hexane/EtOAc). R_f = 0.46 (8:2 hexane/EtOAc). **^{1}H NMR (300 MHz, CDCl$_3$):** *δ* 7.47 (m, 1H), 7.23 (m, 3H), 4.08 (q, *J* = 7.7 Hz, 2H), 3.84 (d, *J* = 17.6 Hz, 1H), 3.75 (d, *J* = 17.4 Hz, 1H), 3.53 (m, 2H), 2.12 (s, 1H), 2.01 (s, 3H), 1.84 (s, 3H), 1.21 (t, *J* = 7.1 Hz, 3H). **^{13}C NMR (75 MHz, CDCl$_3$):** *δ* 176.1 (CO), 135.7 (C), 134.8 (C), 129.4 (CH), 128.8 (CH), 127.0 (CH), 126.9 (CH), 104.8 (C), 103.8 (C), 78.0 (C), 76.2 (C), 59.8 (CH$_2$), 28.3 (CH$_2$), 23.1 (CH), 22.7 (CH$_2$), 14.3 (CH$_3$), 9.5 (CH$_3$), 3.5 (CH$_3$). **HRMS** (APCI-FIA-TOF): *m/z* calculated for $C_{18}H_{21}O_2$ [M + H]$^+$: 269.1536, found 269.1538.

Ethyl 2-(((4-methyl-*N*-(3-phenylprop-2-yn-1-yl)phenyl)sulfonamido)methyl)-3-phenylcycloprop-2-ene-1-carboxylate (1m)

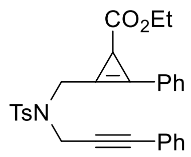

Prepared following the general procedure A, from 4-methyl-*N,N*-bis(3-phenylprop-2-yn-1-yl)benzenesulfonamide (300 mg, 0.75 mmol), using ethyl diazoacetate (148 mg, 1.12 mmol, 87% purity) to afford 225 mg of **1m** (62% yield, colourless oil) after purification by flash chromatography on silica gel (90:10 → 85:15 → 80:20 hexane/EtOAc). R_f = 0.4 (7:3 hexane/EtOAc). **^{1}H NMR (300 MHz, CDCl$_3$):** *δ* 7.84 (m, 2H), 7.50 (m, 2H), 7.39 (m, 3H), 7.35 – 7.23 (m, 5H), 7.11 (m, 2H), 4.65 (s, 2H), 4.54 (d, *J* = 18.4 Hz, 1H), 4.42 (d, *J* = 18.3 Hz, 1H), 4.13 (m, 2H), 2.55 (s, 1H), 2.36 (s, 3H), 1.24 (t, *J* = 7.1 Hz, 3H). **^{13}C NMR (75 MHz, CDCl$_3$):** 174.4 (CO), 143.8 (C), 135.8 (C), 131.5 (2CH), 129.9 (2CH), 129.6 (2CH), 129.5 (CH), 128.8 (2CH), 128.4 (CH), 128.1 (2CH), 127.8 (2CH), 125.8 (C), 122.1 (C), 110.4 (C), 104.3 (C), 86.1 (C), 81.4 (C), 60.4 (CH$_2$), 42.4 (CH$_2$), 37.8 (CH$_2$), 22.9 (CH), 21.4 (CH$_3$), 14.2 (CH$_3$). **HRMS** (APCI-FIA-TOF): *m/z* calculated for $C_{29}H_{28}NO_4S$ [M + H]$^+$: 486.1734, found 486.1727.

Ethyl 2-(((4-methyl-*N*-(3-(4-(trifluoromethyl)phenyl)prop-2-yn-1-yl)phenyl)sulfonamido)methyl)-3-(4-(trifluoromethyl)phenyl)cycloprop-2-ene-1-carboxylate (1n)

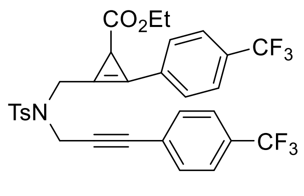

Prepared following the general procedure A (the reaction was carried out at 45 °C in this case), from 4-methyl-*N*,*N*-bis(3-(4-(trifluoromethyl)phenyl)prop-2-yn-1-yl)benzenesulfonamide (255 mg, 0.48 mmol), using ethyl diazoacetate (94 mg, 0.72 mmol, 87% purity) to afford 214 mg of **1n** (72% yield, pale yellow oil) after purification by flash chromatography on silica gel (9:1 → 8:2 hexane/EtOAc). R_f = 0.44 (8:2 hexane/EtOAc). **^{1}H NMR (300 MHz, CDCl$_3$):** *δ* 7.81 (m, 2H), 7.60 (m, 2H), 7.49 (m, 2H), 7.27 (m, 2H), 7.18 (m, 2H), 4.70 (d, *J* = 17.2 Hz, 1H), 4.61 (d, *J* = 17.2 Hz, 1H), 4.52 (d, *J* = 18.6 Hz, 1H), 4.42 (d, *J* = 18.5 Hz, 1H), 4.13 (m, 2H), 2.61 (s, 1H), 2.34 (s, 3H), 1.23 (t, *J* = 7.3 Hz, 3H). **^{13}C NMR (75 MHz, CDCl$_3$):** 173.8 (CO), 144.1 (C), 135.7 (C), 131.7 (2CH),

130.0 (2CH), 129.7 (2CH), 127.7 (2CH), 125.7 (q, *J* = 3.9 Hz, 2CH), 125.0 (q, *J* = 3.3 Hz, 2CH), 123.7 (q, *J* = 272.5 Hz, CF_3), 123.7 (q, *J* = 272.1 Hz, CF_3), 109.1 (C), 107.9 (C), 84.7 (C), 84.0 (C), 60.6 (CH_2), 42.9 (CH_2), 38.0 (CH_2), 23.0 (CH), 21.3 (CH_3), 14.2 (CH_3). **^{19}F NMR (282 MHz, CDCl$_3$):** *δ* -62.9 (s, 3F), -63.0 (s, 3F). **HRMS** (APCI-FIA-TOF): *m/z* calculated for $C_{31}H_{26}F_6NO_4S$ $[M + H]^+$: 622.1481, found 622.1494.

Ethyl 2-(((*N*-(3-(4-methoxyphenyl)prop-2-yn-1-yl)-4-methylphenyl)sulfonamido)methyl)-3-(4-(trifluoromethyl)phenyl)cycloprop-2-ene-1-carboxylate (1o)

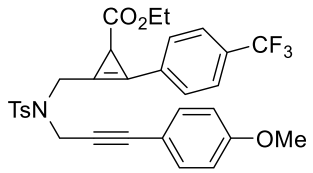

Prepared following the general procedure A (the reaction was carried out at 45 °C in this case), from *N*-(3-(4-methoxyphenyl)prop-2-yn-1-yl)-4-methyl-*N*-(3-(4-(trifluoromethyl)phenyl)prop-2-yn-1-yl)benzenesulfonamide (400 mg, 0.80 mmol), using ethyl diazoacetate (158 mg, 1.20 mmol, 87% purity) to afford 251 mg of **1o** (53% yield, yellow oil) after purification by flash chromatography on silica gel (9:1 → 8:2 hexane/EtOAc). R_f = 0.44 (7:3 hexane/EtOAc). **^{1}H NMR (500 MHz, CDCl$_3$):** *δ* 7.81 (m, 2H), 7.51 (m, 2H), 7.40 (m, 2H), 7.27 (m, 2H), 7.20 (m, 2H), 6.90 (m, 2H), 4.63 (d, *J* = 16.4 Hz, 1H), 4.57 (d, *J* = 16.4 Hz, 1H), 4.52 (d, *J* = 18.5 Hz, 1H), 4.43 (d, *J* = 18.4 Hz, 1H), 4.12 (m, 2H), 3.83 (s, 3H), 2.49 (s, 1H), 2.35 (s, 3H), 1.22 (t, *J* = 7.1 Hz, 3H). **^{13}C NMR (125 MHz, CDCl$_3$):** 174.7 (CO), 160.8 (C), 143.9 (C), 135.8 (C), 131.8 (2CH), 131.5 (2CH), 130.2 (q, *J* = 33.3 Hz, C), 129.7 (2CH), 127.8 (2CH), 125.9 (C), 125.0 (q, *J* = 3.8 Hz, 2CH), 123.7 (q, *J* = 272.0 Hz, CF_3), 118.3 (C), 114.4 (2CH), 110.1 (C), 100.9 (C), 84.5 (C), 84.3 (C), 60.4 (CH_2), 55.4 (CH_3), 42.6 (CH_2), 37.7 (CH_2), 22.9 (CH), 21.4 (CH_3), 14.3 (CH_3). **^{19}F NMR (470 MHz, CDCl$_3$):** *δ* -62.9 (s, 3F). **HRMS** (APCI-FIA-TOF): *m/z* calculated for $C_{31}H_{29}F_3NO_5S$ $[M + H]^+$: 584.1713, found 584.1726.

Ethyl 2-(((4-methyl-*N*-(3-(4-(trifluoromethyl)phenyl)prop-2-yn-1-yl)phenyl)sulfonamido)methyl)cycloprop-2-ene-1-carboxylate (1p)

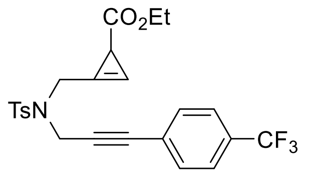

Prepared following the general procedure A, from 4-methyl-*N*-(prop-2-yn-1-yl)-*N*-(3-(4-(trifluoromethyl)phenyl)prop-2-yn-1-yl)benzenesulfonamide (450 mg, 1.15 mmol), using ethyl diazoacetate (226 mg, 1.72 mmol, 87% purity) to afford 191 mg of **1p** (34% yield, colorless oil) after purification by flash chromatography on silica gel (9:1 → 8:2 → 7:3 hexane/EtOAc). R_f = 0.37 (7:3 hexane/EtOAc). **^{1}H NMR (300 MHz, CDCl$_3$):** *δ* 7.77 (m, 2H), 7.50 (m, 2H), 7.29 (m, 4H), 6.56 (s, 1H), 4.50 (s, 2H), 4.42 (s, 2H), 4.12 (m, 2H), 2.34 (s, 3H), 2.22 (s, 1H), 1.22 (t, *J* = 7.3 Hz, 3H). **^{13}C NMR (75 MHz, CDCl$_3$):** *δ* 174.8 (CO), 143.8 (C), 135.7 (C), 131.7 (2CH), 130.0 (q, *J* = 32.5 Hz, C), 129.5 (2CH), 127.7 (2CH), 123.6 (q, *J* = 271.9 Hz, CF_3), 124.9 (q, *J* = 3.7 Hz, 2CH), 110.3 (C), 99.7 (CH), 84.4 (C), 84.2 (C), 60.3 (CH_2), 42.3 (CH_2), 37.5 (CH_2), 21.2 (CH_3), 20.4 (CH), 14.1 (CH_3). **^{19}F NMR (282 MHz, CDCl$_3$):** *δ* -62.9 (s, 3F). **HRMS** (APCI-FIA-TOF): *m/z* calculated for $C_{24}H_{23}F_3NO_4S$ $[M + H]^+$: 478.1294, found 478.1293.

Ethyl 2-(((4-methyl-*N*-(3-(perfluorophenyl)prop-2-yn-1-yl)phenyl)sulfonamido)methyl)cycloprop-2-ene-1-carboxylate (1q)

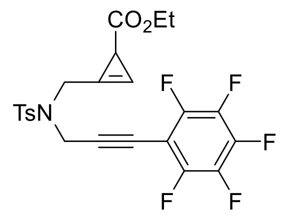

Prepared following the general procedure A, from 4-methyl-*N*-(3-(perfluorophenyl)prop-2-yn-1-yl)-*N*-(prop-2-yn-1-yl)benzenesulfonamide (300 mg, 0.72 mmol), using ethyl diazoacetate (143 mg, 1.09 mmol, 87% purity) to afford 170 mg of **1q** (47% yield, colorless oil) after purification by flash chromatography on silica gel (95:5 → 90:10 → 80:20 hexane/EtOAc). R_f = 0.5 (8:2 hexane/EtOAc). **^{1}H NMR (300 MHz, $CDCl_3$):** *δ* 7.73 (m, 2H), 7.25 (m, 2H), 6.56 (s, 1H), 4.47 (m, 4H), 4.08 (q, *J* = 6.4 Hz, 2H), 2.34 (s, 3H), 2.20 (s, 1H), 1.22 (t, *J* = 7.1 Hz, 3H). **^{13}C NMR (75 MHz, $CDCl_3$):** *δ* 174.8 (CO), 147.2 (m, CF), 144.0 (C), 141.6 (m, CF), 137.4 (m, CF), 135.5 (C), 129.5 (2CH), 127.6 (2CH), 110.2 (C), 100.2 (CH), 94.8 (C), 69.8 (C), 60.4 (CH_2), 42.3 (CH_2), 37.5 (CH_2), 21.2 (CH), 20.4 (CH_3), 14.1 (CH_3). **^{19}F NMR (282 MHz, $CDCl_3$):** *δ* -135.9 (m, 2F), -151.8 (t, *J* = 20.7 Hz, F), -161.7 (m, 2F). **HRMS** (APCI-FIA-TOF): *m/z* calculated for $C_{23}H_{18}F_5NO_4S$ $[M + H]^+$: 500.0949, found 500.0953.

Ethyl 2-(((4-methyl-*N*-(3-(thiophen-2-yl)prop-2-yn-1-yl)phenyl)sulfonamido)methyl)cycloprop-2-ene-1-carboxylate (1r)

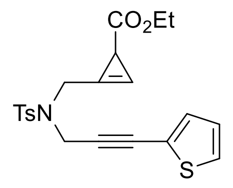

Prepared following the general procedure A, from 4-methyl-*N*-(prop-2-yn-1-yl)-*N*-(3-(thiophen-2-yl)prop-2-yn-1-yl)benzenesulfonamide (305 mg, 0.92 mmol), using ethyl diazoacetate (182 mg, 1.39 mmol, 87% purity) to afford 52 mg of **1r** (13% yield, pale yellow oil) after purification by flash chromatography on silica gel (95:5 → 90:10 → 80:20 hexane/EtOAc). R_f = 0.22 (8:2 hexane/EtOAc). **^{1}H NMR (300 MHz, $CDCl_3$):** *δ* 7.77 (m, 2H), 7.30 (m, 2H), 7.22 (dd, *J* = 5.1, 1.2 Hz, 1H), 6.99 (dd, *J* = 3.6, 1.2 Hz, 1H), 6.92 (dd, *J* = 5.1, 3.6 Hz, 1H), 6.56 (d, *J* = 1.5 Hz, 1H), 4.47 (s, 2H), 4.43 (d, *J* = 2.1 Hz, 2H), 4.12 (qd, *J* = 7.1, 2.0 Hz, 2H), 2.40 (s, 3H), 2.24 (d, *J* = 1.4 Hz, 1H), 1.25 (t, *J* = 7.1 Hz, 3H). **^{13}C NMR (75 MHz, $CDCl_3$):** *δ* 175.0 (CO), 143.9 (C), 135.7 (C), 132.4 (CH), 129.7 (2CH), 127.8 (2CH), 127.4 (CH), 126.8 (CH), 122.0 (C), 110.6 (C), 99.8 (CH), 85.5 (C), 79.2 (C), 60.5 (CH_2), 42.5 (CH_2), 38.0 (CH_2), 21.5 (CH), 20.6 (CH_3), 14.3 (CH_3). **HRMS** (APCI-FIA-TOF): *m/z* calculated for $C_{21}H_{22}NO_4S_2$ $[M + H]^+$: 416.0985, found 416.1000.

N-(But-2-yn-1-yl)-N-((3-(4-chlorobenzoyl)-2-methylcycloprop-1-en-1-yl)methyl)-4-methylbenzene sulfonamide (1v)

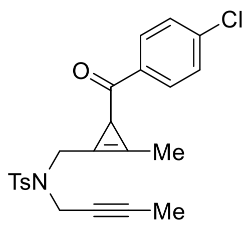

Prepared following the general procedure A, from *N,N*-di(but-2-yn-1-yl)-4-methylbenzenesulfonamide (200 mg, 0.72 mmol), using 1-chloro-4-(diazomethyl)benzene (226 mg, 1.09 mmol) instead of ethyl diazoacetate to afford 113 mg of **1v** (36% yield, colorless oil) after purification by flash chromatography on silica gel (90:10 → 85:15 hexane/EtOAc). R_f = 0.26 (8:2 hexane/EtOAc). **^{1}H NMR (300 MHz, $CDCl_3$):** *δ* 7.89 (m, 2H), 7.70 (m, 2H), 7.41 (m, 2H), 7.23 (m, 2H), 4.35 (m, 2H), 4.06 (m, 2H), 3.04 (s, 1H), 2.36 (s, 3H), 1.98 (s 3H), 1.57 (m, 3H). **^{13}C NMR (75 MHz, $CDCl_3$):** *δ* 201.5 (CO), 143.4 (C), 138.7 (C), 136.5 (C), 136.0 (C), 129.4 (2CH), 129.3 (2CH), 128.6 (2CH), 127.7 (2CH), 107.0 (C), 100.5 (C), 81.8 (C), 71.6 (C), 41.4 (CH_2), 37.4 (CH_2), 27.6 (CH_3), 21.3 (CH), 9.6 (CH_3), 3.2 (CH_3). **HRMS** (APCI-FIA-TOF): *m/z* calculated for $C_{23}H_{23}ClNO_3S$ $[M + H]^+$: 428.1082, found 428.1090.

Ethyl 2-(((N,4-dimethylphenyl)sulfonamido)methyl)cycloprop-2-ene-1-carboxylate (1zd)

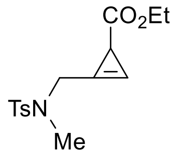

Prepared following the general procedure A, from *N*,4-dimethyl-*N*-(prop-2-yn-1-yl)benzenesulfonamide (250 mg, 1.12 mmol), using ethyl diazoacetate (191 mg, 1.45 mmol, 87% purity) to afford 120 mg of **1zd** (34% yield, colorless oil) after purification by flash chromatography on silica gel (85:15 → 80:20 hexane/EtOAc). R_f = 0.43 (7:3 hexane/EtOAc). **^{1}H NMR (300 MHz, $CDCl_3$):** *δ* 7.68 (m, 2H), 7.31 (m, 2H), 6.32 (s, 1H), 4.32 (d, *J* = 17.6 Hz, 1H), 4.22 (d, *J* = 17.6 Hz, 1H), 4.08 (m, 2H), 2.82 (s, 3H), 2.42 (s, 3H), 2.08 (s, 1H), 1.22 (t, *J* = 7.1 Hz, 3H). **^{13}C NMR (75 MHz, $CDCl_3$):** *δ* 174.9 (CO), 143.6 (C), 134.6 (C), 129.6 (2CH), 127.5 (2CH), 110.3 (C), 98.7 (CH), 60.3 (CH_2), 45.7 (CH_2), 34.8 (CH_3), 21.4 (CH), 20.1 (CH_3), 14.2 (CH_3). **HRMS** (APCI-FIA-TOF): *m/z* calculated for $C_{15}H_{20}NO_4S_2$ $[M + H]^+$: 310.1108, found 310.1110.

Precursor **1zb** was previously reported and the obtained NMR data is in accordance with the reported data.[3]

General procedure B: Cyclopropenation of propargyl malonates followed by propargylation (illustrated for 1s)

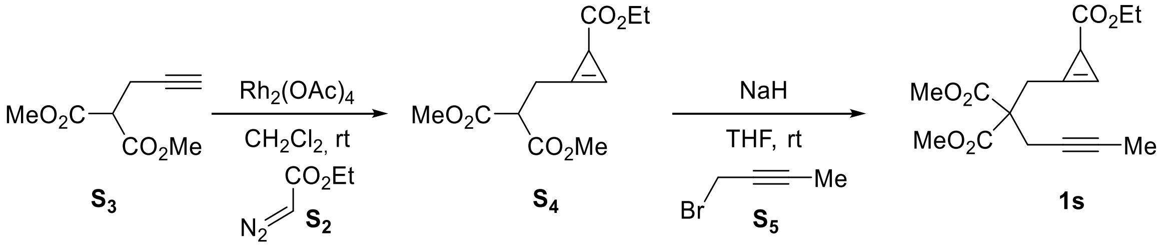

Following the General Procedure A, the reaction of dimethyl 2-(prop-2-yn-1-yl)malonate S_3 (2.5 g, 10 mmol), with ethyl diazoacetate S_2 (1.4 g, 12 mmol, 87% purity) afforded, after purification by flash chromatography

on silica gel (8:1 hexane/EtOAc), dimethyl 2-((3-(ethoxycarbonyl)cycloprop-1-en-1-yl)methyl)malonate **S4** (1.30 g, 52% yield, colorless oil).

NaH (103 mg, 2.58 mmol, 60% dispersion in mineral oil) was added in portions to a stirred solution of **S4** (600 mg, 2.34 mmol) in THF anh. (9.4 mL) at 0 °C. Once the addition was completed, 1-bromo-butyne **S5** (467 mg, 3.5 mmol, 0.30 ml) was added slowly and the mixture was allowed to warm up to room temperature and stirred overnight. The reaction was then quenched with H_2O, extracted with Et_2O and the combined organic phases were washed with brine. The organic phase was then dried over Na_2SO_4, filtered, and concentrated under vacuum. Then, the crude residue was purified by flash chromatography on silica gel (8:1 hexane:EtOAC) to afford 333 mg of dimethyl 2-(but-2-yn-1-yl)-2-((3-(ethoxycarbonyl)cycloprop-1-en-1-yl)methyl)malonate **1s** as a colorless oil (45% yield). R_f = 0.51 (8:2 hexane/EtOAc). **^{1}H NMR (300 MHz, CDCl$_3$):** δ 6.47 (s, 1H), 4.08 (m, 2H), 3.71 (s, 6H), 3.26 (s, 2H), 2.82 (m, 2H), 2.07 (m, 1H), 1.71 (m, 3H), 1.21 (m, 3H). **^{13}C NMR (75 MHz, CDCl$_3$):** δ 175.3 (CO), 169.6 (CO), 169.5 (CO), 111.1 (C), 97.8 (CH), 79.2 (C), 72.8 (C), 60.1 (CH_2), 56.0 (C), 52.8 ($2CH_3$), 28.1 (CH_2), 23.4 (CH_2), 19.5 (CH), 14.2 (CH_3), 3.3 (CH_3). **HRMS** (APCI-FIA-TOF): *m/z* calculated for $C_{16}H_{21}O_6$ [M + H]$^+$: 309.1333, found 309.1334.

Dimethyl 2-((3-(ethoxycarbonyl)cycloprop-1-en-1-yl)methyl)-2-(3-phenylprop-2-yn-1-yl)malonate (1t)

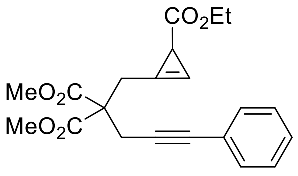

Prepared following the general procedure B, from dimethyl 2-((3-(ethoxycarbonyl)cycloprop-1-en-1-yl)methyl)malonate **S4** (128 mg, 0.5 mmol) and (3-bromoprop-1-yn-1-yl)benzene (117 mg, 0.75 mmol), to afford **1t** (74.0 mg, 40% yield, colorless oil) after purification by flash chromatography on silica gel (9:1 hexane/Et_2O). R_f = 0.57 (8:2 hexane/Et_2O). **^{1}H NMR (300 MHz, CDCl$_3$):** δ 7.35 (m, 2H), 7.26 (m, 3H), 6.54 (s, 1H), 4.10 (q, *J* = 7.2 Hz, 2H), 3.77 (s, 6H), 3.37 (s, 2H), 3.13 (d, *J* = 7.6 Hz, 2H), 2.14 (s, 1H), 1.23 (t, *J* = 7.2 Hz, 3H). **^{13}C NMR (75 MHz, CDCl$_3$):** δ 175.4 (CO), 169.5 (CO), 169.4 (CO), 131.6 (2CH), 128.2 (2CH), 128.1 (CH), 122.9 (C), 111.0 (C), 98.2 (CH), 83.9 (C), 83.7 (C), 60.3 (CH_2), 56.1 (C), 53.0 ($2CH_3$), 28.3 (CH_2), 24.0 (CH_2), 19.6 (CH), 14.2 (CH_3). **HRMS** (APCI-FIA-TOF): *m/z* calculated for $C_{21}H_{23}O_6$ [M + H]$^+$: 371.1489, found 371.1497.

(2*R*,5*S*)-2-*iso*Propyl-5-methylcyclohexyl 2-(((4-methyl-*N*-(pent-2-yn-1-yl)phenyl)sulfonamido)methyl)cyclo-prop-2-ene-1-carboxylate (1u)

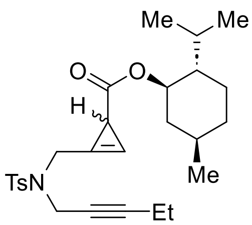

dr = 1:1

Prepared following the general procedure B, using (1*R*,2*S*,5*R*)-2-isopropyl-5-methylcyclohexyl 2-diazoacetate instead of ethyl diazoacetate **S2**, from **S4-Menthol** (183 mg, 0.5 mmol) using 1-bromopent-2-yne (110 mg, 0.75 mmol) to afford 190 mg of **1u** (88% yield, colorless oil, dr = 1:1) after purification by flash chromatography on silica gel (9:1 hexane/Et_2O). R_f = 0.35 (8:2 hexane/Et_2O). **^{1}H NMR (300 MHz, CDCl$_3$):** δ 6.49 (s, 1H), 6.46 (s, 1H), 4.65 (m, 2H), 3.74 (s, 12H), 3.27 (s, 3H), 2.97 – 2.73 (m, 4H), 2.10

(m, 5H), 1.97 (m, 2H), 1.83 (m, 2H), 1.64 (m, 6H), 1.54 – 1.24 (m, 6H), 1.07 (m, 6H), 0.94 – 0.84 (m, 12H), 0.75 (d, *J* = 6.9 Hz, 6H). **^{13}C NMR (75 MHz, $CDCl_3$):** δ 175.04 (CO), 174.84 (CO), 169.7 (C), 169.62 (C), 111.52 (C), 111.21 (C), 98.09 (CH), 97.88 (CH), 85.41 (C), 85.36 (C), 73.98 (CH), 73.17 (C), 56.20 (C), 56.15 (C), 52.89 (CH), 52.86 (CH), 47.14 (CH), 47.08 (CH), 41.09 (CH_2), 40.95 (CH_2), 34.30 (CH_2), 31.35 (CH), 31.32 (CH), 28.19 (CH_2), 28.15 (CH_2), 26.31 (CH), 26.20 (CH), 23.59 (CH_2), 23.47 (CH_2), 23.35 (CH_2), 22.00 (CH_3), 20.74 (CH), 20.69 (CH), 19.78 (CH), 19.73 (CH), 16.48 (CH_3), 16.34 (CH_3), 14.07 (CH_3), 12.29 (CH_2). **HRMS** (APCI-FIA-TOF): *m/z* calculated for $C_{25}H_{37}O_6$ $[M + H]^+$: 433.2585, found 433.2585.

General procedure C: Cyclopropenation of diynes with trifluoromethyl diazomethane (illustrated for **1z**)

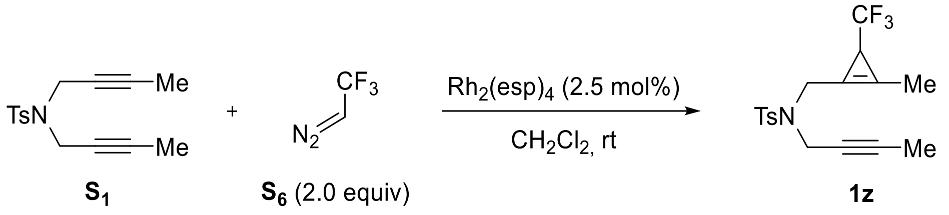

[$Rh_2(esp)_4$] (14.0 mg, 0.018 mmol, 2.5 mol %) was added to a flame dried Schlenk tube and freshly distilled CH_2Cl_2 (1.0 mL). The homogeneous green solution was stirred for 5 min before a *N,N*-di(but-2-yn-1-yl)-4-methylbenzenesulfonamide **S_1** (200 mg, 0.73 mmol) was added. 2-Diazo-1,1,1-trifluoroethane **S_6** (160 mg, 1.46 mmol, 2.9 mL, 0.5M solution in CH_2Cl_2) was added to this solution through a syringe pump at a rate of 0.5 mL/h, at rt. Upon completion of the addition, the solvent was removed under reduced pressure. The crude residue was purified by flash chromatography on silica gel (95:5 $\rightarrow$ 90:10 hexane/EtOAc) to afford 150 mg of *N*-(but-2-yn-1-yl)-4-methyl-*N*-((2-methyl-3-(trifluoromethyl)cycloprop-1-en-1-yl)methyl)benzenesulfonamide **1z**, as a pale yellow oil (58% yield). R_f = 0.49 (8:2 hexane/EtOAc). **^{1}H NMR (500 MHz, $CDCl_3$):** δ 7.72 (m, 2H), 7.31 (m, 2H), 4.34 (m, 2H), 4.06 (dt, *J* = 3.4, 1.7 Hz, 2H), 2.43 (s, 3H), 1.93 (t, *J* = 1.7 Hz, 3H), 1.90 (q, *J* = 4.4 Hz, 1H), 1.62 (t, *J* = 2.4 Hz, 3H). **^{13}C NMR (125 MHz, $CDCl_3$):** δ 143.6 (C), 135.8 (C), 129.4 (2CH), 126.3 (q, *J* = 274.7 Hz, CF_3), 127.7 (2CH), 107.2 (q, *J* = 2.8 Hz, C), 102.0 (q, *J* = 2.8 Hz, C), 82.2 (C), 71.1 (C), 41.8 (CH_2), 37.5 (CH_2), 22.1 (q, *J* = 38.7 Hz, CH), 21.4 (CH_3), 9.4 (CH_3), 3.2 (CH_3). **^{19}F NMR (470 MHz, $CDCl_3$):** δ -66.2 (s, 3F). **HRMS** (APCI-FIA-TOF): *m/z* calculated for $C_{17}H_{19}F_3NO_2S$ $[M + H]^+$: 358.1083, found 358.1089.

4-Methyl-N-((3-(trifluoromethyl)cycloprop-1-en-1-yl)methyl)-N-(3-(4-(trifluoromethyl)phenyl)prop-2-yn-1-yl)benzenesulfonamide (1za)

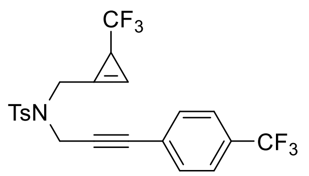

Prepared following the general procedure C, from 4-methyl-*N*-(prop-2-yn-1-yl)-*N*-(3-(4-(trifluoromethyl)phenyl)prop-2-yn-1-yl)benzenesulfonamide (300 mg, 0.77 mmol), using 2-diazo-1,1,1-trifluoroethane (169 mg, 1.53 mmol, 3.1 ml, 0.5 M solution in CH_2Cl_2) to afford 120 mg of **1za** (33% yield, colorless oil) after purification by flash chromatography on silica gel (6:4 → 1:1 hexane/CH_2Cl_2). R_f = 0.5 (4:6 hexane/CH_2Cl_2). **^{1}H NMR (500 MHz, $CDCl_3$):** δ 7.78 (m, 2H), 7.53 (m, 2H), 7.30 (m, 2H), 7.25 (m, 2H), 6.58 (p, *J* = 1.6 Hz, 1H), 4.51 (m, 2H), 4.42 (m, 2H), 2.38 (s, 3H), 2.12 (qd, *J* = 4.3, 1.4 Hz, 1H). **^{13}C NMR (125 MHz, $CDCl_3$):** δ 144.1 (C), 135.6 (C), 131.8 (2CH), 130.4 (q, *J* = 32.7 Hz, C), 129.7 (2CH), 127.8 (2CH), 125.7 (C), 125.6 (q, *J* = 275.2 Hz, CF_3), 125.1 (q, *J* = 3.7 Hz, 2CH), 123.7 (q, *J* = 272.5 Hz, CF_3), 111.9 (q, *J* = 2.9 Hz, C), 99.1 (q, *J* = 3.2 Hz, CH), 84.7 (C), 83.6 (C), 42.7 (CH_2), 37.7 (CH_2), 21.4 (CH_3), 20.2 (q, *J* = 39.9 Hz, CH). **^{19}F NMR (470 MHz, $CDCl_3$):** δ -62.9 (s, 3F), -67.3 (s, 3F). **HRMS** (APCI-FIA-TOF): *m/z* calculated for $C_{22}H_{18}F_6NO_2S$ $[M + H]^+$: 474.0957, found 474.0968.

Procedure for the preparation of ethyl 2-(((*N*-(1-ethynylcyclohexyl)-4-methylphenyl)sulfonamido)methyl)-3-methylcycloprop-2-ene-1-carboxylate (1h)

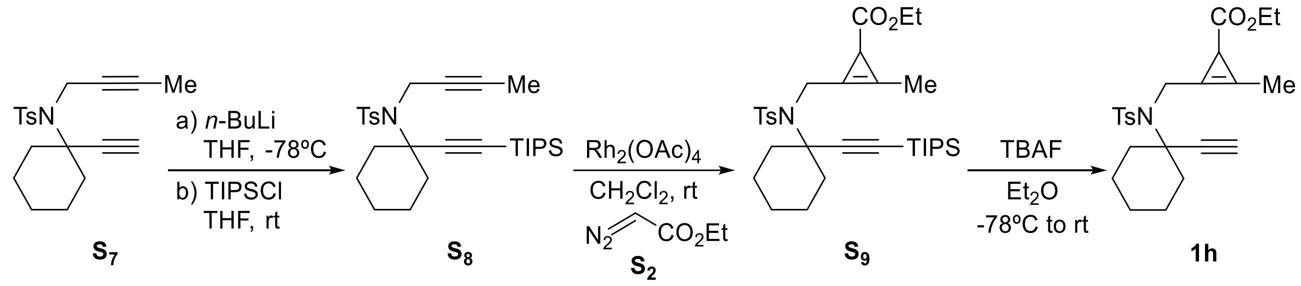

n-BuLi (1.10 mL, 2.87 mmol, 2.5 M solution in THF) was added to a solution of dimethyl *N*-(but-2-yn-1-yl)-4-methyl-*N*-(1-((triisopropylsilyl)ethynyl)cyclohexyl)benzenesulfonamide **S7** (630 mg, 1.9 mmol) in THF (3.8 mL) at -78 °C. After being stirred for 30 min, triisopropylsilyl chloride (553 mg, 2.87 mmol) was added and the resulting mixture was gradually warmed to room temperature and stirred overnight. The reaction was quenched by addition of saturated aqueous solution of NH_4Cl and extracted with EtOAc. The organic phases were washed with water and brine, dried over Na_2SO_4, and concentrated. The resulting crude residue was purified by flash chromatography on silica gel (100:2 → 90:10 hexane/EtOAc) to afford 730 mg of *N*-(but-2-yn-1-yl)-4-methyl-*N*-(1-((triisopropylsilyl)ethynyl)cyclohexyl) benzenesulfonamide **S8** as a pale yellow oil (79% yield).

Following the General Procedure A, *N*-(but-2-yn-1-yl)-4-methyl-*N*-(1-((triisopropylsilyl)ethynyl) cyclohexyl) benzenesulfonamide **S8** (660 mg, 1.36 mmol) was treated with ethyl diazoacetate **S2** (356 mg, 2.72 mmol, 87% purity) to afford, after purification by flash chromatography on silica gel (95:5 → 90:10 hexane/EtOAc), ethyl

2-methyl-3-(((4-methyl-*N*-(1-((triisopropylsilyl)ethynyl) cyclohexyl)phenyl)sulfonamido)methyl)cycloprop-2-ene-1-carboxylate, **S₉**, as a pale yellow oil (113 mg, 14% yield)

TBAF (41.0 mg, 0.16 mmol, 0.16 mL, 1.0 M solution in THF) was added to a solution of ethyl 2-methyl-3-(((4-methyl-*N*-(1-((triisopropylsilyl)ethynyl)cyclohexyl)phenyl)sulfonamido)methyl)cycloprop-2-ene-1-carboxylate **S_9** (82.0 mg, 0.14 mmol) in Et_2O (1.4 mL) at -78 °C. The resulting mixture was slowly warmed to rt. Once the reaction was complete (confirmation by TLC analysis), the solvent was removed under reduced pressure. The resulting crude residue was purified by flash chromatography on silica gel (90:10 → 85:15 hexane/EtOAc) to afford 59.0 mg of ethyl 2-(((*N*-(1-ethynylcyclohexyl)-4-methylphenyl)sulfonamido)methyl)-3-methylcycloprop-2-ene-1-carboxylate, **1h**, as a colorless oil (99% yield). R_f = 0.24 (8:2 hexane/EtOAc). **^{1}H NMR (300 MHz, $CDCl_3$):** *δ* 7.75 (m, 2H), 7.26 (m, 2H), 4.58 (m, 2H), 4.12 (q, *J* = 7.1 Hz, 2H), 2.41 (s, 4H), 2.20 (m, 2H), 2.07 (m, 4H), 1.90 (dtd, *J* = 24.9, 12.1, 4.3 Hz, 2H), 1.61 (m, 5H), 1.25 (t, *J* = 7.1 Hz, 3H), 1.11 (m, 1H). **^{13}C NMR (75 MHz, $CDCl_3$):** *δ* 175.8 (CO), 143.0 (C), 139.7 (C), 129.3 (2CH), 127.3 (2CH), 105.6 (C), 104.7 (C), 83.4 (C), 75.2 (CH), 62.1 (C), 59.9 (CH_2), 42.5 (CH_2), 37.4 (CH_2), 37.1 (CH_2), 24.7 (CH_2), 24.0 (CH), 23.6 (CH_2), 23.5 (CH_2), 21.4 (CH_3), 14.4 (CH_3), 9.6 (CH_3). **HRMS** (APCI-FIA-TOF): *m/z* calculated for $C_{23}H_{30}NO_4S$ $[M + H]^+$: 416.1890, found 416.1905.

Procedure for the preparation of 2-(((*N*-(but-2-yn-1-yl)-4-methylphenyl)sulfonamido) methyl)-3-methyl-*N*-phenylcycloprop-2-ene-1-carboxamide (1w)

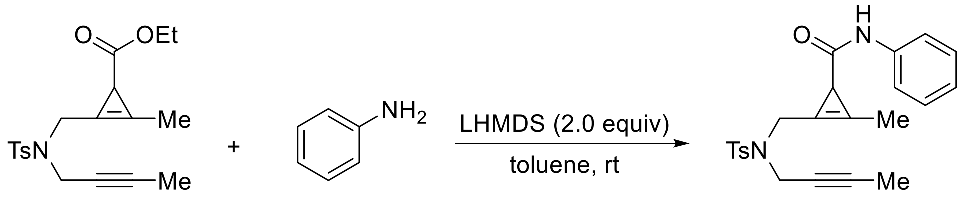

1a S_{10} (1.2 equiv) **1w**

1x was prepared following an adapted procedure of Szostak and coworkers.[4] An oven-dried vial equipped with a stir bar was charged with ethyl 2-(((*N*-(but-2-yn-1-yl)-4-methylphenyl)sulfonamido)methyl)-3-methylcycloprop-2-ene-1-carboxylate **1a** (100 mg, 0.28 mmol), aniline **S_{10}** (31 mg, 0.33 mmol). It was placed under a positive pressure of argon and subjected to three evacuation/backfilling cycles. Toluene (1.1 mL) and LiHMDS (93 mg, 0.55 mmol, 0.55 mL, 1.0 M solution in THF) were sequentially added with vigorous stirring at room temperature, and the reaction was stirred until TLC analysis showed complete conversion. Then, the reaction mixture was quenched with NH_4Cl (aq., 1.0 M), extracted with EtOAc and the organic layer was washed with water, brine, dried over Na_2SO_4 and concentrated. The crude residue was purified by flash chromatography on silica gel (8:2 → 7:3 hexane/EtOAc) to afford 33.0 mg of 2-(((*N*-(but-2-yn-1-yl)-4-methylphenyl)sulfonamido)methyl)-3-methyl-*N*-phenylcycloprop-2-ene-1-carboxamide **1w,** as a pale yellow oil (29% yield). R_f = 0.17 (7:3 hexane/EtOAc). **^{1}H NMR (300 MHz, $CDCl_3$):** *δ* 7.74 (m, 2H), 7.58 (m, 2H), 7.29 (m,

5H), 4.31 (s, 2H), 4.14 (s, 2H), 2.41 (s, 3H), 2.27 (s, 1H), 2.11 (s, 3H), 1.61 (s, 3H). **^{13}C NMR (75 MHz, CDCl$_3$):** *δ* 173.6 (CO), 143.8 (C), 138.4 (C), 135.6 (C), 129.5 (2CH), 128.8 (2CH), 127.7 (2CH), 123.6 (CH), 119.4 (2CH), 109.5 (C), 103.6 (C), 82.2 (C), 71.7 (C), 42.3 (CH$_2$), 38.1 (CH$_2$), 26.8 (CH$_3$), 21.4 (CH), 9.8 (CH$_3$), 3.3 (CH$_3$). **HRMS** (APCI-FIA-TOF): *m/z* calculated for $C_{23}H_{25}N_2O_3S$ [M + H]$^+$: 409.1580, found 409.1582.

Procedure for the preparation of of *N*-(but-2-yn-1-yl)-*N*-((3-(hydroxymethyl)-2-methylcycloprop-1-en-1-yl)methyl)-4-methylbenzenesulfonamide (1y)

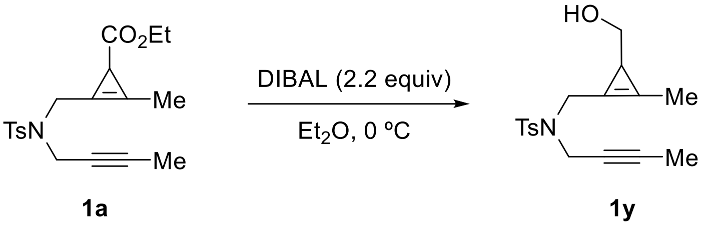

Alkynyl-tethered cyclopropane **1a** (230 mg, 0.636 mmol**)** was dissolved in Et_2O (0.64 ml) in a flame dried flask and cooled to 0 °C. DIBAL (1.4 ml, 1.4 mmol, 1.0 M solution in hexane) was added dropwise and the reaction mixture was stirred at 0 °C until full consumption of alkynyl-tethered cyclopropane (typically 30 min). The reaction was diluted with Et_2O and quenched with water and the mixture was stirred until the two phases were separated. The aqueous layer was extracted with Et_2O, and the combined organic phases were dried over Na_2SO_4, filtered, and concentrated under reduced pressure. The crude was purified by flash column chromatography on silica gel (6:4 $\rightarrow$ 1:1 hexane/EtOAc) to afford 103 mg of alcohol **1y** as a colorless oil (52% yield). R_f = 0.1 (7:3 hexane/EtOAc). **^{1}H NMR (300 MHz, CDCl$_3$):** *δ* 7.75 (m, 2H), 7.31 (m, 2H), 4.31 (d, *J* = 1.7 Hz, 2H), 4.09 (m, 2H), 3.60 (dd, *J* = 10.9, 3.8 Hz, 1H), 3.41 (dd, *J* = 10.9, 4.8 Hz, 1H), 2.44 (s, 3H), 1.99 (m, 3H), 1.62 (m, 4H). **^{13}C-NMR (75 MHz, CDCl$_3$):** *δ* 143.5 (C), 136.0 (C), 129.4 (2CH), 127.8 (2CH), 115.2 (C), 109.6 (C), 81.8 (C), 71.8 (C), 67.6 (CH$_2$), 42.6 (CH$_2$), 37.3 (CH$_2$), 23.6 (CH), 21.5 (CH$_3$), 10.7 (CH$_3$), 3.3 (CH$_3$). **HRMS** (APCI-FIA-TOF): *m/z* calculated for $C_{17}H_{22}NO_3S$ [M+H]$^+$: 320.1315, found 320.1306.

Procedure for the preparation of *N*-((3-(methoxymethyl)-2-methylcycloprop-1-en-1-yl)methyl)-4-methyl-*N*-(prop-2-yn-1-yl)benzenesulfonamide (1x)

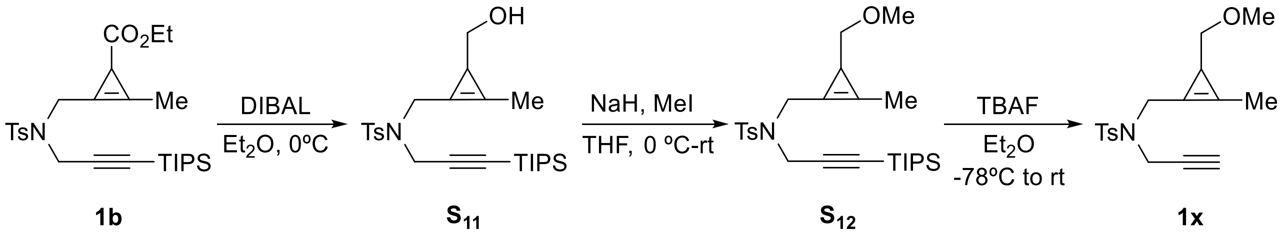

Following the procedure for **1z** synthesis, *N*-((3-(hydroxymethyl)-2-methylcycloprop-1-en-1-yl)methyl)-4-methyl-*N*-(3-(triisopropylsilyl)prop-2-yn-1-yl)benzenesulfonamide **S$_{11}$** was obtained from **1b** (577 mg, 1.14

mmol) and DIBAL-H (358 mg, 2.52 mmol, 2.5 mL, 1.0 M solution in hexane), after purification by flash chromatography on silica gel (85:15 → 80:20 hexane/EtOAc) (350 mg, 66% yield).

A suspension of NaH (22 mg, 0.54 mmol, 60% dispersion in mineral oil) in THF was added to a solution of *N*-((3-(hydroxymethyl)-2-methylcycloprop-1-en-1-yl)methyl)-4-methyl-*N*-(3-(triisopropylsilyl)prop-2-yn-1-yl)benzenesulfonamide **S_{11}** (210 mg, 0.45 mmol) in THF (21 mL, 0.15 M) at 0 °C, and the mixture was stirred for 30 min. Iodomethane (97 mg, 0.68 mmol, 42 µL) was then added dropwise and the reaction mixture was stirred for 24 h at rt. The reaction was then quenched with H_2O, extracted with Et_2O and the combined organic phases were washed with brine. The organic phase was then dried over Na_2SO_4, filtered, and concentrated under vacuum. The crude was purified by flash chromatography on silica gel (9:1 → 8:2 hexane:EtOAc) to afford 100 mg of *N*-((3-(methoxymethyl)-2-methylcycloprop-1-en-1-yl)methyl)-4-methyl-*N*-(3-(triisopropylsilyl)prop-2-yn-1-yl)benzenesulfonamide **S_{12}** as a colorless oil (47% yield).

TBAF (60 mg, 0.23 mmol, 0.23 mL, 1.0 M solution in THF) was added to a solution of *N*-((3-(methoxymethyl)-2-methylcycloprop-1-en-1-yl)methyl)-4-methyl-*N*-(3-(triisopropylsilyl)prop-2-yn-1-yl)benzenesulfonamide **S_{12}** (100 mg, 0.14 mmol) in Et_2O (2.1 mL) at -78 °C. The resulting mixture was slowly warmed to rt and stirred until TLC showed complete conversion. Then, the solvent was removed under reduced pressure and the crude residue was purified by flash chromatography on silica gel (9:1 → 7:3 hexane/EtOAc) to afford 66 mg of *N*-((3-(methoxymethyl)-2-methylcycloprop-1-en-1-yl)methyl)-4-methyl-*N*-(prop-2-yn-1-yl)benzenesulfonamide **1x** as a colorless oil (99% yield). R_f = 0.2 (8:2 hexane/EtOAc). **^{1}H NMR (300 MHz, CDCl$_3$):** δ 7.72 (m, 2H), 7.29 (m, 2H), 4.35 (d, *J* = 1.8 Hz, 2H), 4.15 (m, 2H), 3.27 (s, 3H), 3.23(m, 1H), 3.16 (dd, *J* = 10.0, 5.1 Hz, 1H), 2.42 (s, 3H), 2.09 (t, *J* = 2.5 Hz, 1H), 1.94 (s, 3H), 1.55 (t, *J* = 4.9 Hz, 1H). **^{13}C NMR (75 MHz, CDCl$_3$):** δ 143.5 (C), 135.9 (C), 129.4 (2CH), 127.6 (2CH), 115.5 (C), 108.9 (C), 78.7 (CH_2), 76.5 (C), 73.7 (CH), 58.2 (CH_3), 42.7 (CH_2), 36.5 (CH_2), 21.4 (CH_3), 20.8 (CH_3), 10.6 (CH_3). **HRMS** (APCI-FIA-TOF): *m/z* calculated for $C_{17}H_{22}NO_3S$ $[M + H]^+$: 320.1315, found 320.1300.

Procedure for the preparation of *N*-((3,3-dimethylcycloprop-1-en-1-yl)methyl)-4-methyl-*N*-(3-phenylprop-2-yn-1-yl)benzenesulfonamide (1zc)

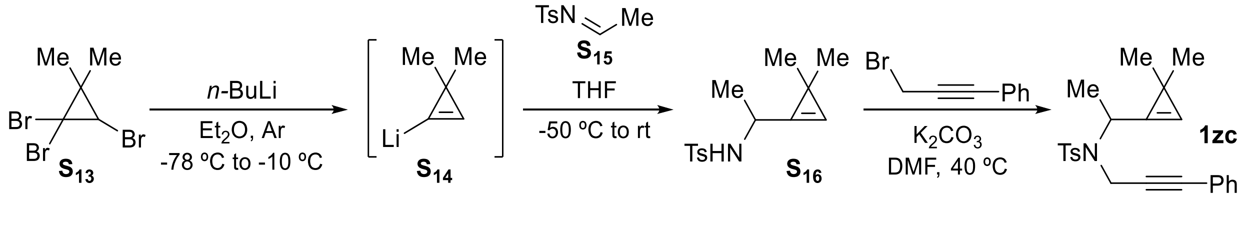

n-BuLi (0.47 g, 7.3 mmol, 2.9 mL, 2.5 M solution in hexanes) was added dropwise at -78 °C to a solution of 1,1,3-tribromo-2,2-dimethylcyclopropane **S_{13}** (1.12 g, 3.65 mmol) in Et_2O (9.1 mL). The resulting mixture was warmed to -10 °C, stirred for 1h at that temperature and cooled down to -50 °C. Then, a solution of *N*-ethylidene-4-methylbenzenesulfonamide **S_{15}** (0.94 g, 4.75 mmol) in THF (4.1 mL) was added and the mixture

was allowed to warm to rt. After 0.5 h at rt, H_2O and Et_2O were added. The layers were separated and the aqueous phase was extracted with Et_2O. The combined organic extracts were washed with brine, dried over Na_2SO_4, filtered and concentrated. The crude residue was purified by flash column chromatography on silica gel (95:5 → 90:10 hexane/EtOAc) to afford 108 mg of *N*-(1-(3,3-dimethylcycloprop-1-en-1-yl)ethyl)-4-methylbenzenesulfonamide **S16** as a pale yellow solid (11% yield).

3-Bromoprop-1-yn-1-yl)benzene (88 mg, 0.45 mmol) was added to a mixture of K_2CO_3 (156 mg, 1.13 mmol) and *N*-(1-(3,3-dimethylcycloprop-1-en-1-yl)ethyl)-4-methylbenzenesulfonamide **S16** (60 mg, 0.23 mmol), in DMF (3.1 mL) at 40 °C, . After being stirred for 7 h at this temperature, the reaction mixture was partitioned between EtOAc and H_2O. The layers were separated and the organic extracts were successively washed with brine, dried over Na_2SO_4, filtered and concentrated under reduced pressure. The crude residue was purified by flash column chromatography on silica gel (95:2 hexane/EtOAc) to afford 32.0 mg of *N*-(1-(3,3-dimethylcycloprop-1-en-1-yl)ethyl)-4-methyl-*N*-(3-phenylprop-2-yn-1-yl)benzenesulfonamide **1zc** as a colorless oil (37% yield). R_f = 0.53 (8:2 hexane/EtOAc). **^{1}H NMR (300 MHz, $CDCl_3$):** *δ* 7.83 (m, 2H), 7.27 (m, 7H), 6.92 (s, 1H), 5.15 (q, *J* = 7.1 Hz, 1H), 4.47 (d, *J* = 18.5 Hz, 1H), 4.14 (d, *J* = 18.5 Hz, 1H), 2.38 (s, 3H), 1.50 (d, *J* = 7.2 Hz, 3H), 1.12 (s, 3H), 1.10 (s, 3H). **^{13}C NMR (75 MHz, $CDCl_3$):** *δ* 143.13 (CO), 137.92 (C), 133.78 (C), 131.41 (2CH), 129.34 (2CH), 128.24 (CH), 128.15 (2CH), 127.50 (2CH), 122.71 (C), 117.02 (CH), 85.62 (C), 84.07 (C), 51.80 (CH), 33.85 (CH_2), 27.35 (CH_3), 26.96 (CH_3), 21.40 (CH_3), 20.71 (C), 19.01 (CH_3). **HRMS** (APCI-FIA-TOF): *m/z* calculated for $C_{23}H_{26}NO_2S$ $[M + H]^+$: 380.1679, found 380.1696.

Procedure for the preparation of deuterated precursors (*d*-1a and *d*-1y)

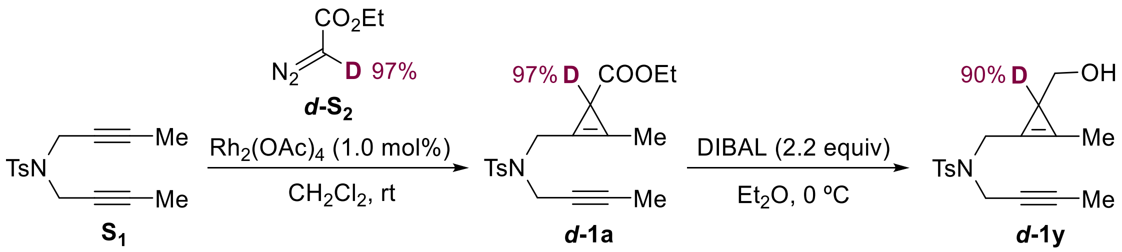

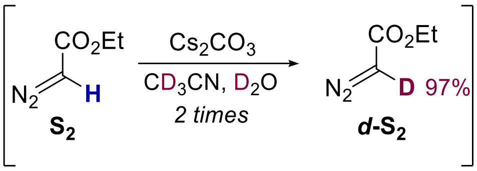

Deuterated ethyl diazoacetate ***d*-S2** was prepared following an adapted procedure of Mo and coworkers.[5] A dried flask equipped with a stir bar was charged with ethyl diazoacetate **S2** (1 g, 7.62 mmol, 87% purity), D_2O (1.5 g, 76.2 mmol, 1.5 mL), CD_3CN (3 mL) and cesium carbonate (3.7 g, 11.4 mmol). The mixture was stirred at room temperature for 24 h and the solvent was removed under reduced pressure at rt. The resulting crude residue was purified by flash column chromatography on silica gel (CH_2Cl_2), collecting the yellow fractions, to

afford ***d*-S$_2$** with 90% deuterium incorporation. The process was repeated using the previous partially deuterated ***d*-S2** (90% D), increasing the deuteration up to 97% ***d*-S2** (632 mg, 72% yield, 97% D, yellow oil).

Ethyl 2-(((*N*-(but-2-yn-1-yl)-4-methylphenyl)sulfonamido)methyl)-3-methylcycloprop-2-ene-1-carboxylate-1-*(**d*-1a**, 116 mg, 29% yield) was then prepared following the General Procedure A, from *N,N*-di(but-2-yn-1-yl)-4-methylbenzenesulfonamide **S$_1$** (300 mg, 1.09 mmol), using deuterated ethyl diazoacetate ***d*-S$_2$** (216 mg, 1.63 mmol, 97 % D) . R_f = 0.2 (8:2 hexane/EtOAc). **^{1}H NMR (300 MHz, CDCl$_3$):** δ 7.73 (m, 2H), 7.29 (m, 2H), 4.33 (s, 2H), 4.17 – 4.02 (m, 4H), 2.42 (s, 3H), 1.93 (s, 3H), 1.60 (m, 3H), 1.24 (m, 3H). **^{13}C NMR (75 MHz, CDCl$_3$):** δ 175.3 (CO), 143.4 (C), 136.0 (C), 129.3 (2CH), 127.8 (2CH), 107.4 (C), 101.2 (C), 81.8 (C), 71.5 (C), 60.0 (CH$_2$), 41.5 (CH$_2$), 37.3 (CH$_2$), 21.4 (CH$_3$), 14.3 (CH$_3$), 9.5 (CH$_3$), 3.2 (CH$_3$). **HRMS** (APCI-FIA-TOF): *m/z* calculated for $C_{19}H_{23}DNO_4S$ [M + H]$^+$: 363.1483, found 363.1491.

Following the same procedure as for the synthesis of **1y**, *N*-(but-2-yn-1-yl)-*N*-((3-(hydroxymethyl)-2-methylcycloprop-1-en-1-yl-3-*d*)methyl)-4-methylbenzenesulfonamide ***d*-1y** (90% deuterium content, 211 mg, 76% yield) was obtained as a colorless oil, from ethyl 2-(((*N*-(but-2-yn-1-yl)-4-methylphenyl)sulfonamido)methyl)-3-methylcycloprop-2-ene-1-carboxylate-1-*d* ***d*-1a** (314 mg, 0.87 mmol, 97% D) and DIBAL (271 mg, 1.90 mmol, 1.9 mL, 1.0 M solution in hexane). Purification was carried out by flash chromatography on silica gel (8:2 $\rightarrow$ 1:1 hexane/EtOAc). R_f = 0.1 (7:3 hexane/EtOAc). **^{1}H NMR (300 MHz, CDCl$_3$):** δ 7.75 (m, 2H), 7.31 (m, 2H), 4.29 (s, 2H), 4.06 (m, 2H), 3.56 (d, J = 10.9 Hz, 1H), 3.39 (dd, J = 11.0 Hz, 1H), 2.41 (s, 3H), 1.96 (s, 3H), 1.74 (s-broad, OH), 1.60 (t, J = 2.3 Hz, 3H). **^{13}C-NMR (75 MHz, CDCl$_3$):** δ 143.4 (C), 135.8 (C), 129.3 (2CH), 127.7 (2CH), 115.0 (C), 109.3 (C), 81.8 (C), 71.6 (C), 67.4 (CH$_2$), 42.5 (CH$_2$), 37.2 (CH$_2$), 23.6 (t, J = 25.5 Hz, CD), 21.4 (CH$_3$), 10.6 (CH$_3$), 3.2 (CH$_3$). **HRMS** (APCI-FIA-TOF): *m/z* calculated for $C_{17}H_{21}DNO_3S$ [M + H]$^+$: 321.1378, found 321.1372.

4. General Procedure for the Co-catalyzed (3+2) cycloaddition of cyclopropene-tethered alkynes 1 (exemplified for 1a).

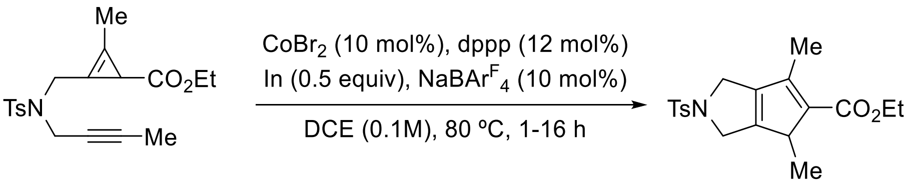

1a **2a**

CoBr$_2$ (2.20 mg, 0.01 mmol), dppp (4.9 mg, 0.012 mmol), In (5.7 mg, 0.05 mmol) and NaBAr$^F_4$ (8.9 mg, 0.01 mmol) were successively added to a dried Schlenk tube under Argon. Then, **1a** (36.0 mg, 0.10 mmol) in 1,2-DCE (1 mL) was added under Ar atmosphere and the resulting mixture was stirred at 80 °C until complete consumption of starting material was indicated by TLC (typically 1-16 h). The mixture was filtered through a

short pad of Florisil® and concentrated under vacuum. The resulting crude was purified by flash column chromatography using silica gel (9:1 hexane/EtOAc), to afford 32.0 mg of ethyl 4,6-dimethyl-2-tosyl-1,2,3,4-tetrahydrocyclopenta[*c*]pyrrole-5-carboxylate **2a**, as a white solid (90% yield). R_f = 0.51 (7:3 hexane/ EtOAc). **^{1}H NMR (300 MHz, $CDCl_3$):** δ 7.75 (m, 2H), 7.33 (m, 2H), 4.38 – 4.10 (m, 6H), 3.28 (m, 1H), 2.43 (s, 3H), 2.22 (d, *J* = 2.1 Hz, 3H), 1.31 (t, *J* = 7.1 Hz, 3H), 1.20 (d, *J* = 7.6 Hz, 3H). **^{13}C NMR (75 MHz, $CDCl_3$):** δ 164.3 (CO), 152.8 (C), 148.3 (C), 144.0 (C), 143.6 (C), 138.4 (C), 134.7 (C), 129.8 (2CH), 127.3 (2CH), 59.6 (CH_2), 51.7 (CH_2), 50.1 (CH_2), 43.8 (CH), 21.5 (CH_3), 14.4 (CH_3), 14.3 (CH_3), 14.2 (CH_3). **HRMS** (APCI-FIA-TOF): *m/z* calculated for $C_{19}H_{25}NO_4S$ $[M + H]^+$: 362.1448, found 362.1451.

Diethyl-4,6,8,9-tetramethyl-2,11-ditosyl-2,3-dihydro-8*H*-5,8b-methano-5a,8a-(methanoiminomethano)cyclopenta[*e*]isoindole-5,7(1*H*)-dicarboxylate (3a)

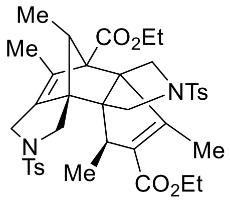

Prepared following the abovementioned general procedure (reaction time extended 96 h), from alkynyl-tethered cyclopropane **1a** (50.0 mg, 0.14 mmol). **3a** (36.0 mg, 72% yield, white solid) was obtained after purification by flash chromatography on silica gel (8:2 → 7:3 hexane/EtOAc). R_f = 0.29 (7:3 hexane/EtOAc). **^{1}H NMR (300 MHz, $CDCl_3$):** δ 7.75 – 7.64 (m, 4H), 7.36 (m, 4H), 4.16 (m, 4H), 3.96 (dd, *J* = 14.5, 1.7 Hz, 1H), 3.71 (dd, *J* = 14.6, 2.3 Hz, 1H), 3.56 (d, *J* = 10.6 Hz, 2H), 3.23 (d, *J* = 10.9 Hz, 2H), 2.98 (d, *J* = 10.8 Hz, 1H), 2.45 (m, 7H), 2.26 (q, *J* = 6.2 Hz, 1H), 2.10 (d, *J* = 7.0 Hz, 1H), 1.93 (d, *J* = 1.9 Hz, 3H), 1.57 (s, 3H), 1.27 (m, 6H), 0.74 (d, *J* = 7.0 Hz, 3H), 0.66 (d, *J* = 6.2 Hz, 3H). **^{13}C NMR (75 MHz, $CDCl_3$):** δ 170.1 (CO), 164.9 (CO), 149.0 (C), 143.9 (C), 140.2 (C), 137.0 (C), 133.1 (C), 131.9 (C), 130.4 (C), 129.8 (2CH), 129.7 (2CH), 128.0 (2CH), 127.5 (2CH), 78.5 (C), 72.8 (C), 66.8 (C), 64.8 (C), 60.8 (CH_2), 59.9 (CH_2), 58.0 (CH), 53.8 (CH_2), 53.1 (CH_2), 47.5 (CH_2), 46.1 (CH_2), 44.0 (CH), 21.6 (CH_3), 21.5 (CH_3), 16.6 (CH_3), 14.3 (CH_3), 14.2 ($2CH_3$), 14.1 (CH_3), 9.0 (CH_3). **HRMS** (APCI-FIA-TOF): *m/z* calculated for $C_{38}H_{47}N_2O_8S_2$ $[M + H]^+$: 723.2768, found 723.2795.

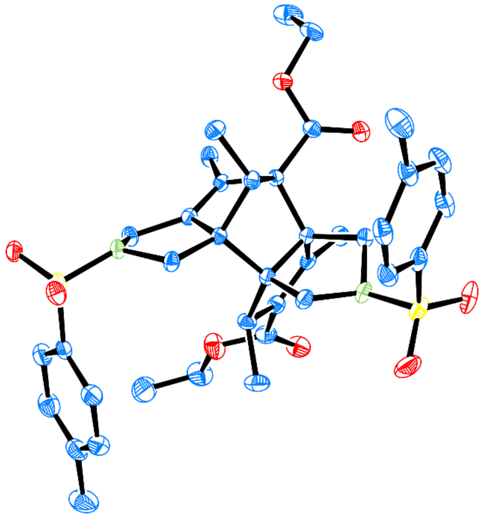

Figure S1. X-ray crystallographic analysis of **3a** (CCDC 2348137)

Ethyl 6-methyl-2-tosyl-4-(triisopropylsilyl)-1,2,3,4-tetrahydrocyclopenta[*c*]pyrrole-5-carboxylate (2b)

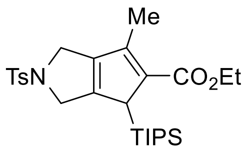

Prepared following the general procedure from alkynyl-tethered cyclopropane **1b** (50 mg, 0.1 mmol). Cycloadduct **2b** (49.0 mg, 99% yield, white solid) was obtained after purification by flash chromatography on silica gel (95:5 hexane/EtOAc). R_f = 0.5 (8:2 hexane/EtOAc). **^{1}H NMR (300 MHz, CD_2Cl_2):** δ 7.76 (m, 2H), 7.38 (m, 2H), 4.57 – 4.23 (m, 6H), 4.10 (m, 1H), 3.88 (s, 1H), 2.45 (s, 3H), 2.20 (s, 3H), 1.31 (t, J = 7.1 Hz, 3H), 1.15 – 0.86 (m, 21H). **^{13}C NMR (75 MHz, CD_2Cl_2):** δ 164.87 (CO), 147.94 (C), 144.50 (C), 143.81 (C), 143.79 (C), 136.56 (C), 134.03 (C), 129.81 (2CH), 127.36 (2CH), 59.64 (CH_2), 53.89 (CH_2), 49.97 (CH_2), 43.41 (CH), 21.17 (CH_3), 18.46 ($3CH_3$), 18.21 ($3CH_3$), 14.11 (CH_3), 13.80 (CH_3), 12.64 (3CH). **HRMS** (APCI-FIA-TOF): *m/z* calculated for $C_{27}H_{42}NO_4SSi$ $[M + H]^+$: 504.2598, found 504.2602.

Ethyl 4-(*tert*-butyl)-6-methyl-2-tosyl-1,2,3,4-tetrahydrocyclopenta[*c*]pyrrole-5-carboxylate (2c)

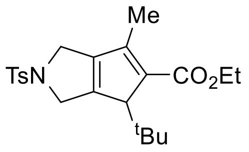

Prepared following the general procedure, from alkynyl-tethered cyclopropane **1c** (30.0 mg, 0.074 mmol). Cycloadduct **2c** (29.4 mg, 98% yield, colorless oil) was obtained after purification by flash chromatography on silica gel (95:5 $\rightarrow$ 90:10 hexane/EtOAc). R_f = 0.37 (8:2 hexane/EtOAc). **^{1}H NMR (300 MHz, $CDCl_3$):** δ 7.74 (m, 2H), 7.33 (m, 2H), 4.36 (t, J = 4.4 Hz, 2H), 4.31 – 4.05 (m, 4H), 3.28 (s, 1H), 2.42 (s, 3H), 2.07 (d, J = 2.0 Hz, 3H), 1.29 (t, J = 7.2 Hz, 3H), 0.86 (s, 9H). **^{13}C NMR (75 MHz, $CDCl_3$):** δ 165.9 (CO), 148.4 (C), 145.8 (C), 145.0 (C), 143.6 (C), 137.2 (C), 134.5 (C), 129.8 (2CH), 127.3 (2CH), 60.4 (CH_3), 60.0 (CH_2), 54.3 (CH_2), 49.6 (CH_2), 34.1 (C), 28.6 ($3CH_3$), 21.5 (CH_3), 14.2 (CH_3), 13.5 (CH_3). **HRMS** (APCI-FIA-TOF): *m/z* calculated for $C_{22}H_{30}NO_4S$ $[M + H]^+$: 404.1890, found 404.1897.

Ethyl 6-methyl-2-tosyl-1,2,3,4-tetrahydrocyclopenta[*c*]pyrrole-5-carboxylate (2d)

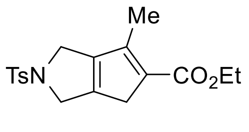

Prepared following the general procedure, from alkynyl-tethered cyclopropane **1d** (42.0 mg, 0.1 mmol). Cycloadduct **2d** (25.0 mg, 73% yield, white solid) was obtained after purification by flash chromatography on silica gel (90:10 $\rightarrow$ 85:15 hexane/EtOAc). R_f = 0.48 (7:3 hexane/EtOAc). **^{1}H NMR (300 MHz, CD_2Cl_2):** δ 7.76 (m, 2H), 7.39 (m, 2H), 4.35 (m, 2H), 4.28 (m, 2H), 4.21 (q, J = 7.0 Hz, 2H), 3.20 (m, 2H), 2.46 (s, 3H), 2.27 (s, 3H), 1.31 (t, J = 7.2 Hz, 3H). **^{13}C NMR (75 MHz, CD_2Cl_2):** δ 164.33 (CO), 148.86 (C), 146.93 (C), 146.11 (C), 143.75 (C), 134.45 (C), 132.69 (C), 129.84 (2CH), 127.31 (2CH), 59.59 (CH_2), 52.81 (CH_2), 50.14 (CH_2), 37.27 (CH_2), 21.20 (CH_3), 14.14 (CH_3), 13.80 (CH_3). **HRMS** (APCI-FIA-TOF): *m/z* calculated for $C_{18}H_{22}NO_4S$ $[M + H]^+$: 348.1264, found 348.1279.

5-Ethyl 2,2-dimethyl 4,6-dimethyl-3,4-dihydropentalene-2,2,5(1*H*)-tricarboxylate (2e)

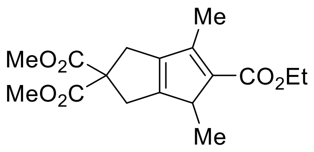

Prepared following the general procedure, from alkynyl-tethered cyclopropane **1e** (32.0 mg, 0.1 mmol). Cycloadduct **2e** (31.7 mg, 99% yield, colorless oil) was obtained after purification by flash chromatography on silica gel (9:1

hexane/Et_2O). R_f = 0.37 (8:2 hexane/ Et_2O). **^{1}H NMR (300 MHz, $CDCl_3$):** δ 4.22 (m, 2H), 3.76 (m, 6H), 3.18 (m, 5H), 2.26 (d, *J* = 2.0 Hz, 3H), 1.31 (t, *J* = 7.1 Hz, 3H), 1.22 (d, *J* = 7.6 Hz, 3H). **^{13}C NMR (75 MHz, $CDCl_3$):** δ 172.2 (CO), 172.1 (CO), 164.7 (CO), 157.0 (C), 150.7 (C), 146.2 (C), 137.6 (C), 63.8 (C), 59.3 (CH_2), 52.9 (CH_3), 43.8 (CH), 37.3 (CH_2), 35.5 (CH_2), 14.5 (CH_3), 14.3 (CH_3), 14.2 (CH_3). **HRMS** (APCI-FIA-TOF): *m/z* calculated for $C_{17}H_{21}O_6$ $[M + H]^+$: 321.1333, found 321.1332.

Diethyl-4,6,8,9-tetramethyl-1*H*,8*H*-5,8b-methano-5a,8a-(methanooxymethano)indeno[4,5-*c*]furan-5,7(3*H*)-dicarboxylate (3f)

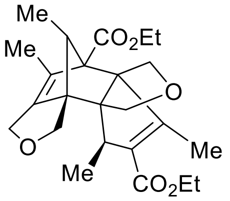

Prepared following the general procedure, from alkynyl-tethered cyclopropane **1f** (50.0 mg, 0.24 mmol). Cycloadduct **3f** (36.0 mg, 73% yield, white colorless oil) was obtained after purification by flash chromatography on silica gel (100:10 → 100:15 → 100:20 hexane/EtOAc). R_f = 0.25 (8:2 hexane/EtOAc). **^{1}H NMR (300 MHz, $CDCl_3$):** δ 4.22 (m, 10H), 3.98 (d, *J* = 10.2 Hz, 1H), 3.77 (d, *J* = 9.6 Hz, 1H), 3.69 (d, *J* = 9.8 Hz, 1H), 3.50 (d, *J* = 9.9 Hz, 1H), 3.07 (q, *J* = 6.2 Hz, 1H), 2.74 (m, 1H), 2.00 (s, 3H), 1.66 (s, 3H), 1.38 – 1.22 (m, 6H), 1.13 (d, *J* = 7.0 Hz, 3H), 0.97 (d, *J* = 6.2 Hz, 3H). **^{13}C NMR (75 MHz, $CDCl_3$):** δ 170.8 (CO), 165.4 (CO), 150.2 (C), 145.9 (C), 136.8 (C), 127.6 (C), 82.2 (C), 74.1 (C), 73.8 (CH_2), 73.1 (CH_2), 69.4 (C), 67.6 (C), 66.3 (CH_2), 65.5 (CH_2), 60.6 (CH_2) 59.8 (CH_2), 57.5 (CH), 43.8 (CH_3), 17.2 (CH_3), 14.4 (CH_3), 14.3 (CH_3), 14.2 (CH_3), 9.5 (CH_3). **HRMS** (APCI-FIA-TOF): *m/z* calculated for $C_{24}H_{33}O_6$ $[M + H]^+$: 417.2272, found 417.2272.

Ethyl 6-methyl-4-(triisopropylsilyl)-3,4-dihydro-1H-cyclopenta[*c*]furan-5-carboxylate (2g)

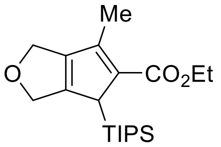

Prepared following the general procedure, from alkynyl-tethered cyclopropane **1g** (51.0 mg, 0.14 mmol). Cycloadduct **2g** (50.0 mg, 99% yield, colorless oil) were obtained after purification by flash chromatography on silica gel (100:5 hexane/EtOAc). R_f = 0.73 (8:2 hexane/EtOAc). **^{1}H NMR (500 MHz, C_6D_6):** δ 4.85 (m, 2H), 4.66 (m, 2H), 4.23 (m, 1H), 3.99 (m, 1H), 3.86 (m, 1H), 2.18 (s, 3H), 1.07 (m, 21H). **^{13}C NMR (125 MHz, C_6D_6):** δ 165.1 (CO), 152.5 (C), 148.8 (C), 144.1 (C), 137.0 (C), 73.3 (CH_2), 69.2 (CH_2), 59.5 (CH_2), 42.6 (CH), 18.9 ($3CH_3$), 18.8 ($3CH_3$), 14.6 (CH_3), 14.5 (CH_3), 12.9 (3CH). **HRMS** (APCI-FIA-TOF): *m/z* calculated for $C_{20}H_{35}O_3Si$ $[M + H]^+$: 351.2350, found 351.2354.

Ethyl 4'-methyl-2'-tosyl-3',6'-dihydro-2'*H*-spiro[cyclohexane-1,1'-cyclopenta[c]pyrrole]-5'-carboxylate (2h)

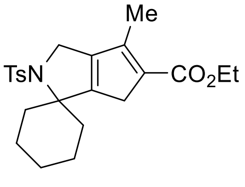

Prepared following the general procedure, from alkynyl-tethered cyclopropane **1h** (55 mg, 0.13 mmol). Cycloadduct **2h** (46 mg, 81% yield, white solid) was obtained after purification by flash chromatography on silica gel (95:5 → 90:10 hexane/EtOAc). R_f = 0.5 (7:3 hexane/EtOAc). **^{1}H NMR (300 MHz, $CDCl_3$):** δ 7.79 (m, 2H), 7.29 (m, 2H), 4.23 (m, 4H), 3.47 (m, 2H), 2.65 (m, 2H), 2.42 (s, 3H), 2.26 (s, 3H), 1.75 (m, 3H), 1.57 – 1.37 (m, 3H), 1.32 (t, *J* = 7.1

Hz, 3H). **^{13}C NMR (75 MHz, $CDCl_3$):** δ 164.6 (CO), 154.0 (C), 148.8 (C), 143.1 (C), 142.8 (C), 138.6 (C), 131.7 (C), 129.4 (CH), 127.2 (CH), 75.7 (C), 59.7 (CH_2), 49.6 (CH_2), 41.2 (CH_2), 36.8 (CH_2), 24.5 (CH_2), 24.4 (CH_2), 21.4 (CH_3), 14.4 (CH_3), 13.9 (CH_3). **HRMS** (APCI-FIA-TOF): *m/z* calculated for $C_{23}H_{30}NO_4S$ $[M + H]^+$: 416.1890, found 416.1899.

5-Ethyl 2,2-dimethyl 4-(triisopropylsilyl)-3,4-dihydropentalene-2,2,5(1*H*)-tricarboxylate (2i)

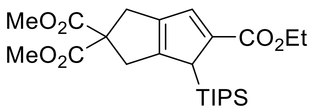

Prepared following the general procedure, from alkynyl-tethered cyclopropane **1i** (45 mg, 0.1 mmol). Cycloadduct **2i** (36.0 mg, 80% yield, colorless oil) was obtained after purification by flash chromatography on silica gel (9:1 hexane/EtOAc). R_f = 0.36 (8:2 hexane/EtOAc). **^{1}H NMR (300 MHz, $CDCl_3$):** δ 7.23 (s, 1H), 4.21 (m, 2H), 3.84 (s, 1H), 3.76 (s, 1H), 3.75 (s, 3H), 3.40 (s, 2H), 3.26 – 3.17 (m, 2H), 1.33 – 1.18 (m, 6H), 1.06 (m, 18H). **^{13}C NMR (75 MHz, $CDCl_3$):** δ 172.2 (CO), 172.1 (CO), 164.6 (CO), 155.3 (C), 144.4 (C), 143.4 (C), 135.9 (CH), 64.6 (C), 59.7 (CH_2), 53.0 (CH_3), 52.9 (CH_3), 43.8 (CH), 40.0 (CH_2), 36.0 (CH_2), 18.9 ($2CH_3$), 18.7 ($2CH_3$), 17.7 (CH), 14.4 (CH), 12.5 ($2CH_3$). **HRMS** (APCI-FIA-TOF): *m/z* calculated for $C_{24}H_{39}O_6Si$ $[M + H]^+$: 451.2510, found 451.2526.

Ethyl 1,4,5,6-tetrahydropentalene-2-carboxylate (2j)

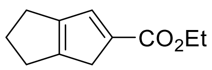

Prepared following the general procedure, from alkynyl-tethered cyclopropane **1j** (18 mg, 0.1 mmol). Cycloadduct **2j** (13 mg, 71% yield, colorless oil) was obtained after purification by flash chromatography on silica gel (100:5 hexane/Et_2O). R_f = 0.47 (9:1 hexane/Et_2O). **^{1}H NMR (500 MHz, $CDCl_3$):** δ 7.33 (s, 1H), 4.23 (q, *J* = 7.1, 2H), 3.15 (s, 2H), 2.55 (m, 2H), 2.49 (m, 2H), 2.34 (q, *J* = 7.3 Hz, 2H), 1.32 (t, *J* = 7.3, 3H). **^{13}C NMR (125 MHz, $CDCl_3$):** δ 164.5 (CO), 157.5 (C), 149.3 (C), 140.1 (CH), 139.3 (C), 59.7 (CH_2), 37.4 (CH_2), 29.7 (CH_2), 28.5 (CH_2), 27.5 (CH_2), 14.4 (CH_3). **HRMS** (APCI-FIA-TOF): *m/z* calculated for $C_{11}H_{15}O_2$ $[M + H]^+$: 179.1067, found 179.1059.

Ethyl 2,2-diphenyl-1,2,3,4-tetrahydrocyclopenta[*c*]silole-5-carboxylate (2k)

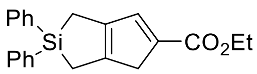

Prepared following the general procedure, from alkynyl-tethered cyclopropane **1k** (100 mg, 0.289 mmol). Cycloadduct **2k** (37.0 mg, 37% yield, colorless oil) was obtained after purification by flash chromatography on silica gel (100:3 $\rightarrow$ 100:6 hexane/EtOAc). R_f = 0.5 (8:2 hexane/EtOAc). **^{1}H NMR (500 MHz, $CDCl_3$):** δ 7.57 (m, 4H), 7.40 (m, 6H), 4.24 (q, *J* = 7.1 Hz, 2H), 3.25 (q, *J* = 2.1 Hz, 2H), 2.15 (s, 2H), 2.09 (t, *J* = 2.0 Hz, 2H), 1.32 (t, *J* = 7.1 Hz, 3H). **^{13}C NMR (125 MHz, $CDCl_3$):** δ 164.4 (CO), 153.7 (C), 144.9 (C), 143.7 (CH), 137.5 (C), 135.4 (2C), 134.5 (4CH), 129.8 (2CH), 128.1 (4CH), 59.8 (CH_2), 41.8 (CH_2), 15.9 (CH_2), 14.4 (CH_3), 13.9 (CH_2). **HRMS** (APCI-FIA-TOF): *m/z* calculated for $C_{22}H_{23}O_2Si$ $[M + H]^+$: 347.1462, found 347.1464.

Ethyl 1,3-dimethyl-4,9-dihydro-1*H*-cyclopenta[*b*]naphthalene-2-carboxylate (2l)

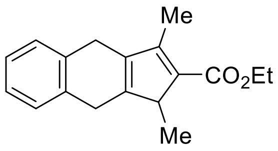

Prepared following the general procedure, from alkynyl-tethered cyclopropane **1l** (50.0 mg, 0.19 mmol). Cycloadduct **2l** (30.0 mg, 60% yield, white solid) was obtained after purification by flash chromatography on silica gel (100:2 → 100:3 → 100:4 hexane/Et_2O). R_f = 0.4 (8:2 hexane/ Et_2O). **^{1}H NMR (300 MHz, $CDCl_3$):** δ 7.25 (m, 4H), 4.29 (m, 2H), 3.70 (m, 4H), 3.29 (m, 1H), 2.38 (s, 3H), 1.37 (t, J = 7.1, 3H), 1.31 (d, J = 7.5, 3H). **^{13}C NMR (75 MHz, $CDCl_3$):** δ 165.2 (CO), 154.0 (C), 148.5 (C), 136.0 (C), 133.6 (C), 133.5 (C), 133.3 (C), 129.3 (CH), 129.1 (CH), 126.2 (CH), 126.1 (CH), 59.3 (CH_2), 48.1 (CH), 29.1 (CH_2), 27.8 (CH_2), 14.5 (CH_3), 14.5 (CH_3), 13.3 (CH_3). **HRMS** (APCI-FIA-TOF): *m/z* calculated for $C_{18}H_{21}O_2$ $[M + H]^+$: 269.1536, found 269.1539.

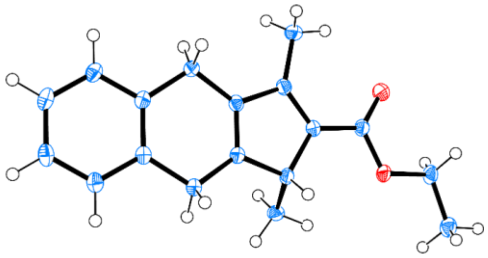

Figure S2. X-ray crystallographic analysis of **2l** (CCDC 2348138)

Ethyl 4,6-diphenyl-2-tosyl-1,2,3,5-tetrahydrocyclopenta[*c*]pyrrole-5-carboxylate (2m')

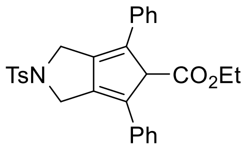

Prepared following the general procedure, from alkynyl-tethered cyclopropane **1m** (50 mg, 0.1 mmol). Cycloadduct **2m'** (30.0 mg, 60% yield, white solid) was obtained after purification by flash chromatography on silica gel (9:1 hexane/EtOAc). R_f = 0.5 (7:3 hexane/EtOAc). **^{1}H NMR (500 MHz, $CDCl_3$):** δ 7.83 (d, J = 8.0 Hz, 2H), 7.34 – 7.23 (m, 10H), 7.36 (m, 2H), 5.22 (s, 1H), 4.52 (d, J = 15.0 Hz, 2H), 4.39 (d, J = 14.9 Hz, 2H), 4.02 (q, J = 7.1 Hz, 2H), 2.44 (s, 3H), 0.99 (t, J = 7.1 Hz, 3H). **^{13}C NMR (125 MHz, $CDCl_3$):** δ 169.9 (CO), 143.4 (C), 144.0 (C), 133.8 (C), 133.4 (C), 133.2 (C), 130.0 (2CH), 128.9 (4CH), 127.5 (2CH), 127.3 (2CH), 126.2 (4CH), 63.7 (CH) 61.4 (CH_2), 47.8 (CH_2), 21.5 (CH_3), 13.9 (CH_3). **HRMS** (APCI-FIA-TOF): *m/z* calculated for $C_{29}H_{28}NO_4S$ $[M + H]^+$: 486.1734, found 486.1731.

Ethyl 2-tosyl-4,6-bis(4-(trifluoromethyl)phenyl)-1,2,3,5-tetrahydrocyclopenta[*c*]pyrrole-5-carboxylate (2n')

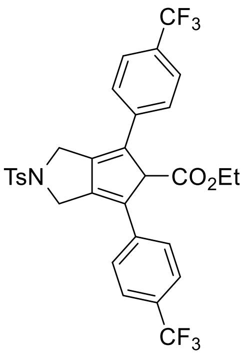

Prepared following the general procedure, from alkynyl-tethered cyclopropane **1n** (50.0 mg, 0.10 mmol). Cycloadduct **2n'** (46.0 mg, 92% yield, white solid) was obtained after purification by flash chromatography on silica gel (CH_2Cl_2). R_f = 0.55 (CH_2Cl_2). **^{1}H NMR (500 MHz, CDCl$_3$):** 7.84 (m, 2H), 7.64 (m, 4H), 7.45 (m, 4H), 7.39 (m, 2H), 5.25 (s, 1H), 4.54 (d, *J* = 15.2 Hz, 2H), 4.41 (d, *J* = 15.0 Hz, 2H), 4.03 (q, *J* = 7.1 Hz, 2H), 2.45 (s, 3H), 1.01 (t, *J* = 7.1 Hz, 3H). **^{13}C NMR (125 MHz, CDCl$_3$):** δ 169.02 (C=O), 146.7 (C), 144.3 (C), 136.3 (C), 133.61 (C), 133.0 (C), 130.1 (2CH), 129.3 (q, *J* = 32.8 Hz, 2C), 127.5 (2CH), 126.4 (4CH), 126.0 (q, *J* = 3.8 Hz, 4CH), 123.9 (q, *J* = 272.0 Hz, 2CF$_3$), 63.9 (CH), 61.9 (CH$_2$), 47.7 (CH$_2$), 21.5 (CH$_3$), 13.9 (CH$_3$). **^{19}F NMR (471 MHz, CDCl$_3$):** δ -62.7 (s, 6F, 2CF$_3$). **HRMS** (APCI-FIA-TOF): *m/z* calculated for $C_{31}H_{26}F_6NO_4S$ $[M + H]^+$: 622.1481, found 622.1491.

Ethyl 4-(4-methoxyphenyl)-2-tosyl-6-(4-(trifluoromethyl)phenyl)-1,2,3,5-tetrahydrocyclopenta[*c*]pyrrole-5-carboxylate (2o')

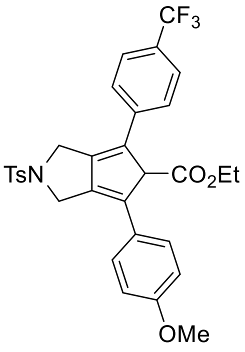

Prepared following the general procedure, from alkynyl-tethered cyclopropane **1o** (100 mg, 0.17 mmol). Cycloadduct **2o'** (39 mg, 39% yield, white solid) was obtained after purification by flash chromatography on silica gel (90:10 $\rightarrow$ 85:15 hexane/EtOAc). R_f = 0.4 (7:3 hexane/EtOAc). **^{1}H NMR (500 MHz, CDCl$_3$):** 7.84 (m, 2H), 7.50 (m, 2H), 7.39 (m, 2H), 7.28 (m, 2H), 7.01 (m, 2H), 6.73 (m, 2H), 5.10 (t, *J* = 2.8 Hz, 1H), 4.67 (dd, *J* = 15.4, 2.2 Hz, 1H), 4.53 (dd, *J* = 17.0, 3.0 Hz, 1H), 4.40 (m, 2H), 4.19 – 4.03 (m, 1H), 3.73 (s, 3H), 2.46 (s, 3H), 1.22 (t, *J* = 7.1 Hz, 3H). **^{13}C NMR (125 MHz, CDCl$_3$):** δ 162.5 (CO), 158.7 (C), 156.3 (C), 144.1 (C), 143.5 (C), 143.0 (C), 136.3 (C), 133.8 (C), 131.3 (C), 130.1 (2CH), 129.5 (q, *J* = 32.7 Hz, C), 129.1 (2CH), 127.6 (2CH), 127.4 (2CH), 127.3 (C), 125.7 (q, *J* = 3.7 Hz, 2CH), 123.8 (q, *J* = 272.0 Hz, CF$_3$), 114.1 (2CH), 63.0 (CH), 60.3 (CH$_2$), 55.1 (CH$_3$), 48.3 (CH$_2$), 48.2 (CH$_2$), 21.6 (CH$_3$), 14.23 (CH$_3$). **^{19}F NMR (471 MHz, CDCl$_3$):** δ -62.8 (s, 3F, CF$_3$). **HRMS** (APCI-FIA-TOF): *m/z* calculated for $C_{31}H_{29}F_3NO_5S$ $[M + H]^+$: 584.1713, found 584.1728.

Ethyl 2-tosyl-6-(4-(trifluoromethyl)phenyl)-1,2,3,4-tetrahydrocyclopenta[*c*]pyrrole-5-carboxylate (2p'')

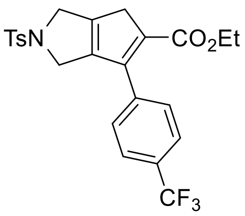

Prepared following the general procedure, from alkynyl-tethered cyclopropane **1p** (55 mg, 0.1 mmol). Cycloadduct **2p''** (36 mg, 65% yield, pale yellow colored solid) was obtained after purification by flash chromatography on silica gel (100:0 $\rightarrow$ 100:1 CH_2Cl_2/EtOAc). R_f = 0.4 (7:3 hexane/EtOAc). **^{1}H NMR (500 MHz, CDCl$_3$):** 7.74 (m, 2H), 7.65 (m, 2H), 7.44 (m, 2H), 7.33 (m, 2H), 4.42 (s, 2H), 4.22 (s, 2H), 4.12 (m, 2H), 3.44 (s, 2H), 2.43 (s, 3H), 1.15 (m, 3H). **^{13}C NMR (125 MHz, CDCl$_3$):** δ 163.4 (CO), 148.6 (C), 147.1 (C), 145.6 (C), 143.7

(C), 137.5 (C),134.4 (C), 134.3 (C), 130.6 (q, *J* = 32.6 Hz, C), 129.9 (2CH), 128.8 (2CH), 127.4 (2CH), 125.0 (q, *J* = 3.7 Hz, 2CH), 124.0 (q, *J* = 272.0 Hz, CF_3), 60.3 (CH_2), 52.6 (CH_2), 50.5 (CH_2), 38.4 (CH_2), 21.5 (CH_3), 14.0 (CH_3). **^{19}F NMR (471 MHz, CDCl$_3$):** δ -62.8 (s, 3F, CF_3). **HRMS** (APCI-FIA-TOF): *m/z* calculated for $C_{24}H_{23}F_3NO_4S$ [M + H]$^+$: 478.1294, found 478.1293.

Ethyl 6-(perfluorophenyl)-2-tosyl-1,2,3,4-tetrahydrocyclopenta[*c*]pyrrole-5-carboxylate (2q'')

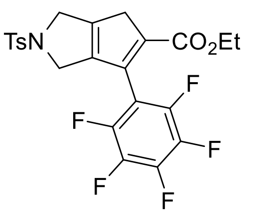

Prepared following the general procedure, from alkynyl-tethered cyclopropane **1q** (50 mg, 0.10 mmol). Cycloadduct **2q''** (35.0 mg, 70% yield, white solid) was obtained after purification by flash chromatography on silica gel (90:10 hexane/EtOAc). R$_f$ = 0.26 (8:2 hexane/EtOAc). **^{1}H NMR (500 MHz, CDCl$_3$):** 7.74 (m, 2H), 7.34 (m, 2H), 4.42 (t, *J* = 4.4 Hz, 2H), 4.22 (s, 2H), 4.14 (q, *J* = 7.1 Hz, 2H), 3.47 (s, 2H), 2.43 (s, 3H), 1.18 (t, *J* = 7.1 Hz, 3H). **^{13}C NMR (125 MHz, CDCl$_3$):** δ 162.4 (CO), 147.7 (C), 144.8 (C), 143.9 (m, C), 143.8 (C), 141.3 (m, C), 139.9 (C), 137.3 (m, C), 134.2 (C), 133.5 (d, *J* = 2.0 Hz, C), 129.9 (2CH), 127.3 (2CH), 109.1 (td, *J* = 18.3, 4.1 Hz, C), 60.6 (CH_2), 52.5 (CH_2), 50.2 (CH_2), 38.4 (CH_2), 21.5 (CH_3), 13.9 (CH_3). **^{19}F NMR (471 MHz, CDCl$_3$):** δ -138.4 (m, 2F), -153.0 (t, *J* = 20.8 Hz, F), -161.8 (m, 2F). **HRMS** (APCI-FIA-TOF): *m/z* calculated for $C_{23}H_{19}F_5NO_4S$ [M + H]$^+$: 500.0949, found 500.0954.

Ethyl 6-(thiophen-2-yl)-2-tosyl-1,2,3,4-tetrahydrocyclopenta[*c*]pyrrole-5-carboxylate (2r'')

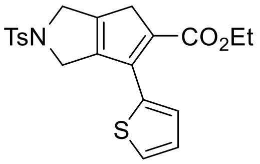

Prepared following the general procedure, from alkynyl-tethered cyclopropane **1r** (42.0 mg, 0.10 mmol). Cycloadduct **2r''** (26.0 mg, 61% yield, white solid) was obtained after purification by flash chromatography on silica gel (95:5 → 90:10 hexane/EtOAc). R$_f$ = 0.4 (7:3 hexane/EtOAc). **^{1}H NMR (300 MHz, CDCl$_3$):** 7.77 (m, 2H), 7.63 (d, *J* = 3.7 Hz, 1H), 7.46 (dd, *J* = 5.0, 1.2 Hz, 1H), 7.33 (m, 2H), 7.10 (ddd, *J* = 4.8, 3.7, 0.9 Hz, 1H), 4.53 (m, 2H), 4.39 (t, *J* = 4.3 Hz, 2H), 4.24 (q, *J* = 7.1 Hz, 2H), 3.42 (s, 2H), 2.42 (s, 3H), 1.30 (t, *J* = 7.1 Hz, 3H). **^{13}C NMR (75 MHz, CDCl$_3$):** δ 163.9 (CO), 147.0 (C), 145.1 (C), 143.7 (C), 142.0 (C), 135.0 (C), 134.4 (C), 130.9 (CH), 129.9 (2CH), 128.8 (CH), 127.4 (2CH), 127.2 (CH), 60.2 (CH_2), 52.4 (CH_2), 52.1 (CH_2), 38.6 (CH_2), 21.5 (CH_3), 14.3 (CH_3). **HRMS** (APCI-FIA-TOF): *m/z* calculated for $C_{21}H_{22}NO_4S_2$ [M + H]$^+$: 416.0985, found 416.1000.

5-Ethyl 2,2-dimethyl 6-methyl-3,4-dihydropentalene-2,2,5(1*H*)-tricarboxylate (2s'')

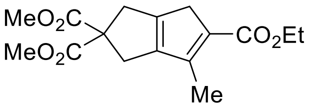

Prepared following the general procedure, from alkynyl-tethered cyclopropane **1s** (110 mg, 0.35 mmol). Cycloadduct **2s''** (96.0 mg, 87% yield, colorless oil) was obtained after purification by flash chromatography on silica gel (85:15 hexane/Et_2O). R$_f$ = 0.32 (85:15 hexane/ Et_2O). **^{1}H NMR (500 MHz, CDCl$_3$):** δ 4.21 (q, *J* = 7.1 Hz, 2H), 3.76 (s, 6H), 3.25 (s, 2H), 3.15 (m, 4H), 2.28 (t, *J* = 2.3 Hz, 3H), 1.30 (t, *J* = 7.1 Hz, 3H). **^{13}C NMR (125 MHz, CDCl$_3$):** δ 172.2 (CO), 165.0 (CO), 151.4 (C), 150.1 (C), 149.3 (C), 131.8 (C), 64.1 (C), 59.4 (CH_2), 53.0 ($2CH_3$), 38.6 (CH_2), 37.8

(CH_2), 35.6 (CH_2), 14.4 (CH_3), 14.2 (CH_3). **HRMS** (APCI-FIA-TOF): *m/z* calculated for $C_{16}H_{21}O_6$ [M + H]$^+$: 309.1333, found 309.1338.

5-Ethyl 2,2-dimethyl 4-methyl-6-phenyl-3,4-dihydropentalene-2,2,5(1*H*)-tricarboxylate (2t'')

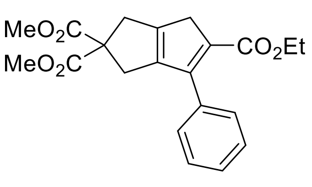

Prepared following the general procedure, from alkynyl-tethered cyclopropane **1t** (37.0 mg, 0.1 mmol). Cycloadduct **2t''** (29.6 mg, 80% yield, colorless oil) was obtained after purification by flash chromatography on silica gel (8:2 hexane/EtOAc). R_f = 0.34 (8:2 hexane/ EtOAc). **^{1}H NMR (300 MHz, $CDCl_3$):** δ 7.39 (m, 5H), 4.11 (q, *J* = 7.2 Hz, 2H), 3.75 (s, 6H), 3.37 (s, 2H), 3.32 (s, 2H), 3.18 (s, 2H), 1.15 (t, *J* = 7.1 Hz, 3H). **^{13}C NMR (75 MHz, $CDCl_3$):** δ 172.0 (C), 164.1 (C), 152.0 (C), 150.6 (C), 148.4 (C), 134.7 (C), 132.4 (C), 131.6 (C), 128.7 (2CH), 128.2 (CH), 127.7 (2CH), 64.1 (C), 59.7 (CH_2), 53.0 (CH_2), 38.8 (CH_2), 38.6 (CH_2), 36.6 (CH_2), 14.1 (CH_3). **HRMS** (APCI-FIA-TOF): calculated for $C_{21}H_{23}O_6$ [M + H]$^+$: 371.1489, found 371.1496.

5-((2*R*,5*R*)-5-Isopropyl-2-methylcyclohexyl) 2,2-dimethyl 4-ethyl-3,4-dihydropentalene-2,2,5(1*H*)-tricarboxylate (2u)

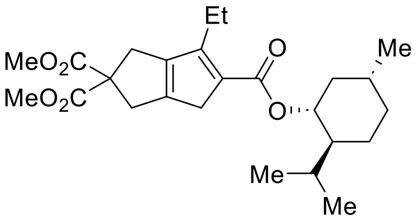

Prepared following the general procedure, from alkynyl-tethered cyclopropane **1u** (43.0 mg, 0.1 mmol). Cycloadduct **2u** (31.0 mg, 71% yield, colorless oil) was obtained after purification by flash chromatography on silica gel (9:1 hexane/EtOAc). R_f = 0.56 (8:2 hexane/EtOAc). **^{1}H NMR (300 MHz, $CDCl_3$):** 4.76 (td, *J* = 10.8, 4.3 Hz, 1H), 3.77 (s, 6H), 3.24 (s, 2H), 3.20 (s, 2H), 3.15 (s, 2H), 2.78 (m, 2H), 2.07 (m, 1H), 1.93 (m, 1H), 1.68 (m, 3H), 1.46 (s, 3H), 1.13 (t, *J* = 7.6 Hz, 3H), 0.99 (m, 1H), 0.91 (m, 7H), 0.78 (d, *J* = 6.9 Hz, 3H). **^{13}C NMR (75 MHz, $CDCl_3$):** δ 172.2 (CO), 164.3 (C), 157.0 (C), 150.6 (C), 148.6 (C), 131.4 (C), 73.1 (CH), 64.4 (C), 53.0 ($2CH_3$), 47.4 (CH), 41.3 (CH_2), 38.5 (CH_2), 37.9 (CH_2), 36.1 (CH_2), 34.4 (CH_2), 31.4 (CH), 26.4 (CH), 23.6 (CH_2), 22.0 (CH_3), 21.6 (CH_2), 20.8 (CH_3), 16.5 (CH_3), 13.0 (CH_3). **HRMS** (APCI-FIA-TOF): *m/z* calculated for $C_{25}H_{37}O_6$ [M + H]$^+$: 433.2585, found 433.2595.

(4-Chlorophenyl)(4,6-dimethyl-2-tosyl-1,2,3,4-tetrahydrocyclopenta[*c*]pyrrol-5-yl)methanone (2v)

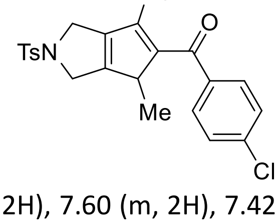

Prepared following the general procedure, from alkynyl-tethered cyclopropane **1v** (52.0 mg, 0.12 mmol). Cycloadduct **2v** (34.0 mg, 72% yield, pale yellow solid) was obtained after purification by flash chromatography on silica gel (95:5 → 90:10 hexane/EtOAc). R_f = 0.27 (8:2 hexane/EtOAc). **^{1}H NMR (500 MHz, $CDCl_3$):** δ 7.77 (m, 2H), 7.60 (m, 2H), 7.42 (m, 2H), 7.35 (m, 2H), 4.34 (m, 2H), 4.30 (m, 1H), 4.22 (m, 1H), 3.65 (m, 1H), 2.45 (s, 3H), 1.78 (d, *J* = 2.1 Hz, 3H), 1.11 (d, *J* = 7.7 Hz, 3H). **^{13}C NMR (125 MHz, $CDCl_3$):** δ 191.6 (CO), 153.5 (C), 148.2

(C), 145.3 (C), 144.3 (C), 143.7 (C), 138.5 (C), 138.4 (C), 134.5 (C), 130.0 (2CH), 129.9 (2CH), 128.8 (2CH), 127.4 (2CH), 51.7 (CH_2), 50.0 (CH_2), 44.8 (CH_3), 21.5 (CH), 15.1 (CH_3), 13.2 (CH_3). **HRMS** (APCI-FIA-TOF): *m/z* calculated for $C_{23}H_{23}ClNO_3S$ $[M + H]^+$: 428.1082, found 428.1068.

4,6-Dimethyl-*N*-phenyl-2-tosyl-1,2,3,4-tetrahydrocyclopenta[*c*]pyrrole-5-carboxamide (2w)

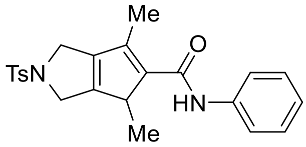

Prepared following the general procedure, from alkynyl-tethered cyclopropane **1w** (33.0 mg, 0.08 mmol). Cycloadduct **2w** (26.0 mg, 78% yield, white solid) was obtained after purification by flash chromatography on silica gel (9:1 → 8:2 hexane/EtOAc). R_f = 0.58 (1:1 hexane/EtOAc). **^{1}H NMR (500 MHz, $CDCl_3$):** δ 7.76 (m, 2H), 7.54 (m, 2H), 7.34 (m, 3H), 7.12 (m, 2H), 4.34 – 4.31 (m, 1H), 4.32 (m, 2H), 3.39 (m, 1H), 2.44 (s, 3H), 2.24 (d, *J* = 2.0 Hz, 3H), 1.24 (d, *J* = 7.6 Hz, 3H). **^{13}C NMR (125 MHz, $CDCl_3$):** δ 162.7 (CO), 150.4 (CO), 144.1 (C), 143.7 (C), 142.9 (C), 142.3 (C), 137.8 (C), 134.5 (C), 129.9 (2CH), 129.1 (2CH), 127.3 (2CH), 124.4 (CH), 119.9 (2CH), 51.7 (CH_2), 50.1 (CH_2), 43.6 (CH_3), 21.5 (CH), 14.0 (CH_3), 13.9 (CH_3). **HRMS** (APCI-FIA-TOF): *m/z* calculated for $C_{23}H_{25}N_2O_3S$ $[M + H]^+$: 409.1580, found 409.1588.

5-(Methoxymethyl)-6-methyl-2-tosyl-1,2,3,4-tetrahydrocyclopenta[*c*]pyrrole (2x)

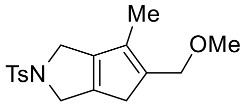

Prepared following the general procedure, from alkynyl-tethered cyclopropane **1x** (21 mg, 0.07 mmol). Cycloadduct **2x** (14.0 mg, 67% yield, white solid) was obtained after purification by flash chromatography on silica gel (85:15 → 80:20 hexane/EtOAc). R_f = 0.32 (7:3 hexane/EtOAc). **^{1}H NMR (300 MHz, $CDCl_3$):** δ 7.74 (m, 2H), 7.32 (m, 2H), 4.30 (m, 2H), 4.23 (m, 2H), 4.10 (s, 2H), 3.29 (s, 3H), 2.89 (s, 2H), 2.43 (s, 3H), 1.86 (s, 3H). **^{13}C NMR (75 MHz, $CDCl_3$):** δ 146.1 (C), 143.3 (C), 140.3 (C), 140.1 (C), 134.7 (C), 134.3 (C), 129.8 (2CH), 127.4 (2CH), 67.7 (CH_2), 57.8 (CH_3), 52.9 (CH_2), 50.5 (CH_2), 37.7 (CH_2), 21.5 (CH_3), 11.6 (CH_3). **HRMS** (APCI-FIA-TOF): *m/z* calculated for $C_{17}H_{22}NO_3S$ $[M + H]^+$: 320.1315, found 320.1300.

(4,6-Dimethyl-2-tosyl-1,2,3,5-tetrahydrocyclopenta[*c*]pyrrol-5-yl) methanol (2y')

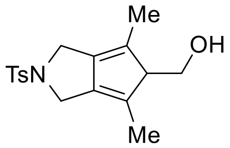

Prepared following the general procedure, from alkynyl-tethered cyclopropane **1y** (32 mg, 0.1 mmol). Cycloadduct **2y'** (13 mg, 41% yield, colorless oil) was obtained after purification by flash chromatography on silica gel (7:3 hexane/EtOAc). R_f = 0.42 (4:6 hexane/EtOAc). **^{1}H NMR (300 MHz, $CDCl_3$):** 7.75 (m, 2H), 7.33 (m, 2H), 4.00 (s, 4H), 3.90 (t, *J* = 4.6 Hz, 2H), 3.10 (m, 1H), 2.43 (s, 3H), 1.85 (s, 6H), 0.84 (t, *J* = 5.6 Hz, OH). **^{13}C NMR (75 MHz, $CDCl_3$):** δ 143.6 (C), 140.4 (C), 133.8 (C), 129.9 (C), 129.7 (2CH), 127.6 (2CH), 66.1 (CH), 60.5 (CH_2), 46.5 ($2CH_2$), 21.5 (CH_3), 12.7 ($2CH_3$). **HRMS** (APCI-FIA-TOF): *m/z* calculated for $C_{17}H_{22}NO_3S$ $[M + H]^+$: 320.1315, found 320.1312.

4,6-Dimethyl-2-tosyl-5-(trifluoromethyl)-1,2,3,5-tetrahydrocyclopenta[*c*]pyrrole (2z')

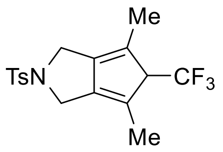

Prepared following the general procedure, from alkynyl-tethered cyclopropane **1z** (100 mg, 0.28 mmol). Cycloadduct **2z'** (72 mg, 72% yield, pale yellow solid) was obtained after purification by flash chromatography on silica gel (95:5 hexane/EtOAc). R_f = 0.44 (8:2 hexane/EtOAc). **^{1}H NMR (500 MHz, $CDCl_3$):** 7.75 (m, 2H), 7.35 (m, 2H), 4.02 (d, *J* = 15.5 Hz, 2H), 3.96 (d, *J* = 15.3 Hz, 2H), 3.62 (m, 1H), 2.44 (s, 3H), 1.89 (s, 6H). **^{13}C NMR (125 MHz, $CDCl_3$):** *δ* 143.8 (C), 143.6 (C), 133.6 (C), 129.8 (2CH), 127.6 (2CH), 125.5 (q, *J* = 280.4 Hz, CF_3), 125.5 (q, *J* = 2.2 Hz, C), 65.2 (q, *J* = 27.1 Hz, CH), 46.3 ($2CH_2$), 21.5 (CH_3), 13.6 (CH_3), 13.6 (CH_3). **^{19}F NMR (471 MHz, $CDCl_3$):** *δ* -65.9 (s, 3F, CF_3). **HRMS** (APCI-FIA-TOF): *m/z* calculated for $C_{17}H_{19}F_3NO_2S$ $[M + H]^+$: 358.1083, found 358.1087.

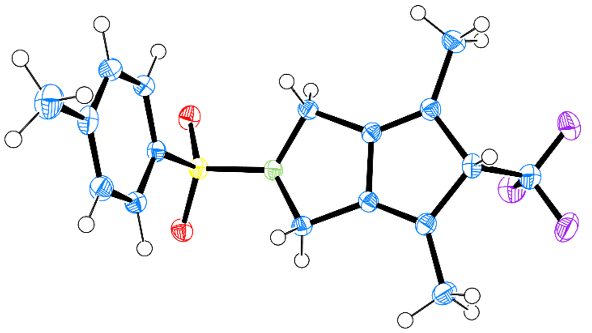

Figure S3. X-ray crystallographic analysis of **2z'** (CCDC 2348141)

2-Tosyl-5-(trifluoromethyl)-4-(4-(trifluoromethyl)phenyl)-1,2,3,5-tetrahydrocyclopenta[*c*]pyrrole (2za')

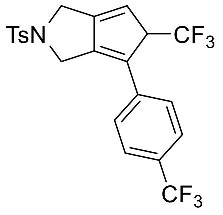

Prepared following the general procedure, from alkynyl-tethered cyclopropane **1za** (42 mg, 0.09 mmol). Cycloadduct **2za'** (40 mg, 95% yield, white solid) was obtained after purification by flash chromatography on silica gel (95:5 hexane/EtOAc). R_f = 0.3 (8:2 hexane/EtOAc). **^{1}H NMR (500 MHz, $CDCl_3$):** 7.78 (m, 2H), 7.63 (m, 2H), 7.35 (m, 4H), 6.02 (s, 1H), 4.45 (dd, *J* = 15.5, 2.3 Hz, 1H), 4.24 – 4.16 (m, 3H), 2.45 (s, 2H), 1.56 (s, 3H). **^{13}C NMR (125 MHz, $CDCl_3$):** *δ* 150.3 (C), 148.0 (C), 144.2 (C), 136.6 (C), 133.4 (C), 130.0 (2CH), 130.7 (C), 129.5 (q, *J* = 32.8 Hz, C), 127.6 (2CH), 127.5 (2CH), 125.6 (q, *J* = 3.7 Hz, 2CH), 124.6 (q, *J* = 279.6 Hz, CF_3), 123.9 (q, *J* = 272.0 Hz, CF_3), 117.5 (q, *J* = 2.8 Hz, CH), 61.4 (q, *J* = 28.6 Hz, CH), 47.6 (CH_2), 46.7 (CH_2), 21.6 (CH_3). **^{19}F NMR (471 MHz, $CDCl_3$):** *δ* -62.7 (s, 3F, CF_3), -65.3 (s, 3F, CF_3). **HRMS** (APCI-FIA-TOF): *m/z* calculated for $C_{22}H_{18}F_6NO_2S$ $[M + H]^+$: 474.0957, found 474.0963.

Methyl 5-phenyl-2-tosyl-2,3-dihydrocyclopenta[*c*]pyrrole-3a(1*H*)-carboxylate (2zb''')

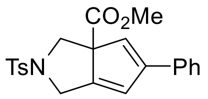

Prepared following the general procedure (extended reaction time to 72 h), from alkynyl-tethered cyclopropane **1zbc** (58.0 mg, 0.15 mmol). Cycloadduct **2zc'''** (24.0 mg, 41% yield, white solid) was obtained after purification by flash chromatography on silica gel (85:15 → 80:20

hexane/EtOAc). R_f = 0.52 (6:4 hexane/EtOAc). **^{1}H NMR (300 MHz, $CDCl_3$):** δ 7.73 (m, 2H), 7.48 (m, 2H), 7.35 (m, 5H), 6.62 (s, 1H), 6.39 (s, 1H), 4.28 (m, 2H), 3.98 (d, J = 12.7 Hz, 1H), 3.56 (s, 3H), 2.73 (d, J = 9.5 Hz, 1H), 2.42 (s, 3H). **^{13}C NMR (75 MHz, $CDCl_3$):** δ 170.1 (CO), 150.6 (C), 150.0 (C), 143.6 (C), 134.3 (C), 133.8 (C), 129.7 (2CH), 128.7 (2CH), 128.7 (CH), 127.5 (2CH), 127.4 (CH), 126.2 (2CH), 124.7 (CH), 70.3 (C), 53.5 (CH_2), 52.9 (CH_3), 45.3 (CH_2), 21.5 (CH_3). **HRMS** (APCI-FIA-TOF): *m/z* calculated for $C_{22}H_{22}NO_4S$ [M + H]$^+$: 396.1264, found 396.1246.

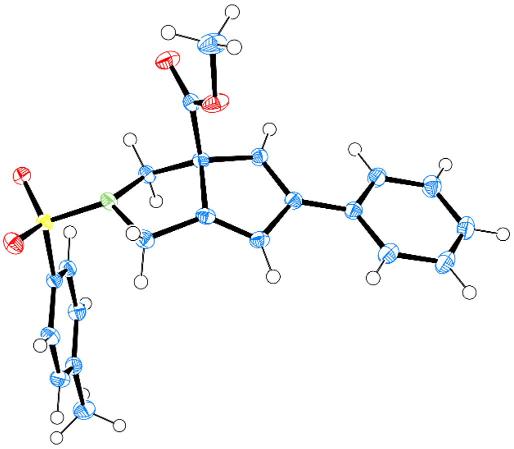

Figure S4. X-ray crystallographic analysis of **2zc'''** (CCDC 2348140)

1,5,5-Trimethyl-4-phenyl-2-tosyl-1,2,3,5-tetrahydrocyclopenta[*c*]pyrrole (2zd'')

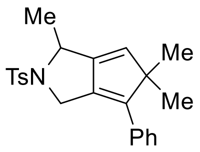

Prepared following the general procedure (extended reaction time to 96 h), from alkynyl-tethered cyclopropane **1zc** (38.0 mg, 0.12 mmol). Cycloadduct **2zc'** (9.9 mg, 26% yield, colorless oil) was obtained after purification by flash chromatography on silica gel (100:2 → 100:5 hexane/EtOAc). R_f = 0.6 (8:2 hexane/EtOAc). **^{1}H NMR (500 MHz, $CDCl_3$):** δ 7.68 (m, 2H), 7.29 (m, 2H), 7.24 – 7.17 (m, 4H), 7.15 (t, J = 7.2 Hz, 1H), 5.72 (s, 1H), 4.42 (d, J = 15.5 Hz, 1H), 4.35 (q, J = 6.4 Hz, 1H), 4.22 (d, J = 15.4 Hz, 1H), 2.33 (s, 3H), 1.48 (s, 3H), 1.27 (s, 3H), 1.19 (s, 3H). **^{13}C NMR (125 MHz, $CDCl_3$):** δ 147.3 (C), 143.4 (C), 142.5 (C), 138.2 (C), 135.4 (CH), 135.0 (C), 134.6 (C), 129.7 (2CH), 128.6 (2CH), 127.5 (2CH), 126.9 (2CH), 126.4 (CH), 60.4 (C), 54.9 (CH), 48.8 (CH_2), 23.0 (CH_3), 22.8 (CH_3), 22.7 (CH_3), 21.5 (CH_3). **HRMS** (APCI-FIA-TOF): *m/z* calculated for $C_{23}H_{26}NO_2S$ [M + H]$^+$: 380.1679, found 380.1682.

Ethyl 4,6-dimethyl-2-tosyl-1,2,3,4-tetrahydrocyclopenta[*c*]pyrrole-5-carboxylate-4-d (*d*-2a)

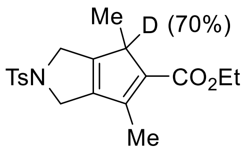

Prepared following the general procedure, from alkynyl-tethered cyclopropane ***d*-1a** (40 mg, 0.11 mmol, 97% D). Cycloadduct ***d*-2a** (39.0 mg, 72% yield, 70% D, colorless oil) was obtained after purification by flash chromatography on silica gel (95:5 → 90:10 hexane/EtOAc). R_f = 0.26 (8:2 hexane/EtOAc). **^{1}H NMR (300 MHz, $CDCl_3$):** δ 7.75 (m, 2H), 7.33 (m, 2H), 4.24 (m, 6H), 2.43 (s, 3H), 2.22 (s, 3H), 1.31 (t, J = 7.1 Hz, 3H), 1.19 (s, 3H). **^{13}C NMR (75 MHz, $CDCl_3$):** δ 164.4 (CO), 152.8 (C), 148.3 (C), 144.1 (C), 143.6 (C), 138.4 (C), 134.7 (C), 129.9 (2CH), 127.3 (2CH), 59.6 (CH_2), 51.7 (CH_2), 50.1

(CH_2), 21.5 (CH_3), 14.4 (CH_3), 14.3 (CH_3), 14.1 (CH_3). **HRMS** (APCI-FIA-TOF): *m/z* calculated for $C_{19}H_{23}DNO_4S$ [M + H]$^+$: 363.1483, found 363.1482.

(4,6-Dimethyl-2-tosyl-1,2,3,5-tetrahydrocyclopenta[*c*]pyrrol-5-yl-5-*d*)methanol (*d*-2y')

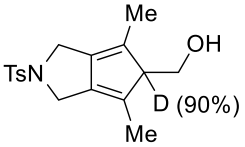

Prepared following the general procedure, from alkynyl-tethered cyclopropane ***d*-1y** (174 mg, 0.54 mmol, 90% D). Cycloadduct ***d*-2y'** (68.0 mg, 39% yield, 90% D, colorless oil) was obtained after purification by flash chromatography on silica gel (7:3 hexane/EtOAc). R_f = 0.42 (4:6 hexane/EtOAc). **^{1}H NMR (300 MHz, $CDCl_3$):** δ 7.75 (m, 2H), 7.33 (m, 2H), 4.02 (s, 4H), 3.90 (d, *J* = 5.3 Hz, 2H), 2.43 (s, 3H), 1.85 (s, 6H), 0.87 (t, *J* = 5.6 Hz, OH). **^{13}C NMR (75 MHz, $CDCl_3$):** δ 143.6 (C), 140.4 (C), 133.8 (C), 129.9 (C), 129.7 (2CH), 127.6 (2CH), 60.5 (CH_2), 46.5 ($2CH_2$), 21.5 (CH_3), 12.8 ($2CH_3$). **HRMS** (APCI-FIA-TOF): *m/z* calculated for $C_{17}H_{21}DNO_3S$ [M + H]$^+$: 321.1378, found 321.1368.

1,1,2-Trimethyl-3-tosyl-2,3,4,9b-tetrahydro-1*H*-benzo[*f*]cyclopropa[*d*]isoindole (4zc)

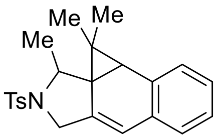

Prepared following the general procedure (extended reaction time to 96 h), from alkynyl-tethered cyclopropane **1zc** (38.0 mg, 0.12 mmol). Cycloadduct **4zc** (23.0 mg, 61% yield, white solid) was obtained after purification by flash chromatography on silica gel (100:2 → 100:5 hexane/EtOAc). R_f = 0.49 (8:2 hexane/EtOAc). **^{1}H NMR (300 MHz, $CDCl_3$):** δ 7.79 (m, 2H), 7.35 (m, 2H), 7.23 (m, 1H), 7.13 (m, 3H), 6.39 (s, 1H), 4.30 (dd, *J* = 14.4, 1.4 Hz, 1H), 4.13 (dd, *J* = 14.4, 2.3 Hz, 1H), 3.93 (q, *J* = 6.5 Hz, 1H), 2.45 (s, 3H), 2.16 (s, 1H), 1.19 (d, *J* = 6.5 Hz, 3H), 0.97 (s, 3H), 0.25 (s, 3H). **^{13}C NMR (75 MHz, $CDCl_3$):** δ 143.4 (C), 135.7 (C), 135.4 (C), 132.2 (C), 131.2 (C), 129.6 (2CH), 129.0 (CH), 127.5 (2CH), 127.1 (CH), 127.0 (CH), 126.2 (CH), 119.0 (CH), 56.9 (CH), 51.1 (CH_2), 44.1 (C), 36.6 (CH), 23.2 (CH_3), 21.8 (CH_3), 21.5 (CH_3), 14.1 (C), 14.0 (CH_3). **HRMS** (APCI-FIA-TOF): *m/z* calculated for $C_{23}H_{26}NO_2S$ [M + H]$^+$: 380.1679, found 380.1686.

5. Synthesis of cyclopentadienyl metal complexes

Synthesis of CpRh(I) - complexes of type [Cp^{R}Rh(cod)] (exemplified for [Cp^{2s}Rh(cod)]).[6]

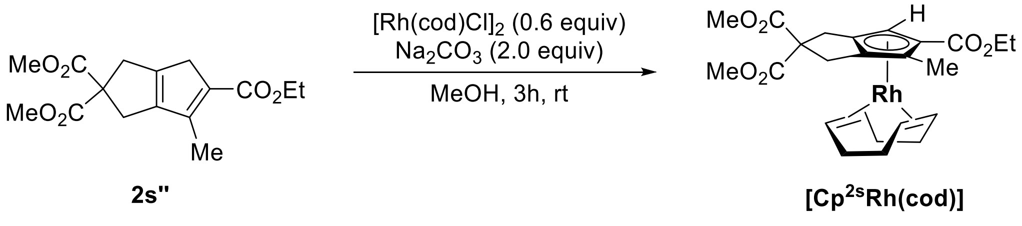

To a solution of cyclopentadiene **2s''** (10.0 mg, 0.032 mmol) in MeOH (0.5 mL) were added $[Rh(cod)Cl]_2$ (9.6 mg, 0.019 mmol) and Na_2CO_3 (7.6 mg, 0.07 mmol). The mixture was stirred at room temperature under inert atmosphere until complete consumption of starting material as indicated by TLC (typically 3 h). The solvent was removed under reduced pressure and the residue was purified by flash chromatography on silica gel (95:5

hexane/EtOAc) to afford 13.0 mg of the rhodium cyclopentadienyl-cyclooctadienyl complex **[Cp^{2s}Rh(cod)]** as a pale yellow solid (73 % yield). R$_f$ = 0.35 (8:2 hexane/EtOAc). **^{1}H NMR (500 MHz, CDCl$_3$):** δ 5.00 (s, 1H), 4.26 (m, 2H), 3.86 (s, 3H), 3.77 (s, 3H), 3.72 (m, 3H), 3.47 (m, 4H), 3.15 (t, *J* = 16.0 Hz, 2H), 2.13 (m, 4H), 1.91 (m, 7H), 1.33 (t, *J* = 7.1 Hz, 3H). **^{13}C NMR (125 MHz, CDCl$_3$):** δ 171.1 (CO), 170.6 (CO), 164.9 (CO), 114.6 (d, *J* = 3.5 Hz, C), 110.4 (d, *J* = 3.6 Hz, C), 95.0 (d, *J* = 3.5 Hz, C), 92.4 (d, *J* = 4.5 Hz, C), 78.3 (d, *J* = 4.1 Hz, CH), 72.6 (d, *J* = 14.0 Hz, CH), 70.1 (d, *J* = 13.7 Hz, CH), 64.8 (C), 59.2 (CH$_2$), 53.1 (CH$_3$), 53.0 (CH$_3$), 34.3 (CH$_2$), 33.3 (CH$_2$), 32.4 (CH$_2$), 31.5 (CH$_2$), 14.6 (CH$_3$), 10.8 (CH$_3$). **HRMS** (APCI-FIA-TOF): *m/z* calculated for $C_{24}H_{32}O_6Rh$ [M + H]$^+$: 519.1248, found 519.1266.

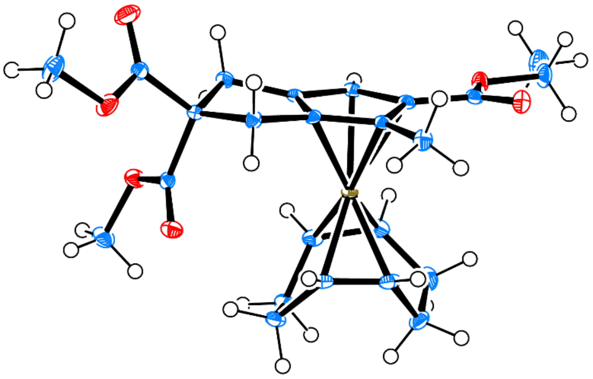

Figure S5. X-ray crystallographic analysis of **[Cp^{2s}Rh(cod)]** (CCDC 2348139)

Complex [Cp^{2p}Rh(cod)]

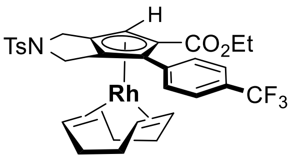

Prepared following the abovementioned general procedure from cycloadduct **2p''** (14.0 mg, 0.03 mmol) and [Rh(cod)Cl]$_2$ (8.7 mg, 0.018 mmol), to afford 17.5 mg of **[Cp^{2p}Rh(cod)]** (85% yield, yellow solid) after purification by flash chromatography on silica gel (95:5 hexane/EtOAc). R$_f$ = 0.55 (7:3 hexane/EtOAc). **^{1}H NMR (500 MHz, CDCl$_3$):** 7.73 (m, 2H), 7.57 (m, 4H), 7.34 (m, 2H), 5.76 (s, 1H), 4.28 (t, *J* = 11.2 Hz, 2H), 4.19 – 4.06 (m, 3H), 4.03 (d, *J* = 12.3 Hz, 1H), 3.62 (m, 2H), 3.54 (m, 2H), 2.42 (s, 3H), 2.28 – 2.16 (m, 4H), 2.00 – 1.87 (m, 4H), 1.17 (t, *J* = 7.1 Hz, 3H). **^{13}C NMR (125 MHz, CDCl$_3$):** δ 165.1 (CO), 144.0 (C), 137.1 (C), 133.5 (C), 130.0 (2CH), 129.9 (2CH), 129.1 (q, *J* = 32.4 Hz, C), 127.5 (2CH), 124.7 (q, *J* = 3.7 Hz, 2CH), 124.2 (q, *J* = 272.0 Hz, CF$_3$), 108.7 (d, *J* = 3.2 Hz, C), 106.2 (d, *J* = 3.4 Hz, C), 101.7 (d, *J* = 3.4 Hz, C), 90.5 (d, *J* = 4.4 Hz, C), 81.5 (d, *J* = 3.6 Hz, CH), 73.1 (d, *J* = 13.8 Hz, 2CH), 72.5 (d, *J* = 14.0 Hz, 2CH), 60.2 (CH$_2$), 47.4 (CH$_2$), 47.3 (CH$_2$), 32.0 (2CH$_2$), 31.9 (2CH$_2$), 21.5 (CH$_3$), 14.4 (CH$_3$). **^{19}F NMR (471 MHz, CDCl$_3$):** δ -62.5 (s, 3F, CF$_3$). **HRMS** (APCI-FIA-TOF): *m/z* calculated for $C_{32}H_{34}F_3NO_4RhS$ [M + H]$^+$: 688.1210, found 688.1225.

Complex [Cp^{2n}Rh(cod)]

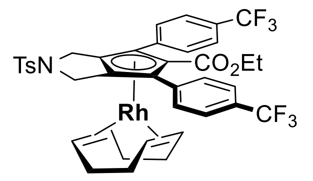

Prepared following the abovementioned general procedure from cycloadduct **2n'** (31.0 mg, 0.03 mmol) and $[Rh(cod)Cl]_2$ (14.8 mg, 0.03 mmol), to afford 30.0 mg of **[Cp^{2n}Rh(cod)]** (72% yield, pale yellow solid) after purification by flash chromatography on silica gel (95:5 hexane/EtOAc). R_f = 0.56 (8:2 hexane/EtOAc). **^{1}H NMR (500 MHz, CDCl$_3$):** 7.71 (m, 2H), 7.63 (s, 8H), 7.33 (m, 2H), 4.28 (d, *J* = 11.8 Hz, 2H), 4.02 (d, *J* = 11.9 Hz, 2H), 3.95 (q, *J* = 7.1 Hz, 2H), 3.64 (s, 4H), 2.42 (s, 3H), 2.32 – 2.26 (m, 4H), 2.00 (q, *J* = 8.0 Hz, 4H), 0.89 (t, *J* = 7.1 Hz, 3H). **^{13}C NMR (125 MHz, CDCl$_3$):** δ 165.4 (CO), 144.1 (C), 137.4 (C), 133.1 (C), 130.0 (2CH), 129.8 (4CH), 129.2 (q, *J* = 32.4 Hz, 2C), 127.6 (2CH), 125.0 (q, *J* = 3.7 Hz, 4CH), 124.1 (q, *J* = 272.1 Hz, $2CF_3$), 105.8 (d, *J* = 3.5 Hz, C), 102.4 (d, *J* = 3.6 Hz, C), 90.2 (d, *J* = 4.0 Hz, C), 74.7 (d, *J* = 13.7 Hz, 2CH), 60.6 (CH_2), 47.0 (CH_2), 31.8 (CH_2), 21.5 (CH_3), 13.7 (CH_3). **^{19}F NMR (471 MHz, CDCl$_3$):** δ -62.5 (s, 3F, CF_3). **HRMS** (APCI-FIA-TOF): *m/z* calculated for $C_{39}H_{37}F_6NO_4RhS$ $[M + H]^+$: 832.1397, found 832.1398.

Synthesis of complex [Cp^{2a}Rh(cod)]

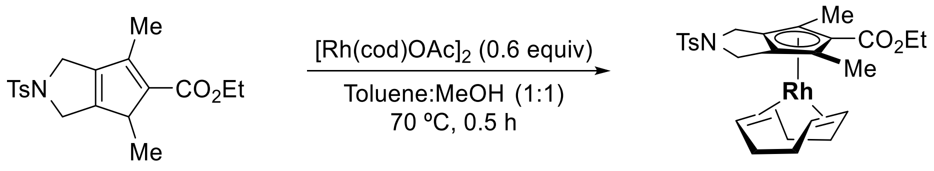

2a **[Cp^{2a}Rh(cod)]**

[Cp^{2a}Rh(cod)] was prepared by adapting a previously reported method, described by Cramer and coworkers.[7] In a sealed tube, $[Rh(cod)OAc]_2$ (60.0 mg, 0.11 mmol) and ethyl 4,6-dimethyl-2-tosyl-1,2,3,4-tetrahydrocyclopenta[*c*]pyrrole-5-carboxylate **2a** (73.0 mg, 0.20 mmol) were dissolved in a mixture of MeOH:Toluene (1:1, 1 mL) and stirred at 70 °C for 30 mins. Then, the solvent was removed under reduced pressure and the residue was purified by flash chromatography on silica gel (90:10 $\rightarrow$ 80:20 hexane/EtOAc) to afford 95 mg of **[Cp^{2a}Rh(cod)]** as a yellow foam (82 % yield). R_f = 0.44 (7:3 hexane/EtOAc). **^{1}H NMR (300 MHz, CDCl$_3$):** 7.80 (m, 2H), 7.35 (m, 2H), 4.35 (d, *J* = 11.1 Hz, 2H), 4.30 – 4.14 (m, 4H), 3.30 (s, 4H), 2.42 (s, 3H), 2.09 – 1.98 (m, 4H), 1.86 (m, 10H), 1.32 (t, *J* = 7.2 Hz, 3H). **^{13}C NMR (75 MHz, CDCl$_3$):** δ 166.2 (CO), 143.6 (C), 134.3 (C), 129.8 (2CH), 127.4 (2CH), 107.3 (d, *J* = 3.8 Hz, 2C), 93.4 (d, *J* = 3.8 Hz, 2C), 91.5 (d, *J* = 3.8 Hz, C), 73.7 (d, *J* = 13.9 Hz, 4CH), 59.3 (CH_2), 47.9 ($2CH_2$), 32.0 ($4CH_2$), 21.4 (CH_3), 14.5 (CH_3), 11.7 ($2CH_3$). **HRMS** (APCI-FIA-TOF): *m/z* calculated for $C_{27}H_{35}NO_4RhS$ $[M + H]^+$: 572.1336, found 572.1337.

Synthesis of CpRh(III) complexes of type [Cp^{R}RhI$_2$]$_2$ (exemplified for **[Cp^{2s}RhI$_2$]$_2$**).[6]

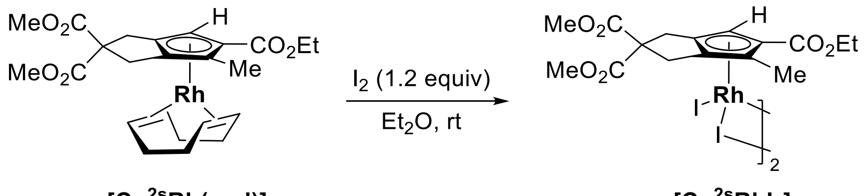

A solution of I_2 (30.5 mg, 0.12 mmol) in Et_2O (1 mL) was added to a stirred solution of **[Cp^{2s}Rh(cod)]** (44.6 mg, 0.1 mmol,) in Et_2O (1 mL) under air. After stirring for 30 min, the solid was filtered, washed with Et_2O and dried in vacuo, to give 35.0 mg of complex **[Cp^{2s}RhI$_2$]$_2$** as a dark purple solid (60% yield). **^{1}H NMR (500 MHz, DMSO):** δ 6.24 (s, 2H), 4.23 (m, *J* = 7.1 Hz, 4H), 3.69 (m, 12H), 3.57 (d, *J* = 18.2 Hz, 2H), 3.48 (d, *J* = 18.2 Hz, 2H), 2.26 (s, 6H), 1.27 (t, *J* = 7.1 Hz, 6H) (Some signals overlapped for the water present in the DMSO). **^{13}C NMR (125 MHz, DMSO):** δ 170.9 (CO), 168.8 (CO), 163.2 (CO), 119.5 (d, *J* = 4.9 Hz, C), 113.0 (d, *J* = 5.5 Hz, C) 100.3 (d, *J* = 4.4 Hz, C), 88.6 (d, *J* = 7.7 Hz, C), 82.1 (d, *J* = 6.6 Hz, CH), 64.7 (C), 61.7 (CH_2), 53.6 (CH_3), 52.9 (CH_3), 32.7 (CH_2), 32.5 (CH_2), 14.0 (CH_3), 12.6 (CH_3). **HRMS** (APCI-FIA-TOF): *m/z* calculated for $C_{32}H_{38}I_3O_{12}Rh_2$ [M + H - I]$^+$: 1200.7607, found 1200.7566.

Complex [Cp^{2a}RhI$_2$]$_2$

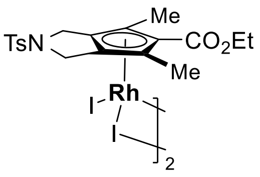

Prepared following the abovementioned general procedure, from complex **[Cp^{2a}Rh(cod)]** (30.0 mg, 0.052 mmol) and I_2 (16.0 mg, 0.063 mmol) to afford 33.0 mg of **[Cp^{2a}RhI$_2$]$_2$** (88% yield, dark purple solid). **^{1}H NMR (300 MHz, DMSO):** 7.76 (m, 2H), 7.46 (m, 2H), 4.42 – 4.16 (m, 6H), 2.41 (s, 3H), 2.22 (s, 6H), 1.27 (t, *J* = 7.1 Hz, 3H). **^{13}C NMR (75 MHz, DMSO):** δ 163.3 (2CO), 144.0 (2C), 133.1 (2C), 130.0 (4CH), 127.6 (4CH), 108.9 (d, *J* = 6.1 Hz, 2C), 98.6 (d, *J* = 5.5 Hz, 2C), 88.1 (d, *J* = 7.0 Hz, 2C), 61.6 (2CH$_2$), 46.9 (4CH$_2$), 21.0 (2CH$_3$), 13.9 (2CH$_3$), 13.0 (4CH$_3$). **HRMS** (APCI-FIA-TOF): *m/z* calculated for $C_{38}H_{45}I_2N_2O_8Rh_2S_2$ [M + H - 2I]$^+$: 1180.8811, found 1180.8817.

Complex [Cp^{2p}RhI$_2$]$_2$

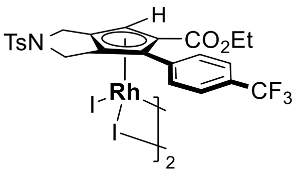

Prepared following the abovementioned general procedure, from complex **[Cp^{2p}Rh(cod)]** (42.0 mg, 0.062 mmol) and I_2 (19.0 mg, 0.074 mmol), to afford 35.1 mg of **[Cp^{2p}RhI$_2$]$_2$** (68% yield, dark purple solid. **^{1}H NMR (500 MHz, DMSO):** 7.84 (m, 4H), 7.78 (m, 4H), 7.75 (m, 4H), 7.43 (m, 4H), 6.63 (s, 2H), 4.69 (d, *J* = 14.6 Hz, 2H), 4.65 (d, *J* = 14.6 Hz, 2H), 4.41 (d, *J* = 14.3 Hz, 2H), 4.16 (m, 6H), 2.40 (s, 6H), 1.14 (t, *J* = 7.1 Hz, 6H) **^{13}C NMR (125 MHz, DMSO):** δ 161.7 (2CO), 143.9 (2C), 133.3 (2C), 132.2 (2C), 132.1 (4CH), 130.0 (4CH), 129.3 (q, *J* = 31.9 Hz, 2C), 127.7 (4CH), 124.3 (q, *J* = 3.6 Hz, 4CH), 124.0 (q, *J* = 272.3 Hz, 2CF$_3$), 117.4 (d, *J* = 4.5 Hz, 2C), 106.8 (d, *J* = 5.7 Hz, 2C), 93.6 (d, *J* = 4.7 Hz, 2C), 93.2 (d, *J* = 6.8 Hz, 2C), 82.8 (d, *J* = 5.9 Hz, 2CH), 62.0 (2CH$_2$), 48.2

(2CH$_2$), 46.9 (2CH$_2$), 21.0 (2CH$_3$), 13.6 (2CH$_3$). **^{19}F NMR (471 MHz, DMSO):** δ -63.2 (s, 6F, 2CF$_3$). **HRMS** (APCI-FIA-TOF): *m/z* calculated for $C_{48}H_{43}F_6I_2N_2O_8Rh_2S_2$ [M + H - 2I]$^+$: 1412.8559, found 1412.8540.

Complex [Cp^{2n}RhI$_2$]$_2$

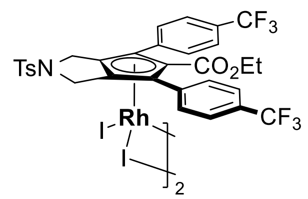

Prepared following the abovementioned general procedure from complex **[Cp^{2n}Rh(cod)]** (27.0 mg, 0.032 mmol) and I_2 (10.0 mg, 0.039 mmol) to afford 24.2 mg of **[Cp^{2n}RhI$_2$]$_2$** (77% yield, dark purple solid) after filtering and subsequent washing with Et$_2$O. **^{1}H NMR (500 MHz, DMSO):** 7.88 (m, 8H), 7.80 (m, 12H), 7.43 (m, 4H), 4.73 (d, *J* = 12.7 Hz, 4H), 4.62 (d, *J* = 12.7 Hz, 4H), 4.06 (q, *J* = 7.2 Hz, 4H), 2.40 (s, 6H), 0.88 (t, *J* = 7.1 Hz, 6H). **^{13}C NMR (125 MHz, DMSO):** δ 161.1 (2CO), 143.9 (2C), 132.9 (2C), 132.4 (4C), 131.5 (8CH), 130.0 (4CH), 129.5 (q, *J* = 32.5 Hz, 4C), 127.9 (4CH), 124.9 (q, *J* = 3.6 Hz, 8CH), 124.0 (q, *J* = 272.3 Hz, 4CF$_3$), 110.0 (d, *J* = 4.9 Hz, 4C), 95.9 (d, *J* = 6.4 Hz, 2C), 94.3 (d, *J* = 5.1 Hz, 4C), 62.5 (2CH$_2$), 48.2 (4CH$_2$), 21.0 (2CH$_3$), 12.9 (2CH$_3$). **^{19}F NMR (471 MHz, DMSO):** δ -61.3 (s, 12F, 4CF$_3$). **HRMS** (APCI-FIA-TOF): *m/z* calculated for $C_{62}H_{49}F_{12}I_2N_2O_8Rh_2S_2$ [M + H - 2I]$^+$: 1700.8933, found 1700.8887.

Synthesis of CpIr(I) complexes of type [Cp^{R}Ir(cod)] (exemplified for [Cp^{2a}Ir(cod)]).[7]

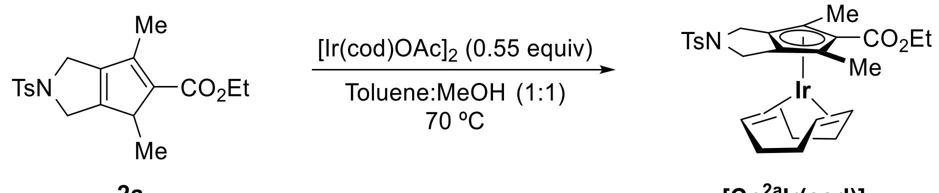

2a

[Cp^{2a}Ir(cod)]

In a sealed tube, [Ir(cod)OAc]$_2$ (22.0 mg, 0.03 mmol) and ethyl 4,6-dimethyl-2-tosyl-1,2,3,4-tetrahydrocyclopenta[*c*]pyrrole-5-carboxylate **2a** (20.0 mg, 0.055 mmol) were dissolved in a mixture of MeOH:Toluene (1:1, 0.5 mL), and stirred at 70 °C. Once TLC analysis indicated complete conversion, the solvent was removed under reduced pressure and the crude residue was purified by flash chromatography on silica gel (95:5 $\rightarrow$ 90:10 hexane/EtOAc), to afford 95.1 mg of **[Cp^{2a}Ir(cod)]** as a colorless oil (82 % yield). R_f = 0.64 (7:3 hexane/EtOAc). **^{1}H NMR (300 MHz, CDCl$_3$):** 7.78 (m, 2H), 7.36 (m, 2H), 4.32 (s, 4H), 4.24 (t, *J* = 7.1 Hz, 2H), 3.12 (m, 4H), 2.43 (s, 3H), 1.99 (s, 6H), 1.92 – 1.73 (m, 8H), 1.31 (t, *J* = 7.1 Hz, 3H). **^{13}C NMR (75 MHz, CDCl$_3$):** δ 166.4 (CO), 143.7 (C), 134.3 (C), 129.8 (2CH), 127.4 (2CH), 103.8 (C), 88.8 (C), 84.8 (C), 59.6 (CH$_2$), 57.0 (4CH), 47.2 (2CH$_2$), 33.4 (4CH$_2$), 21.5 (CH$_3$), 14.4 (CH$_3$), 11.1 (2CH$_3$). **HRMS** (APCI-FIA-TOF): *m/z* calculated for $C_{27}H_{35}IrNO_4S$ [M + H]$^+$: 662.1911, found 662.1929.

Complex [Cp^{2p}Ir(cod)]

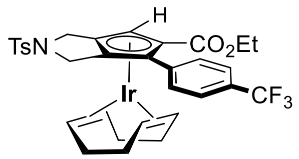

Prepared following the abovementioned procedure, from cyclopentadiene **2p''** (20.1 mg, 0.042 mmol) using [Ir(cod)OMe]$_2$ (17.0 mg, 0.026 mmol) instead of [Ir(cod)OAc]$_2$. **[Cp^{2p}Ir(cod)]** (28.1 mg, 85% yield, white solid. R_f = 0.55 (7:3 hexane/EtOAc). **^{1}H NMR (300 MHz, CDCl$_3$):** 7.71 (m, 2H), 7.57 (s, 4H), 7.34 (m, 2H), 5.86 (s, 1H), 4.33 (m, 2H), 4.22 (m, 2H), 4.09 (m, 2H), 3.44 (m, 2H), 3.34 (m, 2H), 2.43 (s, 3H), 2.09 (m, 4H), 1.87 (m, 4H), 1.13 (t, J = 7.1 Hz, 3H). **^{13}C NMR (75 MHz, CDCl$_3$):** δ 164.8 (CO), 144.1 (C), 136.4 (C), 133.5 (C), 130.6 (2CH), 129.9 (2CH), 129.4 (q, J = 32.7 Hz, C), 127.5 (2CH), 124.8 (q, J = 3.7 Hz, 2CH), 124.1 (q, J = 271.9 Hz, CF$_3$), 105.4 (C), 102.8 (C), 96.1 (C), 84.9 (C), 75.2 (CH), 60.4 (CH$_2$), 56.8 (2CH), 56.1 (2CH), 46.8 (CH$_2$), 46.4 (CH$_2$), 33.4 (2CH$_2$), 33.3 (2CH$_2$), 21.5 (CH$_3$), 14.4 (CH$_3$). **^{19}F NMR (282 MHz, CDCl$_3$):** δ -62.6 (s, 3F, CF$_3$). **HRMS** (APCI-FIA-TOF): m/z calculated for $C_{32}H_{34}F_3IrNO_4S$ [M + H]$^+$: 778.1784, found 778.1807.

Complex [Cp^{2n}Ir(cod)]

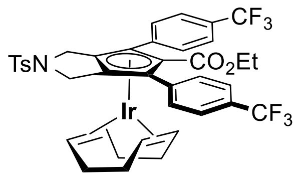

Prepared following the abovementioned procedure from cyclopentadiene **2n'** (15.1 mg, 0.024 mmol), using [Ir(cod)OMe]$_2$ (9.6 mg, 0.014 mmol) instead of [Ir(cod)OAc]$_2$. **[Cp^{2n}Ir(cod)]** [(flash chromatography on silica gel (95:5 hexane/EtOAc), 22.1 mg, 99% yield, white solid]. R_f = 0.86 (8:2 hexane/EtOAc). **^{1}H NMR (500 MHz, CDCl$_3$):** 7.69 (m, 2H), 7.62 (m, 8H), 7.33 (m, 2H), 4.30 (d, J = 10.8 Hz, 2H), 4.20 (d, J = 10.6 Hz, 2H), 3.90 (q, J = 7.1 Hz, 2H), 3.46 (d, J = 4.0 Hz, 4H), 2.43 (s, 3H), 2.19 (m, 4H), 1.93 (q, J = 8.2 Hz, 4H), 0.84 (t, J = 7.1 Hz, 3H). **^{13}C NMR (125 MHz, CDCl$_3$):** δ 164.7 (CO), 144.2 (C), 136.7 (C), 133.2 (C), 130.5 (4CH), 130.0 (2CH), 129.6 (q, J = 32.7 Hz, 2C), 127.6 (2CH), 125.0 (q, J = 3.7 Hz, 4CH), 124.1 (q, J = 35.1 Hz, 2CF$_3$), 103.0 (2C), 96.5 (2C), 85.0 (C), 60.6 (CH$_2$), 58.3 (4CH), 46.3 (2CH$_2$), 33.2 (4CH$_2$), 21.5 (CH$_3$), 13.8 (CH$_3$). **^{19}F NMR (471 MHz, CDCl$_3$):** δ -62.6 (s, 6F, 2CF$_3$). **HRMS** (APCI-FIA-TOF): m/z calculated for $C_{39}H_{37}F_6IrNO_4S$ [M + H]$^+$: 922.1971, found 922.1963.

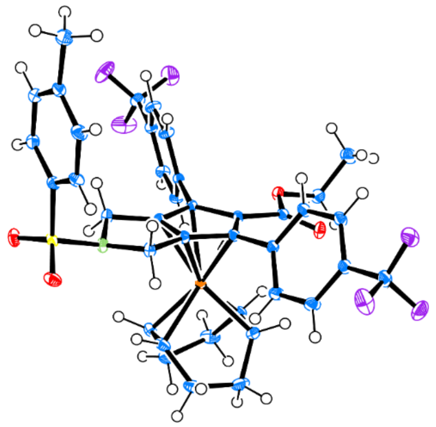

Figure S6. X-ray crystallographic analysis of **[Cp^{2n}Ir(cod)]** (CCDC 2348487)

Synthesis of CpIr(III) complexes of type [Cp^R Ir(CO)I2] (exemplified for [Cp^2a Ir(CO)I2])

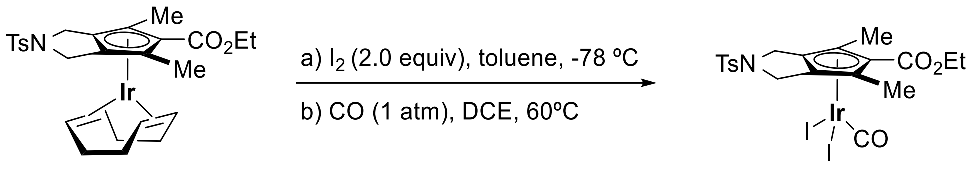

Cyclopentadienyl Ir(III)-*bis*-iodine-carbonyl complexes of type **[Cp^RIr(CO)I_2]** were prepared following an adapted method previously described by Matsunaga and coworkers.[8] **[Cp^{2a}Ir(cod)]** (50.0 mg, 0.076 mmol) was added to a flame-dried round-bottom flask, equipped with a stirring bar, under argon. Toluene (3.8 mL) was added via a syringe and the resulting mixture was cooled down to -78 °C. A solution of I_2 (38.4 mg, 0.151 mmol) in toluene (3.8 mL) was added dropwise at the same temperature and the mixture was stirred for 1 h. The reaction mixture was warmed up to room temperature and the solvent was removed under reduced pressure. The resulting crude residue was redissolved in 1,2-DCE (1.1 mL) and the solution was stirred at 60 °C for 24 h under a CO atmosphere (1 atm). The mixture was concentrated in vacuo and the resulting crude residue was purified by flash chromatography on silica gel (1:1 → 0:1 hexane/CH_2Cl_2), to afford 40.2 mg of **[Cp^{2a}Ir(CO)I_2]** as a deep red oil (63 % global yield for the 2 steps). R_f = 0.4 (CH_2Cl_2). **^{1}H NMR (500 MHz, $CDCl_3$):** 7.77 (m, 2H), 7.35 (m, 2H), 4.82 (d, *J* = 13.6 Hz, 2H), 4.52 (*J* = 12.7 Hz, 2H), 4.36 (q, *J* = 7.0 Hz, 2H), 2.53 (s, 6H), 2.44 (s, 3H), 1.37 (t, *J* = 7.0 Hz, 3H). **^{13}C NMR (125 MHz, $CDCl_3$):** δ 162.3 (CO), 158.9 (CO), 144.8 (C), 133.9 (C), 130.2 (2CH), 127.9 (2CH), 107.6 (C), 100.0 (C), 84.1 (C), 62.6 (CH_2), 46.3 ($2CH_2$), 21.6 (CH_3), 14.3 (CH_3), 13.3 (CH_3). **IR** (ATR, cm^{-1}): 2049.00, 1722.12, 1596.29, 1442.01, 1380.30, 1346.07, 1327.75, 1305.09, 1232.29, 1160.45, 1093.44, 1052.46, 852.86, 811.40, 770.90, 734.26, 704.85, 663.39, 599.27, 557.33, 545.27, 528.88, 491.28. **HRMS** (APCI-FIA-TOF): *m/z* calculated for $C_{19}H_{22}IIrNO_4S$ [M - I, - CO]$^+$: 679.9943, found 679.9941.

Complex [Cp^{2p}Ir(CO)I_2]

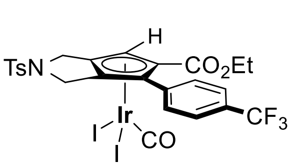

Prepared following the abovementioned procedure, from complex **[Cp^{2p}Ir(cod)]** (106 mg, 0.136 mmol) and I_2 (69.3 mg, 0.27 mmol), to afford 81.2 mg of **[Cp^{2p}Ir(CO)I_2]** [purification by flash chromatography on silica gel (7:3 hexane/EtOAc), 62% global yield, deep red oil]. R_f = 0.3 (7:3 hexane/EtOAc). **^{1}H NMR (500 MHz, $CDCl_3$):** 7.76 (m, 2H), 7.67 (m, 2H), 7.57 (m, 2H), 7.34 (m, 2H), 6.21 (s, 1H), 5.06 (d, *J* = 14.1 Hz, 1H), 4.96 (d, *J* = 14.4 Hz, 1H), 4.80 (d, *J* = 13.8 Hz, 1H), 4.72 (d, *J* = 14.2 Hz, 1H), 4.28 (m, 2H), 2.43 (s, 3H), 1.24 (m, 3H). **^{13}C NMR (125 MHz, $CDCl_3$):** δ 160.4 (CO), 157.0 (CO), 144.9 (C), 133.8 (C), 132.3 (q, *J* = 35.1 Hz, C), 131.2 (2CH), 130.3 (2CH), 129.8 (C), 127.9 (2CH), 125.6 (m, 2CH), 123.4 (q, *J* = 272.7 Hz, CF_3), 115.5 (C), 107.8 (C), 97.5 (C), 89.2 (C), 78.7 (CH), 63.4 (CH_2), 47.2 ($2CH_2$), 21.6 (CH_3), 14.0 (CH_3). **^{19}F NMR (471 MHz, $CDCl_3$):** δ -63.0 (s, 3F, CF_3). **IR** (ATR, cm^{-1}): 2064.91, 1736.58, 1697.53, 1647.39, 1558.68, 1539.88, 1507.10, 1457.92, 1349.44, 1323.89, 1231.33,

1162.87, 1129.12, 1069.82, 1018.23, 839.36, 814.29, 736.19, 665.80, 592.52, 547.68. **HRMS** (APCI-FIA-TOF): *m/z* calculated for $C_{24}H_{21}IIrNO_4S$ [M - I, - CO]$^+$: 795.9817, found 795.9822.

Complex [Cp^{2n}Ir(CO)I$_2$]

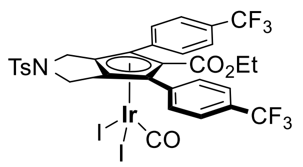

Prepared following the abovementioned procedure, from complex **[Cp^{2n}Ir(cod)]** (112 mg, 0.122 mmol) and I_2 (61.7 mg, 0.24 mmol), to afford 88.0 mg of **[Cp^{2n}Ir(CO)I$_2$]** [purification by flash chromatography on silica gel (8:2 → 7:3 hexane/EtOAc), 66% global yield, deep red solid]. R_f = 0.48 (7:3 hexane/EtOAc). **^{1}H NMR (500 MHz, CDCl$_3$):** 7.78 (m, 2H), 7.71 (m, 4H), 7.54 (m, 4H), 7.32 (m, 2H), 5.30 (m, 2H), 4.98 (m, 2H), 4.21 (q, *J* = 7.1 Hz, 2H), 2.42 (s, 3H), 1.08 (t, *J* = 7.1 Hz, 3H). **^{13}C NMR (125 MHz, CDCl$_3$):** δ 160.5 (CO), 157.2 (CO), 144.9 (C), 133.8 (C), 132.4 (q, *J* = 33.3 Hz, 2C), 130.4 (4CH), 130.3 (2CH), 129.7 (C), 128.0 (2CH), 126.2 (q, *J* = 3.7 Hz, 4CH), 123.4 (q, *J* = 272.7 Hz, 2CF$_3$), 111.9 (2C), 95.4 (C), 91.6 (2C), 64.5 (CH$_2$), 47.4 (2CH$_2$), 21.6 (CH$_3$), 13.5 (CH$_3$). **^{19}F NMR (471 MHz, CDCl$_3$):** δ -63.1 (s, 6F, 2CF$_3$). **IR** (ATR, cm^{-1}): 2065.39, 1735.14, 1618.95, 1597.73, 1447.80, 1409.71, 1380.78, 1322.45, 1243.38, 1158.53, 1128.64, 1112.24, 1096.33, 1069.33, 1015.82, 838.88, 814.78, 762.23, 735.71, 704.85, 669.18, 593.00, 548.17, 527.44, 487.42. **HRMS** (APCI-FIA-TOF): *m/z* calculated for $C_{31}H_{24}F_6IIrNO_4S$ [M - I, - CO]$^+$: 940.0004, found 940.0001.

Synthesis of CpIr(III) complexes of type [Cp^{R}IrI$_2$]$_2$ (exemplified for [Cp^{2a}IrI$_2$]$_2$.[8]

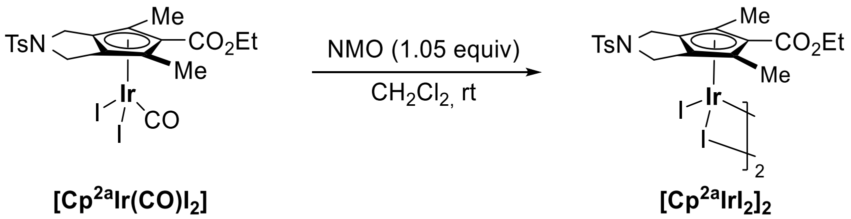

[Cp^{2a}Ir(CO)I$_2$] (40.0 mg, 0.048 mmol) and CH_2Cl_2 (0.6 mL) were added to an oven dried screw-capped vial equipped with a stirring bar, under Ar. Then, NMO (5.90 mg, 0.05 mmol) was added, and the resulting mixture was stirred for 30 min at room temperature. The solvent was removed under reduced pressure and the crude residue was purified by flash chromatography on silica gel (95:5 CH_2Cl_2/EtOAc), to afford 30.2 mg of **[Cp^{2a}IrI$_2$]$_2$** as a brown solid (77 % yield). R_f = 0.72 (9:1 CH_2Cl_2/EtOAc). **^{1}H NMR (500 MHz, CDCl$_3$):** 7.72 (m, 4H), 7.28 (m, 4H), 4.33 – 4.21 (m, 12H), 2.37 (s, 6H), 2.15 (s, 12H), 1.29 (t, *J* = 7.1 Hz, 6H). **^{13}C NMR (125 MHz, CDCl$_3$):** δ 166.0 (2CO), 144.5 (2C), 133.8 (2C), 130.2 (4CH), 127.9 (4CH), 93.4 (2C), 87.8 (2C), 74.0 (2C), 62.3 (2CH$_2$), 46.6 (4CH$_2$), 21.6 (2CH$_3$), 14.4 (2CH$_3$), 13.0 (4CH$_3$). **HRMS** (APCI-FIA-TOF): *m/z* calculated for $C_{38}H_{45}I_2Ir_2N_2O_8S_2$ [M + H - 2I]$^+$: 1360.9960, found 1360.9921.

Complex $[Cp^{2p}IrI_2]_2$

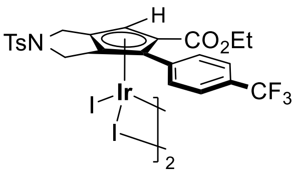

Prepared following the abovementioned procedure, from **$[Cp^{2p}Ir(CO)I_2]$** (55.0 mg, 0.058 mmol) and NMO (7.1 mg, 0.061 mmol), to afford 25.3 mg of **$[Cp^{2p}IrI_2]_2$** [purification by flash chromatography on silica gel (99:1 → 98:2 CH_2Cl_2/EtOAc), 47% yield, reddish brown solid]. R_f = 0.31 (99:1 CH_2Cl_2/EtOAc). **^{1}H NMR (500 MHz, $CDCl_3$):** 7.76 (m, 7H), 7.72 (m, 5H), 7.3 (m, 4H), 6.69 (s, 2H), 4.54 (d, *J* = 14.2 Hz, 2H), 4.45 (dd, *J* = 13.8, 1.8 Hz, 2H), 4.33 (dd, *J* = 13.4, 1.6 Hz, 2H), 4.23 (d, *J* = 13.9 Hz, 2H), 4.13 (q, *J* = 7.1 Hz, 4H), 2.39 (s, 6H), 1.10 (t, *J* = 7.1 Hz, 6H). **^{13}C NMR (125 MHz, $CDCl_3$):** δ 161.8 (2CO), 143.9 (2C), 133.2 (2C), 132.4 (2C), 132.1 (4CH), 130.0 (4CH), 129.1 (q, *J* = 32.0 Hz, 2C), 127.7 (4CH), 124.5 (q, *J* = 3.9 Hz, 4CH), 124.0 (q, *J* = 272.5 Hz, $2CF_3$), 113.2 (2C), 100.1 (2C), 87.3 (2C), 85.8 (2C), 76.3 (2CH), 62.0 ($2CH_2$), 47.4 ($2CH_2$), 46.6 ($2CH_2$), 21.0 ($2CH_3$), 13.7 ($2CH_3$). **^{19}F NMR (471 MHz, DMSO):** δ -61.2 (s, 6F, $2CF_3$). **HRMS** (APCI-FIA-TOF): *m/z* calculated for $C_{48}H_{43}F_6I_2Ir_2N_2O_8S_2$ [M + H - 2I]$^+$: 1592.9707, found 1592.9678.

Complex $[Cp^{2n}IrI_2]_2$

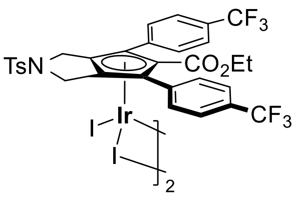

Prepared following the abovementioned procedure, from **$[Cp^{2n}Ir(CO)I_2]$** (49 mg, 0.045 mmol) and NMO (5.5 mg, 0.047 mmol), to afford 30.1 mg of **$[Cp^{2n}IrI_2]_2$** [purification by flash chromatography on silica gel (CH_2Cl_2), 62% yield, deep red solid]. R_f = 0.62 (CH_2Cl_2). **^{1}H NMR (500 MHz, DMSO):** 7.85 (m, 7H), 7.79 (m, 11H), 7.43 (m, 5H), 4.55 (s, 8H), 4.01 (q, *J* = 7.1 Hz, 4H), 2.40 (s, 6H), 0.83 (t, *J* = 7.1 Hz, 6H). **^{13}C NMR (125 MHz, DMSO):** δ 161.1 (2CO), 143.9 (2C), 132.9 (2C), 132.5 (4C), 131.4 (8CH), 130.0 (8CH), 129.3 (q, *J* = 31.9 Hz, 4C), 127.9 (8CH), 125.0 (m, 8CH), 124.0 (q, *J* = 272.5 Hz, $4CF_3$), 106.0 (4C), 89.8 (2C), 85.7 (4C), 62.5 ($2CH_2$), 47.6 ($4CH_2$), 21.0 ($2CH_3$), 12.7 ($2CH_3$) **^{19}F NMR (471 MHz, DMSO):** δ -61.3 (s, 12F, $4CF_3$). **HRMS** (APCI-FIA-TOF): *m/z* calculated for $C_{62}H_{49}F_{12}I_2Ir_2N_2O_8S_2$ [M + H - 2I]$^+$: 1881.0081, found 1881.0134.

6. Catalytic transformations promoted by CpM(III) complexes

Rh(III)-catalyzed annulation of *N*-(pivaloyloxy)benzamide with styrene (synthesis of **6**)

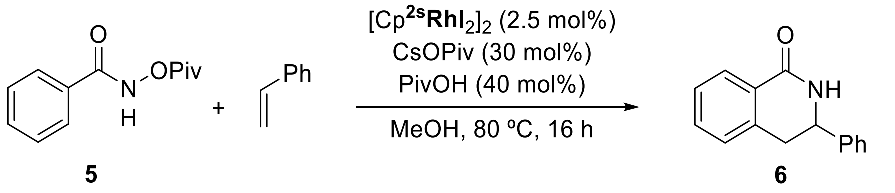

Reaction carried out following the general procedure described by Glorius et al.,[9] using **$[Cp^{2s}RhI_2]_2$** instead of $[Cp^*RhCl_2]_2$. A dried sealed tube equipped with a stirring bar and filled with argon was charged with **$[Cp^{2s}RhI_2]_2$** (4.50 mg, 0.003 mmol, 2.5 mol%), CsOPiv (9.50 mg, 0.041 mmol, 30 mol%), PivOH (2.80 mg, 0.027 mmol, 20 mol%), *N*-(pivaloyloxy)benzamide **5** (30.0 mg, 0.136 mmol), styrene (21.1 mg, 0.20 mmol, 23.5 µL) and dry

MeOH (0.8 mL). The tube was capped with a rubber septum and stirred at 80 °C for 16 h. After cooling to room temperature, the solvent was removed under reduced pressure and the crude residue was purified by flash chromatography on silica gel (7:3 hexane/EtOAc), to afford 23.1 mg of 3-phenyl-3,4-dihydroisoquinolin-1(2*H*)-one **6** as a white solid (77% yield). R_f = 0.46 (1:1 hexane/EtOAc). **^{1}H NMR (300 MHz, $CDCl_3$):** δ 8.11 (d, *J* = 7.6 Hz, 1H), 7.51 – 7.30 (m,7H), 7.17 (d, *J* = 7.4 Hz, 1H), 6.42 (s, 1H), 4.84 (m, 1H), 3.15 (m, 3H). **^{13}C NMR (75 MHz, $CDCl_3$):** δ 166.2 (C), 140.9 (C), 137.4 (C), 132.3 (CH), 128.8 (2CH), 128.3 (C), 128.2 (CH), 127.9 (CH), 127.2 (CH), 127.1 (CH), 126.3 (2CH), 55.9 (CH), 37.2 (CH_2). **HRMS** (APCI-FIA-TOF): *m/z* calculated for $C_{15}H_{14}NO$ $[M + H]^+$: 224.1070, found 224.1061.

Rh(III)-catalyzed annulation of 2-alkenyl anilide 7 with diphenylacetylene (synthesis of **8**)

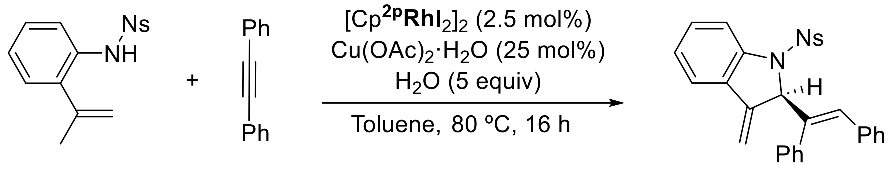

7 **8**

Reaction carried out following the procedure previously described by Mascareñas, Gulías et al.,[10] using **[Cp^{2p}RhI$_2$]$_2$** instead of $[Cp^ERhCl_2]_2$. A Schlenk tube was loaded with 4-nitro-*N*-(2-(prop-1-en-2-yl)phenyl)benzenesulfonamide **7** (20.0 mg, 0.063 mmol), **[Cp^{2p}RhI$_2$]$_2$** (3.70 mg, 0.002 mmol, 2.5 mol%), $Cu(OAc)_2 \cdot H_2O$ (2.90 mg, 0.016 mmol, 25 mol%), diphenylacetilene (16.8 mg, 0.094 mmol), H_2O (5.7 mg, 0.314 mmol, 5.7 µL) and toluene (0.7 mL). The tube was capped with a rubber septum and stirred at 80 °C under an atmosphere of air (with a balloon of air), for 16 h. The solvent was removed under reduced pressure and the crude residue was purified by flash chromatography on silica gel (9:1 → 8:2 hexane/EtOAc) to afford 16.0 mg of (*E*)-2-(1,2-diphenylvinyl)-3-methylene-1-((4-nitrophenyl)sulfonyl)indoline **8** as yellow solid (52% yield). R_f = 0.42 (8:2 hexane/EtOAc). **^{1}H NMR (300 MHz, $CDCl_3$):** δ 8.23 (m, 2H), 7.95 (m, 2H), 7.62 (d, *J* = 8.1 Hz, 1H), 7.27 (m, 1H), 7.20 (s, 5H), 7.11 (m, 3H), 7.03 (t, *J* = 7.5 Hz, 1H), 6.96 (m, 2H), 6.72 (s, 1H), 5.46 (m, 2H), 4.99 (m, 1H). **^{13}C NMR (75 MHz, $CDCl_3$):** δ 150.3 (C), 143.4 (C), 143.0 (C), 143.0 (C), 139.7 (C), 136.5 (C), 135.9 (C), 130.3 (CH), 130.0 (2CH), 129.9 (2CH), 129.7 (C), 129.4 (2CH), 128.3 (4CH), 127.9 (2CH), 127.6 (CH), 127.3 (CH), 124.9 (CH), 124.3 (2CH), 121.3 (CH), 115.7 (2CH), 105.2 (CH_2), 73.8 (CH). **HRMS** (APCI-FIA-TOF): *m/z* calculated for $C_{29}H_{23}N_2O_4S$ $[M + H]^+$: 495.1373, found 495.1378.

Rh(III)-catalyzed annulation of anilide 9 with diphenylacetylene (synthesis of **10**)

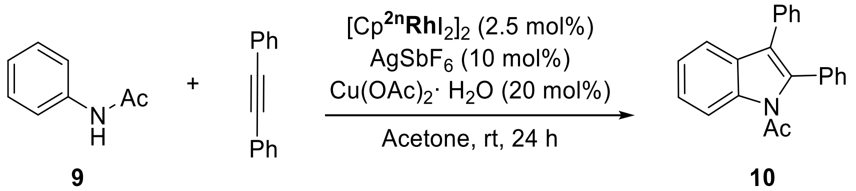

9 **10**

Prepared following the general procedure described by Tanaka et al.,[11] using **[Cp^{2n}RhI$_2$]$_2$** instead of [Cp^{E}RhCl$_2$]$_2$. AgSbF$_6$ (3.8 mg, 0.011 mmol, 10 mol%), **[Cp^{2n}RhI$_2$]$_2$** (5.4 mg, 0.003 mmol, 2.5 mol%), Cu(OAc)$_2$·H$_2$O (4.4 mg, 0.022 mmol, 20 mol%), *N*-phenylacetamide **9** (15 mg, 0.11 mmol), diphenylacetylene (21.8 mg, 0.12 mmol), and acetone (0.5 mL) were added to a screw-cap vial, under air. The mixture was sealed and stirred at rt under air for 24 h. The solvent was removed and the crude was purified by flash chromatography on silica gel (85:15 hexane/EtOAc) to afford 34.1 mg of 1-(2,3-diphenyl-1*H*-indol-1-yl)ethan-1-one **10**, as white solid (99% yield). R$_f$ = 0.55 (8:2 hexane/EtOAc). **^{1}H NMR (300 MHz, CDCl$_3$):** δ 8.50 (d, *J* = 8.3 Hz, 1H), 7.60 (d, *J* = 7.3 Hz, 1H), 7.49 – 7.23 (m, 12H), 2.04 (s, 3H). **^{13}C NMR (75 MHz, CDCl$_3$):** δ 171.5 (CO), 136.8 (C), 135.0 (C), 133.1 (C), 133.0 (C), 130.8 (2CH), 130.0 (2CH), 129.3 (C), 128.6 (2CH), 128.2 (2CH), 126.9 (CH), 125.5 (CH), 123.7 (CH), 123.4 (C), 119.5 (CH), 116.2 (CH), 27.9 (CH$_3$). **HRMS** (APCI-FIA-TOF): *m/z* calculated for C$_{22}$H$_{18}$NO [M + H]$^+$: 312.1383, found 312.1388.

Ir(III)-catalyzed C-H amidation of benzyl methyl ether with tosyl azide (synthesis of **12**)

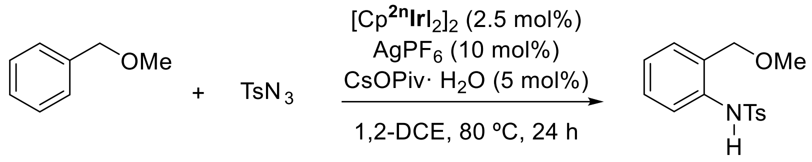

11 **12**

The reaction was carried out following the procedure previously described by Matsunaga and coworkers,[8] using **[Cp^{2n}IrI$_2$]$_2$** instead of [Cp^{E}IrI$_2$]$_2$, and at 80 °C. To a sealed tube equipped with a stirring bar, (methoxymethyl)benzene **11** (20 mg, 0.1 mmol), tosyl azide (15 mg, 0.12 mmol), **[Cp^{2n}IrI$_2$]$_2$** (5.4 mg, 0.003 mmol, 2.5 mol%), AgPF$_6$ (2.6 mg, 0.01 mmol, 10 mol%), CsOPiv (1.2 mg, 0.005 mmol, 5 mol%) and 1,2-DCE (1 mL) were added, under argon. The reaction mixture was stirred for 24 h at 80 °C and cooled down to room temperature. The solvent was removed under reduced pressure and the crude was purified by flash chromatography on silica gel (85:15 hexane/EtOAc), to afford 18.1 mg of *N*-(2-(methoxymethyl)phenyl)-4-methylbenzenesulfonamide **12** as white solid (63% yield). R$_f$ = 0.4 (7:3 hexane/EtOAc). **^{1}H NMR (300 MHz, CDCl$_3$):** δ 8.01 (s, 1H), 7.67 (m, H), 7.57 (d, *J* = 7.7 Hz, 1H), 7.30 (m, 1H), 7.23 (m, 2H), 7.07 (m, 2H), 4.13 (s, 2H), 3.30 (s, 3H), 2.40 (s, 3H). **^{13}C NMR (75 MHz, CDCl$_3$):** δ 143.7 (C), 137.3 (C), 136.7 (C), 129.6 (2CH), 129.3 (CH), 129.3 (CH), 128.1 (C), 126.9 (2CH), 124.7 (CH), 122.6 (CH), 73.4 (CH$_2$), 57.9 (CH$_3$), 21.5 (CH$_2$). **HRMS** (APCI-FIA-TOF): *m/z* calculated for C$_{15}$H$_{18}$NO$_3$S [M + H]$^+$: 292.1002, found 292.1002.

7. Computational details

All the calculations reported in this paper were performed with the Gaussian 09 suite of programs.[12] Electron correlation was partially taken into account using the hybrid functional usually denoted as B3LYP[13] in conjunction with the D3 dispersion correction suggested by Grimme et al.[14] using the standard double-ζ

quality def2-SVP[15] basis set for all atoms. The Polarization Continuum Model (PCM)[16] was used to model the effects of the solvent (dichloroethane = DCE). Geometries were fully optimized in solution without any geometry or symmetry constraints. Reactants, intermediates, and products were characterized by frequency calculations,[17] and have positive definite Hessian matrices. Transition structures (TS's) show only one negative eigenvalue in their diagonalized force constant matrices, and their associated eigenvectors were confirmed to correspond to the motion along the reaction coordinate under consideration using the Intrinsic Reaction Coordinate (IRC) method.[18] This computational level was selected to enable a direct comparison with the analogous process involving the cobalt(I)-catalyzed intramolecular (3+2) cycloadditions of ynilidenecyclopropanes, reported recently by us.[19] Single-point energy calculations were carried out at the same DFT level in conjunction with the much larger def2-TZVPP[4] basis set. This level is denoted PCM(DCE)-B3LYP-D3/def2-TZVPP//PCM(DCE)-B3LYP-D3/def2-SVP. The computed thermochemistry data were corrected following Grimme's quasi-harmonic (QHA) model for entropy[20] with a frequency cut-off value of 100.0 cm^{-1} using the GoodVibes[21] program at 353.15 K.

Cartesian coordinates (in Å) and free energies (in a.u.) of all the stationary points discussed in the text. All calculations have been performed at the PCM(DCE)-B3LYP-D3/def2-TZVPP//PCM(DCE)-B3LYP-D3/def2-SVP level.

1a', G = -617.324042

```
C        -0.319067000     -0.357034000     -1.103023000
H        -0.420224000     -0.346120000     -2.204144000
H        -0.044782000     -1.396742000     -0.844940000
C        -1.645965000      0.025239000     -0.438970000
H        -1.905929000      1.063819000     -0.702925000
H        -1.529412000      0.000633000      0.657175000
C        -2.794656000     -0.911958000     -0.853524000
H        -2.524468000     -1.953416000     -0.600523000
H        -2.904943000     -0.889855000     -1.953003000
C        -4.076353000     -0.576328000     -0.228696000
C        -5.128081000     -0.279908000      0.300438000
C        -6.397802000      0.069957000      0.934801000
H        -6.277907000      0.918001000      1.629326000
H        -7.154893000      0.357762000      0.186515000
H        -6.804948000     -0.776991000      1.511772000
C         0.800973000      0.526972000     -0.714399000
C         1.321474000      1.512953000     -0.056824000
C         2.292314000      0.712289000     -0.905783000
H         2.694862000      1.125104000     -1.844952000
C         1.284544000      2.644689000      0.890980000
H         0.263160000      2.823574000      1.258673000
H         1.939416000      2.437772000      1.754081000
H         1.657591000      3.567104000      0.415648000
C         3.273515000     -0.225735000     -0.279399000
O         4.365803000     -0.487390000     -0.739168000
O         2.817916000     -0.764326000      0.870967000
C         3.692261000     -1.673652000      1.541314000
H         3.923109000     -2.540094000      0.902292000
H         4.637710000     -1.180031000      1.814619000
```

H	3.162980000	-2.001692000	2.444498000

Co(dppp)$^+$, G = -3110.05268

Co	0.079705000	-0.965814000	-1.005874000
P	-1.451586000	-0.205069000	0.264441000
P	1.636652000	-0.343948000	0.294050000
C	1.428660000	-0.888601000	2.072454000
H	1.987338000	-1.829799000	2.178089000
H	1.922430000	-0.146040000	2.717020000
C	-0.994847000	0.017994000	2.041620000
H	-1.910419000	0.033857000	2.650470000
H	-0.535252000	1.014641000	2.138086000
C	-0.033508000	-1.092203000	2.503427000
H	-0.393271000	-2.064384000	2.127607000
H	-0.063675000	-1.159149000	3.602588000
C	3.228758000	-1.101519000	-0.217713000
C	4.426105000	-0.393841000	-0.402950000
C	3.194792000	-2.483480000	-0.486464000
C	5.577184000	-1.066281000	-0.828099000
H	4.467551000	0.680788000	-0.215417000
C	4.346054000	-3.151626000	-0.907316000
H	2.252988000	-3.029899000	-0.370247000
C	5.541149000	-2.442176000	-1.077047000
H	6.507289000	-0.509321000	-0.965586000
H	4.309945000	-4.224897000	-1.109272000
H	6.443276000	-2.961852000	-1.408961000
C	1.922666000	1.469762000	0.393486000
C	2.748677000	2.069983000	1.360995000
C	1.263216000	2.283542000	-0.542047000
C	2.905665000	3.458176000	1.387882000
H	3.280911000	1.460655000	2.095445000
C	1.416012000	3.672196000	-0.510745000
H	0.614157000	1.814665000	-1.283776000
C	2.237182000	4.261134000	0.455487000
H	3.550996000	3.915442000	2.141840000
H	0.885778000	4.292181000	-1.237594000
H	2.356467000	5.346970000	0.485647000
C	-2.265127000	1.358032000	-0.248789000
C	-2.987397000	2.170702000	0.639918000
C	-2.155556000	1.737046000	-1.597555000
C	-3.586618000	3.347726000	0.181627000
H	-3.089353000	1.893657000	1.691500000
C	-2.763047000	2.908849000	-2.055251000
H	-1.582451000	1.109129000	-2.286587000
C	-3.476375000	3.717585000	-1.163730000
H	-4.143343000	3.978655000	0.878798000
H	-2.672723000	3.194816000	-3.105912000
H	-3.946265000	4.638754000	-1.516880000
C	-2.795548000	-1.459468000	0.235174000
C	-3.329150000	-2.092190000	1.368367000
C	-3.257961000	-1.833502000	-1.040211000
C	-4.315241000	-3.074903000	1.223989000
H	-2.987912000	-1.831287000	2.371616000
C	-4.244566000	-2.810412000	-1.181780000
H	-2.845477000	-1.345730000	-1.929022000
C	-4.775368000	-3.434571000	-0.046409000
H	-4.726044000	-3.560234000	2.112693000
H	-4.599305000	-3.085777000	-2.177914000
H	-5.546488000	-4.201323000	-0.152935000

INT0, G = -3727.408467

C	-1.382558000	-2.038617000	2.991436000
C	-0.739122000	-2.041117000	1.650824000
C	-0.971049000	1.184044000	2.989739000
C	-1.905372000	0.385588000	3.817719000
C	-2.433478000	-0.922033000	3.204400000
C	-0.338882000	-2.719687000	0.620027000
C	-0.214846000	-4.017753000	-0.081855000
H	-1.055157000	-4.151791000	-0.783811000
C	-0.428573000	2.295054000	2.601773000
Co	-0.111234000	-0.904112000	0.302952000
P	-1.680986000	-0.470945000	-1.132686000
C	0.266671000	1.039226000	2.140152000
H	0.217530000	0.812622000	1.045584000
H	-2.753071000	1.033310000	4.094319000
H	-1.379577000	0.147565000	4.761124000
H	-2.949147000	-0.712725000	2.254545000
H	-3.201330000	-1.306965000	3.893083000
H	-0.594847000	-1.954688000	3.761553000
H	-1.858149000	-3.021378000	3.152285000
H	0.711372000	-4.079382000	-0.672696000
H	-0.238733000	-4.857345000	0.632162000
P	1.493360000	-0.240642000	-1.170461000
C	0.980059000	-0.319841000	-2.955813000
H	0.484052000	0.621993000	-3.227871000
H	1.884822000	-0.390171000	-3.575542000
C	-1.398556000	-1.373268000	-2.729858000
H	-2.015876000	-0.882870000	-3.498036000
H	-1.848636000	-2.364343000	-2.571900000
C	0.065047000	-1.534475000	-3.187560000
H	0.054960000	-1.775146000	-4.262026000
H	0.504383000	-2.413400000	-2.687651000
C	2.154980000	1.453286000	-0.896974000
C	1.412565000	2.576279000	-1.303184000
C	3.319180000	1.650256000	-0.133197000
C	1.821546000	3.864159000	-0.947058000
H	0.503011000	2.461980000	-1.890909000
C	3.723950000	2.939321000	0.223003000
H	3.914505000	0.795759000	0.189187000
C	2.973701000	4.050198000	-0.176773000
H	1.228239000	4.722441000	-1.271125000
H	4.629991000	3.073802000	0.818843000
H	3.288241000	5.056983000	0.108387000
C	2.971484000	-1.335082000	-1.129370000
C	4.051649000	-1.108769000	-2.002101000
C	3.031424000	-2.411429000	-0.232838000
C	5.162148000	-1.954700000	-1.984053000
H	4.036623000	-0.262024000	-2.692949000
C	4.143251000	-3.260727000	-0.218112000
H	2.214080000	-2.571611000	0.471041000
C	5.207811000	-3.035916000	-1.094892000
H	5.996155000	-1.768655000	-2.664960000
H	4.175938000	-4.096299000	0.485092000
H	6.076585000	-3.698598000	-1.084086000
C	-3.403258000	-0.959666000	-0.681024000
C	-4.493537000	-0.144450000	-1.034659000
C	-3.651307000	-2.175524000	-0.020000000
C	-5.799953000	-0.537547000	-0.727885000
H	-4.332456000	0.803124000	-1.550433000
C	-4.957801000	-2.567113000	0.279703000

H	-2.825397000	-2.819045000	0.280259000
C	-6.036240000	-1.747413000	-0.069630000
H	-6.635248000	0.109594000	-1.006031000
H	-5.130825000	-3.513432000	0.797507000
H	-7.057596000	-2.050418000	0.173106000
C	-1.865057000	1.309437000	-1.539407000
C	-2.012381000	2.191114000	-0.454121000
C	-1.869954000	1.826788000	-2.844718000
C	-2.142879000	3.563790000	-0.667750000
H	-2.034486000	1.802757000	0.565278000
C	-1.987574000	3.205163000	-3.055764000
H	-1.780400000	1.171409000	-3.711890000
C	-2.119212000	4.075849000	-1.969994000
H	-2.260095000	4.235705000	0.184848000
H	-1.978479000	3.596422000	-4.075523000
H	-2.209391000	5.151444000	-2.138055000
C	-0.255628000	3.757703000	2.532203000
H	0.656594000	4.046917000	3.079901000
H	-0.117896000	4.077314000	1.487347000
H	-1.114451000	4.288129000	2.968388000
C	1.547801000	0.653985000	2.809377000
O	1.893551000	1.061520000	3.896279000
O	2.275667000	-0.217286000	2.086847000
C	3.528918000	-0.618936000	2.658863000
H	4.158553000	0.261014000	2.854588000
H	3.364326000	-1.157989000	3.603057000
H	4.005436000	-1.274119000	1.922698000

TS1, G = -3727.370599 (i = -252 cm-1)

C	-2.333837000	-2.686183000	2.094889000
C	-1.269700000	-2.306743000	1.139757000
C	-1.227001000	0.399326000	2.363476000
C	-2.032674000	-0.486758000	3.281832000
C	-3.037151000	-1.472976000	2.701165000
C	-0.476352000	-2.676342000	0.189635000
C	-0.078822000	-3.846011000	-0.632959000
H	-0.793792000	-3.987605000	-1.461747000
C	-0.762545000	1.603168000	2.566855000
Co	-0.324899000	-0.795772000	0.496972000
P	-1.695405000	-0.056067000	-1.046723000
C	0.244734000	1.092969000	1.609496000
H	0.308185000	1.633264000	0.661270000
H	-2.551155000	0.228563000	3.943627000
H	-1.312051000	-1.028097000	3.921611000
H	-3.658302000	-0.971007000	1.945408000
H	-3.714545000	-1.806503000	3.502720000
H	-1.867513000	-3.282443000	2.900887000
H	-3.057184000	-3.353373000	1.595935000
H	0.926272000	-3.756270000	-1.064099000
H	-0.113155000	-4.760871000	-0.019015000
P	1.530857000	-0.447229000	-0.926461000
C	1.082363000	-0.342132000	-2.724610000
H	0.806255000	0.703043000	-2.915947000
H	1.986597000	-0.520665000	-3.323030000
C	-1.463992000	-0.984387000	-2.639490000
H	-2.036971000	-0.428832000	-3.396644000
H	-2.012711000	-1.923690000	-2.492281000
C	-0.035012000	-1.308657000	-3.130098000
H	-0.068399000	-1.358439000	-4.229586000
H	0.240231000	-2.319182000	-2.799678000

C	2.607866000	1.037604000	-0.746651000
C	2.064023000	2.310624000	-1.007408000
C	3.938375000	0.945839000	-0.305700000
C	2.832623000	3.461669000	-0.823986000
H	1.036968000	2.419490000	-1.360038000
C	4.706924000	2.100597000	-0.131788000
H	4.381849000	-0.029066000	-0.096263000
C	4.155927000	3.360481000	-0.382900000
H	2.391208000	4.440343000	-1.027071000
H	5.741390000	2.012541000	0.209013000
H	4.756924000	4.261429000	-0.237854000
C	2.697404000	-1.860571000	-0.780542000
C	3.354144000	-2.435409000	-1.880230000
C	2.919391000	-2.390879000	0.503901000
C	4.209330000	-3.527715000	-1.700774000
H	3.207673000	-2.045560000	-2.888455000
C	3.784194000	-3.473304000	0.680476000
H	2.418086000	-1.953659000	1.369607000
C	4.425468000	-4.049342000	-0.421818000
H	4.707374000	-3.970763000	-2.566566000
H	3.950588000	-3.873379000	1.683573000
H	5.092611000	-4.903812000	-0.284256000
C	-3.482212000	-0.357904000	-0.704450000
C	-4.390447000	0.683361000	-0.455106000
C	-3.942588000	-1.687061000	-0.660016000
C	-5.726256000	0.397816000	-0.152518000
H	-4.071043000	1.724547000	-0.496845000
C	-5.277859000	-1.967580000	-0.370235000
H	-3.254860000	-2.518250000	-0.825204000
C	-6.173684000	-0.924883000	-0.107788000
H	-6.419243000	1.219280000	0.043964000
H	-5.616259000	-3.005773000	-0.338518000
H	-7.217402000	-1.144447000	0.128984000
C	-1.584489000	1.728084000	-1.485338000
C	-1.715934000	2.687901000	-0.463710000
C	-1.331837000	2.171694000	-2.795911000
C	-1.568235000	4.048730000	-0.737830000
H	-1.939983000	2.372836000	0.555646000
C	-1.176770000	3.534716000	-3.066571000
H	-1.244996000	1.467899000	-3.623616000
C	-1.285543000	4.476117000	-2.039123000
H	-1.670477000	4.774919000	0.071727000
H	-0.969095000	3.858379000	-4.088913000
H	-1.157515000	5.539651000	-2.253061000
C	-0.843944000	2.780453000	3.459803000
H	0.086440000	2.869992000	4.045283000
H	-0.937919000	3.709319000	2.872709000
H	-1.694920000	2.706793000	4.152090000
C	1.564374000	0.839154000	2.288943000
O	1.901079000	-0.213997000	2.789900000
O	2.291471000	1.955964000	2.355603000
C	3.542528000	1.874884000	3.055210000
H	3.369847000	1.653043000	4.118890000
H	4.175097000	1.090073000	2.618709000
H	4.019299000	2.853742000	2.935221000

INT1, G = -3727.419892

C	-1.563952000	-3.718826000	0.471467000
C	-0.771570000	-2.780090000	-0.360251000
C	-0.672827000	-1.276629000	1.582659000

C	-1.844344000	-1.885633000	2.284406000
C	-2.510334000	-3.041285000	1.476002000
C	0.028095000	-2.491839000	-1.307250000
C	0.772657000	-2.963119000	-2.498497000
H	0.497610000	-2.396146000	-3.401281000
C	0.691264000	-1.457603000	2.121560000
Co	-0.251769000	-0.892593000	-0.101481000
P	-1.956234000	0.376881000	-0.547302000
C	1.548773000	-0.391945000	2.103477000
H	1.161671000	0.598399000	1.870440000
H	-2.601115000	-1.097923000	2.436833000
H	-1.558833000	-2.233887000	3.289566000
H	-3.375946000	-2.649169000	0.933641000
H	-2.898298000	-3.795685000	2.176566000
H	-0.846863000	-4.369109000	1.001606000
H	-2.143316000	-4.379733000	-0.194716000
H	1.857597000	-2.841714000	-2.363958000
H	0.561977000	-4.027892000	-2.687790000
P	1.104560000	0.394986000	-1.387335000
C	0.458338000	0.599541000	-3.121572000
H	0.623360000	1.639177000	-3.441520000
H	1.087953000	-0.033376000	-3.763907000
C	-1.993619000	0.924047000	-2.350956000
H	-1.818671000	2.008370000	-2.372582000
H	-3.026242000	0.770027000	-2.692906000
C	-1.017372000	0.203965000	-3.287486000
H	-1.312000000	0.430426000	-4.324894000
H	-1.127810000	-0.884795000	-3.170392000
C	1.371357000	2.111241000	-0.760511000
C	0.530095000	3.179220000	-1.114085000
C	2.384687000	2.338350000	0.186535000
C	0.685809000	4.435435000	-0.522725000
H	-0.260993000	3.050621000	-1.850945000
C	2.534852000	3.591919000	0.783165000
H	3.068322000	1.540312000	0.471216000
C	1.682813000	4.644527000	0.434314000
H	0.014818000	5.248974000	-0.807010000
H	3.321606000	3.736599000	1.527027000
H	1.796535000	5.624340000	0.904244000
C	2.823383000	-0.224207000	-1.564919000
C	3.713787000	0.394549000	-2.460086000
C	3.272974000	-1.288886000	-0.771370000
C	5.031653000	-0.055643000	-2.561126000
H	3.384864000	1.237569000	-3.073033000
C	4.595549000	-1.733045000	-0.868286000
H	2.590467000	-1.774624000	-0.073630000
C	5.475016000	-1.118775000	-1.764015000
H	5.717758000	0.428682000	-3.260014000
H	4.934168000	-2.561279000	-0.242294000
H	6.508176000	-1.465756000	-1.841925000
C	-3.582496000	-0.456284000	-0.388381000
C	-4.477985000	-0.145464000	0.645930000
C	-3.913244000	-1.465508000	-1.311500000
C	-5.684988000	-0.843964000	0.760562000
H	-4.239756000	0.641651000	1.363868000
C	-5.123509000	-2.151295000	-1.199844000
H	-3.218850000	-1.733925000	-2.111138000
C	-6.009298000	-1.845773000	-0.158874000
H	-6.374524000	-0.599710000	1.572007000
H	-5.371923000	-2.933345000	-1.920997000
H	-6.952883000	-2.388683000	-0.066644000

C	-2.138698000	1.933516000	0.403201000
C	-1.404146000	2.133039000	1.579629000
C	-2.999197000	2.949361000	-0.052569000
C	-1.508734000	3.334881000	2.284862000
H	-0.743149000	1.344574000	1.938913000
C	-3.100624000	4.150002000	0.652333000
H	-3.597812000	2.809958000	-0.955823000
C	-2.350774000	4.347422000	1.818342000
H	-0.921094000	3.481073000	3.193808000
H	-3.768234000	4.935191000	0.289723000
H	-2.426305000	5.291815000	2.362669000
C	1.081409000	-2.848343000	2.574544000
H	0.211397000	-3.401466000	2.949951000
H	1.486786000	-3.410214000	1.716798000
H	1.854670000	-2.828002000	3.349580000
C	2.987050000	-0.352624000	2.456927000
O	3.578189000	0.686842000	2.677253000
O	3.595773000	-1.548865000	2.457201000
C	5.002335000	-1.551441000	2.731615000
H	5.541365000	-0.985359000	1.957383000
H	5.208495000	-1.101323000	3.713514000
H	5.311096000	-2.603173000	2.718575000

TS2, G = -3727.412703 (i = -174 cm-1)

C	-1.571282000	-3.150577000	2.186824000
C	-0.790598000	-2.475425000	1.118415000
C	-0.966696000	-0.585270000	1.872750000
C	-2.192883000	-0.820998000	2.722942000
C	-2.764513000	-2.235980000	2.497067000
C	-0.339988000	-2.529291000	-0.082349000
C	0.025192000	-3.535382000	-1.109566000
H	-0.414459000	-3.302752000	-2.090373000
C	0.337743000	-0.199670000	2.493653000
Co	-0.232795000	-0.594352000	0.190321000
P	-1.867320000	0.360107000	-0.879638000
C	0.949052000	0.930936000	2.020213000
H	0.378408000	1.665081000	1.452402000
H	-2.954691000	-0.062440000	2.476647000
H	-1.940887000	-0.674660000	3.786035000
H	-3.447529000	-2.230729000	1.636167000
H	-3.332019000	-2.586436000	3.372096000
H	-0.957065000	-3.301093000	3.090037000
H	-1.885214000	-4.142098000	1.822884000
H	1.116870000	-3.571900000	-1.247116000
H	-0.319067000	-4.536378000	-0.804157000
P	1.366695000	-0.286616000	-1.340263000
C	0.807426000	-0.369054000	-3.122113000
H	1.126885000	0.565732000	-3.607495000
H	1.359648000	-1.187997000	-3.604965000
C	-1.612552000	0.478431000	-2.725919000
H	-1.198389000	1.477788000	-2.932484000
H	-2.608580000	0.438071000	-3.190630000
C	-0.697505000	-0.590484000	-3.336768000
H	-0.870565000	-0.597755000	-4.424919000
H	-0.987060000	-1.592258000	-2.988929000
C	2.128530000	1.384701000	-1.160200000
C	1.286099000	2.510469000	-1.127691000
C	3.511621000	1.565783000	-0.999329000
C	1.814671000	3.789791000	-0.951794000
H	0.204836000	2.398902000	-1.215982000

C	4.037679000	2.849586000	-0.821342000
H	4.185156000	0.707729000	-1.002164000
C	3.194254000	3.962586000	-0.797678000
H	1.142792000	4.650781000	-0.922111000
H	5.115584000	2.975530000	-0.693534000
H	3.608808000	4.962403000	-0.648978000
C	2.800913000	-1.419817000	-1.181056000
C	3.642942000	-1.761308000	-2.251828000
C	3.088585000	-1.911618000	0.103228000
C	4.745702000	-2.594772000	-2.040421000
H	3.456468000	-1.372853000	-3.255287000
C	4.195838000	-2.735952000	0.314109000
H	2.453133000	-1.636083000	0.945382000
C	5.023448000	-3.083428000	-0.759194000
H	5.393336000	-2.857806000	-2.880161000
H	4.409092000	-3.109123000	1.318567000
H	5.886816000	-3.733365000	-0.597408000
C	-3.423367000	-0.598404000	-0.686047000
C	-4.520813000	-0.089329000	0.027207000
C	-3.486926000	-1.907799000	-1.196951000
C	-5.658836000	-0.878484000	0.226569000
H	-4.494392000	0.923520000	0.432548000
C	-4.629154000	-2.687324000	-1.006727000
H	-2.640483000	-2.332960000	-1.736321000
C	-5.716888000	-2.176337000	-0.289149000
H	-6.503934000	-0.472432000	0.787491000
H	-4.665067000	-3.701290000	-1.411945000
H	-6.606763000	-2.790295000	-0.131637000
C	-2.337783000	2.075400000	-0.402569000
C	-2.131543000	2.512697000	0.915504000
C	-2.934708000	2.955453000	-1.324541000
C	-2.496264000	3.803831000	1.305617000
H	-1.687324000	1.835543000	1.644177000
C	-3.293040000	4.248715000	-0.934531000
H	-3.135168000	2.644590000	-2.350904000
C	-3.071274000	4.677715000	0.378406000
H	-2.323888000	4.126257000	2.334937000
H	-3.751242000	4.922820000	-1.662070000
H	-3.349024000	5.691040000	0.678045000
C	0.947074000	-1.136998000	3.506038000
H	0.195996000	-1.398838000	4.267506000
H	1.250608000	-2.079036000	3.020168000
H	1.823632000	-0.707418000	4.002654000
C	2.335959000	1.399454000	2.271180000
O	2.640779000	2.571027000	2.318158000
O	3.224565000	0.397573000	2.378259000
C	4.598218000	0.768071000	2.543629000
H	4.924814000	1.399047000	1.704106000
H	4.741658000	1.320493000	3.484036000
H	5.164366000	-0.170525000	2.558669000

INT2, G = -3727.438736

C	-2.967628000	-2.726702000	1.925579000
C	-1.795787000	-2.099998000	1.225758000
C	-1.411947000	-0.821262000	1.901900000
C	-2.669875000	-0.305805000	2.611695000
C	-3.710936000	-1.468404000	2.471765000
C	-1.111417000	-2.118177000	0.078595000
C	-1.071563000	-3.071347000	-1.064470000
H	-1.508235000	-2.649523000	-1.985246000

C	-0.056734000	-0.835683000	2.500416000
Co	-0.311687000	-0.313962000	0.307630000
P	-1.772671000	0.509964000	-1.067902000
C	0.670402000	0.332028000	2.362052000
H	0.122851000	1.260024000	2.159899000
H	-3.082140000	0.615342000	2.173469000
H	-2.441254000	-0.081713000	3.665408000
H	-4.488354000	-1.170668000	1.755789000
H	-4.211701000	-1.677332000	3.428801000
H	-2.619707000	-3.360521000	2.760707000
H	-3.599691000	-3.347092000	1.272360000
H	-0.038790000	-3.361876000	-1.314104000
H	-1.637925000	-3.985716000	-0.816838000
P	1.452760000	-0.143442000	-1.159677000
C	1.017420000	0.369112000	-2.883500000
H	0.897373000	1.461697000	-2.856043000
H	1.883709000	0.177386000	-3.533854000
C	-1.593272000	0.184676000	-2.888332000
H	-1.891315000	1.114132000	-3.397408000
H	-2.378888000	-0.544273000	-3.132876000
C	-0.241796000	-0.333375000	-3.413212000
H	-0.263308000	-0.237523000	-4.510265000
H	-0.164616000	-1.412604000	-3.212759000
C	2.784928000	1.032839000	-0.706730000
C	2.414585000	2.273257000	-0.159844000
C	4.144177000	0.746417000	-0.914622000
C	3.389865000	3.217340000	0.167900000
H	1.363015000	2.504821000	0.023430000
C	5.117217000	1.692729000	-0.579830000
H	4.445042000	-0.217048000	-1.332142000
C	4.742314000	2.928555000	-0.040092000
H	3.089482000	4.174109000	0.600708000
H	6.173298000	1.461666000	-0.738950000
H	5.506032000	3.663606000	0.225009000
C	2.253617000	-1.784545000	-1.309767000
C	2.516138000	-2.416882000	-2.535446000
C	2.546901000	-2.460939000	-0.110918000
C	3.056766000	-3.707496000	-2.559147000
H	2.301830000	-1.921391000	-3.483412000
C	3.093884000	-3.744243000	-0.139114000
H	2.354462000	-1.976041000	0.846872000
C	3.343606000	-4.374117000	-1.364473000
H	3.252859000	-4.191683000	-3.518776000
H	3.318480000	-4.257032000	0.799237000
H	3.761567000	-5.383392000	-1.387507000
C	-3.564053000	0.269327000	-0.768467000
C	-4.384192000	1.285943000	-0.252070000
C	-4.126573000	-0.991690000	-1.043755000
C	-5.740564000	1.042184000	-0.012891000
H	-3.977099000	2.275375000	-0.040326000
C	-5.484115000	-1.224600000	-0.817560000
H	-3.511510000	-1.804370000	-1.431120000
C	-6.294177000	-0.209515000	-0.296972000
H	-6.367001000	1.841273000	0.390205000
H	-5.906397000	-2.207453000	-1.038465000
H	-7.354965000	-0.394663000	-0.112701000
C	-1.397035000	2.283859000	-0.781115000
C	-1.427064000	2.742779000	0.552426000
C	-0.958611000	3.160540000	-1.788295000
C	-1.009757000	4.036108000	0.871517000
H	-1.780565000	2.084479000	1.347641000

C	-0.540175000	4.454456000	-1.464898000
H	-0.935819000	2.851231000	-2.833478000
C	-0.555194000	4.892246000	-0.136959000
H	-1.034392000	4.370714000	1.910739000
H	-0.196615000	5.121661000	-2.258433000
H	-0.216956000	5.900732000	0.111249000
C	0.513323000	-2.122440000	3.040724000
H	-0.159865000	-2.483140000	3.835429000
H	0.528288000	-2.898912000	2.264012000
H	1.522983000	-2.002964000	3.445862000
C	2.089030000	0.621621000	2.695844000
O	2.455818000	1.713546000	3.071485000
O	2.923218000	-0.406162000	2.482154000
C	4.321626000	-0.159138000	2.690926000
H	4.663395000	0.651089000	2.031775000
H	4.512801000	0.115690000	3.738196000
H	4.831559000	-1.095977000	2.438779000

INT3, G = -3727.476637

C	-3.822696000	-2.816343000	-0.179122000
C	-2.601338000	-1.980183000	0.169221000
C	-2.913802000	-1.144543000	1.326557000
C	-4.396117000	-1.253934000	1.607859000
C	-4.759428000	-2.640238000	1.033874000
C	-1.448602000	-1.888064000	-0.537920000
C	-1.093064000	-2.768856000	-1.706178000
H	-1.315299000	-3.833042000	-1.516055000
C	-1.973674000	-0.424058000	2.003802000
Co	0.046438000	-0.784230000	-0.198796000
P	-0.954887000	0.971282000	-1.071317000
C	-0.549875000	-0.653025000	1.698312000
H	0.126366000	0.035763000	2.225763000
H	-4.931166000	-0.459933000	1.057069000
H	-4.653131000	-1.145236000	2.671722000
H	-5.824787000	-2.730380000	0.773802000
H	-4.535767000	-3.414442000	1.787062000
H	-3.573782000	-3.869340000	-0.389443000
H	-4.287039000	-2.411570000	-1.096671000
H	-1.659096000	-2.462904000	-2.604519000
H	-0.023742000	-2.702578000	-1.987461000
P	2.195388000	0.158963000	-0.625444000
C	2.108667000	1.249460000	-2.121159000
H	1.845343000	2.261937000	-1.779780000
H	3.101814000	1.318583000	-2.587851000
C	-0.398844000	1.128374000	-2.844373000
H	-0.574821000	2.169584000	-3.152214000
H	-1.070945000	0.501929000	-3.447508000
C	1.057390000	0.711835000	-3.117756000
H	1.318194000	1.056294000	-4.130672000
H	1.107880000	-0.389631000	-3.157501000
C	3.110381000	1.084955000	0.661028000
C	3.880629000	2.222848000	0.373129000
C	2.992457000	0.652558000	1.993892000
C	4.515603000	2.919256000	1.405776000
H	3.989935000	2.578405000	-0.653061000
C	3.634342000	1.346275000	3.022408000
H	2.389599000	-0.226638000	2.231831000
C	4.393014000	2.485015000	2.729798000
H	5.108947000	3.806726000	1.172893000
H	3.534746000	1.001122000	4.054160000

H	4.888427000	3.034290000	3.533946000
C	3.332231000	-1.211879000	-1.107086000
C	3.745561000	-1.432128000	-2.432136000
C	3.754652000	-2.107368000	-0.106924000
C	4.566504000	-2.521132000	-2.746686000
H	3.441845000	-0.763528000	-3.238310000
C	4.580849000	-3.186881000	-0.423432000
H	3.434357000	-1.969235000	0.926092000
C	4.987550000	-3.399816000	-1.745599000
H	4.879271000	-2.676577000	-3.782124000
H	4.904584000	-3.868222000	0.367203000
H	5.630145000	-4.248172000	-1.993277000
C	-2.786202000	1.057811000	-1.167011000
C	-3.509426000	1.852733000	-0.263362000
C	-3.487426000	0.283305000	-2.106704000
C	-4.906240000	1.891355000	-0.316105000
H	-2.986853000	2.448617000	0.486261000
C	-4.882445000	0.324009000	-2.158083000
H	-2.954799000	-0.369407000	-2.801005000
C	-5.595785000	1.131480000	-1.265240000
H	-5.454960000	2.516229000	0.392360000
H	-5.413073000	-0.281124000	-2.896811000
H	-6.687154000	1.162339000	-1.305799000
C	-0.497495000	2.594970000	-0.327632000
C	0.211653000	2.656284000	0.878963000
C	-0.856689000	3.796520000	-0.967699000
C	0.579109000	3.887208000	1.431769000
H	0.494989000	1.739288000	1.385171000
C	-0.492614000	5.025446000	-0.415648000
H	-1.433796000	3.780808000	-1.895172000
C	0.230450000	5.073718000	0.783671000
H	1.146161000	3.909073000	2.364993000
H	-0.775293000	5.950702000	-0.923266000
H	0.518425000	6.037679000	1.209966000
C	-2.302160000	0.602970000	3.054190000
H	-3.381047000	0.793116000	3.131641000
H	-1.929709000	0.296194000	4.047474000
H	-1.808342000	1.562034000	2.817853000
C	-0.005780000	-2.034674000	1.786083000
O	0.865058000	-2.337521000	0.946231000
O	-0.475977000	-2.880496000	2.665264000
C	-0.029654000	-4.251275000	2.582253000
H	-0.305621000	-4.669842000	1.603996000
H	1.059770000	-4.306686000	2.713522000
H	-0.544676000	-4.781473000	3.390277000

TS3, G = -3727.440277 (i = -376 cm-1)

C	-3.579727000	-2.914340000	-0.931895000
C	-2.550190000	-2.062567000	-0.238971000
C	-3.174884000	-1.297392000	0.811320000
C	-4.668114000	-1.462867000	0.752251000
C	-4.819693000	-2.811598000	-0.003112000
C	-1.202693000	-1.909428000	-0.399287000
C	-0.195195000	-2.839658000	-1.004922000
H	-0.056097000	-3.763896000	-0.423390000
C	-2.296284000	-0.722290000	1.674143000
Co	0.137318000	-0.607427000	-0.443110000
P	-0.787427000	1.272035000	-0.992210000
C	-0.880177000	-1.084221000	1.379707000
H	-0.173447000	-0.283260000	1.651198000

H	-5.113764000	-0.638953000	0.172153000
H	-5.148119000	-1.466754000	1.741996000
H	-5.768539000	-2.887298000	-0.553744000
H	-4.792707000	-3.638478000	0.725073000
H	-3.243695000	-3.948535000	-1.107042000
H	-3.789609000	-2.475977000	-1.924560000
H	-0.437844000	-3.085066000	-2.048955000
H	0.828049000	-2.374971000	-1.038972000
P	2.204142000	0.230023000	-0.752143000
C	2.280282000	1.591626000	-1.997767000
H	2.075212000	2.535248000	-1.468687000
H	3.293138000	1.675391000	-2.416057000
C	-0.201104000	1.764686000	-2.707165000
H	-0.305744000	2.857681000	-2.778098000
H	-0.917593000	1.333431000	-3.419713000
C	1.224966000	1.335977000	-3.089428000
H	1.507257000	1.882222000	-4.003326000
H	1.227862000	0.266487000	-3.361183000
C	3.059243000	0.853030000	0.750238000
C	3.904925000	1.973272000	0.722494000
C	2.827740000	0.193356000	1.970625000
C	4.499023000	2.434451000	1.901461000
H	4.107446000	2.500313000	-0.211647000
C	3.431189000	0.652612000	3.144107000
H	2.170271000	-0.679034000	2.009710000
C	4.262568000	1.777862000	3.113356000
H	5.149416000	3.312000000	1.870472000
H	3.244016000	0.133119000	4.087049000
H	4.726405000	2.142385000	4.033157000
C	3.321439000	-1.098097000	-1.375352000
C	3.996436000	-1.035328000	-2.603794000
C	3.447620000	-2.253501000	-0.579137000
C	4.794913000	-2.105947000	-3.023625000
H	3.909356000	-0.159731000	-3.249226000
C	4.251615000	-3.314601000	-0.999098000
H	2.903580000	-2.334325000	0.366299000
C	4.928086000	-3.243413000	-2.223032000
H	5.315211000	-2.045773000	-3.982703000
H	4.345458000	-4.203273000	-0.370220000
H	5.553901000	-4.076082000	-2.553164000
C	-2.618102000	1.327980000	-1.181158000
C	-3.436312000	2.085810000	-0.329081000
C	-3.217284000	0.562246000	-2.197441000
C	-4.822689000	2.111306000	-0.518313000
H	-2.998414000	2.669560000	0.482155000
C	-4.599555000	0.594541000	-2.389229000
H	-2.605208000	-0.071667000	-2.843655000
C	-5.406118000	1.377988000	-1.554908000
H	-5.446290000	2.710645000	0.149259000
H	-5.048959000	0.001973000	-3.189551000
H	-6.487942000	1.404283000	-1.705143000
C	-0.404247000	2.745150000	0.047229000
C	0.228720000	2.568528000	1.286260000
C	-0.726075000	4.050177000	-0.370970000
C	0.553094000	3.667479000	2.088109000
H	0.493804000	1.569369000	1.626656000
C	-0.405875000	5.147628000	0.430142000
H	-1.240559000	4.216918000	-1.320261000
C	0.238607000	4.959077000	1.659795000
H	1.060782000	3.506683000	3.041767000
H	-0.660306000	6.155493000	0.093480000

H	0.493463000	5.820950000	2.281241000
C	-2.620202000	0.175983000	2.823634000
H	-3.702485000	0.344167000	2.913815000
H	-2.245912000	-0.247809000	3.771673000
H	-2.130459000	1.157901000	2.701268000
C	-0.325510000	-2.327640000	2.022481000
O	0.847156000	-2.648804000	1.933826000
O	-1.227765000	-3.039607000	2.698541000
C	-0.768711000	-4.258491000	3.300310000
H	-0.410375000	-4.956849000	2.529264000
H	0.049237000	-4.057149000	4.007317000
H	-1.633609000	-4.680717000	3.824758000

INT4, G = -3727.480005

C	-1.911643000	-1.657691000	-2.555807000
C	-1.812018000	-1.528470000	-1.054269000
C	-3.131375000	-1.200152000	-0.500542000
C	-4.101518000	-0.954695000	-1.617672000
C	-3.448024000	-1.739628000	-2.791043000
C	-0.994710000	-1.976452000	-0.022616000
C	0.309805000	-2.650271000	-0.221695000
H	0.763908000	-3.011631000	0.707898000
C	-3.164838000	-1.395663000	0.836136000
Co	0.144794000	-0.498346000	-0.671408000
P	-0.594308000	1.570419000	-0.783277000
C	-1.784233000	-1.845757000	1.288974000
H	-1.310698000	-1.087460000	1.935783000
H	-4.154686000	0.118618000	-1.855267000
H	-5.123638000	-1.291618000	-1.390952000
H	-3.742247000	-1.353820000	-3.778189000
H	-3.761283000	-2.795112000	-2.739284000
H	-1.355839000	-2.519939000	-2.955272000
H	-1.497107000	-0.757347000	-3.043014000
H	0.276480000	-3.452684000	-0.974818000
H	1.095945000	-1.949091000	-0.684396000
P	2.249570000	0.271285000	-0.631016000
C	2.529478000	1.888513000	-1.495615000
H	2.410930000	2.692535000	-0.754104000
H	3.569892000	1.920626000	-1.849532000
C	0.105925000	2.447101000	-2.265869000
H	0.011782000	3.530675000	-2.101605000
H	-0.557898000	2.202099000	-3.105990000
C	1.541591000	2.062873000	-2.661927000
H	1.926195000	2.841051000	-3.339789000
H	1.502878000	1.136215000	-3.256496000
C	2.941034000	0.455252000	1.058180000
C	3.938813000	1.397115000	1.354918000
C	2.453076000	-0.375591000	2.081460000
C	4.427601000	1.513494000	2.659495000
H	4.341710000	2.047976000	0.576312000
C	2.953540000	-0.267148000	3.380391000
H	1.660797000	-1.096711000	1.871747000
C	3.937444000	0.682955000	3.672891000
H	5.197117000	2.256095000	2.883612000
H	2.563082000	-0.917459000	4.166623000
H	4.321566000	0.777951000	4.691388000
C	3.419597000	-0.899228000	-1.455703000
C	3.735893000	-0.782143000	-2.820537000
C	3.932737000	-1.989900000	-0.730887000
C	4.553286000	-1.731785000	-3.441815000

H	3.354882000	0.047807000	-3.417291000
C	4.746841000	-2.937954000	-1.356101000
H	3.711575000	-2.100020000	0.332772000
C	5.059110000	-2.812752000	-2.713651000
H	4.794542000	-1.622535000	-4.501931000
H	5.142834000	-3.774802000	-0.775710000
H	5.696881000	-3.553579000	-3.201677000
C	-2.380410000	2.030208000	-0.834278000
C	-3.124547000	1.895980000	0.351423000
C	-3.027126000	2.504553000	-1.987672000
C	-4.481290000	2.219215000	0.382128000
H	-2.640178000	1.540366000	1.262554000
C	-4.385399000	2.840741000	-1.952144000
H	-2.489353000	2.622020000	-2.928590000
C	-5.116828000	2.694217000	-0.771011000
H	-5.043730000	2.100755000	1.311094000
H	-4.870935000	3.213700000	-2.857055000
H	-6.178437000	2.951395000	-0.746652000
C	-0.031118000	2.551532000	0.679439000
C	0.124420000	1.879863000	1.901853000
C	0.221604000	3.932841000	0.627052000
C	0.535624000	2.564266000	3.048298000
H	-0.056587000	0.805894000	1.948217000
C	0.641294000	4.616529000	1.770984000
H	0.094884000	4.491214000	-0.302416000
C	0.801967000	3.934013000	2.982928000
H	0.666243000	2.018512000	3.985037000
H	0.843583000	5.688877000	1.715084000
H	1.136307000	4.471527000	3.873562000
C	-4.298393000	-1.188568000	1.783612000
H	-5.215256000	-0.902058000	1.248852000
H	-4.499505000	-2.100303000	2.369649000
H	-4.067617000	-0.385434000	2.506439000
C	-1.678986000	-3.145586000	2.074172000
O	-0.716007000	-3.426238000	2.753592000
O	-2.718773000	-3.958897000	1.883268000
C	-2.668629000	-5.235963000	2.538222000
H	-1.794894000	-5.809386000	2.194933000
H	-2.605260000	-5.106967000	3.628625000
H	-3.596565000	-5.752282000	2.267180000

^{3}[Co(dppp)$^{+}$], G = -3110.103972

Co	0.121173000	-1.215670000	-1.247196000
P	-1.554871000	-0.428418000	0.064217000
P	1.813229000	-0.535675000	0.102928000
C	1.482551000	-1.228373000	1.812390000
H	2.060784000	-2.163579000	1.865278000
H	1.922671000	-0.548263000	2.557178000
C	-0.981276000	-0.372780000	1.841244000
H	-1.863171000	-0.434145000	2.495922000
H	-0.510102000	0.606781000	2.015748000
C	0.006094000	-1.517817000	2.141626000
H	-0.317206000	-2.431271000	1.611609000
H	-0.050042000	-1.763154000	3.214541000
C	3.596200000	-0.921437000	-0.127890000
C	4.630624000	0.019648000	-0.008547000
C	3.914436000	-2.248584000	-0.471054000
C	5.960349000	-0.366226000	-0.213275000
H	4.405592000	1.057481000	0.244760000
C	5.242582000	-2.633732000	-0.664766000

H	3.113594000	-2.985100000	-0.592962000
C	6.269773000	-1.690845000	-0.536358000
H	6.757905000	0.374826000	-0.117319000
H	5.476353000	-3.669156000	-0.924840000
H	7.309375000	-1.988585000	-0.693621000
C	1.719621000	1.293578000	0.312015000
C	2.142655000	1.963174000	1.474356000
C	1.141465000	2.043685000	-0.725901000
C	1.982976000	3.346535000	1.593223000
H	2.600322000	1.411850000	2.298580000
C	0.979442000	3.426216000	-0.607414000
H	0.791935000	1.545072000	-1.632691000
C	1.396402000	4.079857000	0.555203000
H	2.313736000	3.852740000	2.503373000
H	0.506016000	3.985703000	-1.417096000
H	1.260940000	5.159436000	0.656116000
C	-2.133561000	1.272284000	-0.305202000
C	-2.497651000	2.209379000	0.674707000
C	-2.159613000	1.650069000	-1.658459000
C	-2.880915000	3.501910000	0.302074000
H	-2.477661000	1.945692000	1.734189000
C	-2.551095000	2.937501000	-2.030240000
H	-1.850275000	0.929206000	-2.421630000
C	-2.909254000	3.867791000	-1.047564000
H	-3.156006000	4.227123000	1.071784000
H	-2.564666000	3.219814000	-3.085783000
H	-3.205503000	4.879894000	-1.333864000
C	-3.092408000	-1.434909000	0.144469000
C	-4.216561000	-1.049792000	0.895292000
C	-3.126995000	-2.637287000	-0.577421000
C	-5.351173000	-1.863243000	0.927267000
H	-4.212191000	-0.107947000	1.450761000
C	-4.262755000	-3.453747000	-0.543436000
H	-2.256330000	-2.927162000	-1.172960000
C	-5.374740000	-3.067037000	0.210161000
H	-6.222708000	-1.557242000	1.511313000
H	-4.278981000	-4.389430000	-1.107720000
H	-6.264473000	-3.701015000	0.237450000

^{3}INT0, G = -3727.43309

C	-0.468668000	-0.139373000	3.596292000
C	-0.513759000	-1.336574000	2.742012000
C	-0.074050000	2.756850000	2.400604000
C	-1.067477000	2.360772000	3.432802000
C	-1.549767000	0.913131000	3.240137000
C	-0.593330000	-2.453838000	2.211619000
C	-0.792300000	-3.877203000	1.897060000
H	-1.681578000	-4.008778000	1.259910000
C	0.548896000	3.561630000	1.597495000
Co	-0.331442000	-1.126917000	0.621880000
P	-2.111063000	-0.746365000	-0.815857000
C	0.982361000	2.119713000	1.515013000
H	0.674352000	1.496630000	0.663757000
H	-1.920264000	3.057643000	3.399309000
H	-0.616378000	2.470942000	4.435888000
H	-1.859382000	0.771125000	2.193171000
H	-2.449850000	0.734785000	3.846688000
H	0.536810000	0.294184000	3.496978000
H	-0.565052000	-0.450258000	4.650162000
H	0.071514000	-4.293440000	1.356150000

H	-0.936849000	-4.457089000	2.822465000
P	1.277493000	-0.894483000	-1.114466000
C	0.496753000	-1.423421000	-2.727859000
H	0.066278000	-0.536290000	-3.215381000
H	1.293148000	-1.792483000	-3.390163000
C	-1.999500000	-1.994141000	-2.196547000
H	-2.479052000	-1.592784000	-3.102410000
H	-2.626222000	-2.836900000	-1.866712000
C	-0.578082000	-2.506752000	-2.500591000
H	-0.640195000	-3.140339000	-3.399651000
H	-0.254160000	-3.181489000	-1.687070000
C	2.095835000	0.710352000	-1.483107000
C	1.380869000	1.732593000	-2.131782000
C	3.381501000	0.992145000	-0.988812000
C	1.936171000	3.006687000	-2.275147000
H	0.376077000	1.554986000	-2.513627000
C	3.930845000	2.269206000	-1.126749000
H	3.956023000	0.215686000	-0.483093000
C	3.208808000	3.282681000	-1.765994000
H	1.359882000	3.787104000	-2.777745000
H	4.927865000	2.472414000	-0.728353000
H	3.636951000	4.282865000	-1.866825000
C	2.658167000	-2.090053000	-0.910701000
C	3.653595000	-2.260751000	-1.889263000
C	2.709573000	-2.862496000	0.259671000
C	4.678788000	-3.188742000	-1.695908000
H	3.637447000	-1.657786000	-2.801073000
C	3.735383000	-3.794477000	0.452379000
H	1.950974000	-2.719857000	1.032671000
C	4.720435000	-3.957837000	-0.525467000
H	5.450719000	-3.311847000	-2.459476000
H	3.764849000	-4.388234000	1.369083000
H	5.524600000	-4.682676000	-0.376963000
C	-3.841446000	-0.912434000	-0.223671000
C	-4.942971000	-0.504946000	-0.995877000
C	-4.057314000	-1.476566000	1.044077000
C	-6.241336000	-0.672944000	-0.508805000
H	-4.787851000	-0.047263000	-1.976332000
C	-5.358911000	-1.644158000	1.529111000
H	-3.203192000	-1.774719000	1.656735000
C	-6.450964000	-1.244099000	0.752972000
H	-7.093729000	-0.354050000	-1.113837000
H	-5.518121000	-2.082614000	2.517306000
H	-7.467924000	-1.371239000	1.132373000
C	-2.084392000	0.918774000	-1.598185000
C	-1.866238000	2.008029000	-0.734871000
C	-2.228358000	1.164654000	-2.973007000
C	-1.799768000	3.310716000	-1.229307000
H	-1.734337000	1.838082000	0.335200000
C	-2.147577000	2.470839000	-3.470928000
H	-2.394638000	0.345841000	-3.674850000
C	-1.933481000	3.545427000	-2.602569000
H	-1.627790000	4.140713000	-0.541120000
H	-2.251715000	2.645518000	-4.544363000
H	-1.867276000	4.562956000	-2.994721000
C	0.852266000	4.874894000	0.995624000
H	1.889638000	5.163361000	1.231768000
H	0.780405000	4.814974000	-0.102124000
H	0.172198000	5.657170000	1.363348000
C	2.280815000	1.706364000	2.126222000
O	3.002360000	2.413603000	2.795831000

O	2.554065000	0.405183000	1.882389000
C	3.779568000	-0.101868000	2.419342000
H	4.635966000	0.475691000	2.040154000
H	3.775225000	-0.045709000	3.518179000
H	3.850088000	-1.144320000	2.089217000

^{3}TS1, G = -3727.404400 (i = -268 cm-1)

C	-0.719945000	-3.733932000	1.112567000
C	-0.315044000	-2.902872000	-0.032385000
C	-1.363912000	-1.095259000	2.078705000
C	-2.513227000	-1.973438000	1.700244000
C	-2.154182000	-3.470555000	1.611454000
C	0.035842000	-2.492601000	-1.146494000
C	0.382246000	-2.535432000	-2.575891000
H	0.460428000	-3.584790000	-2.900990000
C	-0.786387000	-0.592492000	3.133515000
Co	-0.080834000	-0.735433000	0.163677000
P	-1.796095000	0.490299000	-1.016878000
C	0.361404000	-0.372870000	2.213877000
H	0.571148000	0.668873000	1.921713000
H	-2.946917000	-1.640810000	0.753575000
H	-3.283360000	-1.818299000	2.476517000
H	-2.881084000	-3.960304000	0.944984000
H	-2.252460000	-3.941984000	2.601159000
H	-0.018269000	-3.549178000	1.938529000
H	-0.611895000	-4.793108000	0.825671000
H	-0.392831000	-2.049571000	-3.187326000
H	1.339824000	-2.039184000	-2.780980000
P	1.609177000	0.631251000	-0.968569000
C	0.943809000	1.743090000	-2.299353000
H	0.423635000	2.562769000	-1.784339000
H	1.793091000	2.210811000	-2.818210000
C	-1.435874000	0.770591000	-2.829135000
H	-2.098452000	1.587017000	-3.153335000
H	-1.828846000	-0.127091000	-3.328606000
C	0.017202000	1.021284000	-3.287774000
H	-0.029323000	1.614232000	-4.214725000
H	0.480636000	0.068039000	-3.580266000
C	2.415592000	1.848381000	0.150054000
C	1.602884000	2.818679000	0.766078000
C	3.787236000	1.820588000	0.441786000
C	2.156657000	3.748967000	1.649364000
H	0.529980000	2.858354000	0.565260000
C	4.337229000	2.753146000	1.326281000
H	4.431014000	1.067366000	-0.015287000
C	3.526327000	3.718467000	1.932202000
H	1.511441000	4.496868000	2.116758000
H	5.407841000	2.722599000	1.543613000
H	3.960515000	4.444212000	2.624225000
C	2.971158000	-0.314848000	-1.753316000
C	3.456856000	-0.043509000	-3.043690000
C	3.501374000	-1.407410000	-1.040191000
C	4.447358000	-0.852369000	-3.610781000
H	3.070346000	0.794025000	-3.625780000
C	4.499178000	-2.204054000	-1.605450000
H	3.141029000	-1.626691000	-0.034295000
C	4.970258000	-1.933243000	-2.895100000
H	4.810646000	-0.633173000	-4.617700000
H	4.905340000	-3.044702000	-1.037673000
H	5.743207000	-2.563722000	-3.341270000

C	-3.507510000	-0.174178000	-1.144257000
C	-4.642509000	0.536932000	-0.727395000
C	-3.660518000	-1.482423000	-1.643346000
C	-5.910044000	-0.050617000	-0.811138000
H	-4.542855000	1.550321000	-0.333821000
C	-4.927008000	-2.062517000	-1.731193000
H	-2.782879000	-2.062759000	-1.941901000
C	-6.055794000	-1.347363000	-1.312262000
H	-6.787711000	0.512041000	-0.483422000
H	-5.032269000	-3.078737000	-2.118607000
H	-7.046995000	-1.802955000	-1.374508000
C	-1.997559000	2.170812000	-0.287494000
C	-2.074782000	2.249090000	1.115969000
C	-2.018030000	3.361883000	-1.032261000
C	-2.161887000	3.485682000	1.758802000
H	-2.053747000	1.333781000	1.710256000
C	-2.092319000	4.600615000	-0.386253000
H	-1.968194000	3.344435000	-2.121714000
C	-2.161417000	4.666782000	1.008579000
H	-2.217343000	3.525687000	2.849194000
H	-2.095032000	5.517875000	-0.979798000
H	-2.214313000	5.636261000	1.509558000
C	-0.974619000	-0.380475000	4.588384000
H	-1.955418000	-0.746115000	4.924177000
H	-0.185274000	-0.912135000	5.144332000
H	-0.886487000	0.689958000	4.838434000
C	1.621914000	-1.141667000	2.449675000
O	2.715089000	-0.800333000	2.045344000
O	1.423675000	-2.255549000	3.171200000
C	2.577204000	-3.069374000	3.428451000
H	2.997266000	-3.447948000	2.484579000
H	3.347653000	-2.489440000	3.956441000
H	2.228112000	-3.901286000	4.050701000

^{3}INT1, G = -3727.423552

C	-1.024924000	-3.675229000	1.196704000
C	-0.480411000	-2.909324000	0.065827000
C	-1.329031000	-1.037516000	1.866589000
C	-2.616016000	-1.790109000	1.974749000
C	-2.473540000	-3.274675000	1.533432000
C	-0.096920000	-2.543742000	-1.050718000
C	0.252077000	-2.678321000	-2.473153000
H	0.365872000	-3.748157000	-2.707899000
C	-0.587249000	-0.412191000	2.790257000
Co	-0.201624000	-0.682752000	0.313049000
P	-1.817774000	0.490951000	-1.025280000
C	0.693255000	-0.055730000	2.098845000
H	0.964588000	1.006479000	2.088703000
H	-3.398128000	-1.300323000	1.377311000
H	-2.951767000	-1.744001000	3.024210000
H	-3.106314000	-3.469323000	0.654682000
H	-2.829979000	-3.940024000	2.333732000
H	-0.367187000	-3.544114000	2.068314000
H	-0.990188000	-4.739008000	0.906482000
H	-0.546355000	-2.278016000	-3.115366000
H	1.191631000	-2.172957000	-2.727459000
P	1.514992000	0.501848000	-0.941031000
C	0.926935000	1.473490000	-2.414797000
H	0.478030000	2.392228000	-2.012092000
H	1.818417000	1.803348000	-2.966636000

C	-1.501682000	0.598135000	-2.853943000
H	-2.130700000	1.418971000	-3.230205000
H	-1.951293000	-0.316791000	-3.266657000
C	-0.044151000	0.738494000	-3.344545000
H	-0.070716000	1.275801000	-4.305058000
H	0.365967000	-0.252422000	-3.580047000
C	2.385628000	1.828625000	-0.010757000
C	1.640474000	2.931564000	0.445942000
C	3.755444000	1.759084000	0.279770000
C	2.261016000	3.953290000	1.166938000
H	0.568109000	2.998064000	0.255705000
C	4.373490000	2.786023000	1.000168000
H	4.344395000	0.901181000	-0.046271000
C	3.631168000	3.883750000	1.444456000
H	1.668302000	4.803069000	1.514083000
H	5.442084000	2.721618000	1.219122000
H	4.117423000	4.682587000	2.009743000
C	2.856666000	-0.594674000	-1.549043000
C	3.597091000	-0.321825000	-2.711716000
C	3.166299000	-1.742833000	-0.798364000
C	4.622302000	-1.182603000	-3.114596000
H	3.388658000	0.562805000	-3.315241000
C	4.196384000	-2.596489000	-1.197727000
H	2.591163000	-1.986628000	0.093224000
C	4.925676000	-2.319328000	-2.358571000
H	5.186788000	-0.960662000	-4.023262000
H	4.423627000	-3.484381000	-0.603188000
H	5.727690000	-2.990109000	-2.675691000
C	-3.519499000	-0.186380000	-0.975044000
C	-4.617349000	0.557274000	-0.515251000
C	-3.707586000	-1.522189000	-1.376108000
C	-5.885203000	-0.030763000	-0.455536000
H	-4.487433000	1.593423000	-0.197905000
C	-4.976377000	-2.101360000	-1.321550000
H	-2.859746000	-2.124627000	-1.711784000
C	-6.067412000	-1.357708000	-0.856489000
H	-6.734217000	0.554520000	-0.094477000
H	-5.110873000	-3.140068000	-1.632257000
H	-7.058917000	-1.813837000	-0.806486000
C	-1.934187000	2.212924000	-0.398819000
C	-1.987471000	2.395308000	0.996722000
C	-1.911395000	3.343460000	-1.232823000
C	-2.010618000	3.679444000	1.543841000
H	-1.993874000	1.527746000	1.658433000
C	-1.921314000	4.628180000	-0.680023000
H	-1.875687000	3.242486000	-2.318324000
C	-1.967807000	4.799789000	0.706726000
H	-2.048052000	3.803088000	2.628497000
H	-1.891424000	5.497988000	-1.340153000
H	-1.969753000	5.805036000	1.134359000
C	-0.865748000	-0.071499000	4.220757000
H	-1.865036000	-0.392119000	4.548664000
H	-0.109618000	-0.541729000	4.873505000
H	-0.779809000	1.019142000	4.370575000
C	1.894957000	-0.869601000	2.384015000
O	3.039216000	-0.458355000	2.366536000
O	1.611879000	-2.173032000	2.643076000
C	2.723839000	-3.019315000	2.943556000
H	3.447505000	-3.028187000	2.114539000
H	3.241276000	-2.677307000	3.852679000
H	2.311478000	-4.023944000	3.096989000

^{3}TS2, G = -3727.399976 (i = -246 cm-1)

C	-1.979538000	-3.897844000	0.425656000
C	-0.882851000	-2.926678000	0.134178000
C	-1.270156000	-1.610264000	1.685207000
C	-2.409460000	-2.380698000	2.271307000
C	-3.104609000	-3.203874000	1.187788000
C	0.136384000	-2.748621000	-0.634177000
C	0.942404000	-3.512992000	-1.617710000
H	0.417524000	-4.443090000	-1.891617000
C	-0.048772000	-1.226594000	2.396841000
Co	-0.253330000	-0.992105000	0.143662000
P	-1.882209000	0.383637000	-0.856454000
C	0.287151000	0.106985000	2.355522000
H	-0.475953000	0.841618000	2.097221000
H	-3.110931000	-1.685012000	2.762585000
H	-2.041265000	-3.055079000	3.070126000
H	-3.663993000	-2.541211000	0.511487000
H	-3.814450000	-3.926473000	1.615740000
H	-1.554150000	-4.712292000	1.038767000
H	-2.313782000	-4.344597000	-0.524675000
H	1.149698000	-2.942312000	-2.532099000
H	1.920128000	-3.781247000	-1.188748000
P	1.436328000	0.029397000	-1.000349000
C	0.816785000	0.640377000	-2.640972000
H	0.486652000	1.673472000	-2.461548000
H	1.670870000	0.719625000	-3.328745000
C	-1.731272000	-0.050260000	-2.657790000
H	-2.320863000	0.672797000	-3.240420000
H	-2.250298000	-1.015157000	-2.747066000
C	-0.306540000	-0.217061000	-3.245806000
H	-0.369070000	0.009820000	-4.321178000
H	-0.021733000	-1.277117000	-3.186068000
C	2.237772000	1.543008000	-0.311278000
C	1.432289000	2.564488000	0.213788000
C	3.629725000	1.734459000	-0.366492000
C	1.996694000	3.756647000	0.669230000
H	0.355479000	2.434031000	0.276581000
C	4.193281000	2.928590000	0.093592000
H	4.281728000	0.957689000	-0.767286000
C	3.380478000	3.942703000	0.611047000
H	1.347476000	4.532805000	1.080308000
H	5.276608000	3.063744000	0.047068000
H	3.826505000	4.872377000	0.972431000
C	2.878260000	-1.061617000	-1.306652000
C	3.432917000	-1.294975000	-2.571680000
C	3.440593000	-1.688477000	-0.179230000
C	4.529973000	-2.154092000	-2.710541000
H	3.018305000	-0.823830000	-3.464203000
C	4.541266000	-2.533985000	-0.319799000
H	3.017013000	-1.508471000	0.811524000
C	5.085562000	-2.773502000	-1.588379000
H	4.949201000	-2.336756000	-3.702763000
H	4.972514000	-3.012843000	0.562579000
H	5.941979000	-3.442786000	-1.699821000
C	-3.636569000	-0.003475000	-0.441497000
C	-4.048087000	0.149755000	0.894328000
C	-4.574839000	-0.441500000	-1.390868000
C	-5.361426000	-0.129297000	1.274070000
H	-3.333052000	0.460836000	1.656369000
C	-5.888475000	-0.732676000	-1.005827000

H	-4.303052000	-0.562115000	-2.439829000
C	-6.285955000	-0.578270000	0.324673000
H	-5.658979000	-0.009310000	2.318452000
H	-6.603656000	-1.078830000	-1.755948000
H	-7.311837000	-0.807973000	0.622172000
C	-1.890398000	2.231018000	-0.781632000
C	-2.026632000	2.844144000	0.479391000
C	-1.703974000	3.050275000	-1.907053000
C	-1.972887000	4.232623000	0.611171000
H	-2.161637000	2.241264000	1.378764000
C	-1.640360000	4.441928000	-1.772389000
H	-1.603003000	2.622495000	-2.905246000
C	-1.771808000	5.037452000	-0.515831000
H	-2.076719000	4.684827000	1.600122000
H	-1.487758000	5.060406000	-2.660019000
H	-1.717175000	6.123637000	-0.413195000
C	0.814118000	-2.312029000	2.997336000
H	0.174094000	-3.046336000	3.509115000
H	1.340281000	-2.850088000	2.192924000
H	1.560046000	-1.925048000	3.699921000
C	1.533241000	0.738092000	2.859874000
O	1.560430000	1.844284000	3.352276000
O	2.627577000	-0.018362000	2.676771000
C	3.879826000	0.551490000	3.083175000
H	4.062441000	1.487021000	2.536196000
H	3.879616000	0.754305000	4.163940000
H	4.644602000	-0.192350000	2.831449000

^{3}INT2, G = -3727.459072

C	-2.817850000	-2.755171000	1.663064000
C	-1.384678000	-2.334237000	1.405213000
C	-1.028289000	-1.293067000	2.302395000
C	-2.234455000	-0.817751000	3.082410000
C	-3.424419000	-1.538740000	2.404087000
C	-0.512199000	-2.525791000	0.327983000
C	-0.703033000	-3.518246000	-0.762800000
H	-1.646967000	-3.348330000	-1.317471000
C	0.375055000	-0.939746000	2.579577000
Co	-0.301042000	-0.628811000	0.414543000
P	-1.957592000	0.394028000	-1.038448000
C	0.691222000	0.396971000	2.488375000
H	-0.118943000	1.128823000	2.423397000
H	-2.343263000	0.278083000	3.097626000
H	-2.104451000	-1.129791000	4.134780000
H	-3.892547000	-0.865404000	1.672466000
H	-4.201468000	-1.828515000	3.125532000
H	-2.798231000	-3.650008000	2.310520000
H	-3.365415000	-3.026315000	0.750525000
H	0.122945000	-3.505345000	-1.488484000
H	-0.766660000	-4.537790000	-0.343274000
P	1.387924000	-0.287542000	-1.126923000
C	0.824788000	0.077679000	-2.859980000
H	0.680826000	1.167748000	-2.902500000
H	1.664934000	-0.142468000	-3.535728000
C	-1.765703000	0.013031000	-2.855094000
H	-1.934500000	0.939705000	-3.424632000
H	-2.608286000	-0.646822000	-3.105724000
C	-0.453901000	-0.663266000	-3.278600000
H	-0.468759000	-0.748895000	-4.377030000
H	-0.435157000	-1.696371000	-2.898752000

C	2.521068000	1.129121000	-0.827088000
C	1.982538000	2.301777000	-0.273902000
C	3.870157000	1.108453000	-1.219891000
C	2.776839000	3.440743000	-0.120977000
H	0.940271000	2.329179000	0.046942000
C	4.662033000	2.249607000	-1.064824000
H	4.304689000	0.202339000	-1.647490000
C	4.117073000	3.417908000	-0.518384000
H	2.344074000	4.341045000	0.320163000
H	5.710724000	2.225376000	-1.371007000
H	4.740380000	4.306877000	-0.395411000
C	2.477870000	-1.753300000	-1.254234000
C	2.548412000	-2.565021000	-2.396570000
C	3.214206000	-2.111232000	-0.109669000
C	3.342727000	-3.718362000	-2.392887000
H	1.985989000	-2.318099000	-3.298493000
C	4.011870000	-3.255743000	-0.113701000
H	3.167764000	-1.485197000	0.783275000
C	4.074692000	-4.065728000	-1.254884000
H	3.387608000	-4.344425000	-3.287271000
H	4.584022000	-3.519462000	0.779234000
H	4.694693000	-4.965511000	-1.255469000
C	-3.764231000	0.167446000	-0.774933000
C	-4.601003000	1.162009000	-0.244056000
C	-4.306718000	-1.101944000	-1.051832000
C	-5.948222000	0.886193000	0.014362000
H	-4.212102000	2.158675000	-0.031043000
C	-5.652561000	-1.371550000	-0.799255000
H	-3.675566000	-1.896990000	-1.457534000
C	-6.477114000	-0.378574000	-0.258122000
H	-6.587201000	1.670165000	0.428019000
H	-6.055545000	-2.363223000	-1.018163000
H	-7.529076000	-0.590357000	-0.052804000
C	-1.637480000	2.190625000	-0.810352000
C	-1.692395000	2.678671000	0.511752000
C	-1.214300000	3.057051000	-1.829925000
C	-1.327109000	3.992344000	0.806948000
H	-2.020022000	2.017869000	1.318602000
C	-0.840164000	4.372251000	-1.531538000
H	-1.159444000	2.721428000	-2.866288000
C	-0.889951000	4.841711000	-0.216649000
H	-1.370507000	4.348150000	1.838651000
H	-0.501047000	5.029385000	-2.335577000
H	-0.586853000	5.865834000	0.012623000
C	1.323957000	-2.047570000	2.963003000
H	0.986475000	-2.444350000	3.935590000
H	1.277306000	-2.868175000	2.236814000
H	2.356125000	-1.701984000	3.062049000
C	1.998823000	1.076672000	2.662093000
O	2.079599000	2.239503000	2.995985000
O	3.064882000	0.318203000	2.368321000
C	4.350786000	0.949511000	2.456851000
H	4.397295000	1.809087000	1.774948000
H	4.543500000	1.287244000	3.485421000
H	5.079656000	0.187343000	2.158621000

^{3}INT3, G = -3727.484468

C	-2.919558000	-3.624618000	-0.769538000
C	-1.981501000	-2.587240000	-0.172584000
C	-2.678642000	-1.888142000	0.867353000

C	-4.141407000	-2.267231000	0.855453000
C	-4.320164000	-3.065900000	-0.452428000
C	-0.697576000	-2.286832000	-0.576100000
C	0.175709000	-3.257676000	-1.302735000
H	0.803817000	-3.790306000	-0.565111000
C	-2.066513000	-1.005798000	1.755449000
Co	-0.083008000	-0.539556000	0.036125000
P	-1.182208000	1.350955000	-0.898438000
C	-0.632401000	-0.990626000	1.956454000
H	-0.266638000	-0.140966000	2.550477000
H	-4.805583000	-1.391050000	0.918559000
H	-4.360668000	-2.890415000	1.741913000
H	-4.626513000	-2.382444000	-1.259039000
H	-5.087670000	-3.849169000	-0.375654000
H	-2.746579000	-4.586044000	-0.249779000
H	-2.748512000	-3.800247000	-1.841406000
H	-0.403609000	-4.028078000	-1.840135000
H	0.871079000	-2.771286000	-2.000600000
P	2.040103000	0.248021000	-0.765777000
C	1.812893000	1.310922000	-2.265549000
H	1.663620000	2.340092000	-1.904388000
H	2.740967000	1.321143000	-2.854964000
C	-0.753286000	1.452661000	-2.714250000
H	-0.816300000	2.509263000	-3.015613000
H	-1.556299000	0.924944000	-3.249649000
C	0.608429000	0.856071000	-3.119353000
H	0.791280000	1.144641000	-4.166360000
H	0.541034000	-0.245021000	-3.124528000
C	3.037042000	1.227546000	0.420323000
C	3.806675000	2.336531000	0.033258000
C	2.975770000	0.865680000	1.778444000
C	4.495706000	3.081027000	0.995256000
H	3.874270000	2.634326000	-1.014995000
C	3.673812000	1.609308000	2.733774000
H	2.379235000	0.004889000	2.094329000
C	4.428736000	2.721545000	2.345460000
H	5.086906000	3.946684000	0.686759000
H	3.619473000	1.322577000	3.786833000
H	4.965108000	3.308693000	3.094919000
C	3.095648000	-1.171016000	-1.259678000
C	3.611195000	-1.348292000	-2.552407000
C	3.323002000	-2.159842000	-0.283259000
C	4.354830000	-2.493571000	-2.861138000
H	3.439250000	-0.605923000	-3.333599000
C	4.076459000	-3.292695000	-0.593484000
H	2.895114000	-2.056975000	0.717398000
C	4.592485000	-3.464026000	-1.884104000
H	4.749811000	-2.623358000	-3.871683000
H	4.251032000	-4.051669000	0.173154000
H	5.174560000	-4.356015000	-2.128183000
C	-3.009920000	1.457682000	-0.841985000
C	-3.674298000	2.424501000	-0.069201000
C	-3.763095000	0.467628000	-1.499616000
C	-5.068833000	2.406636000	0.034276000
H	-3.106272000	3.193705000	0.457148000
C	-5.156116000	0.461959000	-1.402955000
H	-3.264240000	-0.314262000	-2.078631000
C	-5.812777000	1.429202000	-0.633181000
H	-5.574271000	3.163589000	0.638805000
H	-5.732607000	-0.306161000	-1.923742000
H	-6.902374000	1.418695000	-0.553819000

C	-0.564597000	2.920574000	-0.174686000
C	0.137277000	2.868096000	1.040541000
C	-0.741852000	4.165573000	-0.805846000
C	0.674627000	4.027590000	1.606402000
H	0.285887000	1.914649000	1.547615000
C	-0.211321000	5.325219000	-0.236768000
H	-1.299069000	4.239143000	-1.742810000
C	0.503463000	5.257270000	0.966106000
H	1.238254000	3.960315000	2.539366000
H	-0.353438000	6.286698000	-0.735903000
H	0.926906000	6.165452000	1.401804000
C	-2.899384000	-0.021662000	2.537815000
H	-3.778978000	0.320703000	1.975731000
H	-3.256578000	-0.490064000	3.472369000
H	-2.304985000	0.858959000	2.822655000
C	0.200018000	-2.183880000	2.305848000
O	1.383825000	-2.086257000	2.588372000
O	-0.444736000	-3.353084000	2.307796000
C	0.338763000	-4.520742000	2.584653000
H	1.132599000	-4.640553000	1.831793000
H	0.801051000	-4.453923000	3.580244000
H	-0.355766000	-5.367710000	2.541112000

^{3}TS3, G = -3727.471287 (i = -264 cm-1)

C	-3.402214000	-3.214809000	-0.833337000
C	-2.342970000	-2.343831000	-0.199361000
C	-2.958103000	-1.555460000	0.850488000
C	-4.458407000	-1.682558000	0.796948000
C	-4.714420000	-2.449989000	-0.527318000
C	-1.007980000	-2.248962000	-0.430547000
C	-0.142447000	-3.296044000	-1.076588000
H	0.541578000	-3.732168000	-0.329671000
C	-2.093242000	-0.963813000	1.712391000
Co	0.105299000	-0.576600000	-0.283006000
P	-1.021518000	1.361106000	-0.962450000
C	-0.672787000	-1.313118000	1.450309000
H	0.037243000	-0.506216000	1.753592000
H	-4.965605000	-0.705565000	0.819949000
H	-4.818189000	-2.254183000	1.670313000
H	-4.903334000	-1.724764000	-1.331711000
H	-5.586724000	-3.116977000	-0.471932000
H	-3.394064000	-4.199284000	-0.328855000
H	-3.239475000	-3.398263000	-1.905349000
H	-0.760987000	-4.114640000	-1.479429000
H	0.490385000	-2.901097000	-1.886608000
P	2.217882000	0.344032000	-0.768962000
C	2.099853000	1.707346000	-2.010788000
H	1.873918000	2.630366000	-1.454592000
H	3.071843000	1.866592000	-2.499113000
C	-0.418153000	1.885857000	-2.659239000
H	-0.474400000	2.983962000	-2.710161000
H	-1.141077000	1.498961000	-3.391768000
C	0.991397000	1.401696000	-3.042314000
H	1.255249000	1.878922000	-3.999285000
H	0.964594000	0.317520000	-3.249023000
C	3.146972000	1.006171000	0.667209000
C	3.886572000	2.198026000	0.615243000
C	3.062935000	0.296135000	1.879222000
C	4.525159000	2.677838000	1.763316000
H	3.968986000	2.765549000	-0.313767000

C	3.709771000	0.775730000	3.020954000
H	2.492853000	-0.636603000	1.936305000
C	4.436731000	1.970642000	2.966632000
H	5.093446000	3.609911000	1.715273000
H	3.639411000	0.217465000	3.957617000
H	4.934668000	2.350098000	3.862212000
C	3.310964000	-0.948371000	-1.487814000
C	4.129951000	-0.760680000	-2.611649000
C	3.289945000	-2.203268000	-0.849933000
C	4.919181000	-1.813664000	-3.087785000
H	4.163455000	0.200654000	-3.127864000
C	4.089517000	-3.246697000	-1.320910000
H	2.643484000	-2.363963000	0.018087000
C	4.903277000	-3.054787000	-2.443631000
H	5.550557000	-1.660087000	-3.966384000
H	4.068688000	-4.215225000	-0.815330000
H	5.521784000	-3.873891000	-2.818639000
C	-2.851983000	1.426754000	-1.102587000
C	-3.637127000	2.014227000	-0.094988000
C	-3.494510000	0.786764000	-2.176898000
C	-5.031996000	1.982782000	-0.174856000
H	-3.161582000	2.502321000	0.757718000
C	-4.889389000	0.769305000	-2.261063000
H	-2.912080000	0.288283000	-2.956070000
C	-5.662998000	1.366978000	-1.260851000
H	-5.627583000	2.445790000	0.615730000
H	-5.372793000	0.277878000	-3.108802000
H	-6.753459000	1.347962000	-1.324824000
C	-0.597823000	2.807454000	0.100148000
C	0.161141000	2.608237000	1.263367000
C	-0.988259000	4.116142000	-0.239415000
C	0.543678000	3.689238000	2.064254000
H	0.477833000	1.604323000	1.546287000
C	-0.613227000	5.195782000	0.562461000
H	-1.595954000	4.296212000	-1.129827000
C	0.158405000	4.984996000	1.712954000
H	1.149845000	3.512375000	2.955531000
H	-0.922468000	6.207310000	0.288228000
H	0.457133000	5.833145000	2.333780000
C	-2.447127000	-0.046043000	2.841592000
H	-3.523618000	0.173559000	2.858424000
H	-2.164837000	-0.489319000	3.812422000
H	-1.902041000	0.909931000	2.757634000
C	-0.084729000	-2.565592000	2.043273000
O	1.104660000	-2.818905000	1.985142000
O	-0.979457000	-3.354116000	2.634615000
C	-0.492404000	-4.587117000	3.184635000
H	-0.074282000	-5.223009000	2.390254000
H	0.286982000	-4.393885000	3.936015000
H	-1.358479000	-5.074439000	3.646780000

^{3}INT4, G = -3727.522645

C	-2.155844000	-2.970438000	-0.283112000
C	-0.770665000	-2.457785000	-0.004461000
C	-0.719187000	-1.770206000	1.278537000
C	-2.087937000	-1.718609000	1.901588000
C	-2.884243000	-2.782174000	1.085073000
C	0.504717000	-2.573270000	-0.531344000
C	0.927616000	-3.367045000	-1.727488000
H	1.110124000	-4.416354000	-1.435614000

C	0.579948000	-1.457805000	1.611900000
Co	-0.033204000	-0.431919000	-0.409293000
P	-1.948959000	0.896800000	-0.734666000
C	1.482785000	-2.177612000	0.585678000
H	2.342251000	-1.574493000	0.253121000
H	-2.537089000	-0.718596000	1.806318000
H	-2.060080000	-1.945777000	2.977866000
H	-3.936059000	-2.493916000	0.952453000
H	-2.871022000	-3.737042000	1.633211000
H	-2.137305000	-4.023509000	-0.603496000
H	-2.649999000	-2.402363000	-1.083369000
H	0.149342000	-3.361791000	-2.504325000
H	1.861686000	-2.983867000	-2.164430000
P	1.484553000	1.319647000	-0.694589000
C	0.651243000	2.835847000	-1.377607000
H	0.093987000	3.339620000	-0.573382000
H	1.440321000	3.525453000	-1.712487000
C	-1.732540000	2.103266000	-2.153102000
H	-2.284299000	3.017319000	-1.886252000
H	-2.247028000	1.670743000	-3.023302000
C	-0.284589000	2.444670000	-2.540905000
H	-0.324574000	3.282339000	-3.254877000
H	0.154951000	1.603468000	-3.104405000
C	2.442315000	1.844871000	0.772468000
C	1.942027000	2.817342000	1.655031000
C	3.621383000	1.154821000	1.111882000
C	2.609445000	3.093974000	2.852072000
H	1.023508000	3.359280000	1.426402000
C	4.285481000	1.436168000	2.307940000
H	4.026632000	0.393609000	0.441277000
C	3.780285000	2.404435000	3.182672000
H	2.207741000	3.852084000	3.528639000
H	5.199148000	0.892052000	2.558337000
H	4.298344000	2.620702000	4.119919000
C	2.741991000	0.962411000	-1.984069000
C	3.861401000	1.794077000	-2.167214000
C	2.549887000	-0.130011000	-2.844724000
C	4.773661000	1.527480000	-3.190002000
H	4.027284000	2.647377000	-1.504877000
C	3.461990000	-0.392074000	-3.872559000
H	1.683689000	-0.781875000	-2.712588000
C	4.575721000	0.434485000	-4.043645000
H	5.643652000	2.175155000	-3.322280000
H	3.301676000	-1.246241000	-4.534611000
H	5.292837000	0.228351000	-4.841917000
C	-3.614983000	0.187677000	-1.057077000
C	-4.627122000	0.158235000	-0.083414000
C	-3.837259000	-0.461083000	-2.286980000
C	-5.838184000	-0.493690000	-0.340532000
H	-4.479407000	0.645591000	0.881920000
C	-5.048888000	-1.106277000	-2.542629000
H	-3.054496000	-0.481758000	-3.050751000
C	-6.054221000	-1.124569000	-1.568796000
H	-6.616043000	-0.505458000	0.426771000
H	-5.205452000	-1.602190000	-3.503589000
H	-7.001295000	-1.631924000	-1.767139000
C	-2.183323000	2.008319000	0.717679000
C	-1.372115000	1.835303000	1.849582000
C	-3.136667000	3.043863000	0.715208000
C	-1.501127000	2.679737000	2.956945000
H	-0.625338000	1.041773000	1.861220000

C	-3.268141000	3.884783000	1.821960000
H	-3.789809000	3.192362000	-0.148449000
C	-2.449184000	3.705638000	2.944643000
H	-0.852856000	2.534703000	3.824263000
H	-4.013062000	4.684018000	1.809234000
H	-2.552568000	4.367794000	3.807663000
C	1.093294000	-0.845898000	2.875757000
H	0.262684000	-0.558210000	3.536118000
H	1.738727000	-1.556177000	3.419293000
H	1.705312000	0.046821000	2.679981000
C	2.123300000	-3.379330000	1.298943000
O	3.197009000	-3.323497000	1.849824000
O	1.337282000	-4.457254000	1.289833000
C	1.809289000	-5.606819000	2.008485000
H	2.773012000	-5.946381000	1.601581000
H	1.935329000	-5.366972000	3.074658000
H	1.044412000	-6.380862000	1.878076000

^{3}INT5, G = -3727.531187

C	2.696214000	-2.298047000	-1.795947000
C	1.220880000	-2.052804000	-1.635634000
C	0.736845000	-1.181595000	-2.571520000
C	1.814303000	-0.691448000	-3.489451000
C	3.110729000	-1.137873000	-2.747897000
C	0.128856000	-2.589998000	-0.825069000
C	0.284050000	-3.721881000	0.147330000
H	0.209193000	-4.684832000	-0.387066000
C	-0.765184000	-1.131082000	-2.541434000
Co	0.124850000	-0.728428000	0.079027000
P	1.883194000	0.251780000	1.217881000
C	-1.057521000	-1.956301000	-1.276384000
H	1.779230000	0.392965000	-3.686923000
H	1.713532000	-1.183242000	-4.475724000
H	3.490732000	-0.299005000	-2.143081000
H	3.915110000	-1.425462000	-3.439486000
H	2.852506000	-3.286306000	-2.264127000
H	3.259090000	-2.297813000	-0.852834000
H	1.272682000	-3.680774000	0.627609000
H	-0.506119000	-3.714811000	0.906997000
P	-1.392733000	0.832180000	0.919544000
C	-0.745861000	1.731696000	2.421465000
H	-0.091392000	2.555092000	2.101441000
H	-1.610677000	2.182686000	2.929951000
C	1.500225000	0.553341000	3.015444000
H	2.110095000	1.400929000	3.361741000
H	1.876872000	-0.329440000	3.553269000
C	0.013142000	0.770508000	3.355886000
H	-0.037985000	1.161169000	4.384252000
H	-0.503868000	-0.203540000	3.386039000
C	-1.832636000	2.120637000	-0.305963000
C	-1.178551000	3.363635000	-0.346744000
C	-2.753588000	1.800167000	-1.323371000
C	-1.452762000	4.273401000	-1.372686000
H	-0.436937000	3.634168000	0.405678000
C	-3.025481000	2.713965000	-2.343734000
H	-3.251748000	0.829160000	-1.323671000
C	-2.377273000	3.954518000	-2.371068000
H	-0.932345000	5.233872000	-1.390057000
H	-3.745009000	2.452600000	-3.123545000
H	-2.589470000	4.667638000	-3.171313000

C	-2.969661000	0.151921000	1.560846000
C	-4.157649000	0.900755000	1.621514000
C	-2.945179000	-1.148573000	2.088378000
C	-5.302408000	0.348659000	2.201103000
H	-4.191251000	1.912736000	1.211421000
C	-4.088278000	-1.693200000	2.680910000
H	-2.036961000	-1.749305000	2.007246000
C	-5.267987000	-0.945750000	2.736401000
H	-6.225932000	0.931612000	2.237588000
H	-4.059874000	-2.710337000	3.077621000
H	-6.166176000	-1.373633000	3.188244000
C	3.513662000	-0.587001000	1.255992000
C	4.656799000	-0.055802000	0.639757000
C	3.576841000	-1.867505000	1.837494000
C	5.845019000	-0.794693000	0.608849000
H	4.624337000	0.933146000	0.178370000
C	4.766450000	-2.597208000	1.812987000
H	2.688713000	-2.306622000	2.301947000
C	5.903371000	-2.062947000	1.193656000
H	6.729209000	-0.373372000	0.124421000
H	4.803834000	-3.589293000	2.269181000
H	6.832461000	-2.637187000	1.166150000
C	2.201993000	1.905024000	0.470988000
C	1.964678000	2.052523000	-0.907326000
C	2.644934000	3.018534000	1.204482000
C	2.163441000	3.281066000	-1.540435000
H	1.614893000	1.202144000	-1.494328000
C	2.831863000	4.251799000	0.572649000
H	2.847015000	2.939502000	2.274111000
C	2.591815000	4.386150000	-0.799471000
H	1.965809000	3.375903000	-2.610502000
H	3.169153000	5.111225000	1.156833000
H	2.735956000	5.352506000	-1.288369000
C	-1.373614000	-1.740833000	-3.823130000
H	-1.061784000	-1.159769000	-4.704465000
H	-1.035023000	-2.780611000	-3.953436000
H	-2.470276000	-1.729016000	-3.764268000
C	-2.414171000	-2.350854000	-0.867262000
O	-2.698149000	-3.277482000	-0.132643000
O	-3.350428000	-1.539650000	-1.406177000
C	-4.716128000	-1.818807000	-1.076520000
H	-4.980378000	-2.849183000	-1.356083000
H	-4.886459000	-1.683556000	0.000318000
H	-5.317794000	-1.102223000	-1.648143000
H	-1.145295000	-0.099088000	-2.448069000

INT2-Rh, G = -2455.306567

C	-4.059505000	-2.579109000	0.159806000
C	-2.739072000	-1.845612000	0.356131000
C	-2.870091000	-0.949277000	1.502091000
C	-4.319969000	-0.930226000	1.937898000
C	-4.850774000	-2.294484000	1.451108000
C	-1.665233000	-1.903858000	-0.474312000
C	-1.555029000	-2.908235000	-1.589961000
H	-2.014992000	-3.879256000	-1.338244000
C	-1.817619000	-0.306054000	2.086138000
Rh	0.036342000	-0.815077000	-0.352005000
P	-0.912123000	1.045142000	-1.202569000
C	-0.442372000	-0.663614000	1.683778000
H	0.323144000	-0.029716000	2.156572000

H	-4.850935000	-0.110326000	1.422725000
H	-4.451857000	-0.777530000	3.018837000
H	-5.941030000	-2.301393000	1.302104000
H	-4.614728000	-3.064926000	2.204525000
H	-3.931294000	-3.655166000	-0.039810000
H	-4.572867000	-2.152876000	-0.721539000
H	-2.053990000	-2.534748000	-2.502204000
H	-0.503091000	-3.106762000	-1.869225000
P	2.249958000	0.219592000	-0.586280000
C	2.240294000	1.540673000	-1.895439000
H	1.895683000	2.486733000	-1.452015000
H	3.271113000	1.696251000	-2.245786000
C	-0.182249000	1.397855000	-2.884719000
H	-0.401659000	2.455257000	-3.097350000
H	-0.752000000	0.804729000	-3.613902000
C	1.323963000	1.108493000	-3.059516000
H	1.648415000	1.623016000	-3.977190000
H	1.468916000	0.032767000	-3.259152000
C	3.047314000	0.935883000	0.897890000
C	3.547898000	2.245658000	0.961572000
C	3.096745000	0.122056000	2.045785000
C	4.086631000	2.733499000	2.157244000
H	3.518863000	2.898050000	0.087221000
C	3.638195000	0.612001000	3.235815000
H	2.713945000	-0.901495000	2.003930000
C	4.131160000	1.921299000	3.294349000
H	4.472217000	3.755047000	2.197691000
H	3.672755000	-0.028457000	4.120368000
H	4.550342000	2.307121000	4.226708000
C	3.512036000	-0.956657000	-1.230027000
C	4.873216000	-0.602699000	-1.257140000
C	3.118768000	-2.207707000	-1.730016000
C	5.819768000	-1.484956000	-1.781348000
H	5.197411000	0.363166000	-0.860630000
C	4.068576000	-3.089284000	-2.258290000
H	2.068100000	-2.502713000	-1.701818000
C	5.418602000	-2.729311000	-2.284338000
H	6.875260000	-1.202323000	-1.795908000
H	3.750456000	-4.060433000	-2.644996000
H	6.161411000	-3.418836000	-2.693006000
C	-2.726710000	1.003947000	-1.469511000
C	-3.596107000	1.635239000	-0.566284000
C	-3.265633000	0.243372000	-2.521991000
C	-4.981142000	1.526137000	-0.728233000
H	-3.199780000	2.211363000	0.271050000
C	-4.648476000	0.134655000	-2.678774000
H	-2.613194000	-0.284433000	-3.220972000
C	-5.510539000	0.778625000	-1.783097000
H	-5.646672000	2.026814000	-0.021157000
H	-5.053955000	-0.457825000	-3.502184000
H	-6.592726000	0.692953000	-1.906232000
C	-0.636510000	2.623530000	-0.291663000
C	0.106899000	2.680287000	0.893688000
C	-1.160354000	3.817789000	-0.824809000
C	0.336229000	3.902415000	1.534338000
H	0.525057000	1.774066000	1.320708000
C	-0.936591000	5.036081000	-0.182489000
H	-1.757699000	3.800550000	-1.739507000
C	-0.185135000	5.081712000	0.998839000
H	0.929719000	3.923836000	2.450859000
H	-1.351088000	5.954099000	-0.605573000

H	-0.008303000	6.037104000	1.498585000
C	-1.970477000	0.738796000	3.160517000
H	-3.022095000	0.990580000	3.350969000
H	-1.515436000	0.406291000	4.109959000
H	-1.453528000	1.669521000	2.872476000
C	0.001044000	-2.091239000	1.829035000
O	0.789615000	-2.541134000	0.982052000
O	-0.461016000	-2.794833000	2.831151000
C	-0.089281000	-4.188183000	2.902095000
H	-0.461007000	-4.716278000	2.012570000
H	1.003230000	-4.288707000	2.962042000
H	-0.564636000	-4.576916000	3.808885000

TS3-Rh, G = -2455.269959 (i = -323 cm-1)

C	-3.889128000	-2.540781000	-0.969601000
C	-2.779849000	-1.855873000	-0.209921000
C	-3.342982000	-1.046663000	0.835442000
C	-4.839861000	-0.989287000	0.707272000
C	-5.150511000	-2.274805000	-0.105445000
C	-1.415770000	-1.939359000	-0.336009000
C	-0.743509000	-3.164600000	-0.920656000
H	-0.914704000	-4.059291000	-0.298940000
C	-2.439411000	-0.624241000	1.760585000
Rh	0.175527000	-0.588599000	-0.387851000
P	-0.627656000	1.384163000	-0.975618000
C	-1.076166000	-1.139948000	1.519305000
H	-0.297555000	-0.425639000	1.829437000
H	-5.132595000	-0.090332000	0.142458000
H	-5.361085000	-0.953986000	1.675213000
H	-6.065536000	-2.185255000	-0.708777000
H	-5.294984000	-3.117164000	0.590320000
H	-3.697869000	-3.610991000	-1.145167000
H	-3.978486000	-2.067228000	-1.963849000
H	-1.169577000	-3.357550000	-1.918440000
H	0.349033000	-3.067290000	-1.054150000
P	2.303151000	0.261175000	-0.704034000
C	2.478361000	1.694227000	-1.852294000
H	2.287938000	2.608850000	-1.269725000
H	3.509431000	1.761709000	-2.227667000
C	0.023977000	1.960261000	-2.636831000
H	-0.079496000	3.055519000	-2.653165000
H	-0.672027000	1.568187000	-3.391243000
C	1.459984000	1.547788000	-2.997960000
H	1.784123000	2.163647000	-3.851474000
H	1.457537000	0.505454000	-3.357569000
C	3.187688000	0.707180000	0.845431000
C	4.085259000	1.784967000	0.907756000
C	2.928490000	-0.043486000	2.007950000
C	4.707081000	2.113669000	2.116882000
H	4.306937000	2.381636000	0.021030000
C	3.558667000	0.284873000	3.210581000
H	2.224840000	-0.881251000	1.986685000
C	4.444816000	1.366856000	3.269332000
H	5.398651000	2.958820000	2.155754000
H	3.350189000	-0.303410000	4.107554000
H	4.929776000	1.627759000	4.213149000
C	3.288225000	-1.116501000	-1.429202000
C	3.988367000	-1.015423000	-2.641704000
C	3.273900000	-2.348172000	-0.744923000
C	4.668004000	-2.125308000	-3.156605000

H	4.013274000	-0.078801000	-3.200952000
C	3.956715000	-3.451086000	-1.261374000
H	2.724407000	-2.453880000	0.194898000
C	4.655145000	-3.342583000	-2.469781000
H	5.210257000	-2.033917000	-4.100902000
H	3.940062000	-4.399484000	-0.718977000
H	5.187109000	-4.206592000	-2.875259000
C	-2.447495000	1.524566000	-1.221386000
C	-3.257203000	2.342457000	-0.418606000
C	-3.037670000	0.791612000	-2.267332000
C	-4.624903000	2.464531000	-0.690923000
H	-2.827289000	2.899230000	0.415181000
C	-4.400438000	0.918316000	-2.538734000
H	-2.432443000	0.110122000	-2.870717000
C	-5.196172000	1.766102000	-1.757619000
H	-5.243156000	3.110410000	-0.063095000
H	-4.844393000	0.351306000	-3.360309000
H	-6.262606000	1.867467000	-1.972014000
C	-0.213219000	2.774048000	0.171346000
C	0.353219000	2.511243000	1.428235000
C	-0.450608000	4.111933000	-0.198153000
C	0.692607000	3.557052000	2.292454000
H	0.554570000	1.485305000	1.731783000
C	-0.114355000	5.155845000	0.665941000
H	-0.911689000	4.350149000	-1.159225000
C	0.462220000	4.881150000	1.912208000
H	1.145632000	3.329044000	3.259893000
H	-0.303279000	6.188759000	0.364050000
H	0.730459000	5.700387000	2.583617000
C	-2.691266000	0.332848000	2.882953000
H	-3.753777000	0.605620000	2.950045000
H	-2.369661000	-0.087948000	3.850424000
H	-2.110909000	1.260510000	2.730121000
C	-0.628272000	-2.462474000	2.058921000
O	0.530067000	-2.831918000	1.968482000
O	-1.592314000	-3.182619000	2.632694000
C	-1.222297000	-4.475286000	3.134264000
H	-0.866896000	-5.119347000	2.316099000
H	-0.427589000	-4.385254000	3.889144000
H	-2.129666000	-4.896017000	3.582651000

8. NMR spectra

1H NMR (300 MHz, CDCl3)

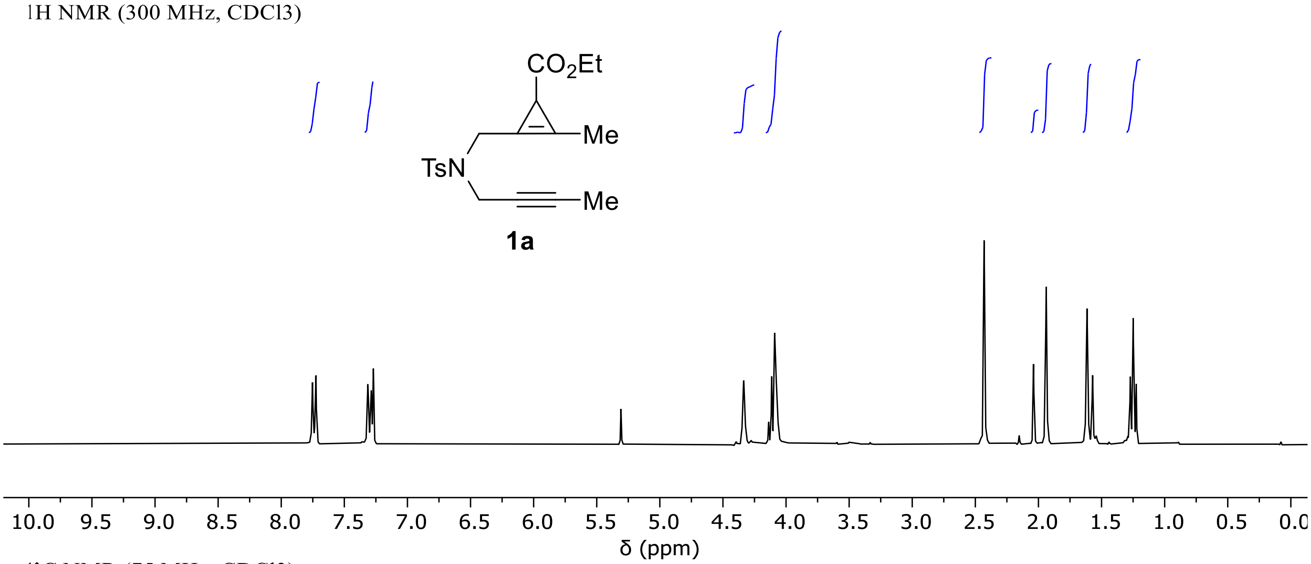

13C NMR (75 MHz, CDCl3)

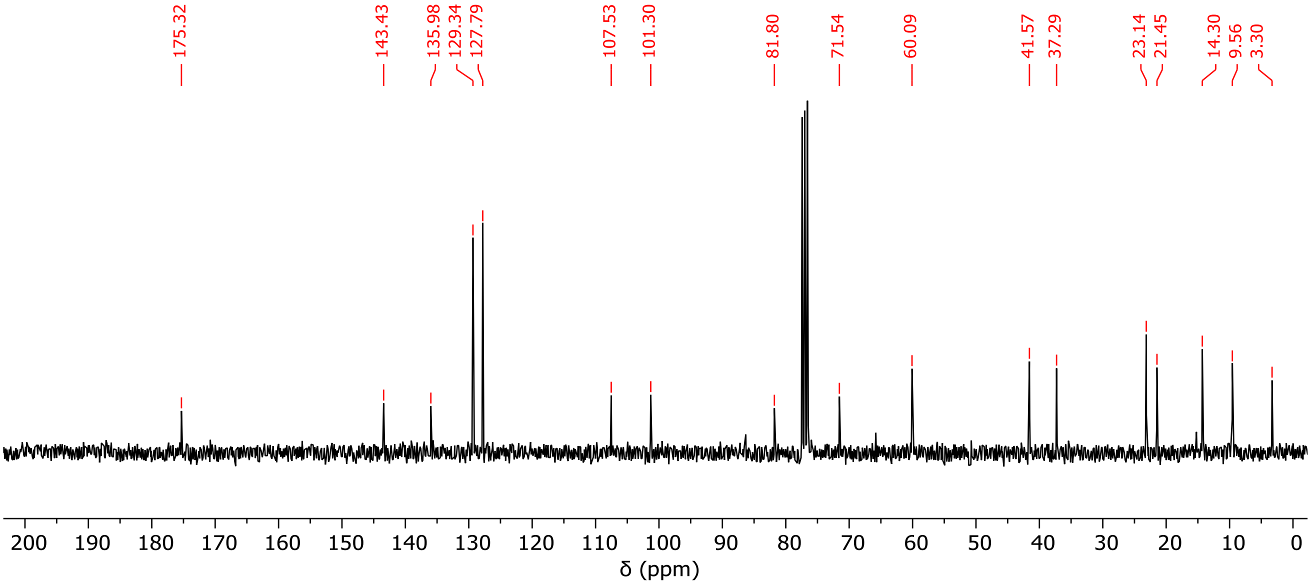

DEPT NMR (75 MHz, CDCl3)

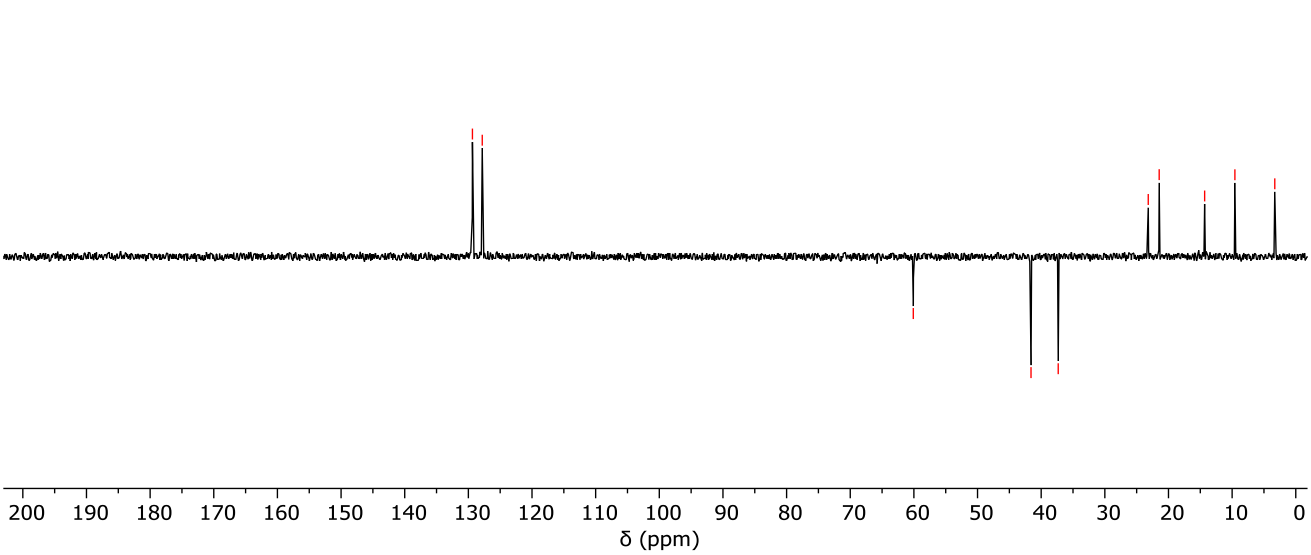

1H NMR (300 MHz, CDCl3)

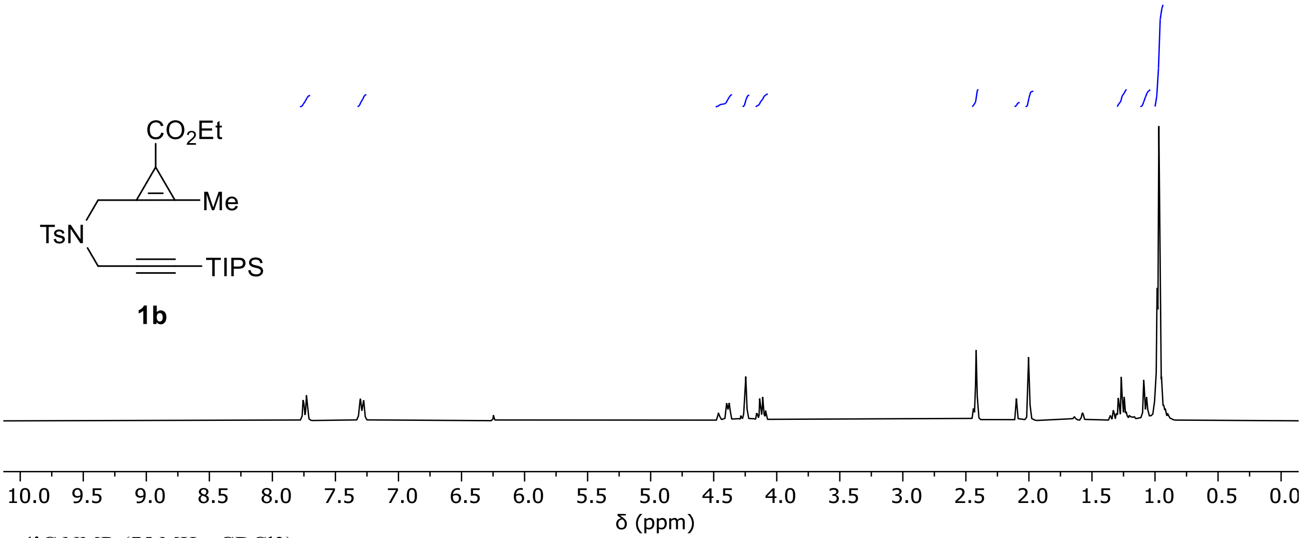

13C NMR (75 MHz, CDCl3)

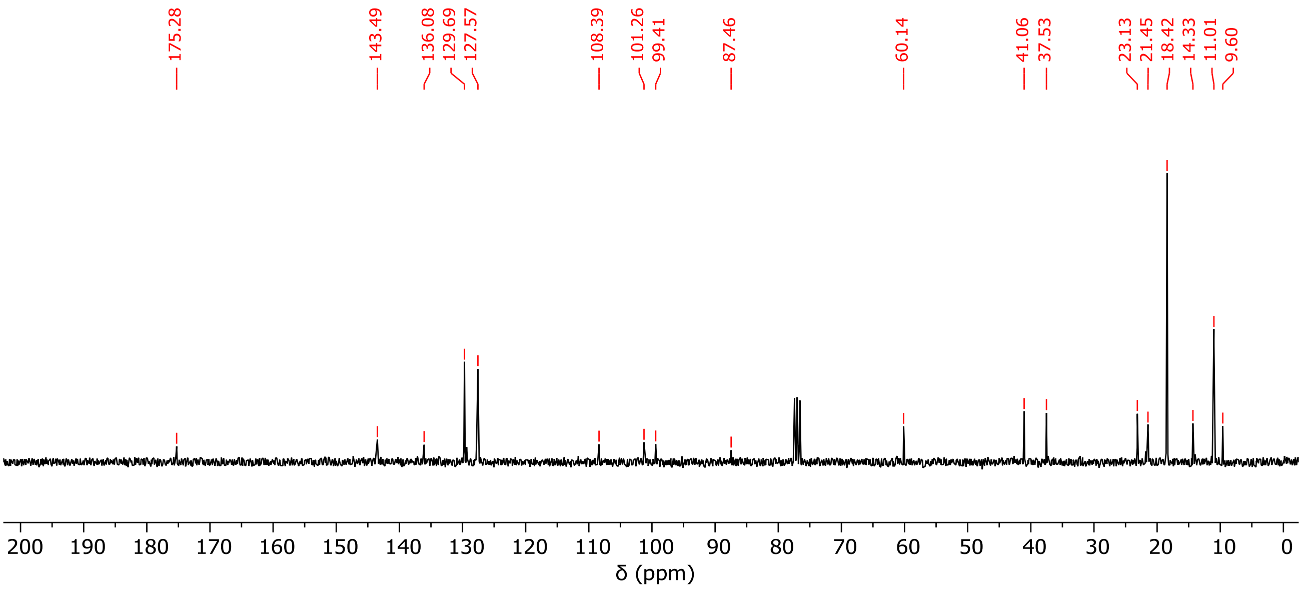

DEPT NMR (75 MHz, CDCl3)

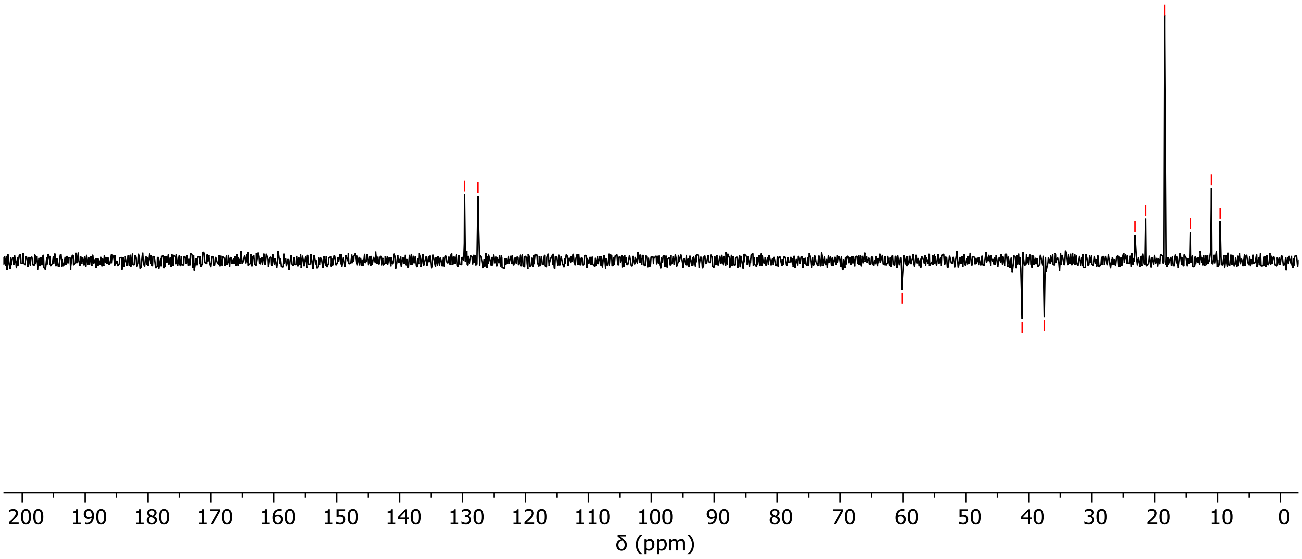

1H NMR (300 MHz, CDCl3)

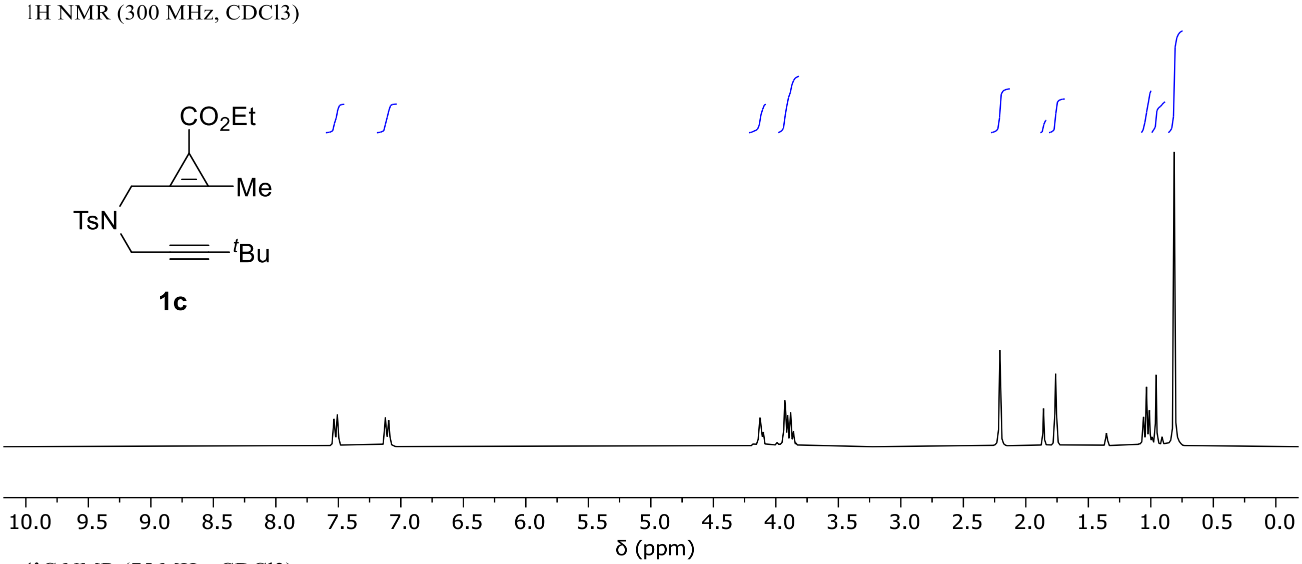

13C NMR (75 MHz, CDCl3)

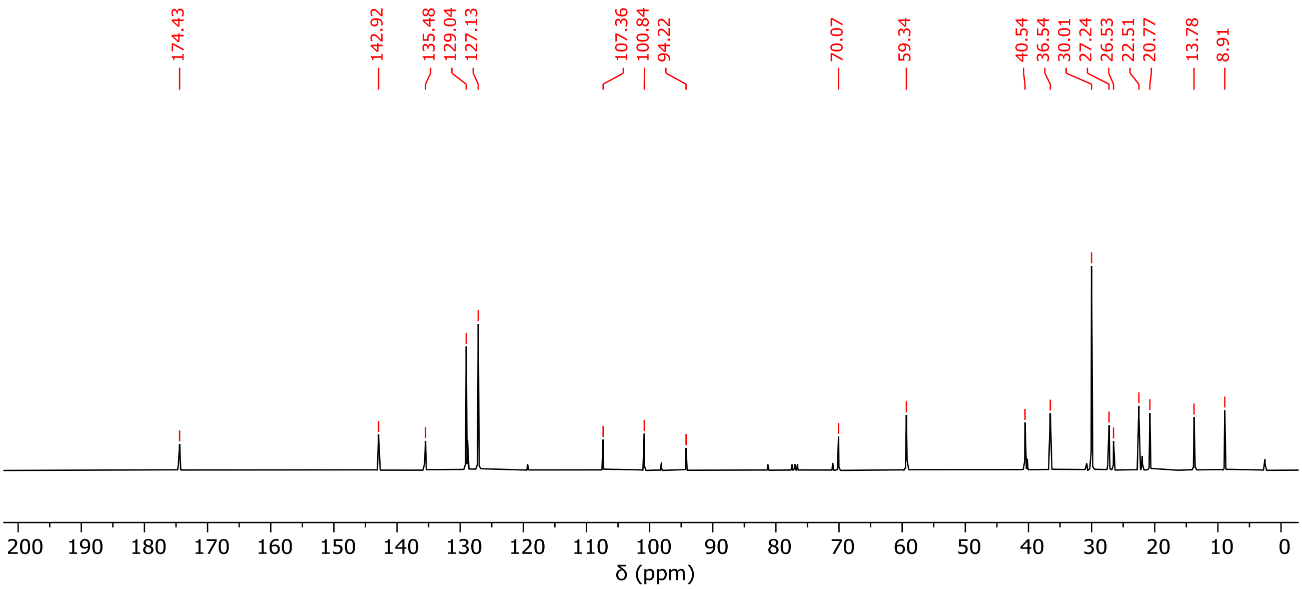

DEPT NMR (75 MHz, CDCl3)

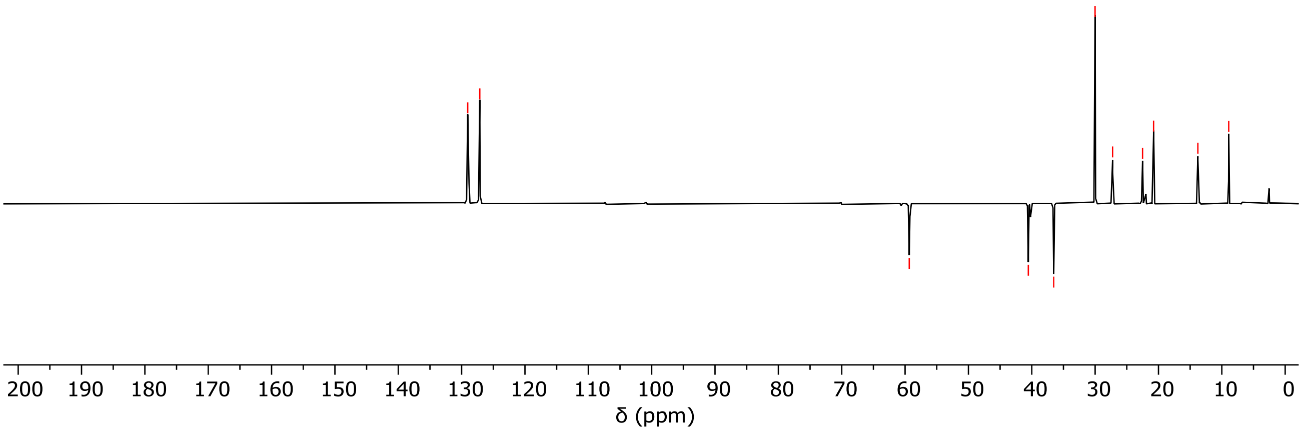

1H NMR (300 MHz, CDCl3)

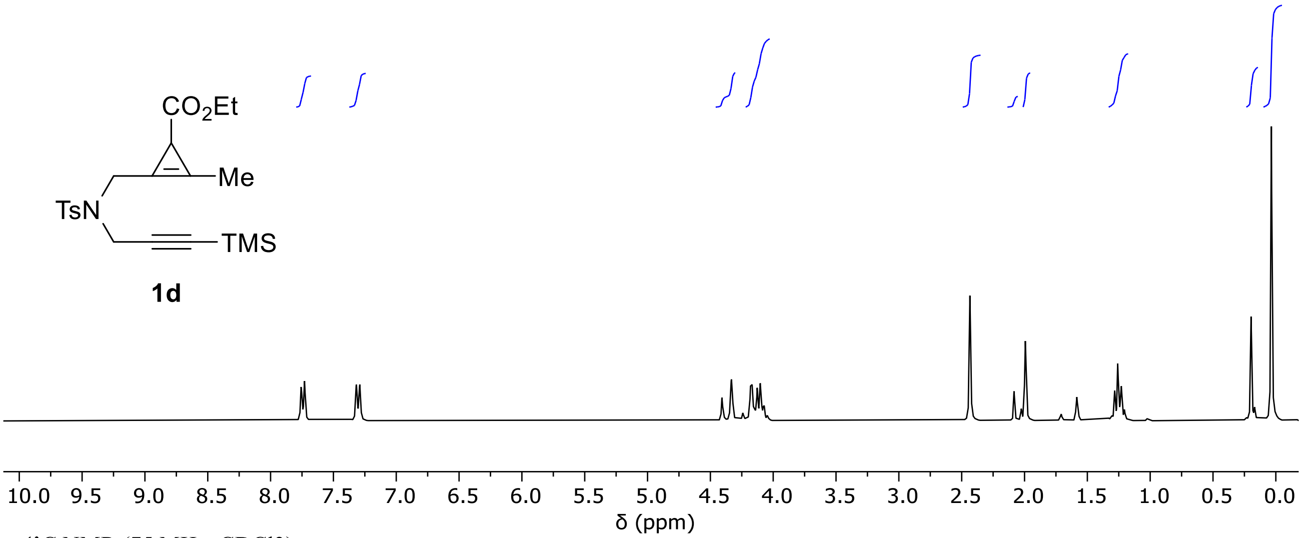

13C NMR (75 MHz, CDCl3)

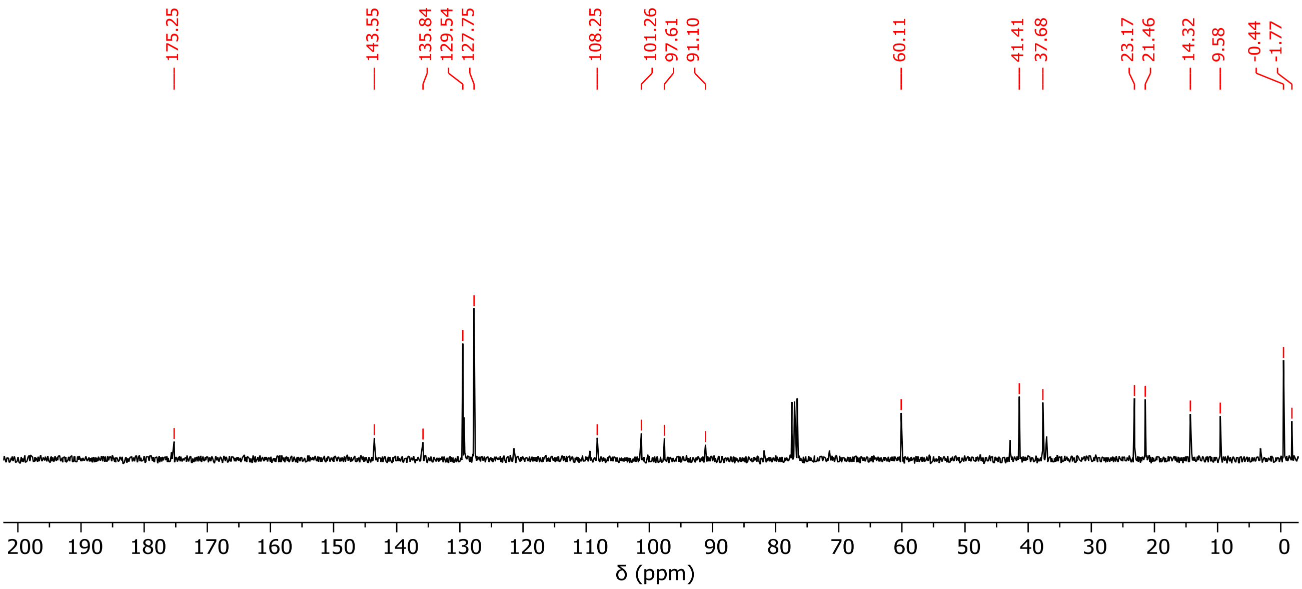

DEPT NMR (75 MHz, CDCl3)

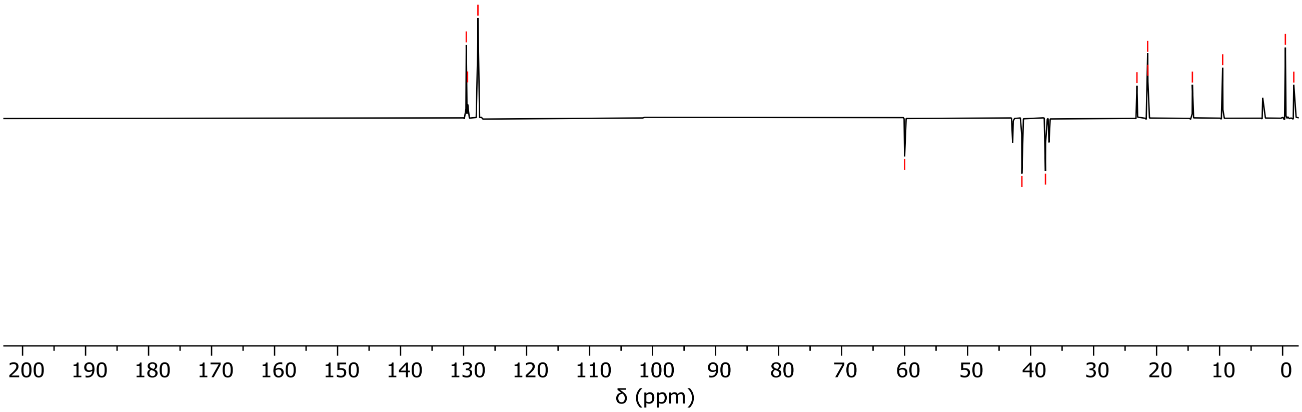

1H NMR (300 MHz, CDCl3)

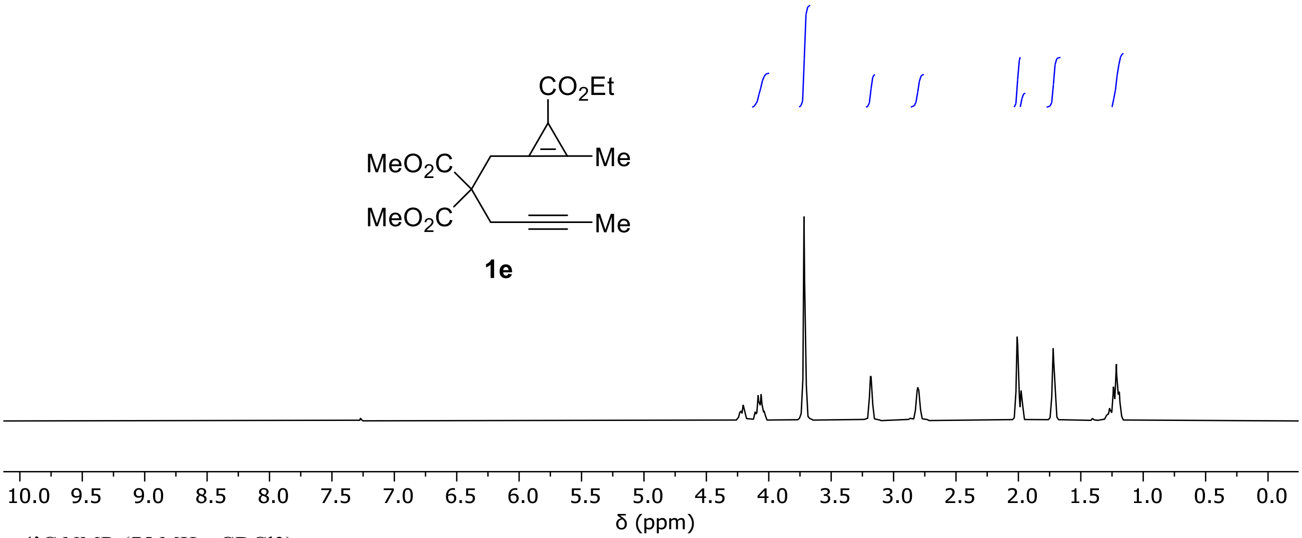

13C NMR (75 MHz, CDCl3)

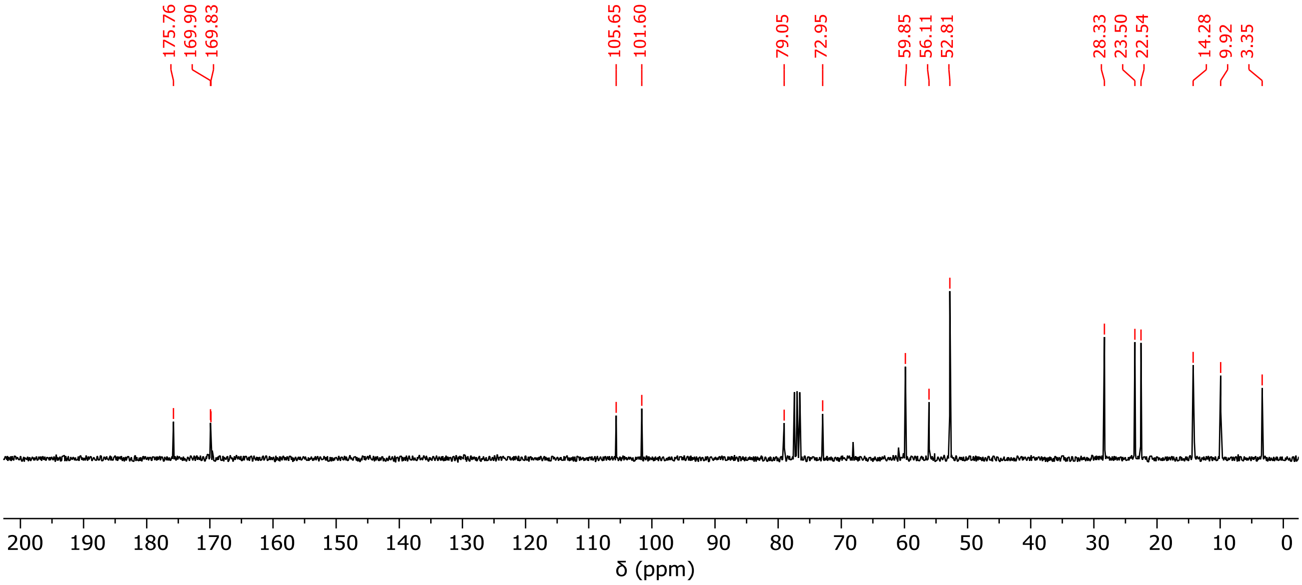

DEPT NMR (75 MHz, CDCl3)

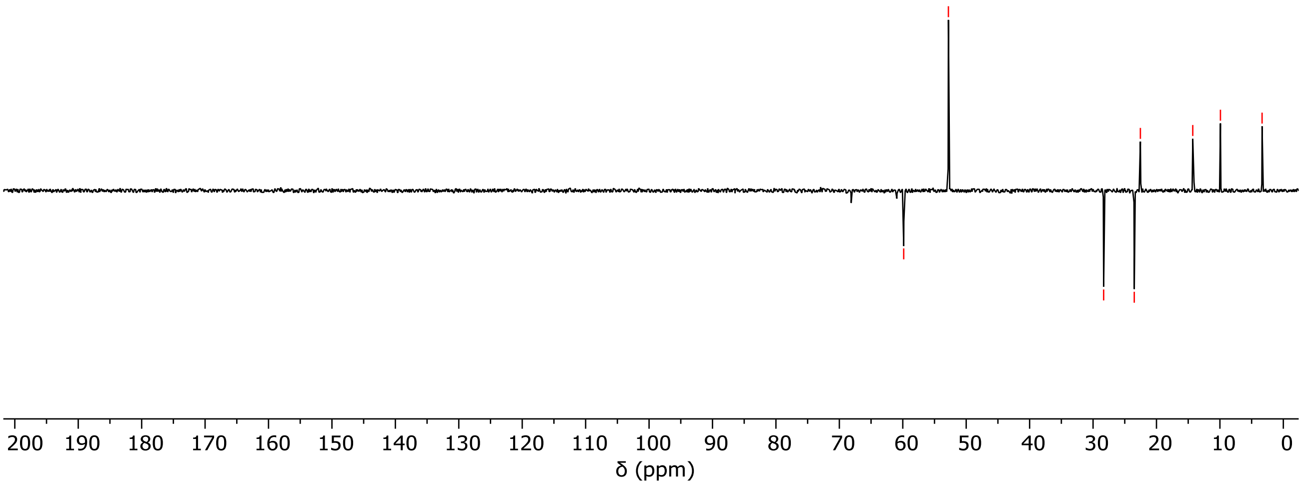

1H NMR (300 MHz, CDCl3)

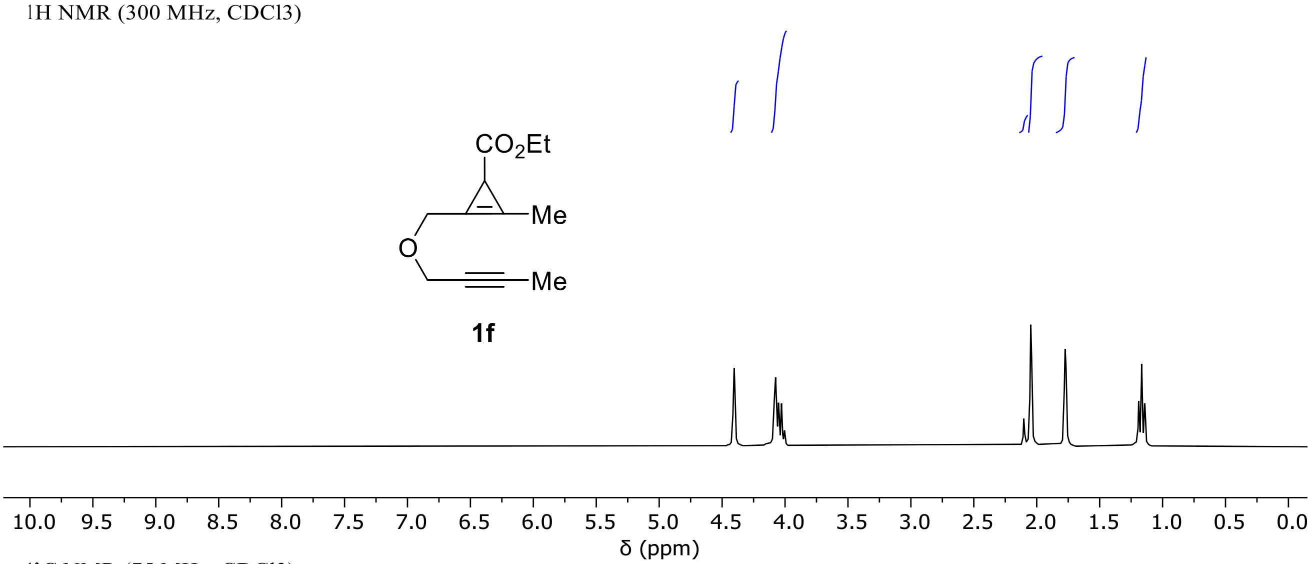

13C NMR (75 MHz, CDCl3)

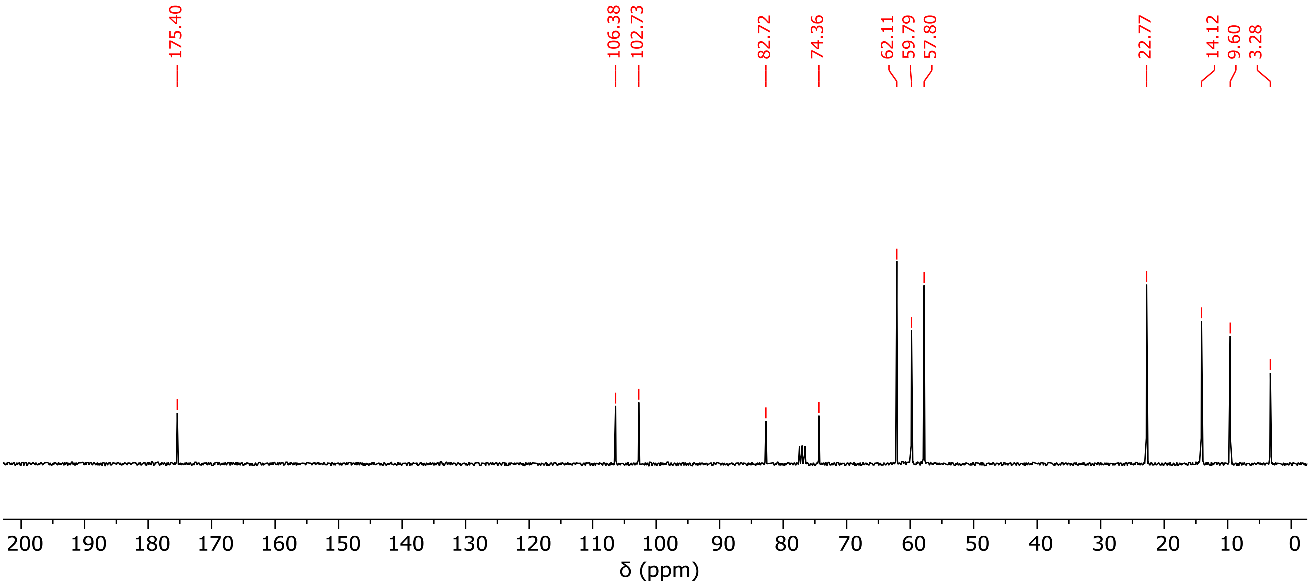

DEPT NMR (75 MHz, CDCl3)

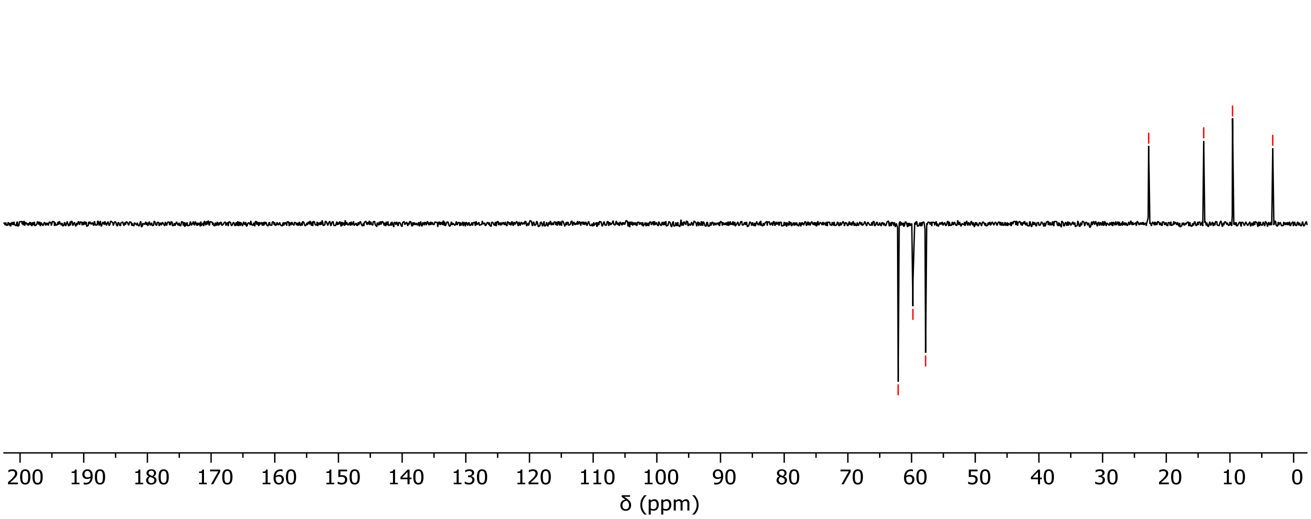

1H NMR (300 MHz, CDCl3)

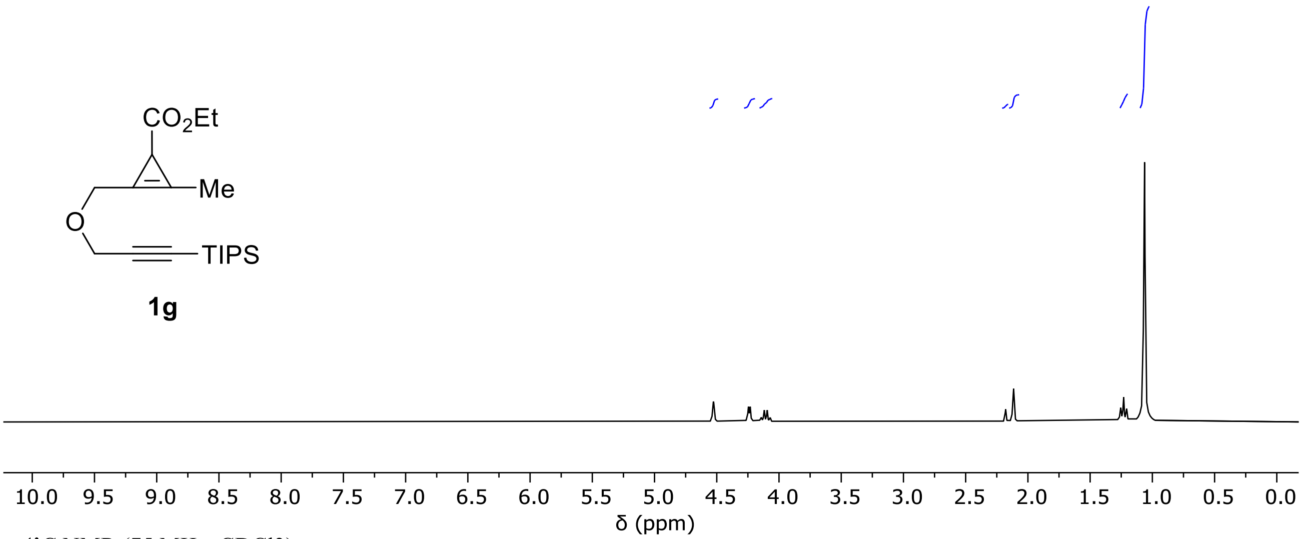

13C NMR (75 MHz, CDCl3)

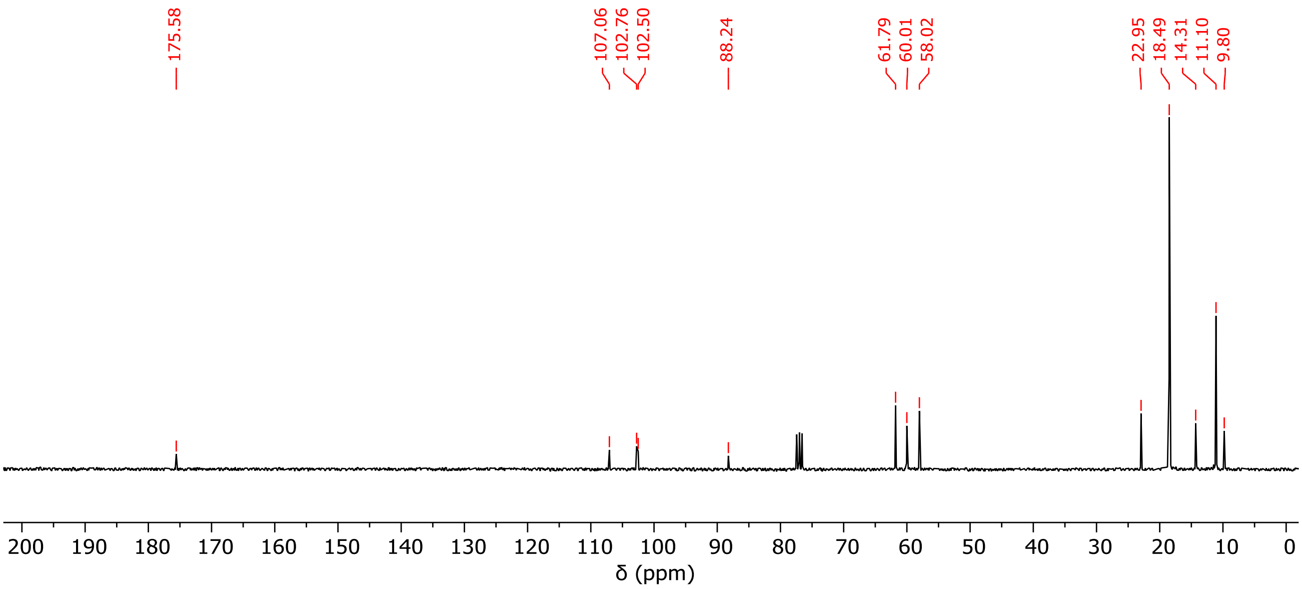

DEPT NMR (75 MHz, CDCl3)

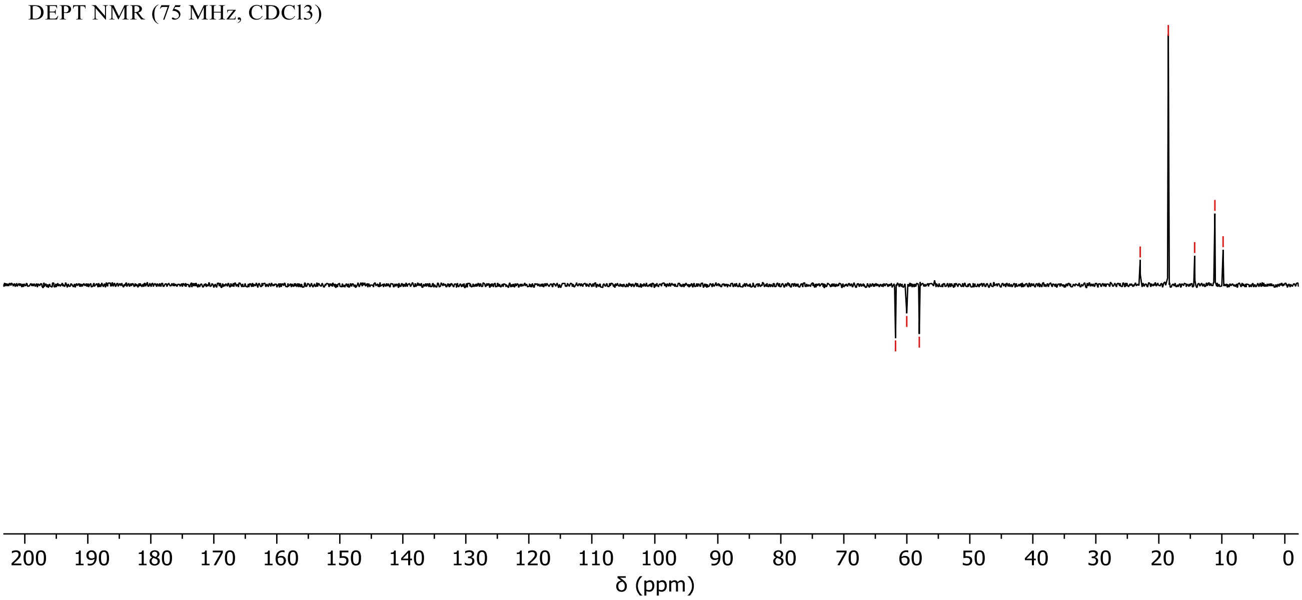

1H NMR (300 MHz, CDCl3)

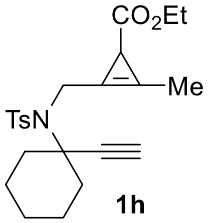

1h

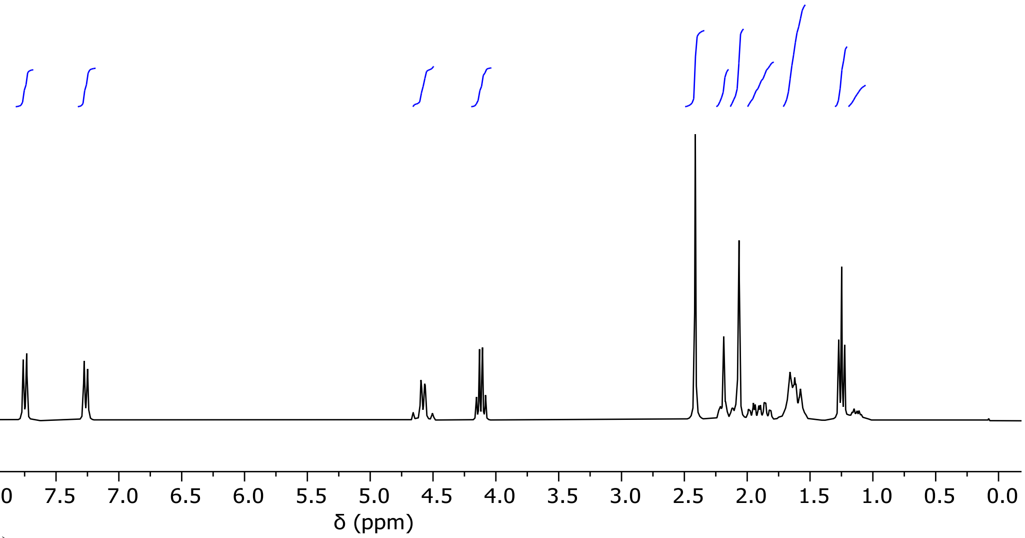

13C NMR (75 MHz, CDCl3)

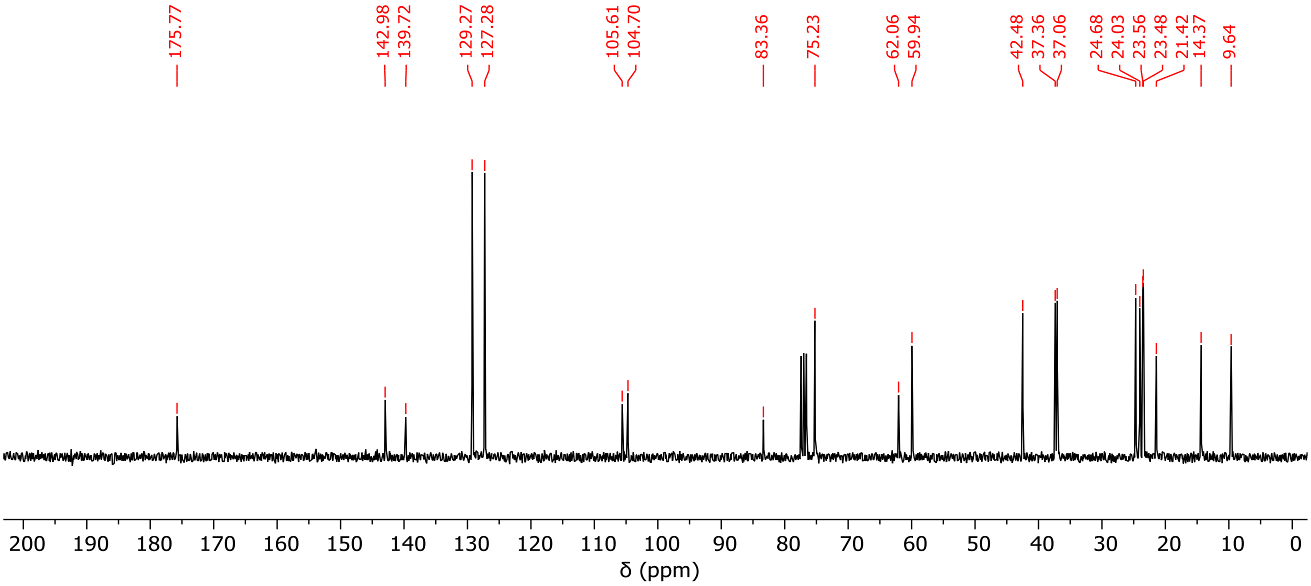

DEPT NMR (75 MHz, CDCl3)

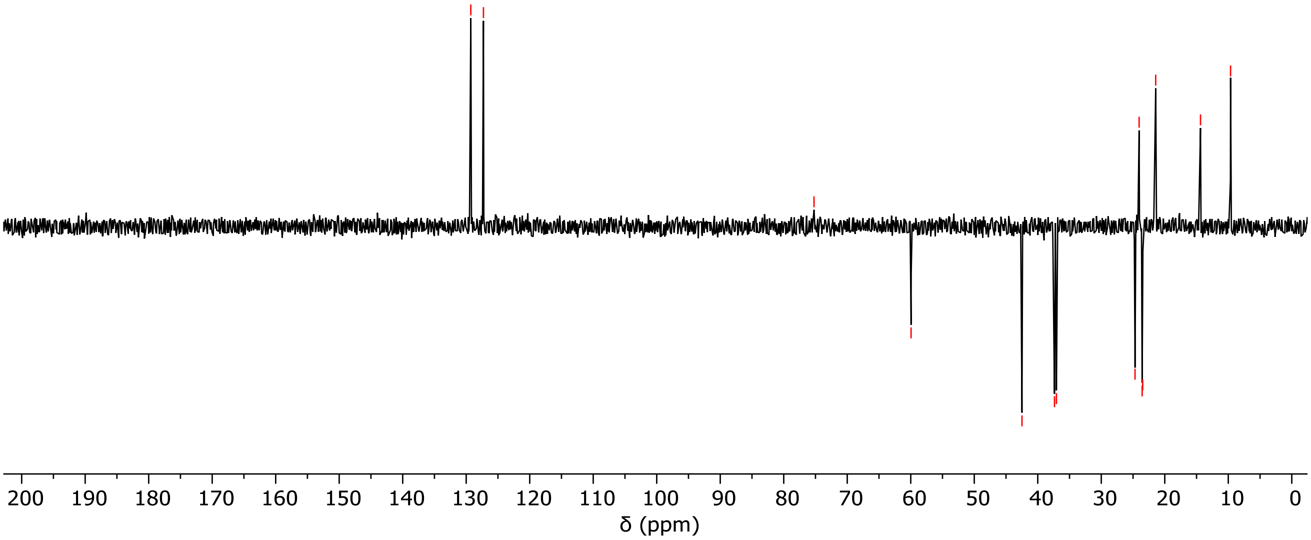

1H NMR (300 MHz, CDCl3)

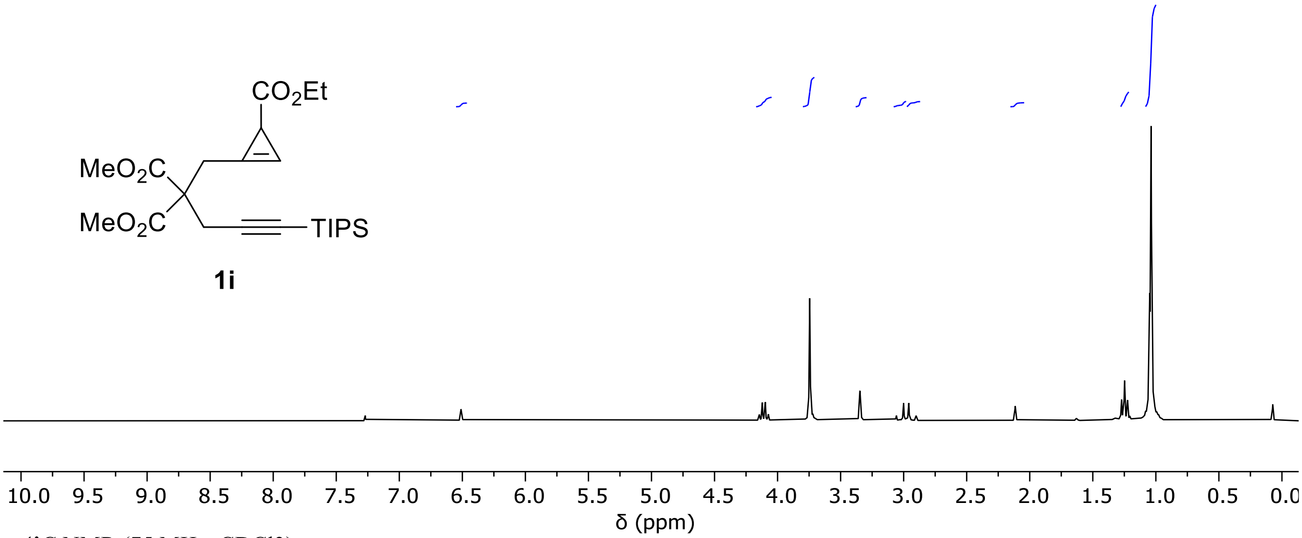

13C NMR (75 MHz, CDCl3)

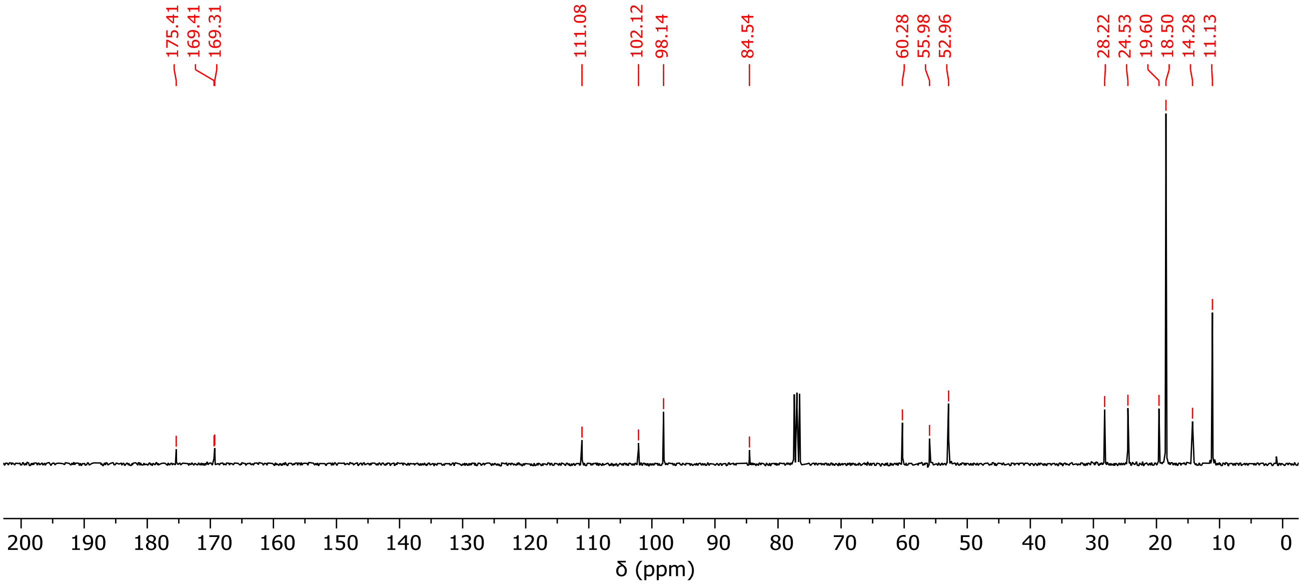

DEPT NMR (75 MHz, CDCl3)

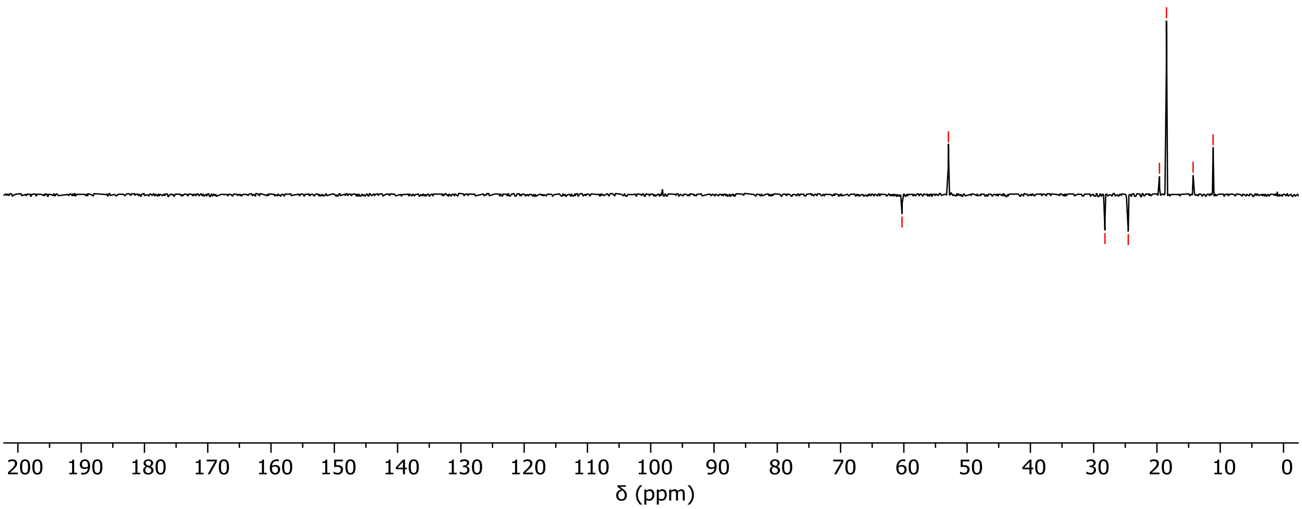

1H NMR (300 MHz, CDCl3)

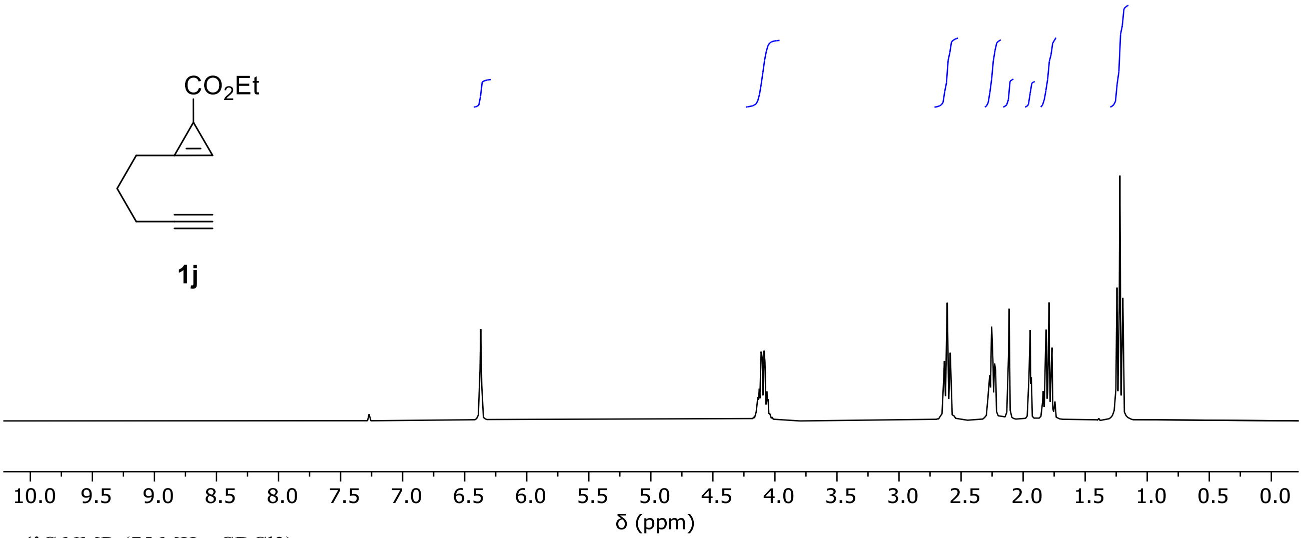

1j

13C NMR (75 MHz, CDCl3)

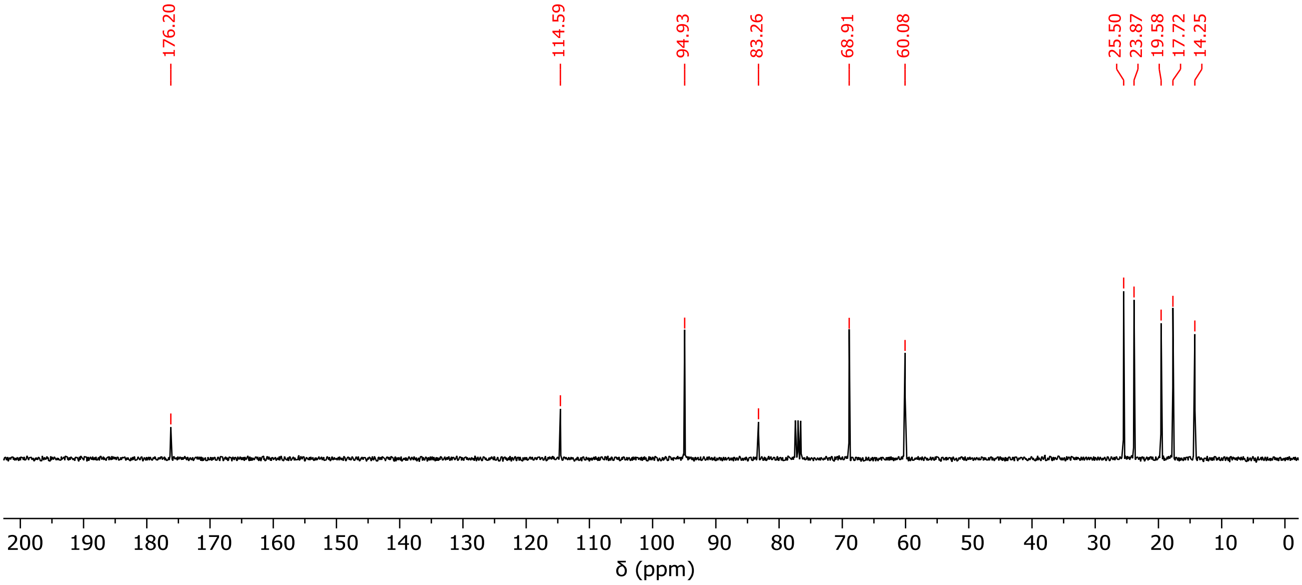

DEPT NMR (75 MHz, CDCl3)

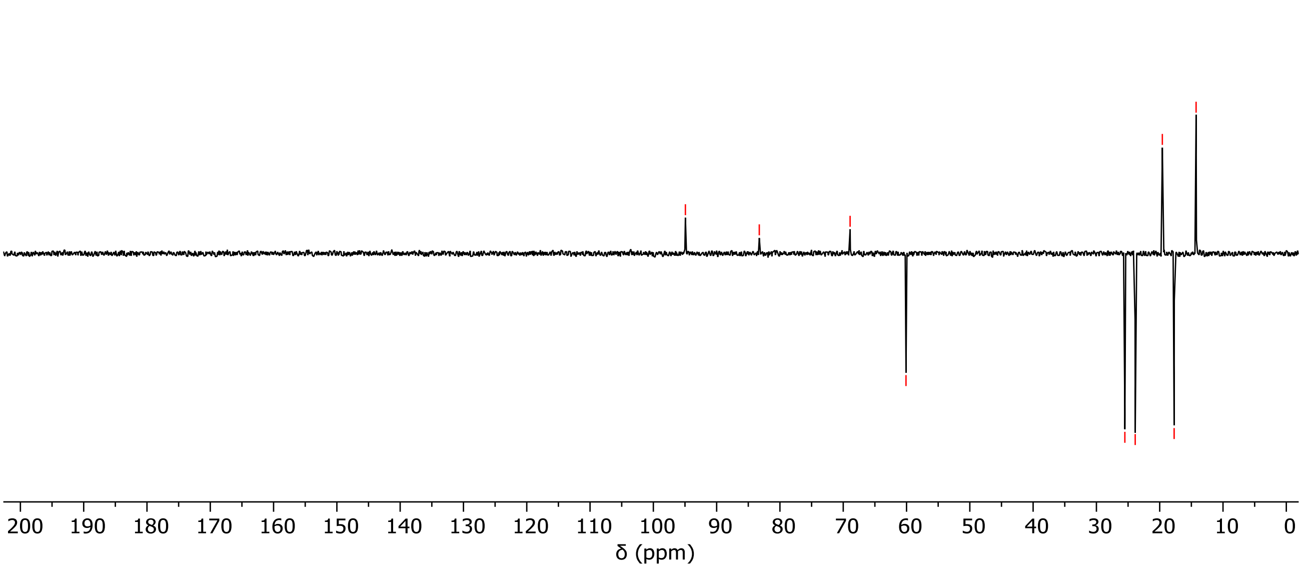

1H NMR (300 MHz, CDCl3)

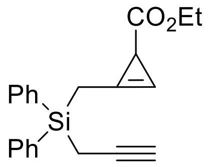

1k

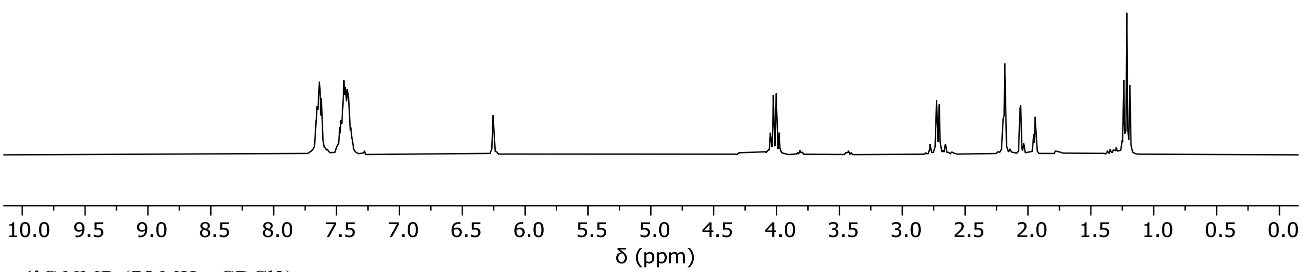

13C NMR (75 MHz, CDCl3)

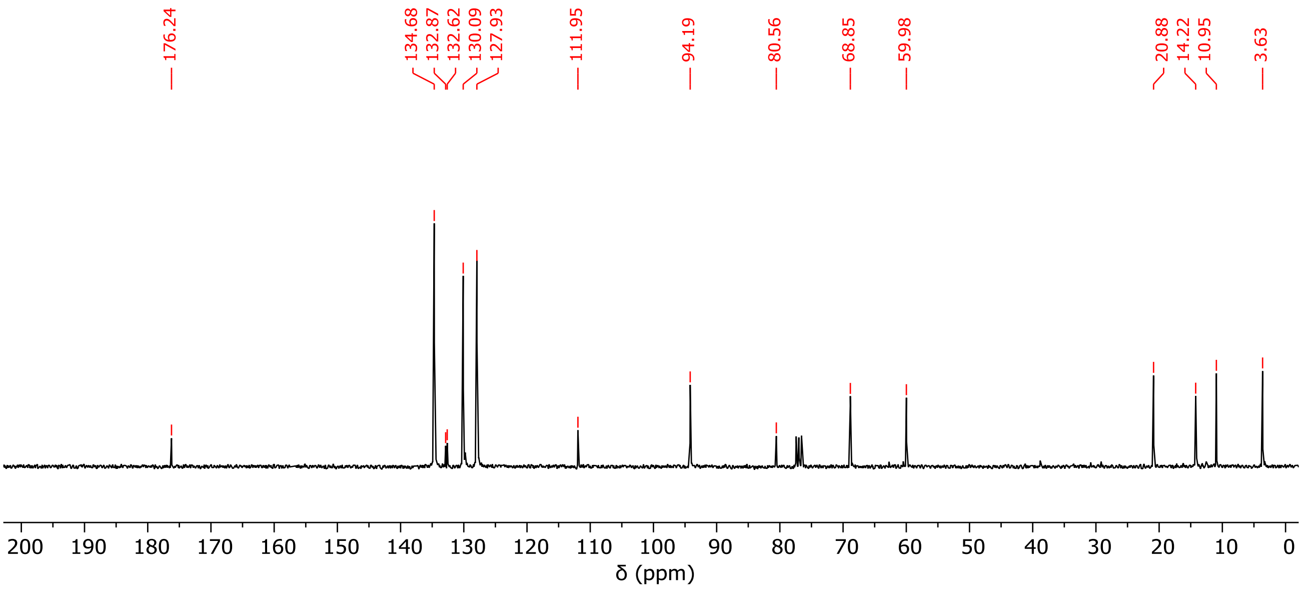

DEPT NMR (75 MHz, CDCl3)

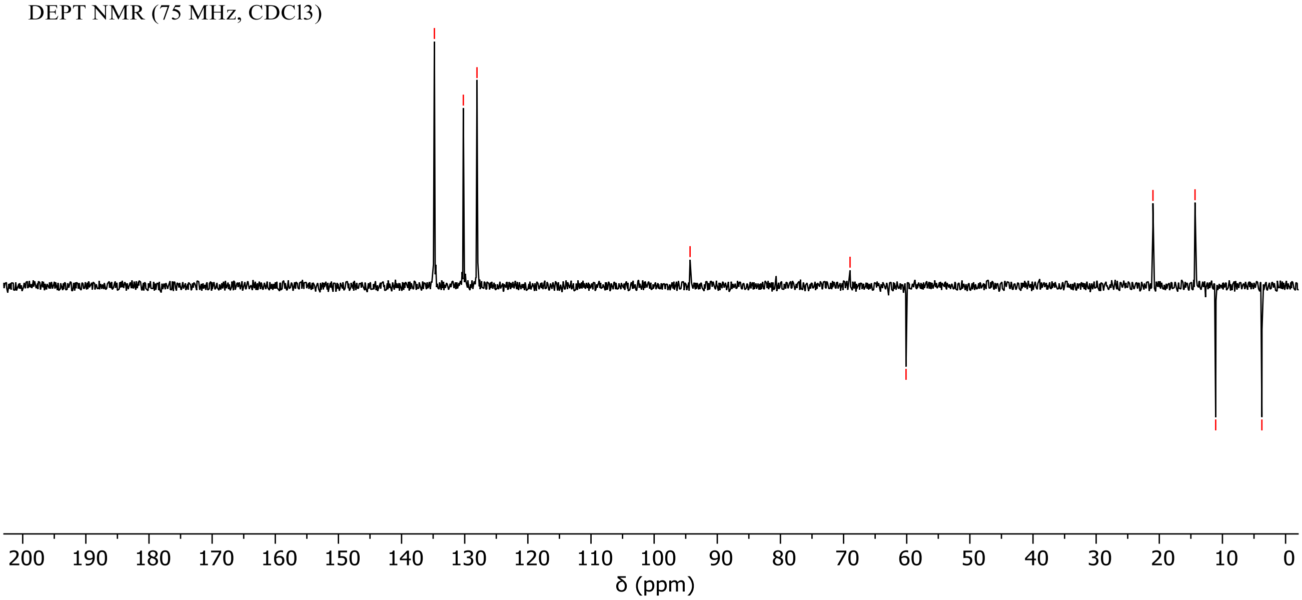

1H NMR (300 MHz, CDCl3)

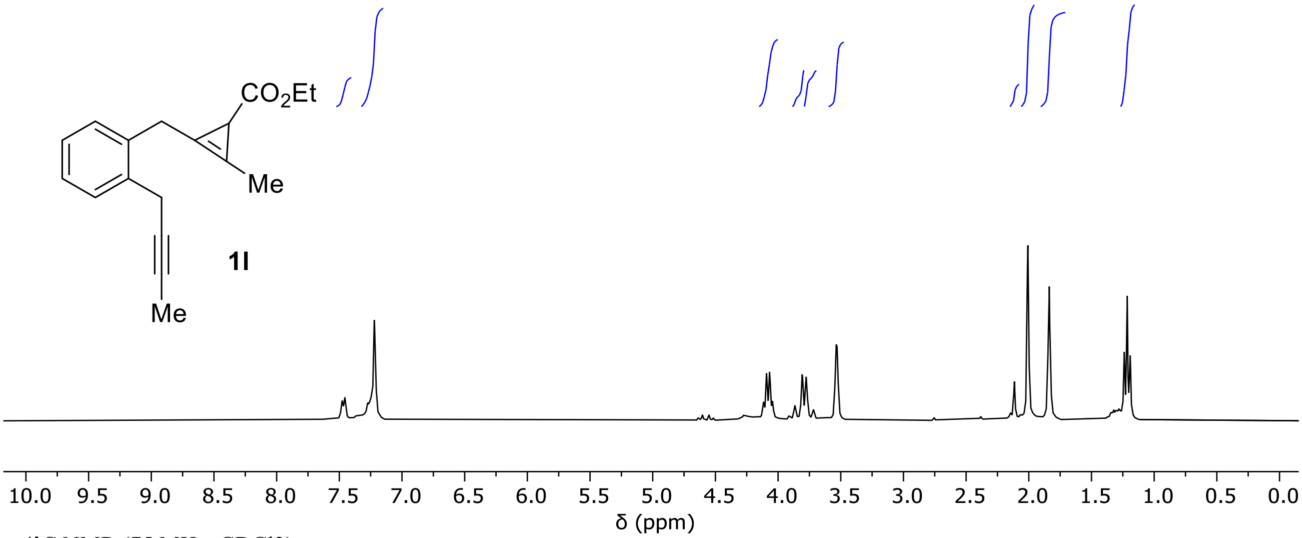

13C NMR (75 MHz, CDCl3)

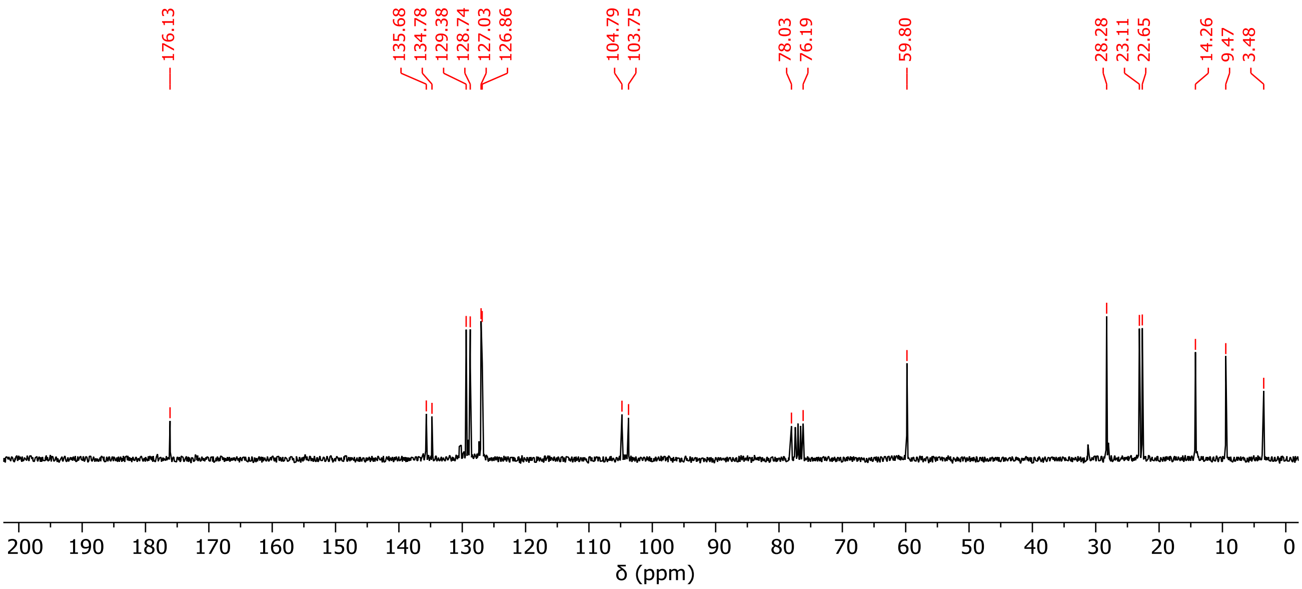

DEPT NMR (75 MHz, CDCl3)

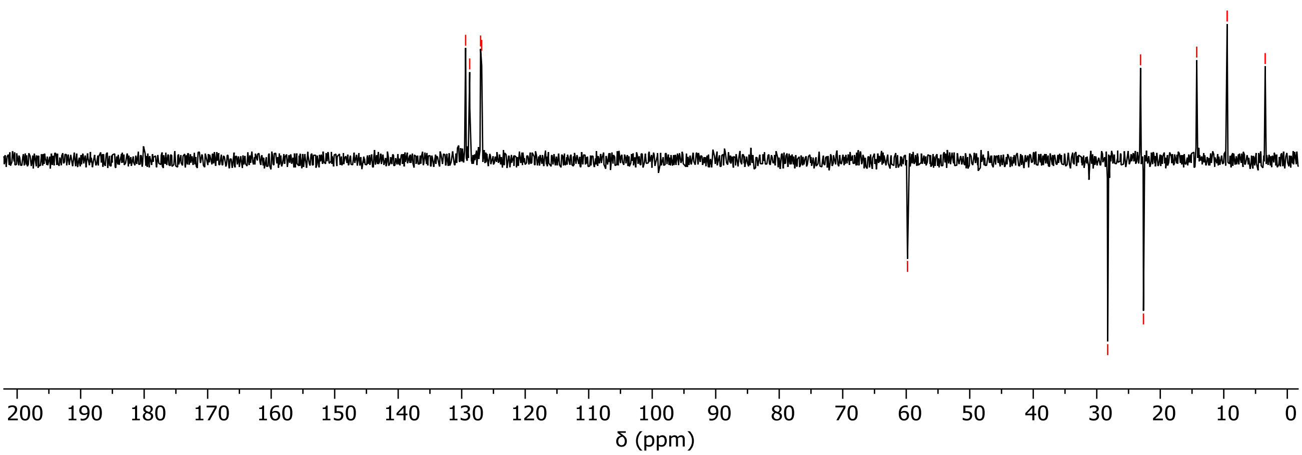

1H NMR (300 MHz, CDCl3)

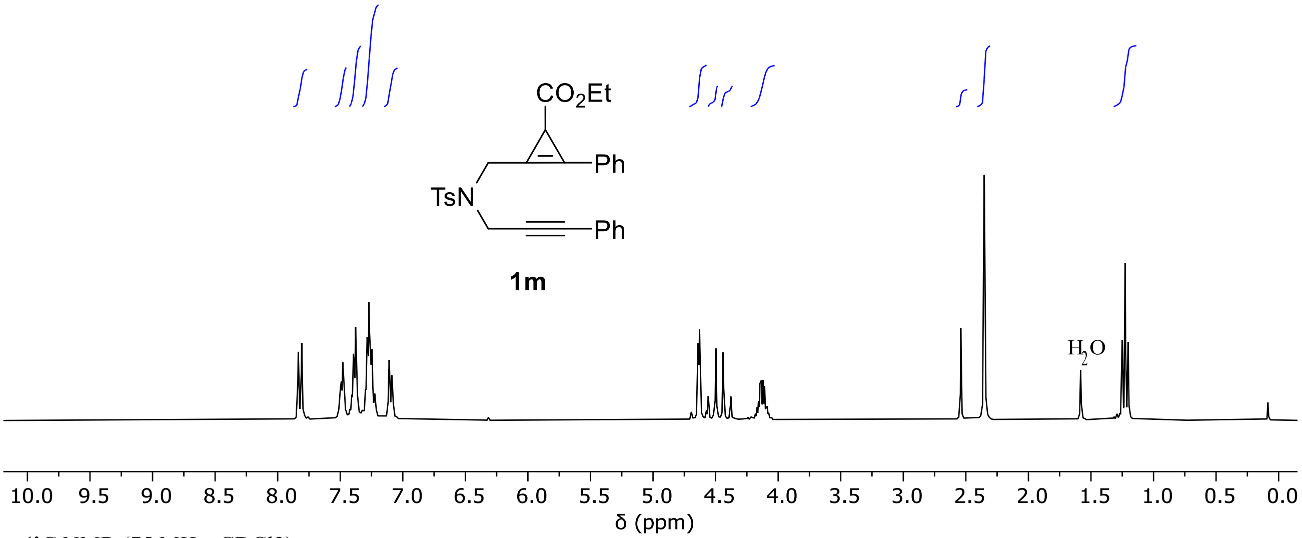

13C NMR (75 MHz, CDCl3)

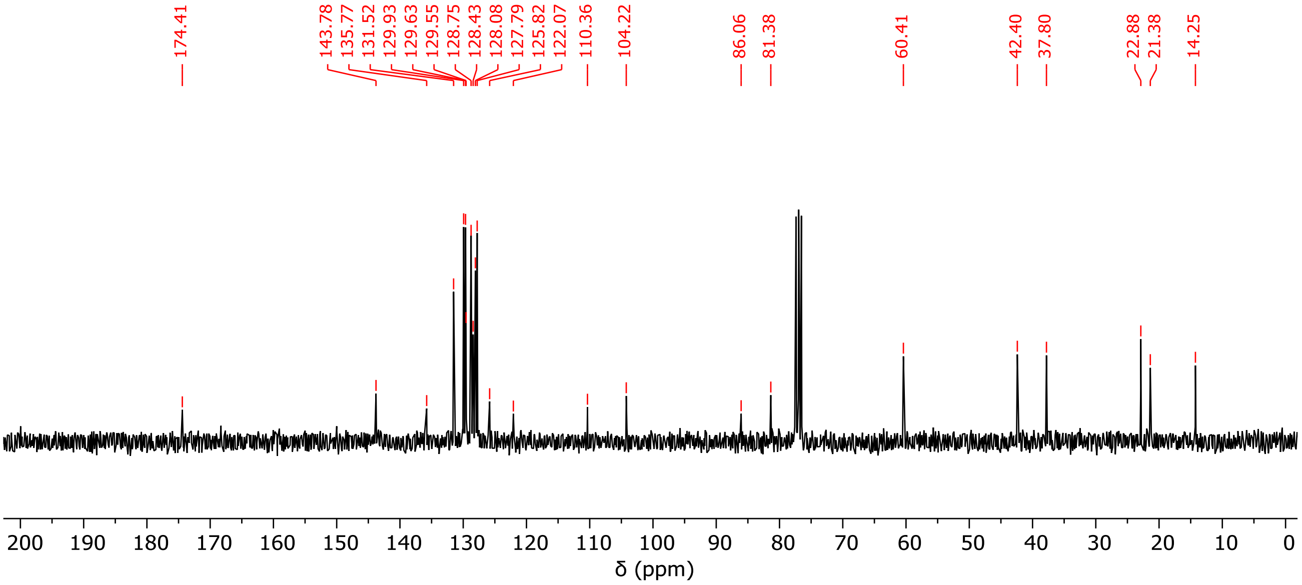

DEPT NMR (75 MHz, CDCl3)

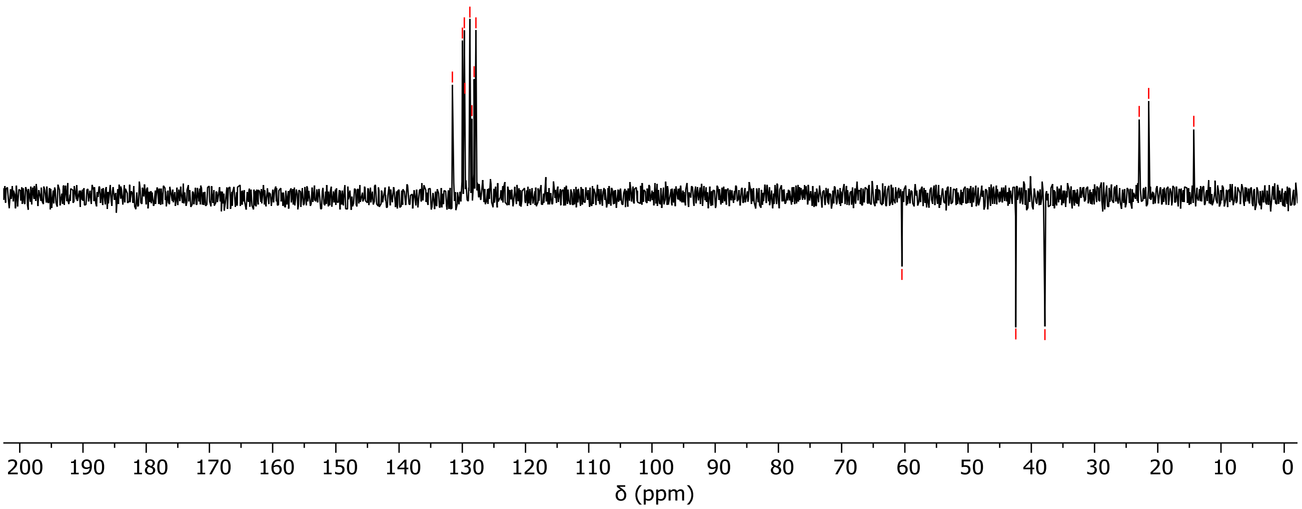

1H NMR (300 MHz, CDCl3)

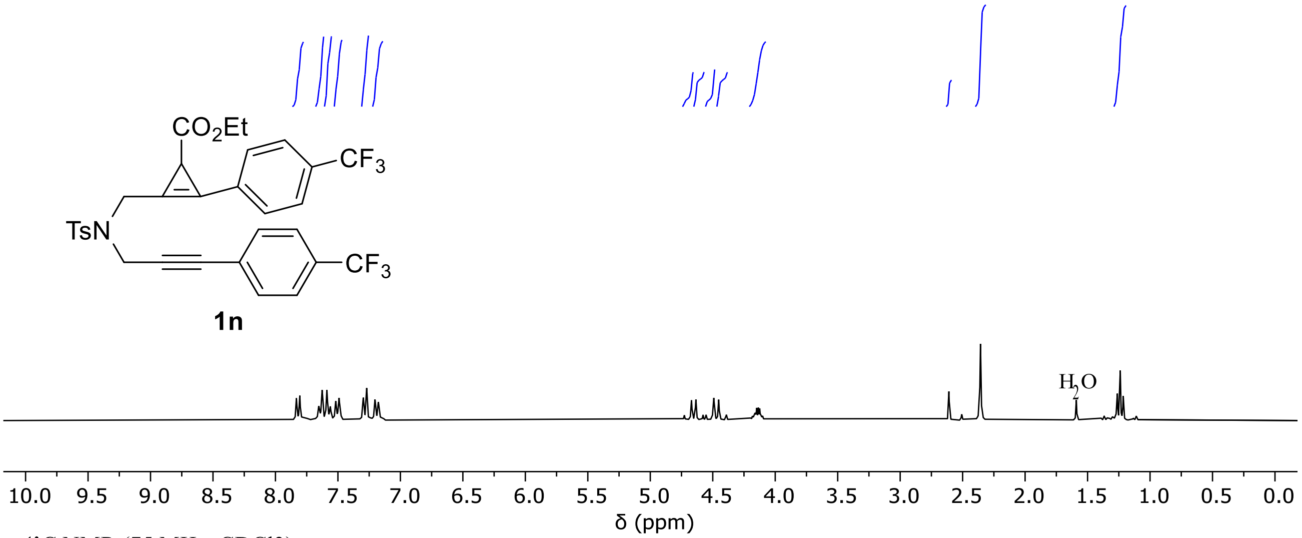

13C NMR (75 MHz, CDCl3)

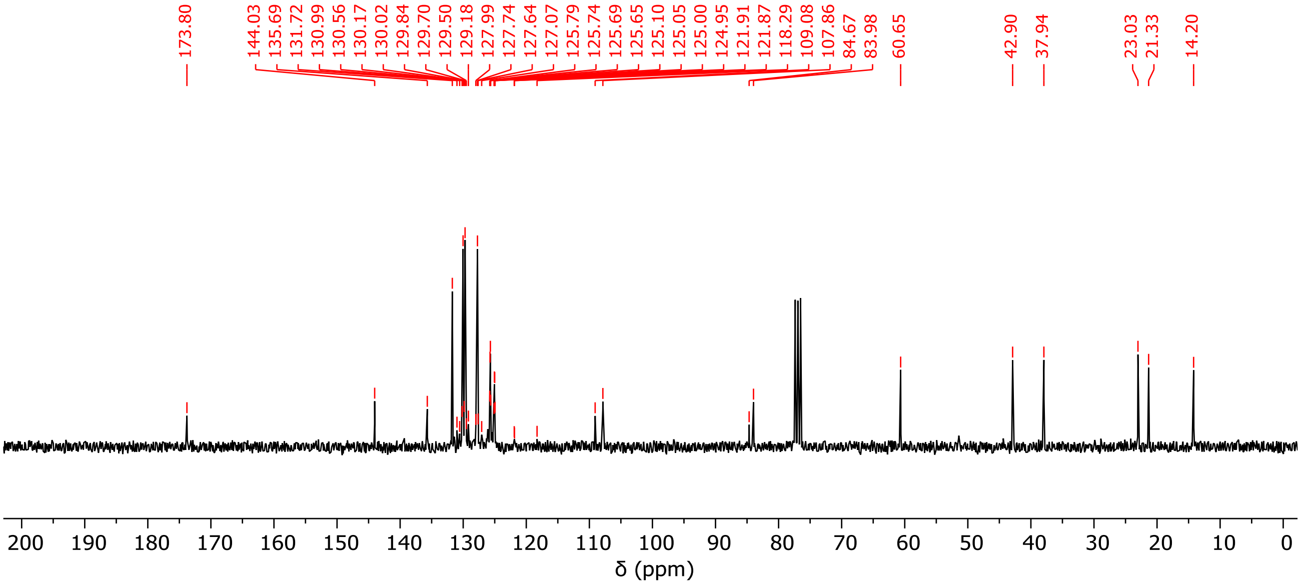

DEPT NMR (75 MHz, CDCl3)

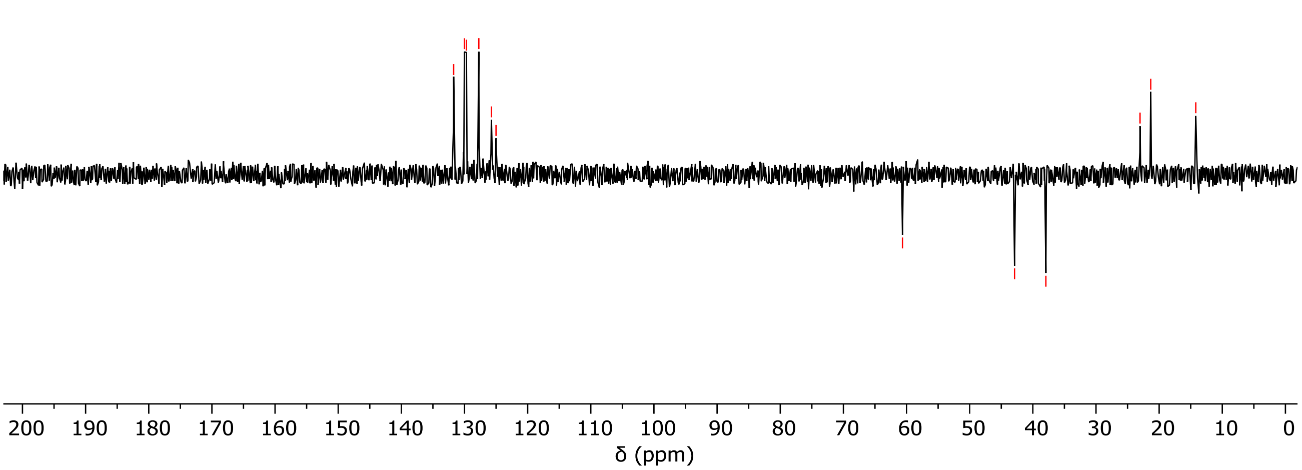

19F NMR (282 MHz, CDCl3)

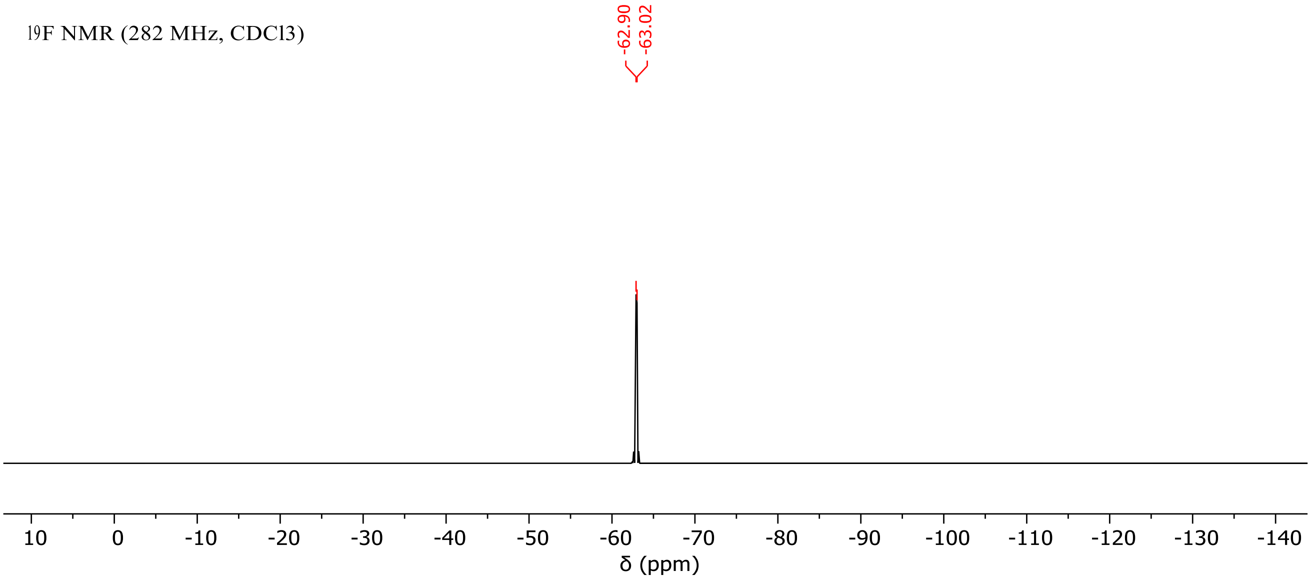

1H NMR (500 MHz, CDCl3)

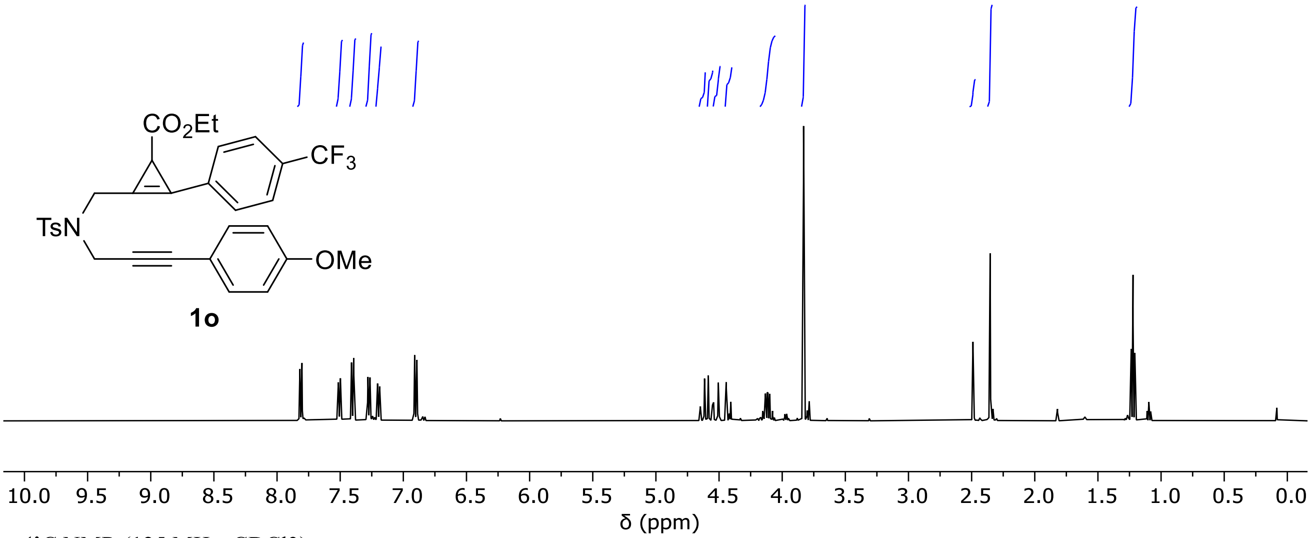

13C NMR (125 MHz, CDCl3)

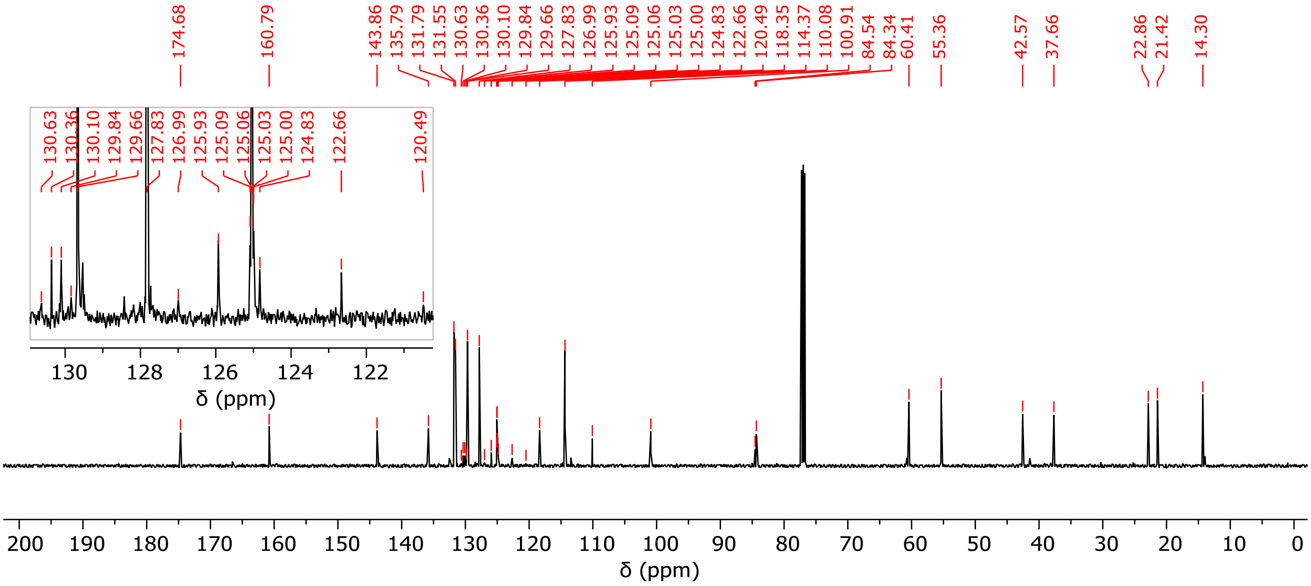

DEPT NMR (125 MHz, CDCl3)

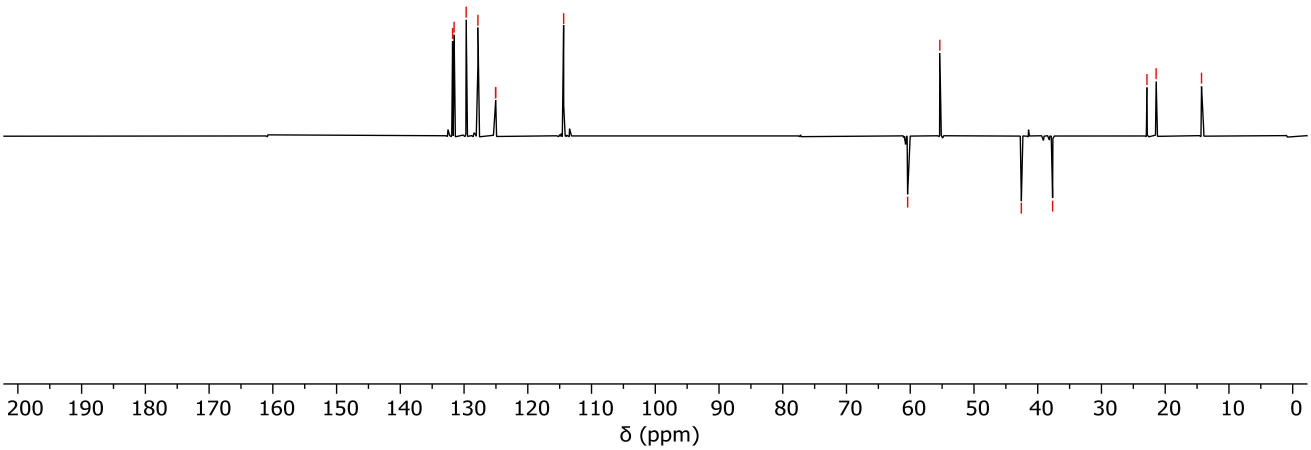

19F NMR (470 MHz, CDCl3)

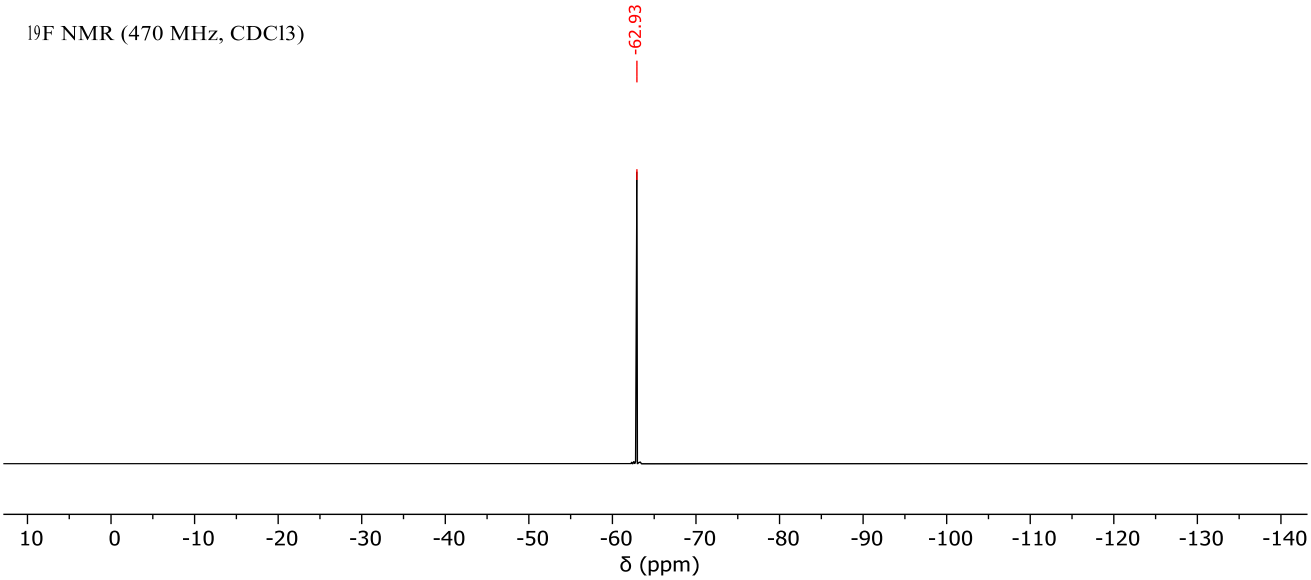

1H NMR (300 MHz, CDCl3)

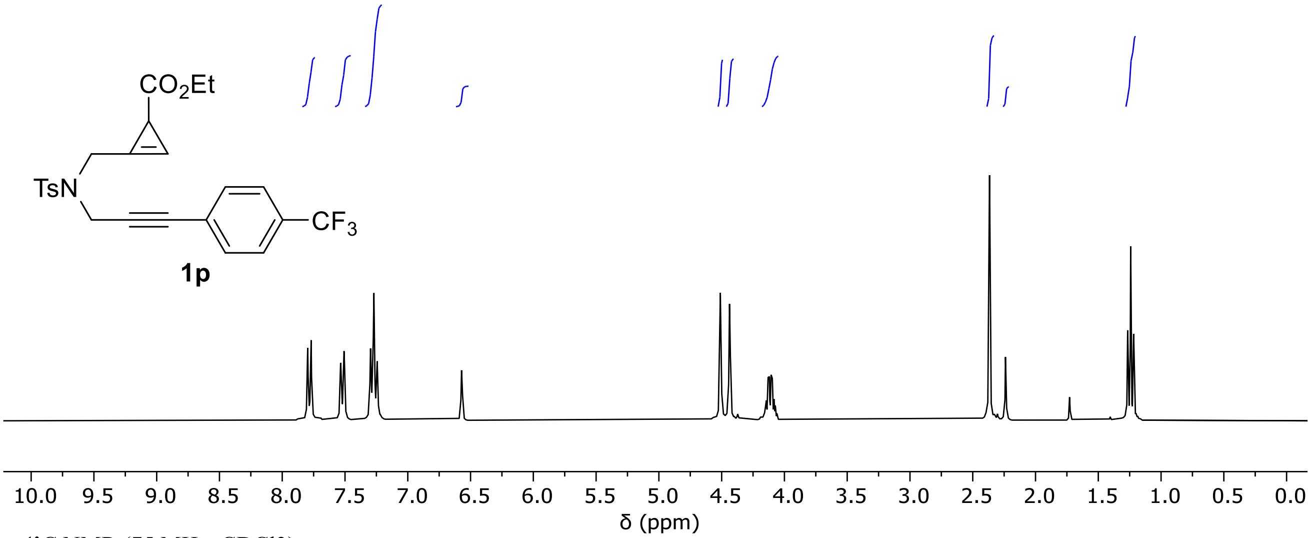

13C NMR (75 MHz, CDCl3)

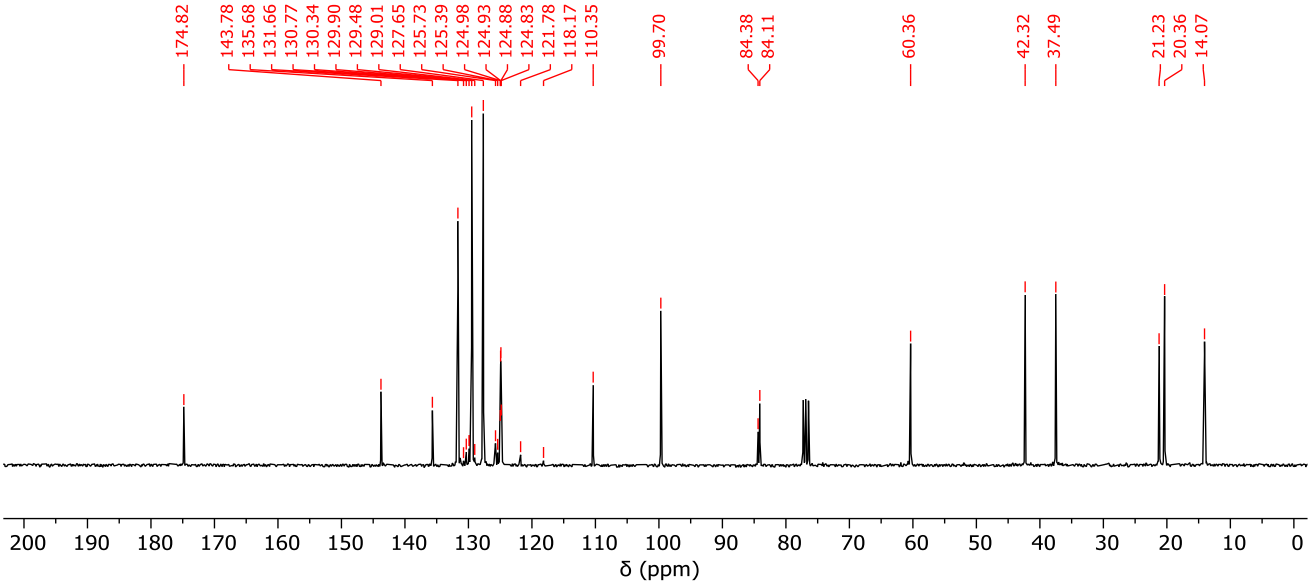

DEPT NMR (75 MHz, CDCl3)

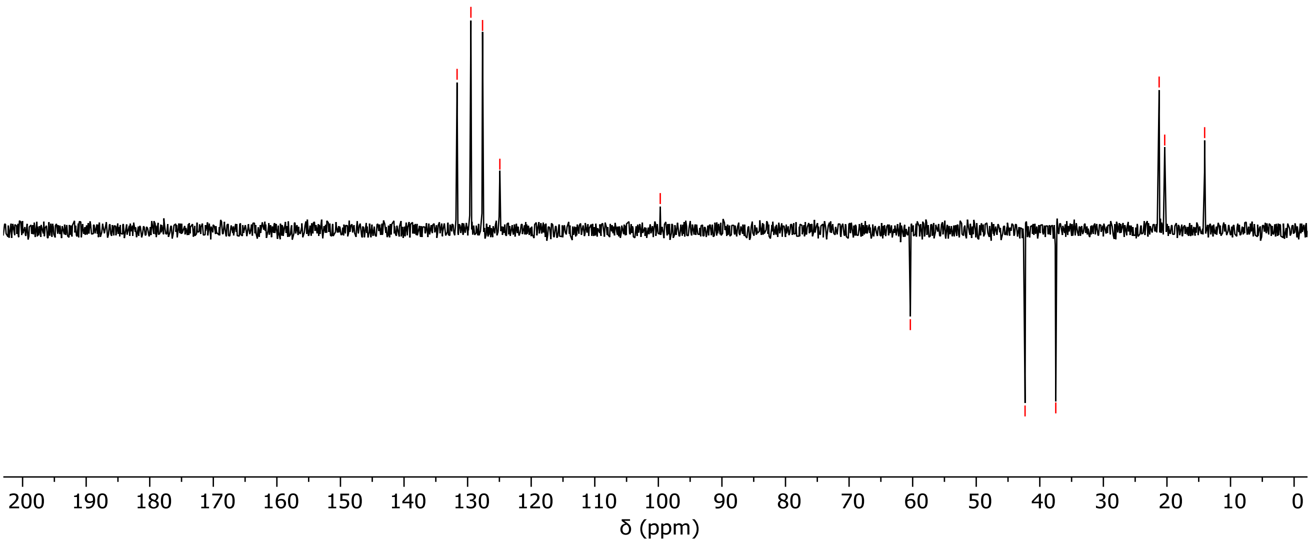

19F NMR (282 MHz, CDCl3)

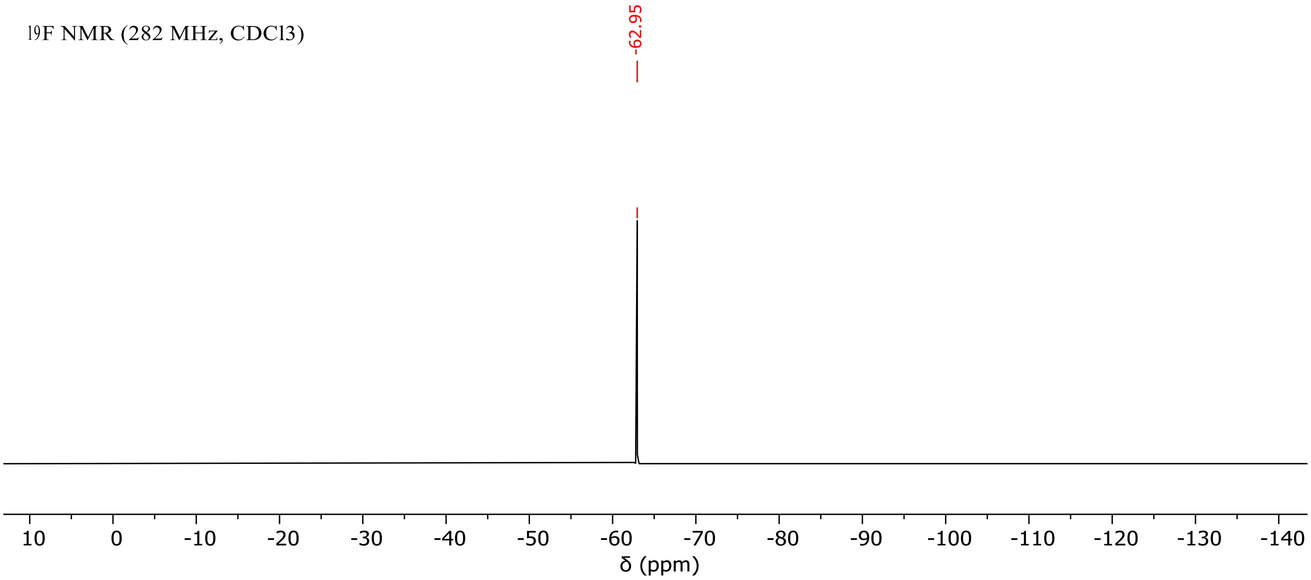

1H NMR (300 MHz, CDCl3)

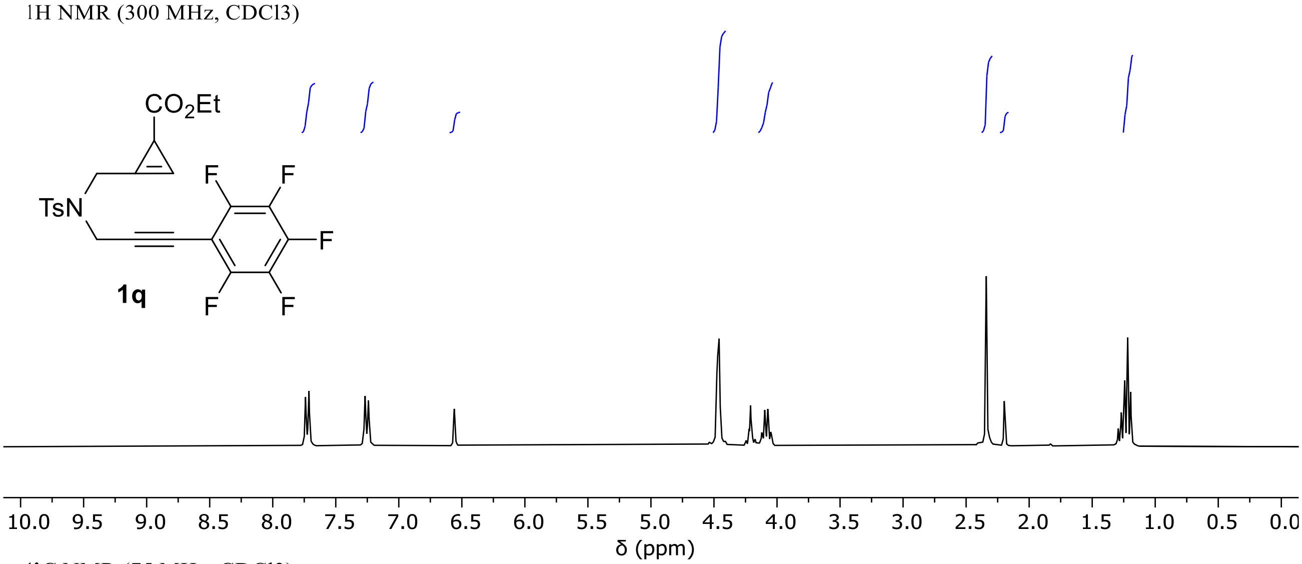

13C NMR (75 MHz, CDCl3)

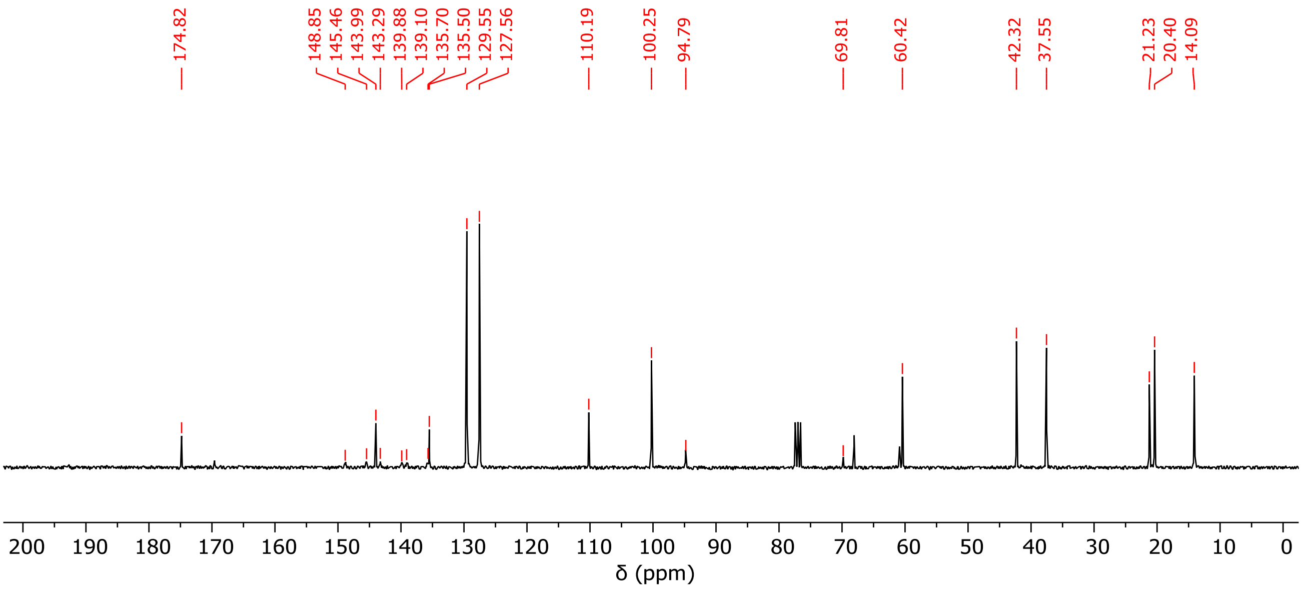

DEPT NMR (75 MHz, CDCl3)

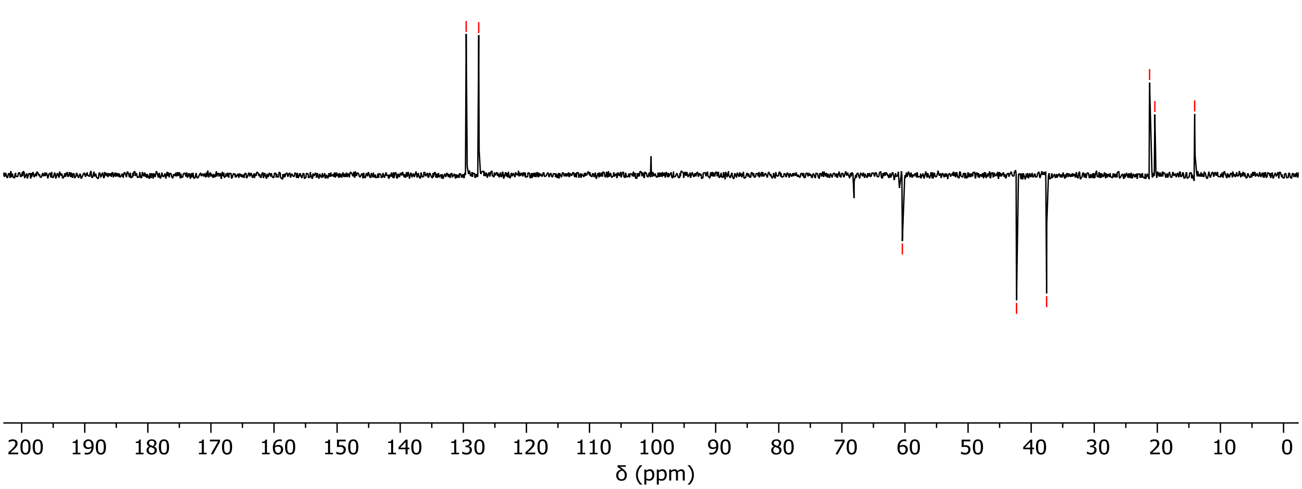

19F NMR (282 MHz, CDCl3)

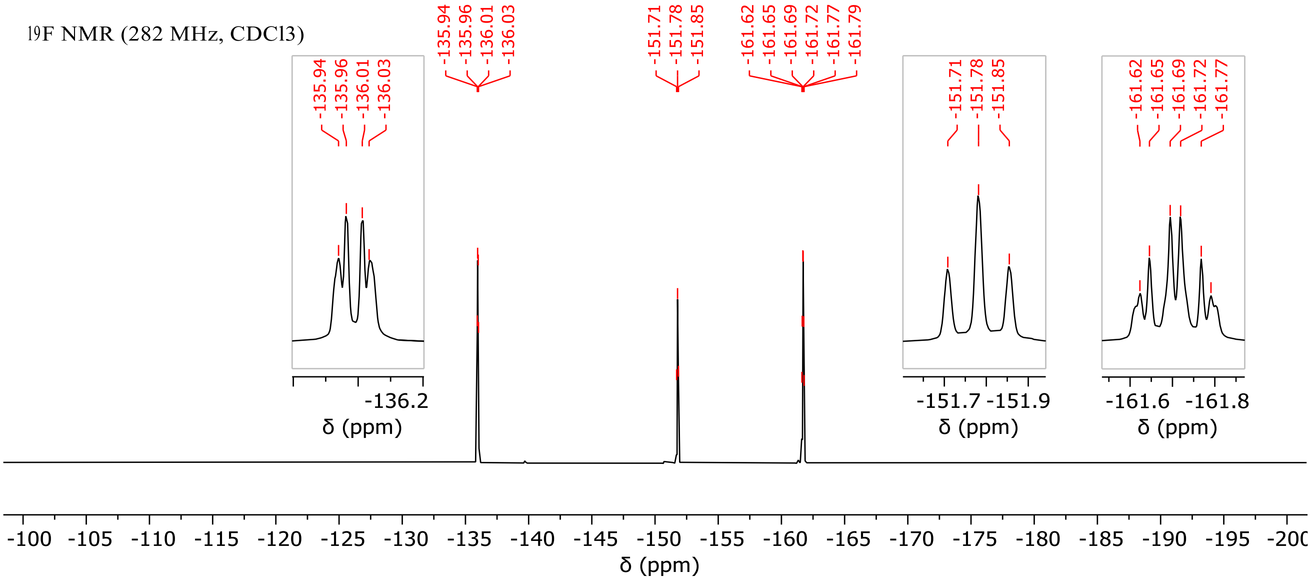

1H NMR (300 MHz, CDCl3)

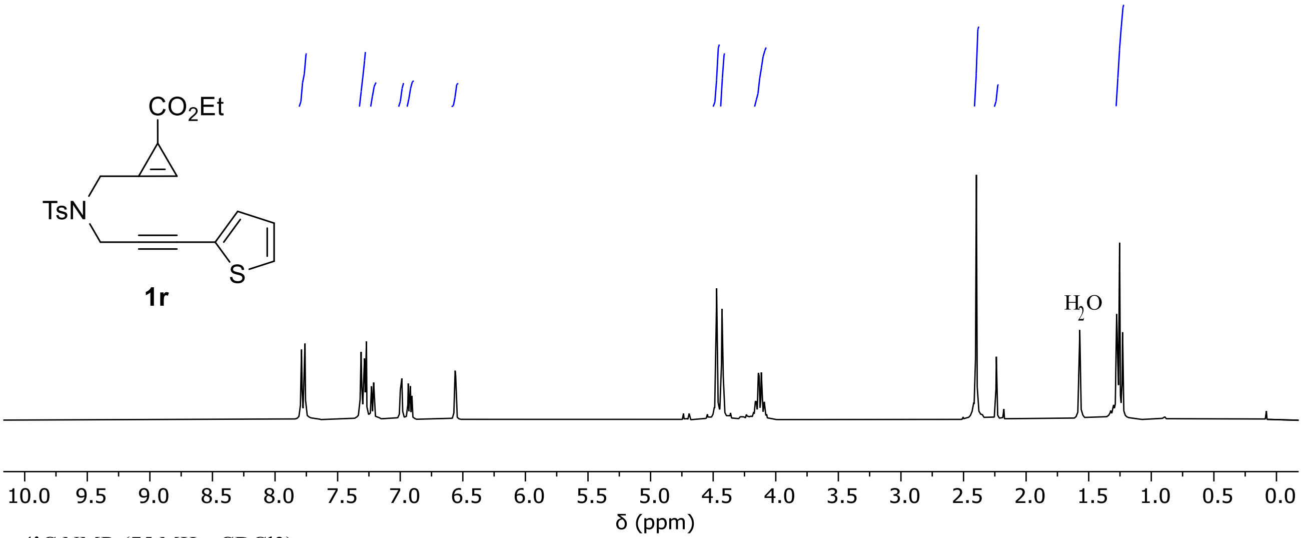

13C NMR (75 MHz, CDCl3)

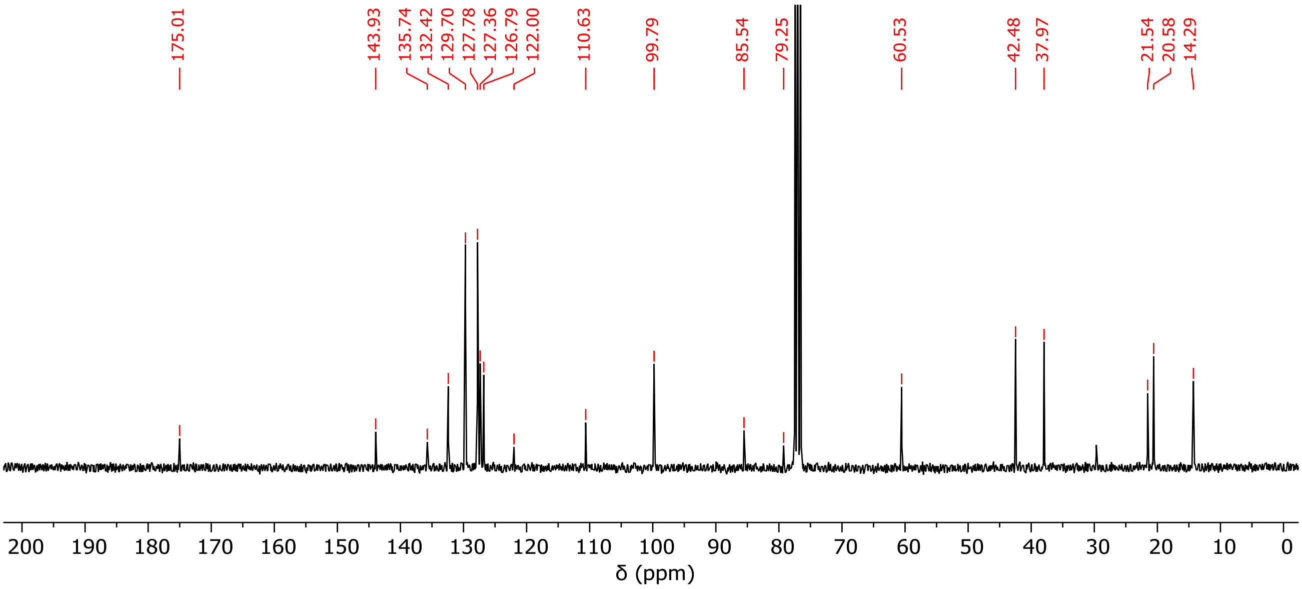

DEPT NMR (75 MHz, CDCl3)

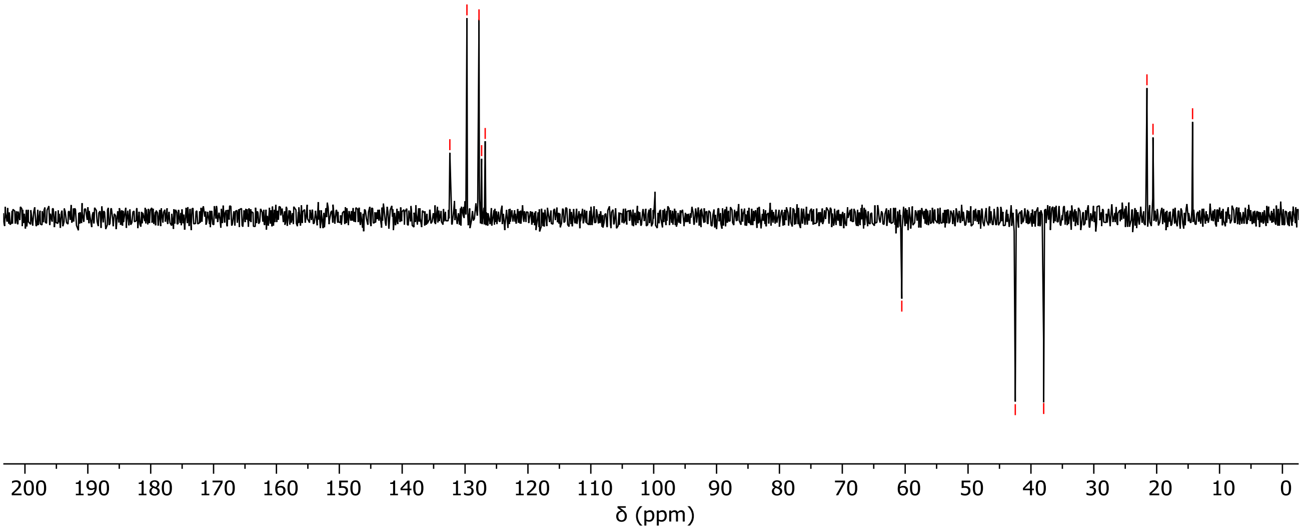

1H NMR (300 MHz, CDCl3)

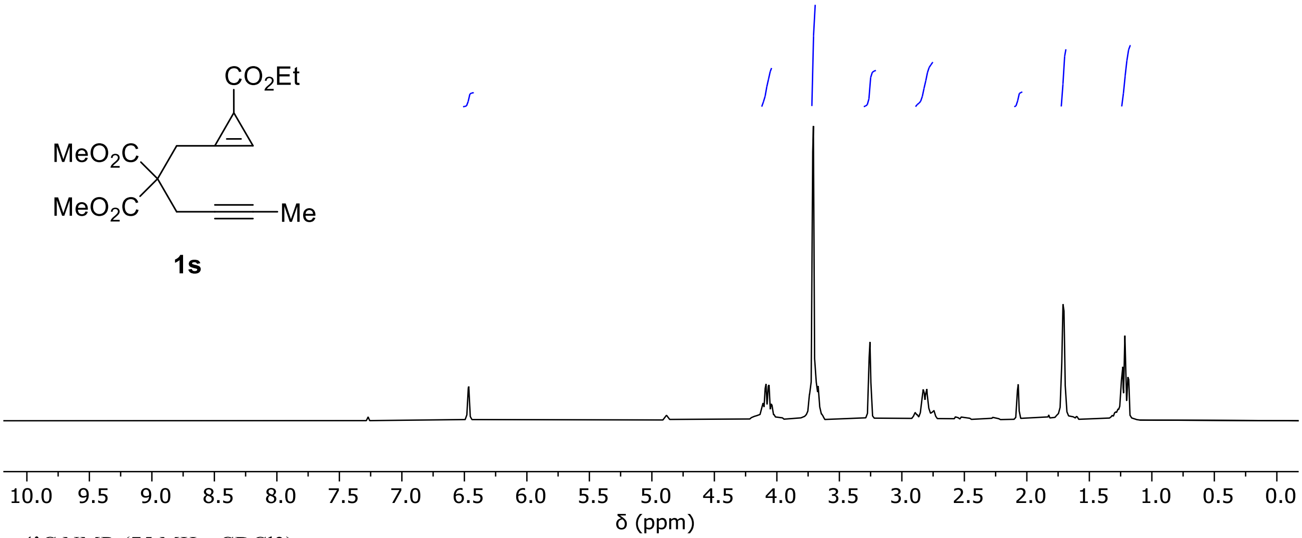

13C NMR (75 MHz, CDCl3)

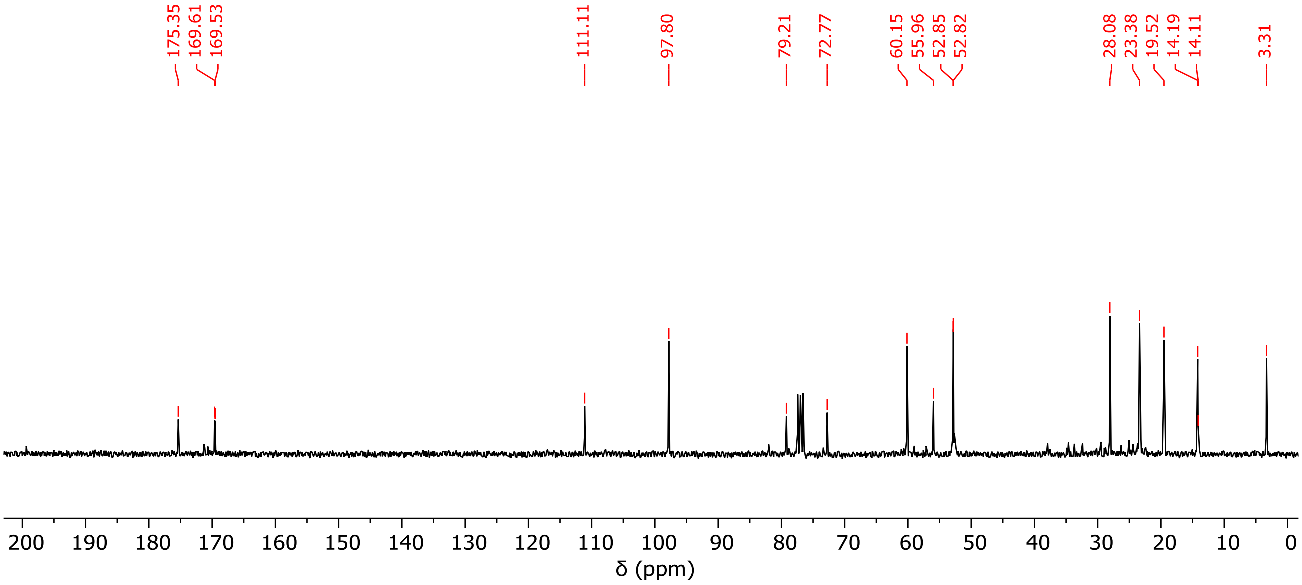

DEPT NMR (75 MHz, CDCl3)

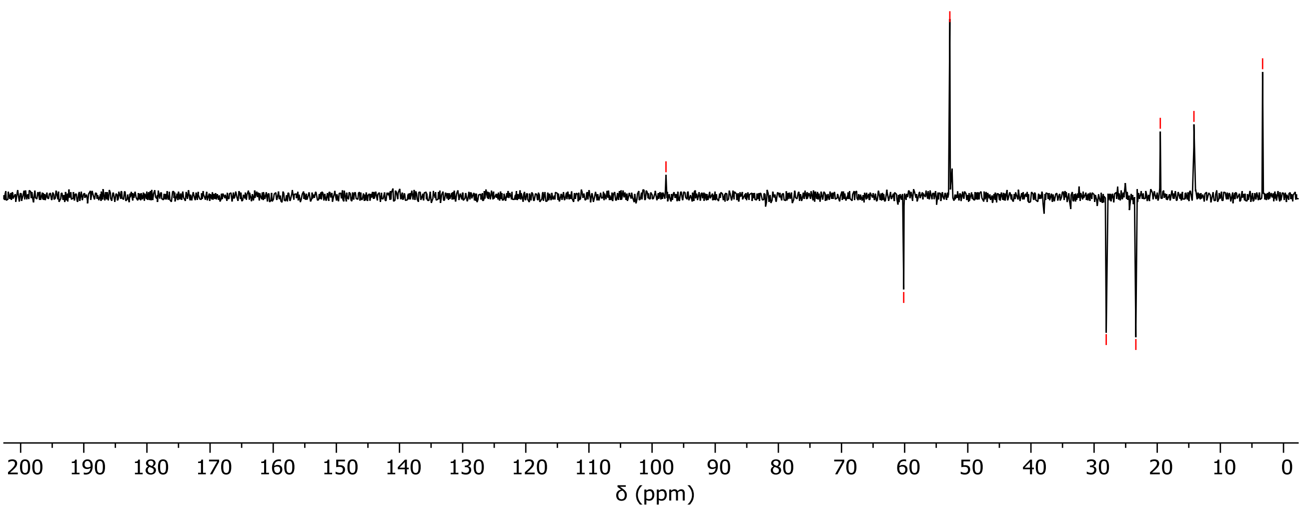

1H NMR (300 MHz, CDCl3)

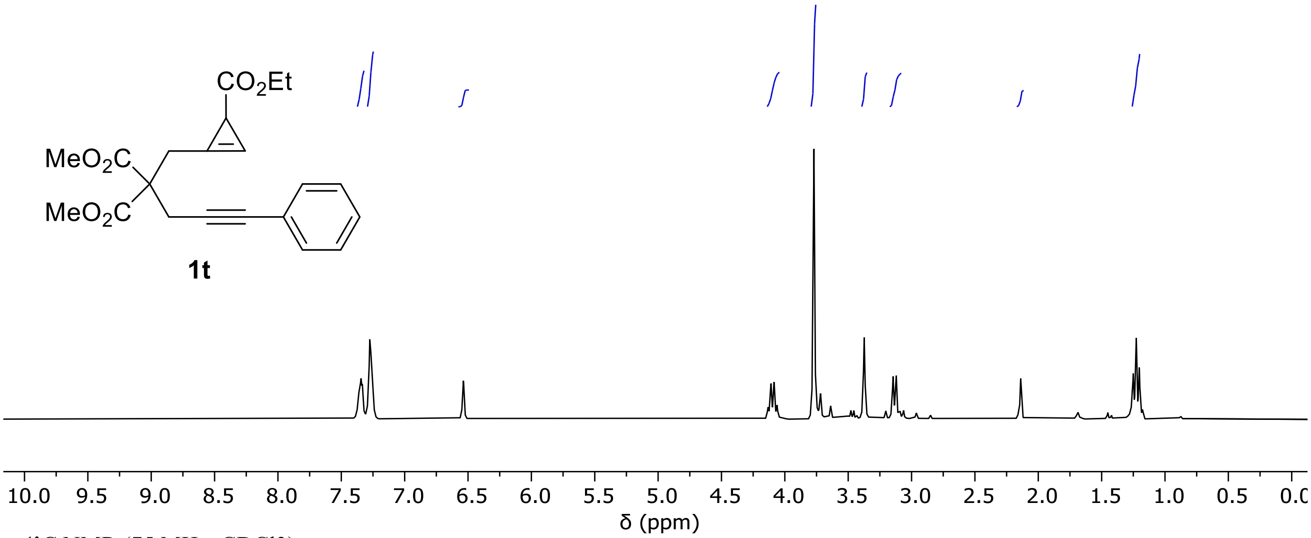

13C NMR (75 MHz, CDCl3)

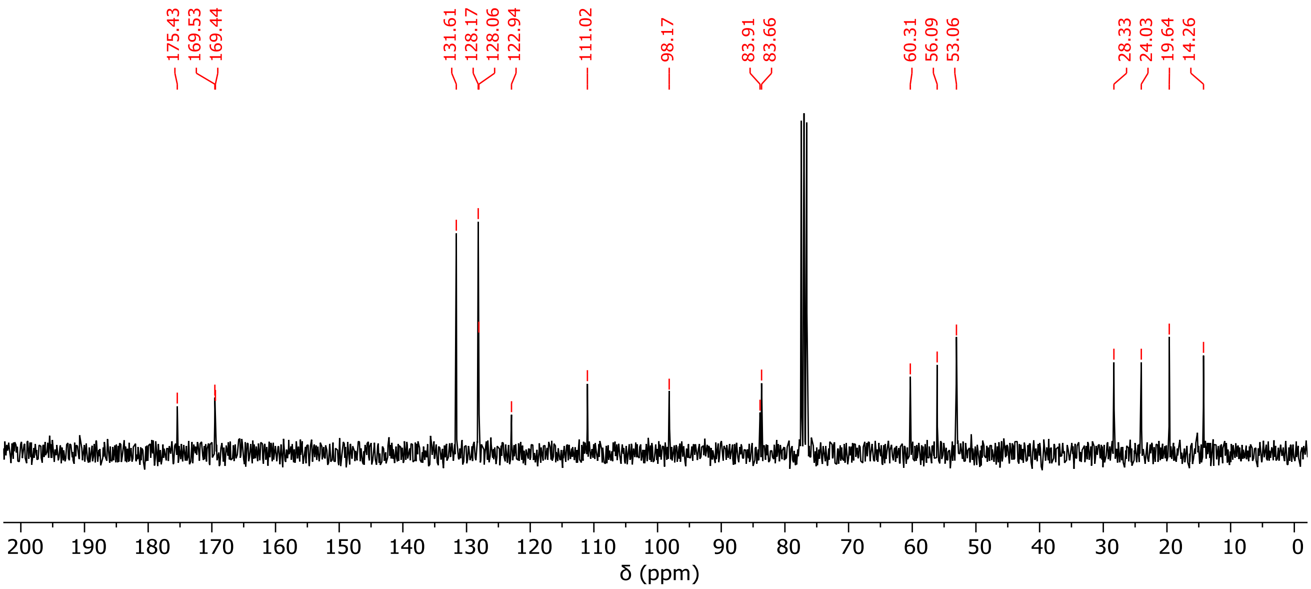

DEPT NMR (75 MHz, CDCl3)

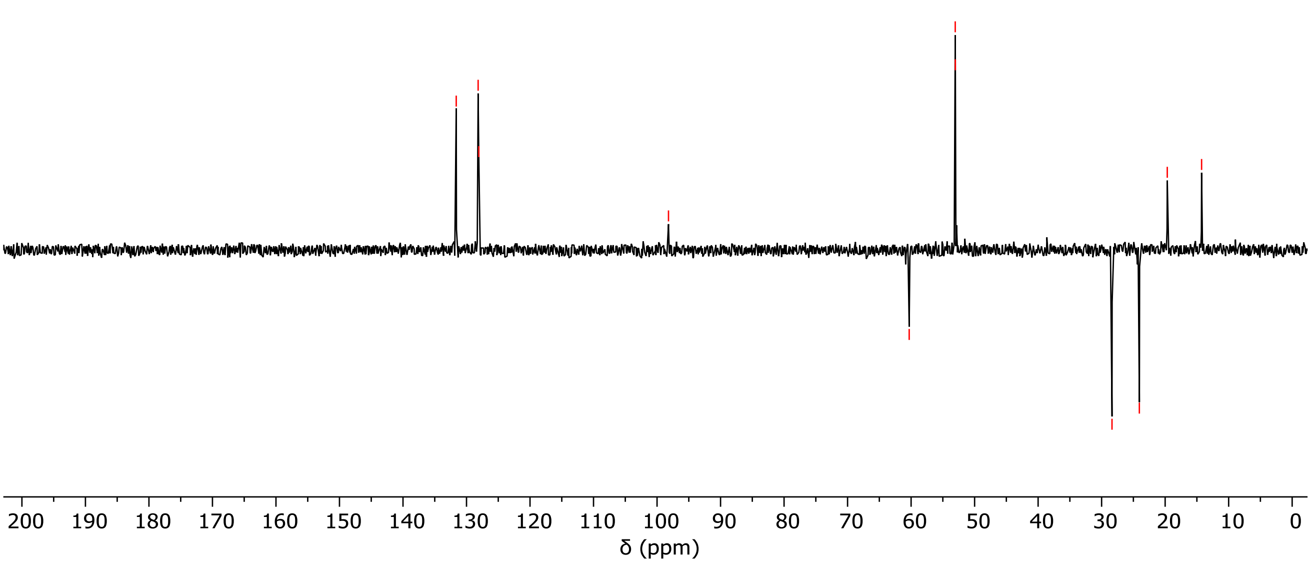

1H NMR (300 MHz, CDCl3)

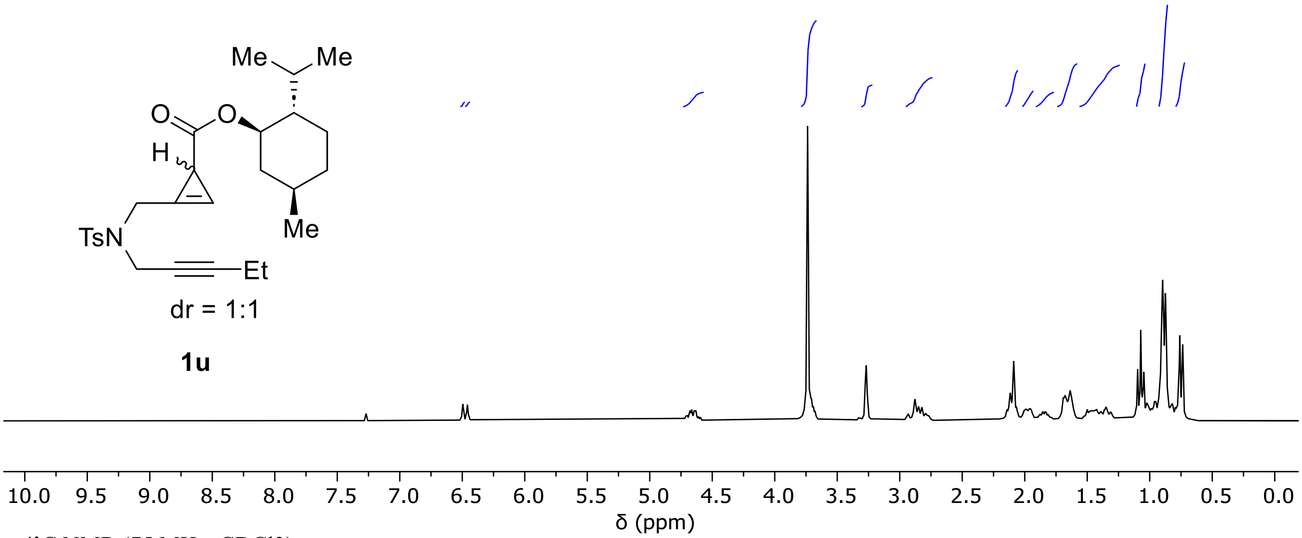

13C NMR (75 MHz, CDCl3)

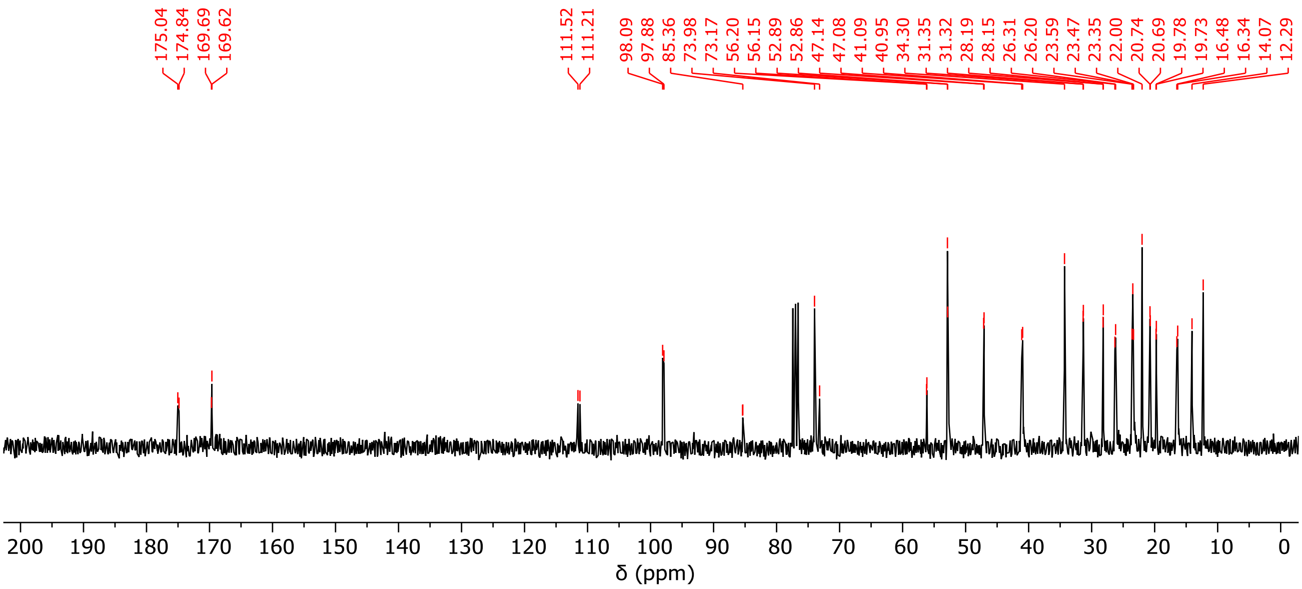

DEPT NMR (75 MHz, CDCl3)

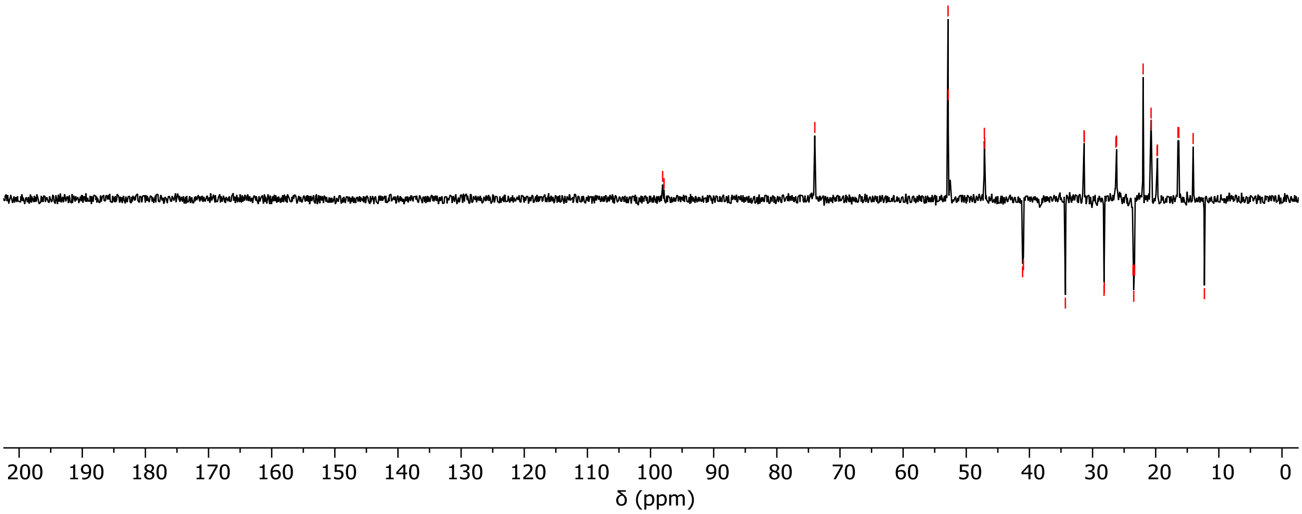

1H NMR (300 MHz, CDCl3)

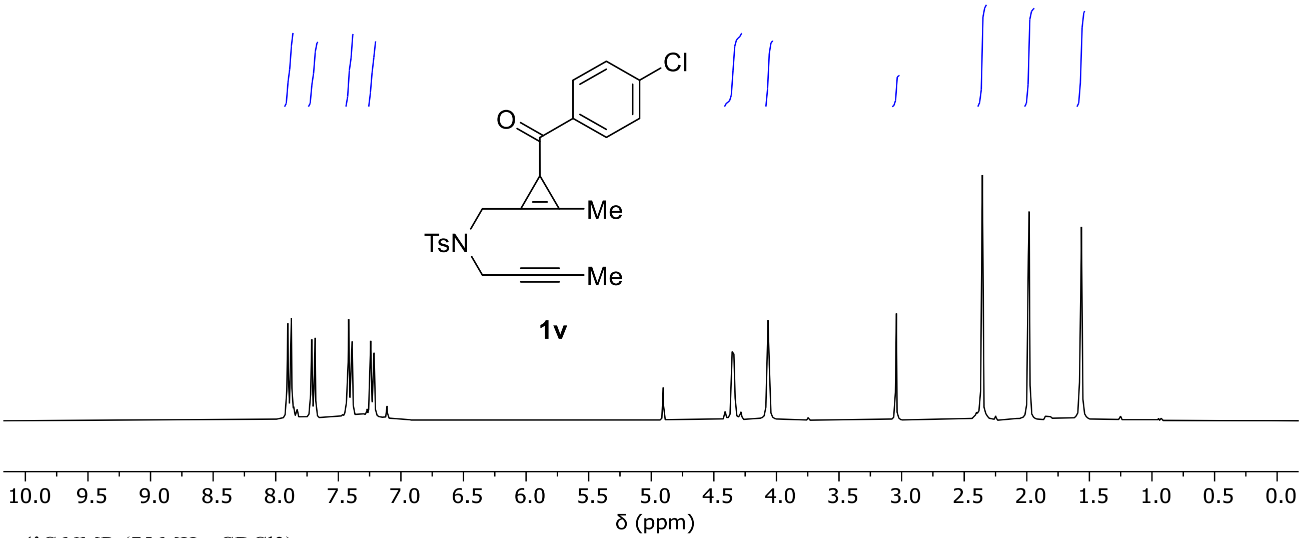

13C NMR (75 MHz, CDCl3)

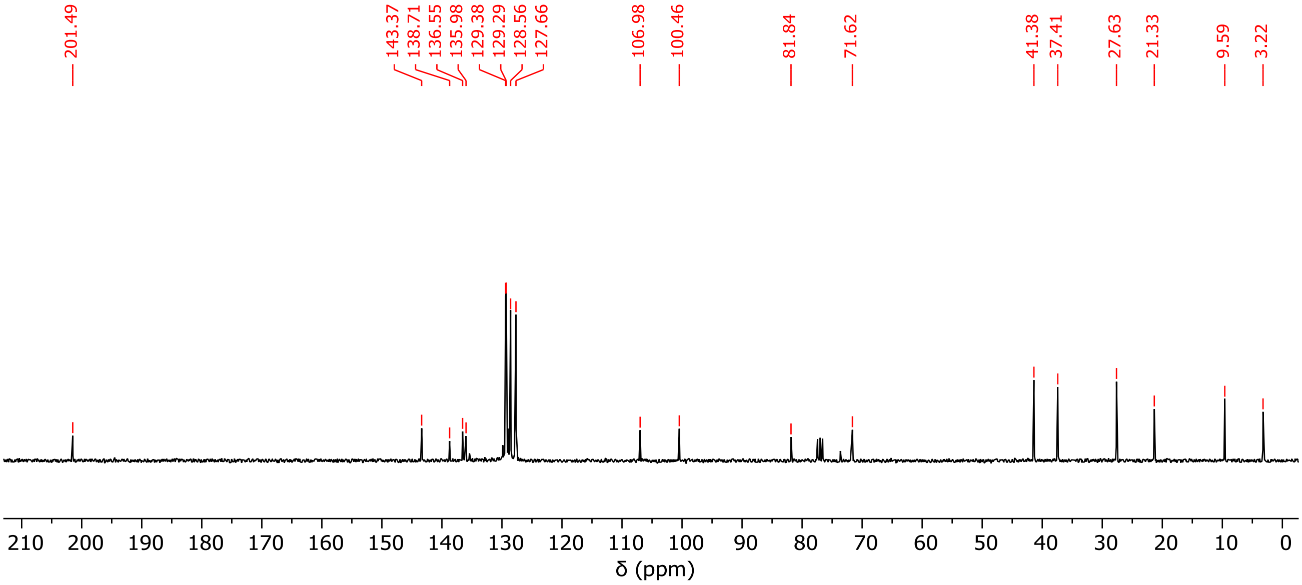

DEPT NMR (75 MHz, CDCl3)

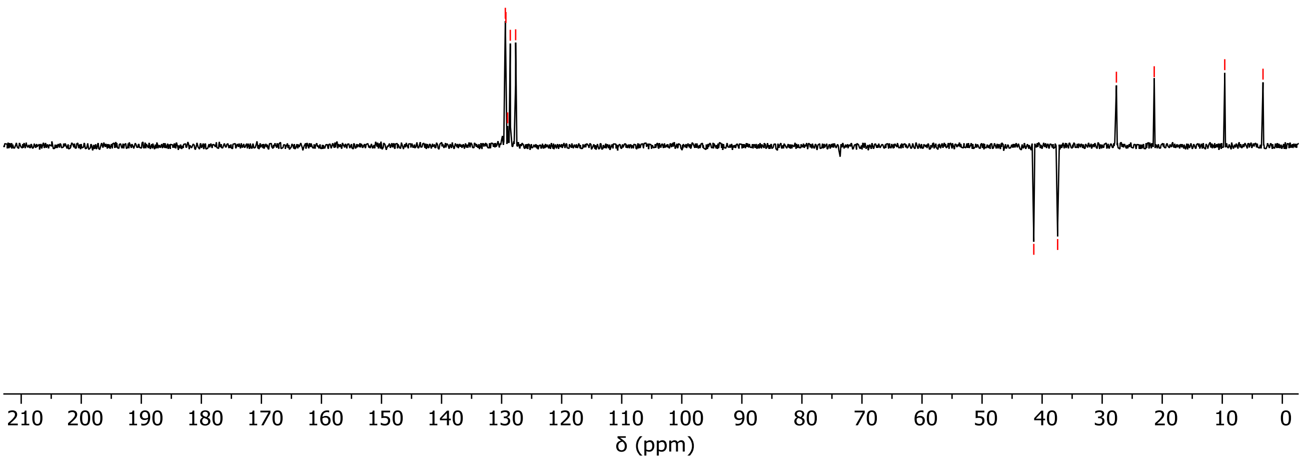

1H NMR (300 MHz, CDCl3)

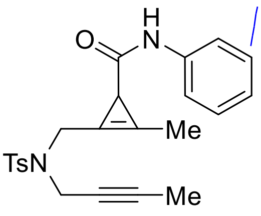

1w

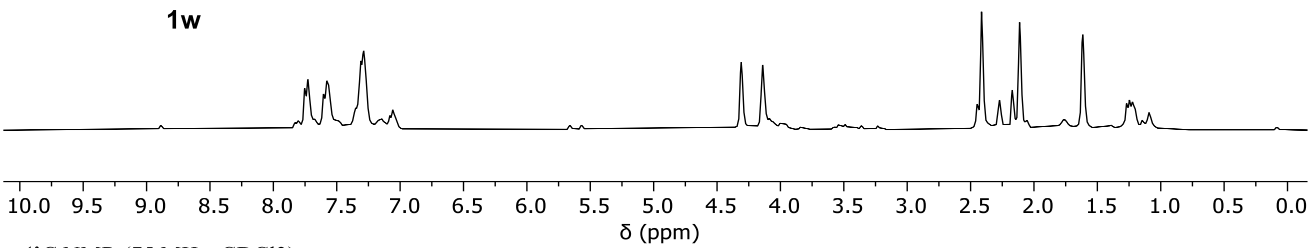

13C NMR (75 MHz, CDCl3)

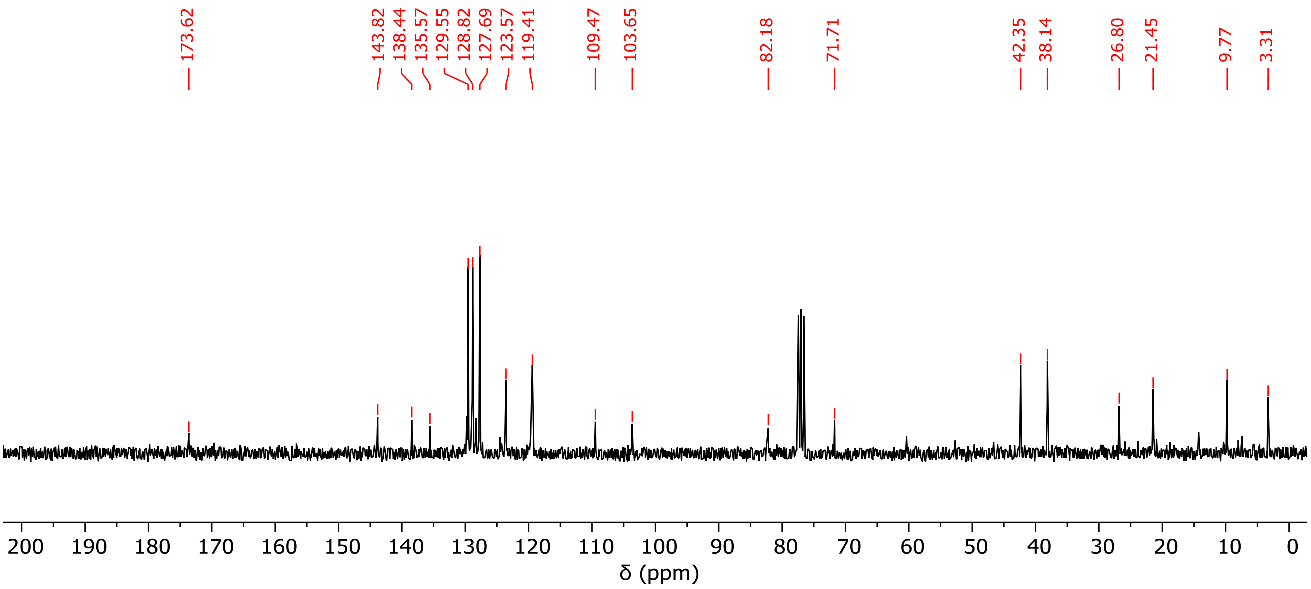

DEPT NMR (75 MHz, CDCl3)

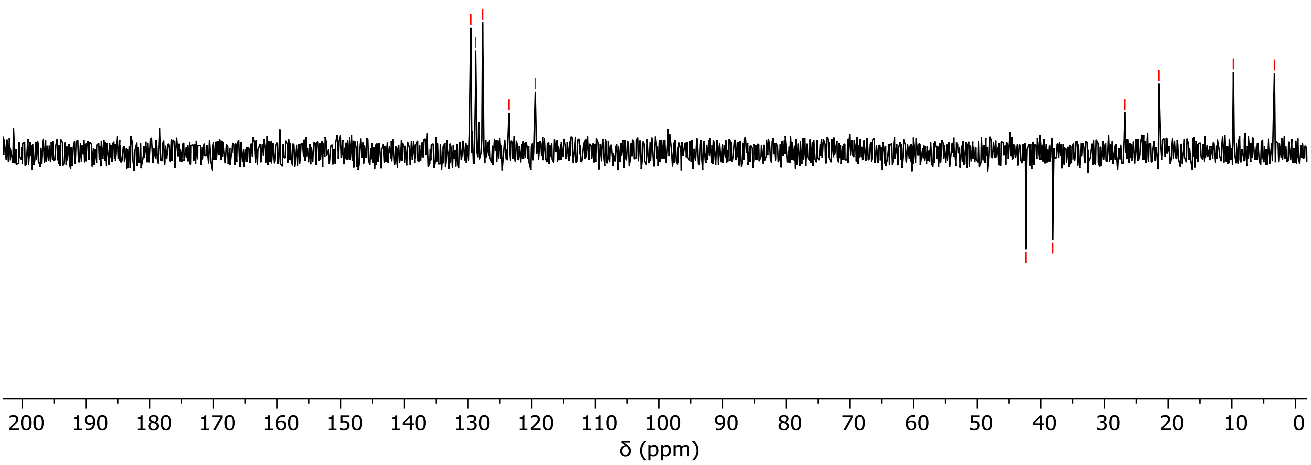

1H NMR (300 MHz, CDCl3)

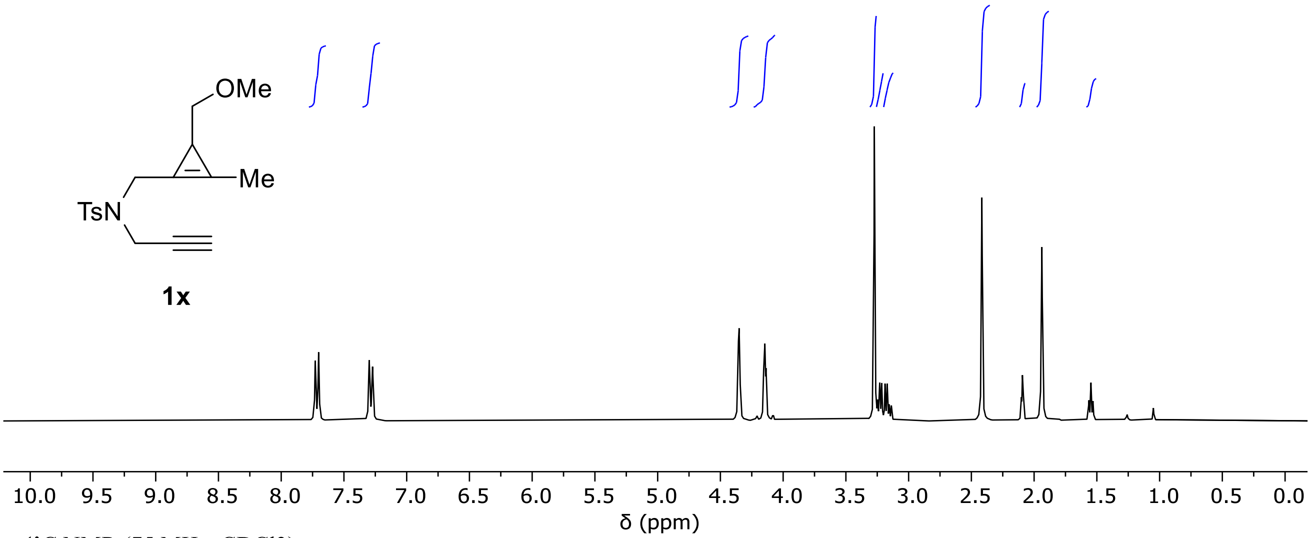

13C NMR (75 MHz, CDCl3)

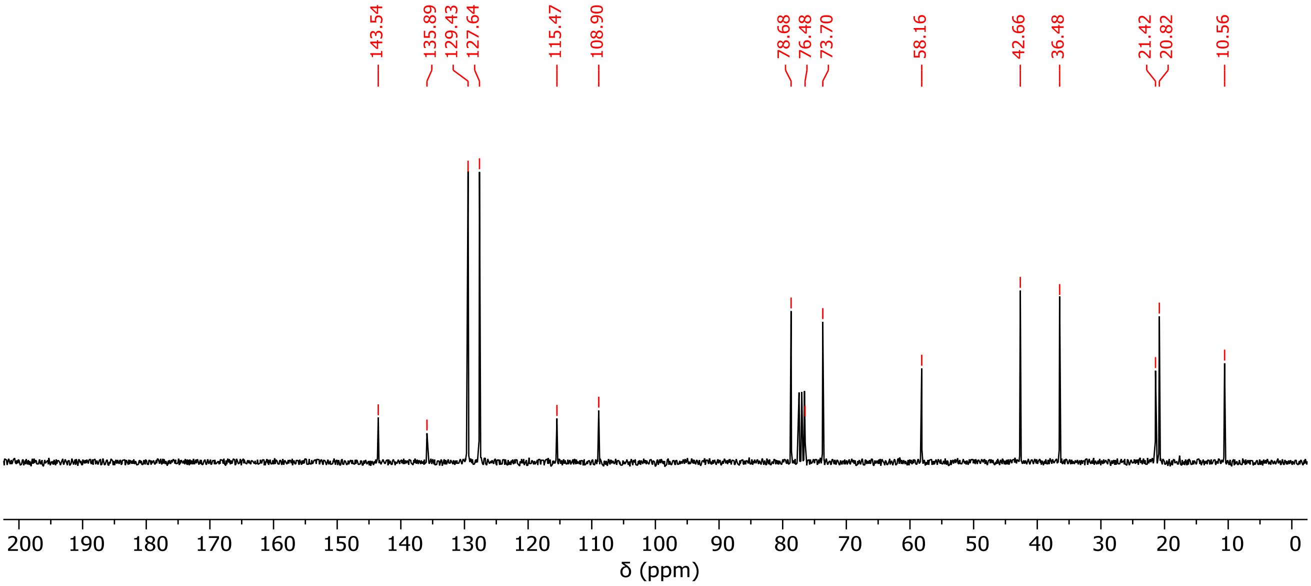

DEPT NMR (75 MHz, CDCl3)

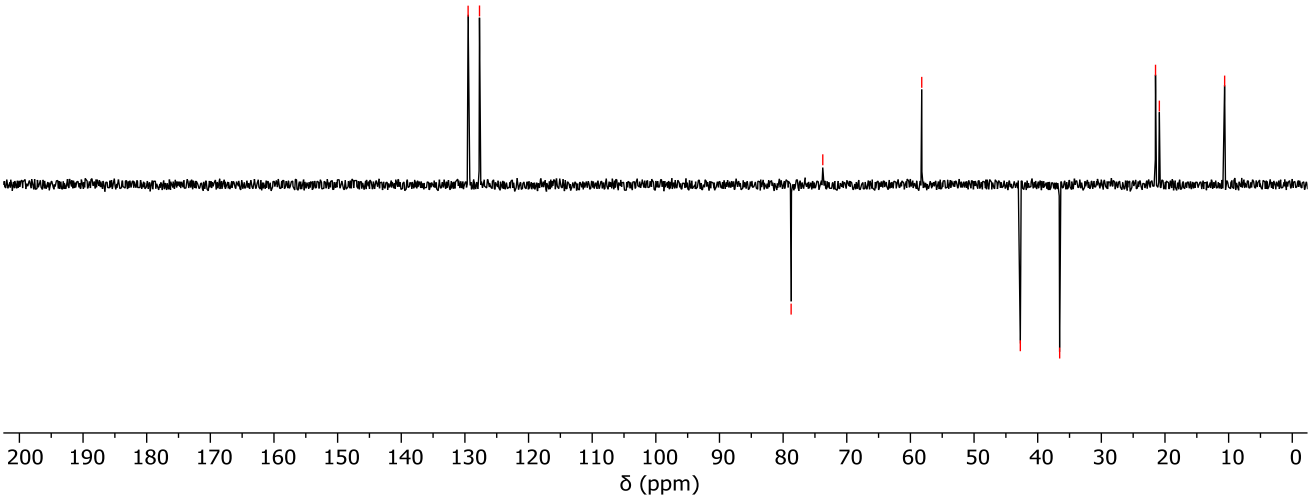

1H NMR (300 MHz, CDCl3)

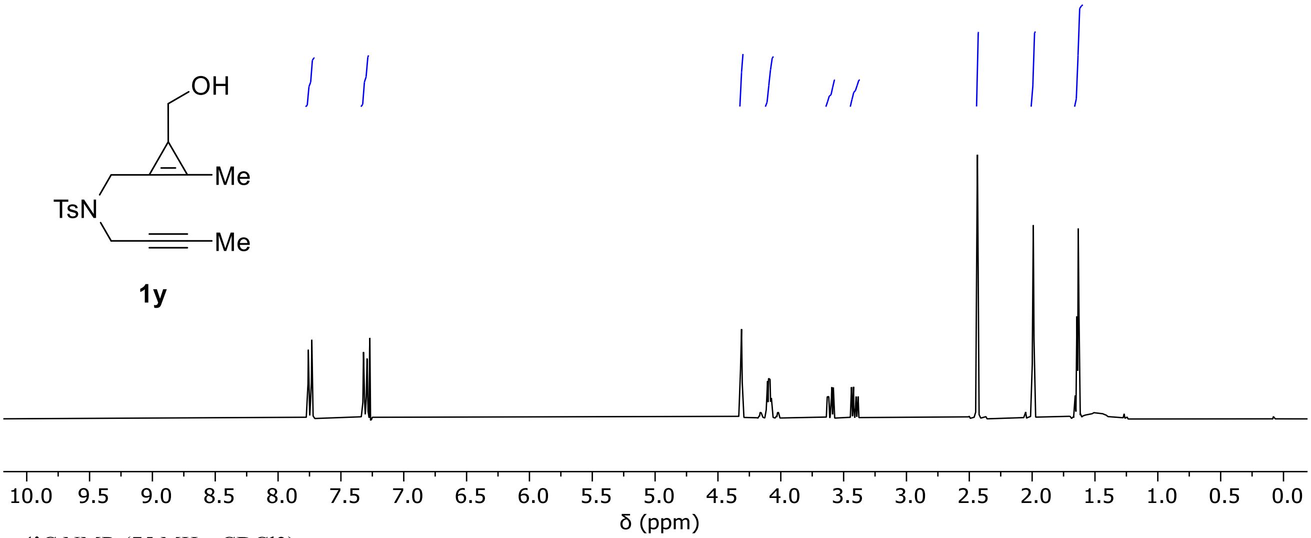

13C NMR (75 MHz, CDCl3)

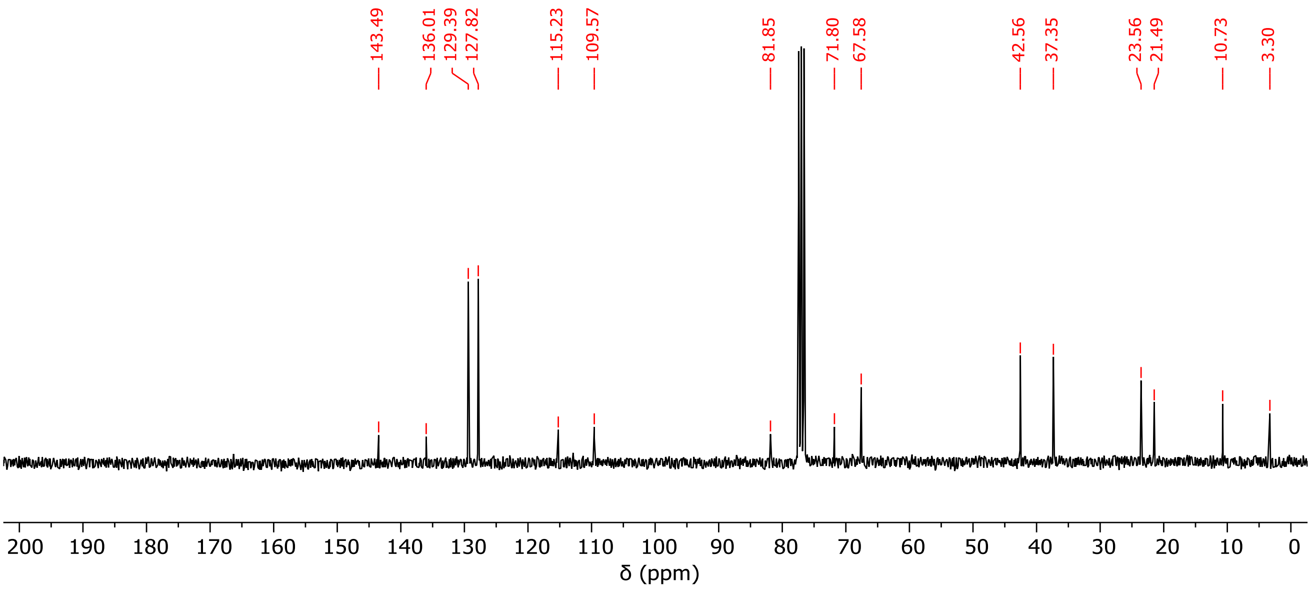

DEPT NMR (75 MHz, CDCl3)

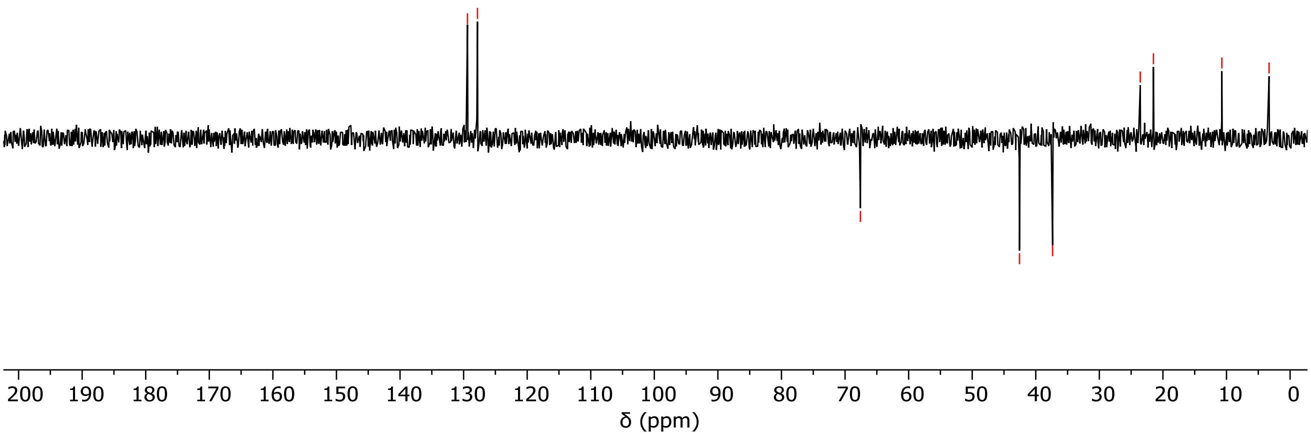

1H NMR (500 MHz, CDCl3)

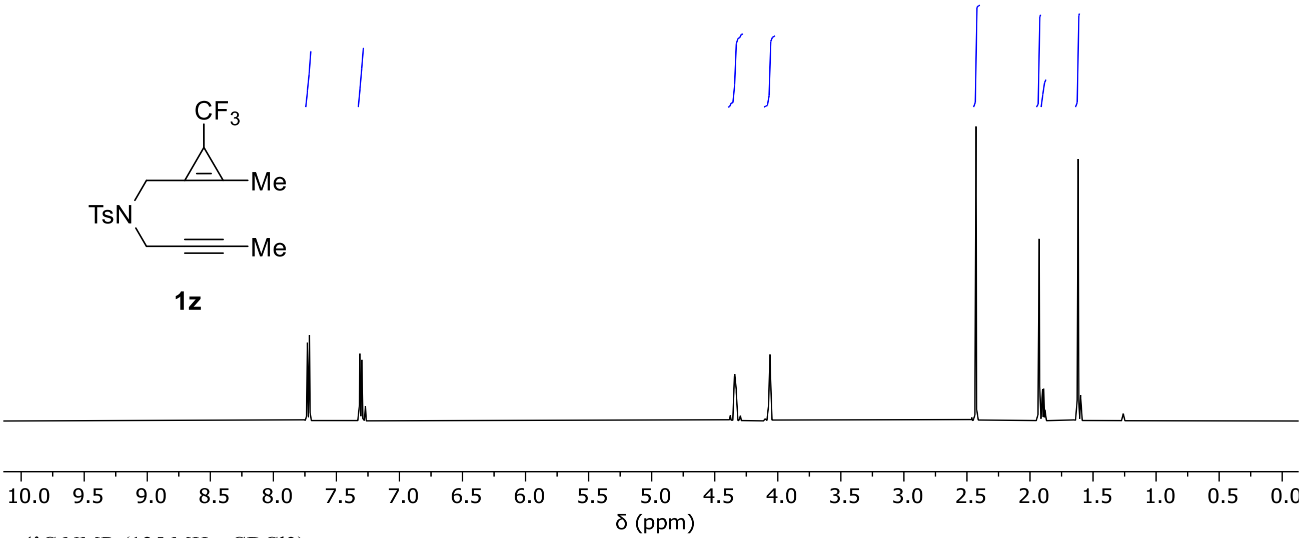

13C NMR (125 MHz, CDCl3)

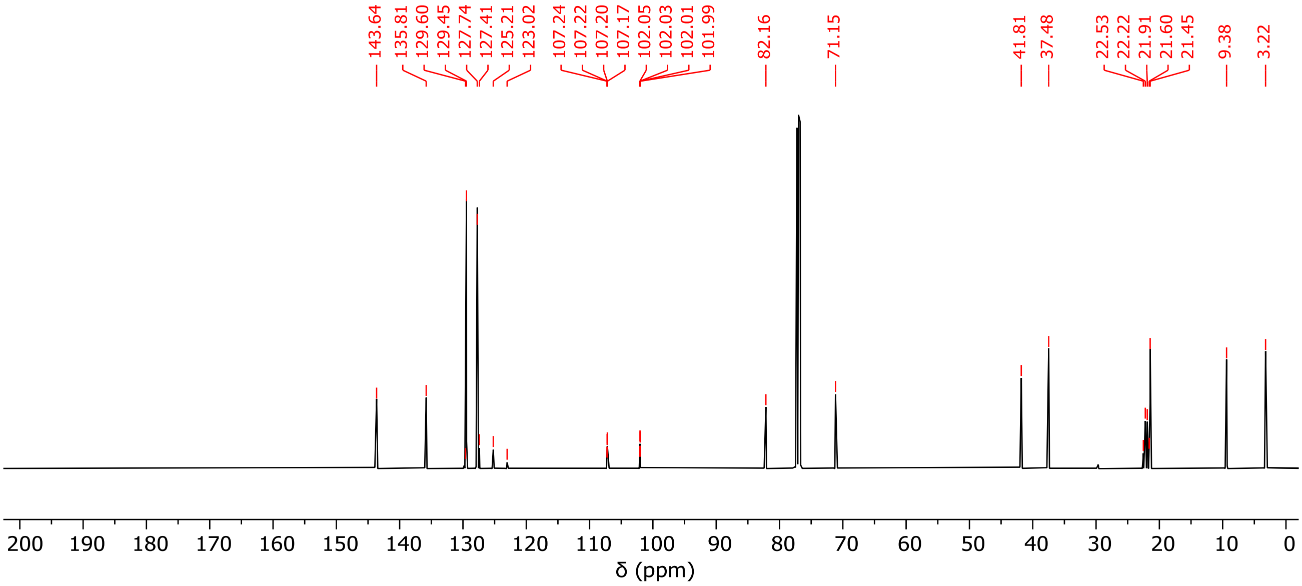

DEPT NMR (125 MHz, CDCl3)

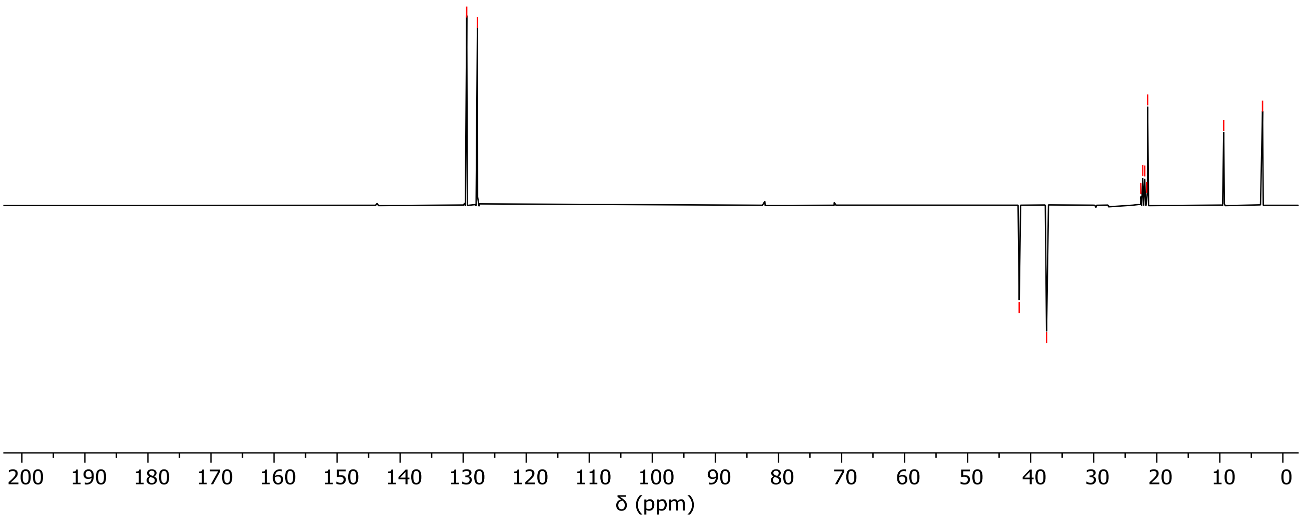

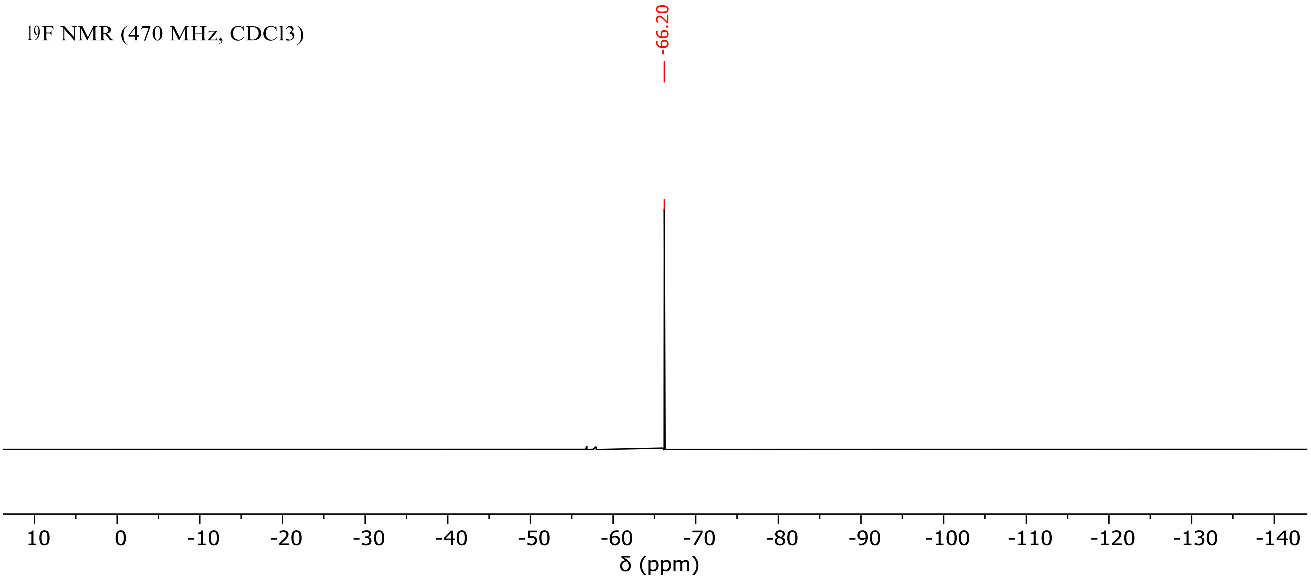
19F NMR (470 MHz, CDCl3)
-66.20
10
0
-10
-20
-30
-40
-50
-60
-70
-80
-90
-100
-110
-120
-130
-140
δ (ppm)

1H NMR (500 MHz, CDCl3)

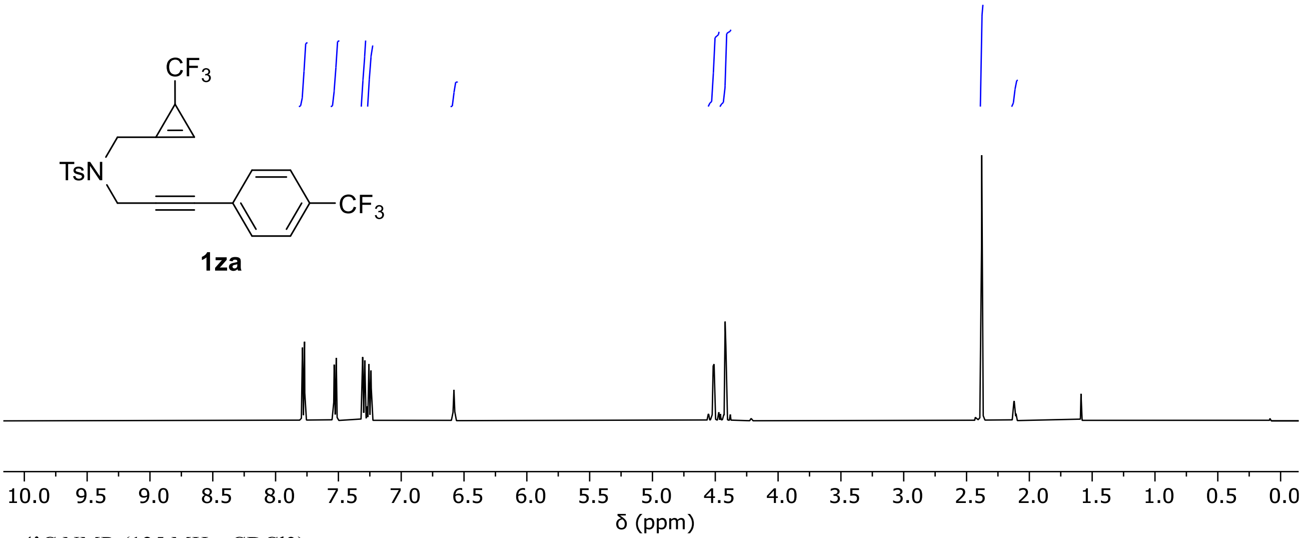

13C NMR (125 MHz, CDCl3)

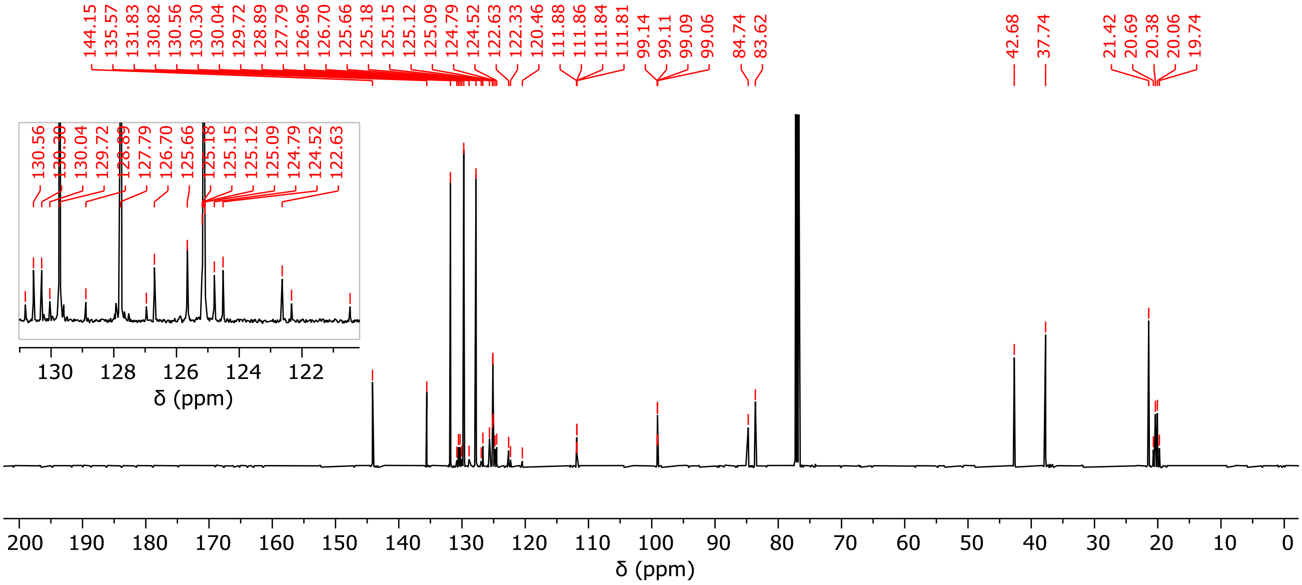

DEPT NMR (125 MHz, CDCl3)

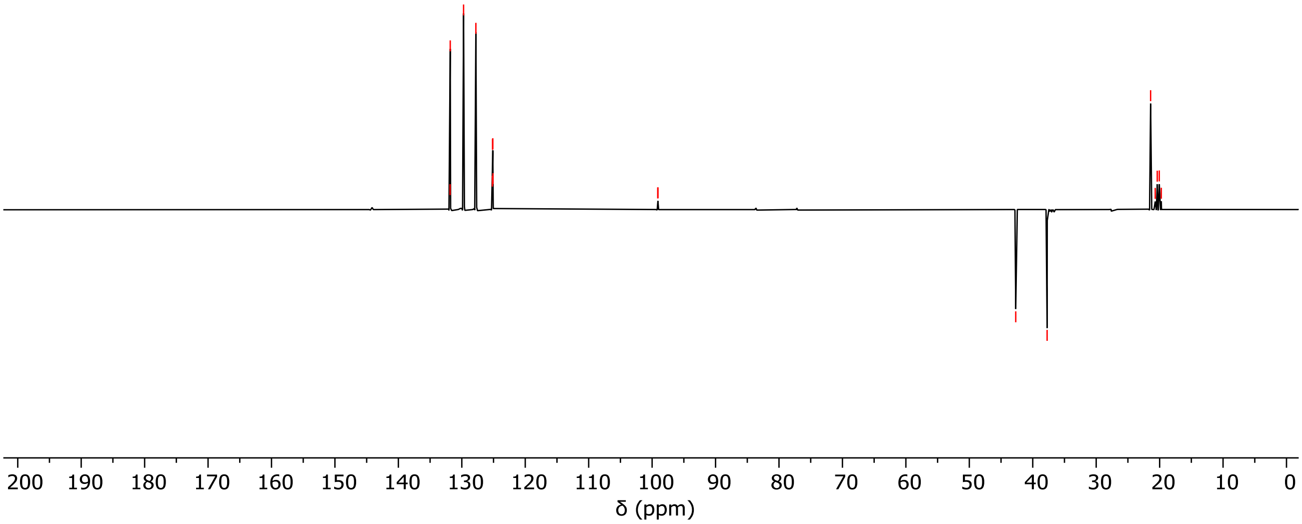

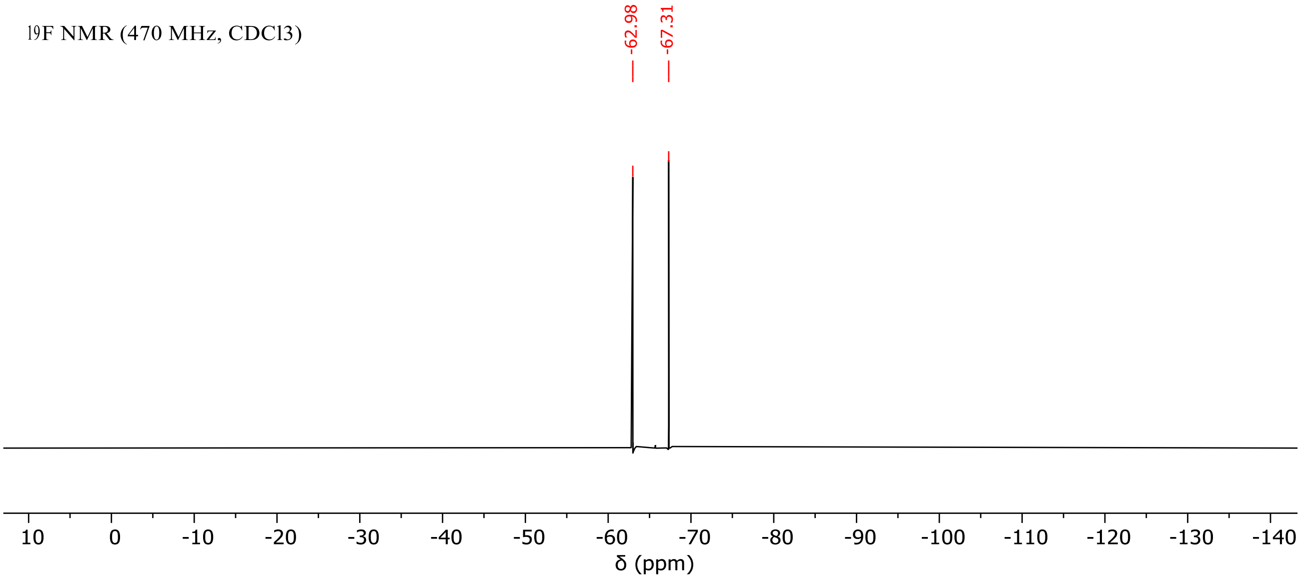

19F NMR (470 MHz, CDCl3)
-62.98
-67.31
10
0
-10
-20
-30
-40
-50
-60
-70
-80
-90
-100
-110
-120
-130
-140
δ (ppm)

1H NMR (300 MHz, CDCl3)

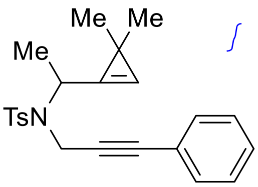

1zc

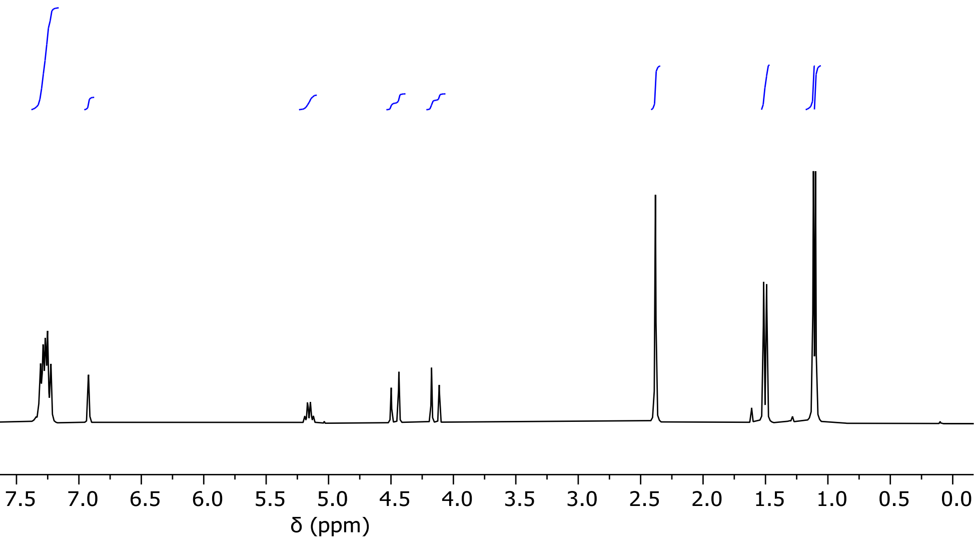

13C NMR (75 MHz, CDCl3)

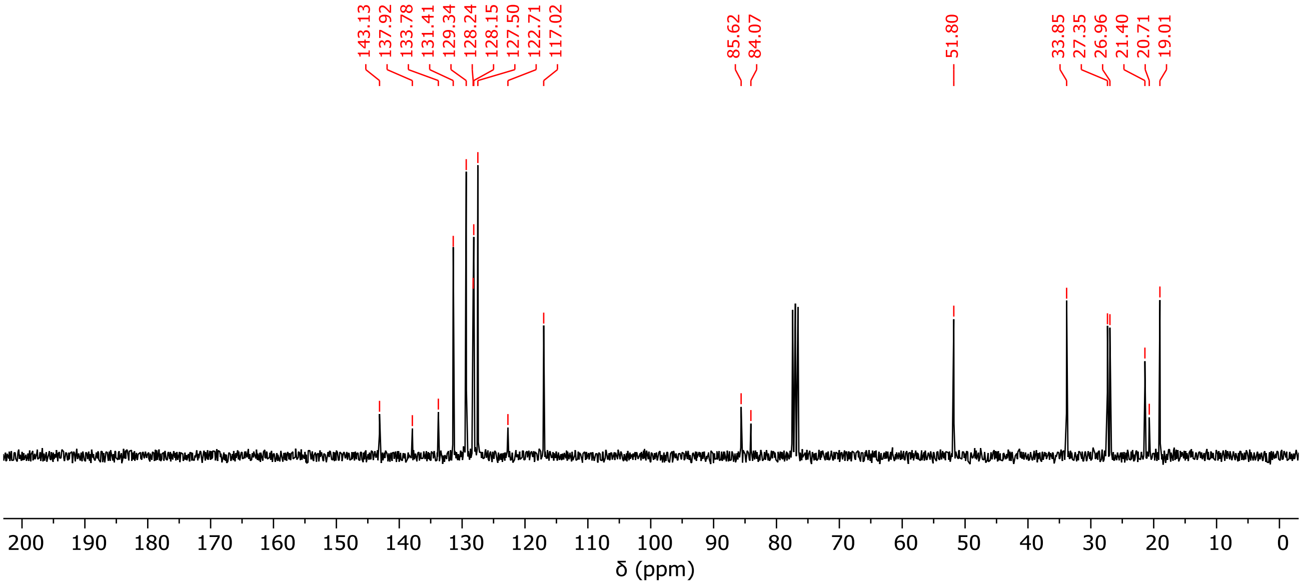

DEPT NMR (75 MHz, CDCl3)

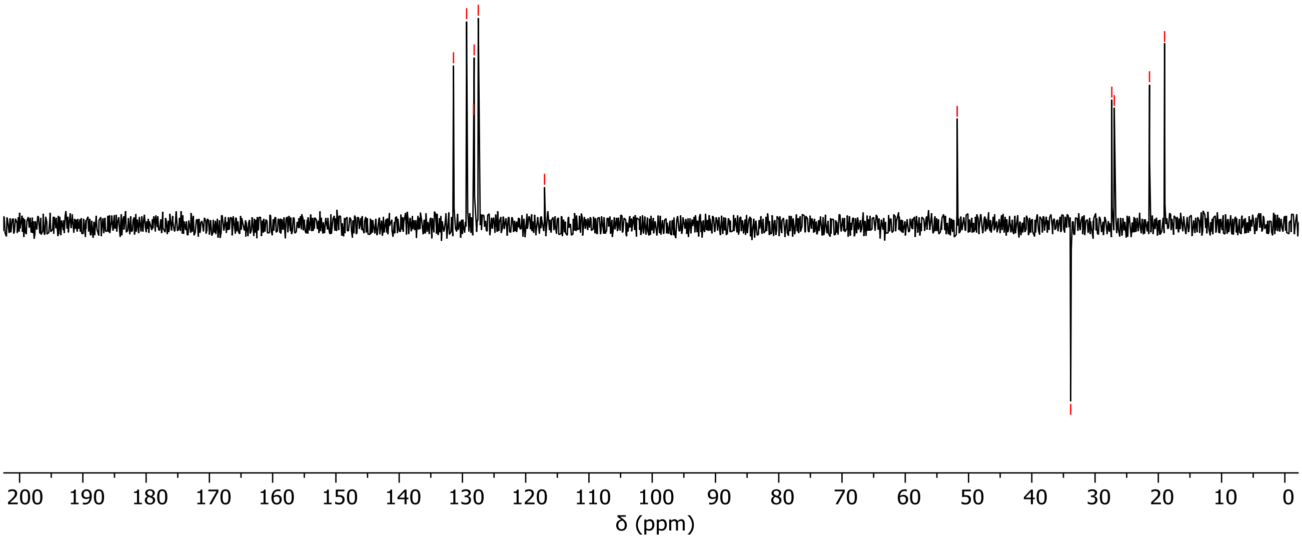

1H NMR (300 MHz, CDCl3)

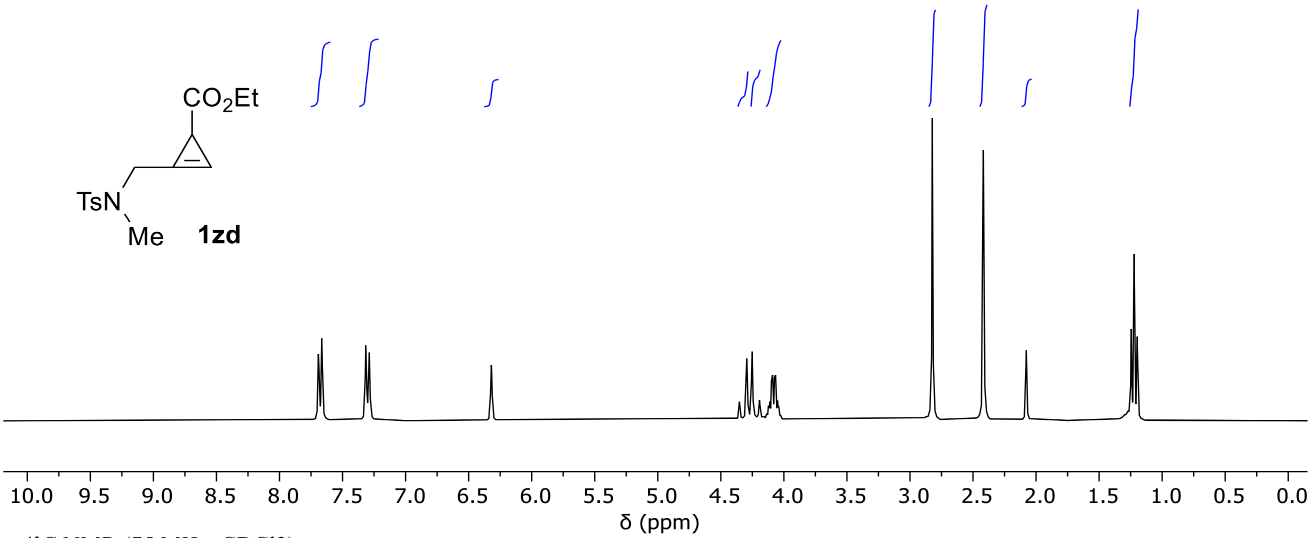

13C NMR (75 MHz, CDCl3)

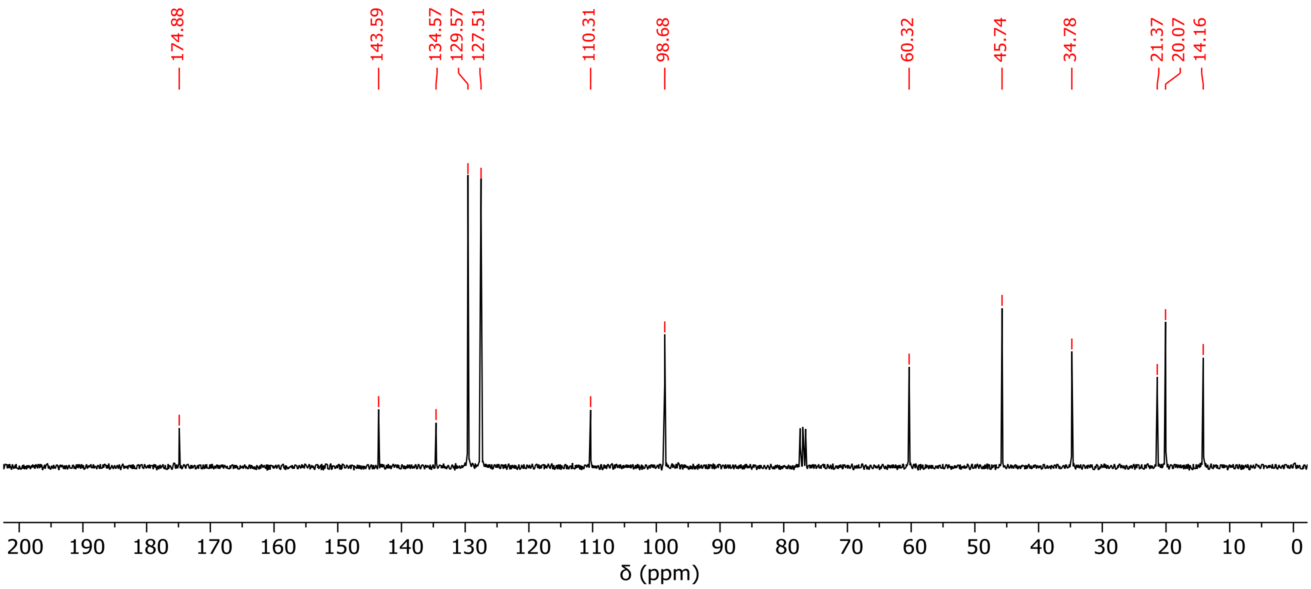

DEPT NMR (75 MHz, CDCl3)

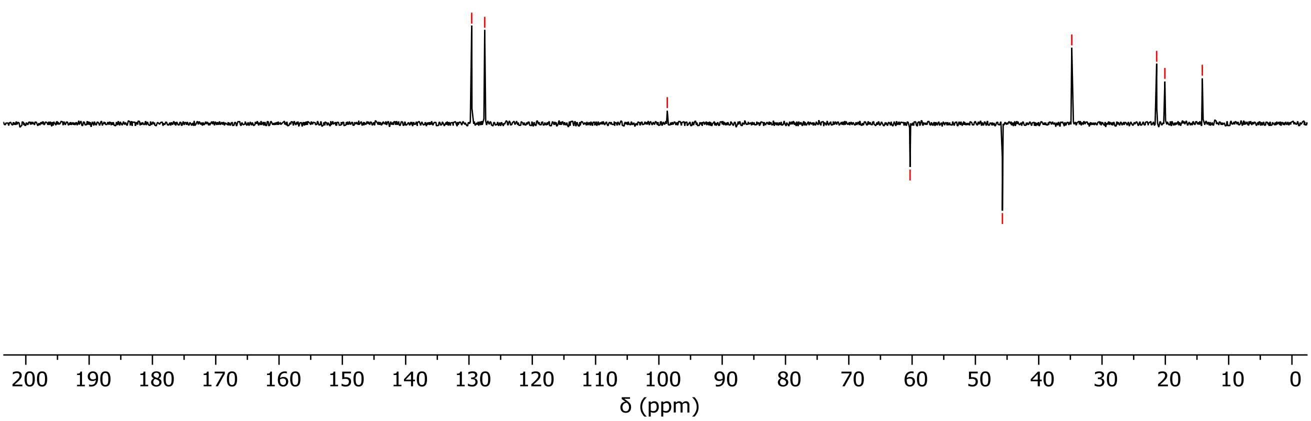

1H NMR (300 MHz, CDCl3)

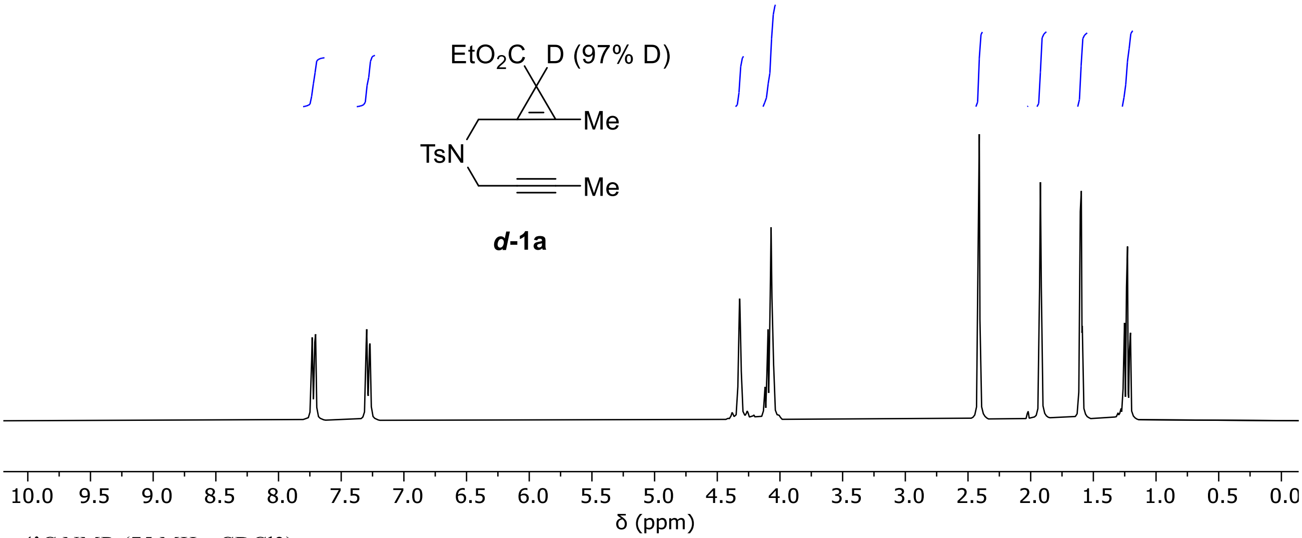

13C NMR (75 MHz, CDCl3)

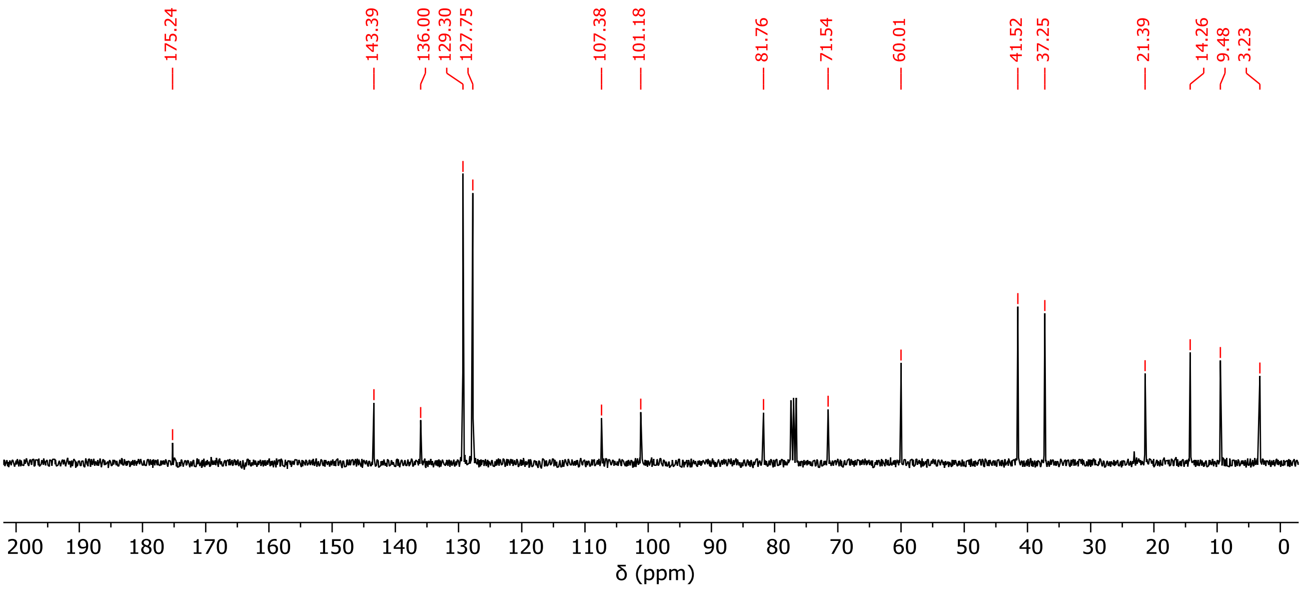

DEPT NMR (75 MHz, CDCl3)

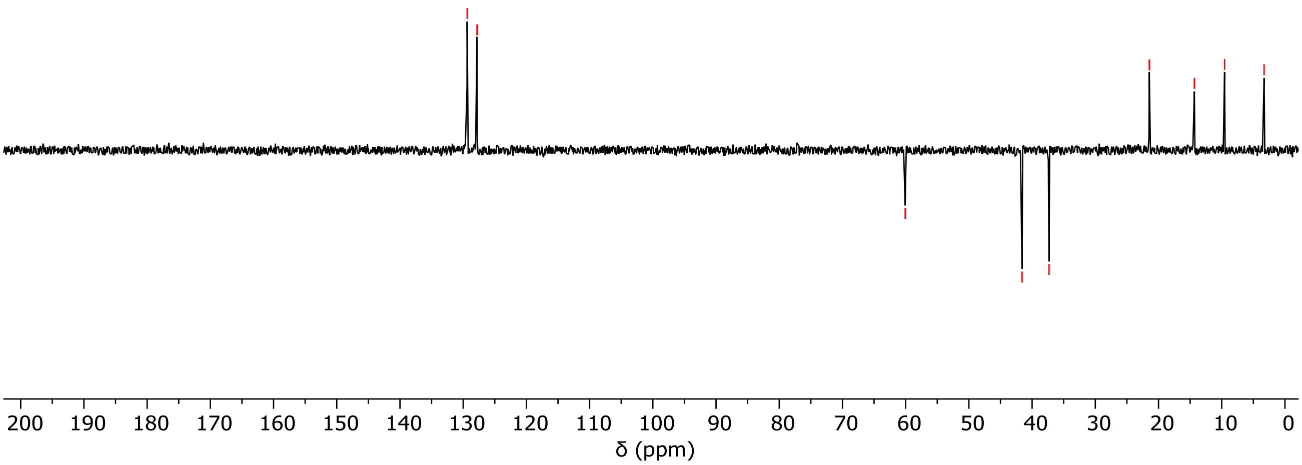

1H NMR (300 MHz, CDCl3)

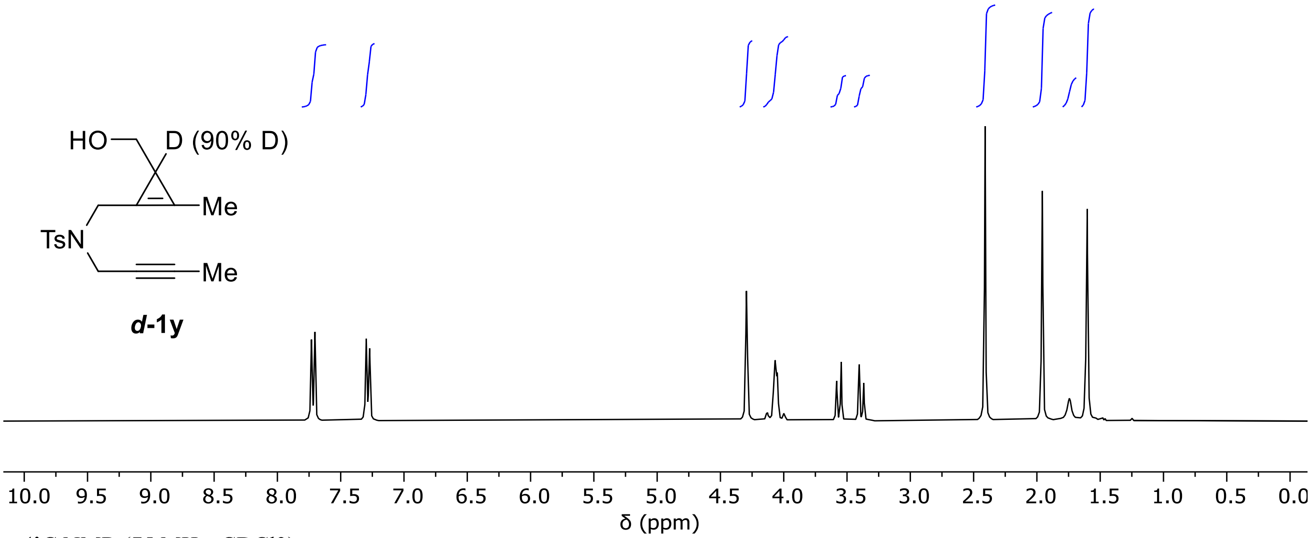

13C NMR (75 MHz, CDCl3)

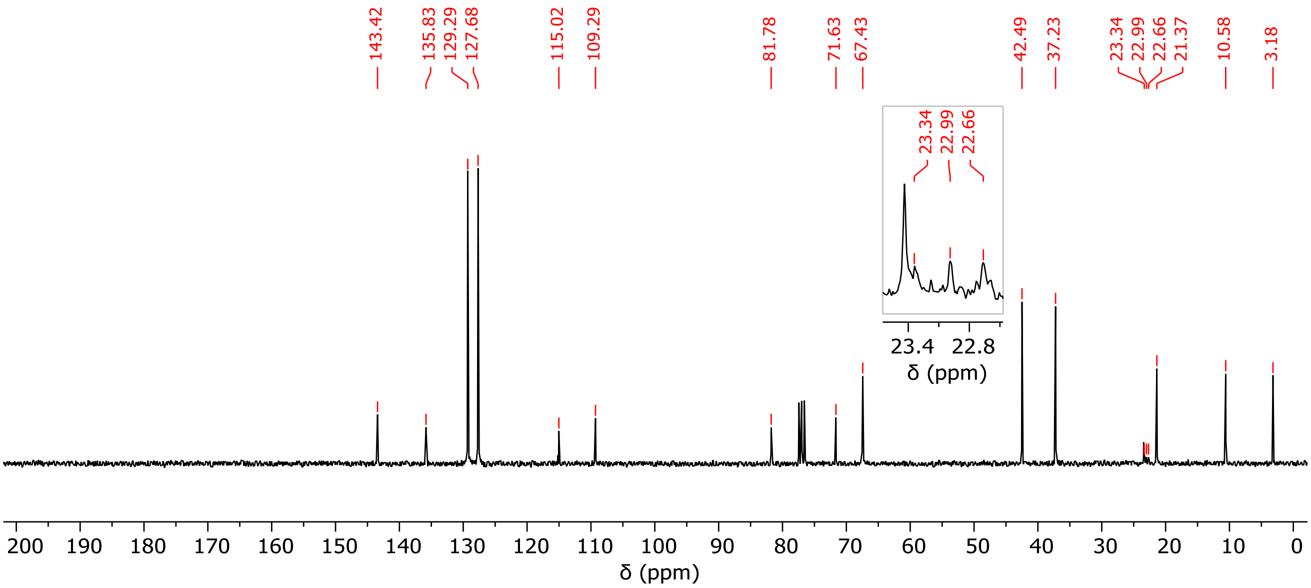

DEPT NMR (75 MHz, CDCl3)

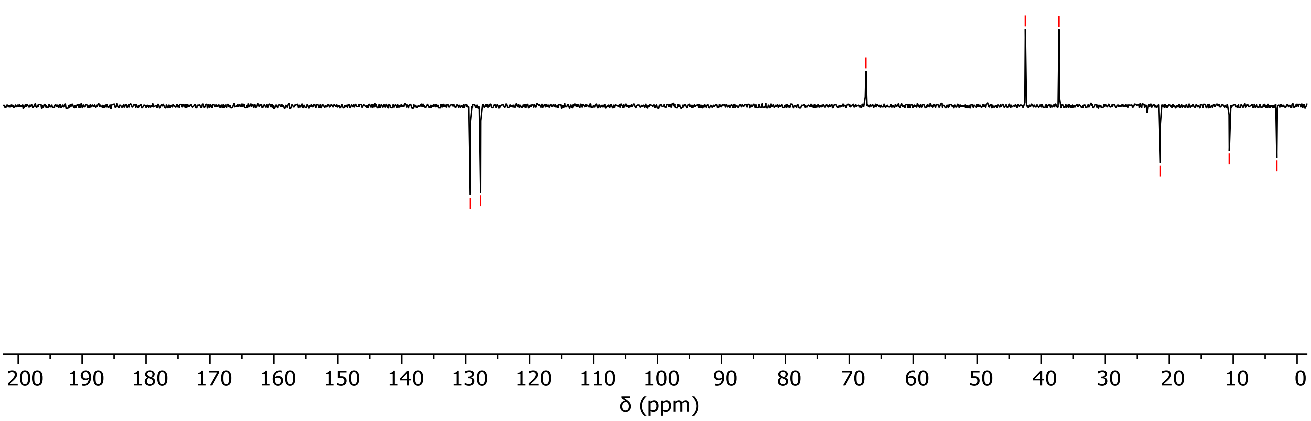

1H NMR (300 MHz, CDCl3)

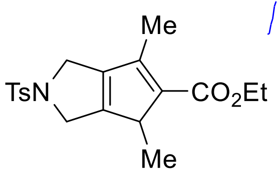

2a

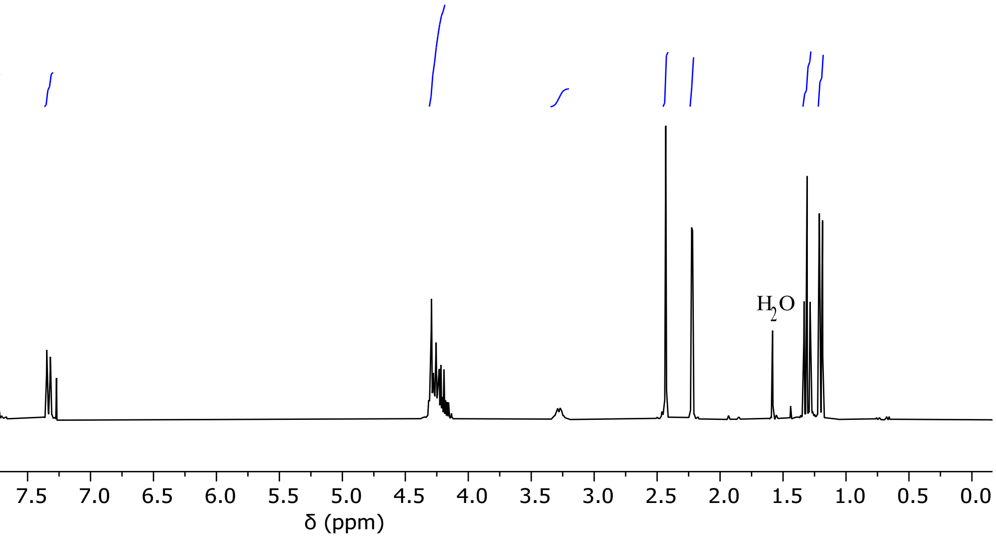

13C NMR (75 MHz,CDCl3)

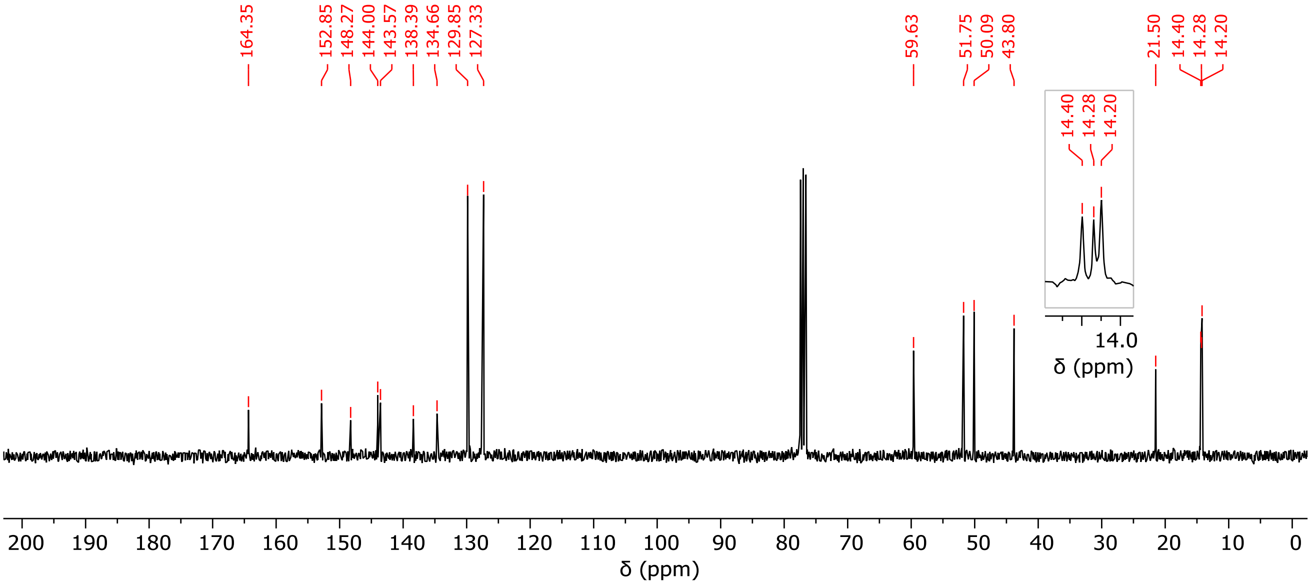

DEPT NMR (75 MHz,CDCl3)

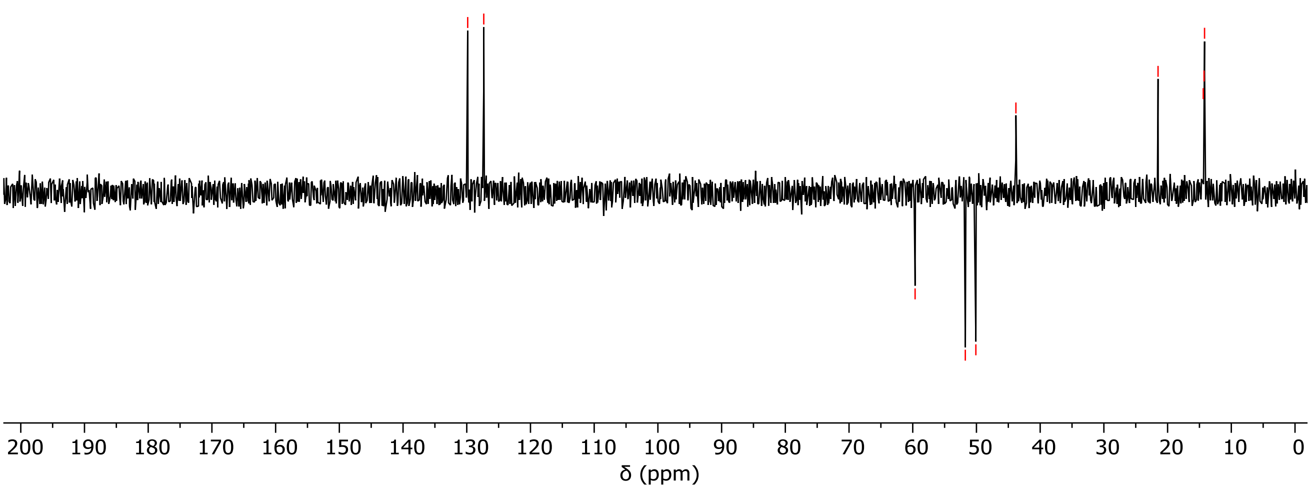

1H NMR (300 MHz, CDCl3)

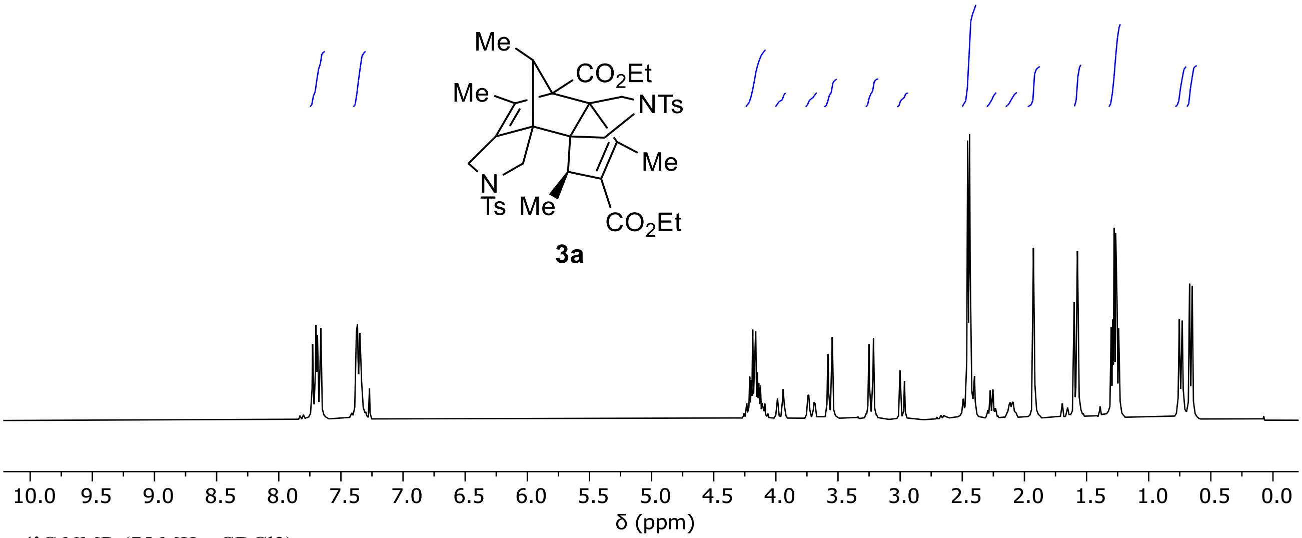

13C NMR (75 MHz, CDCl3)

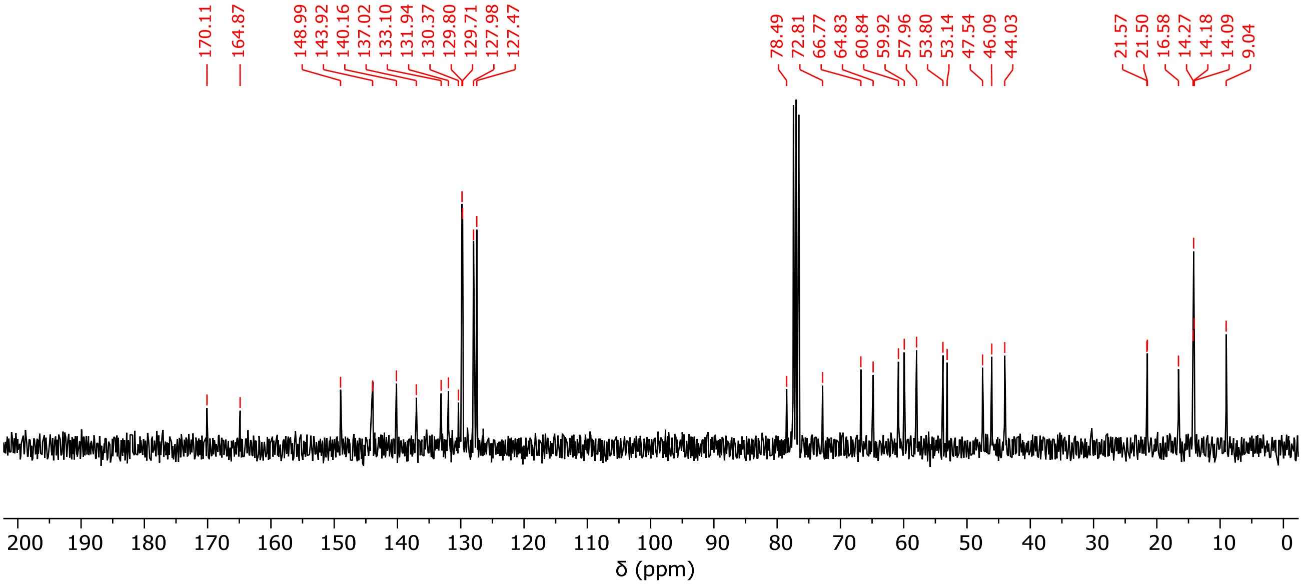

DEPT NMR (75 MHz, CDCl3)

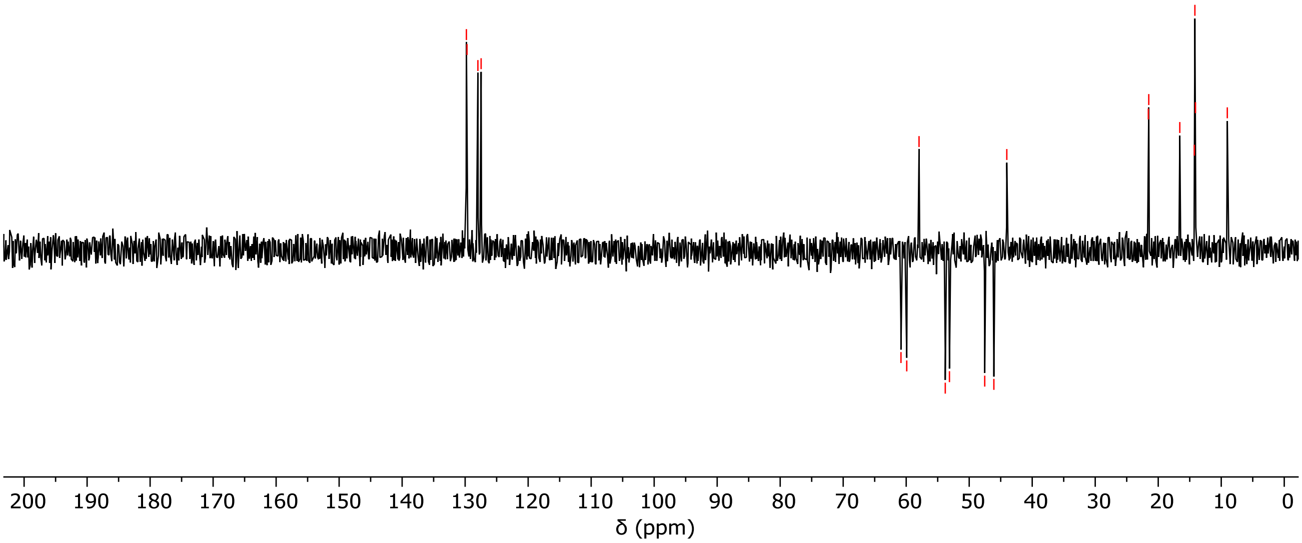

1H NMR (300 MHz, CD2Cl2)

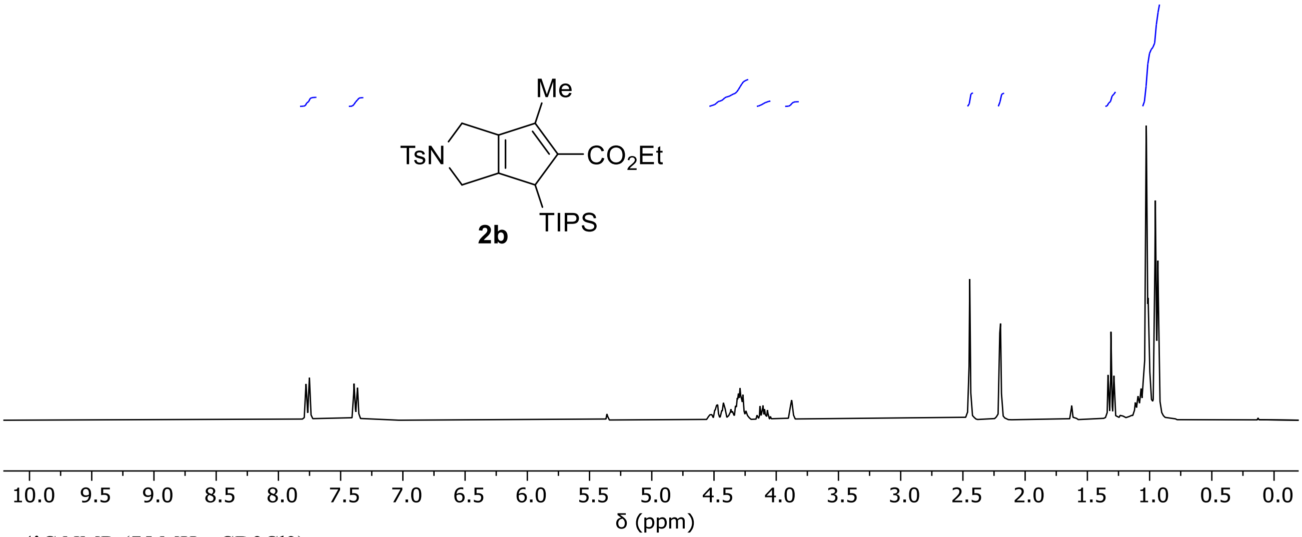

13C NMR (75 MHz, CD2Cl2)

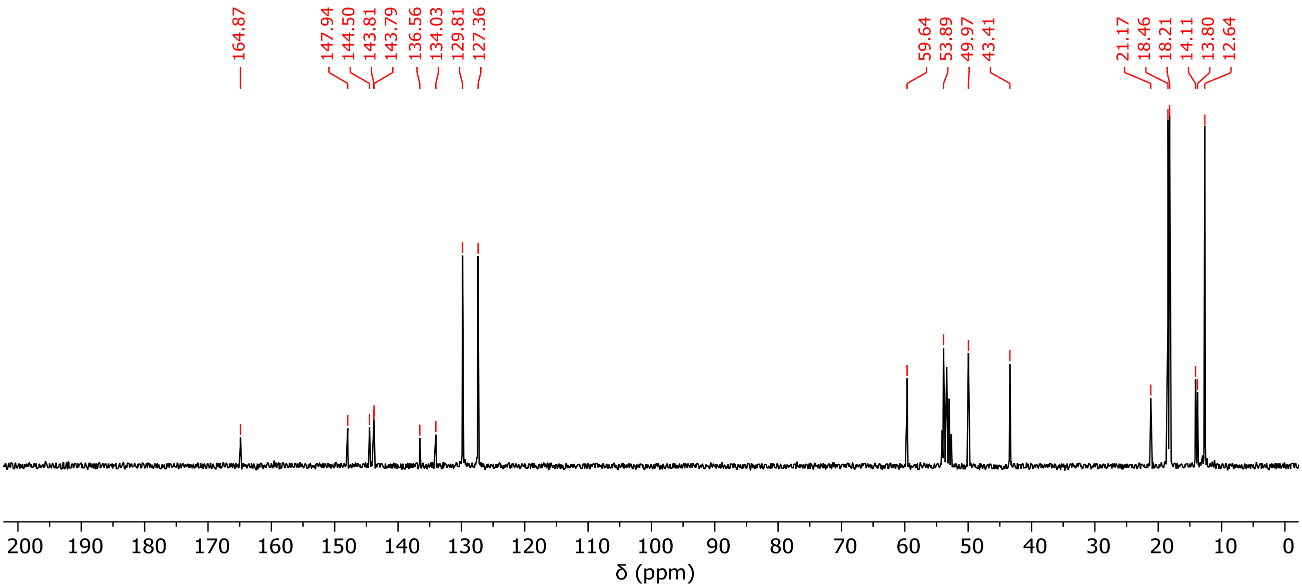

DEPT NMR (75 MHz, CD2Cl2)

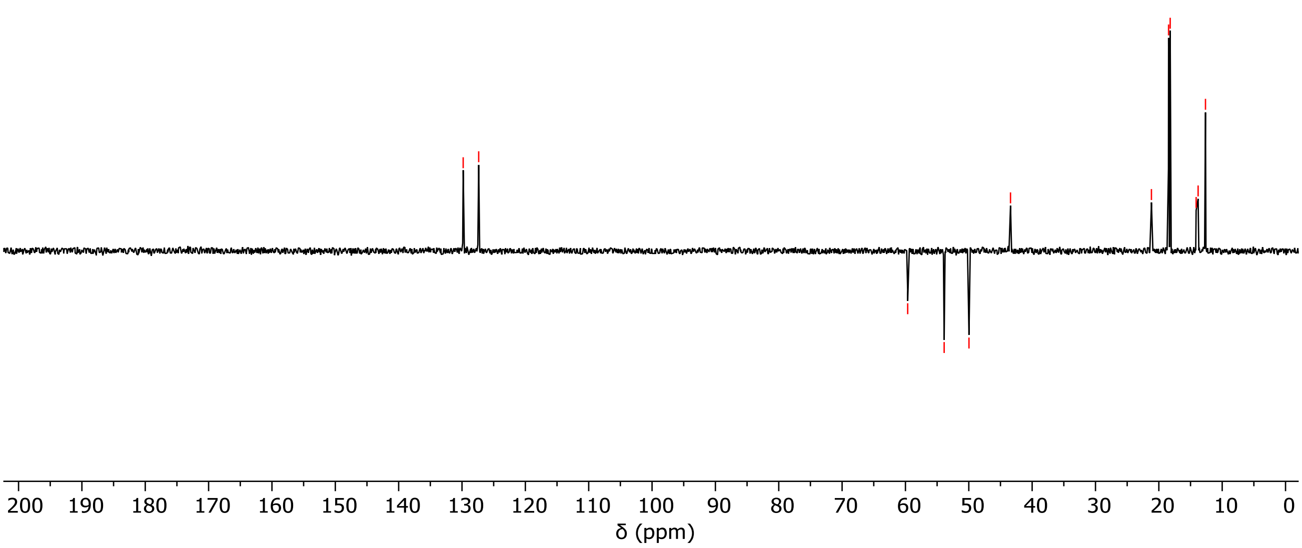

1H NMR (300 MHz, CDCl3)

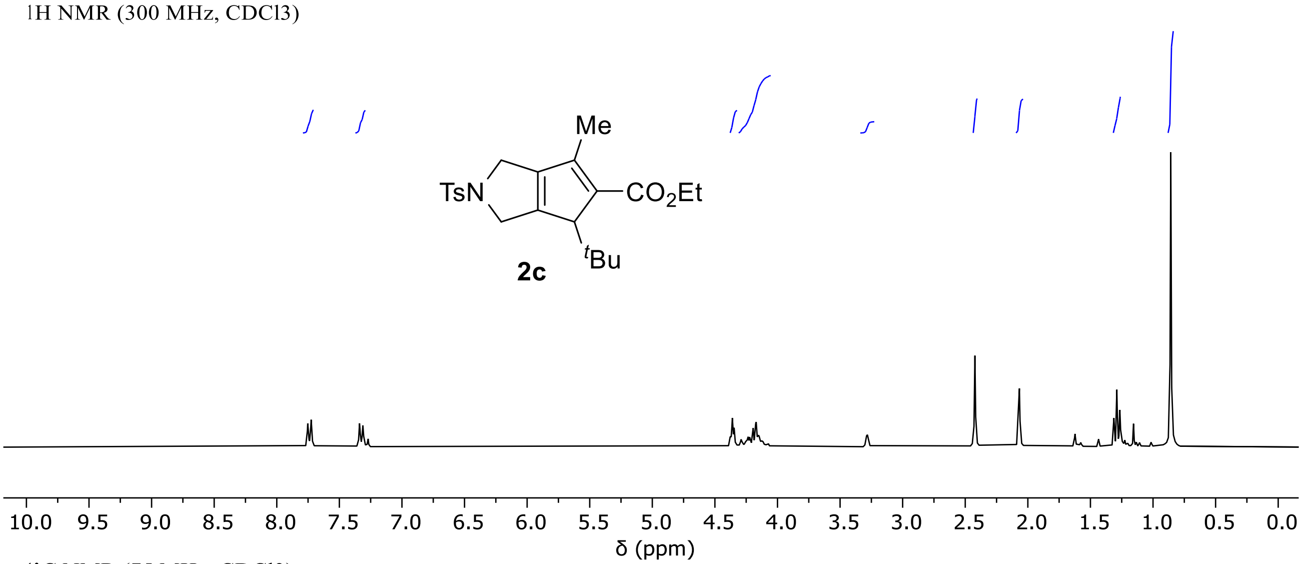

13C NMR (75 MHz, CDCl3)

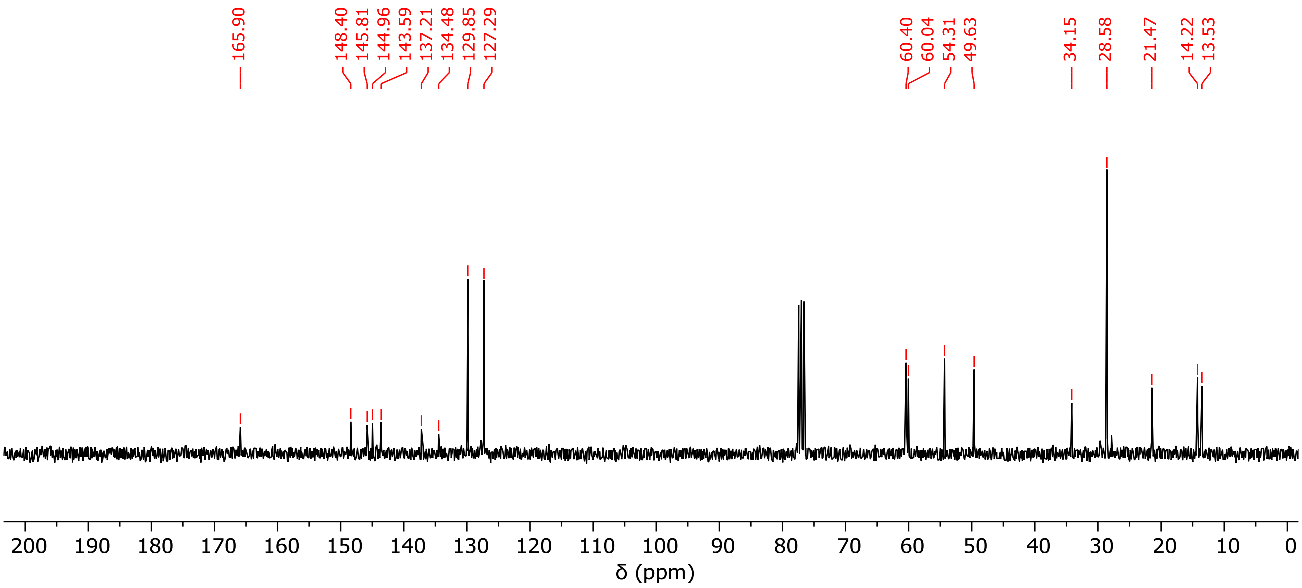

DEPT NMR (75 MHz, CDCl3)

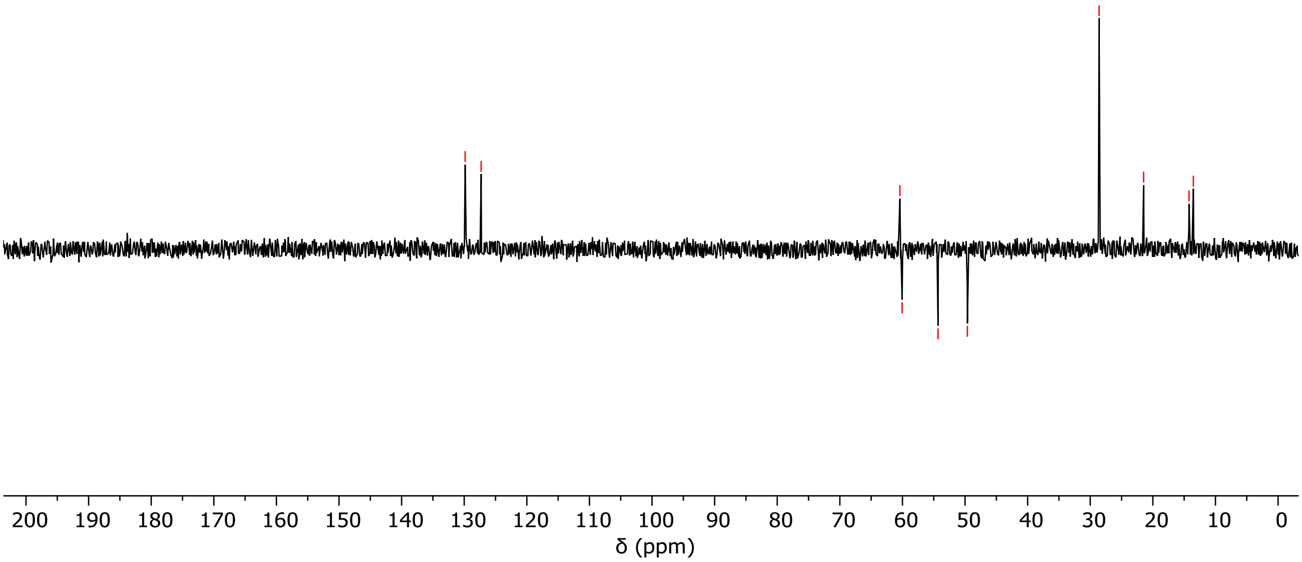

1H NMR (300 MHz, CD2Cl2)

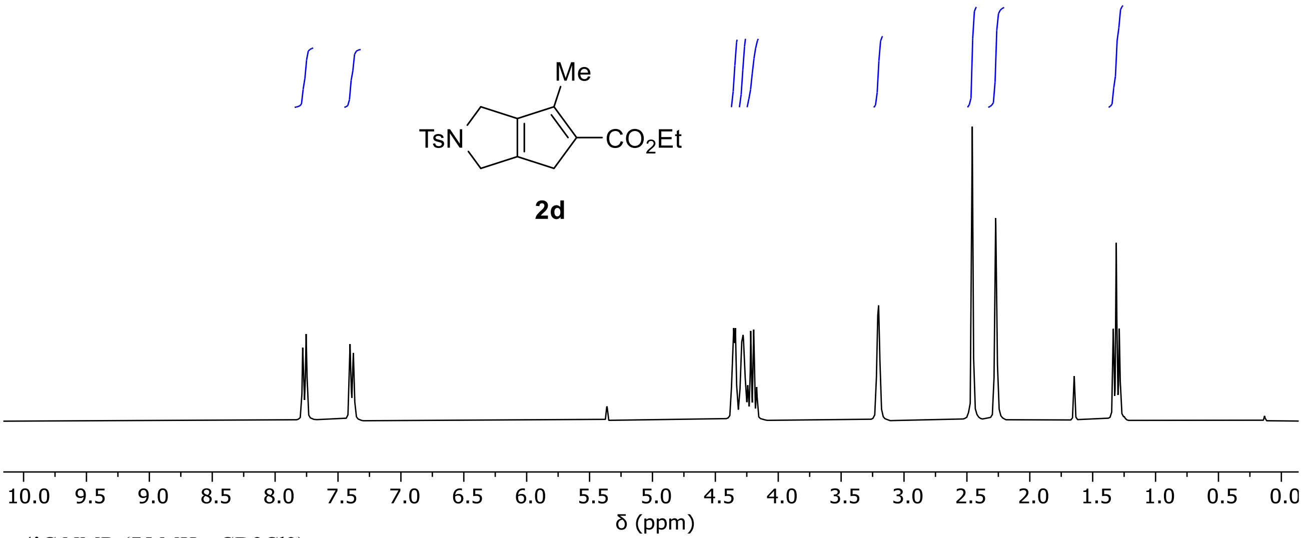

13C NMR (75 MHz, CD2Cl2)

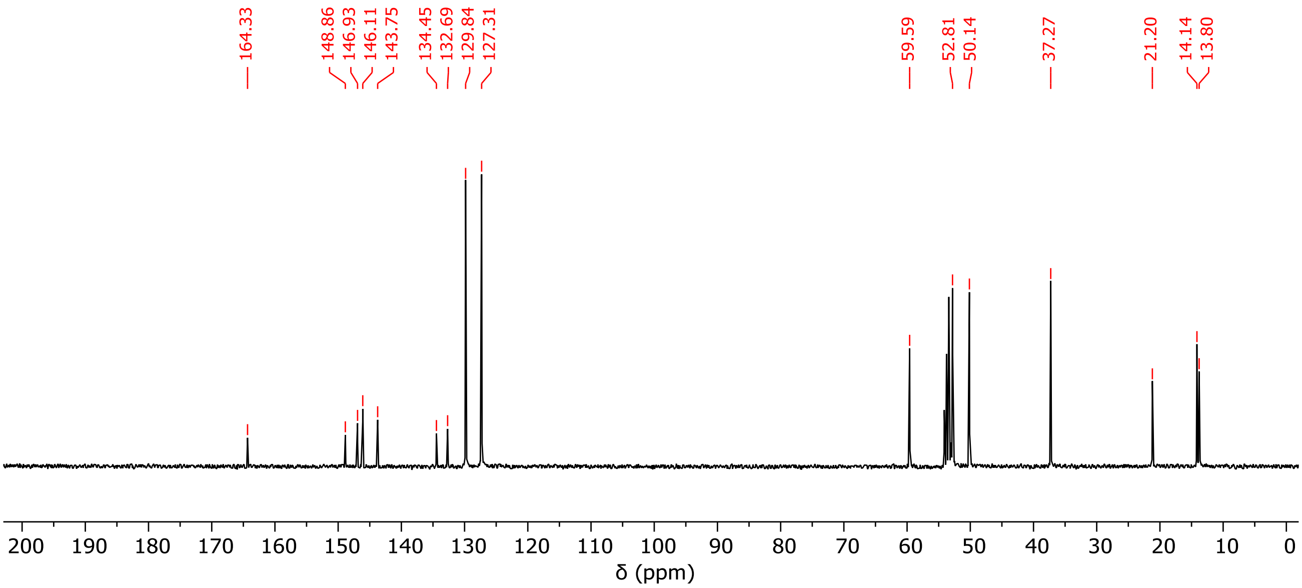

DEPT NMR (75 MHz, CD2Cl2)

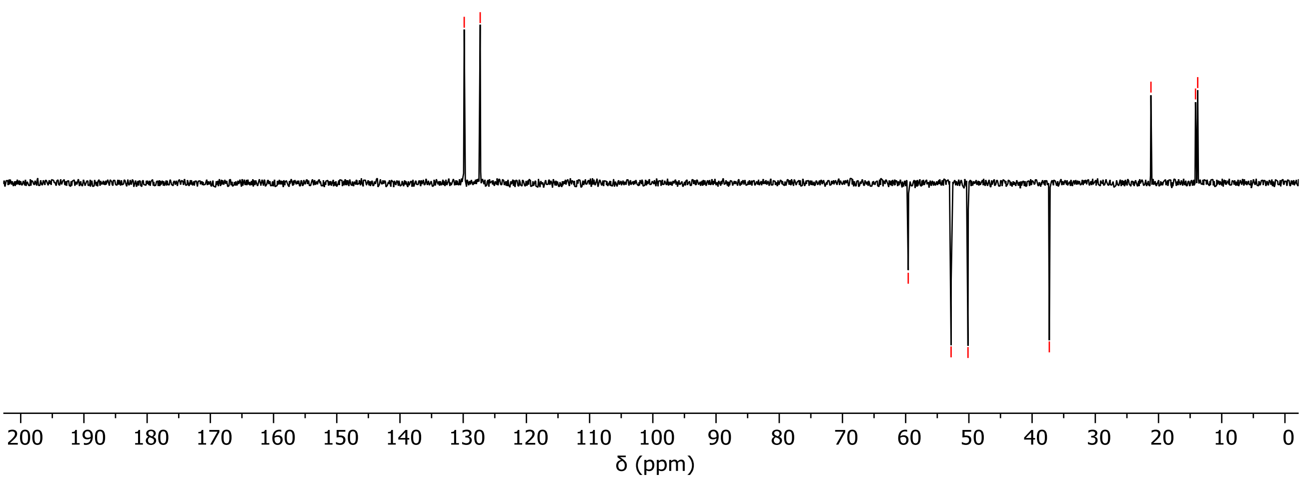

1H NMR (300 MHz, CDCl3)

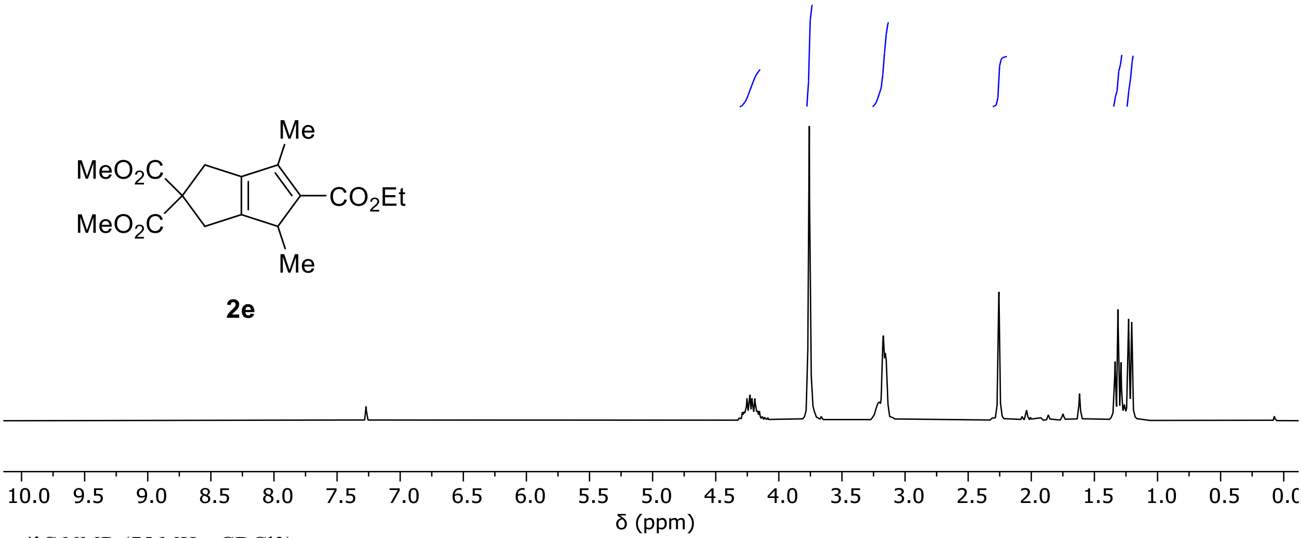

2e

13C NMR (75 MHz, CDCl3)

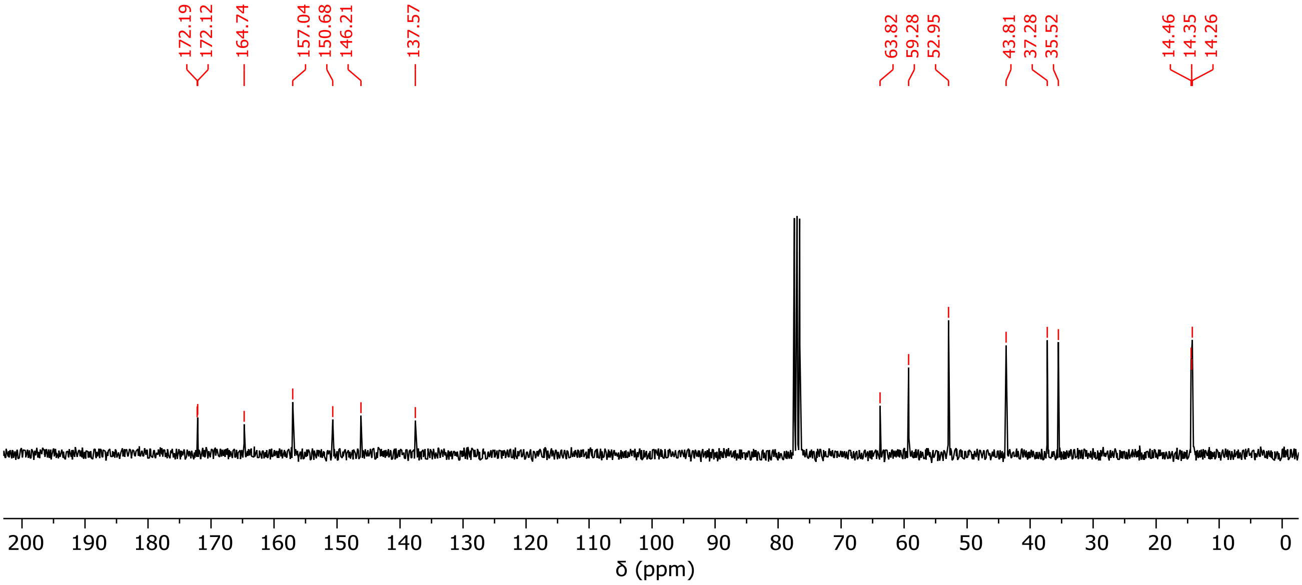

DEPT NMR (75 MHz, CDCl3)

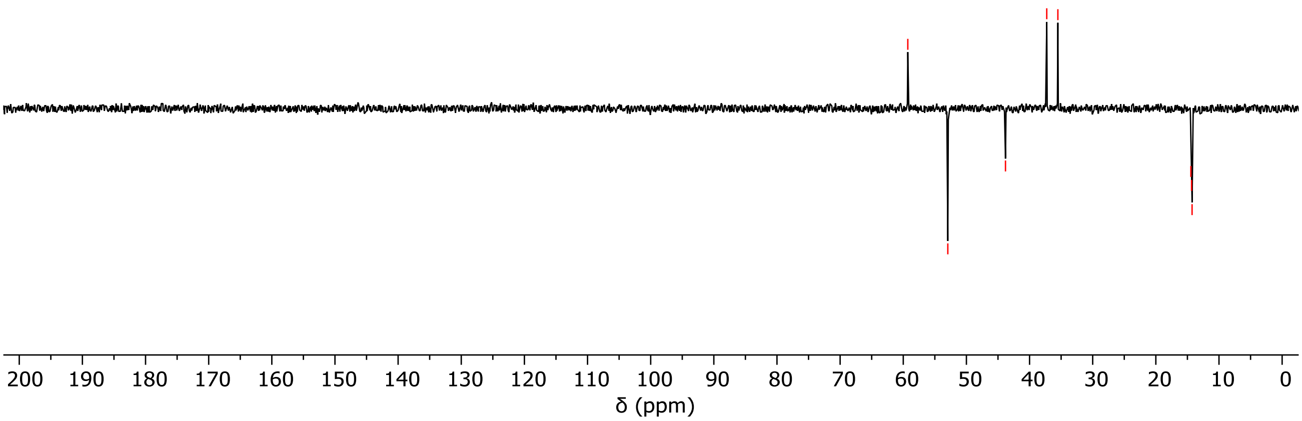

1H NMR (300 MHz, CDCl3)

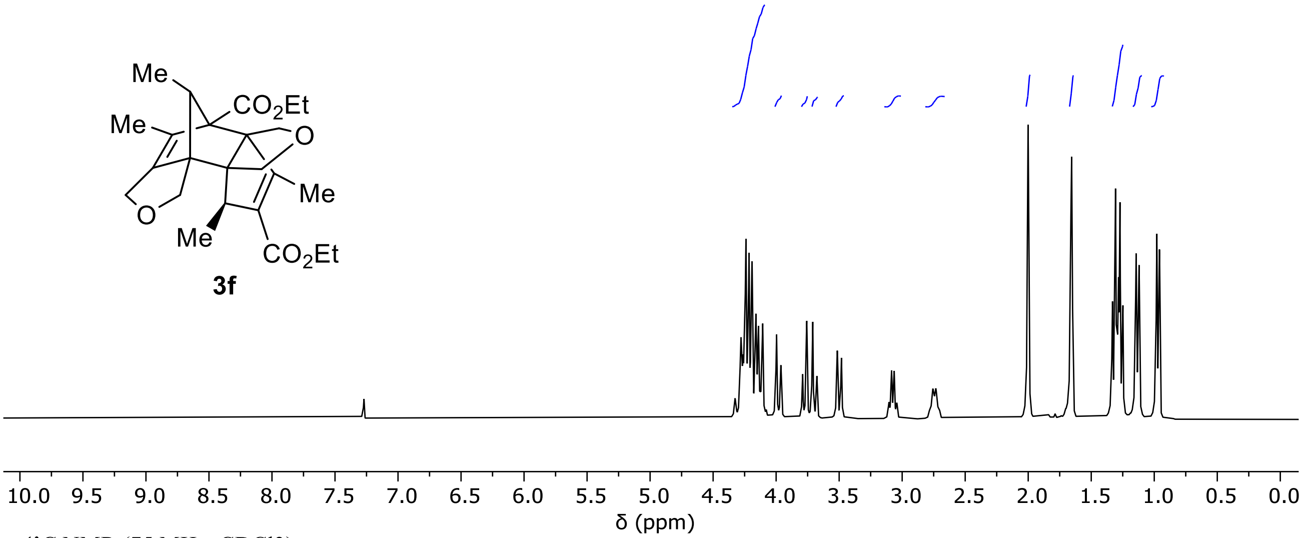

13C NMR (75 MHz, CDCl3)

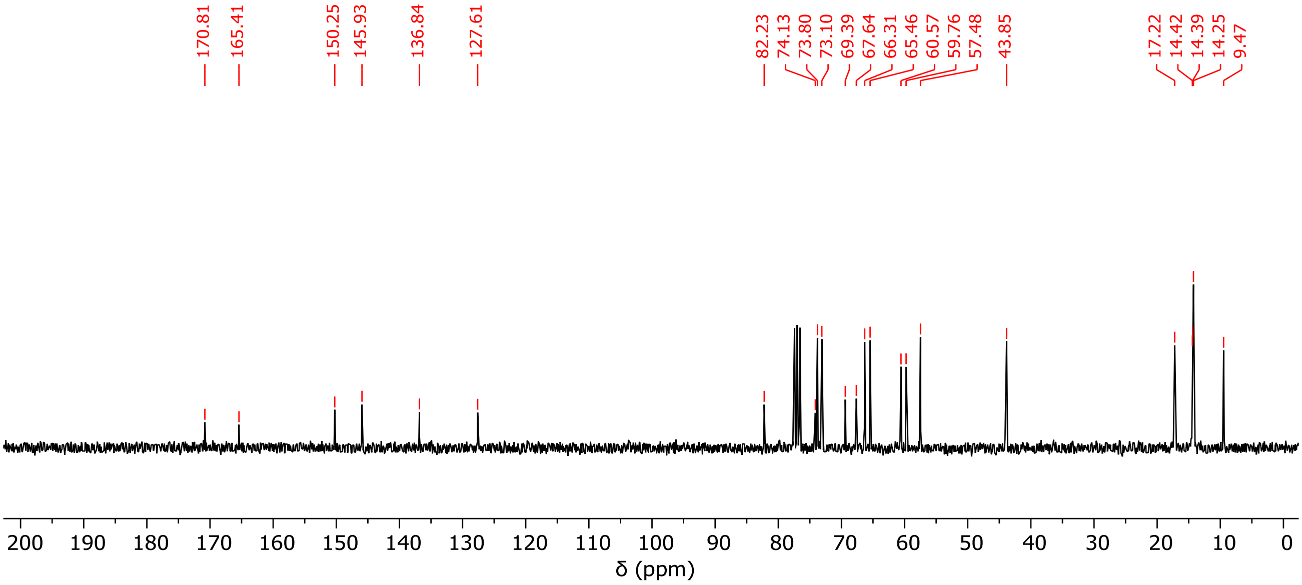

DEPT NMR (75 MHz, CDCl3)

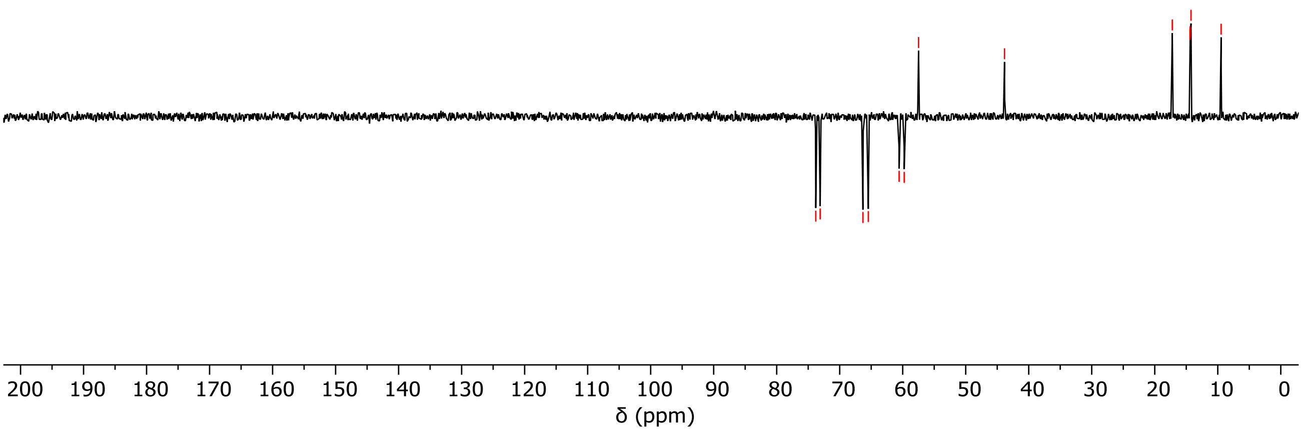

1H NMR (300 MHz, C6D6)

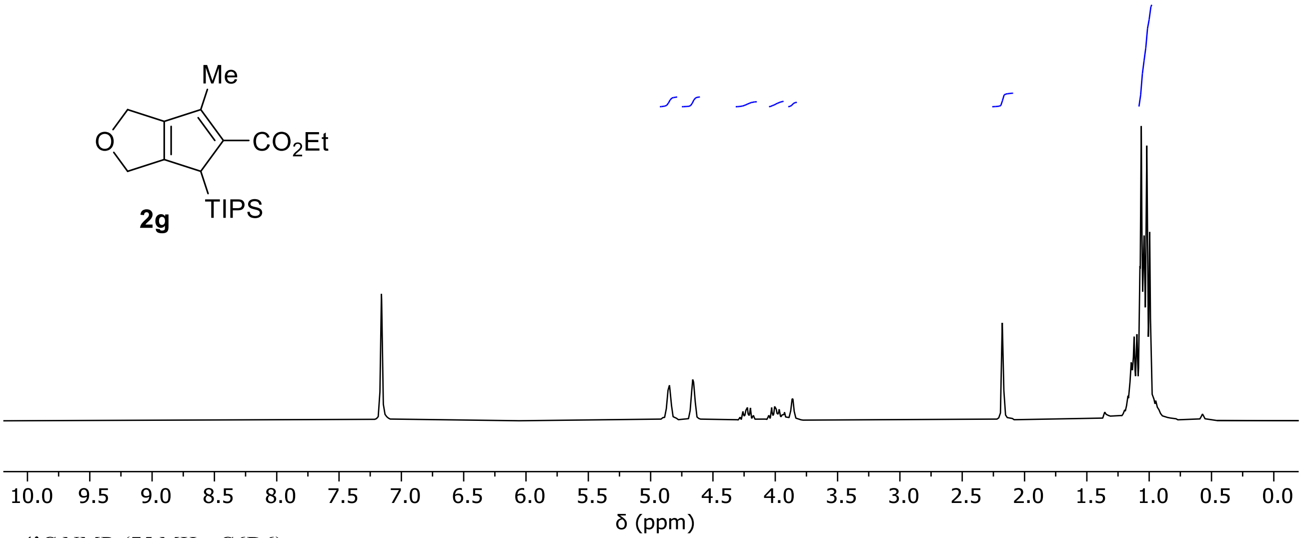

13C NMR (75 MHz, C6D6)

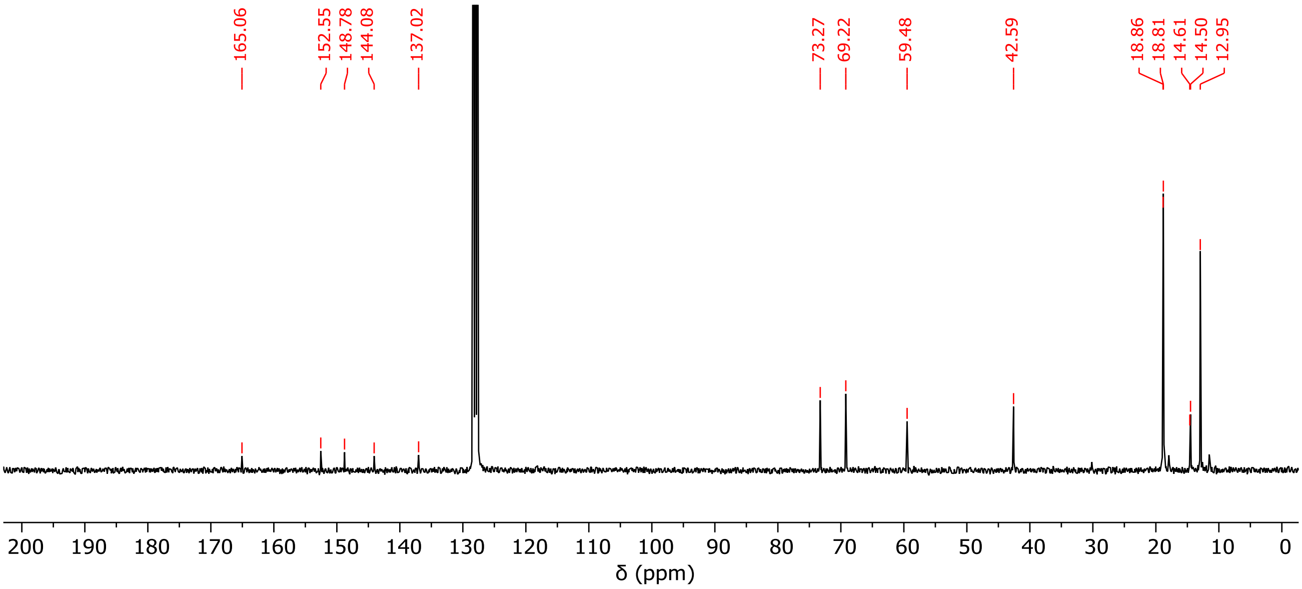

DEPT NMR (75 MHz, C6D6)

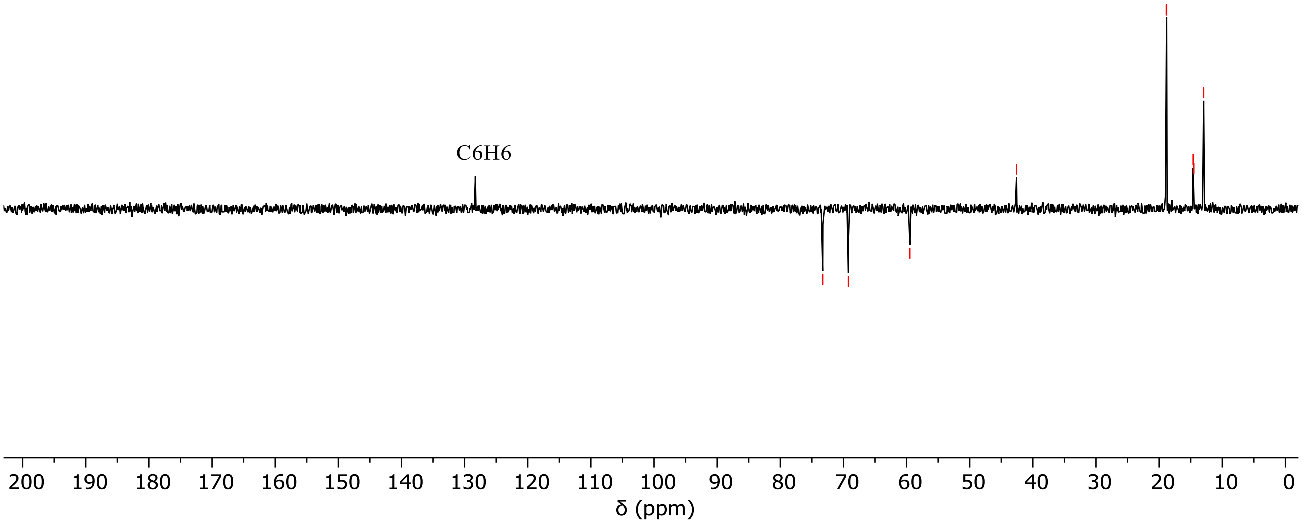

1H NMR (300 MHz, CDCl3)

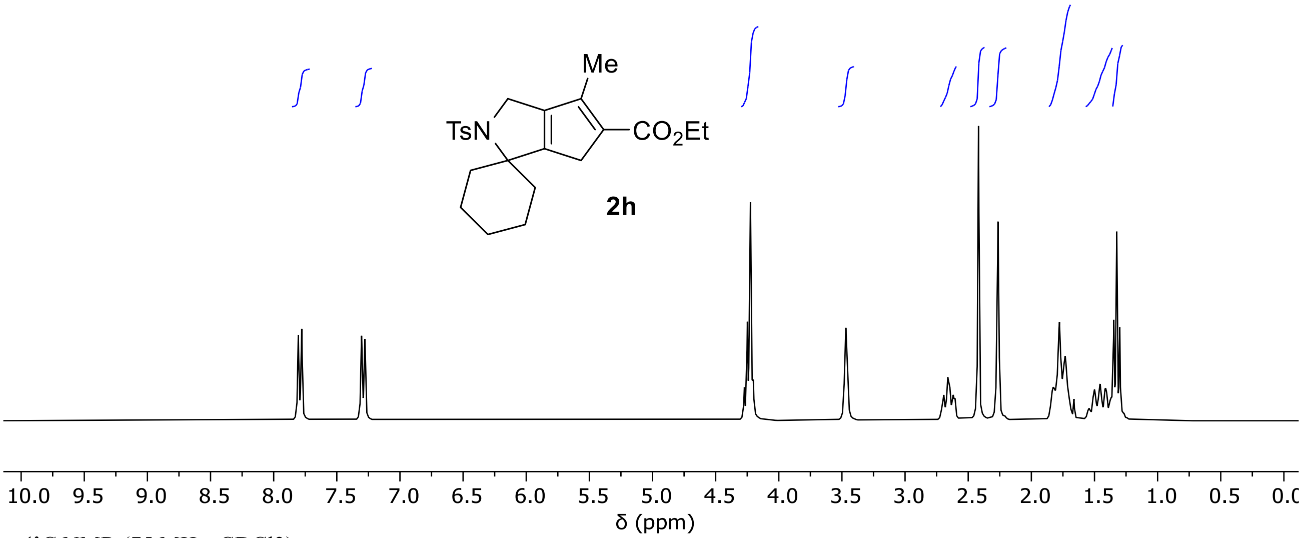

13C NMR (75 MHz, CDCl3)

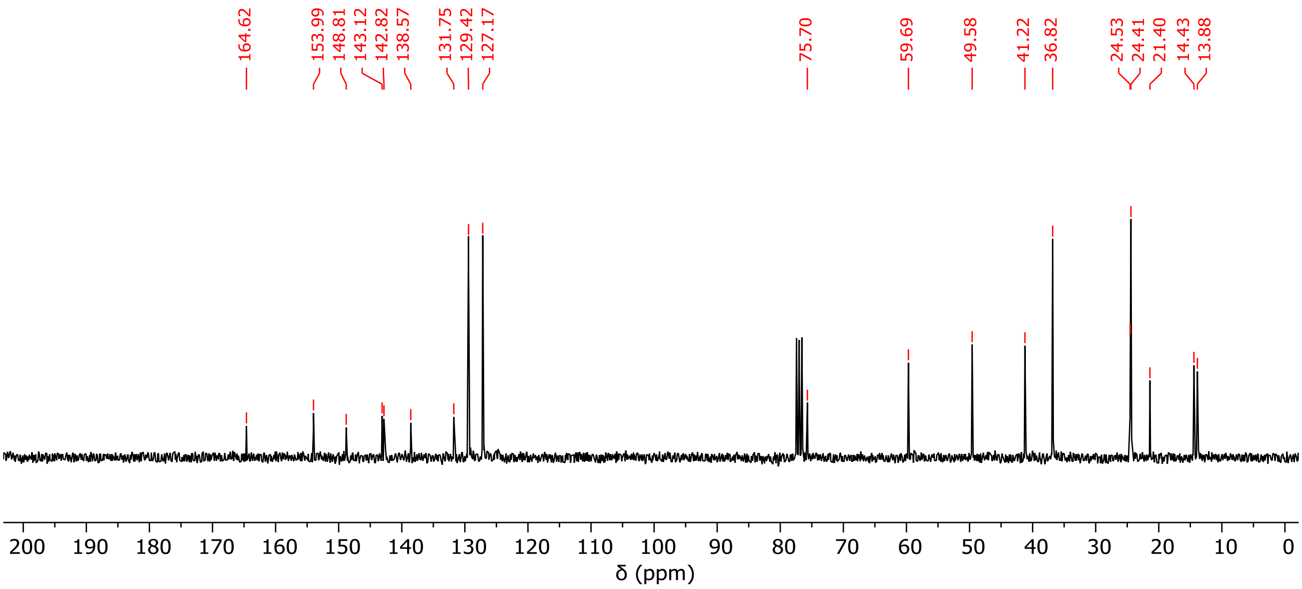

DEPT NMR (75 MHz, CDCl3)

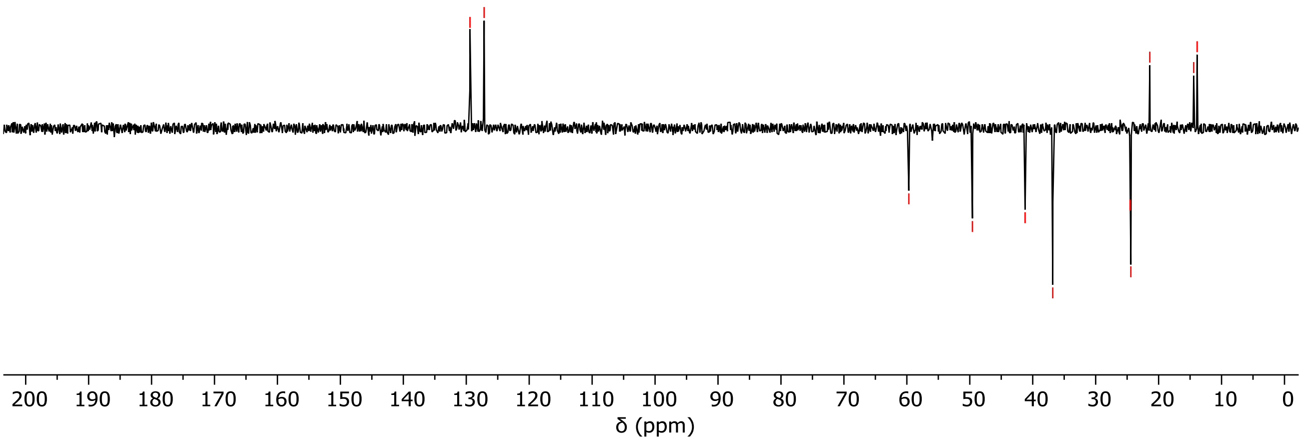

1H NMR (300 MHz, CDCl3)

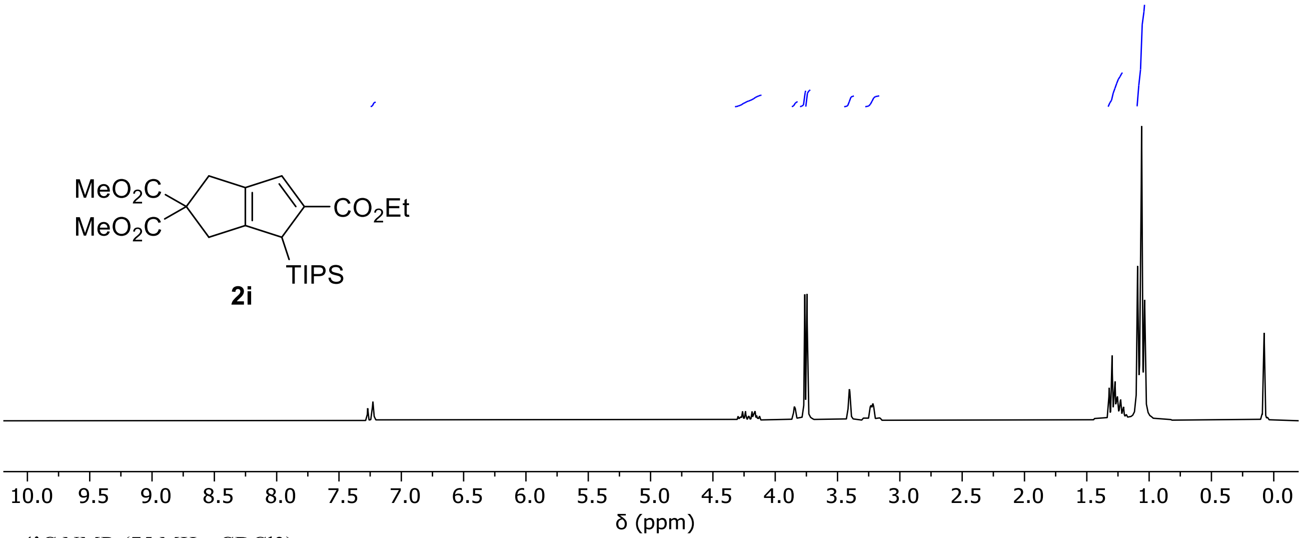

13C NMR (75 MHz, CDCl3)

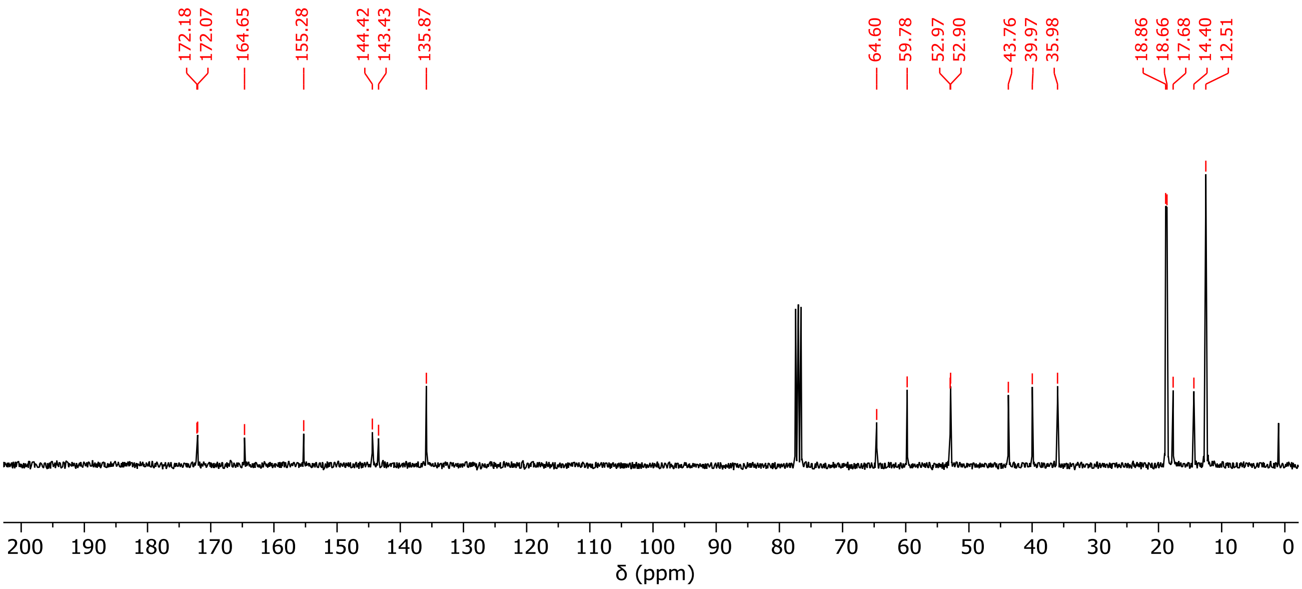

DEPT NMR (75 MHz, CDCl3)

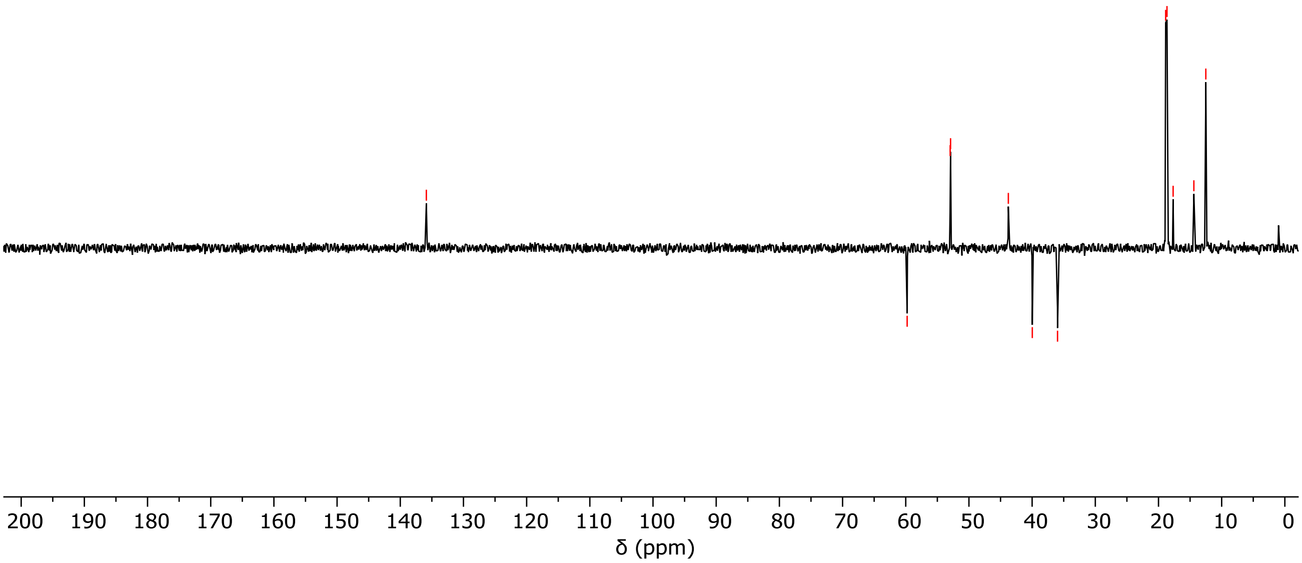

1H NMR (500 MHz, CDCl3)

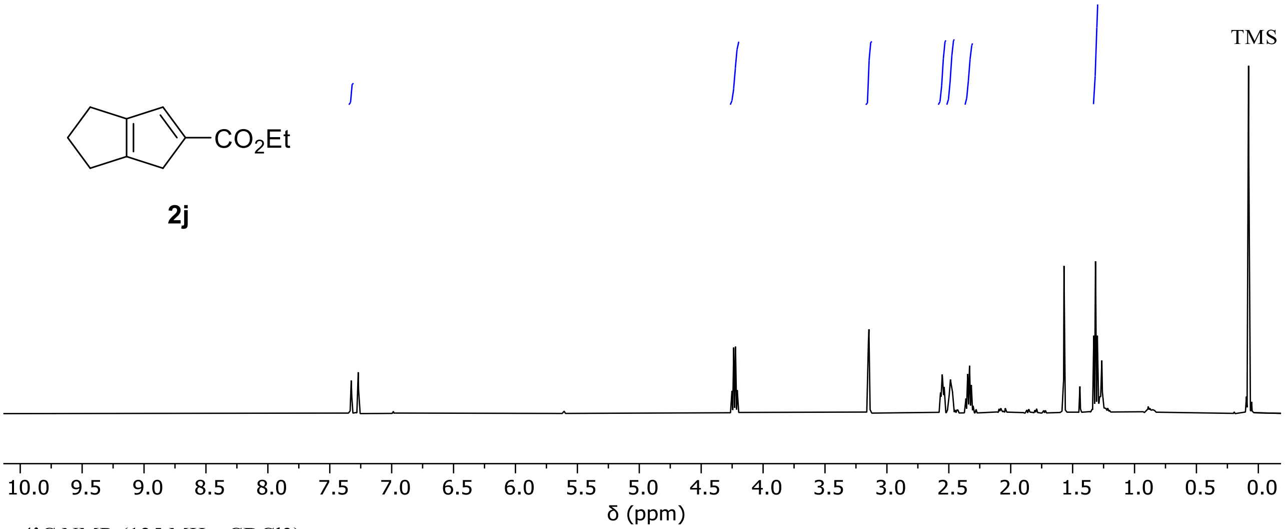

2j

13C NMR (125 MHz, CDCl3)

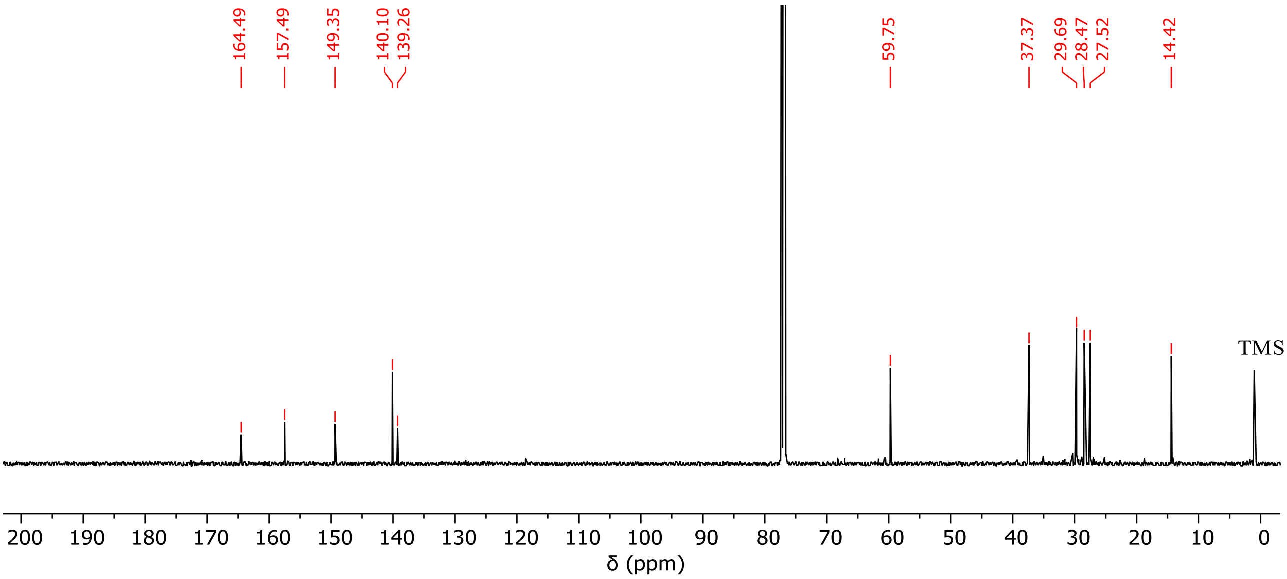

DEPT NMR (125 MHz, CDCl3)

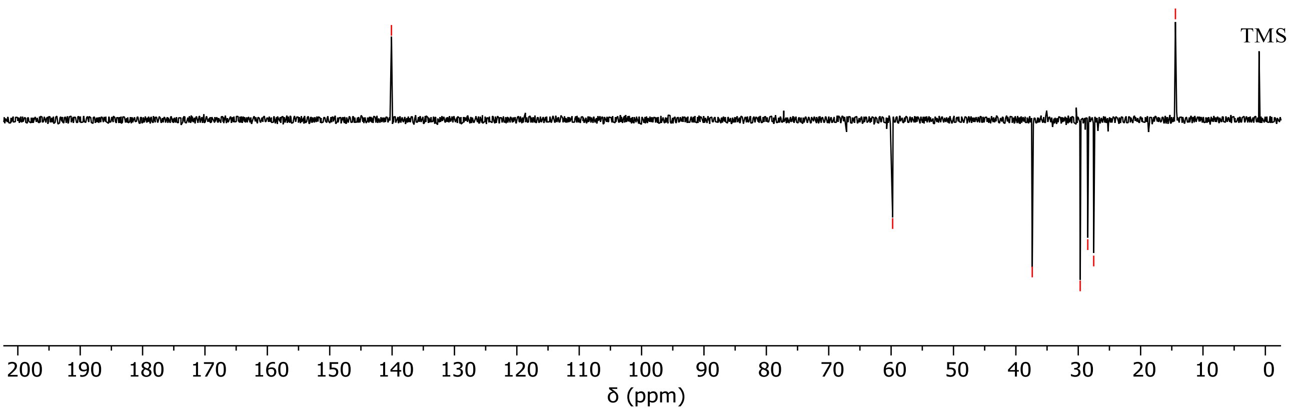

1H NMR (500 MHz, CDCl3)

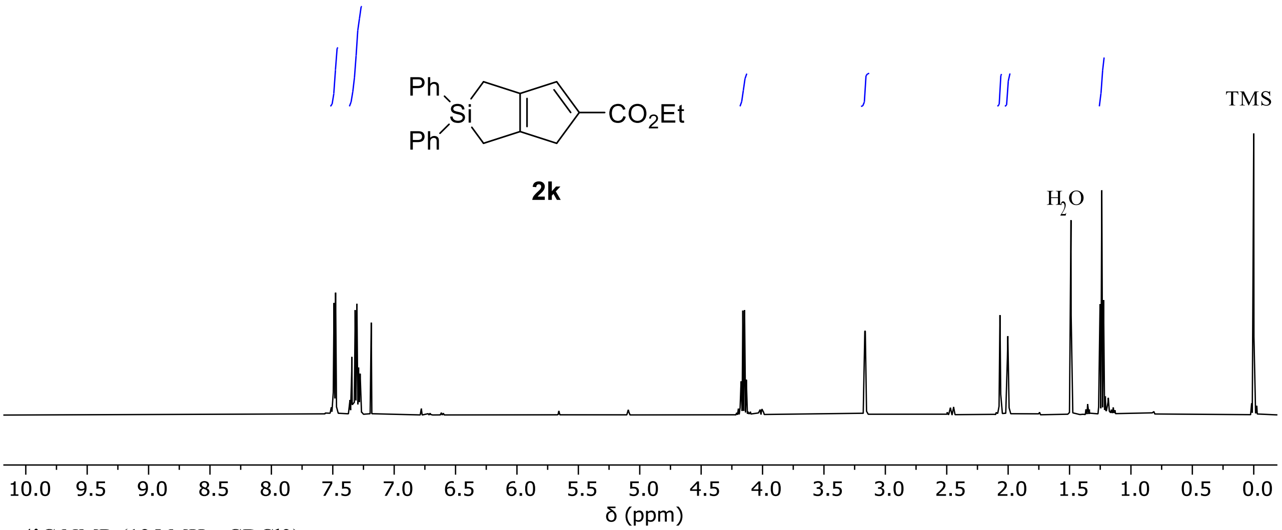

13C NMR (125 MHz, CDCl3)

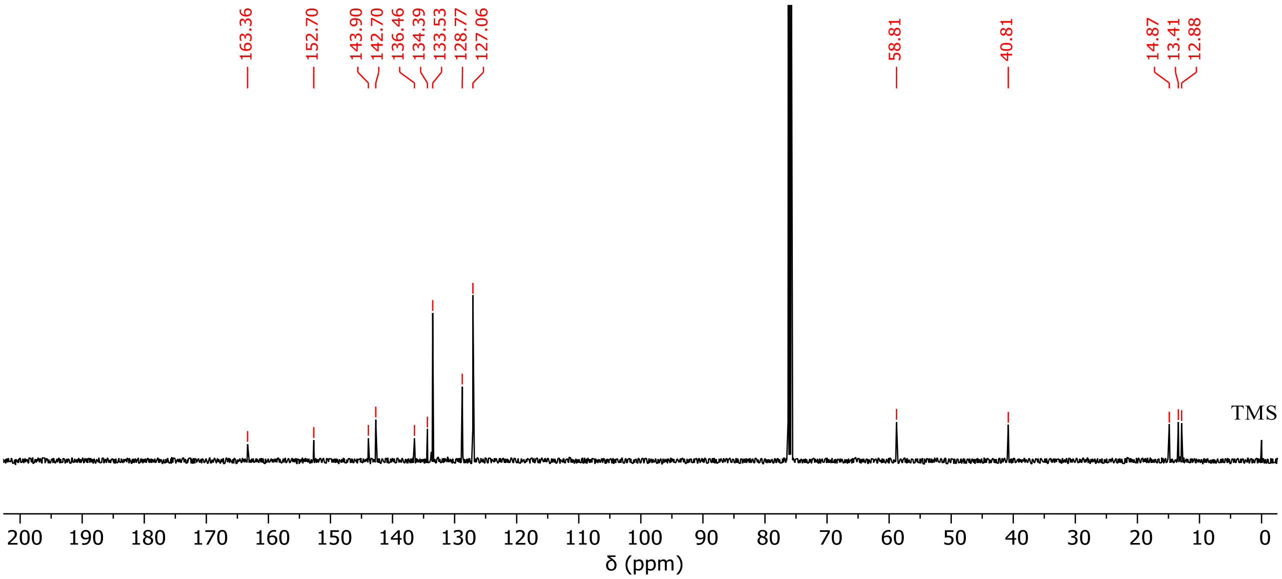

DEPT NMR (125 MHz, CDCl3)

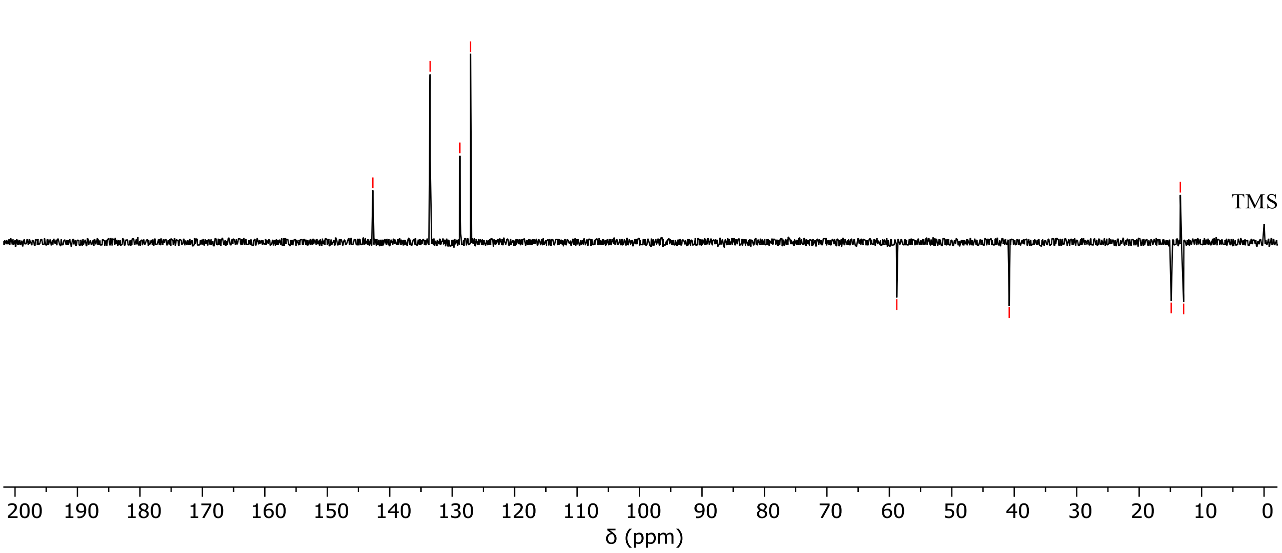

1H NMR (300 MHz, CDCl3)

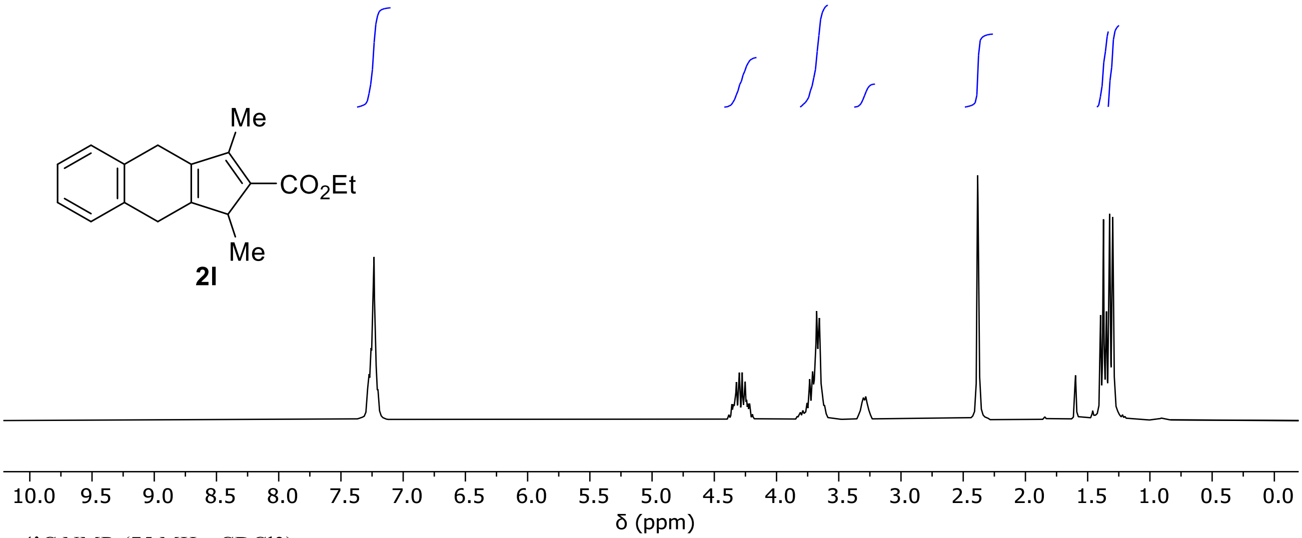

13C NMR (75 MHz, CDCl3)

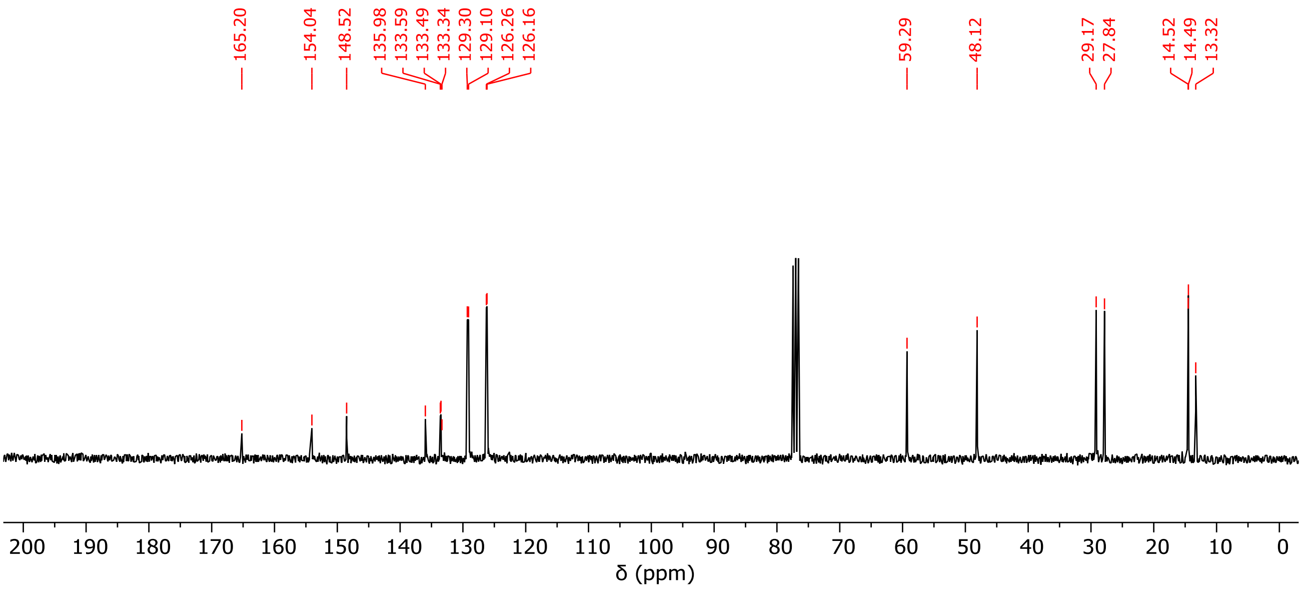

DEPT NMR (75 MHz, CDCl3)

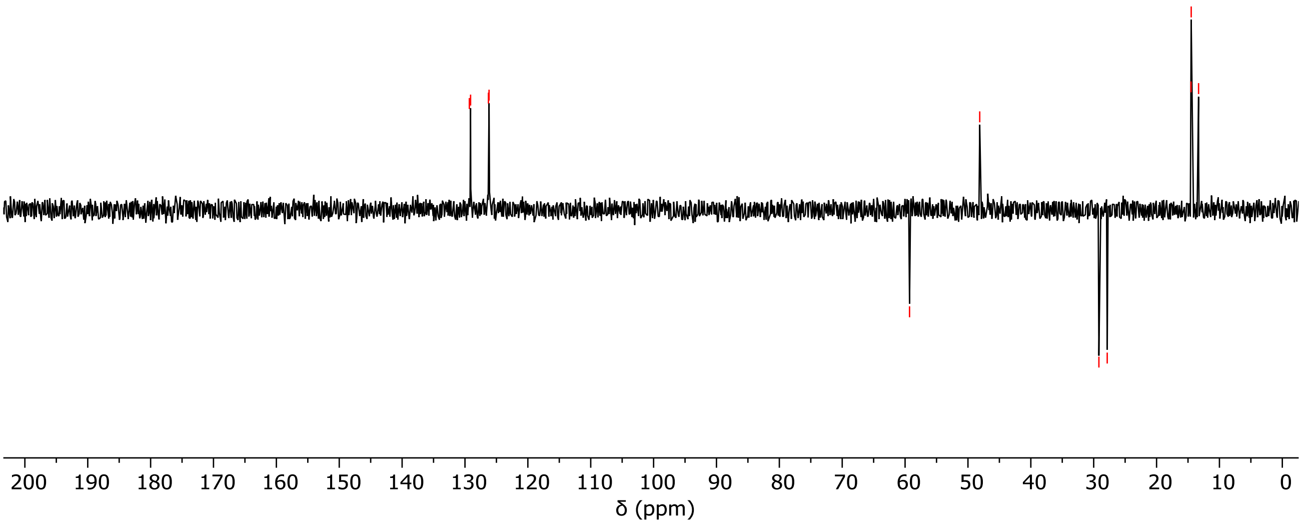

1H NMR (500 MHz, CDCl3)

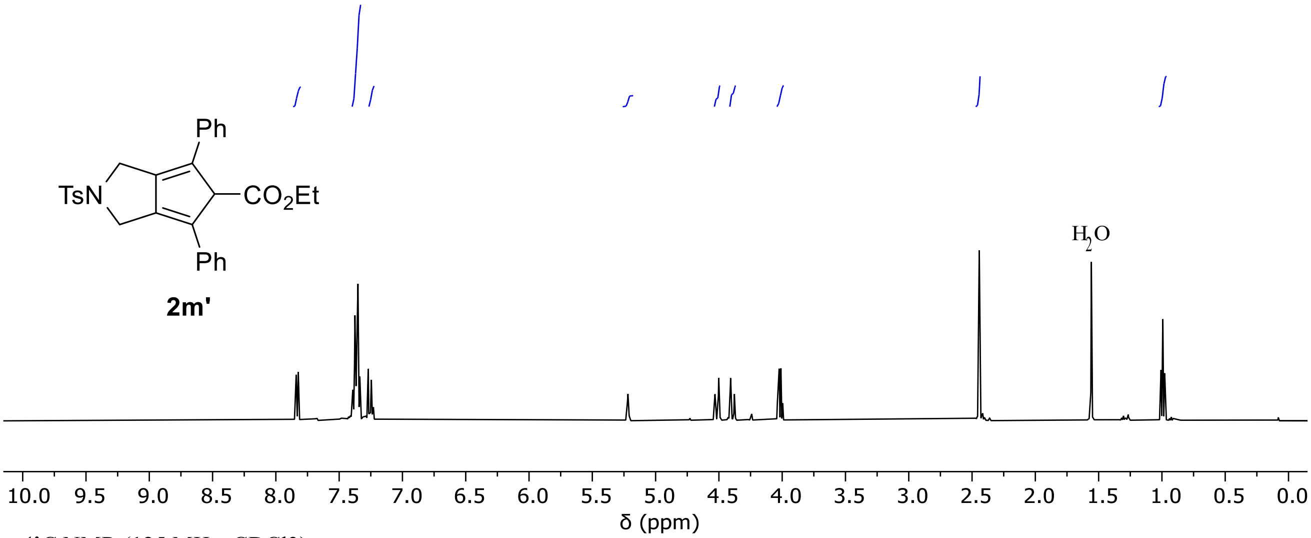

13C NMR (125 MHz, CDCl3)

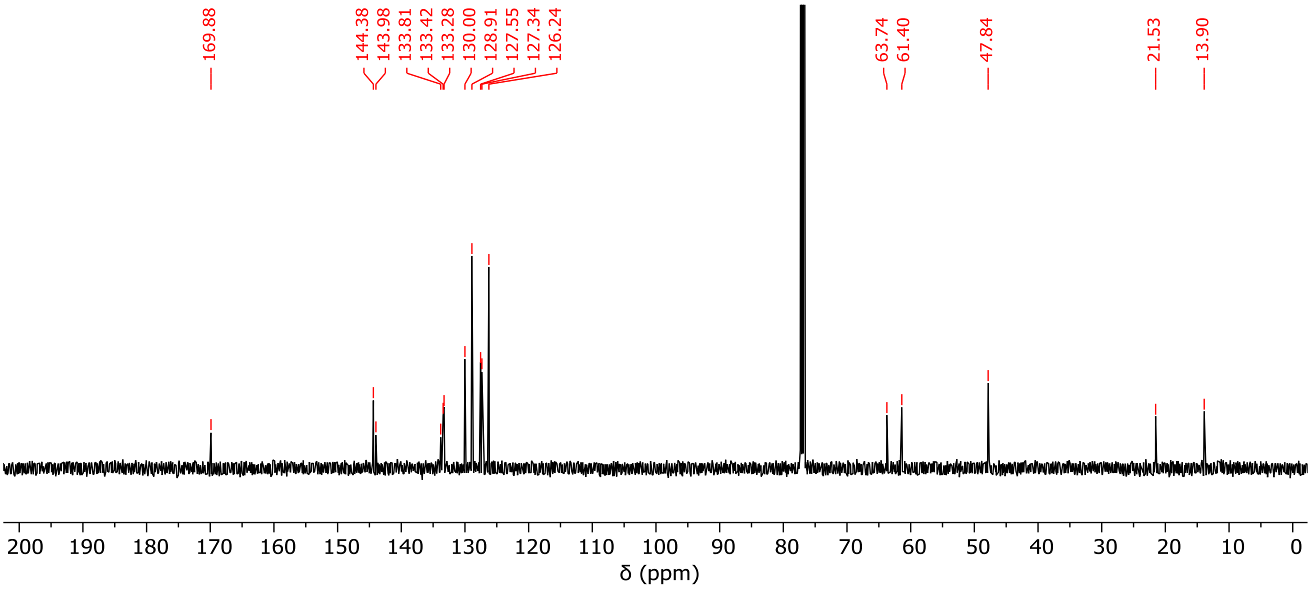

DEPT NMR (125 MHz, CDCl3)

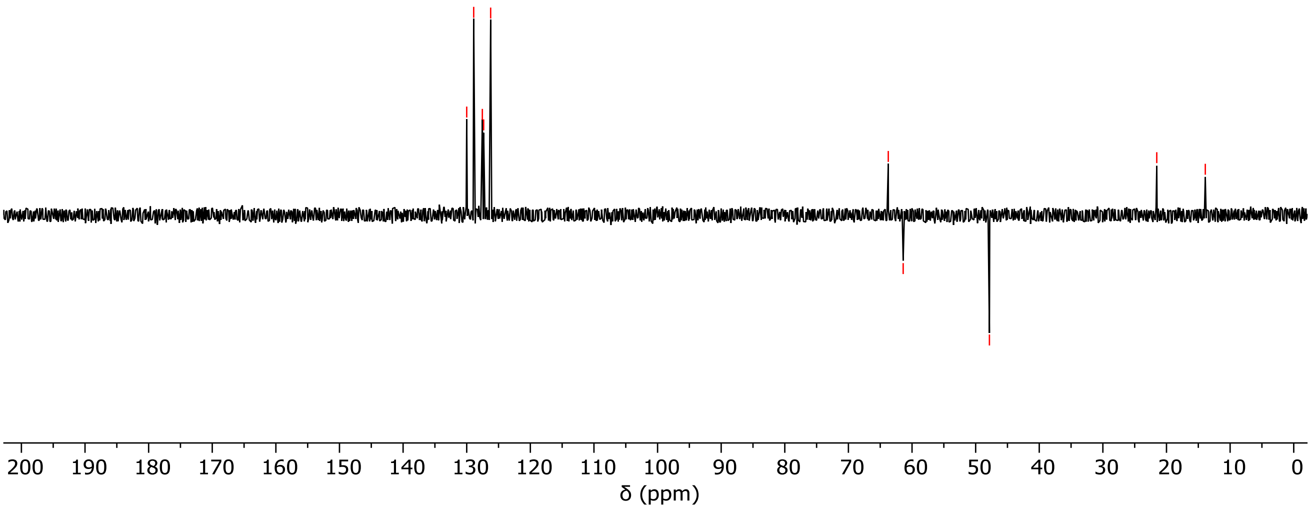

1H NMR (500 MHz, CDCl3)

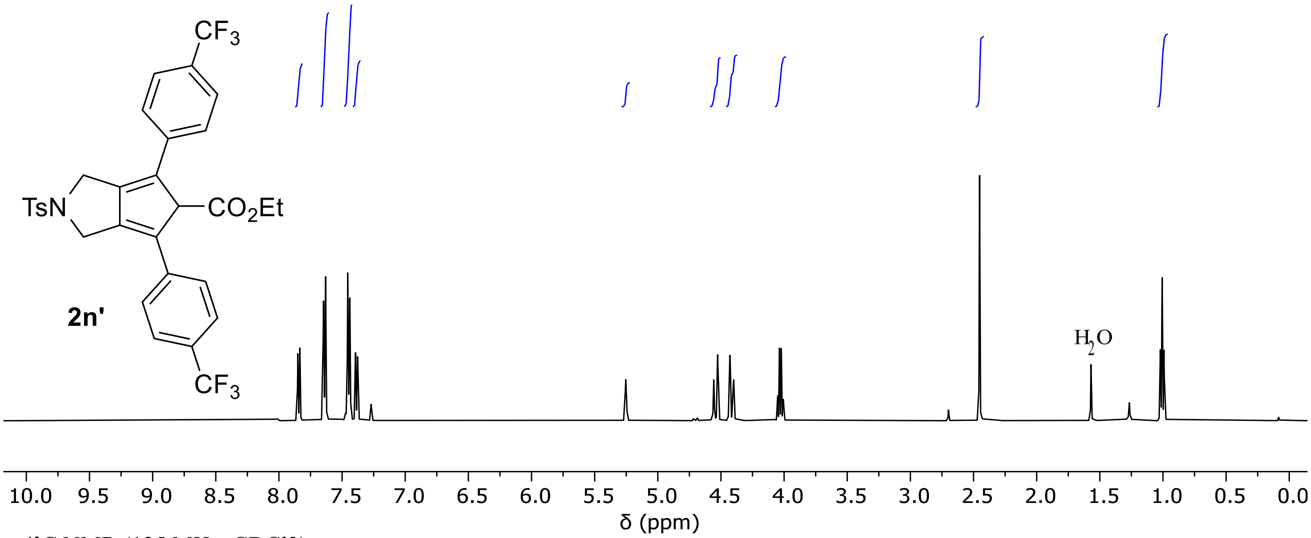

13C NMR (125 MHz, CDCl3)

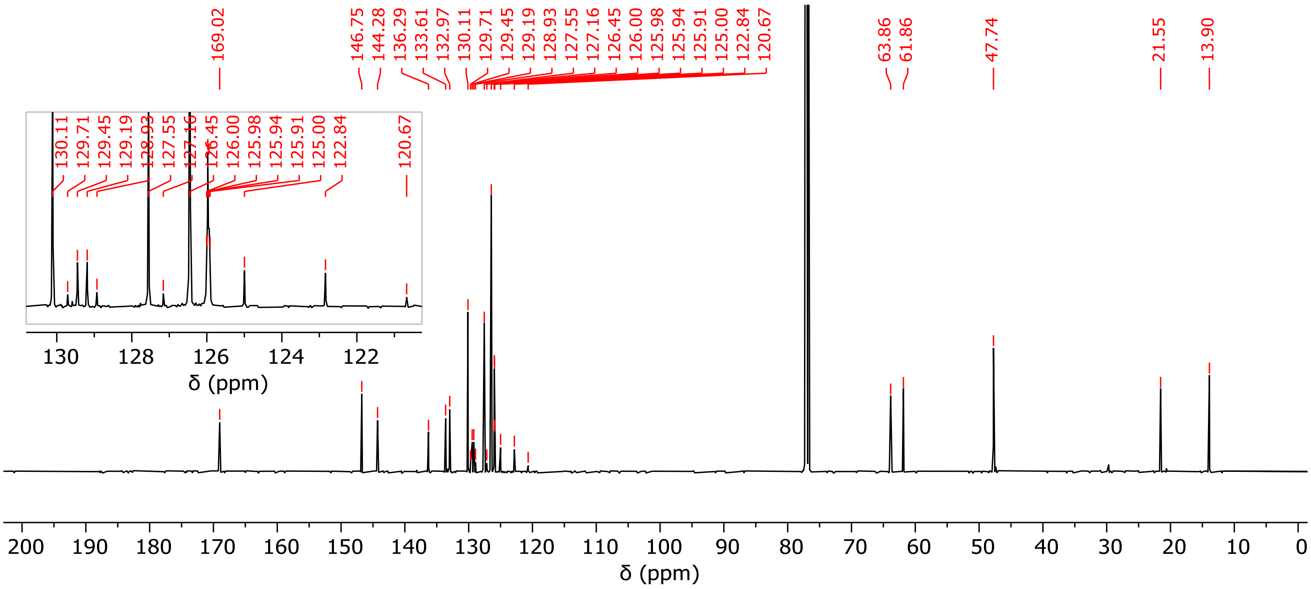

DEPT NMR (125 MHz, CDCl3)

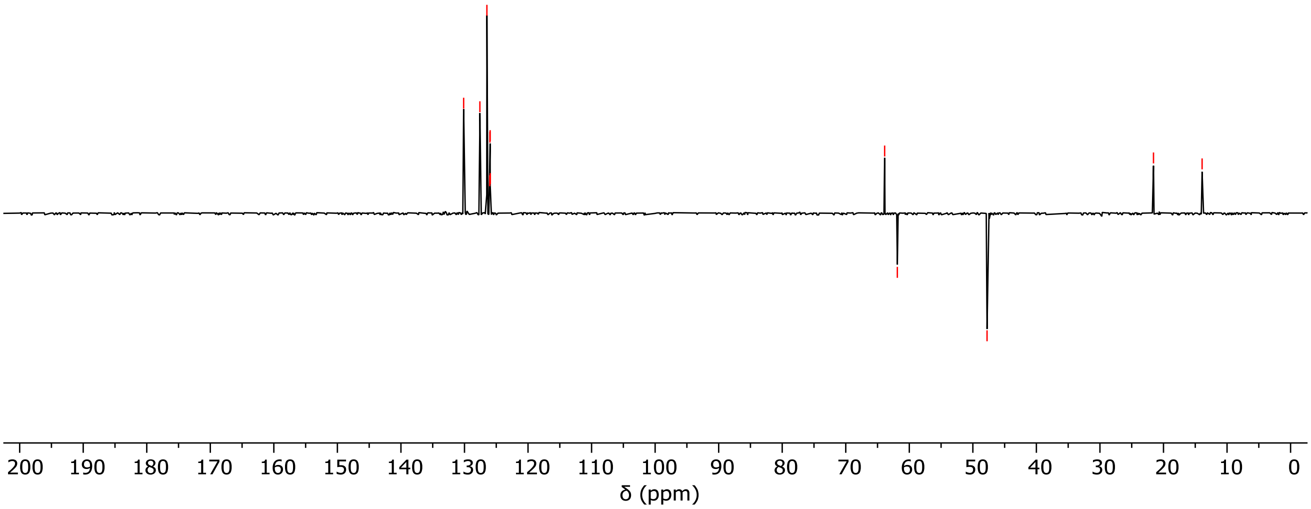

1H NMR (500 MHz, CDCl3)

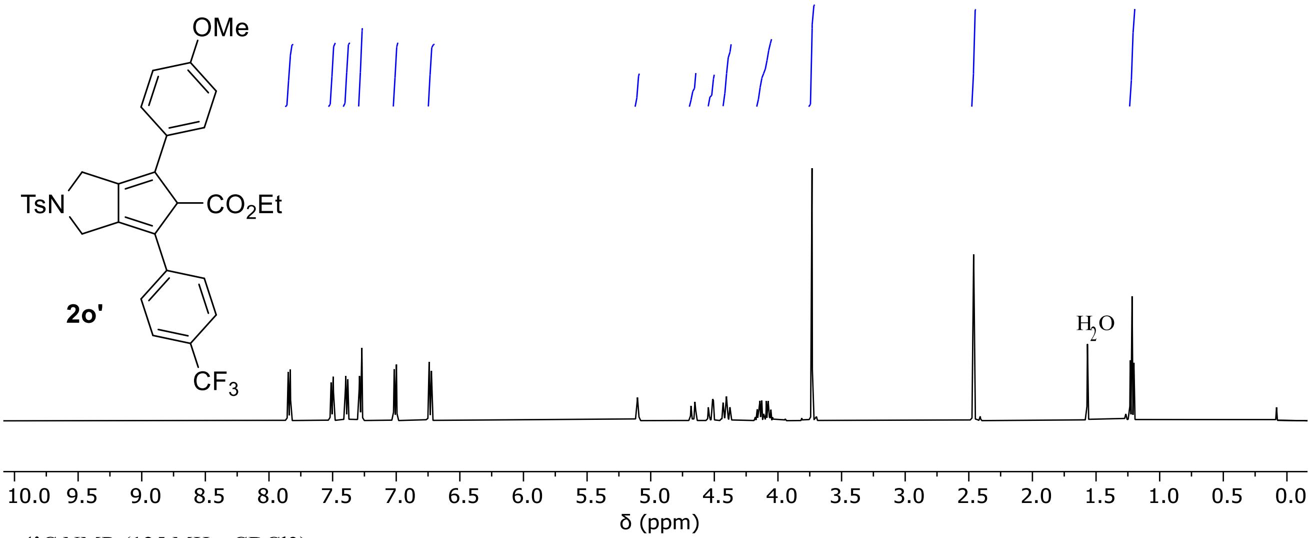

13C NMR (125 MHz, CDCl3)

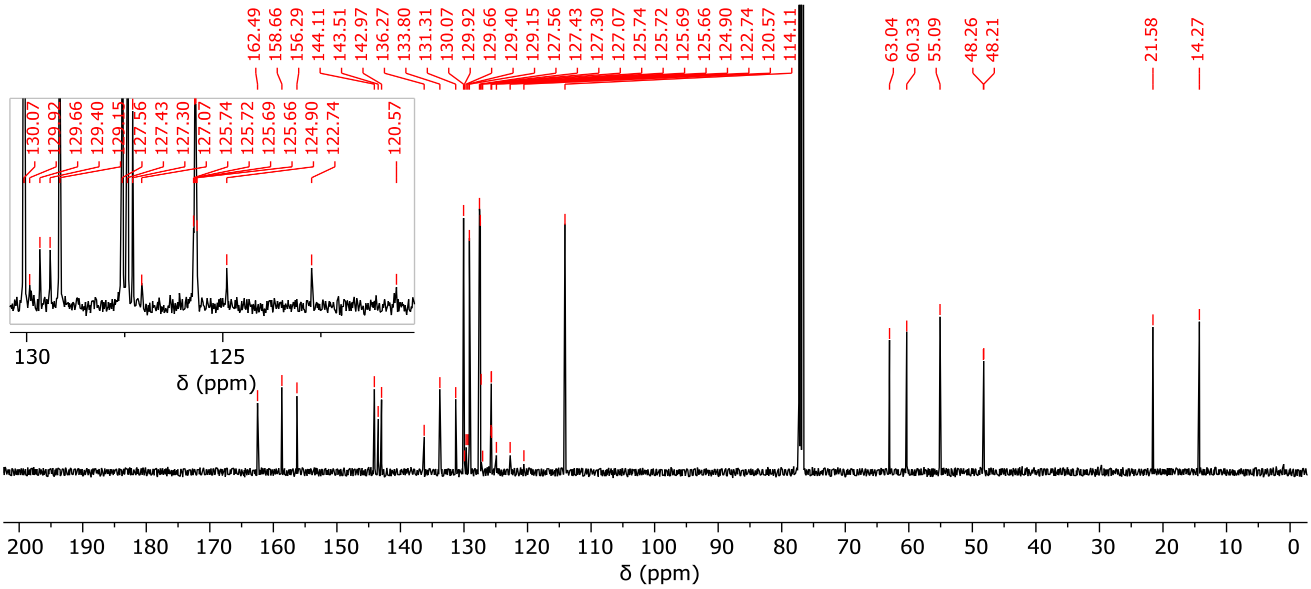

DEPT NMR (125 MHz, CDCl3)

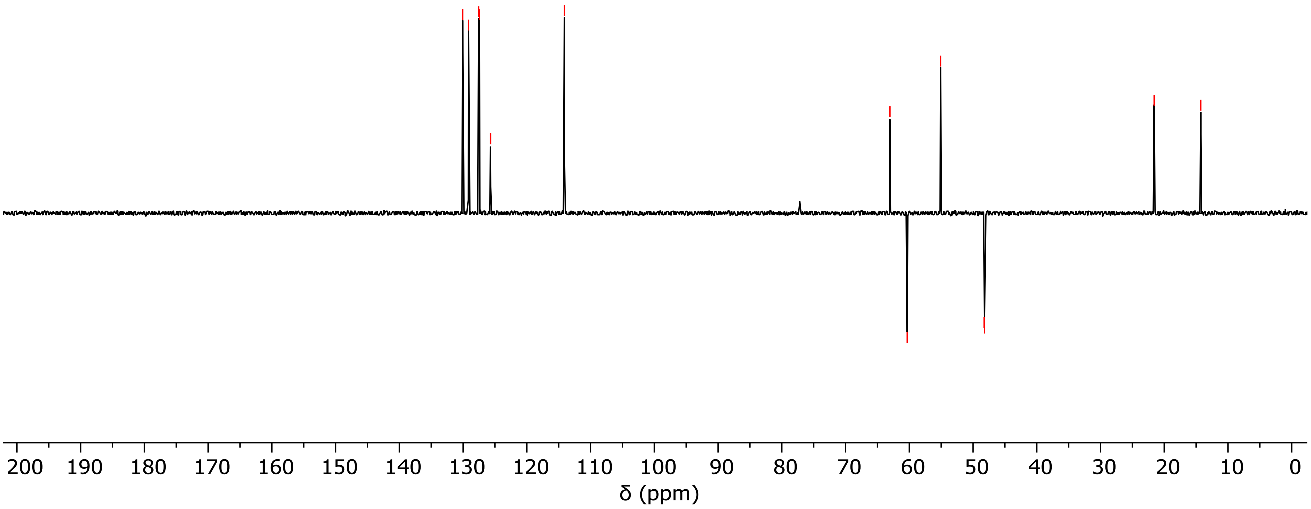

^{19}F NMR (470 MHz, $CDCl_3$)

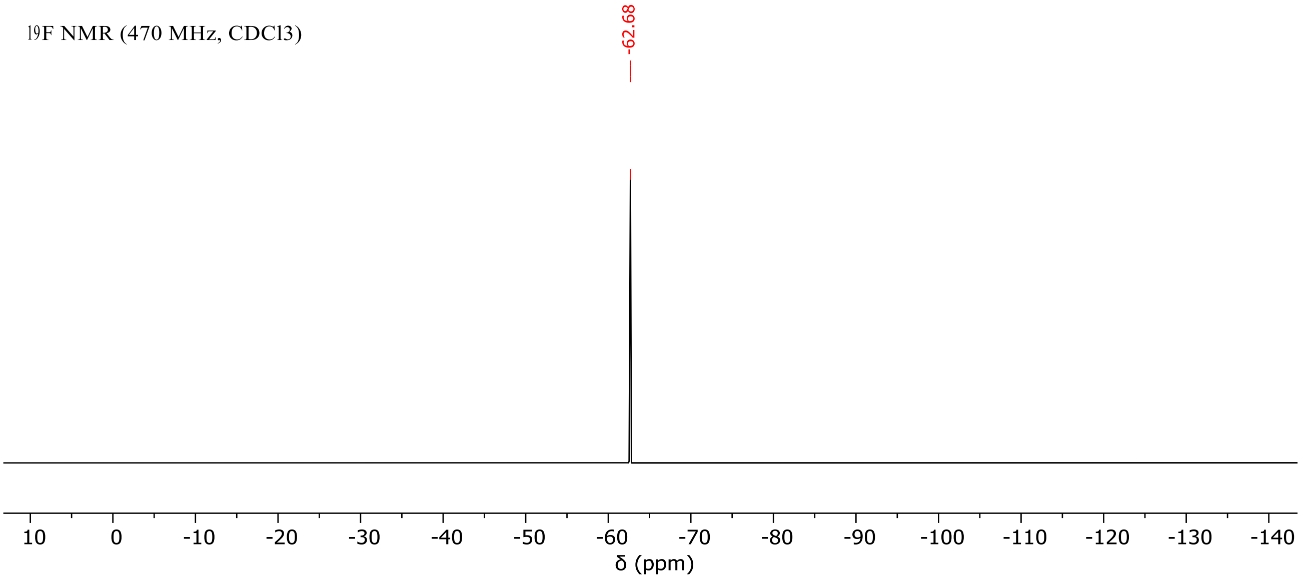

1H NMR (500 MHz, CDCl3)

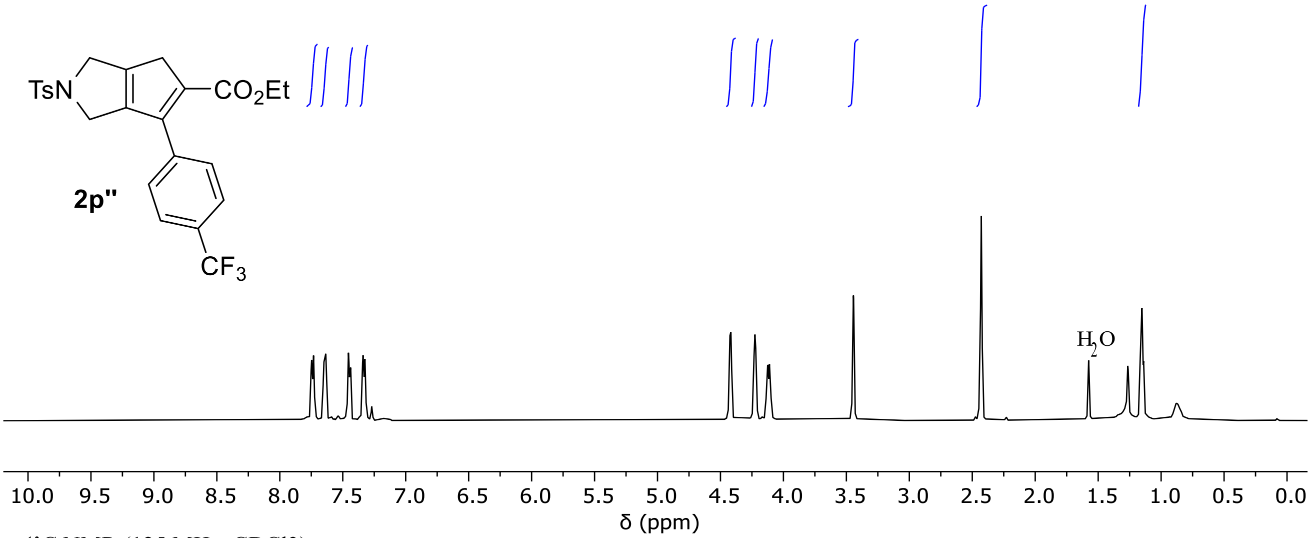

13C NMR (125 MHz, CDCl3)

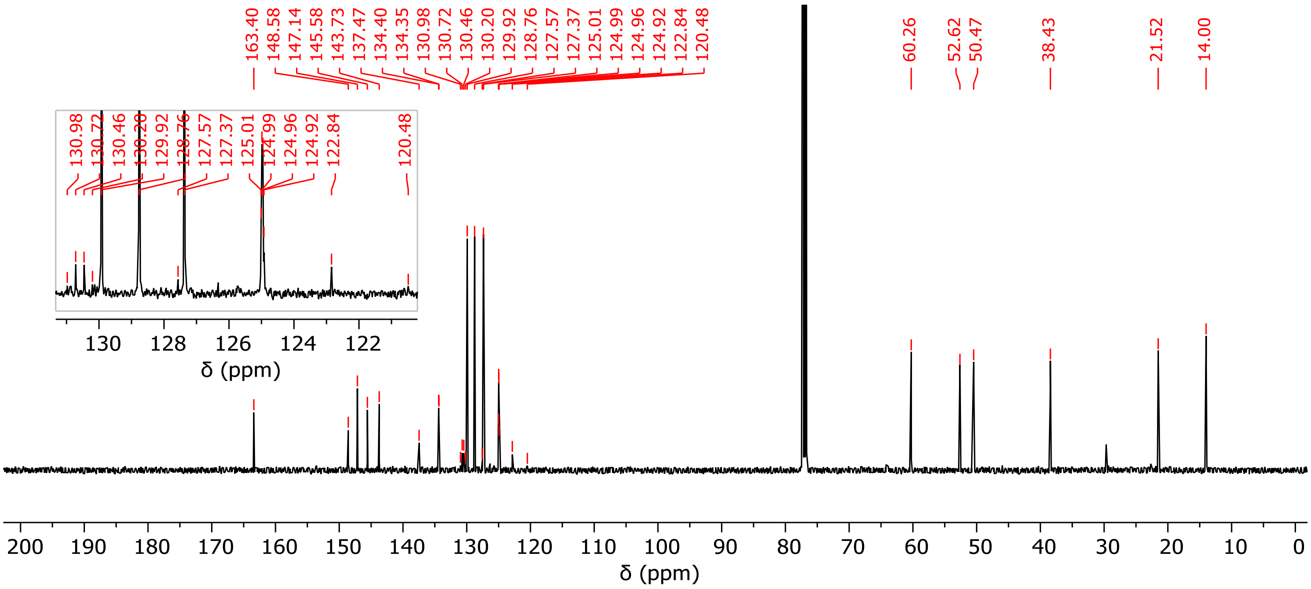

DEPT NMR (125 MHz, CDCl3)

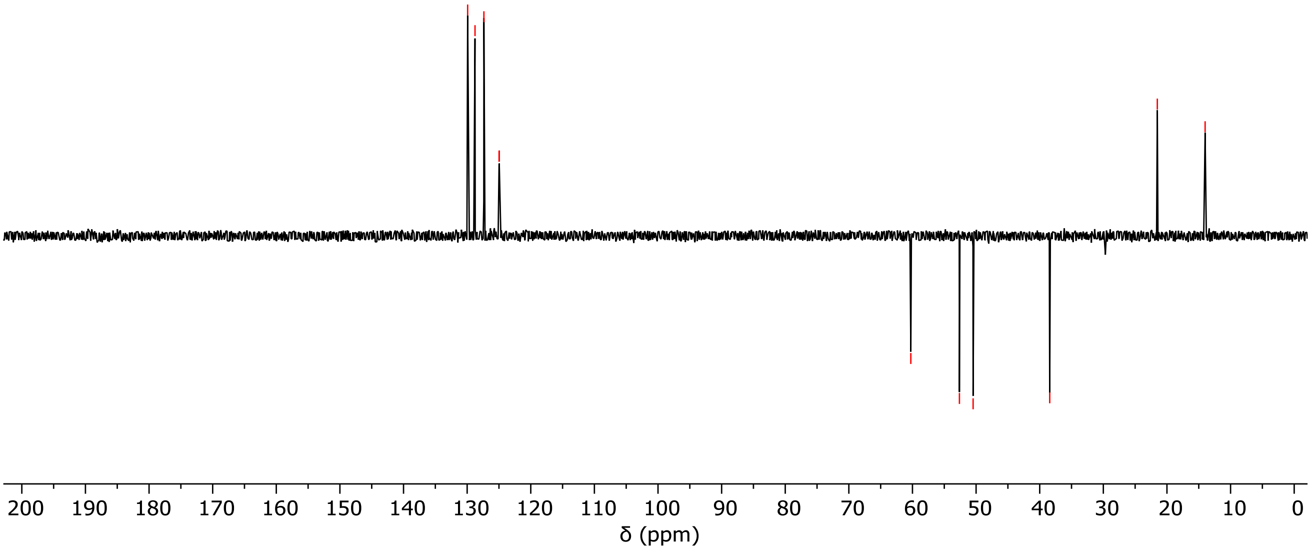

^{19}F NMR (470 MHz, $CDCl_3$)

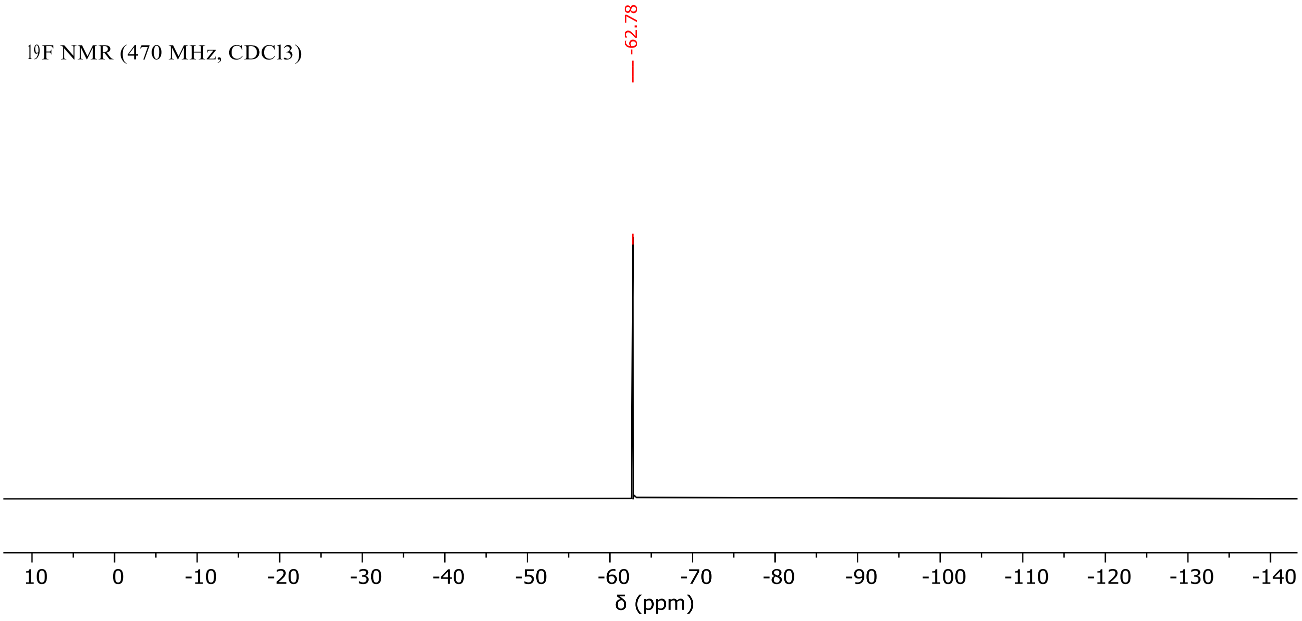

1H NMR (500 MHz, CDCl3)

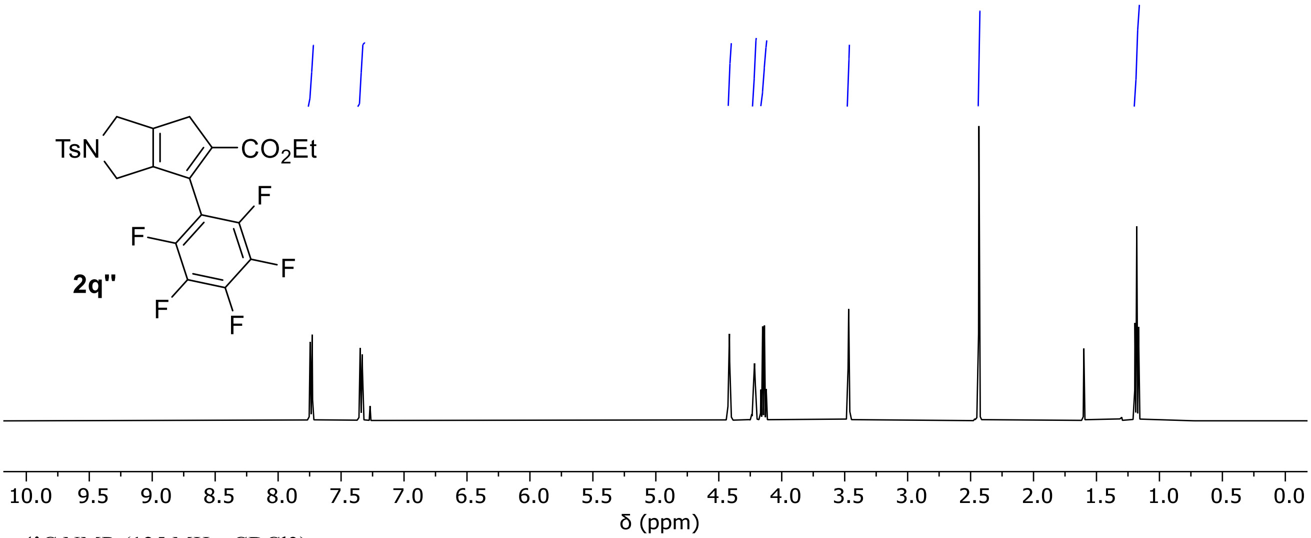

13C NMR (125 MHz, CDCl3)

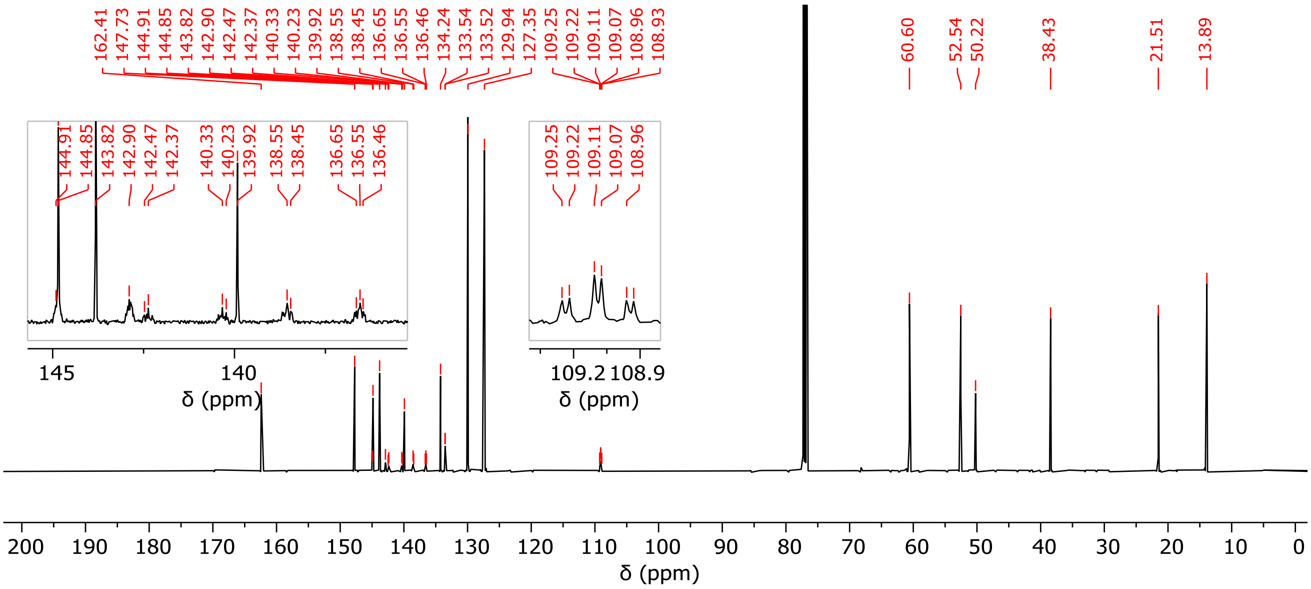

DEPT NMR (125 MHz, CDCl3)

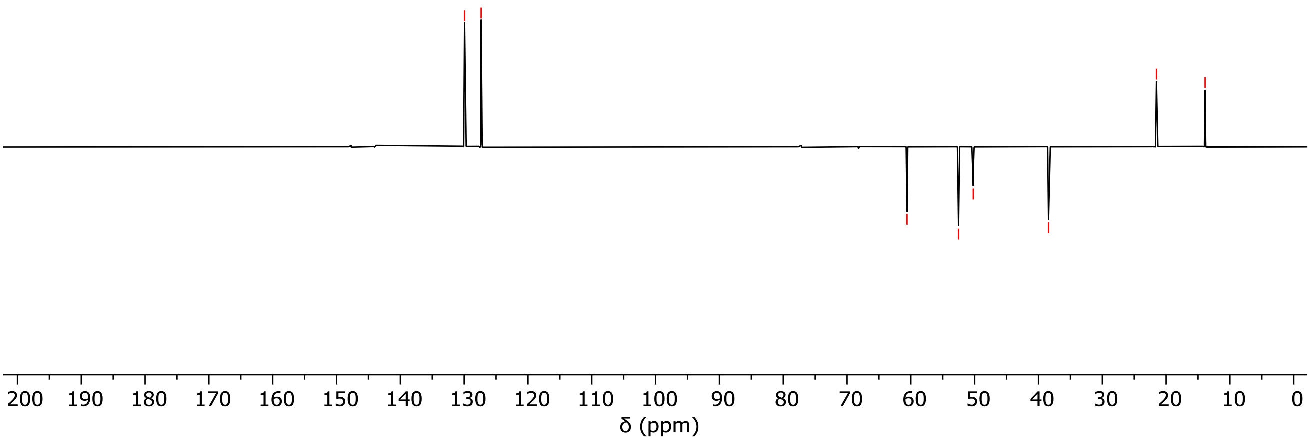

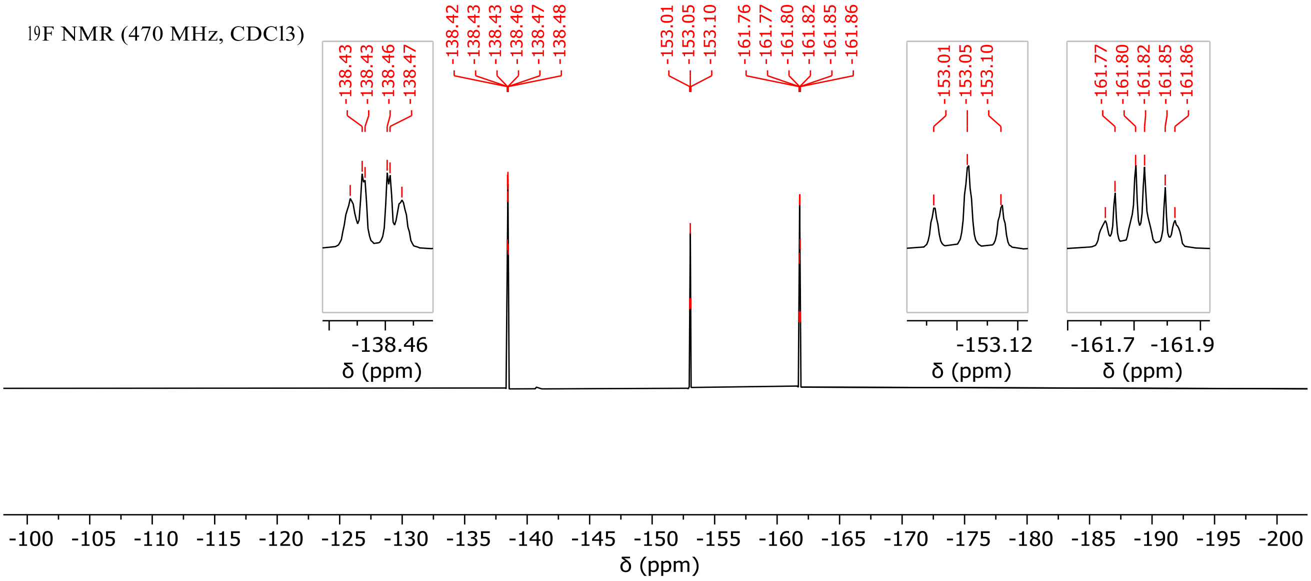
19F NMR (470 MHz, CDCl3)
-138.42
-138.43
-138.43
-138.46
-138.47
-138.48
-153.01
-153.05
-153.10
-161.76
-161.77
-161.80
-161.82
-161.85
-161.86
-138.46
δ (ppm)
-153.12
δ (ppm)
-161.7 -161.9
δ (ppm)
-100 -105 -110 -115 -120 -125 -130 -135 -140 -145 -150 -155 -160 -165 -170 -175 -180 -185 -190 -195 -200
δ (ppm)

1H NMR (300 MHz, CDCl3)

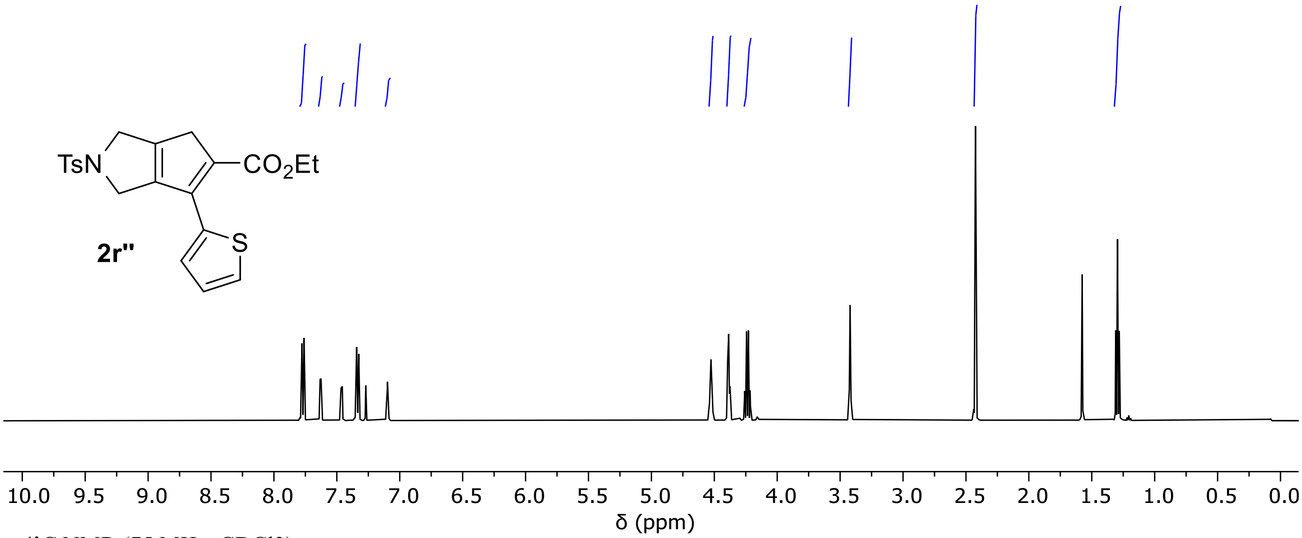

13C NMR (75 MHz, CDCl3)

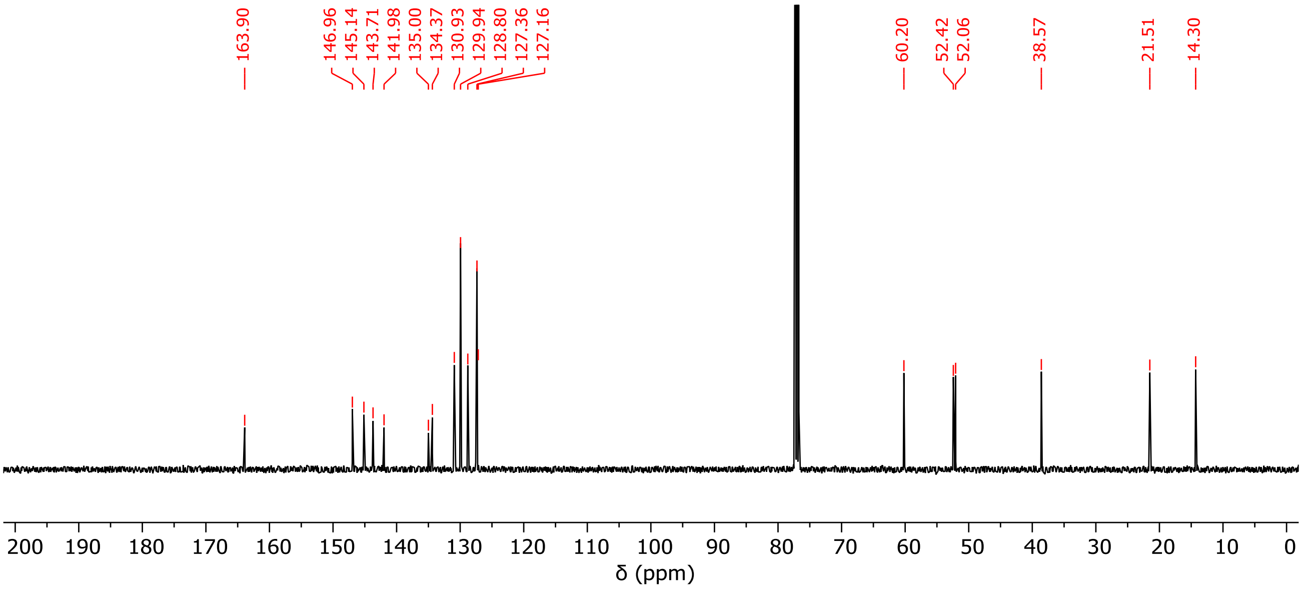

DEPT NMR (75 MHz, CDCl3)

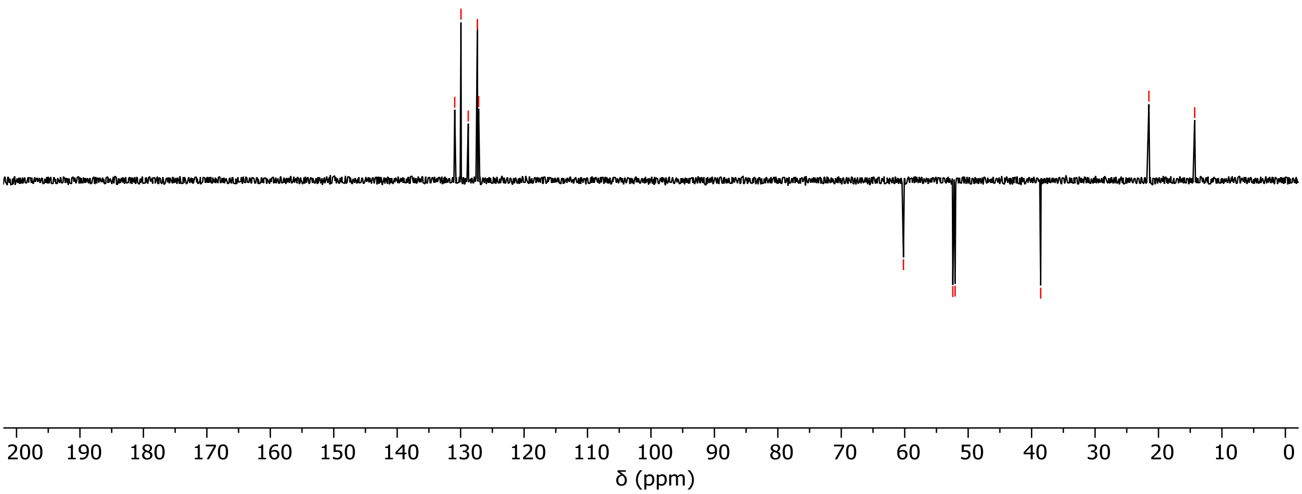

lH NMR (500 MHz, CDCl3)

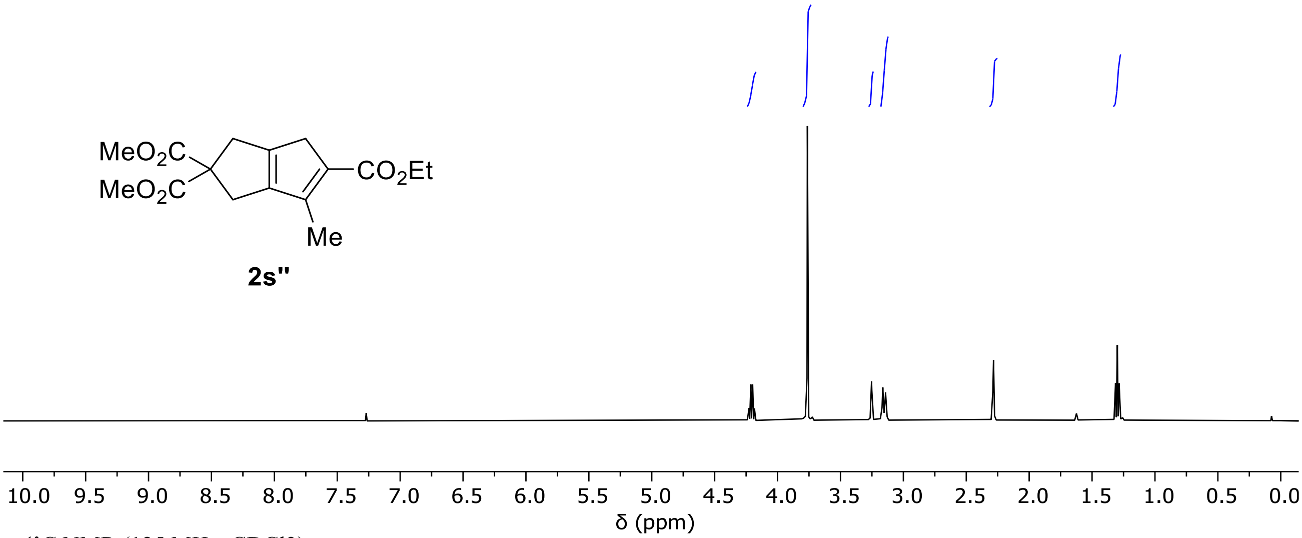

l3C NMR (125 MHz, CDCl3)

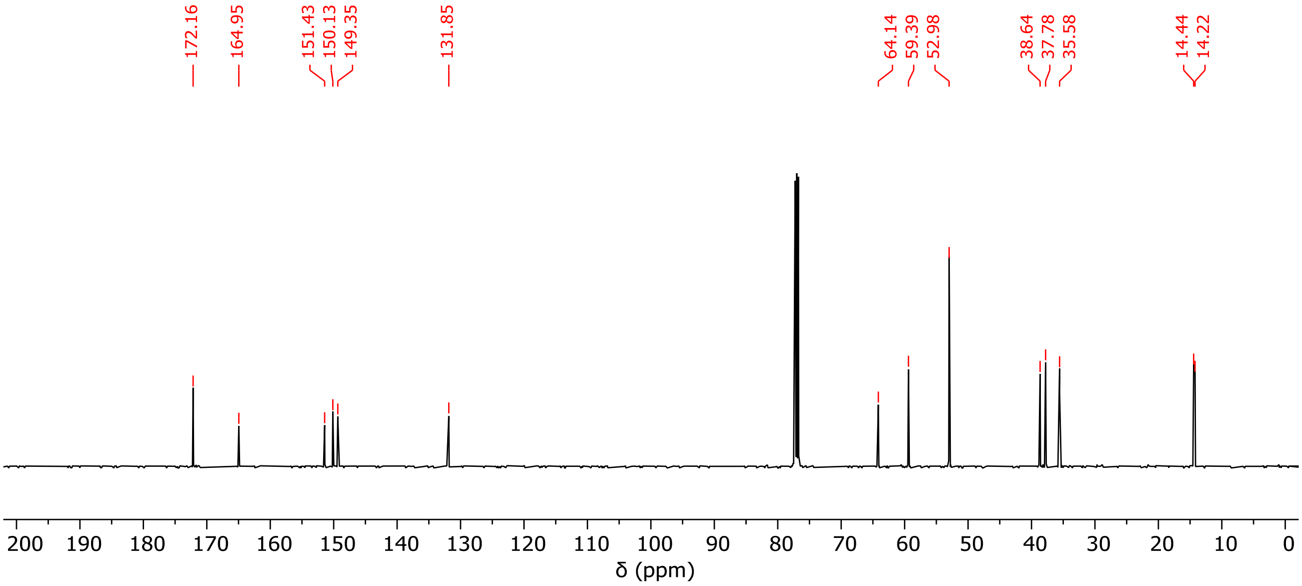

DEPT NMR (125 MHz, CDCl3)

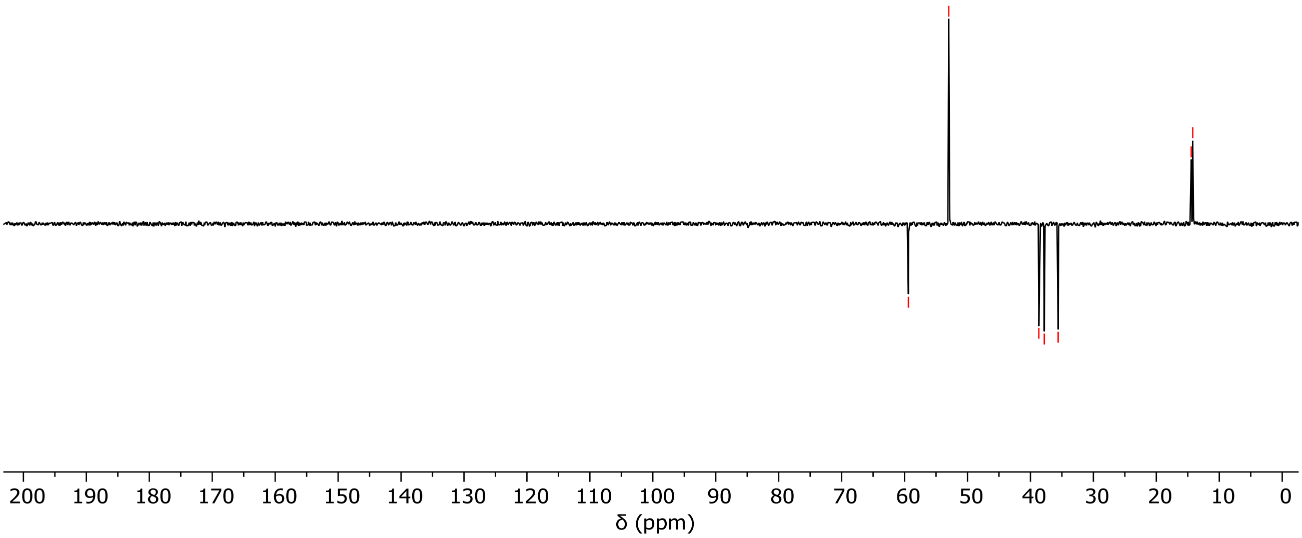

1H NMR (300 MHz, CDCl3)

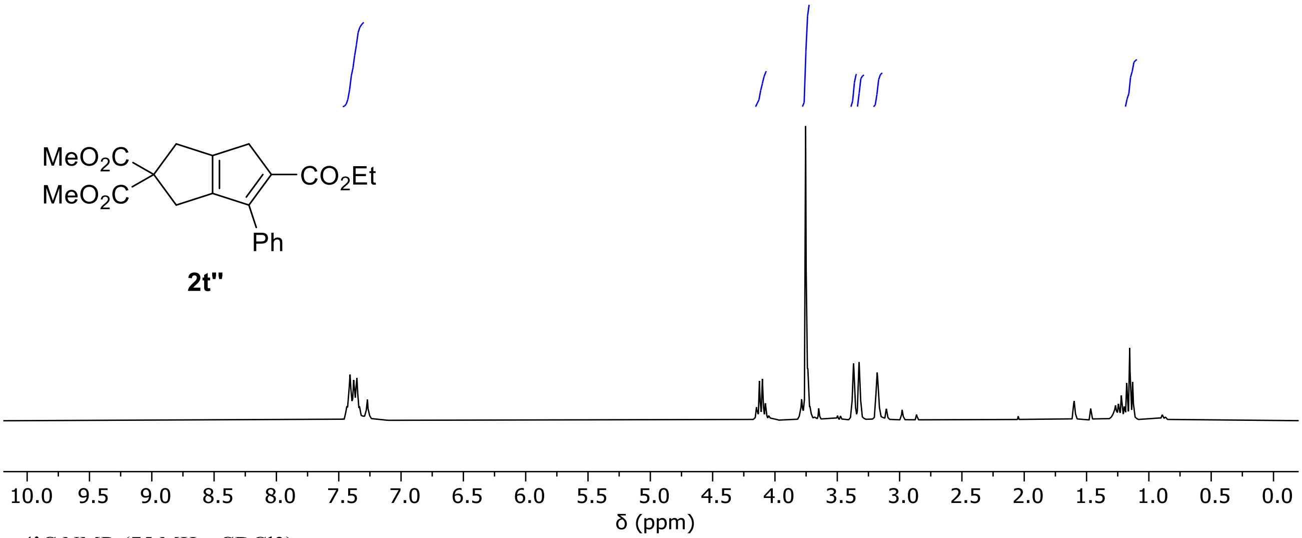

2t''

13C NMR (75 MHz, CDCl3)

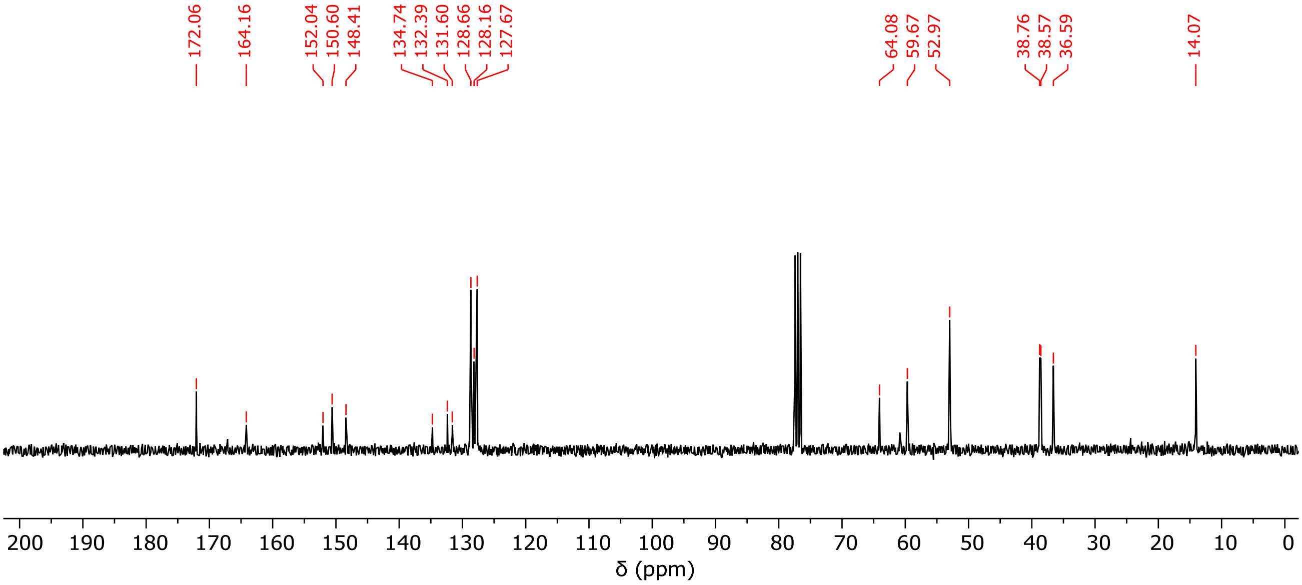

DEPT NMR (75 MHz, CDCl3)

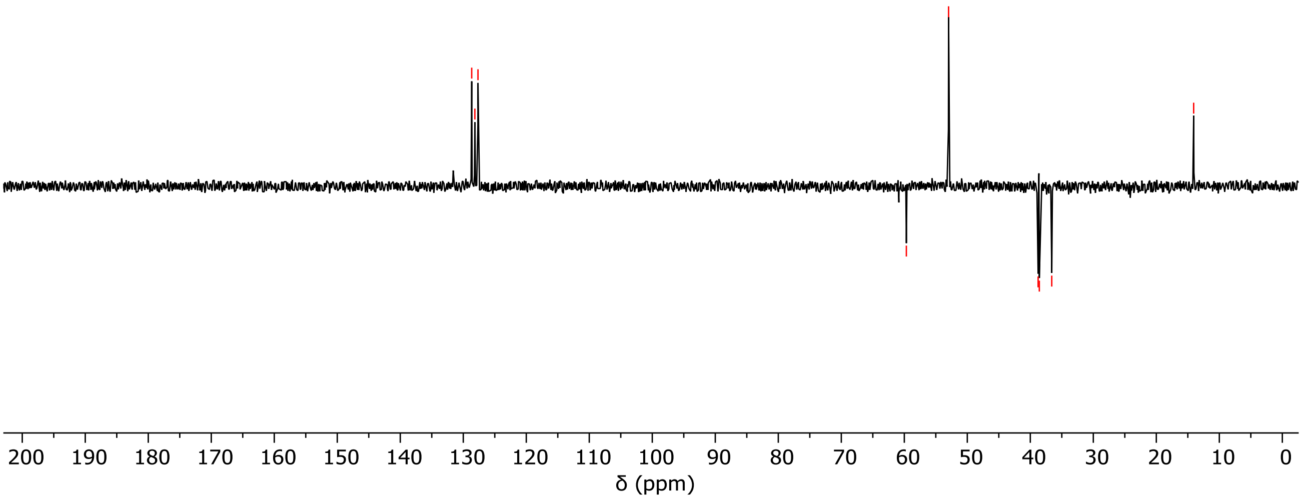

1H NMR (300 MHz, CDCl3)

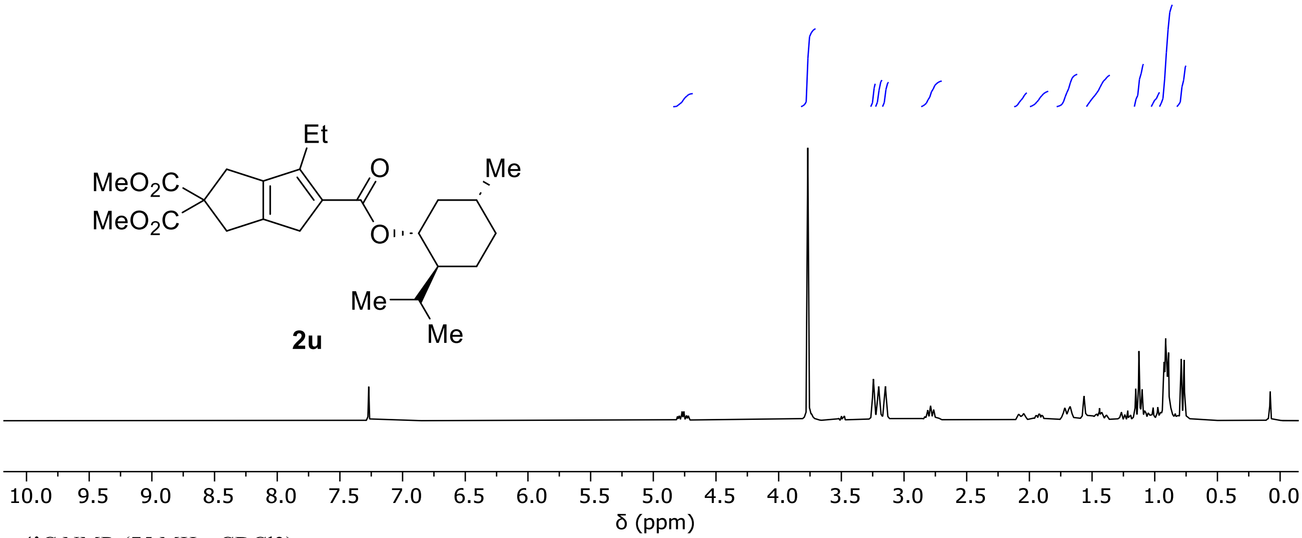

13C NMR (75 MHz, CDCl3)

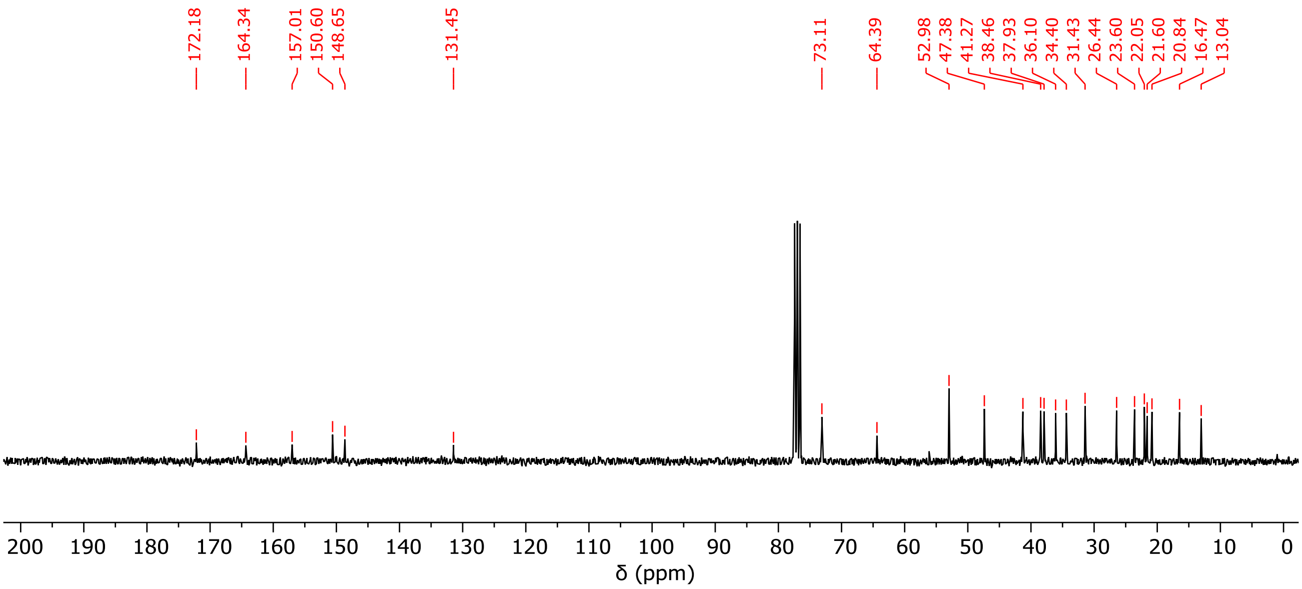

DEPT NMR (75 MHz, CDCl3)

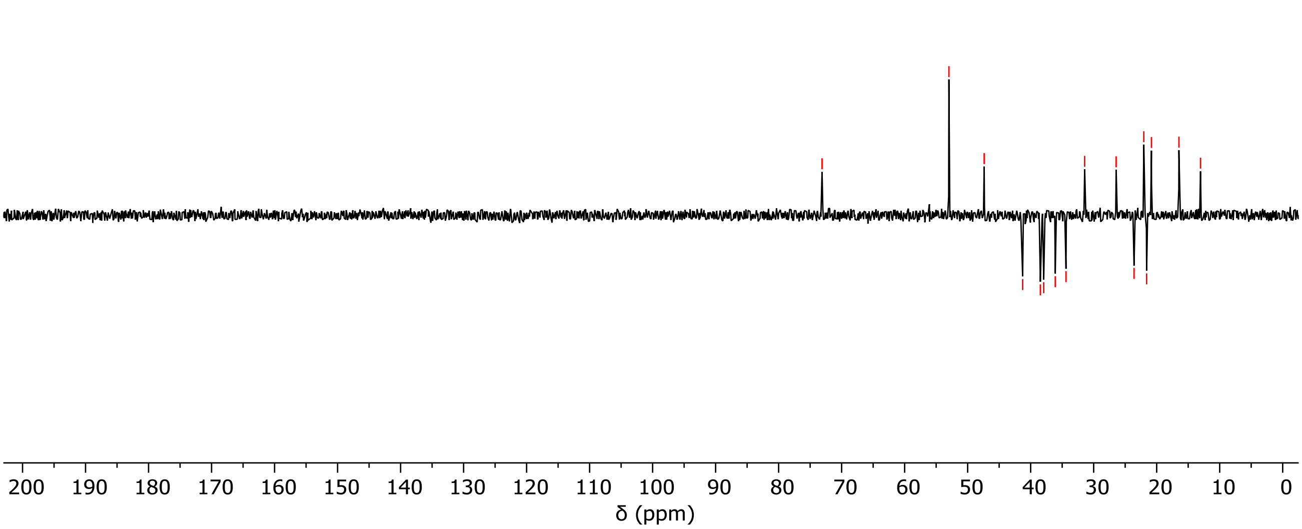

1H NMR (500 MHz, CDCl3)

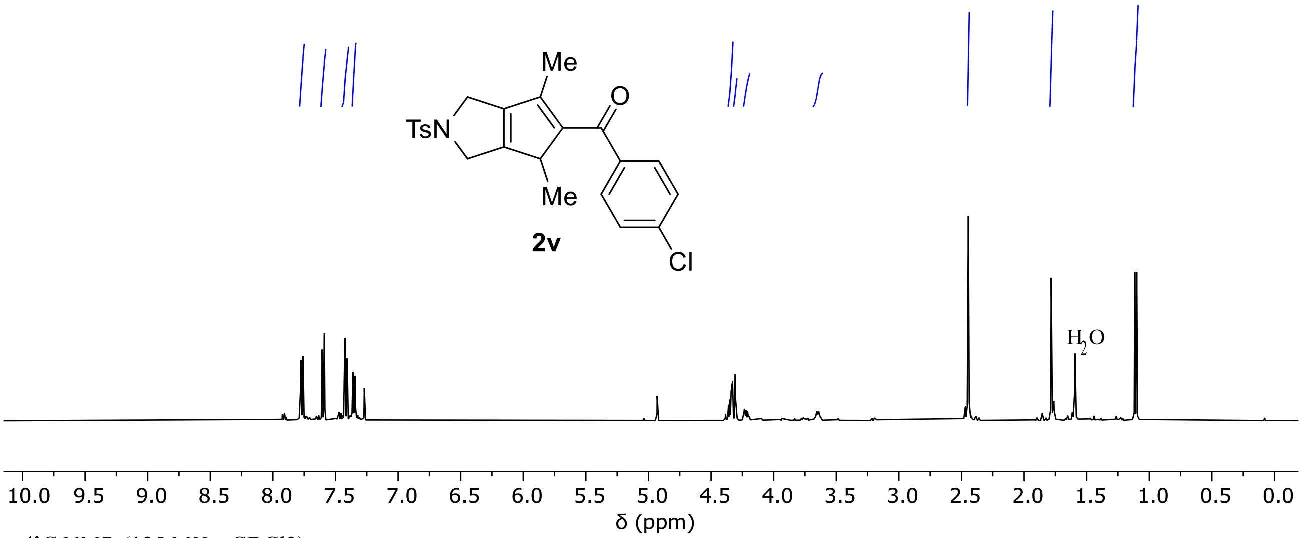

13C NMR (125 MHz, CDCl3)

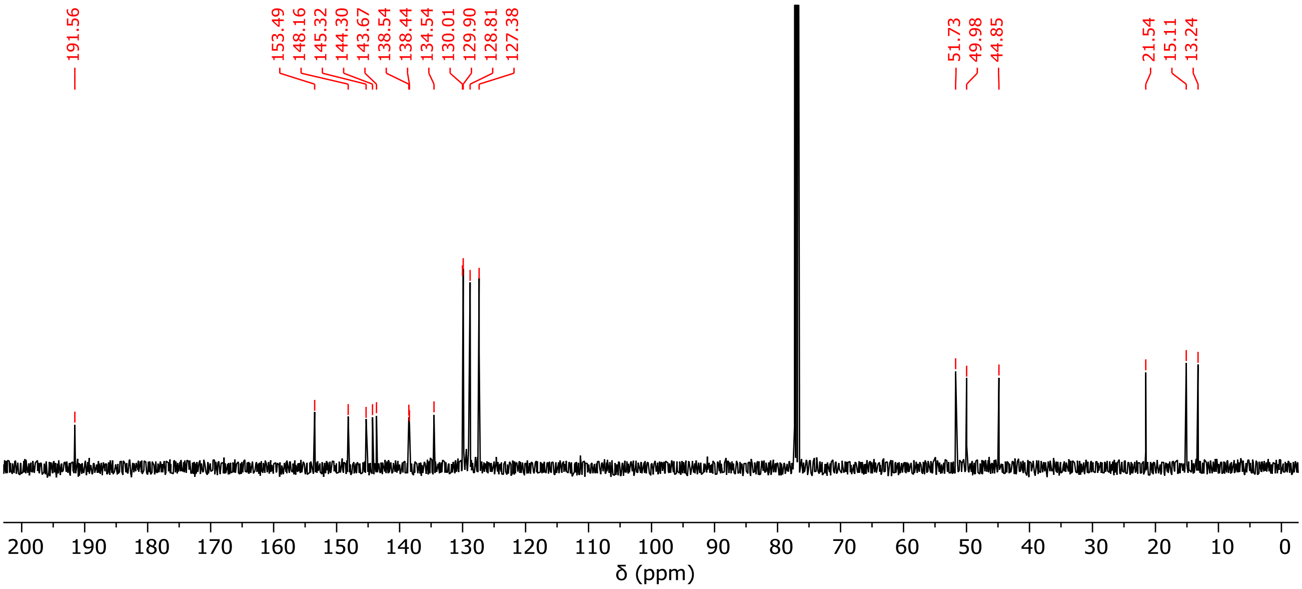

DEPT NMR (125 MHz, CDCl3)

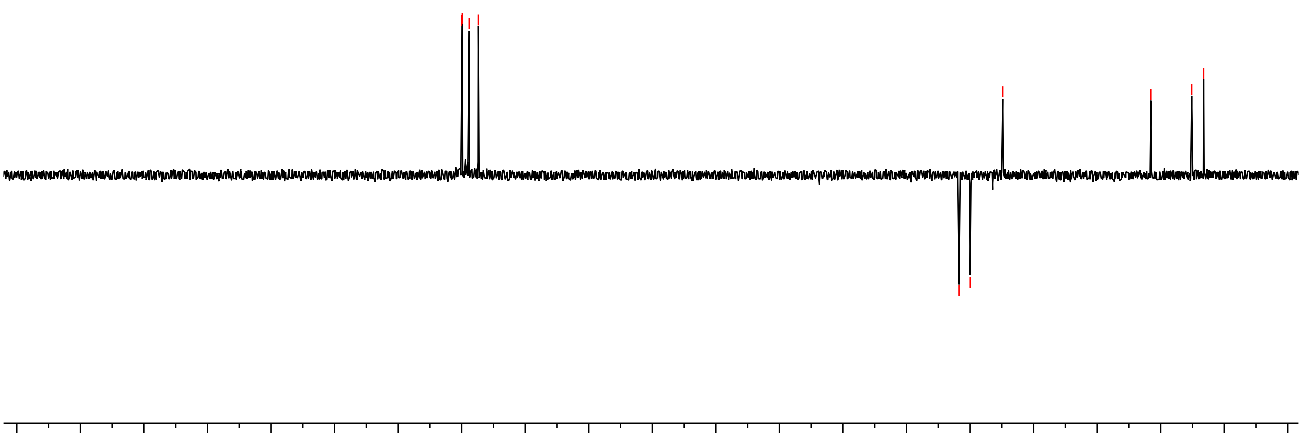

1H NMR (500 MHz, CDCl3)

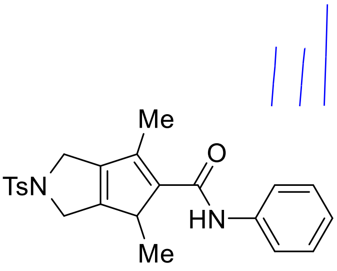

2w

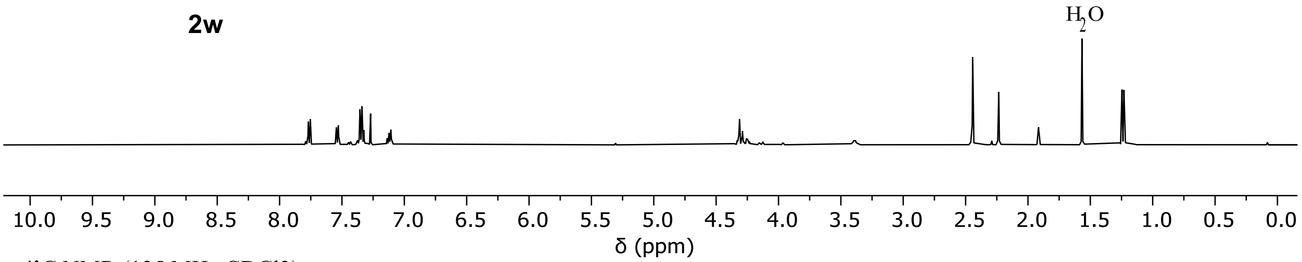

13C NMR (125 MHz,CDCl3)

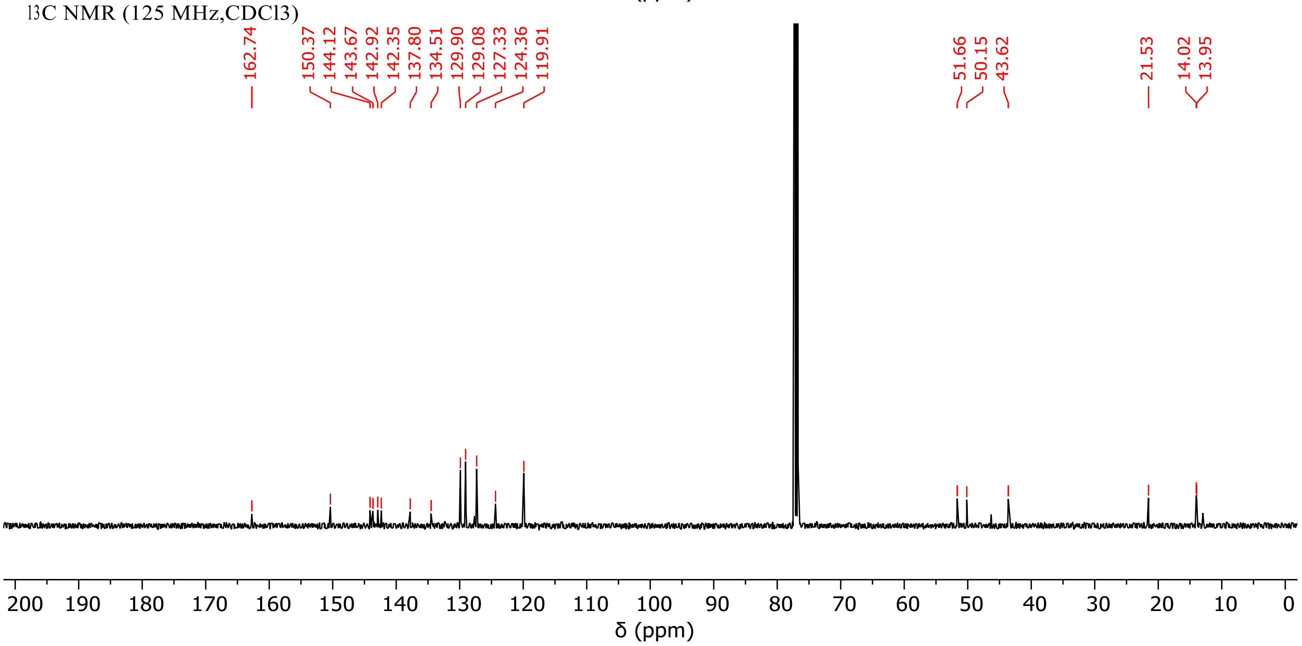

DEPT NMR (125 MHz,CDCl3)

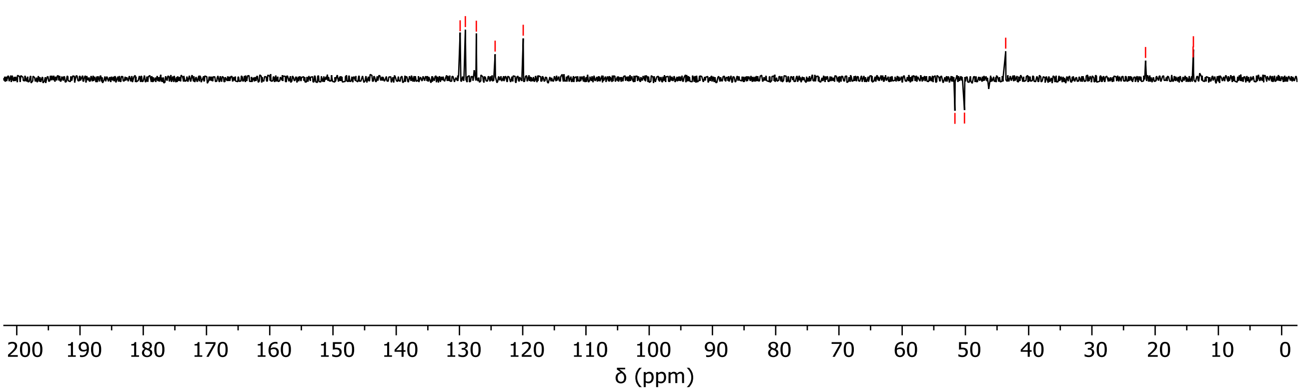

1H NMR (300 MHz, CDCl3)

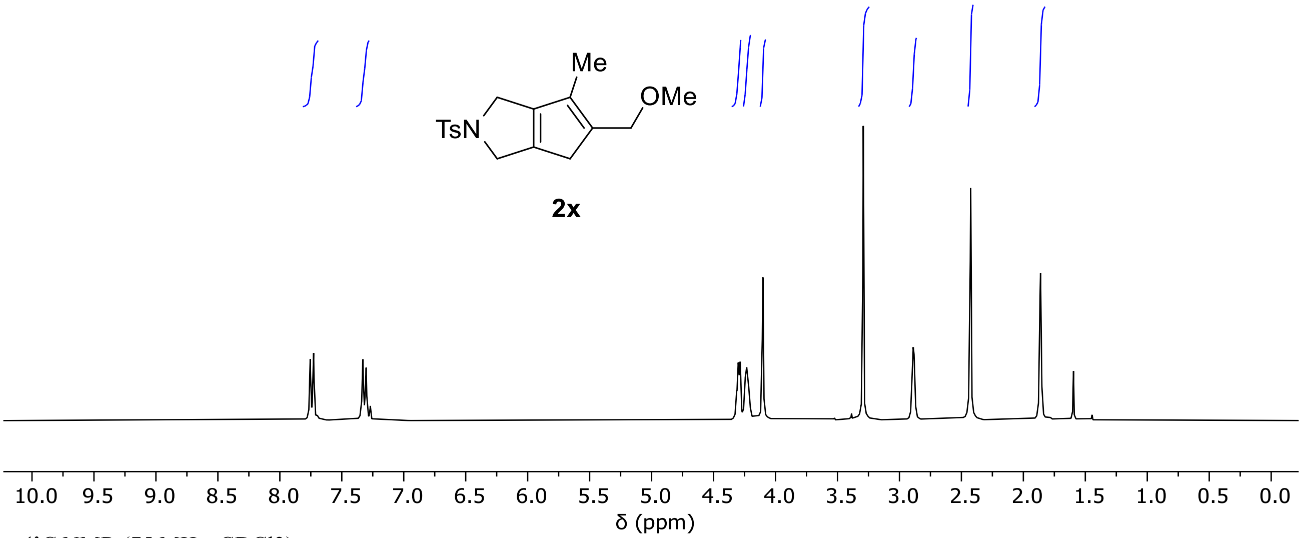

13C NMR (75 MHz, CDCl3)

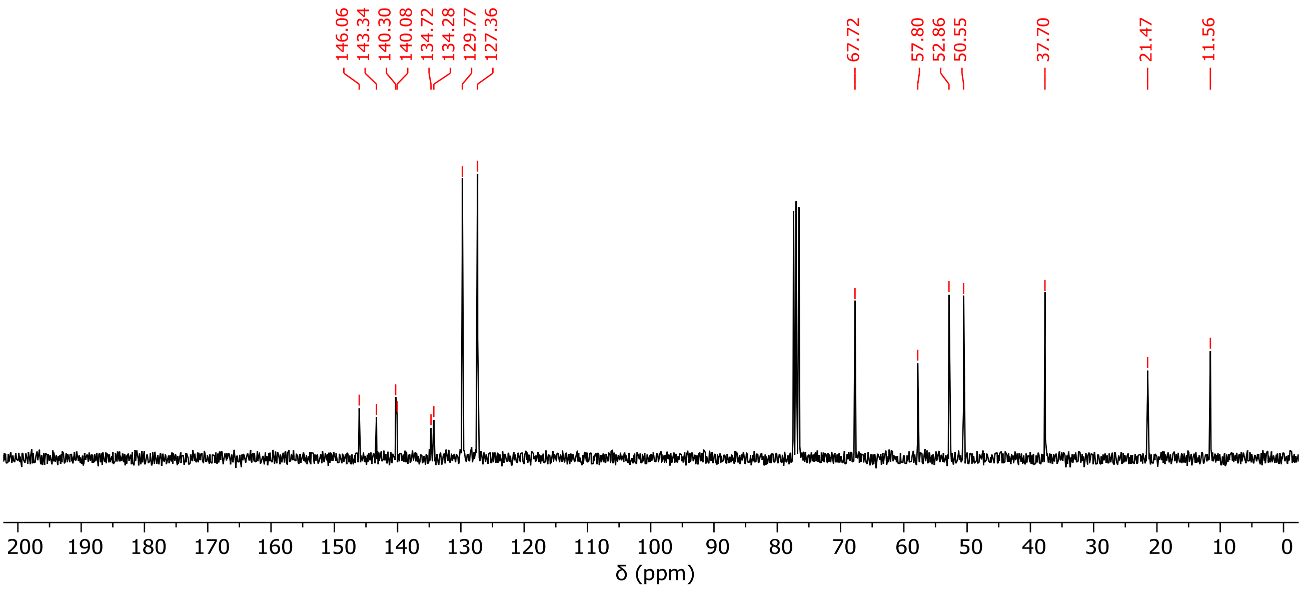

DEPT NMR (75 MHz, CDCl3)

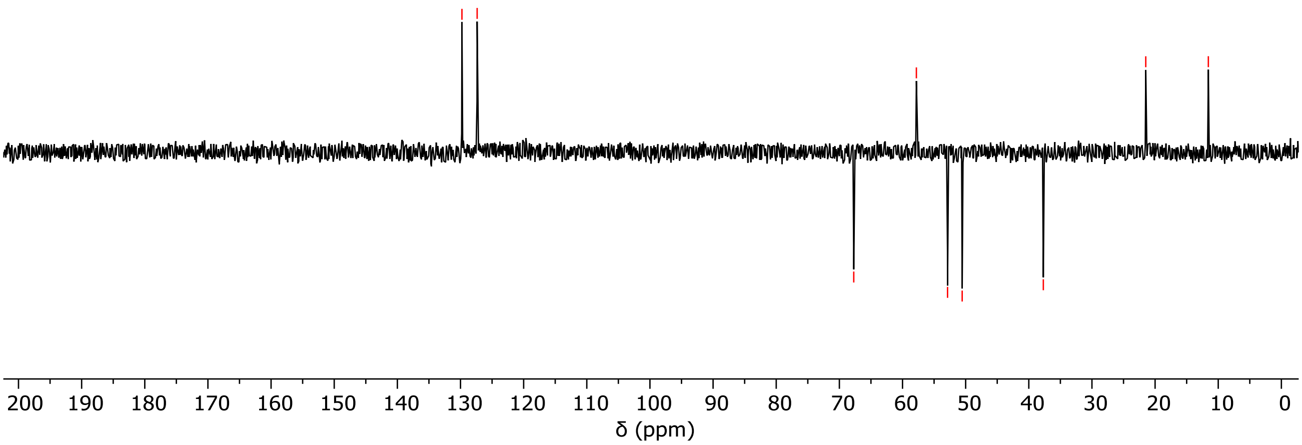

1H NMR (300 MHz, CDCl3)

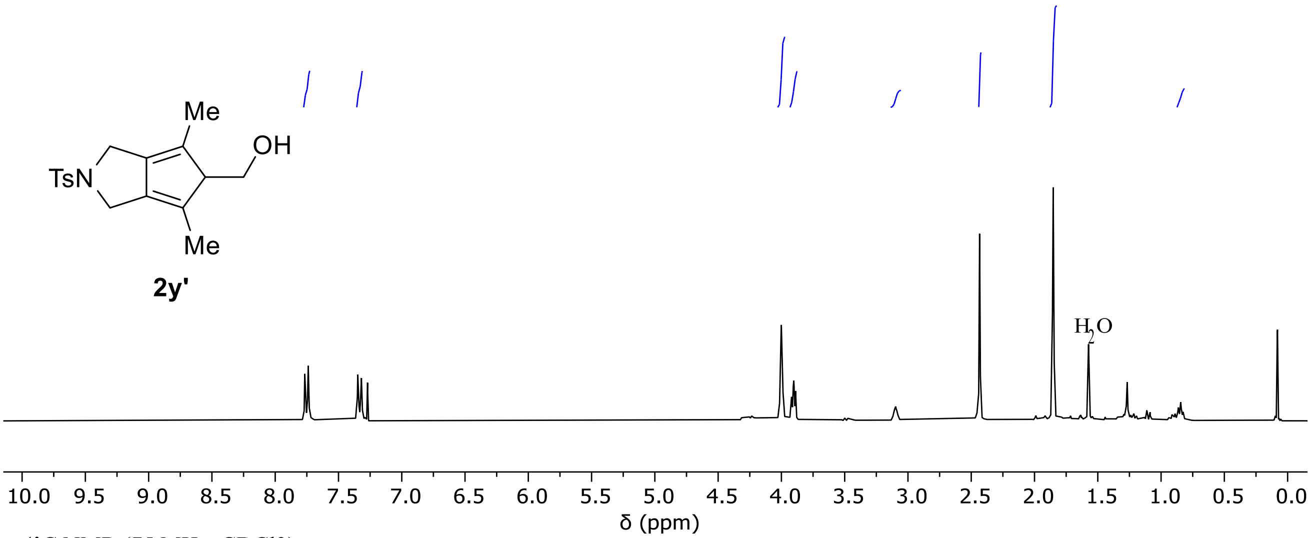

13C NMR (75 MHz, CDCl3)

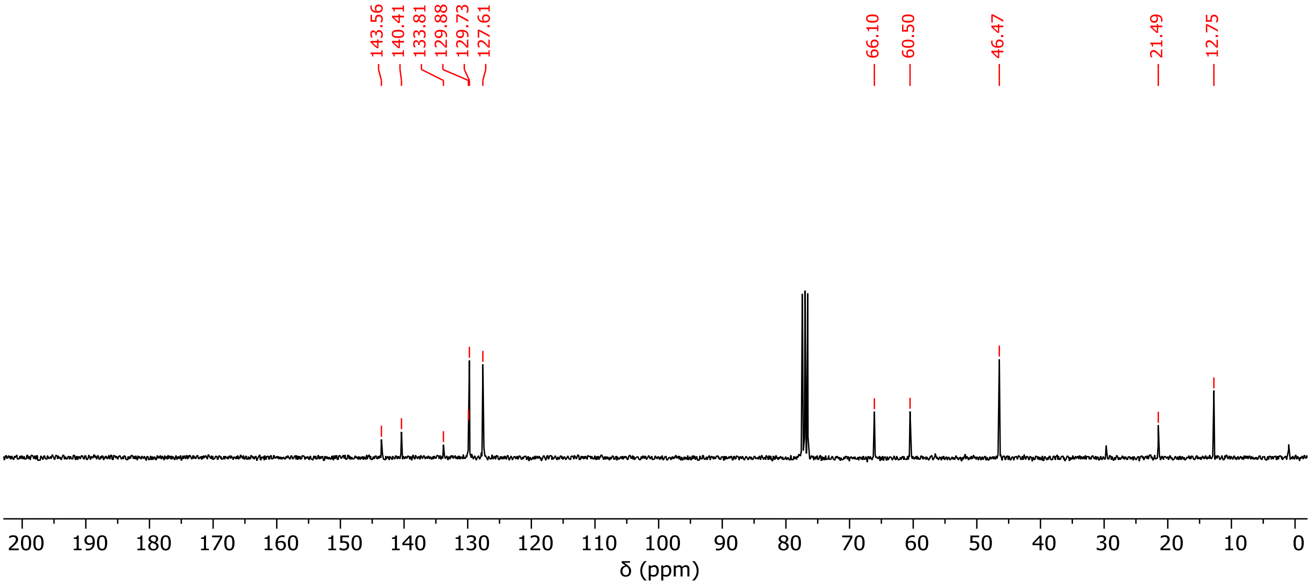

DEPT NMR (75 MHz, CDCl3)

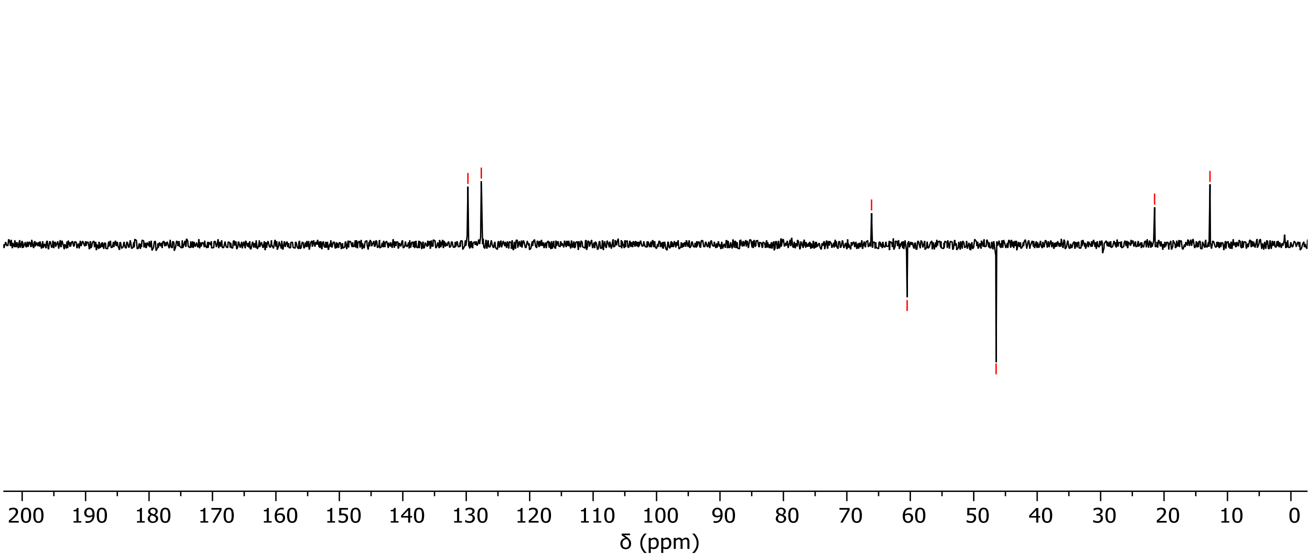

1H NMR (500 MHz, CDCl3)

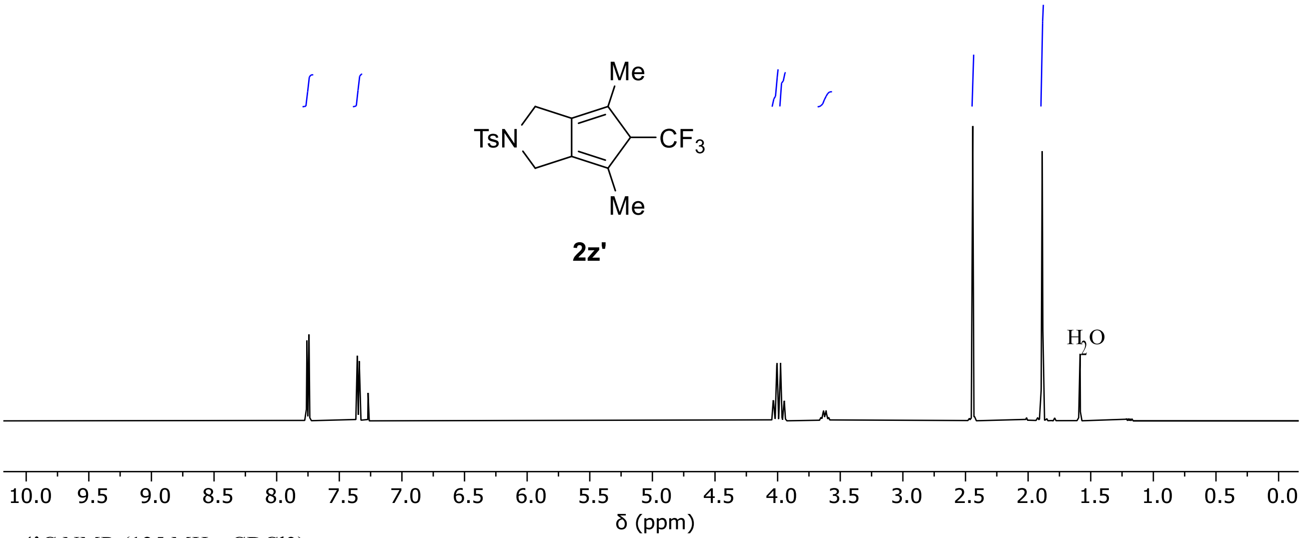

13C NMR (125 MHz, CDCl3)

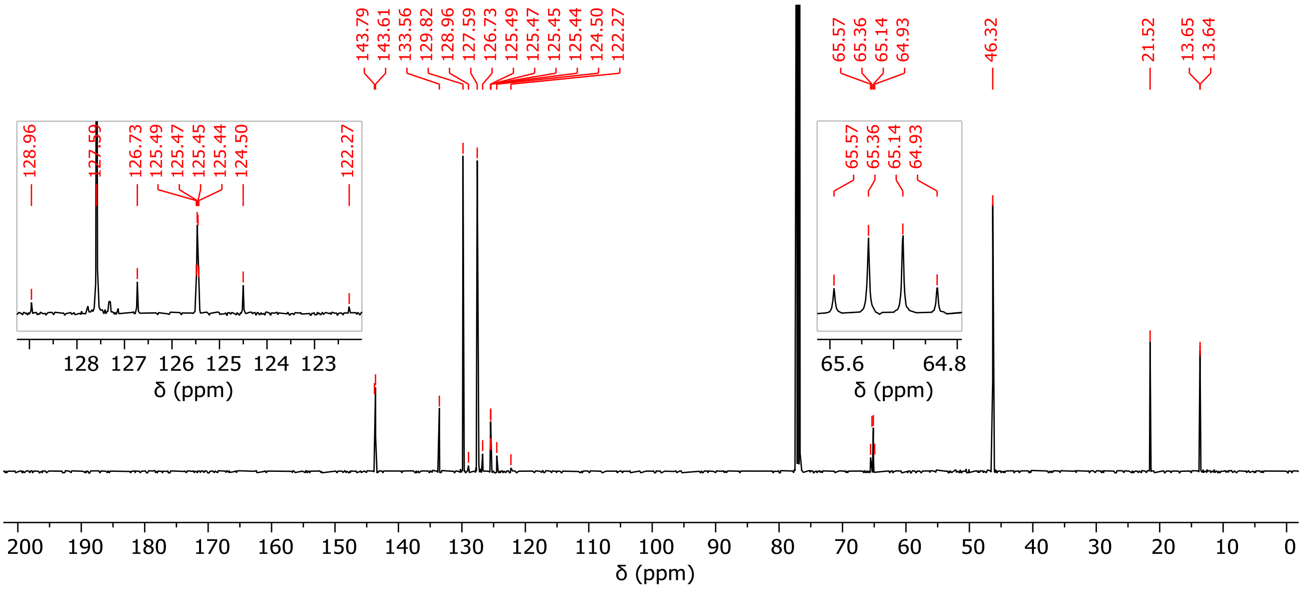

DEPT NMR (125 MHz, CDCl3)

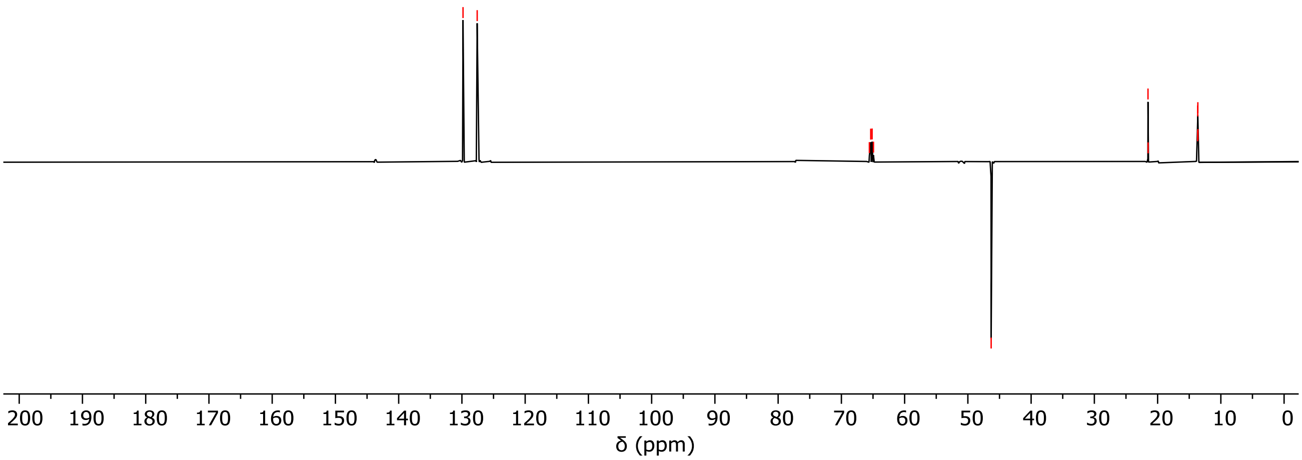

^{19}F NMR (470 MHz, $CDCl_3$)

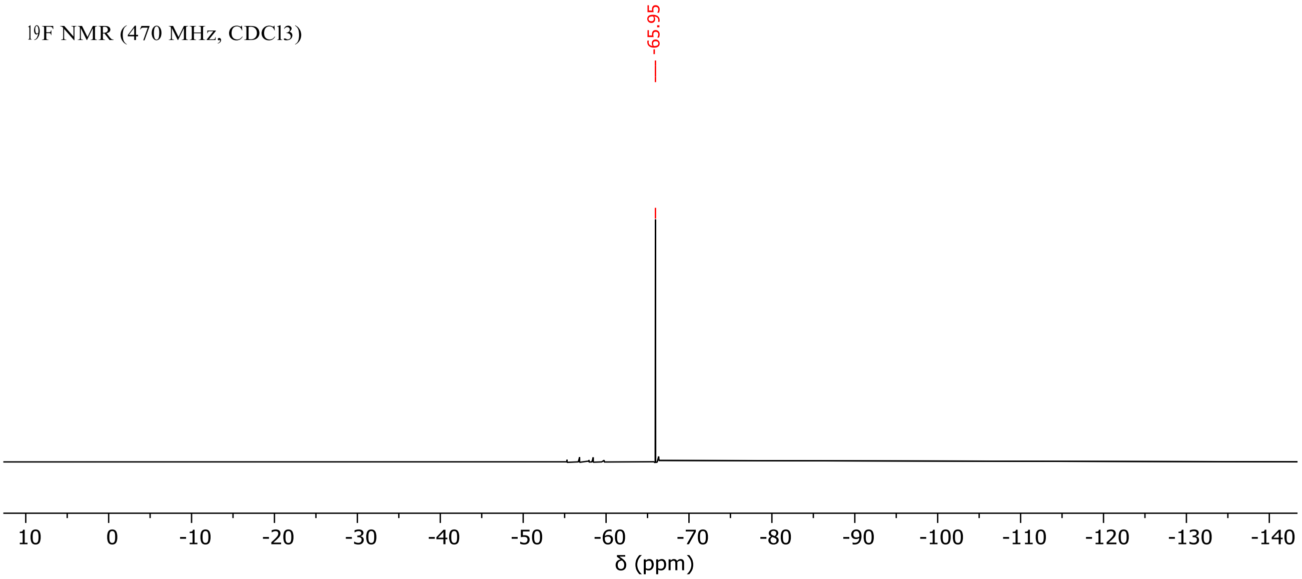

1H NMR (500 MHz, CDCl3)

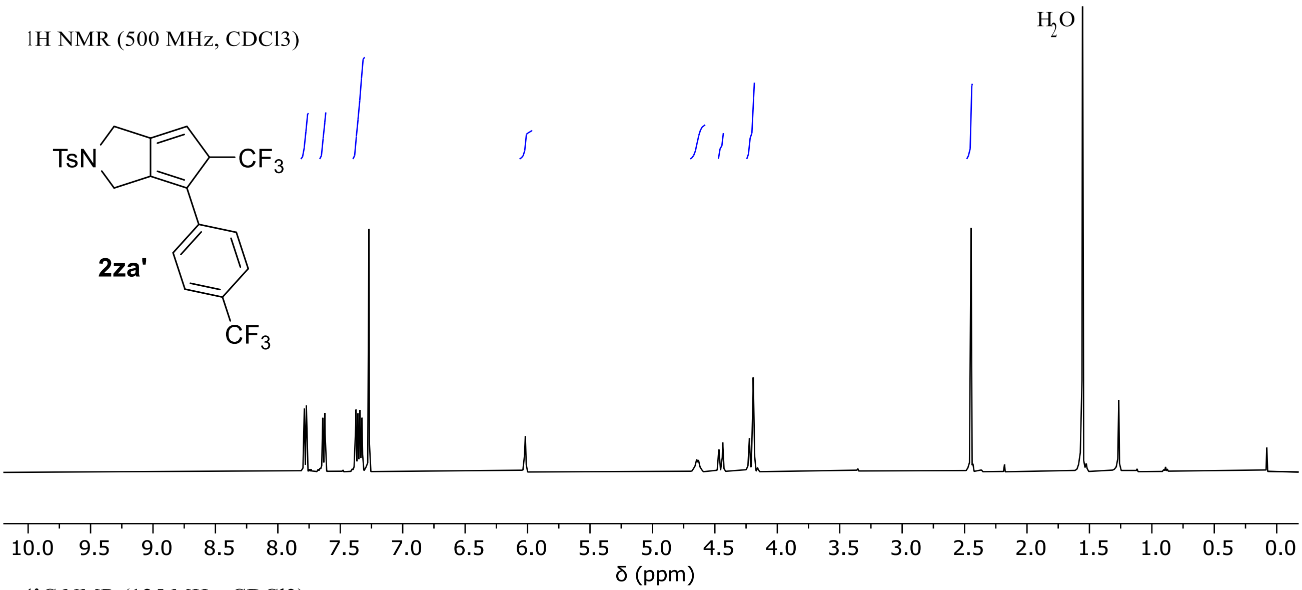

13C NMR (125 MHz, CDCl3)

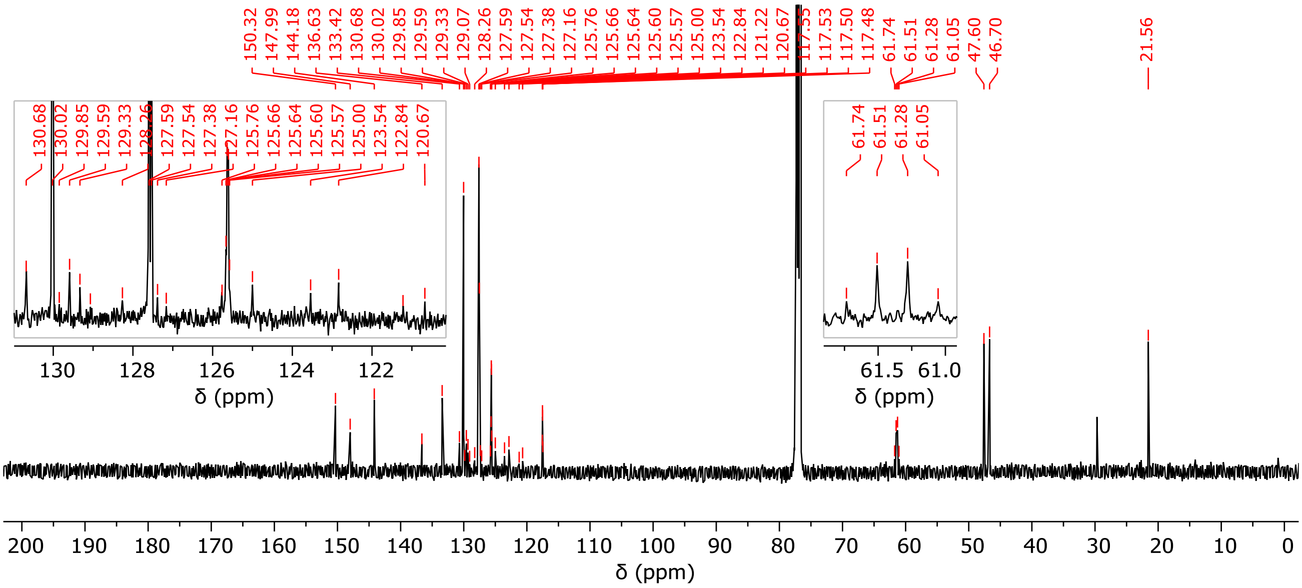

DEPT NMR (125 MHz, CDCl3)

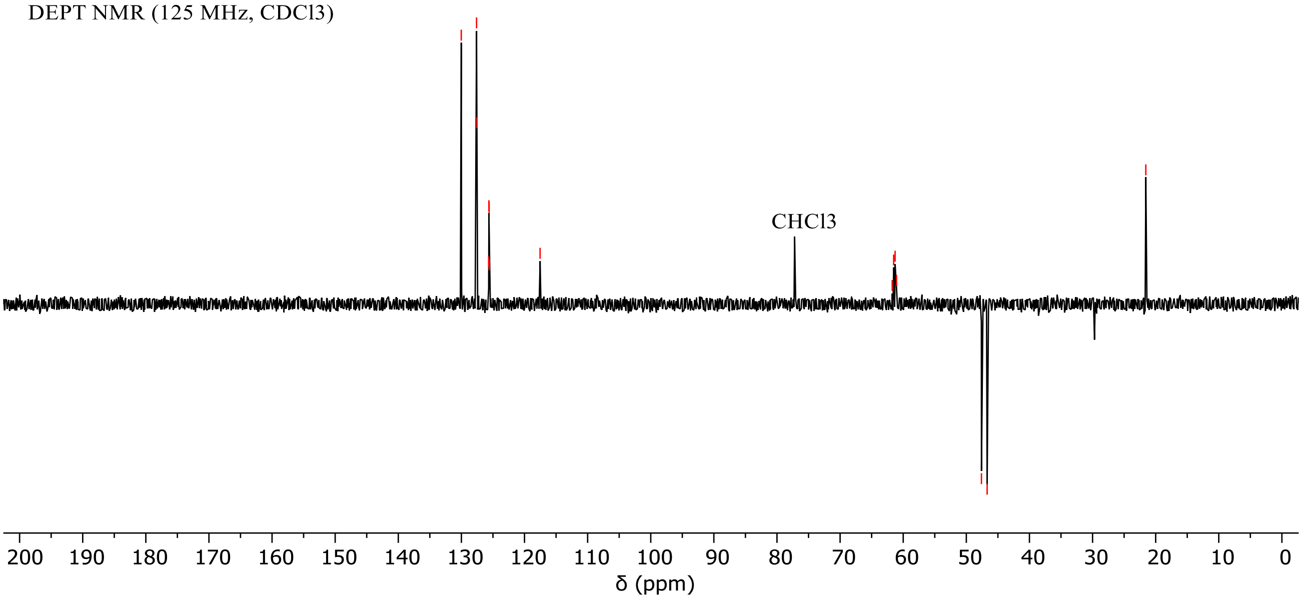

19F NMR (470 MHz, CDCl3)

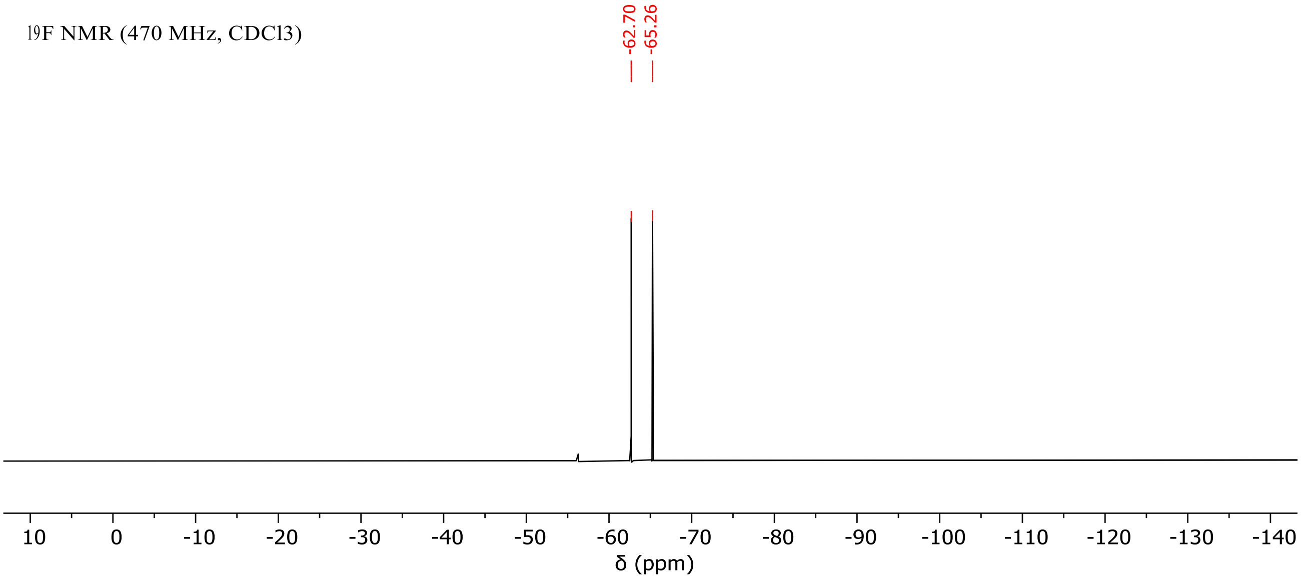

1H NMR (300 MHz, CDCl3)

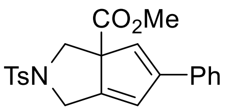

2zb'''

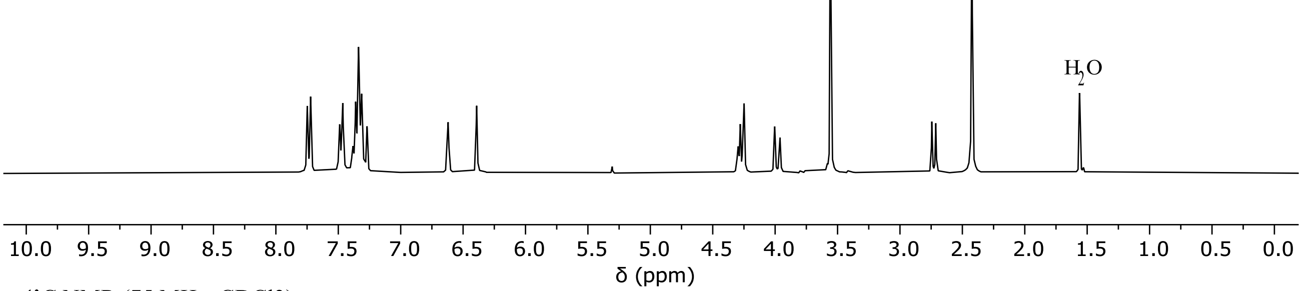

13C NMR (75 MHz, CDCl3)

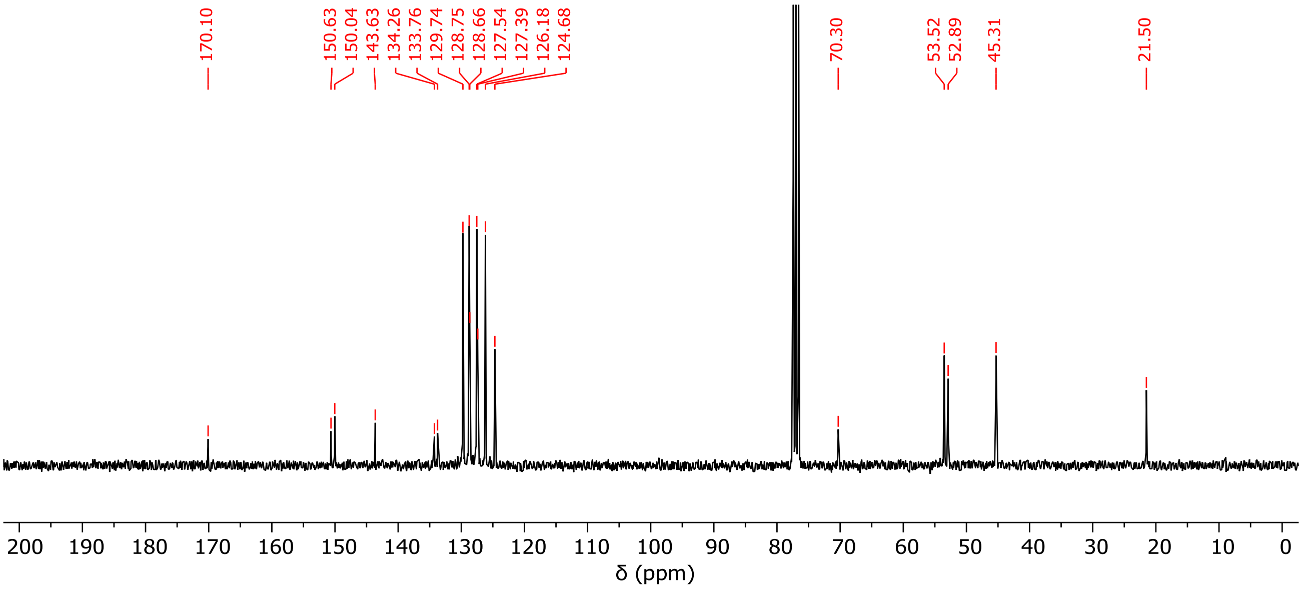

DEPT NMR (75 MHz, CDCl3)

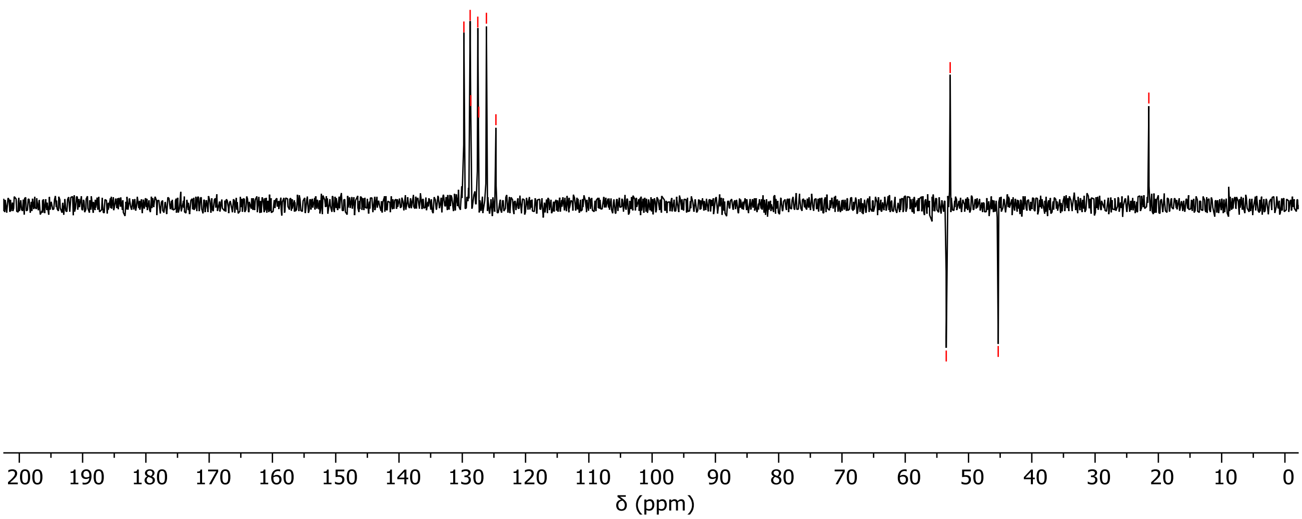

1H NMR (500 MHz, CDCl3)

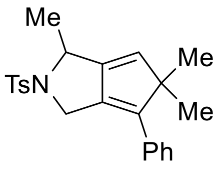

2zc'

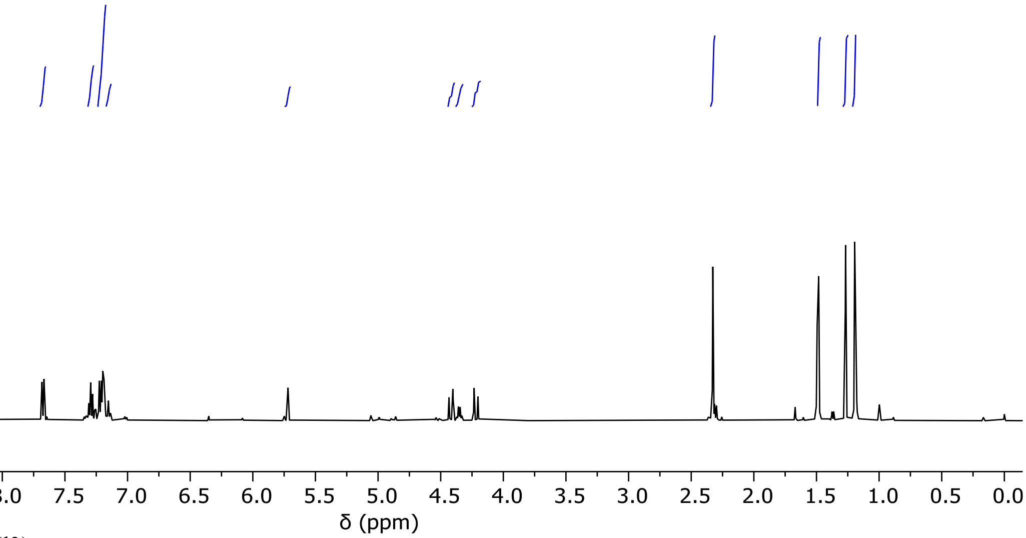

13C NMR (125 MHz, CDCl3)

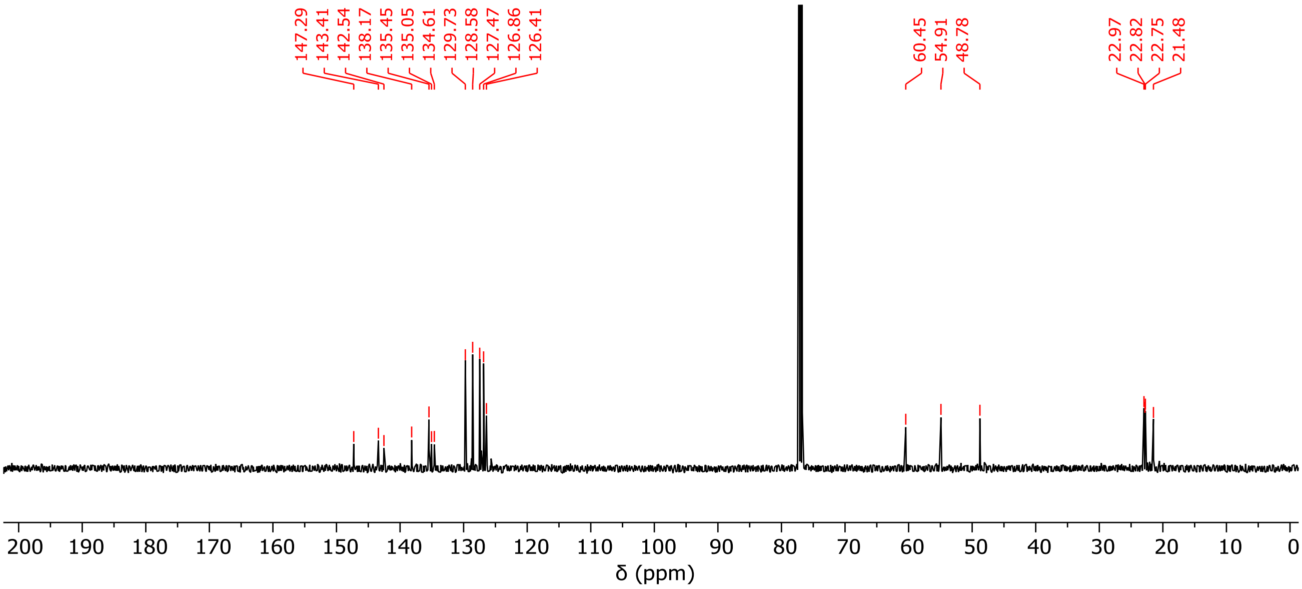

DEPT NMR (125 MHz, CDCl3)

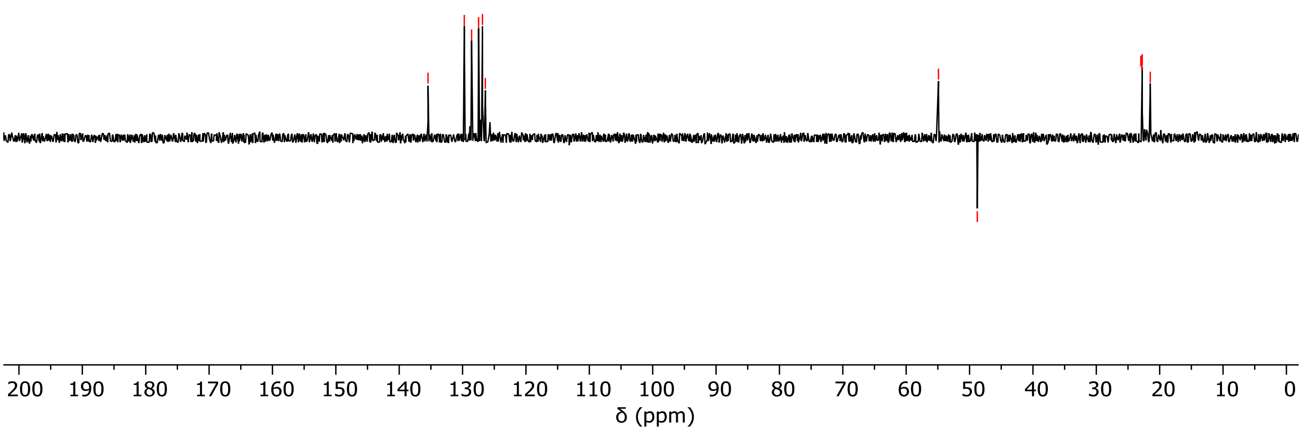

1H NMR (500 MHz, CDCl3)

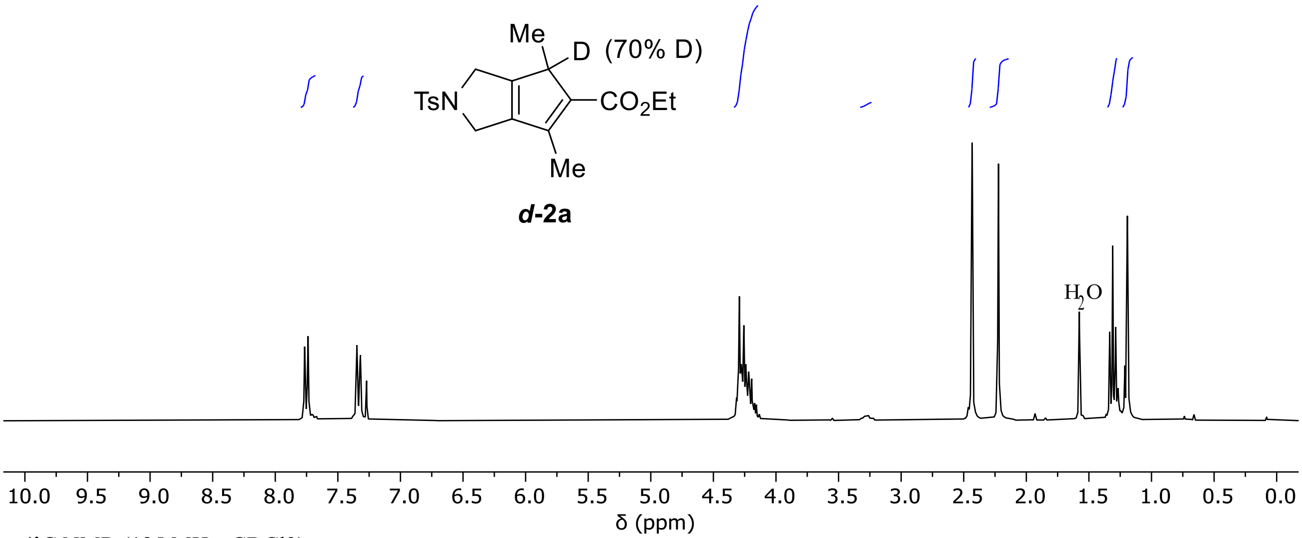

13C NMR (125 MHz, CDCl3)

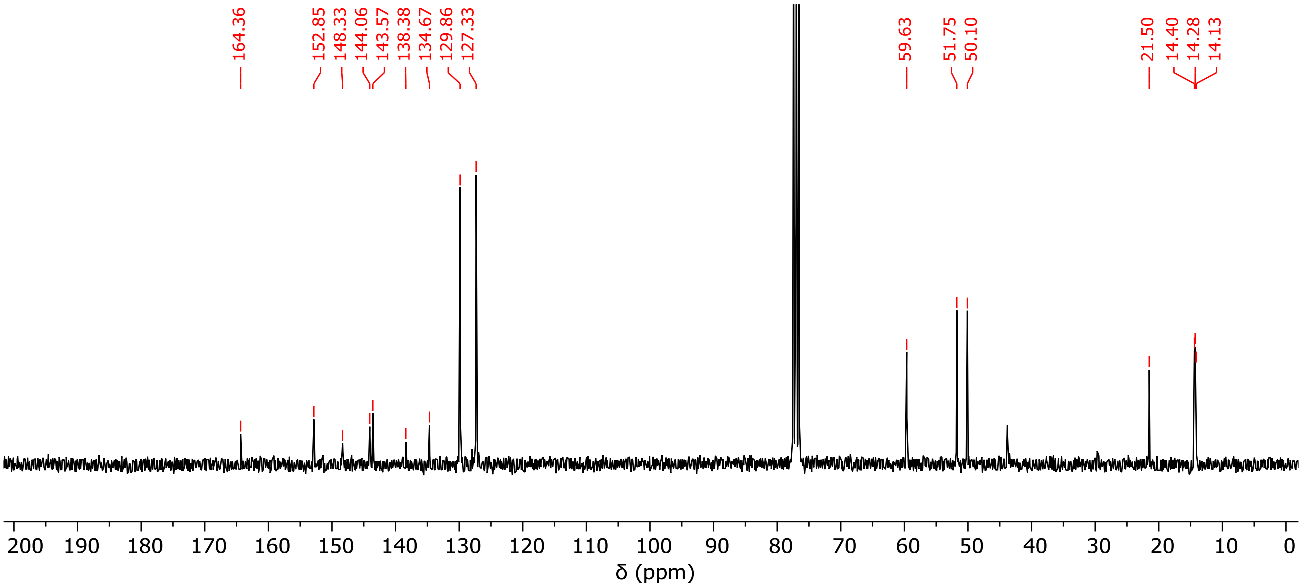

DEPT NMR (125 MHz, CDCl3)

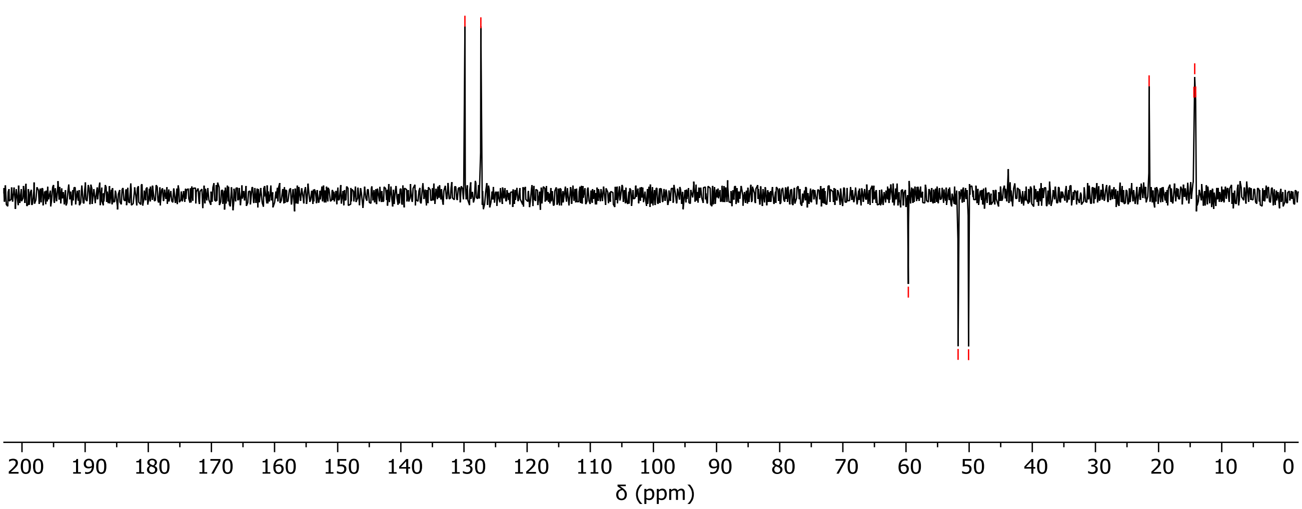

1H NMR (300 MHz, CDCl3)

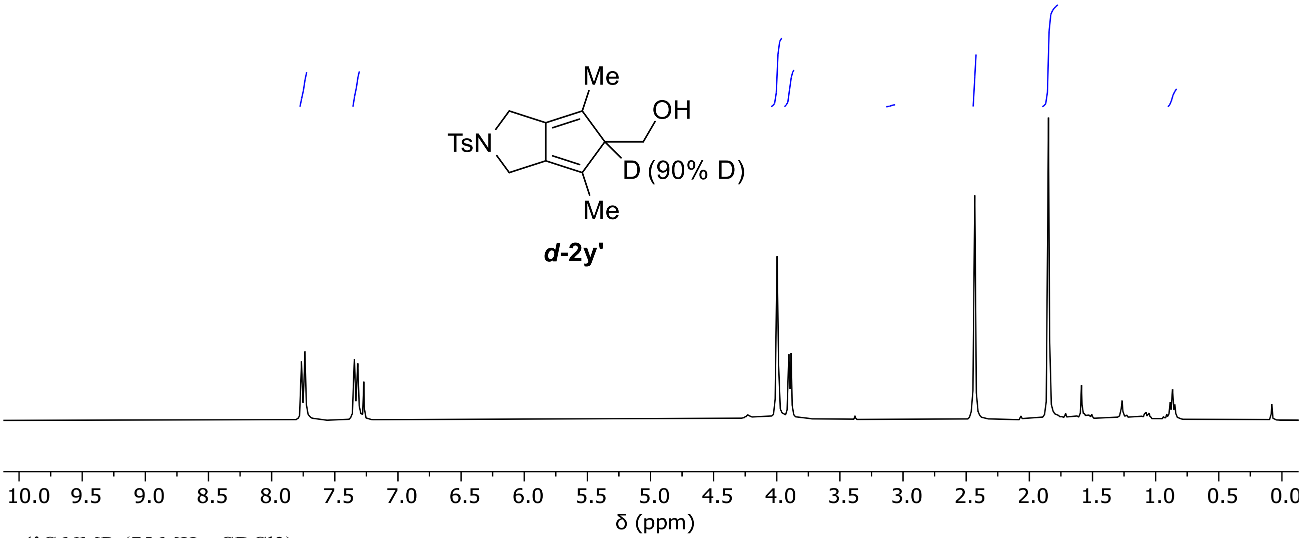

13C NMR (75 MHz, CDCl3)

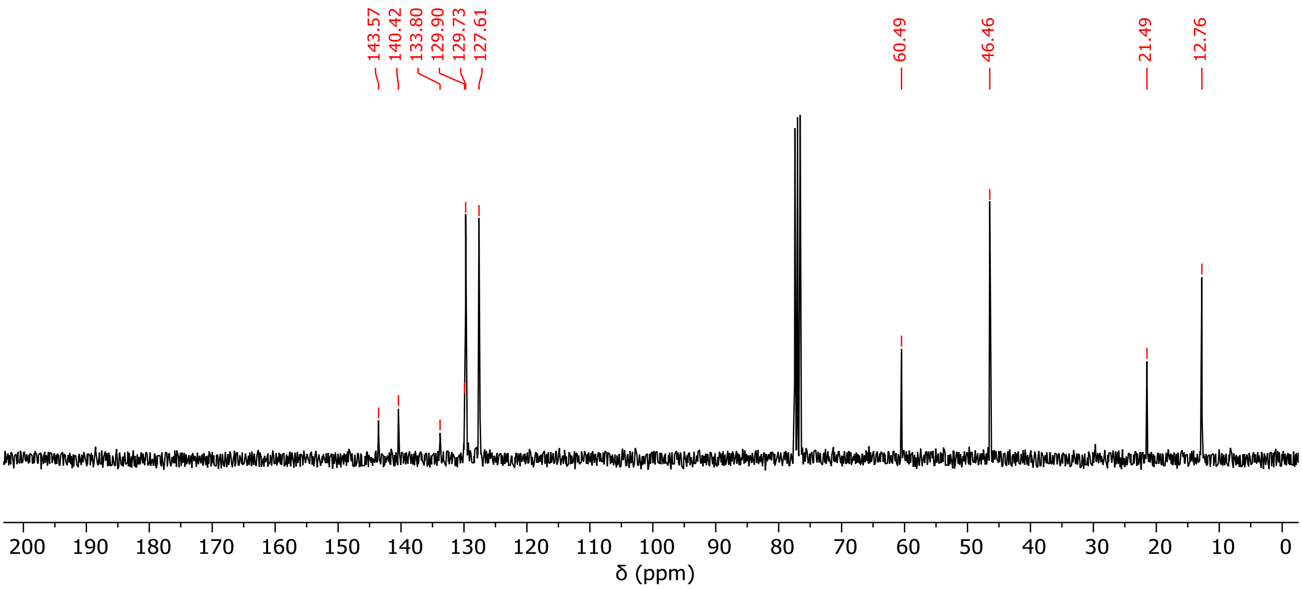

DEPT NMR (75 MHz, CDCl3)

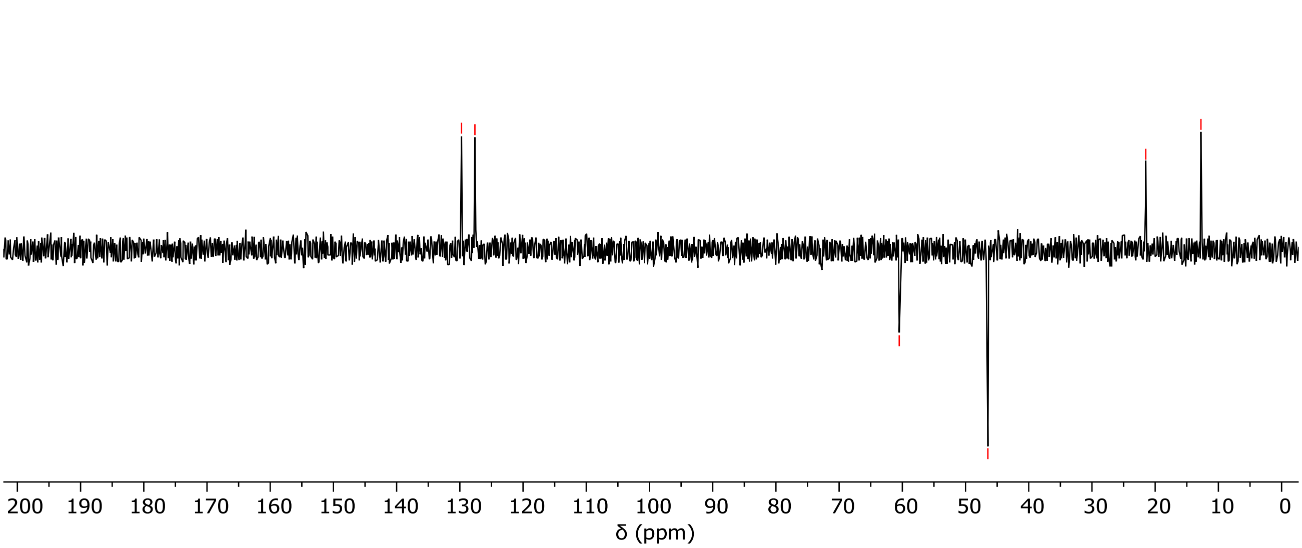

1H NMR (300 MHz, CDCl3)

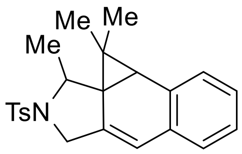

4zc

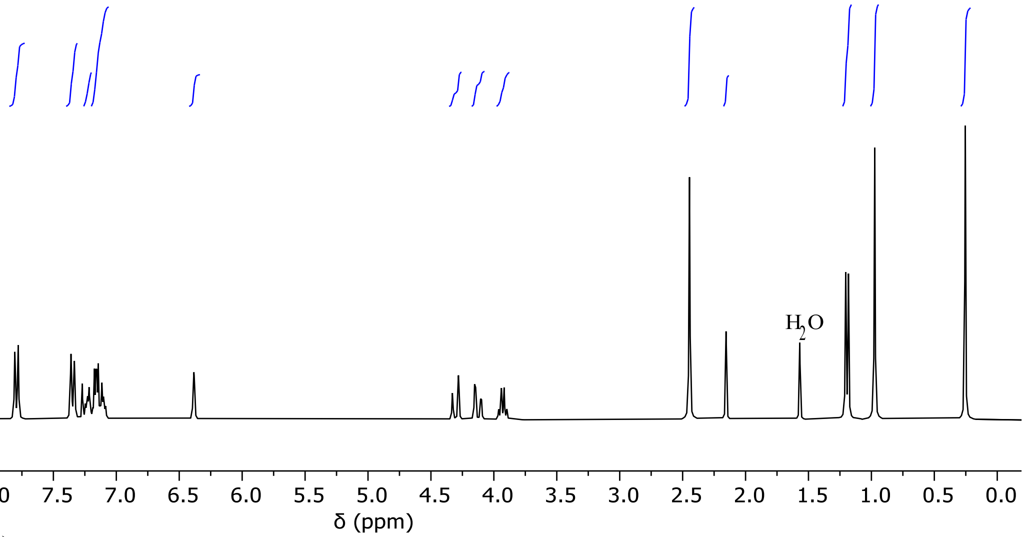

13C NMR (75 MHz, CDCl3)

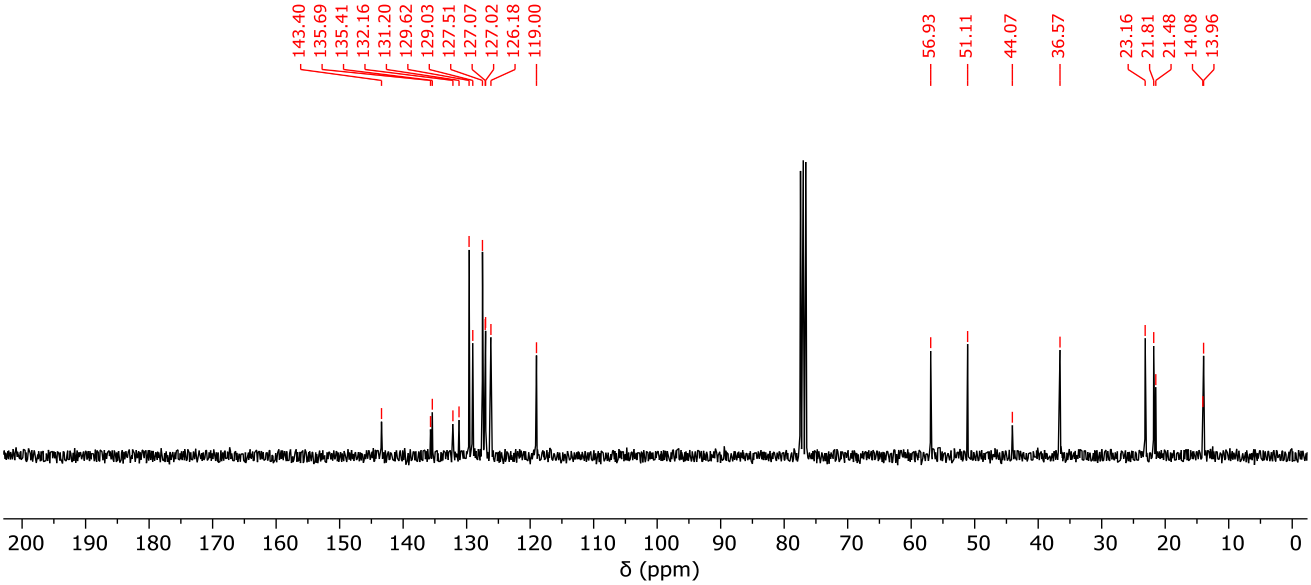

DEPT NMR (75 MHz, CDCl3)

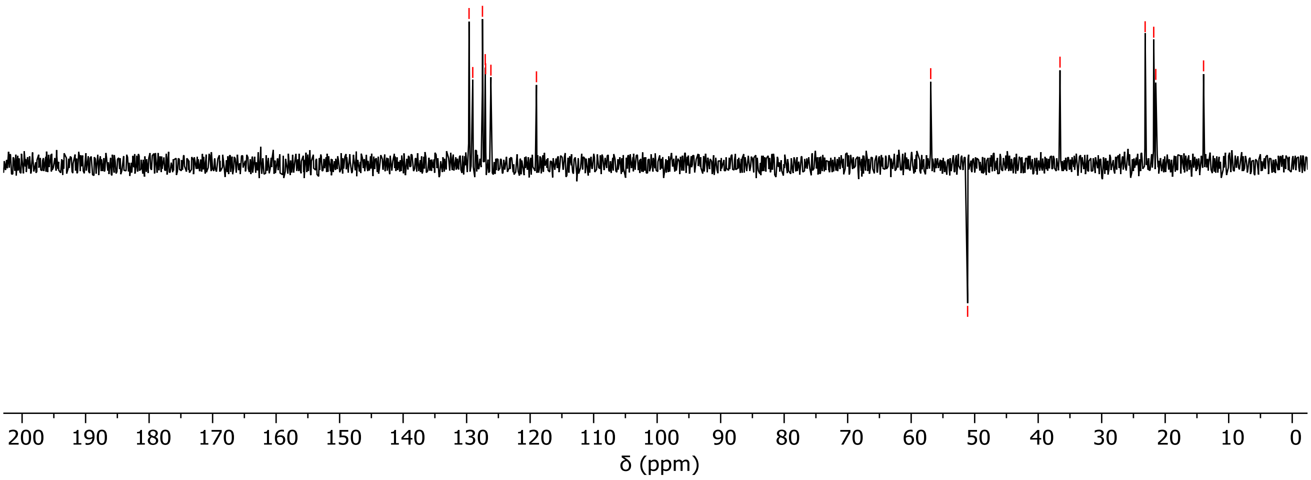

1H NMR (500 MHz, CDCl3)

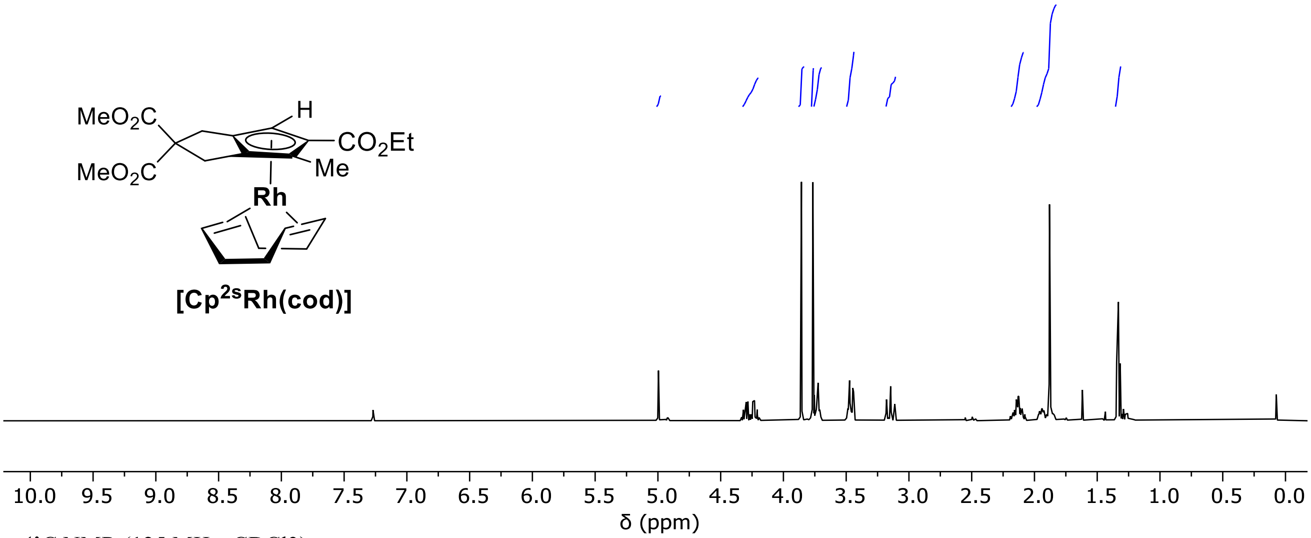

13C NMR (125 MHz, CDCl3)

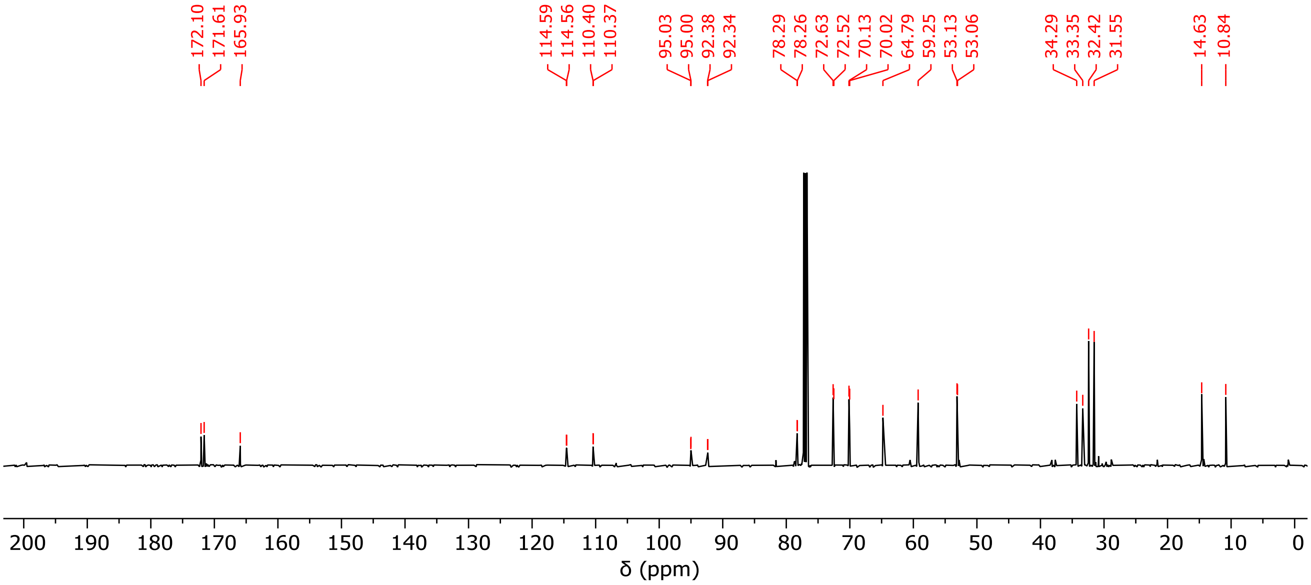

DEPT NMR (125 MHz, CDCl3)

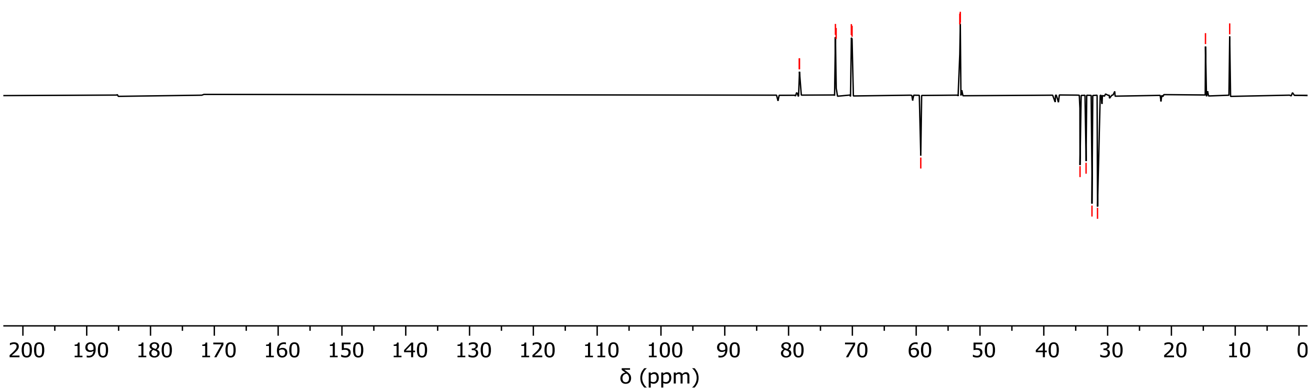

1H NMR (300 MHz, CDCl3)

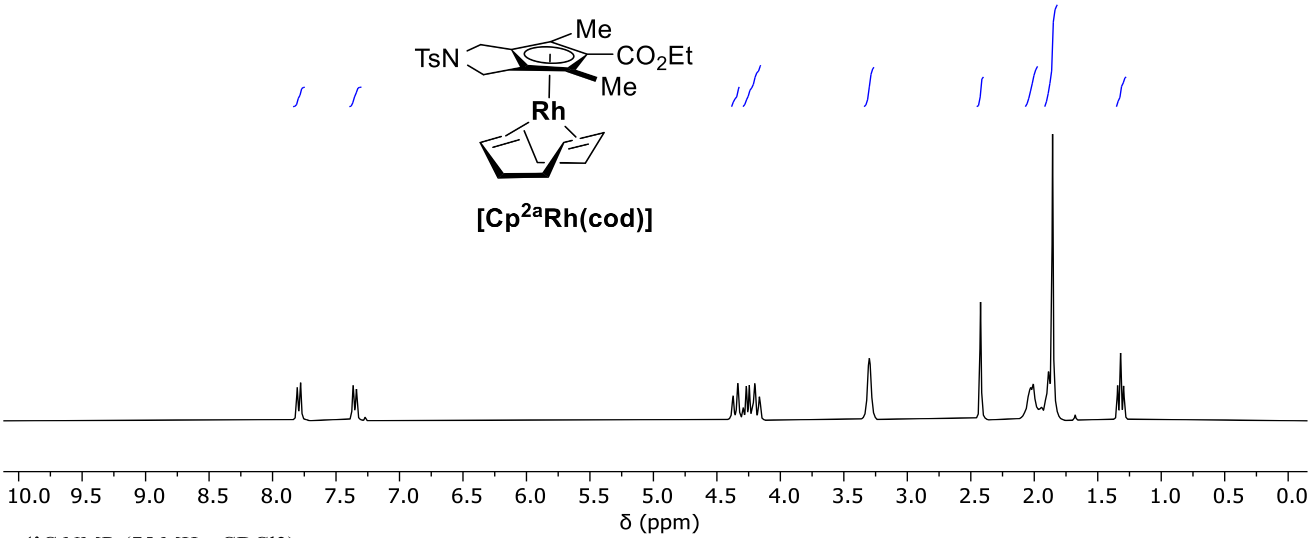

13C NMR (75 MHz, CDCl3)

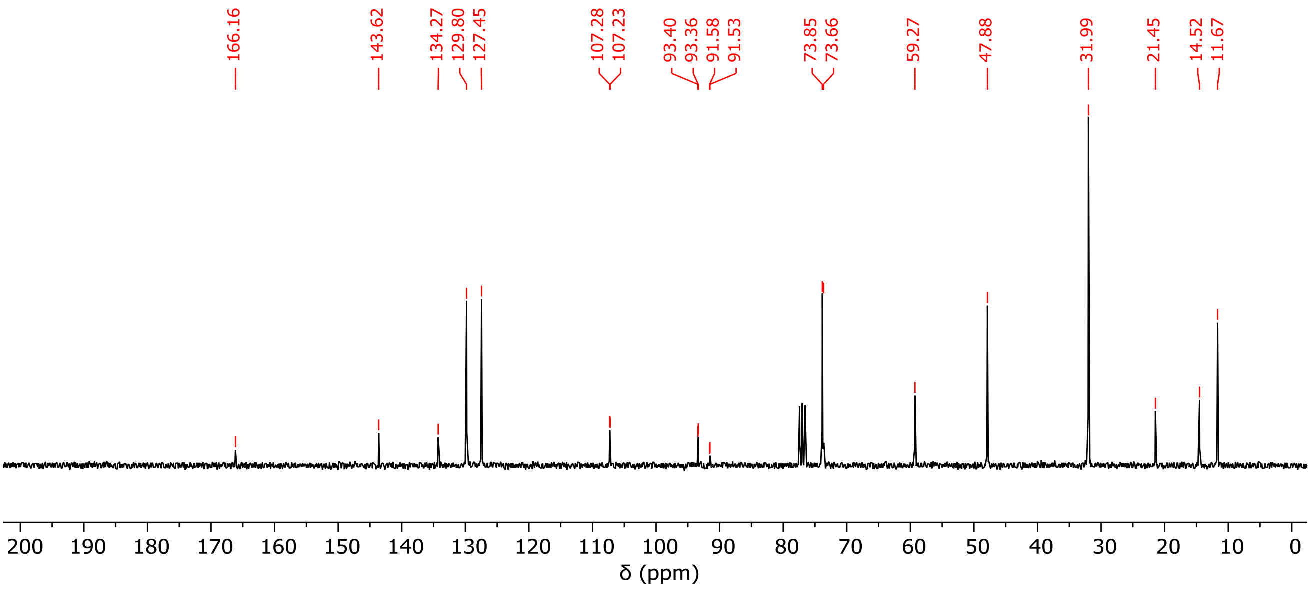

DEPT NMR (75 MHz, CDCl3)

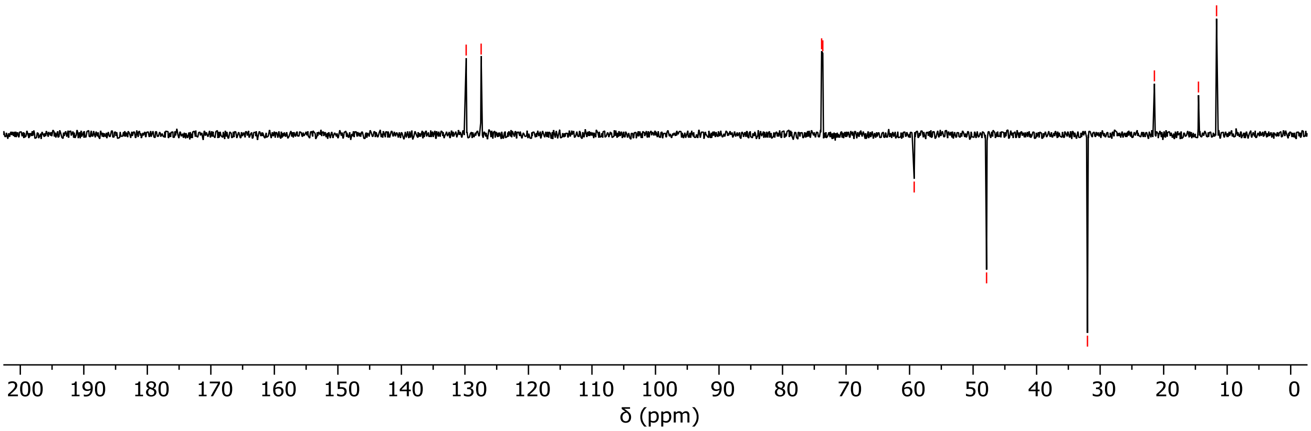

1H NMR (500 MHz, CDCl3)

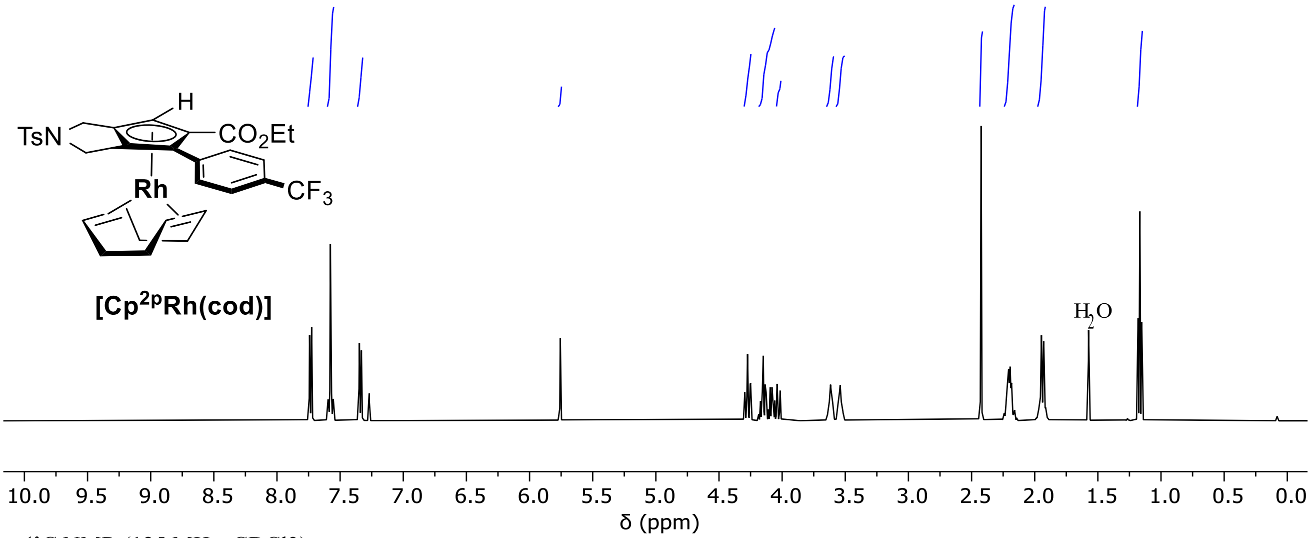

13C NMR (125 MHz, CDCl3)

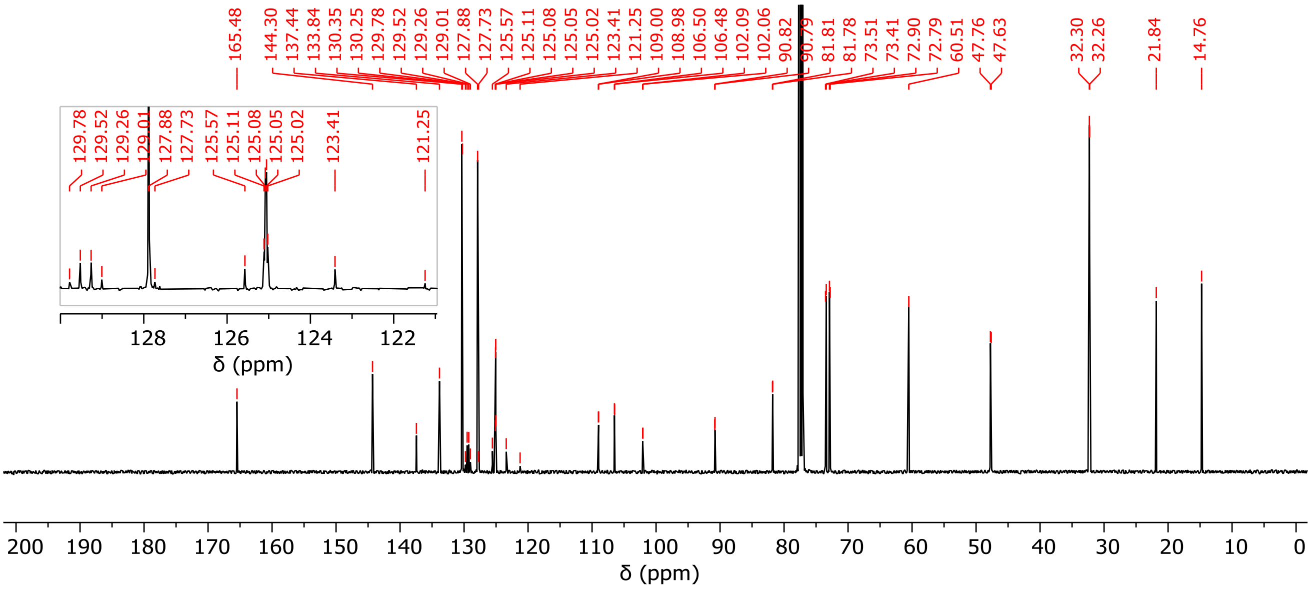

DEPT NMR (125 MHz, CDCl3)

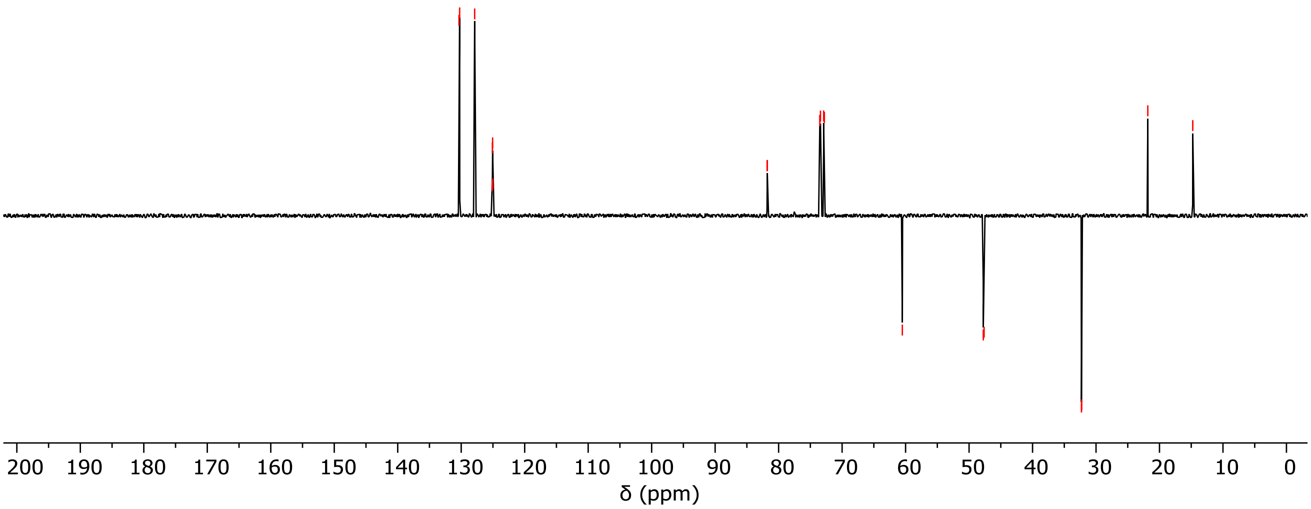

19F NMR (470 MHz, CDCl3)

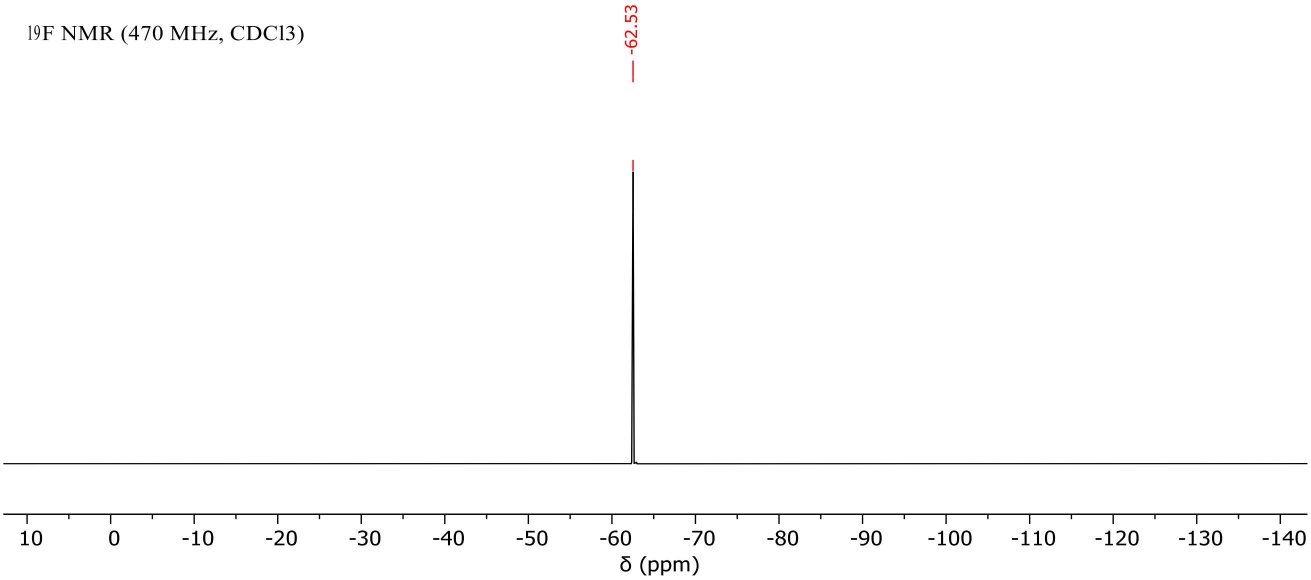

1H NMR (500 MHz, CDCl3)

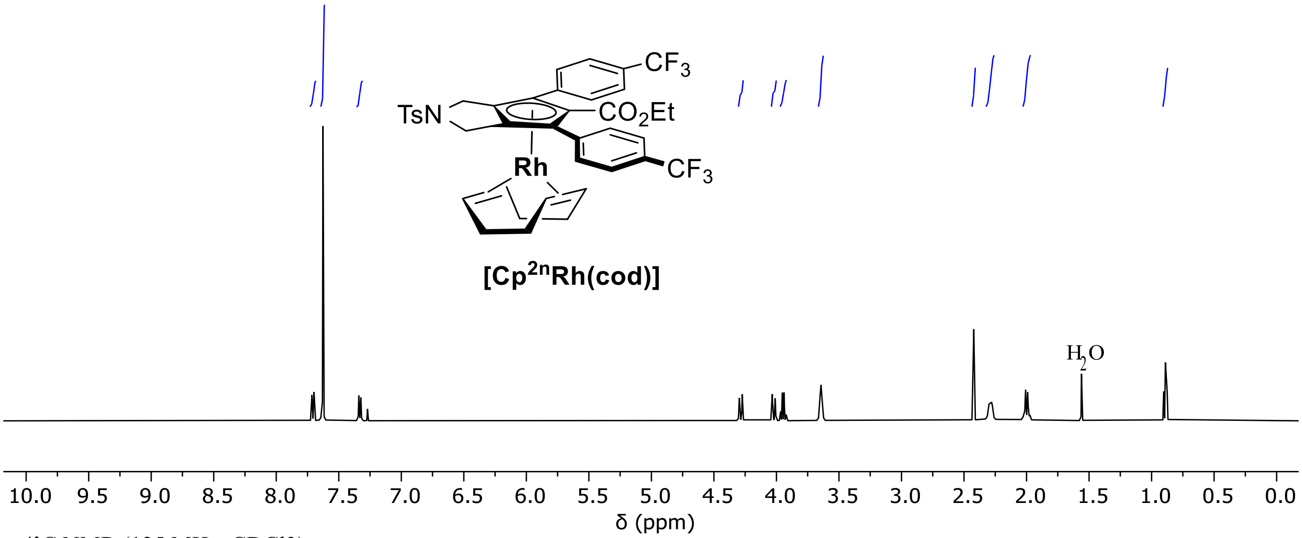

13C NMR (125 MHz, CDCl3)

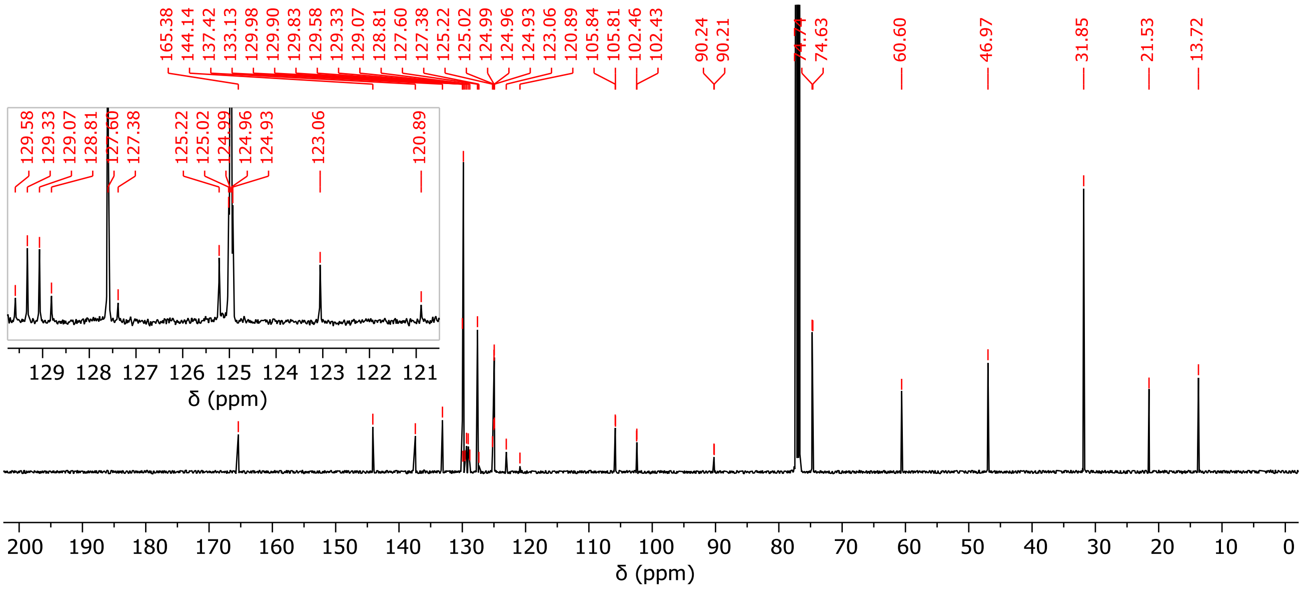

DEPT NMR (125 MHz, CDCl3)

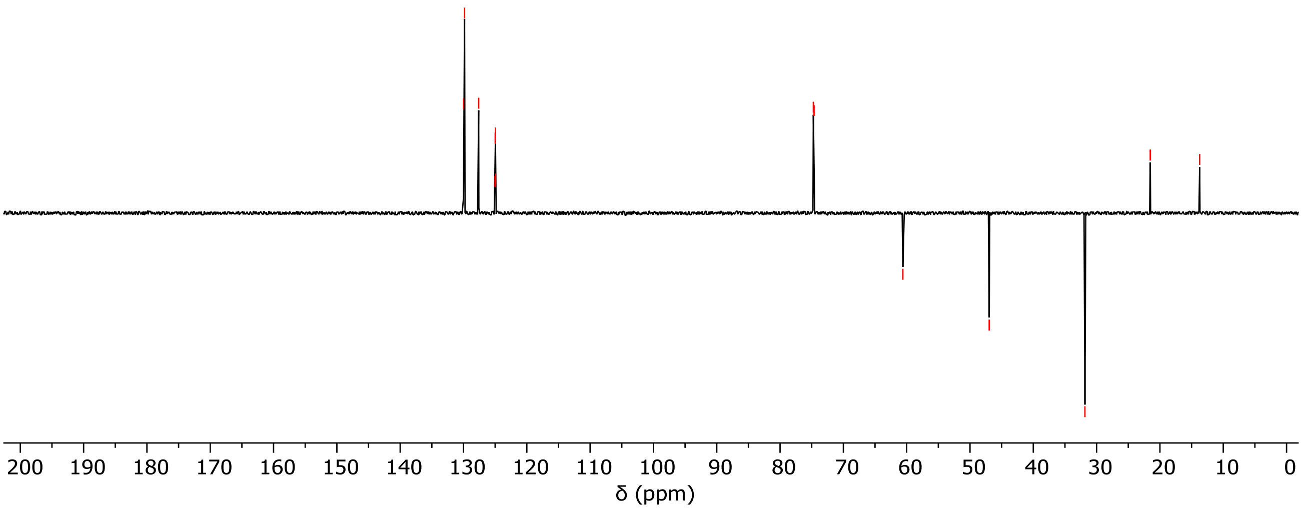

19F NMR (470 MHz, CDCl3)

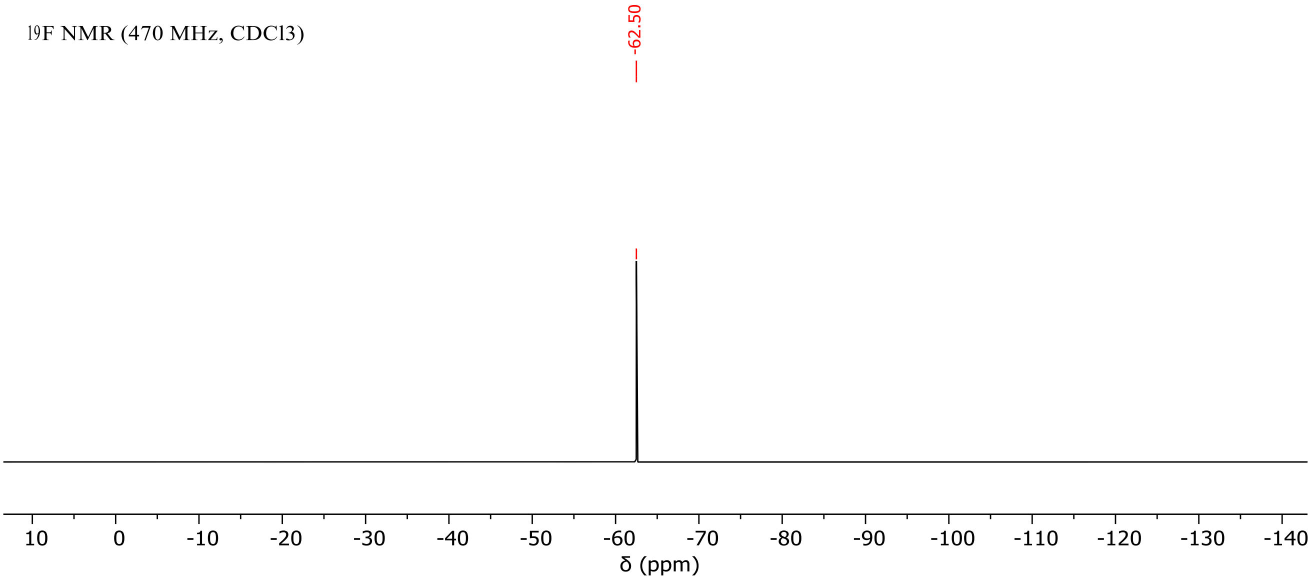

1H NMR (500 MHz, DMSO)

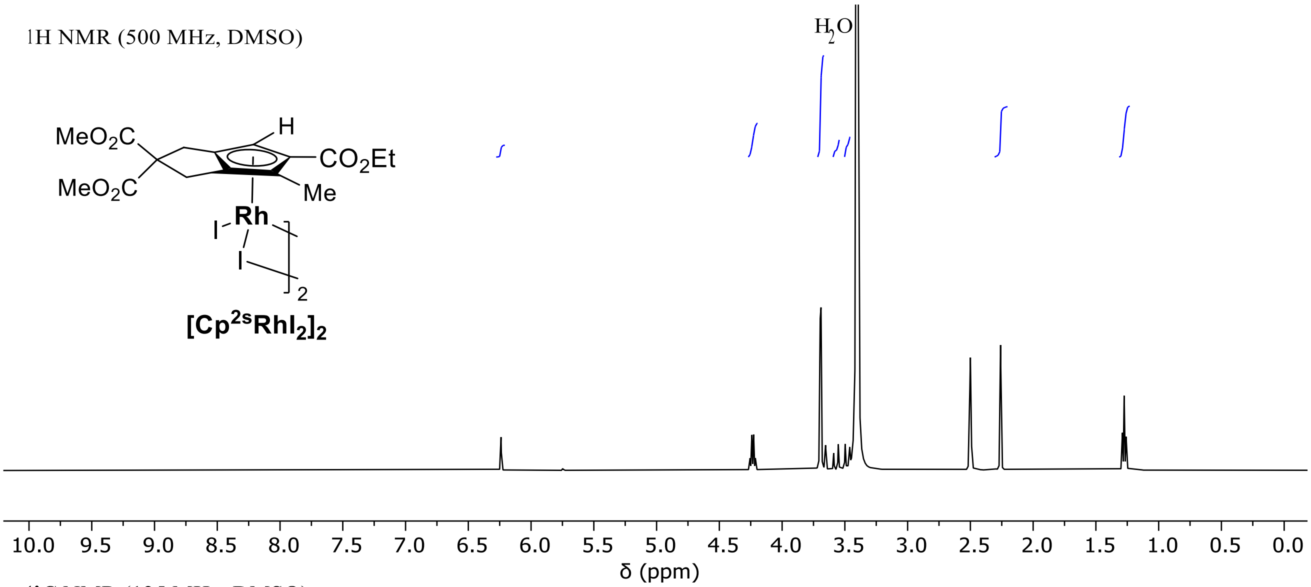

13C NMR (125 MHz, DMSO)

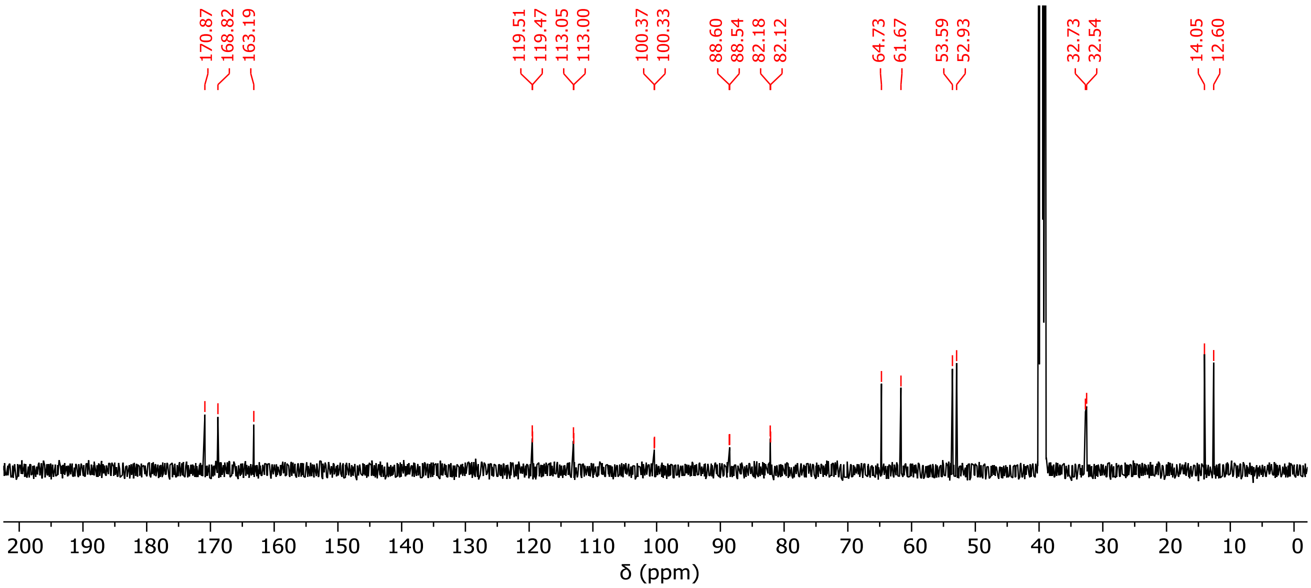

DEPT NMR (125 MHz, DMSO)

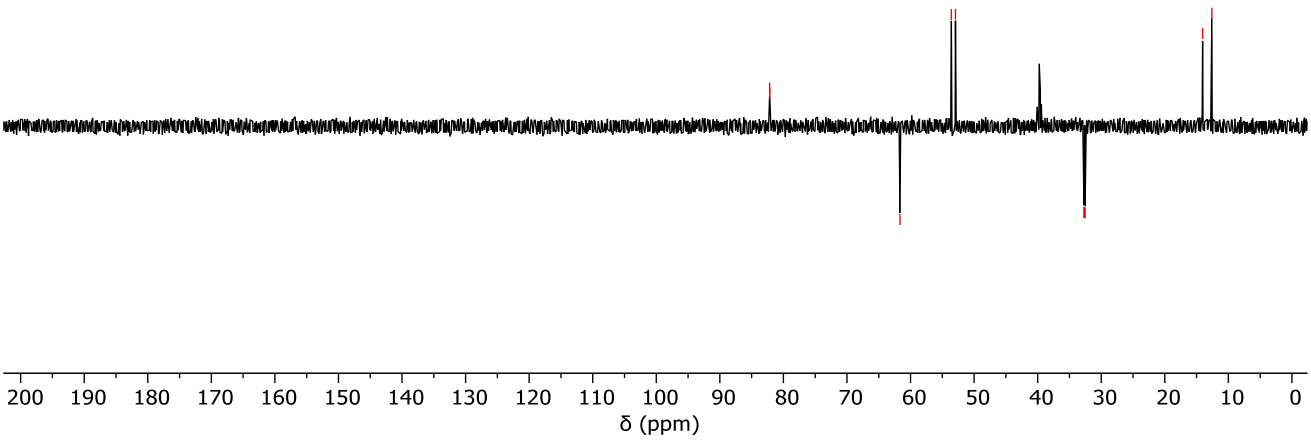

1H NMR (300 MHz, DMSO)

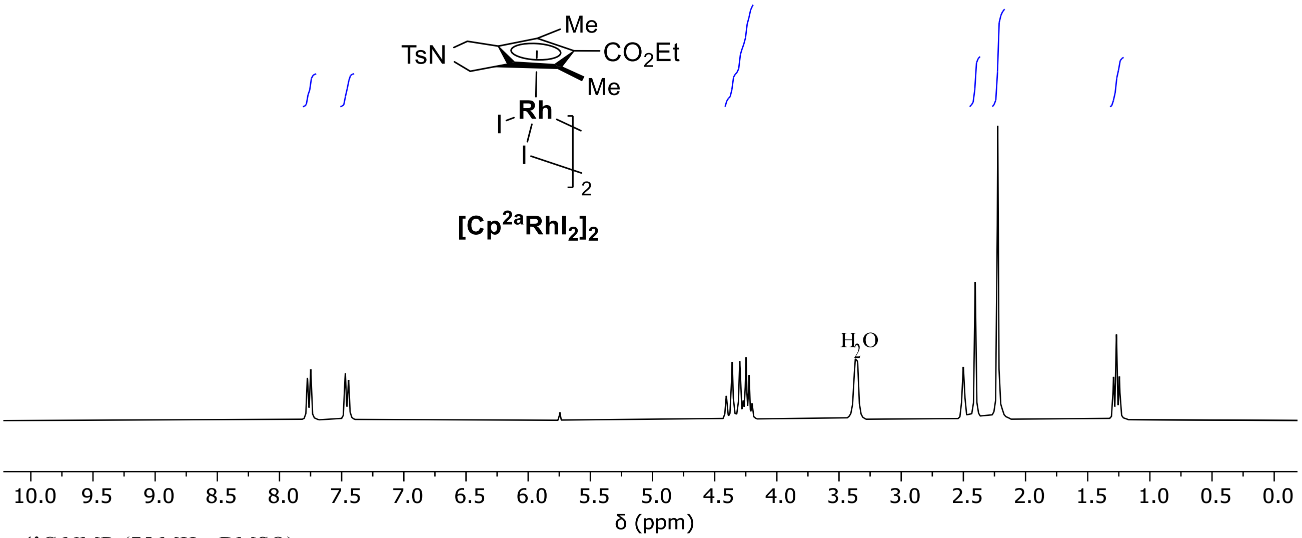

13C NMR (75 MHz, DMSO)

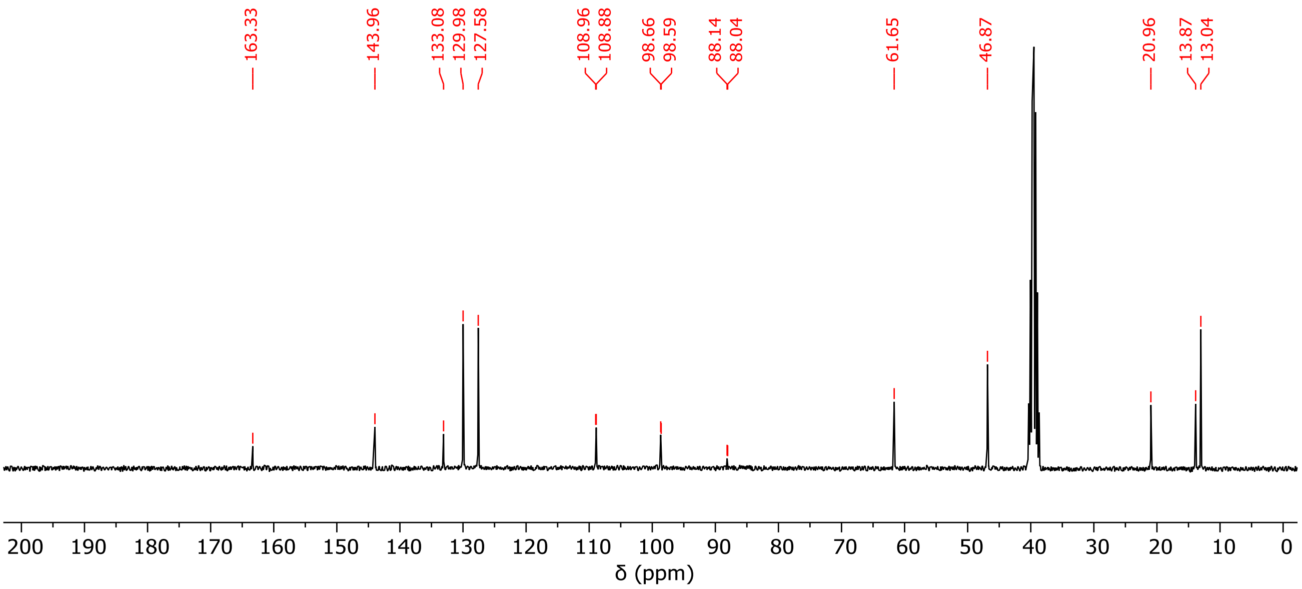

DEPT NMR (75 MHz, DMSO)

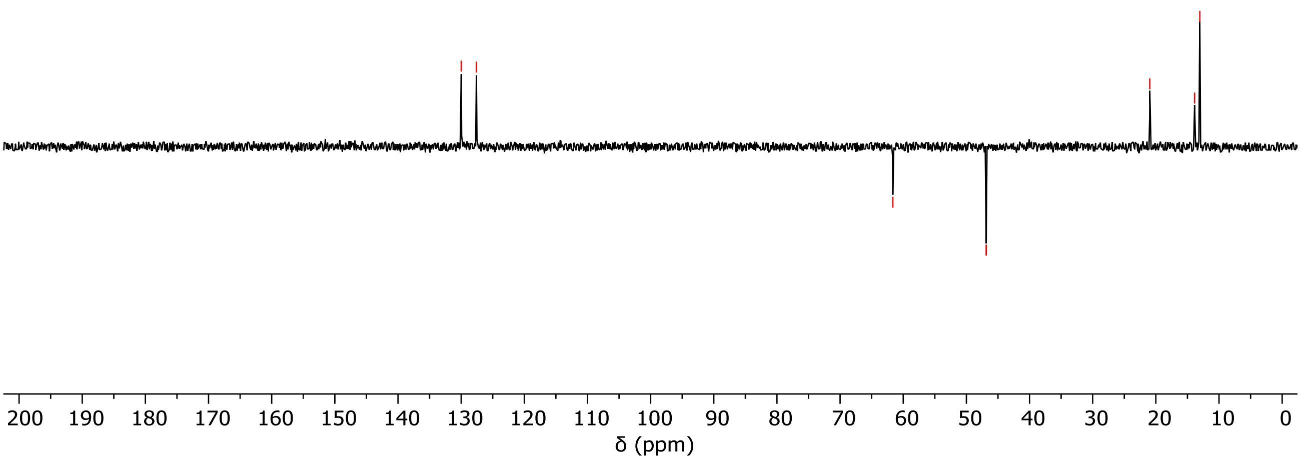

1H NMR (500 MHz, DMSO)

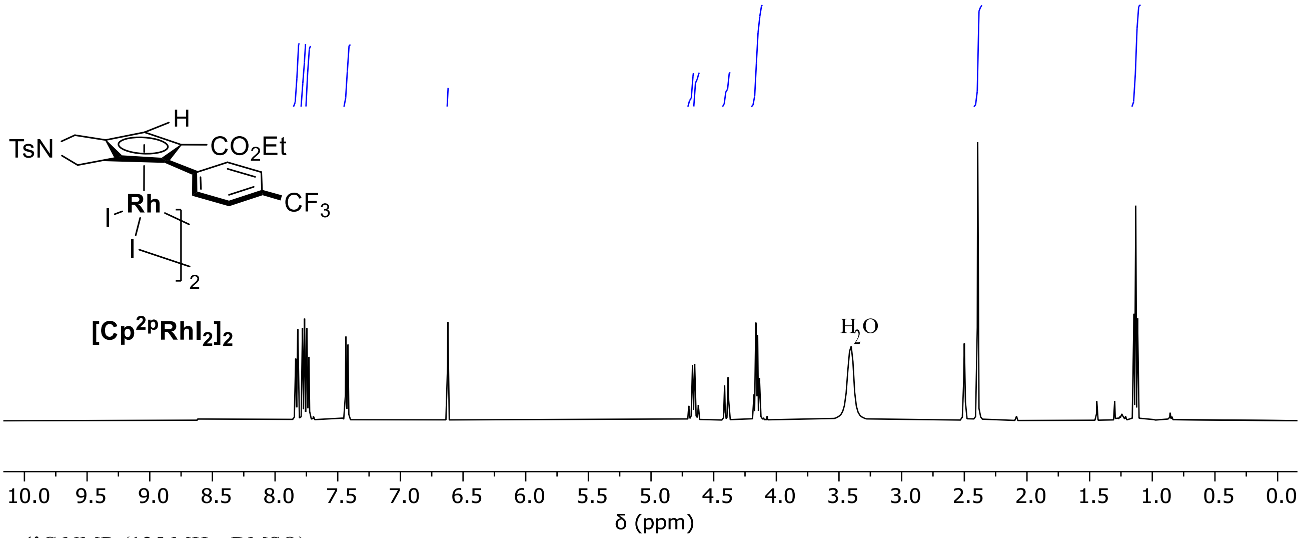

13C NMR (125 MHz, DMSO)

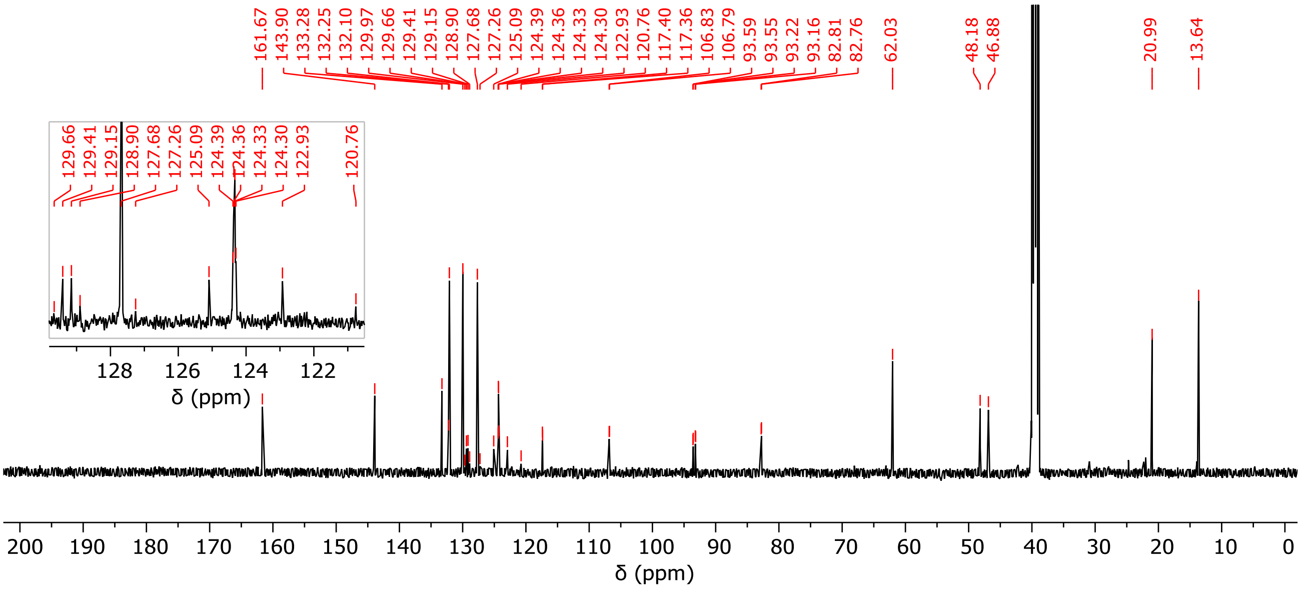

DEPT NMR (125 MHz, DMSO)

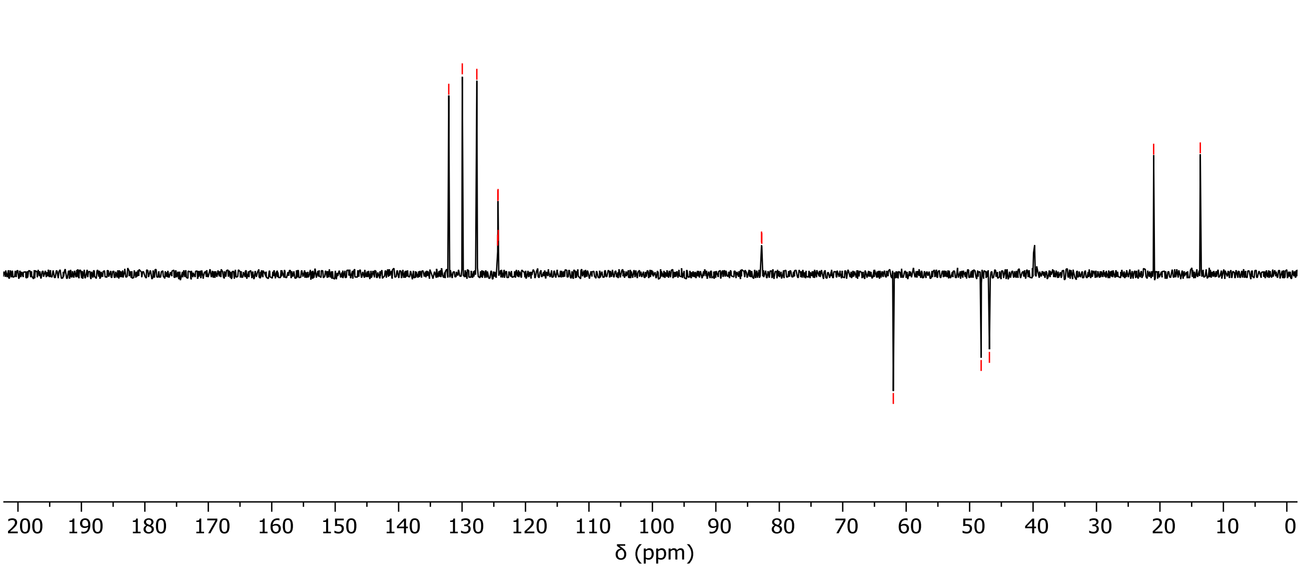

^{19}F NMR (470 MHz, DMSO)

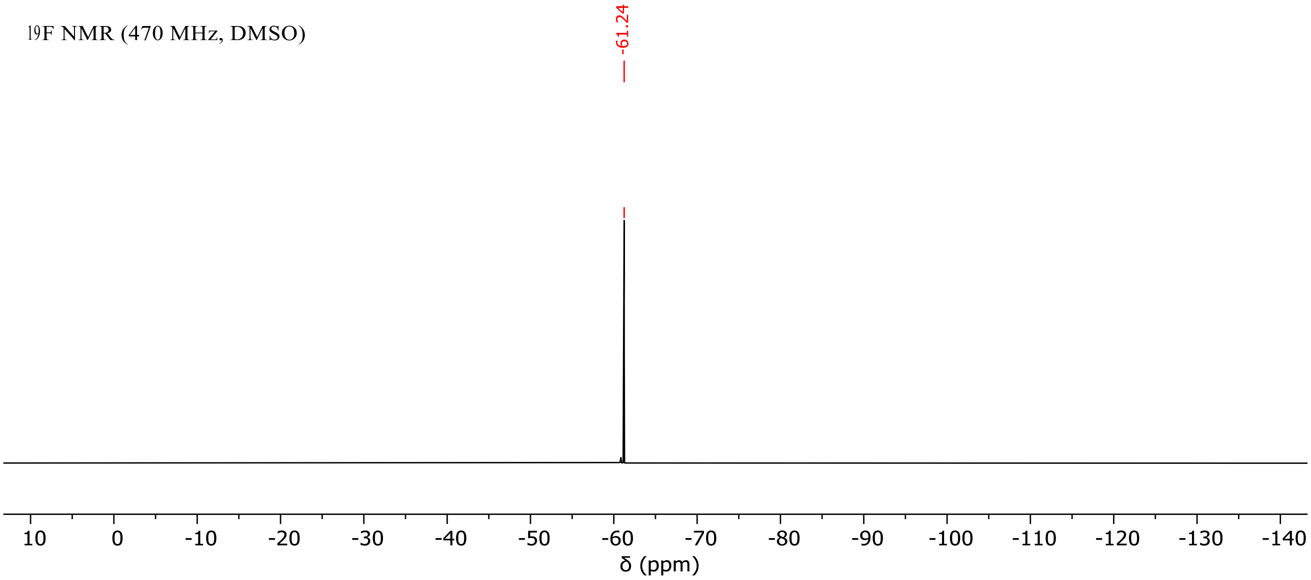

1H NMR (500 MHz, DMSO)

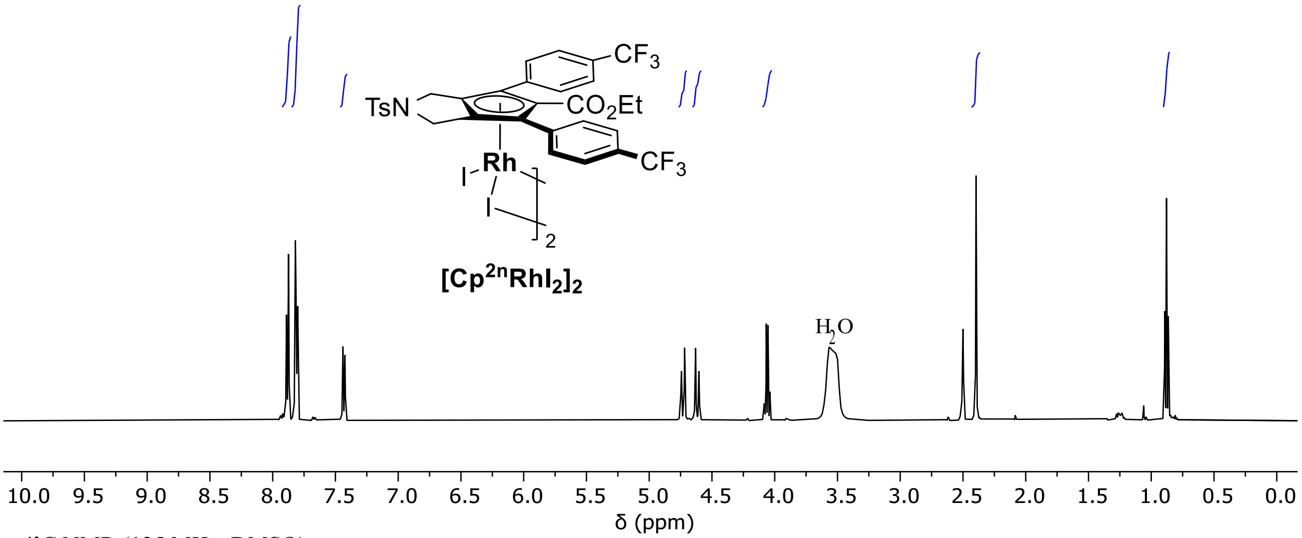

13C NMR (125 MHz, DMSO)

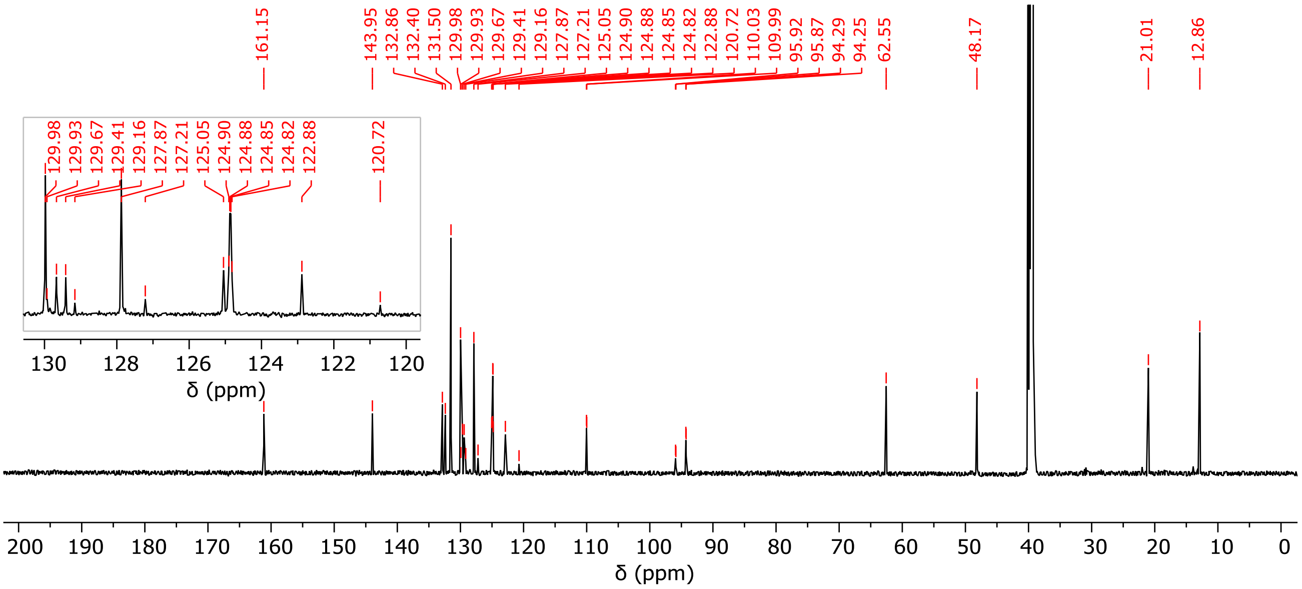

DEPT NMR (125 MHz, DMSO)

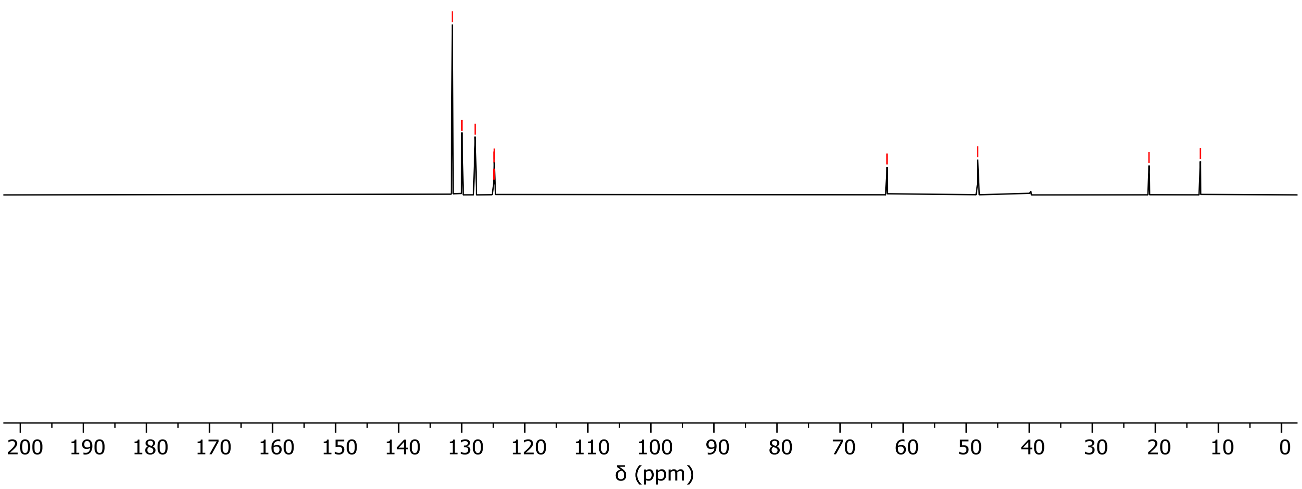

19F NMR (470 MHz, DMSO)

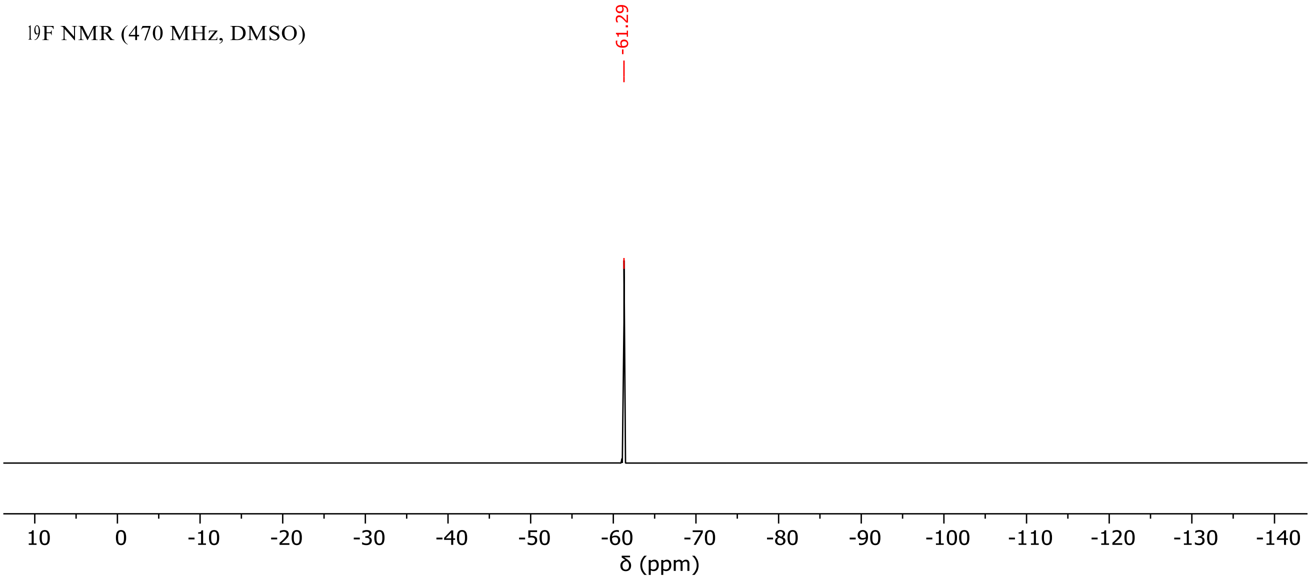

1H NMR (300 MHz, CDCl3)

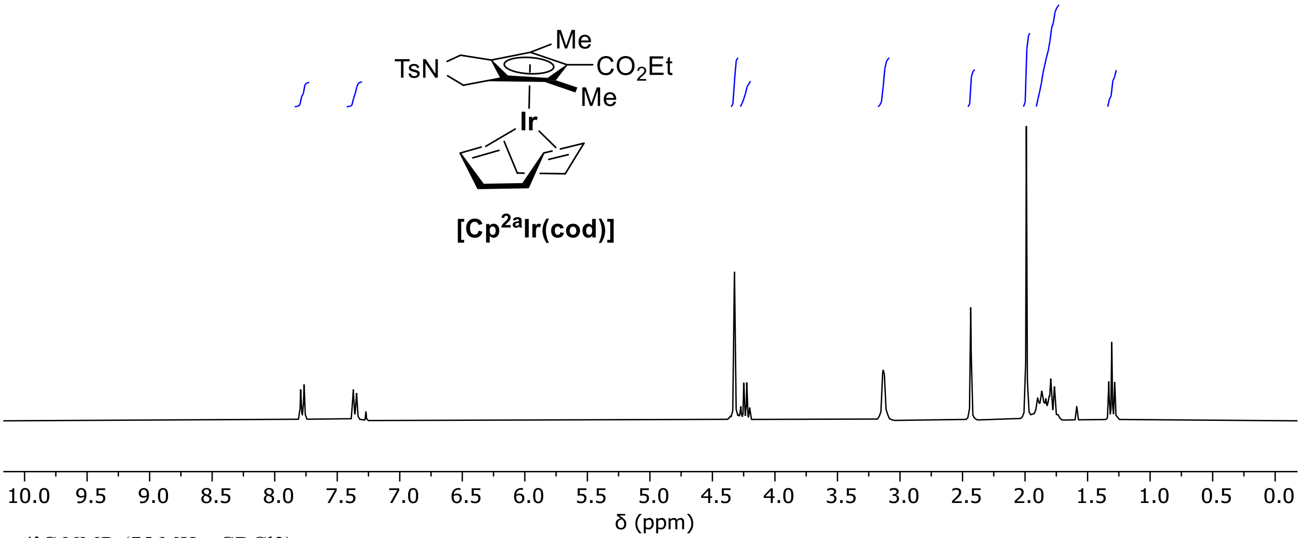

13C NMR (75 MHz, CDCl3)

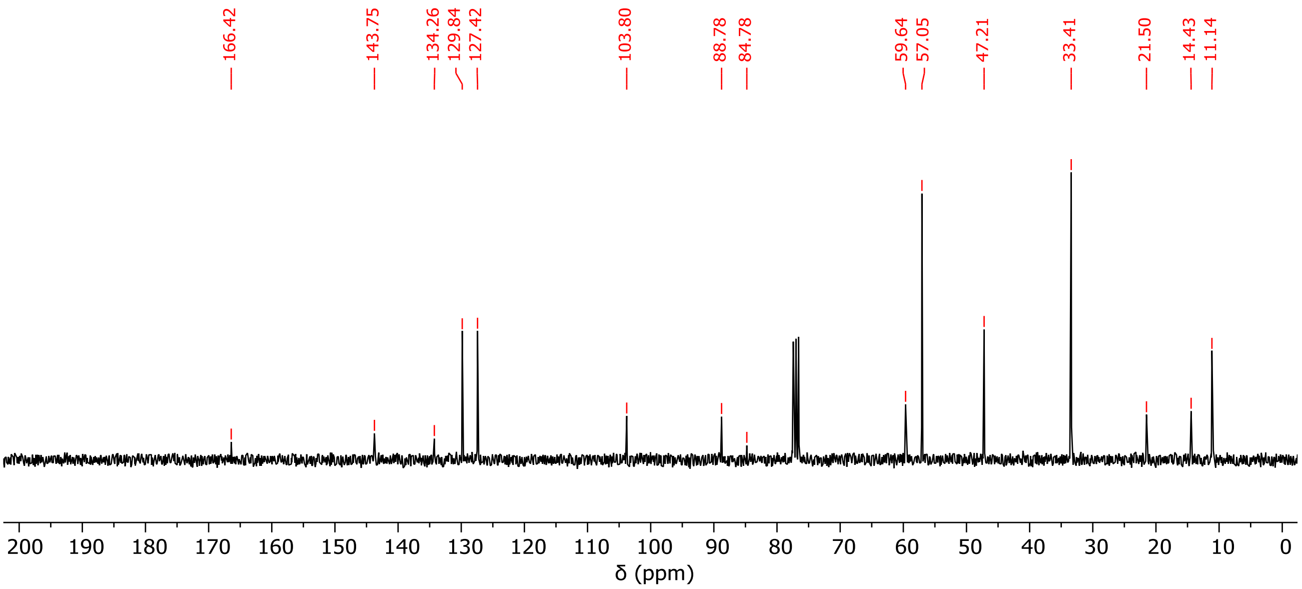

DEPT NMR (75 MHz, CDCl3)

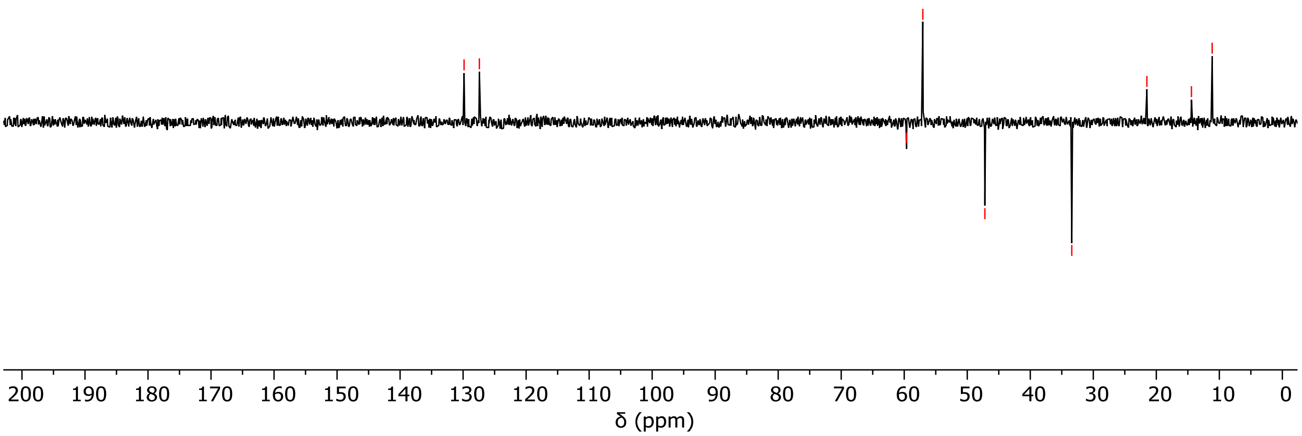

1H NMR (300 MHz, CDCl3)

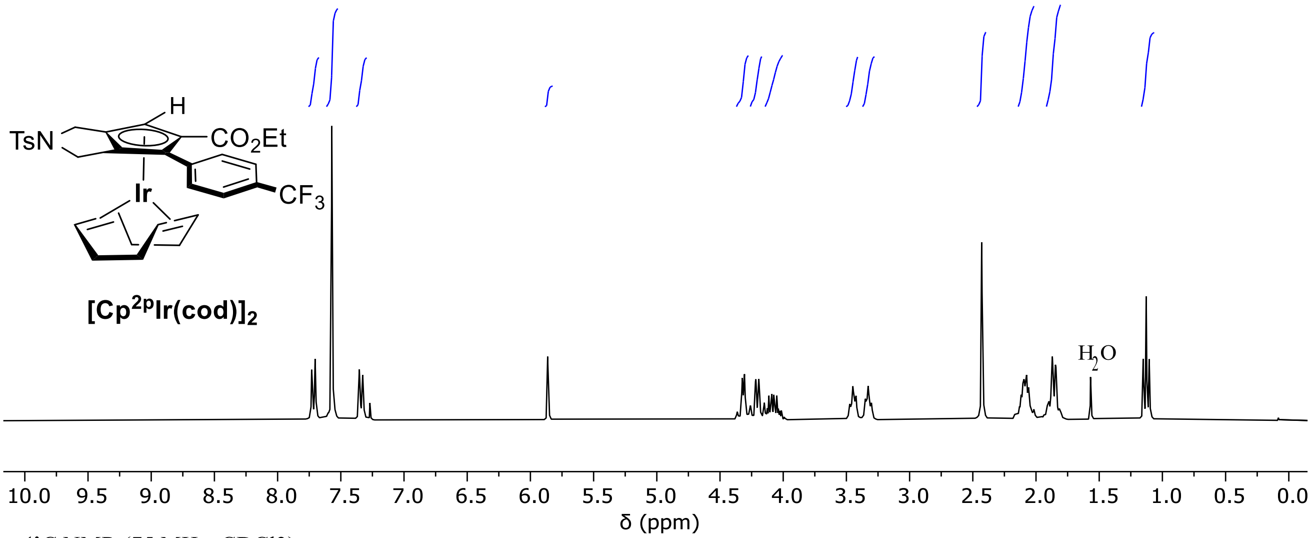

13C NMR (75 MHz, CDCl3)

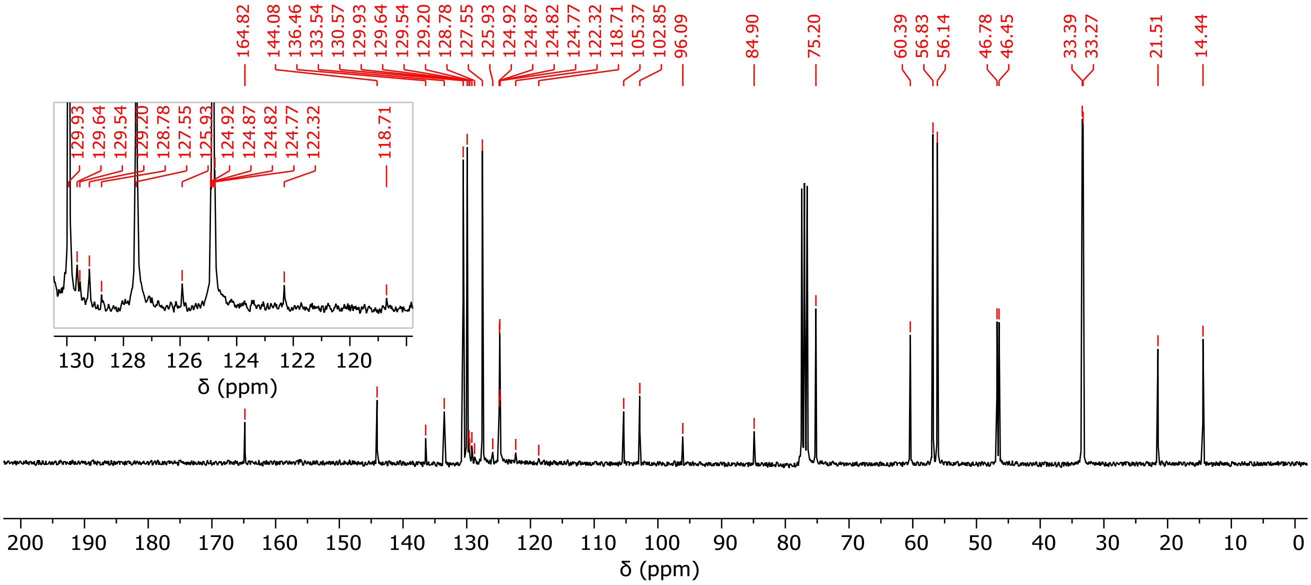

DEPT NMR (75 MHz, CDCl3)

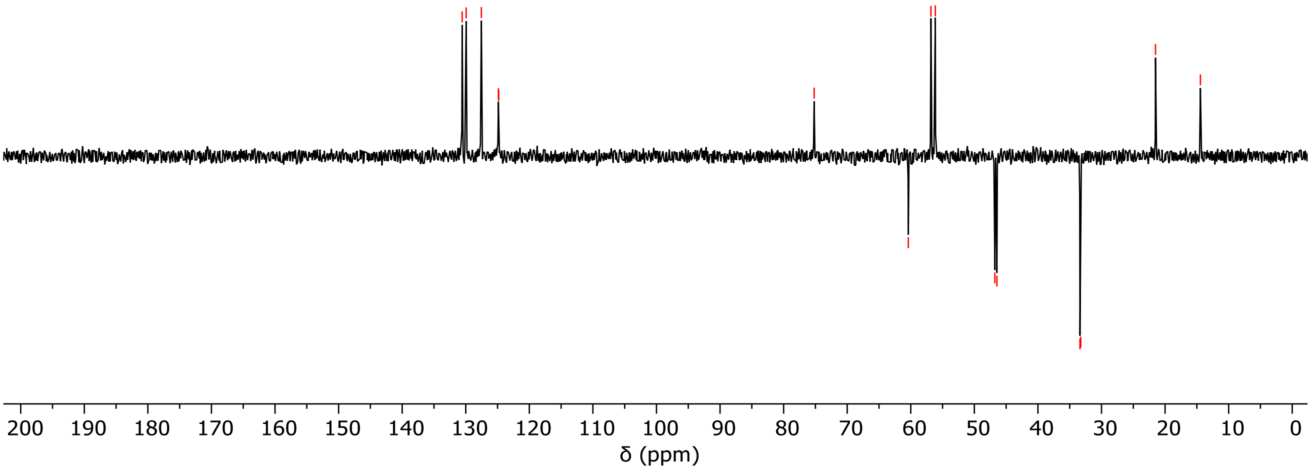

^{19}F NMR (282 MHz, $CDCl_3$)

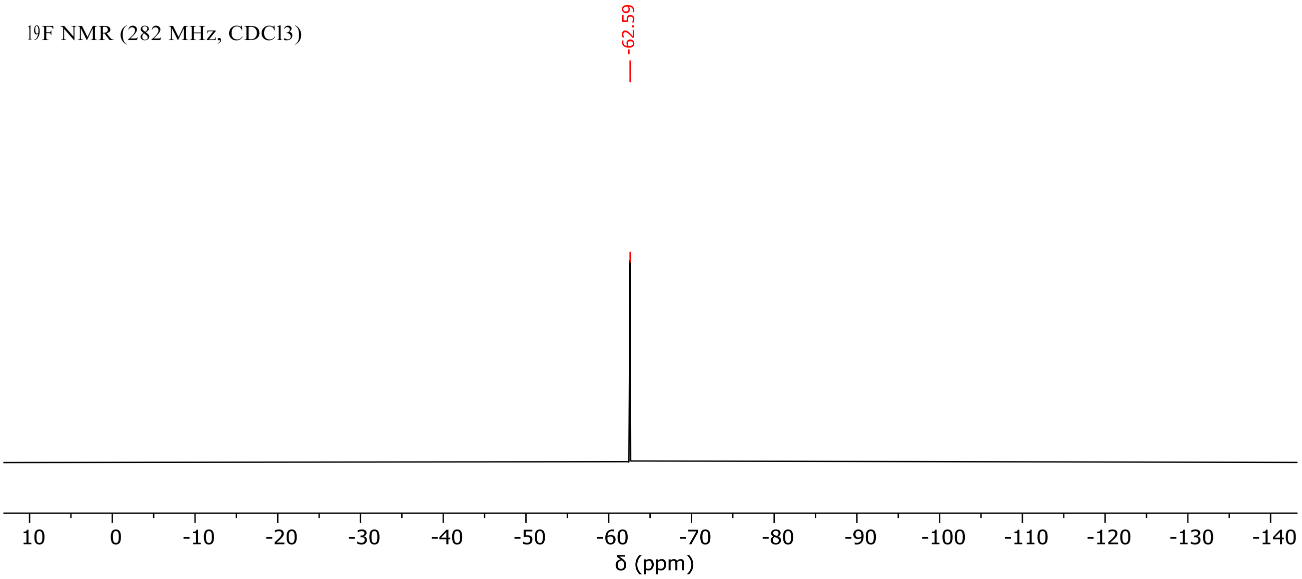

1H NMR (500 MHz, CDCl3)

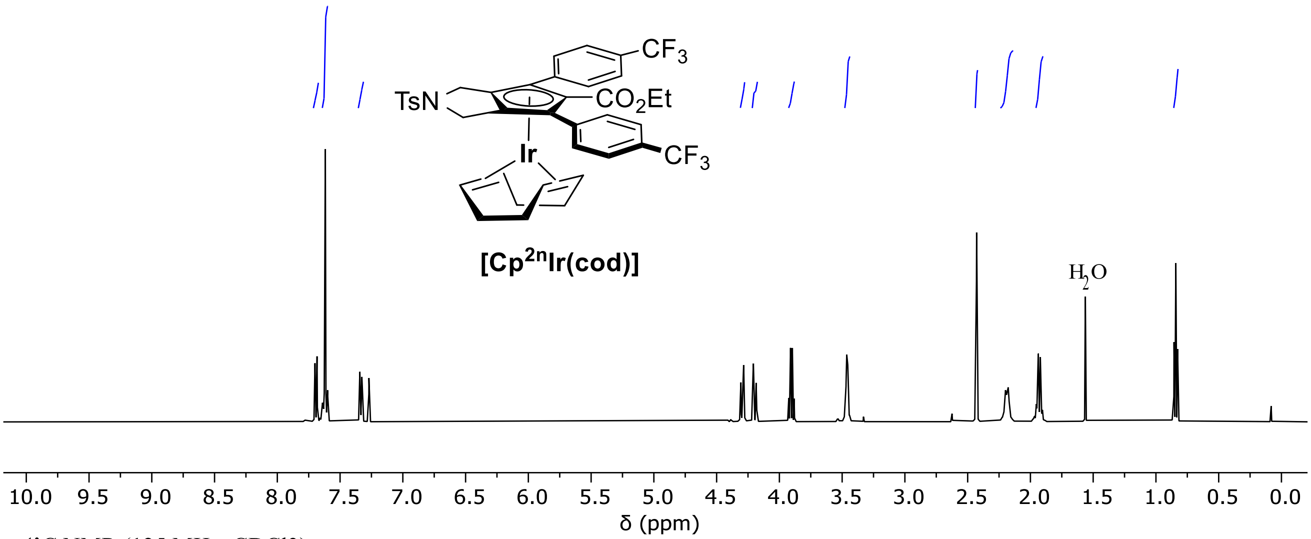

13C NMR (125 MHz, CDCl3)

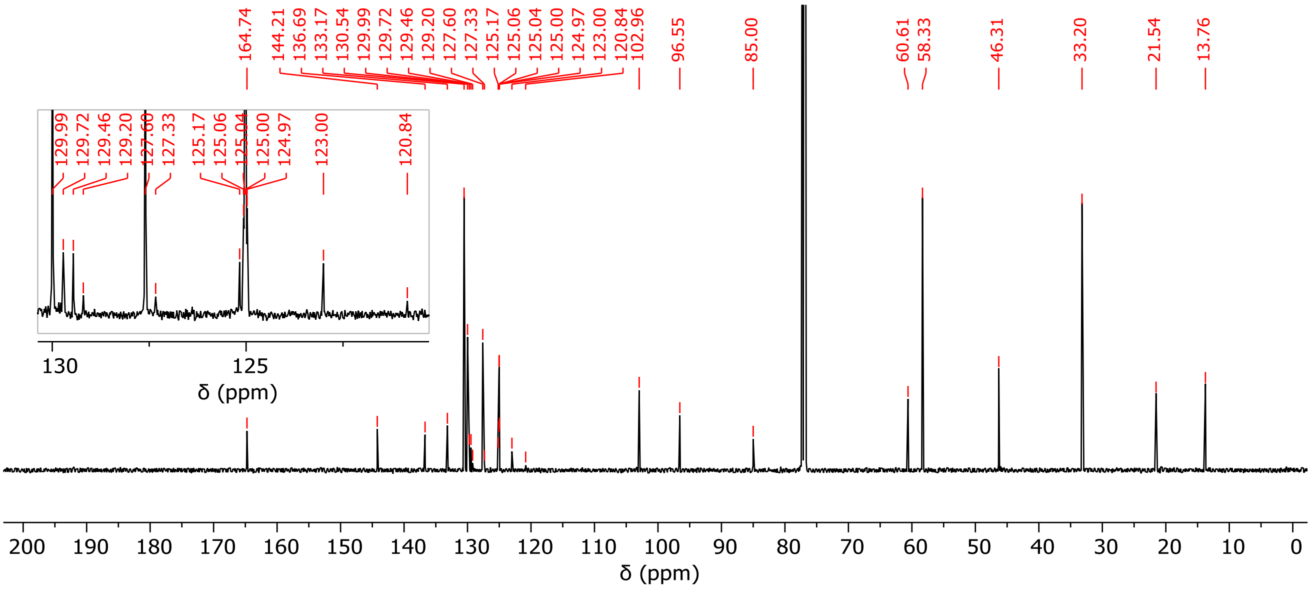

DEPT NMR (125 MHz, CDCl3)

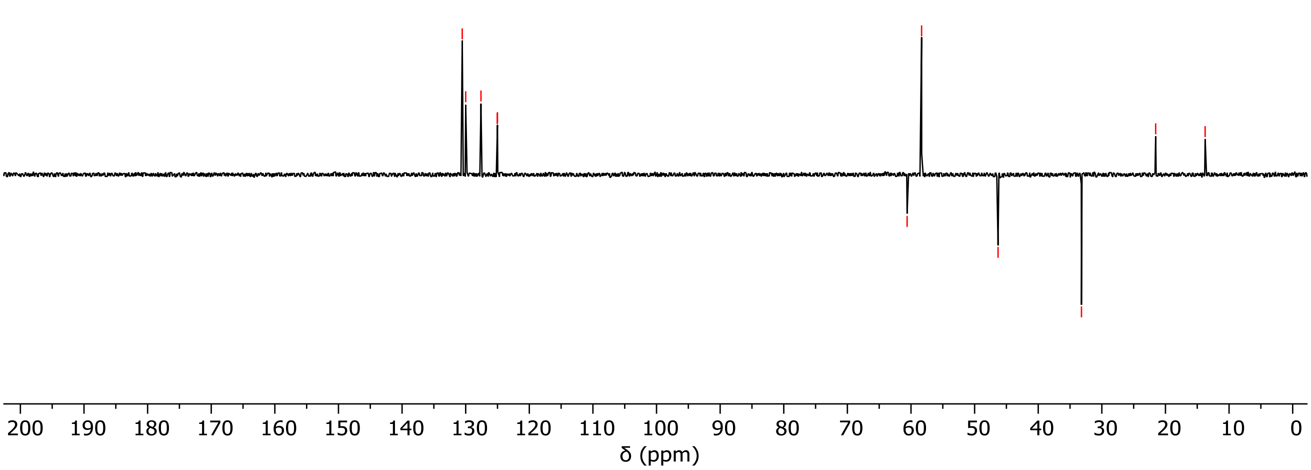

19F NMR (470 MHz, CDCl3)

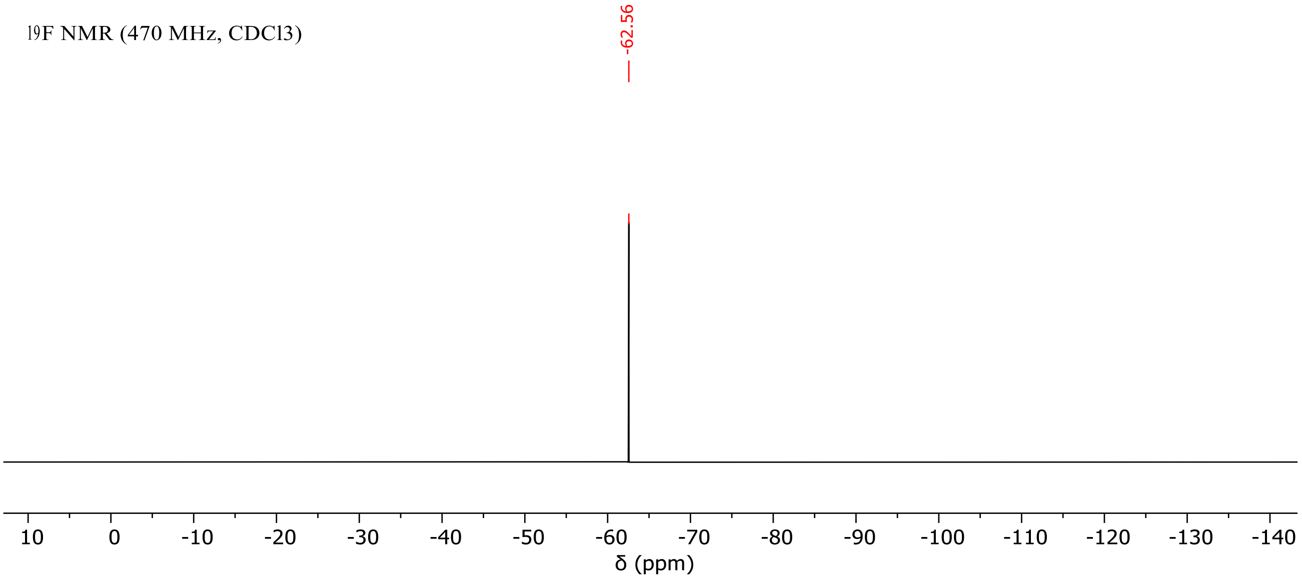

1H NMR (500 MHz, CDCl3)

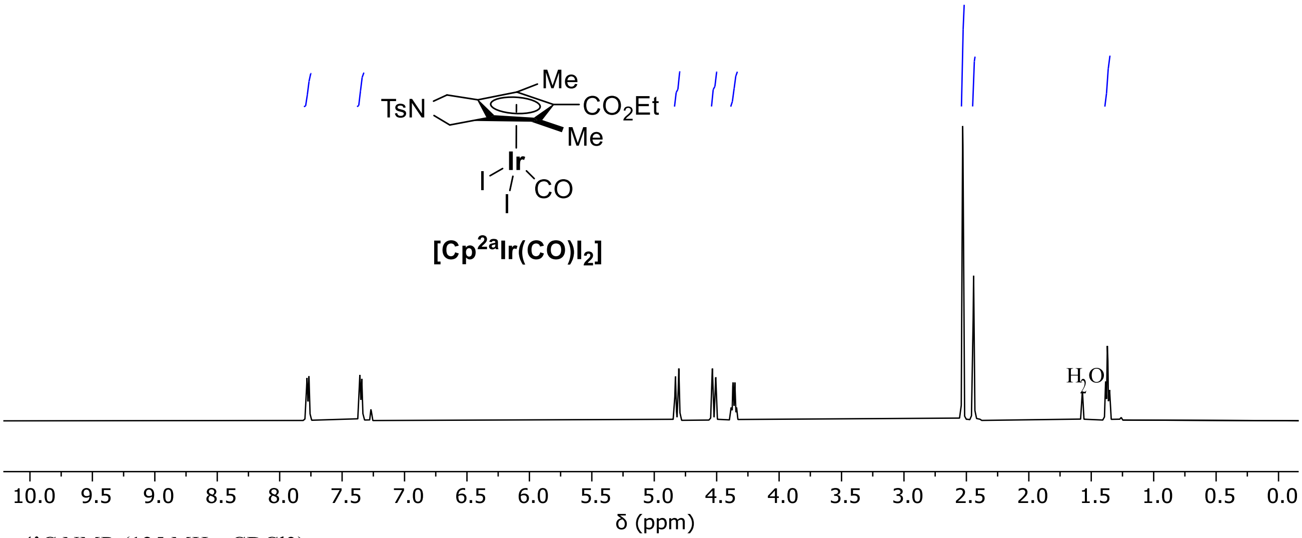

13C NMR (125 MHz, CDCl3)

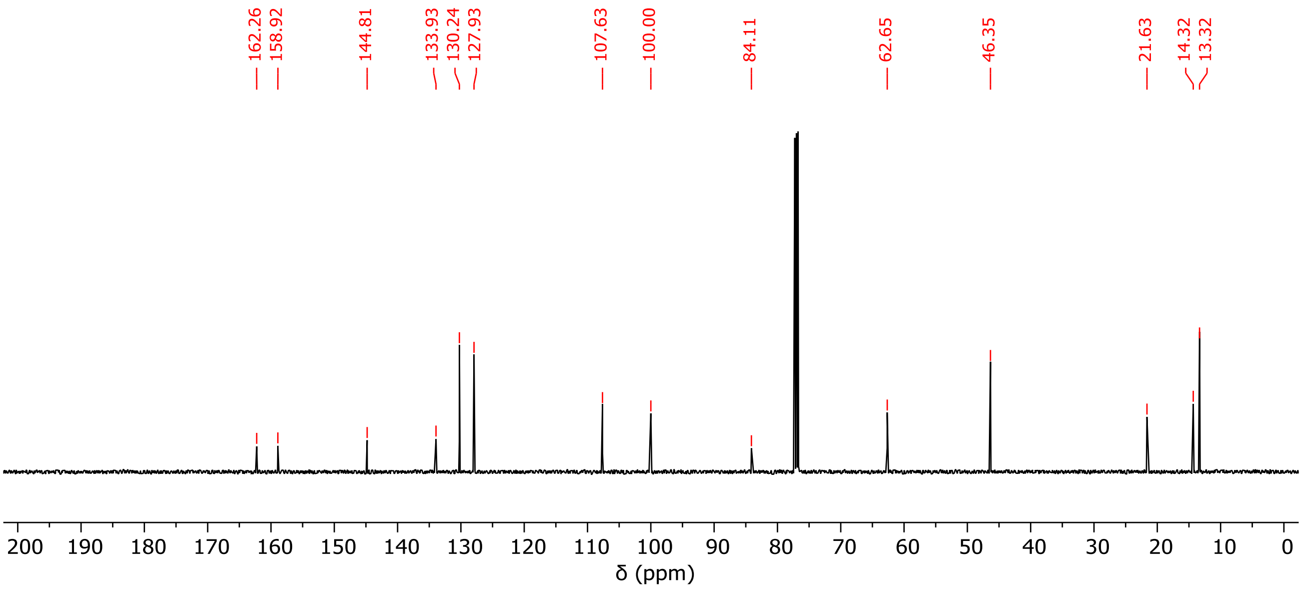

DEPT NMR (125 MHz, CDCl3)

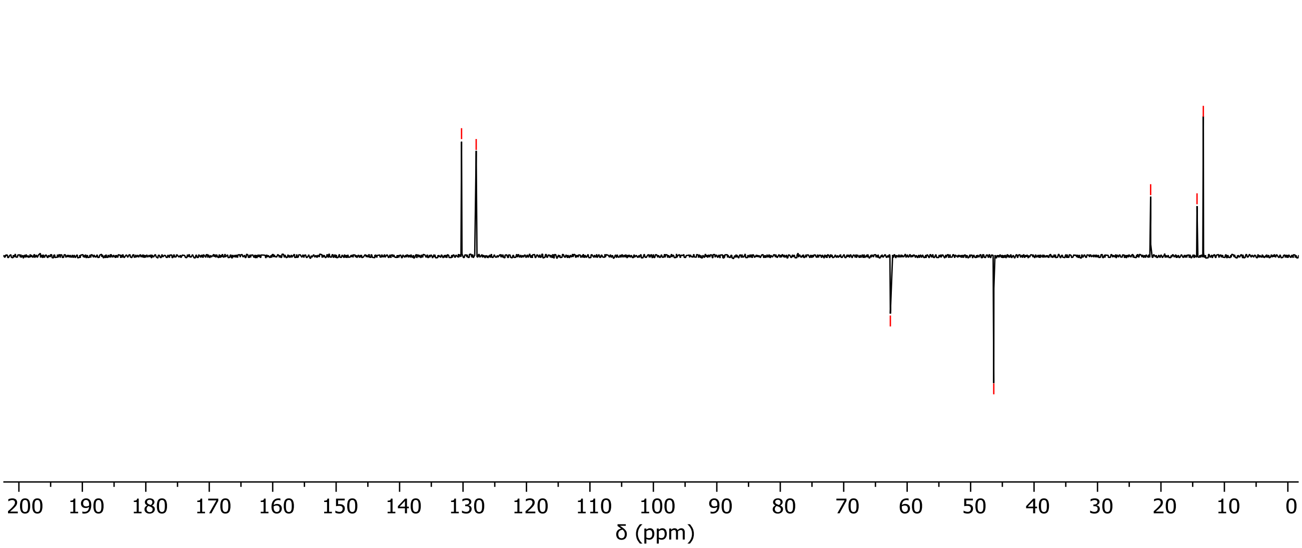

1H NMR (500 MHz, CDCl3)

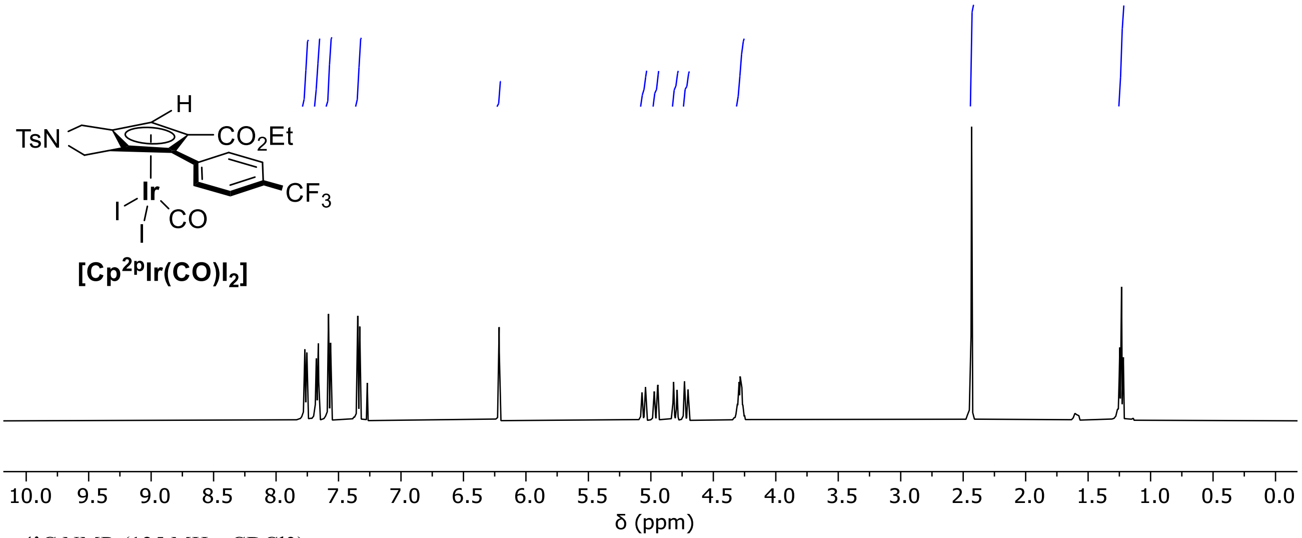

13C NMR (125 MHz, CDCl3)

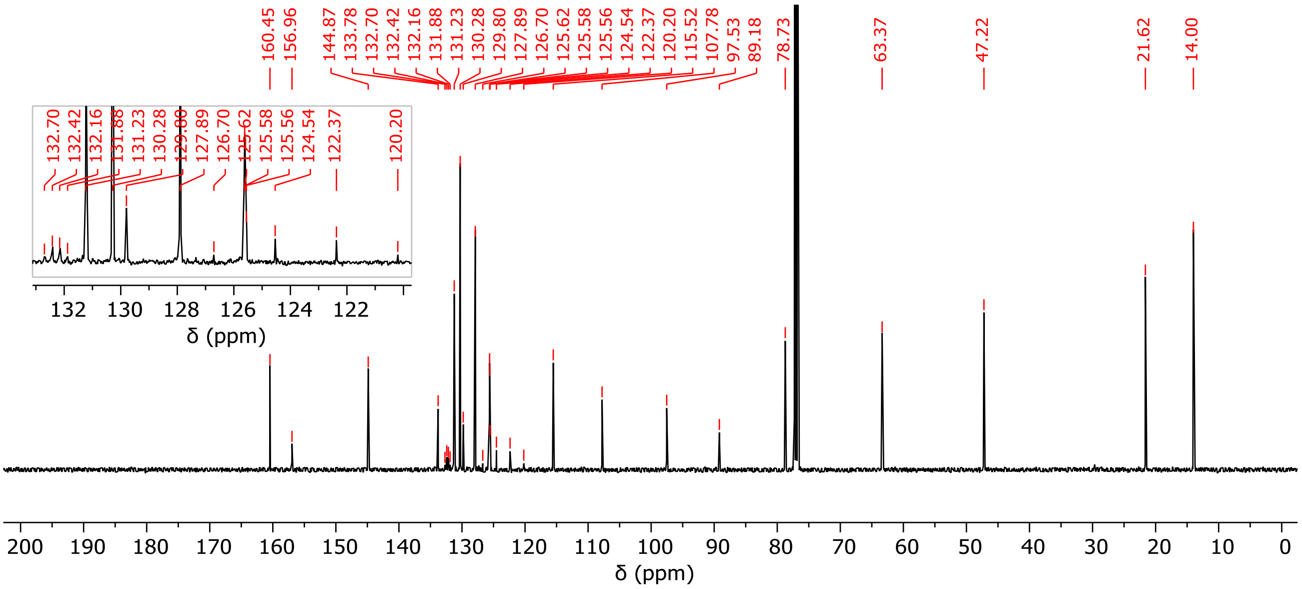

DEPT NMR (125 MHz, CDCl3)

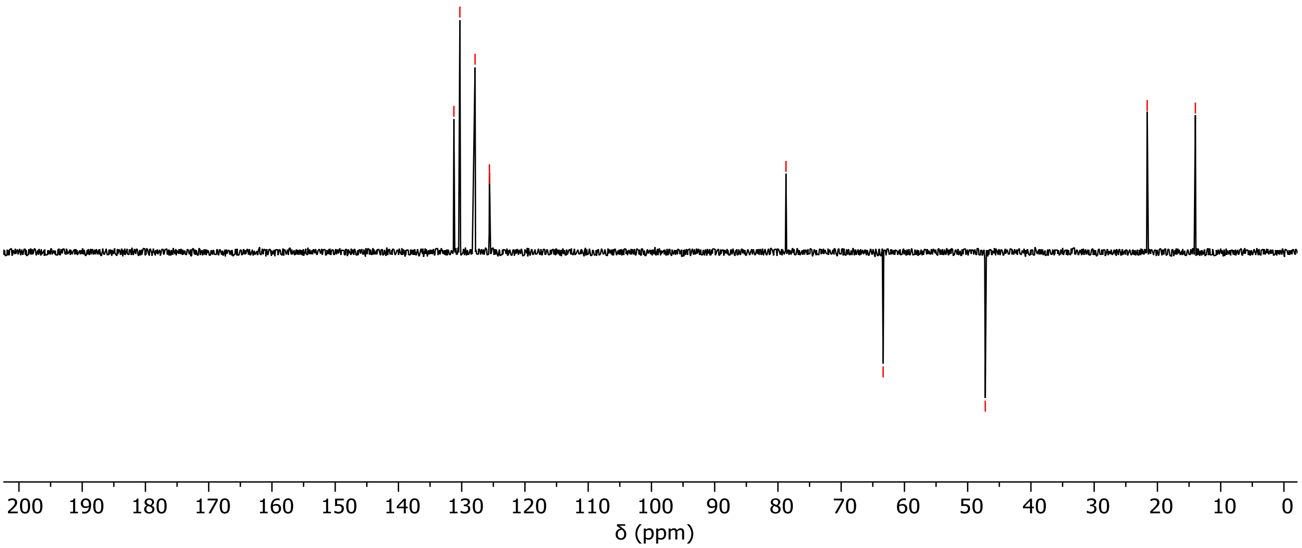

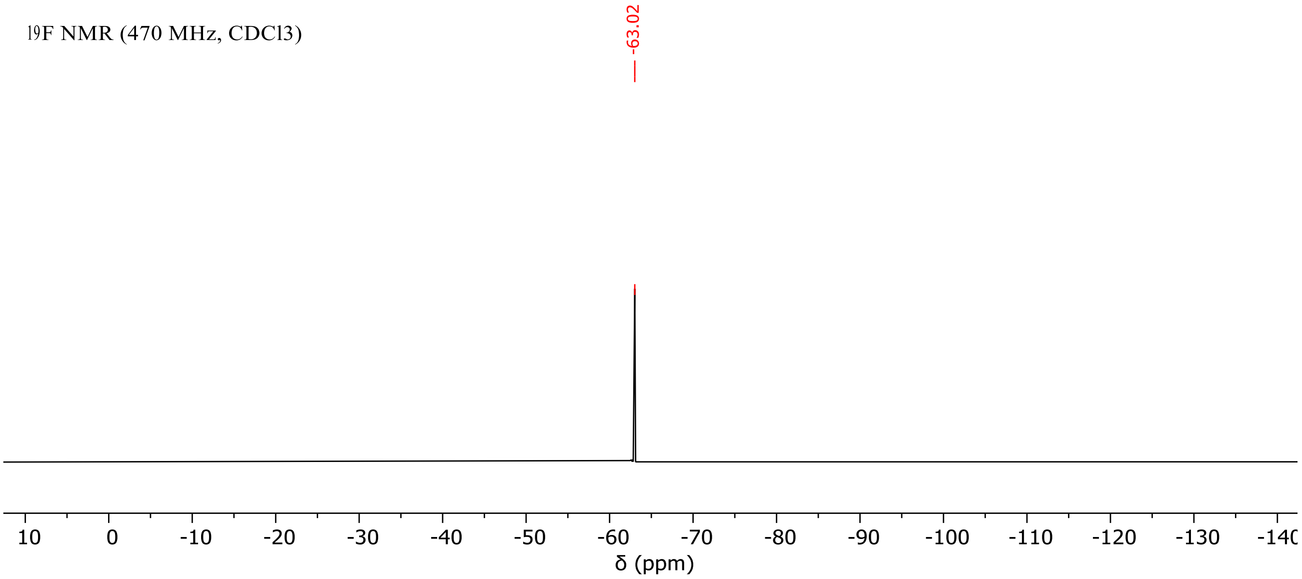
19F NMR (470 MHz, CDCl3)
-63.02
10
0
-10
-20
-30
-40
-50
-60
-70
-80
-90
-100
-110
-120
-130
-140
δ (ppm)

1H NMR (500 MHz, CDCl3)

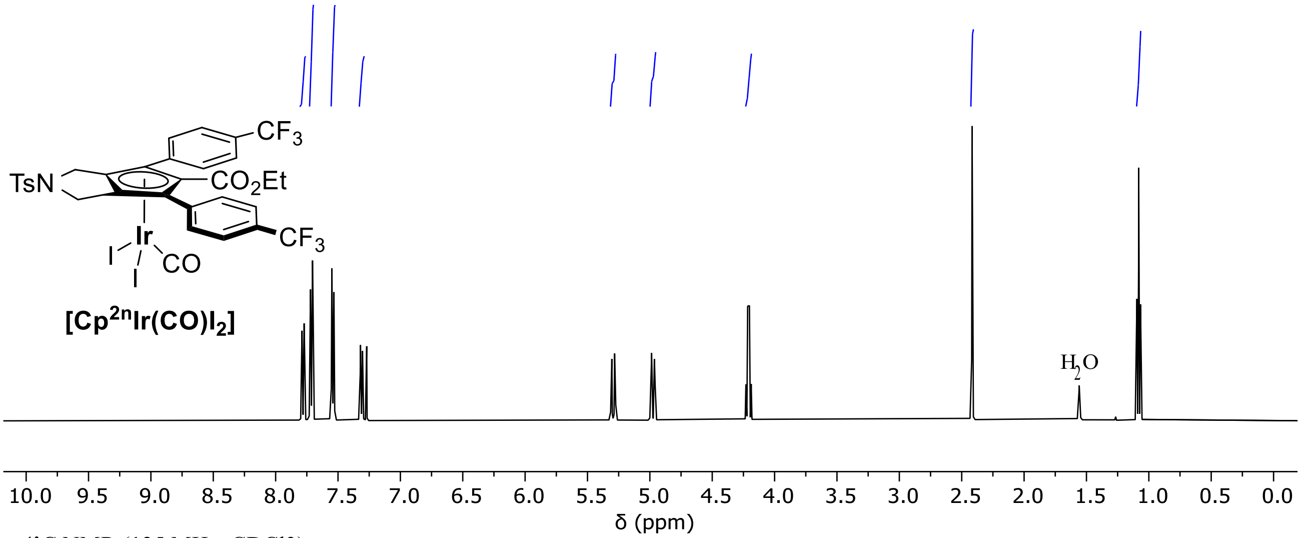

13C NMR (125 MHz, CDCl3)

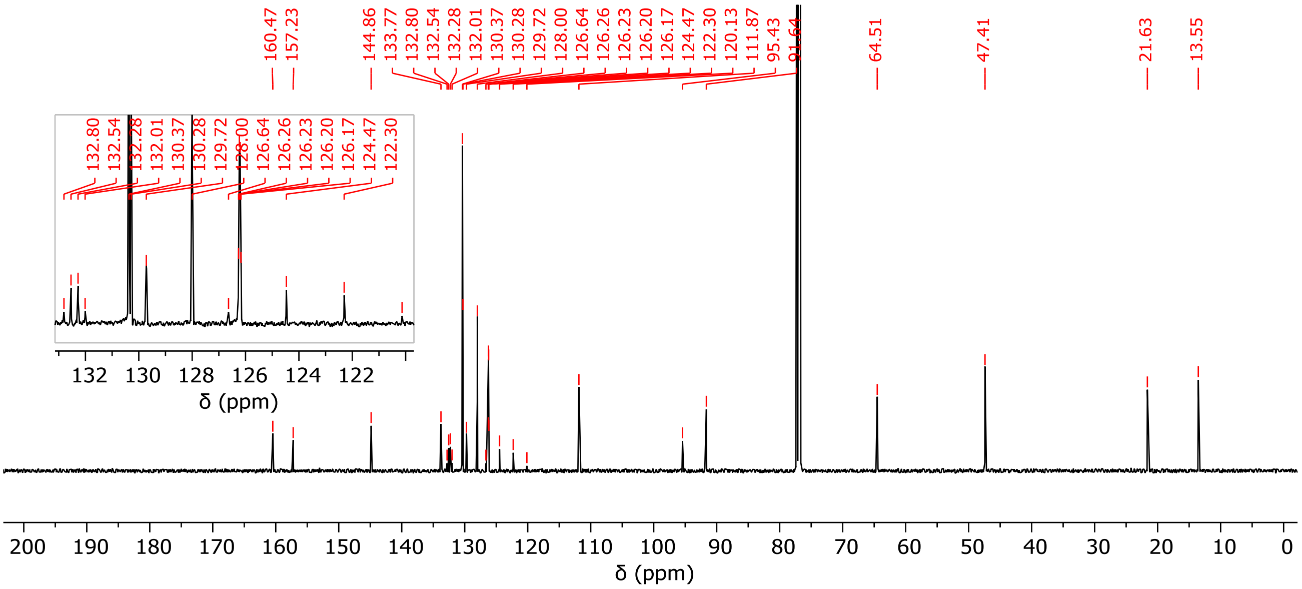

DEPT NMR (125 MHz, CDCl3)

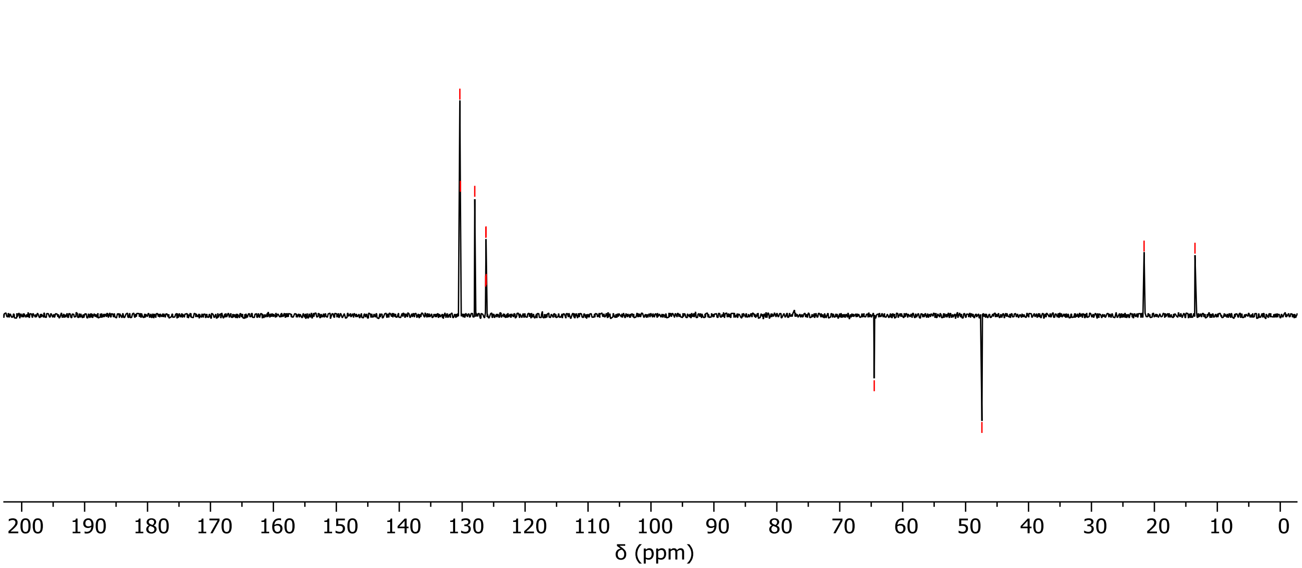

^{19}F NMR (470 MHz, $CDCl_3$)

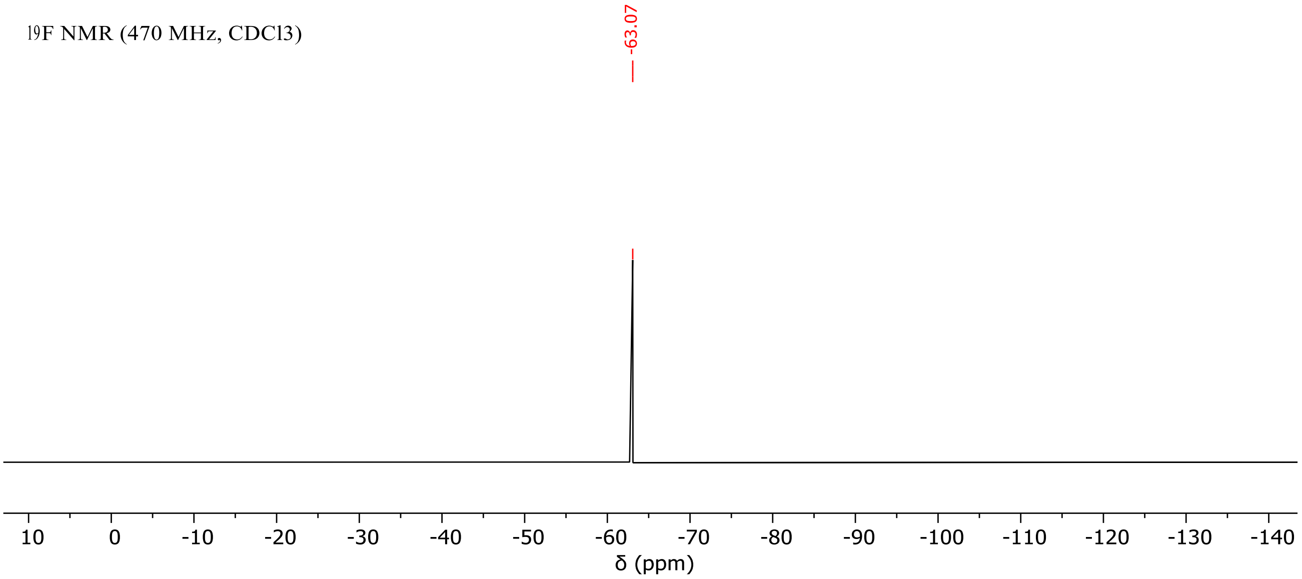

1H NMR (500 MHz, CDCl3)

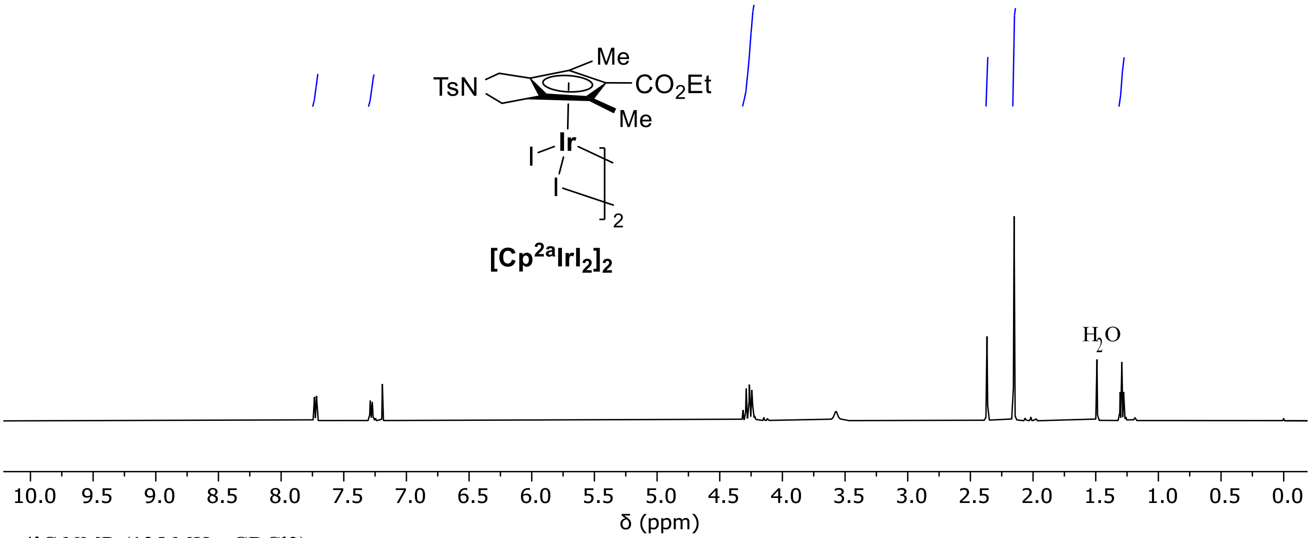

13C NMR (125 MHz, CDCl3)

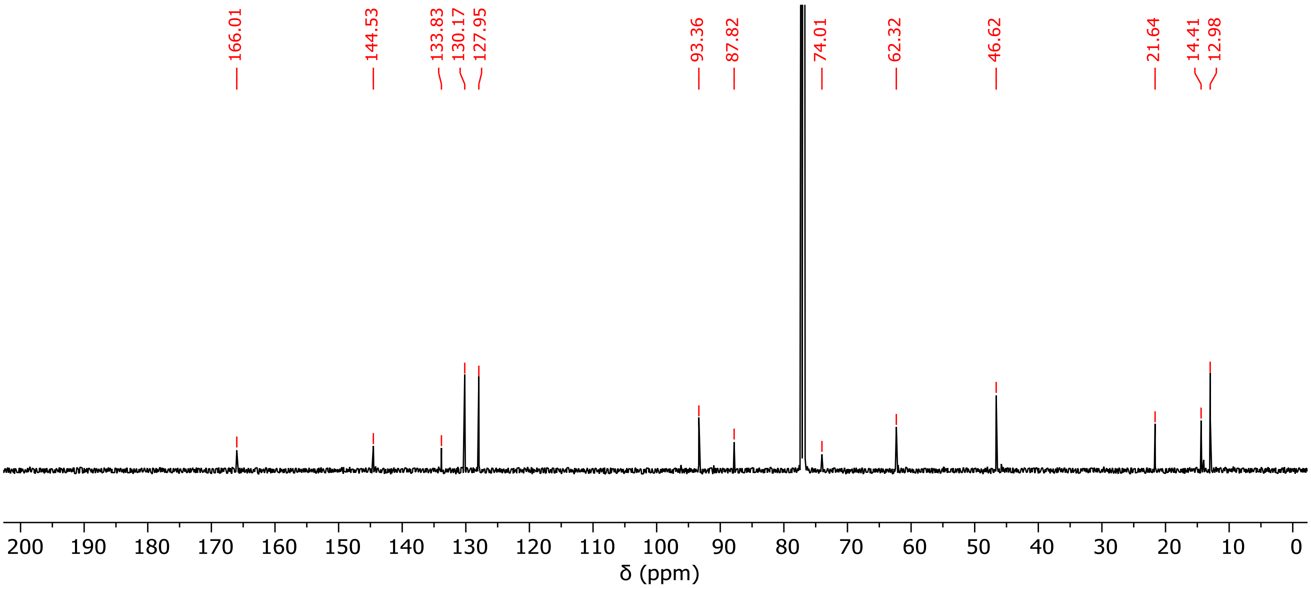

DEPT NMR (125 MHz, CDCl3)

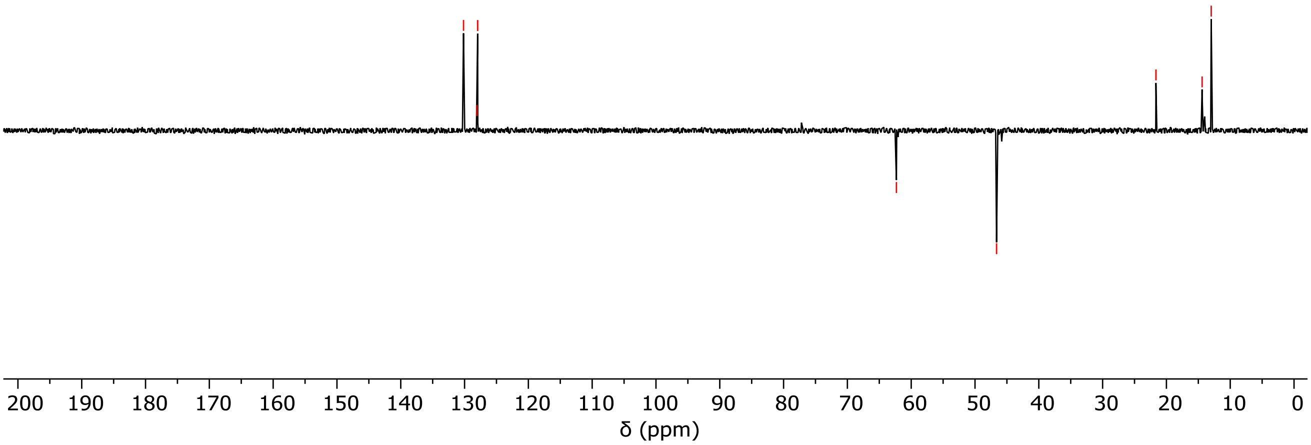

1H NMR (500 MHz, DMSO)

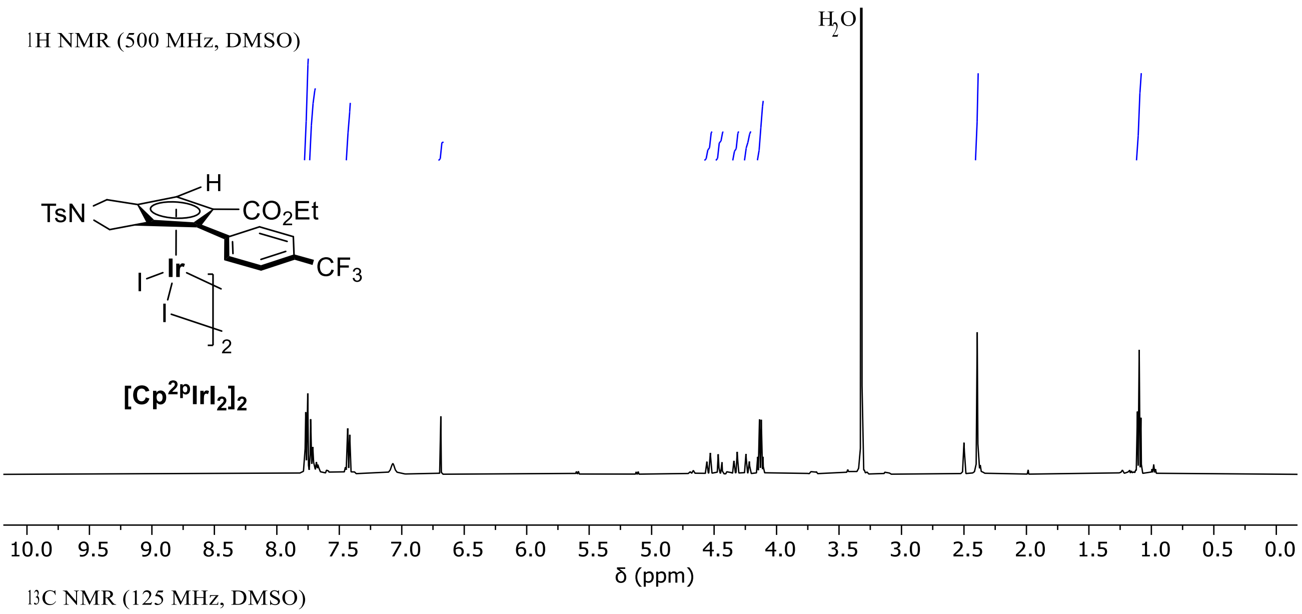

13C NMR (125 MHz, DMSO)

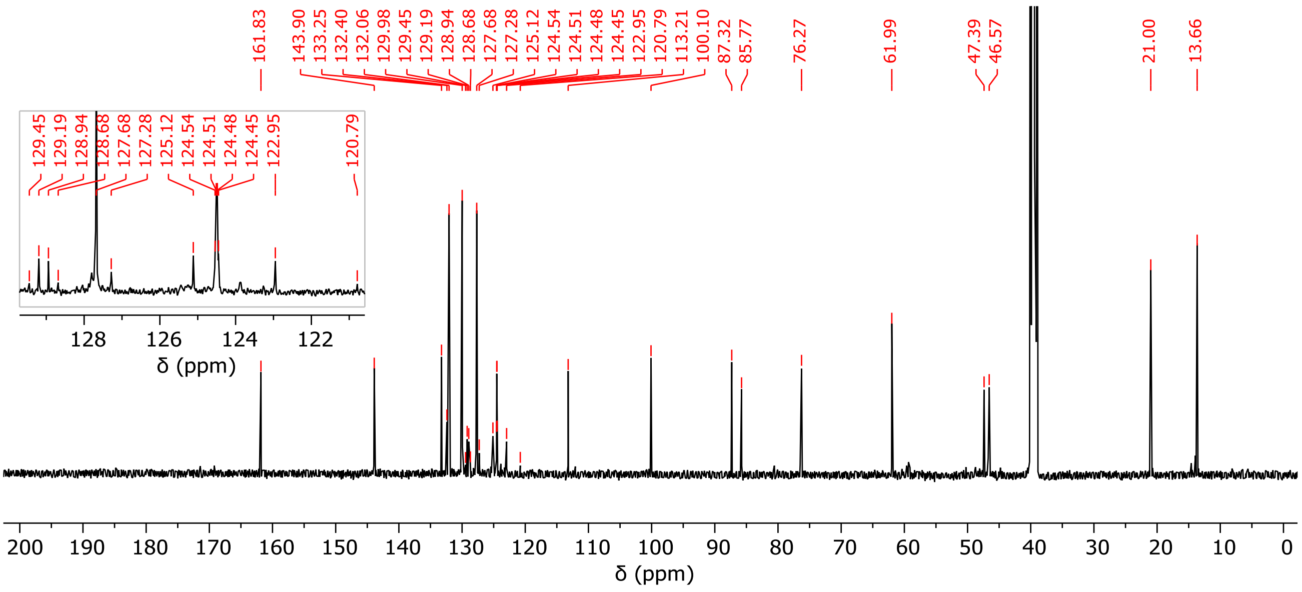

DEPT NMR (125 MHz, DMSO)

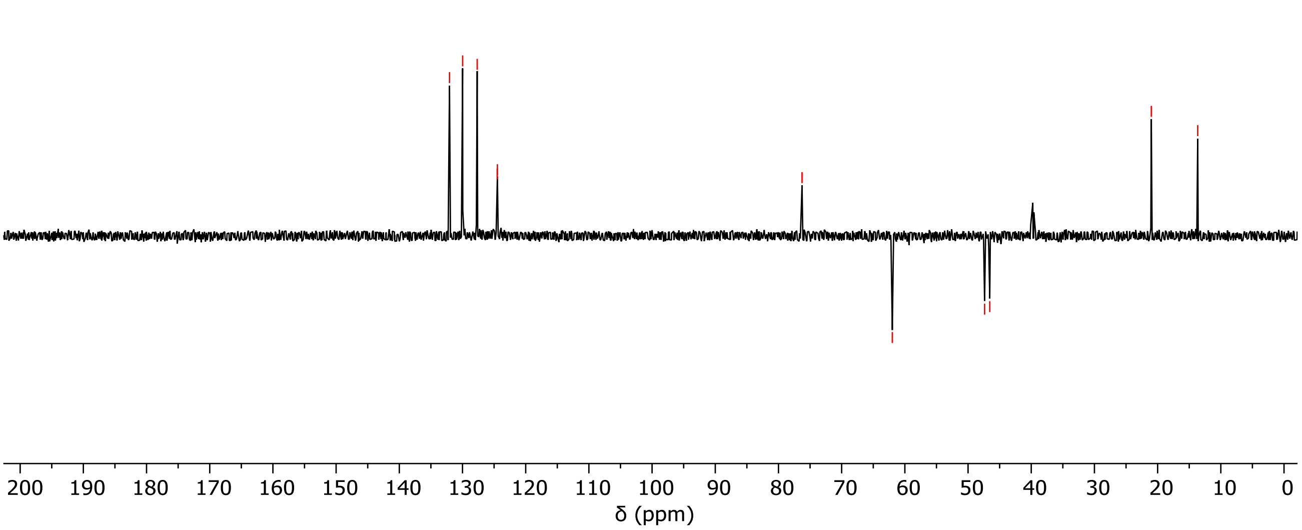

19F NMR (470 MHz, DMSO)

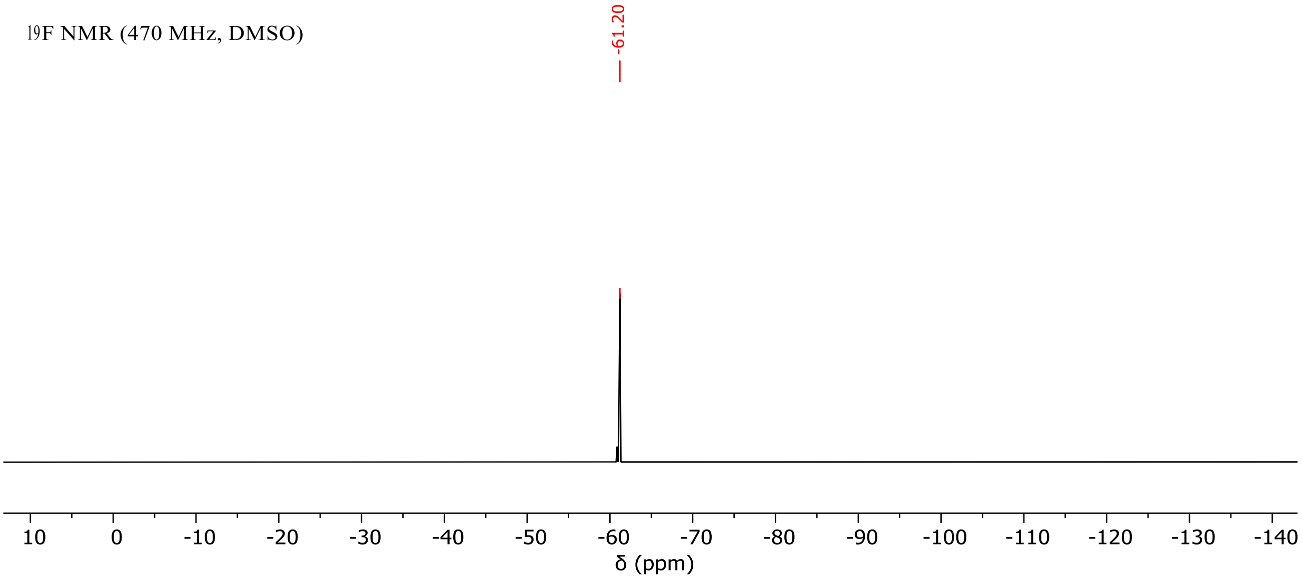

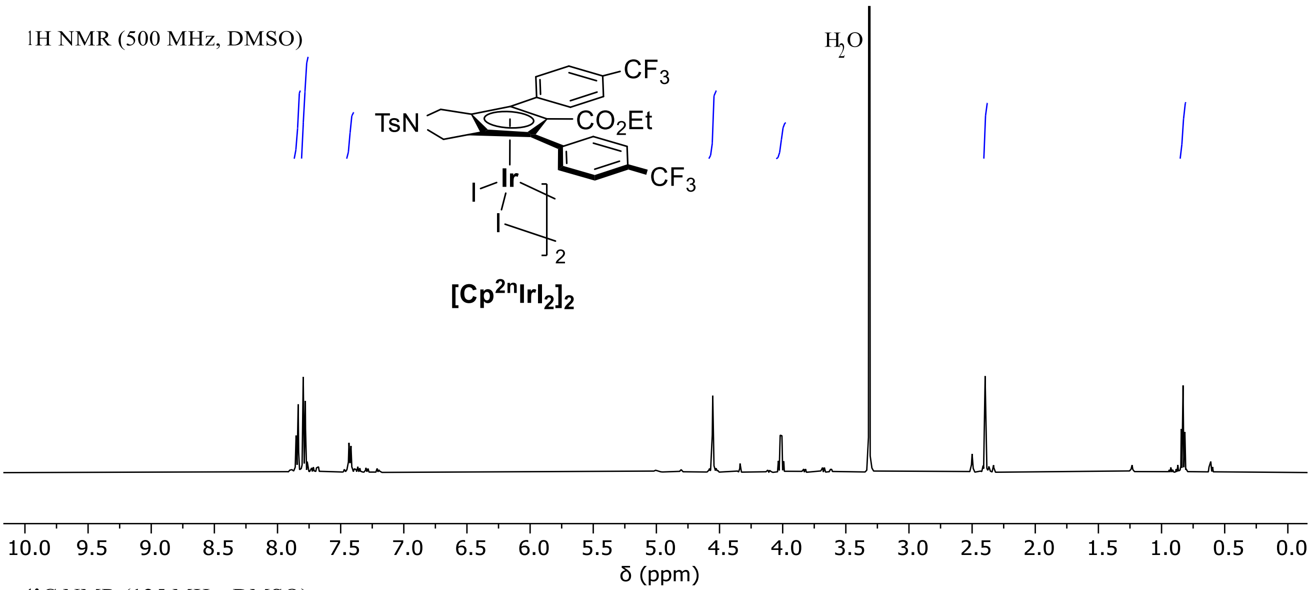
1H NMR (500 MHz, DMSO)
H2O
TsN
CF3
CO2Et
CF3
Ir
I
I
I
2
[Cp2nIrI2]2
10.0 9.5 9.0 8.5 8.0 7.5 7.0 6.5 6.0 5.5 5.0 4.5 4.0 3.5 3.0 2.5 2.0 1.5 1.0 0.5 0.0
δ (ppm)

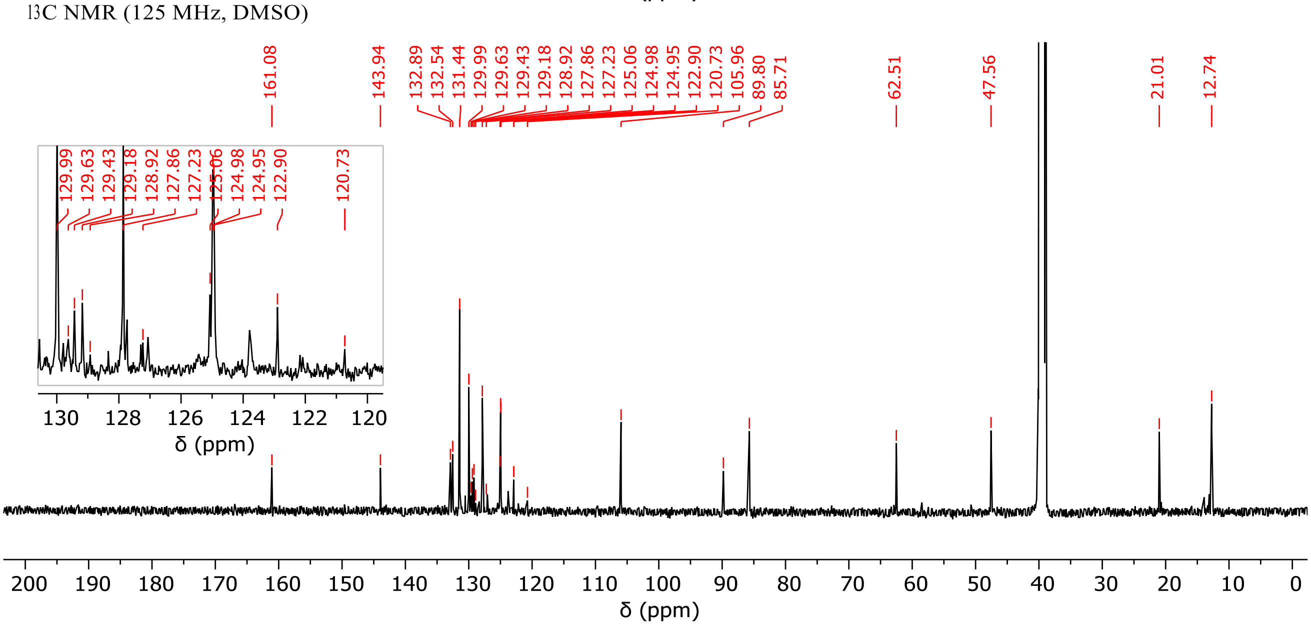
13C NMR (125 MHz, DMSO)
161.08
143.94
132.89
132.54
131.44
129.99
129.63
129.43
129.18
128.92
127.86
127.23
125.06
124.98
124.95
122.90
120.73
105.96
89.80
85.71
62.51
47.56
21.01
12.74
130 128 126 124 122 120
δ (ppm)
200 190 180 170 160 150 140 130 120 110 100 90 80 70 60 50 40 30 20 10 0
δ (ppm)

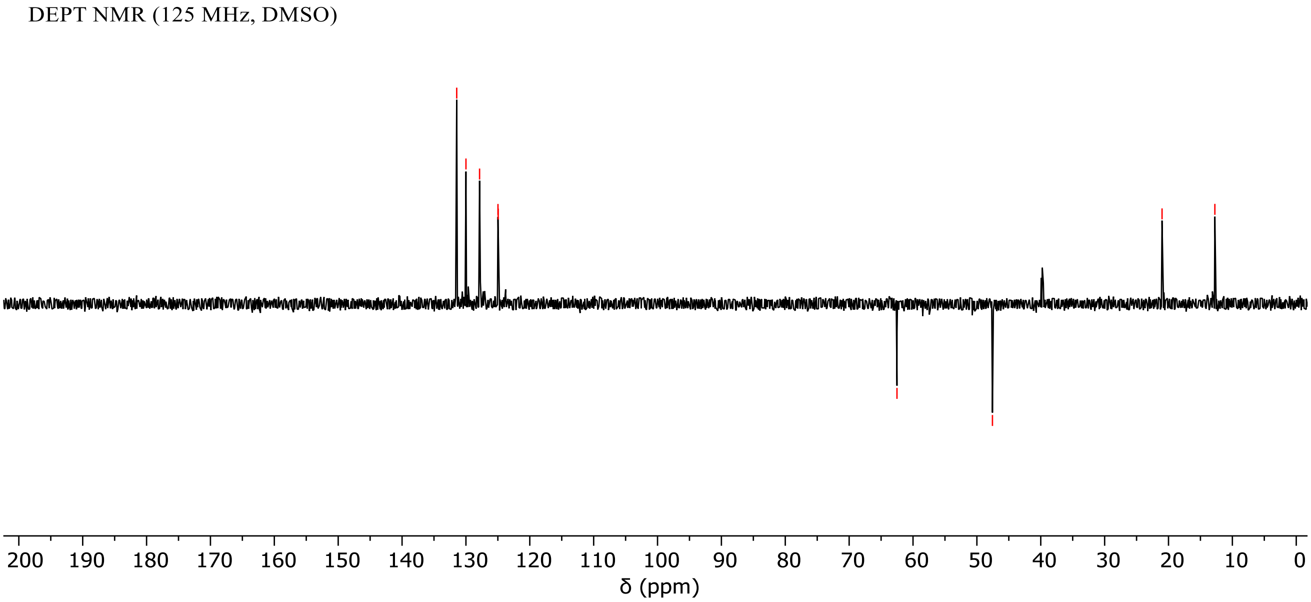
DEPT NMR (125 MHz, DMSO)
200 190 180 170 160 150 140 130 120 110 100 90 80 70 60 50 40 30 20 10 0
δ (ppm)

19F NMR (470 MHz, DMSO)

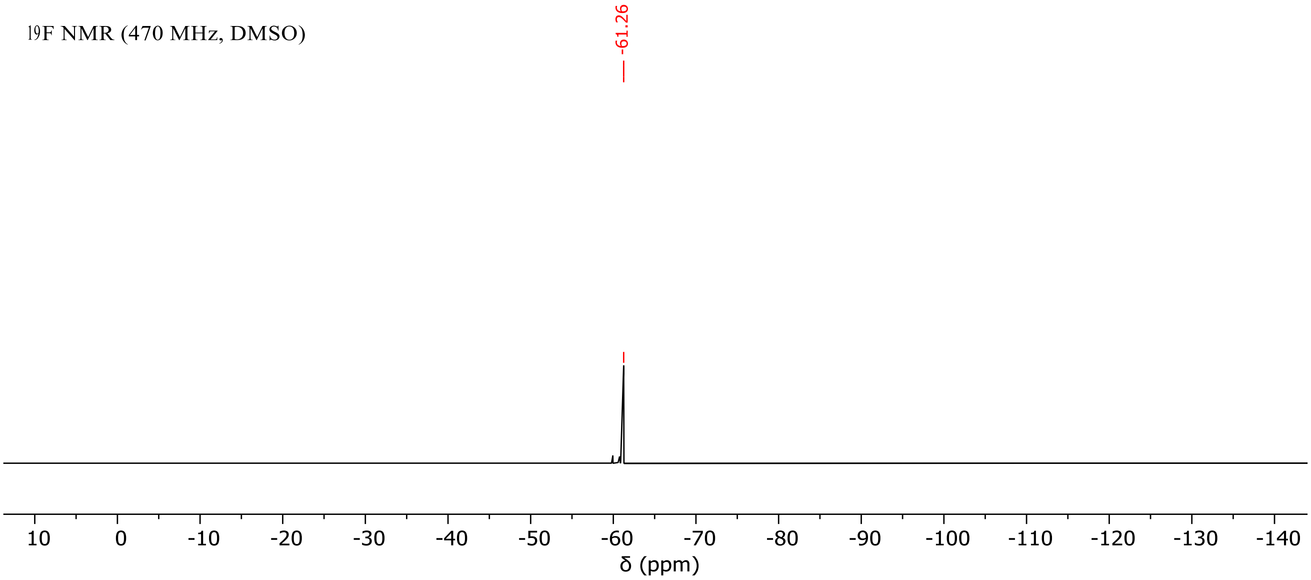

1H NMR (300 MHz, CDCl3)

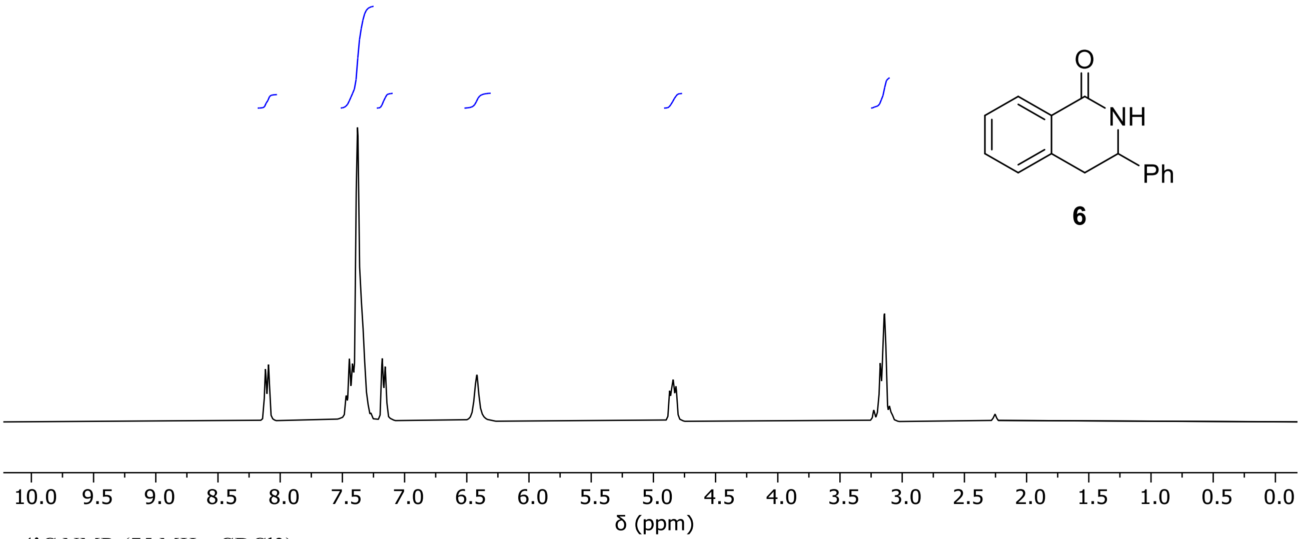

13C NMR (75 MHz, CDCl3)

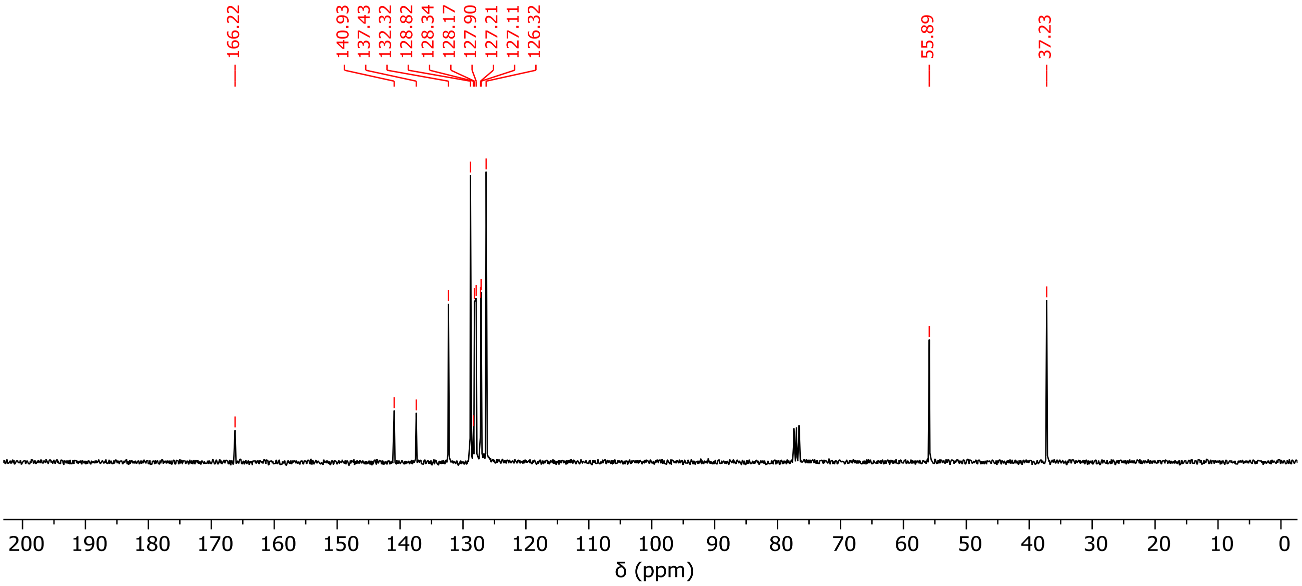

DEPT NMR (75 MHz, CDCl3)

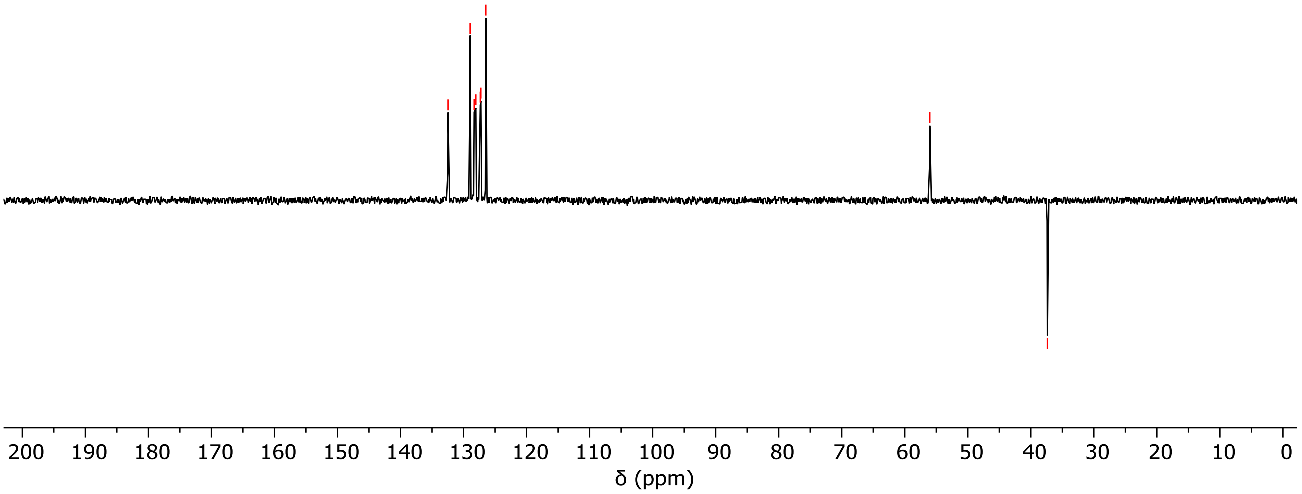

1H NMR (300 MHz, CDCl3)

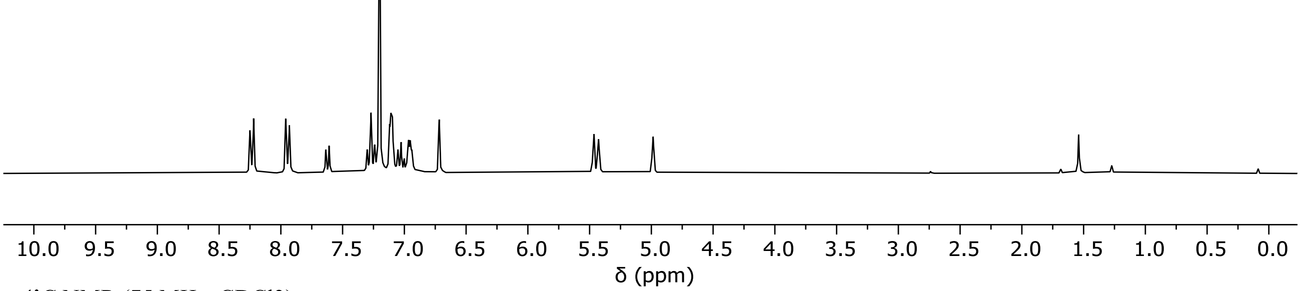

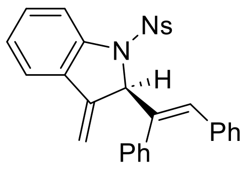

8

13C NMR (75 MHz, CDCl3)

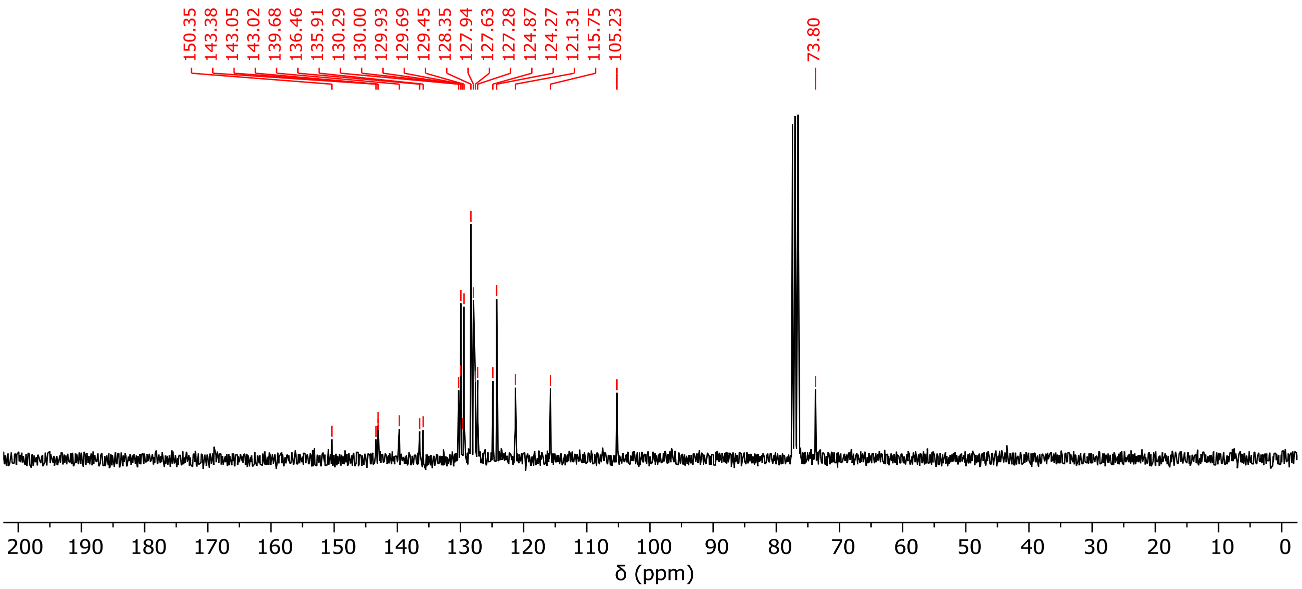

DEPT NMR (75 MHz, CDCl3)

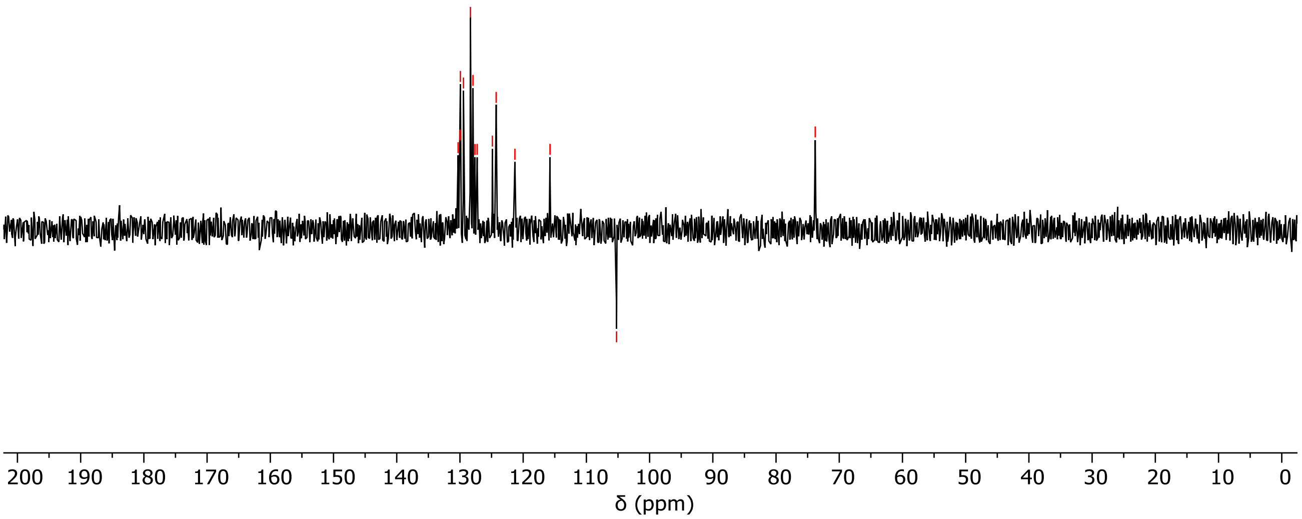

1H NMR (300 MHz, CDCl3)

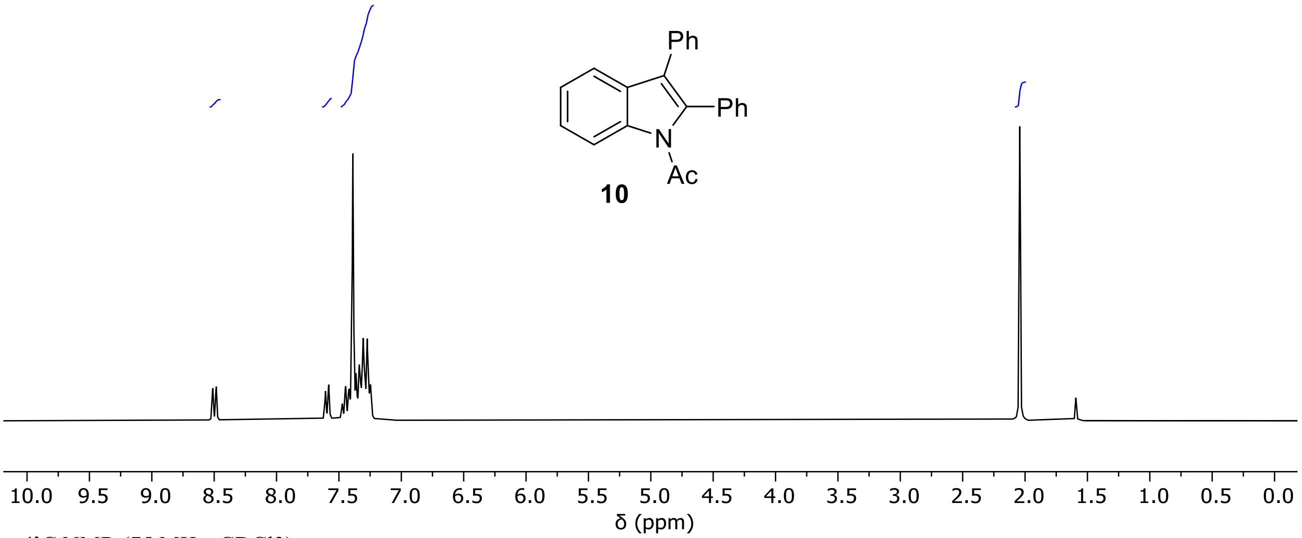

13C NMR (75 MHz, CDCl3)

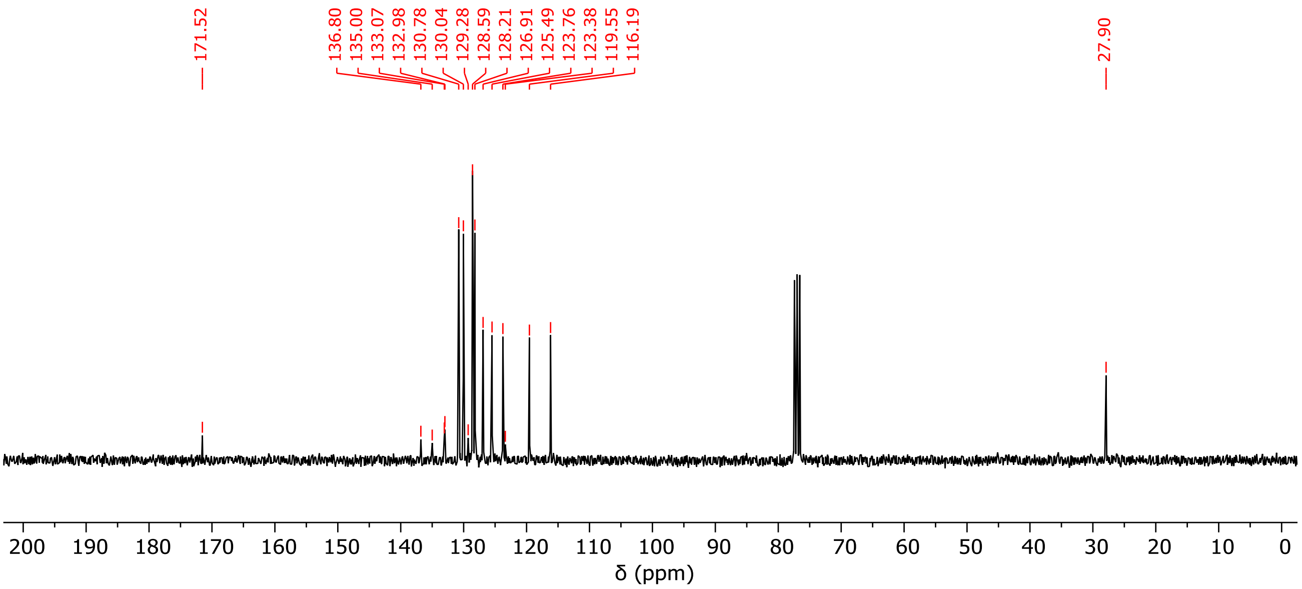

DEPT NMR (75 MHz, CDCl3)

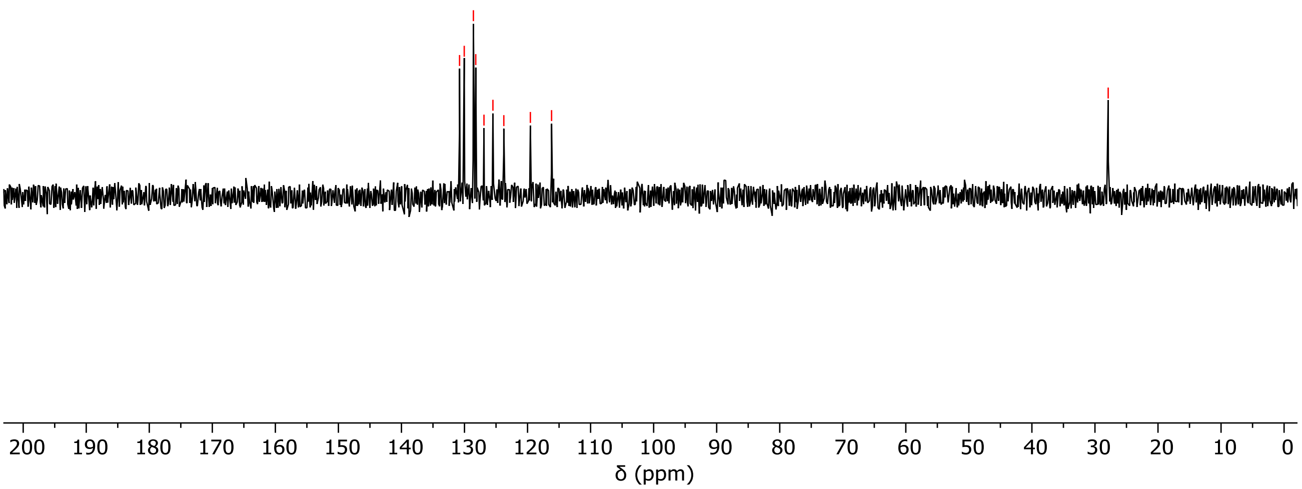

1H NMR (300 MHz, CDCl3)

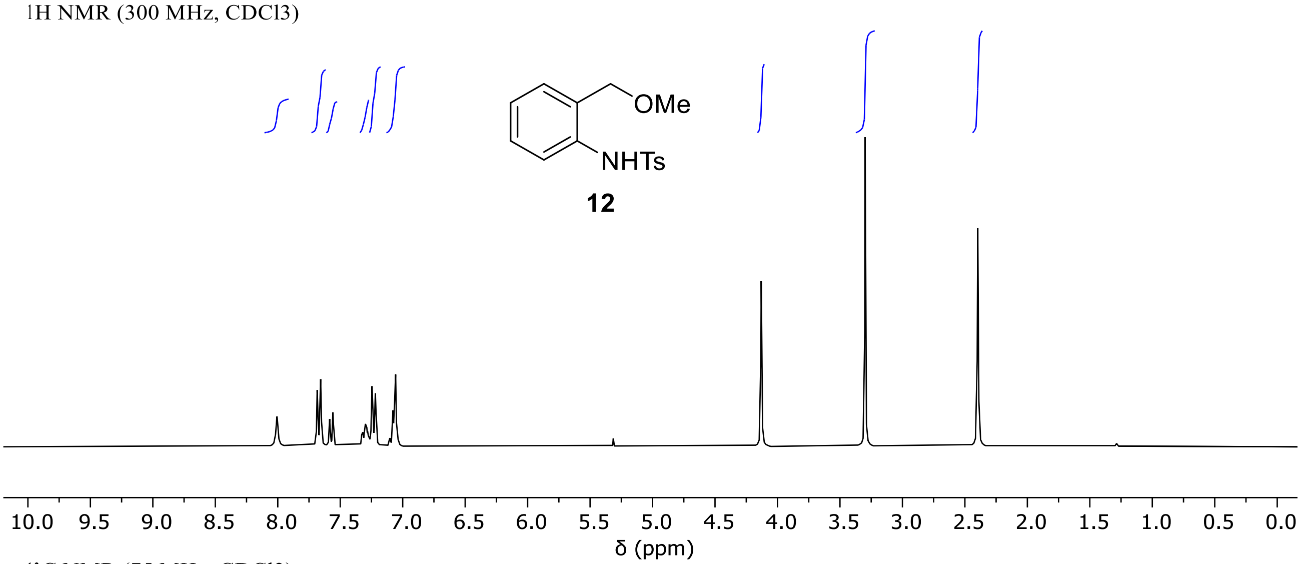

13C NMR (75 MHz, CDCl3)

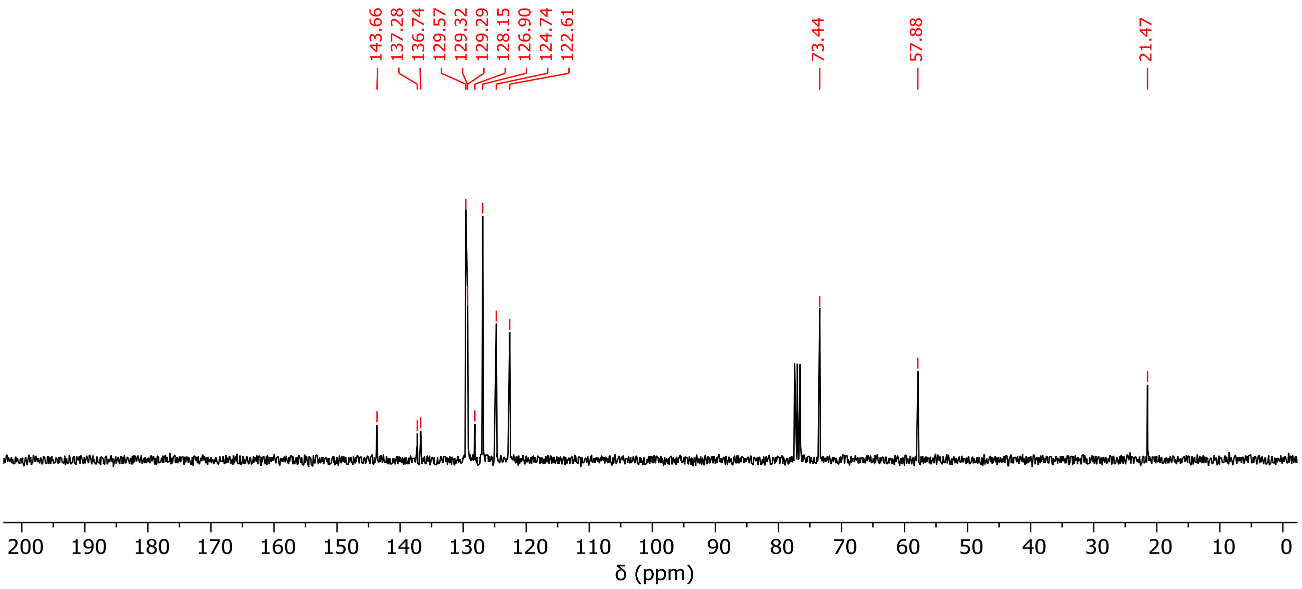

DEPT NMR (75 MHz, CDCl3)

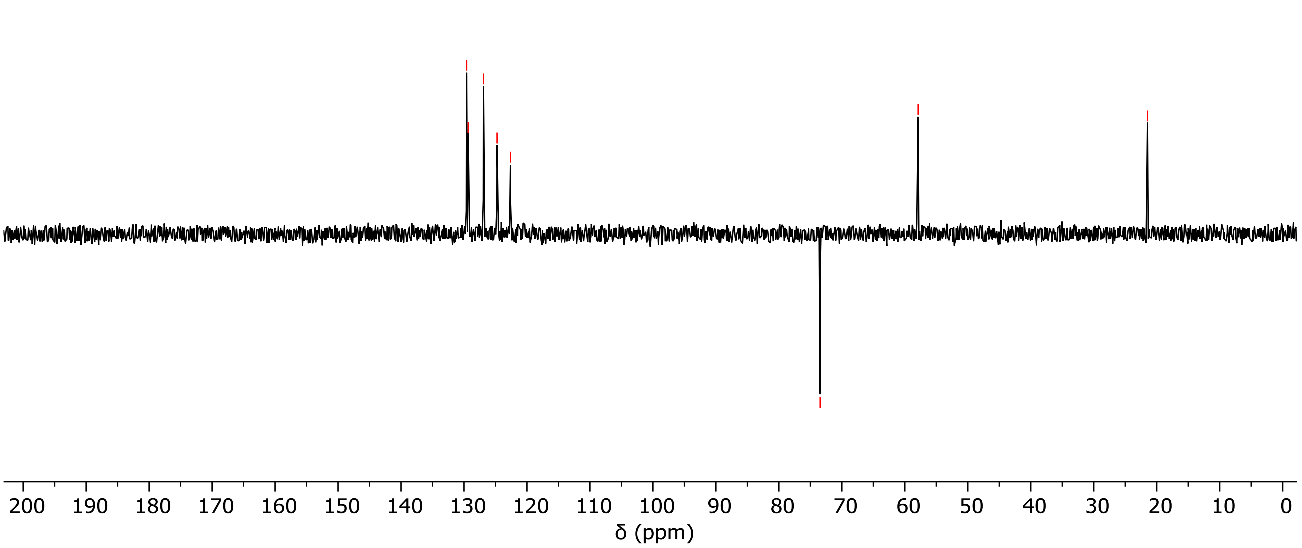

1H NMR (300 MHz, CDCl3)

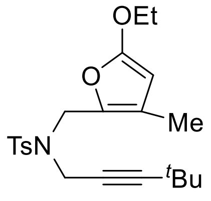

13c

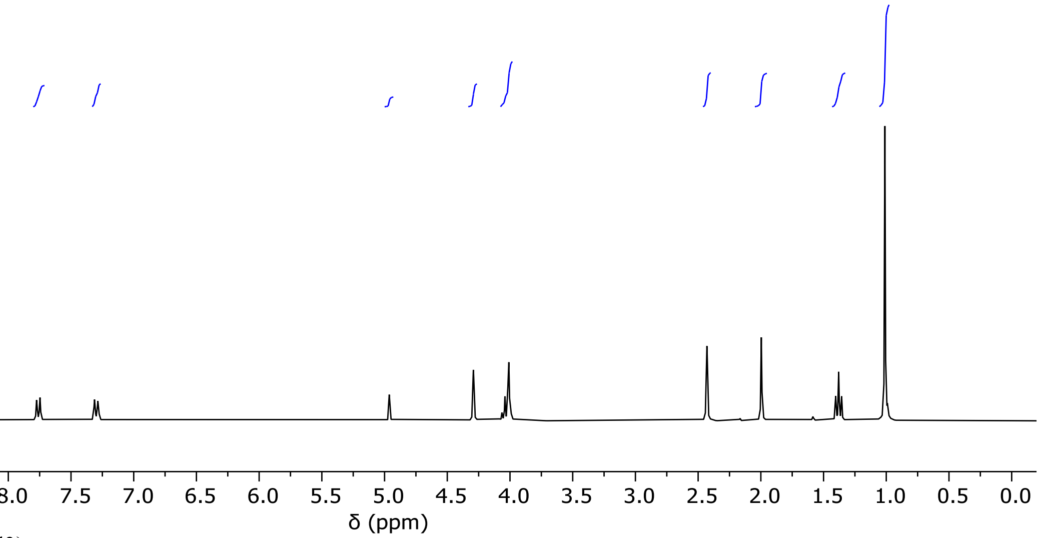

13C NMR (75 MHz, CDCl3)

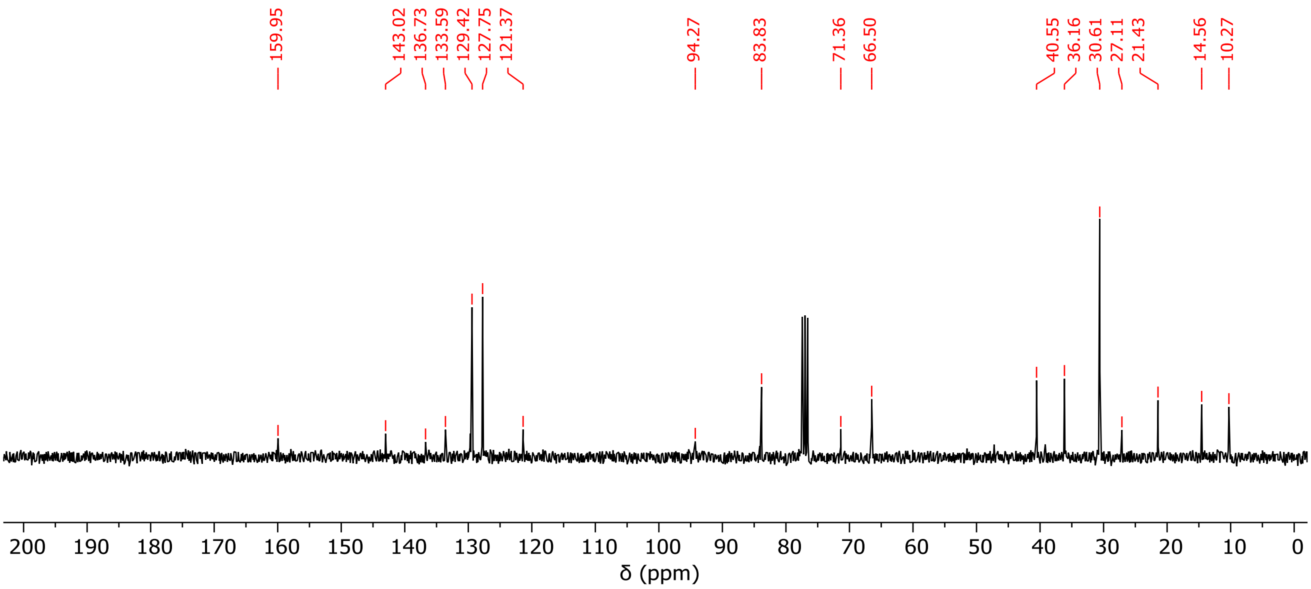

DEPT NMR (75 MHz, CDCl3)

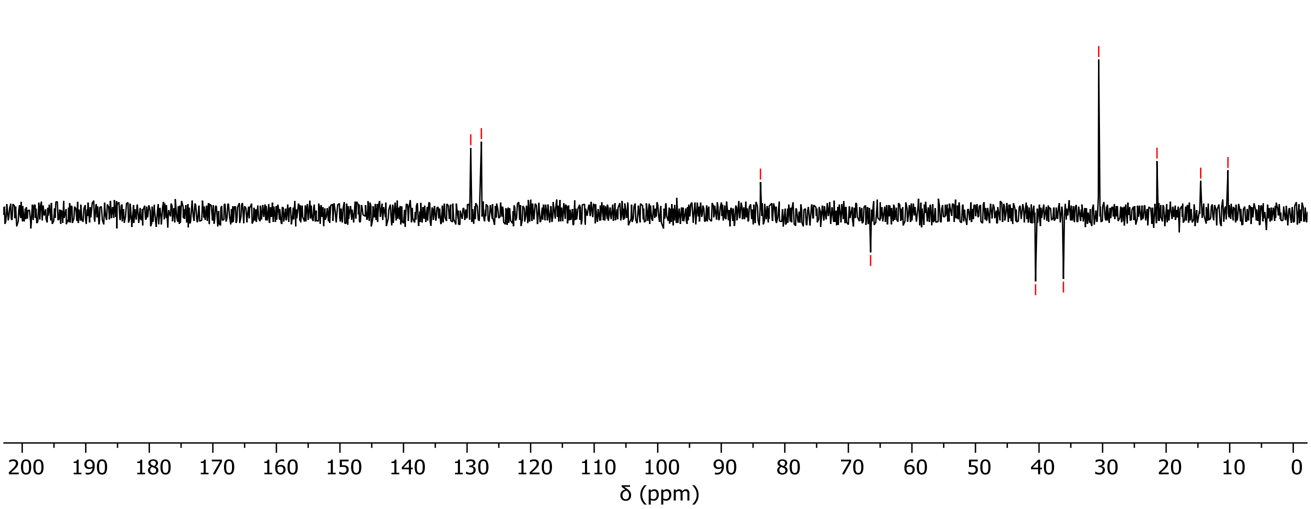

9. IR Study

IR spectra of $[Cp^RIr(CO)I_2]$ complexes

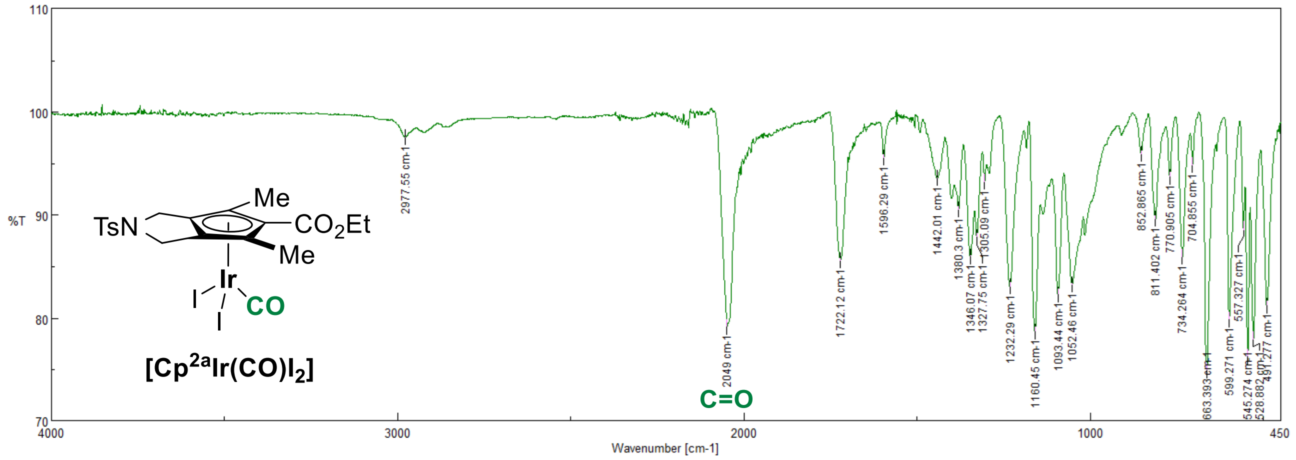

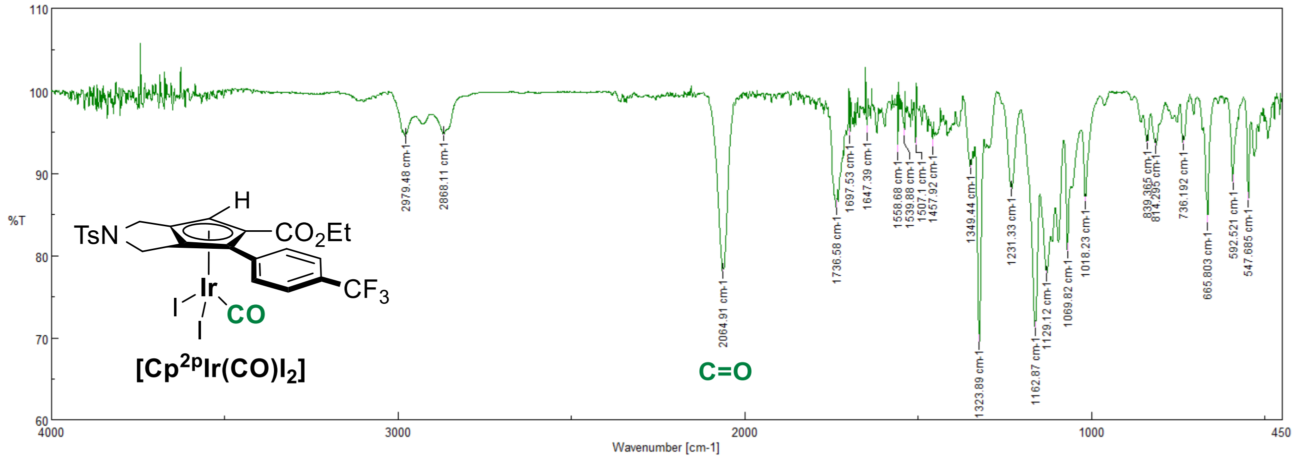

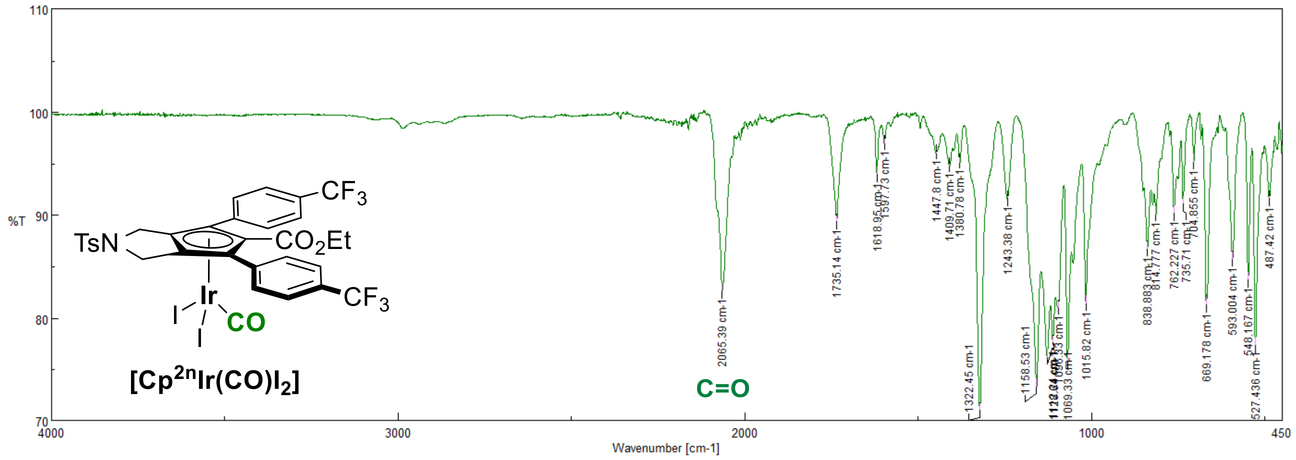

Comparative IR C=O (carbonyl) stretching frequencies of different $[CpIr(CO)I]_2$ complexes

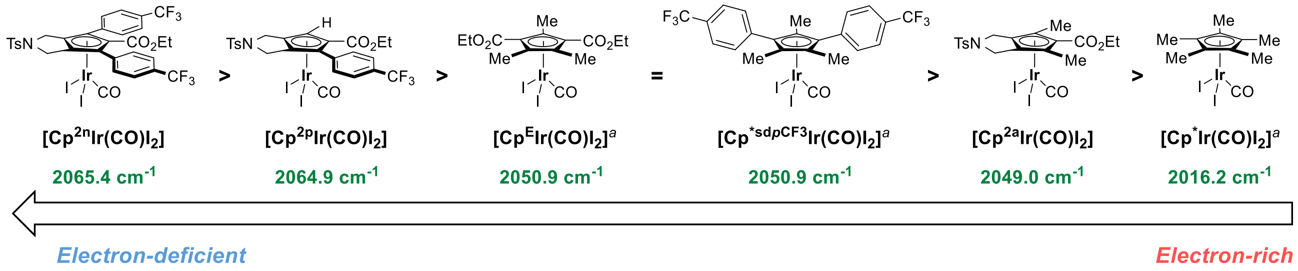

[a] C=O stretching frequencies values have been taken from Matsunaga's studies (see reference 8).

Higher CO stretching frequencies reflect weaker π-back donation compared to the $[Cp^*Ir(CO)I_2]$ complex, which supports the electron-deficient nature of the complexes developed in this work, being among the most electron-deficient Cps described to date.

10. References

[1] Dolomanov, O.V.; Bourhis, L. J.; Gildea, R. J.; Howard J. A. K.; Puschmann, H., OLEX2: A Complete Structure Solution, Refinement and Analysis Program. *J. Appl. Cryst.* **2009**, *42*, 339-341.

[2] Padwa A.; Kassir J. M.; Xu S. L., Rhodium-Catalyzed Ring-Opening Reaction of Cyclopropenes. Control of Regioselectivity by the Oxidation State of the Metal. *J. Org. Chem.* **1991**, *56*, 6971-6972.

[3] Kikuchi, T.; Yasui, T.; Yamamoto, Y., Cycloaddition of Cyclopropenes with Alkynes via Carbon–Carbon Double Bond Cleavage Enabled by a Ruthenium Catalyst: Synthesis of Cyclopentadienes and Cycloheptatrienes. *ACS Catal.* **2023,** *13*, 9656-9666.

[4] Li, G.; Ji, C.-L.; Hong, X.; Szostak, M., Highly Chemoselective, Transition-Metal-Free Transamidation of Unactivated Amides and Direct Amidation of Alkyl Esters by N–C/O–C Cleavage. *J. Am. Chem. Soc.* **2019**, *141*, 11161-11172.

[5] Liu, Q.; Li, M.; Xiong, R.; Mo, F., Direct Carboxylation of the Diazo Group ipso-C(sp^2)-H bond with Carbon Dioxide: Access to Unsymmetrical Diazomalonates and Derivatives. *Org. Lett.* **2017**, *19*, 6756-6759.

[6] Yan, X.; Jiang, J.; Wang, J., A Class of Readily Tunable Planar-Chiral Cyclopentadienyl Rhodium(III) Catalysts for Asymmetric C-H Activation. *Angew. Chem. Int. Ed.* **2022,** *61*, e202201522.

[7] Audic, B.; Wodrich, M. D.; Cramer, N., Mild Complexation Protocol for Chiral Cp^xRh and Ir Complexes Suitable for *in situ* Catalysis. *Chem. Sci.* **2019,** *10*, 781-787.

[8] Tomita, E.; Kojima, M.; Nagashima, Y.; Tanaka, K.; Sugiyama, H.; Segawa, Y.; Furukawa, A.; Maenaka, K.; Maeda, S.; Yoshino, T.; Matsunaga, S., An Electron-Deficient Cp^E Iridium(III) Catalyst: Synthesis, Characterization, and Application to Ether-Directed C−H Amidation. *Angew. Chem. Int. Ed.* **2023**, e202301259.

[9] Rakshit, S.; Grohmann, C.; Besset, T.; Glorius, F., Rh(III)-Catalyzed Directed C−H Olefination Using an Oxidizing Directing Group: Mild, Efficient, and Versatile. *J. Am. Chem. Soc.* **2011,** *133*, 2350-2353.

[10] Font, M.; Cendon, B.; Seoane, A.; Mascarenas, J. L.; Gulias, M., Rhodium(III)-Catalyzed Annulation of 2-Alkenyl Anilides with Alkynes through C-H Activation: Direct Access to 2-Substituted Indolines. *Angew. Chem. Int. Ed.* **2018,** *57*, 8255-8259.

[11] Hoshino, Y.; Shibata, Y.; Tanaka, K., Oxidative Annulation of Anilides with Internal Alkynes Using an (Electron-Deficient η5-Cyclopentadienyl)Rhodium(III) Catalyst Under Ambient Conditions. *Adv. Synth. Catal.* **2014,** *356*, 1577-1585.

[12] Gaussian 09, Revision D.01, M. J. Frisch, G. W. Trucks, H. B. Schlegel, G. E. Scuseria, M. A. Robb, J. R. Cheeseman, G. Scalmani, V. Barone, G. A. Petersson, H. Nakatsuji, X. Li, M. Caricato, A. Marenich, J. Bloino, B. G. Janesko, R. Gomperts, B. Mennucci, H. P. Hratchian, J. V. Ortiz, A. F. Izmaylov, J. L. Sonnenberg, D. Williams-Young, F. Ding, F. Lipparini, F. Egidi, J. Goings, B. Peng, A. Petrone, T. Henderson, D. Ranasinghe, V. G. Zakrzewski, J. Gao, N. Rega, G. Zheng, W. Liang, M. Hada, M. Ehara, K. Toyota, R. Fukuda, J. Hasegawa, M. Ishida, T. Nakajima, Y. Honda, O. Kitao, H. Nakai, T. Vreven, K.

Throssell, J. A. Montgomery, Jr., J. E. Peralta, F. Ogliaro, M. Bearpark, J. J. Heyd, E. Brothers, K. N. Kudin, V. N. Staroverov, T. Keith, R. Kobayashi, J. Normand, K. Raghavachari, A. Rendell, J. C. Burant, S. S. Iyengar, J. Tomasi, M. Cossi, J. M. Millam, M. Klene, C. Adamo, R. Cammi, J. W. Ochterski, R. L. Martin, K. Morokuma, O. Farkas, J. B. Foresman and D. J. Fox, Gaussian, Inc., Wallingford CT, **2009**.

[13] (a) Becke, A. D. Density-functional thermochemistry. III. The role of exact exchange. *J. Chem. Phys*. **1993**, *98*, 5648-5652; (b) Lee, C.; Yang, W.; Parr, R. G., Development of the Colic-Salvetti correlation-energy formula into a functional of the electron density. *Phys. Rev. B* **1988**, *37*, 785-789; (c) Vosko, S. H.; Wilk, L.; Nusair, M., Accurate Spin-Dependent Electron Liquid Correlation Energies for Local Spin Density Calculations: A Critical Analysis. *Can. J. Phys*. **1980**, *58*, 1200-1211.

[14] Grimme, S.; Antony, J.; Ehrlich, S.; Krieg, H., A Consistent and Accurate *ab initio* Parametrization of Density Functional Dispersion Correction (DFT-D) for the 94 Elements H-Pu. *J. Chem. Phys*. **2010**, *132*, 154104.

[15] Weigend, F.; Ahlrichs, R., Balanced Basis Sets of Split Valence, Triple Zeta Valence and Quadruple Zeta Valence Quality for H to Rn: Design and Assessment of Accuracy. *Phys. Chem. Chem. Phys*. **2005**, *7*, 3297-3305.

[16] (a) Miertuš, S.; Scrocco, E.; Tomasi, J., Electrostatic Interaction of a Solute with a Continuum. A Direct Utilizaion of AB initio Molecular Potentials for the Prevision of Solvent Effects. *Chem. Phys*. **1981**, *55*, 117-129; (b) Pascual-Ahuir, J. L.; Silla, E.; Tuñón, I., GEPOL: An Improved Description of Molecular Surfaces. III. A New Algorithm for the Computation of a Solvent-Excluding Surface. *J. Comput. Chem.* **1994**, *15*, 1127-1138; (c) Barone, V.; Cossi, M., Quantum Calculation of Molecular Energies and Energy Gradients in Solution by a Conductor Solvent Model. *J. Phys. Chem. A* **1998**, *102*, 1995-2001.

[17] McIver, J. W.; Komornicki, A. K., Structure of Transition States in Organic Reactions. General Theory and an Application to the Cyclobutene-Butadiene Isomerization Using a Semiempirical Molecular Orbital Method. *J. Am. Chem. Soc.* **1972**, *94*, 2625-2633.

[18] González, C.; Schlegel, H. B., Reaction Path Following in Mass-Weighted Internal Coordinates. *J. Phys. Chem*. **1990**, *94*, 5523-5527.

[19] Da Concepción, E.; Fernández, I.; Mascareñas, J. L.; López, F., Highly Enantioselective Cobalt-Catalyzed (3+2) Cycloadditions of Alkynylidenecyclopropanes. *Angew. Chem. Int. Ed*. **2021**, *60*, 8182-8188.

[20] Grimme, S., Supramolecular Binding Thermodynamics by Dispersion-Corrected Density Functional Theory. *Chem. Eur. J*. **2012**, *18*, 9955-9964.

[21] Luchini, G. Alegre-Requena, J. V.; Funes-Ardoiz, I. Paton R. S., Goodvibes: Automated Thermochemistry for Heterogeneous Computational Chemistry Data. *F1000Research* **2020**, *9*, 291.
